# Biguanide Complexes of Boron and Aluminum

**DOI:** 10.1021/acs.inorgchem.6c00595

**Published:** 2026-06-04

**Authors:** Lukáš Vlk, Tomáš Chlupatý, Alena Hoffmannová, Zdeňka Růžičková, Aleksandra Szymańska, Benjamin Théron, Raluca Malacea-Kabbara, Pierre Le Gendre, Jędrzej Walkowiak, Aleš Růžička

**Affiliations:** † Department of General and Inorganic Chemistry, Faculty of Chemical Technology, 48252 University of Pardubice, Studentská 573, Pardubice 532 10, Czech Republic; ‡ Univ. Bourgogne Europe, 131788 CNRS, ICMUB UMR 6302, 9 Avenue Alain Savary, 21000 Dijon, France; § Center for Advanced Technologies, Adam Mickiewicz University, Uniwersytetu Poznańskiego 10, Poznań 61-614, Poland; ∥ Faculty of Chemistry, Adam Mickiewicz University, Uniwersytetu Poznańskiego 8, Poznań 61-614, Poland

## Abstract

In search of an alternative to the dominant industrial processes, facilitated mostly by transition-metal catalytic systems, current economic and environmental aspects are leading to a growing interest in the chemistry and application of main group element-based catalysts and materials. Mono- and dinuclear boron and aluminum complexes bearing a biguanide ligand (substituted 4,6-dimethoxypyrimidin-2-yl-guanidinate; L) were prepared by deprotonation of **LH_2_** with BH_3_·Me_2_S, BF_3_·Et_2_O, Me_3_Al, or Me_2_AlCl or, in some cases, by transmetallation reactions of the lithium complex. Complexes were characterized by multinuclear NMR and *sc*-XRD analyses. Two coordination isomers, where the central atoms can occur both in the four- and six-membered *N*,*N*′-chelate ring, were found for homobimetallic aluminum and boron biguanide complexes. A thorough study was performed to understand the formation and isomerization process, where thermally and kinetically conducted isomerization was explored. The relative Gibbs free energies of optimized structures and hypothetical arrangements were calculated and compared in order to support the described isomerization. The activity of selected complexes was tested in the ring-opening polymerization (ROP) of *rac*-lactide. While the boron complexes are inactive, dimethylaluminum species show moderate activity and good control over the polymer mass and dispersity after ^*i*^PrOH co-initiation. Furthermore, the species effectively promote hydroboration of unsaturated C–C bonds.

## Graphical abstract

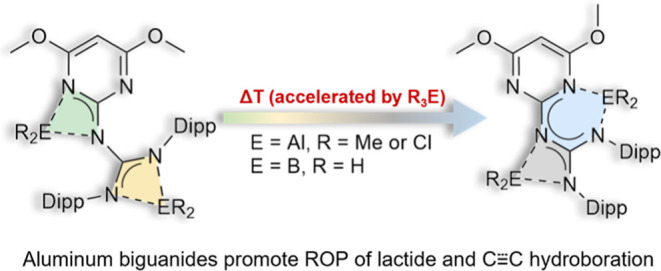

## Introduction

Electron-deficient Group 13 elements such as boron and aluminum are at the center of interest in inorganic chemistry, not only because of the traditional reactivity of their complexes with nucleophiles and unsaturated systems but also because of their role in catalytically driven transformations and the preparation of valuable materials.^1–3^

Unlike noble metals, they form precise and robust complexes and organometallic species, exhibit higher activity in certain transformations, and are abundant, inexpensive and generally less toxic.^2^ The majority of these features and abilities are connected to the fact that B and Al compounds are among the strongest neutral and cationic Lewis acids.^1,2^ These catalysts have historically been associated with the hydrosilylation of carbonyls and alkenes, hydroboration, dehydrogenative coupling, carbocation-type rearrangements, cycloadditions, Diels–Alder reactions, the borylation of C–H, C–C or C–heteroatom bonds, and various polymerization reactions.^2–5^ For aluminum, the portfolio is even wider and includes heterogeneous processes such as petrochemical reactions on alumina, zeolites, or amorphous aluminum compounds. Other homogeneous reactions include Friedel–Crafts alkylation and acylation, aldol and Mannich reactions, Diels–Alder cycloadditions, epoxide ring opening, and carbonyl activation for nucleophilic addition and polymerization.^1–3^

Landmark works by Piers on B­(C_6_F_5_)_3_ and similar compounds, borocations and Frustrated Lewis Pairs by Stephan, as well as the reactivity of low-valent aluminum species by Roesky, have brought novel approaches and targets.^6–13^

In particular, the β-diketiminate system, used by Roesky^9^ (Figure 1), is one of the most prominent ligands responsible for stabilizing the first mononuclear Al­(I) complex in a six-membered chelate. Other nitrogen-rich ligands that form a robust chelate around the central Group 13 element include amidinates and guanidinates, where the chelate rings are usually contracted to the four-membered (Figure 1).^14–25^ The virtual connection of several types of these ligands leads, among others, to biguanides—fused guanidines, which can act as doubly deprotonated compounds capable of forming four- and six-membered rings around the central atom.^15,16,21–28^ Boron-biguanide (sometimes in the literature called also bis-guanidinates) complexes were prepared by Milks^23^ from the parent biguanide and by Lappert^29^ using an addition of amidoboranes to carbodiimides in 1960s. Lappert’s synthetic approach was followed by Dorokhov in 1970s^30–32^ and the same compounds were used for combined theoretical/PES studies in 2016.^33^ In aluminum chemistry, the fusing of two carbodiimides during the attempted addition of organoaluminum species, yielding dinuclear complexes containing a biguanide ligand, was reported by us.^34^ The aluminum guanidinate to biguanide ring expansion by carbodiimide was found by Wei.^35^ Main group complexes containing ethylene-bridged biguanide prepared by the addition of an amine to bis-carbodiimide were studied by Kretchmer later on.^36^ During the last five years, Nembenna tried to find some photochemical applications for symmetrical boron-biguanide BODIPY-like species or aluminum complexes as catalysts in hydroboration reactions.^14,21,22,25^ Noteworthy, the tetra-substituted CBGs employed by Nembenna afforded, aside from several boron or aluminum monometallic complexes (general scheme in Figure 1) also a homobimetallic bis­(aluminum) species in one case (Figure 1).^21^

**Figure 1:**
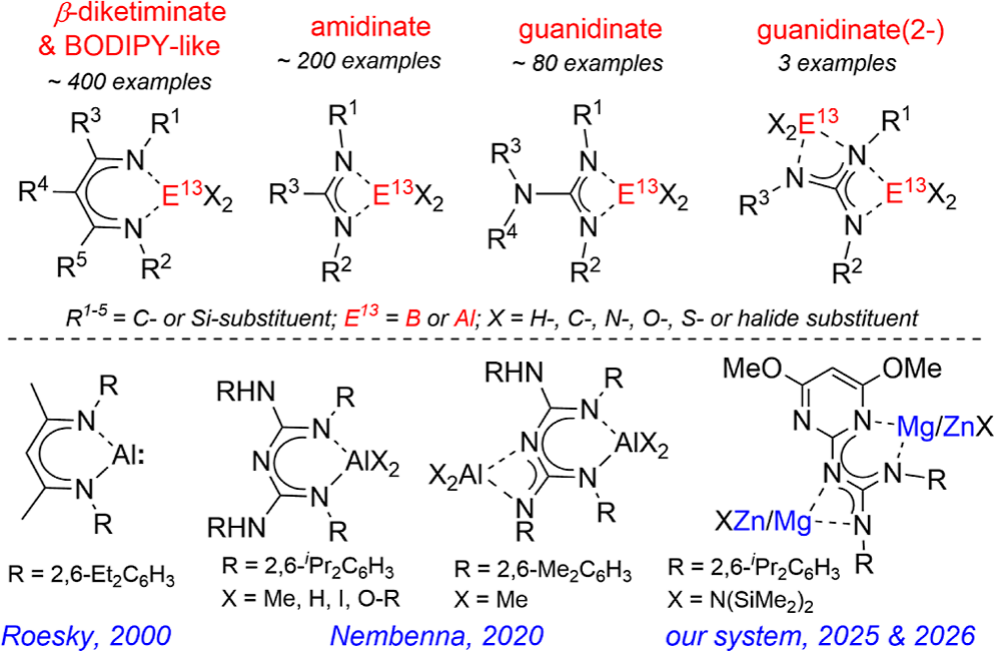
Overview of Group 13 complexes bearing *N*,*N′*-chelating ligands and biguanide Zn/Mg complexes.^2,4,5,9,14–25^

Our own system, which contains an electron-rich 4,6-(dimethoxy)-2-aminopyrimidyl moiety as well as a sterically hindered guanidine part, can act as both a mono- and a dianionic ligand for zinc and magnesium.^15,16^ These complexes have been found to be some of the most effective co-initiators for the ring-opening polymerization of esters, lactones, and carbonates. The motivation behind this work was to extend the successful use of this ligand to lighter members of group 13 and explore coordination chemistry and possible applications.

## Results and Discussion

### Synthesis and Characterization of Mono- and Bimetallic Compounds

Thanks to the nonsymmetric structure of **LH**_**2**_ with two discrete N–H groups of different acidity, these can be deprotonated sequentially and thus the ligand can act as both **LH** ^**–**^ and **L** ^**2–**^. The first deprotonation (Scheme 1) of **LH**_**2**_ was carried out by 1 equiv of ^*n*^BuLi in diethyl ether to yield **LH­(Li)** ^*4*^ (number 4 in superscript means coordination in a four-membered chelate) essentially quantitatively. The ^1^H NMR spectrum recorded in THF-*d*_8_ shows the presence of one significantly deshielded NH group (δ = 9.16 ppm), which is suggested to be involved in resonance-assisted H-bond. Based on the similarity to the chemical shift (9.66 ppm in THF-*d*_8_) of the NH group in **LH**_**2**_, one can suggest that the intramolecular NH···N bridge, creating thus a 6-membered ring, is retained and the lithium ion is coordinated elsewhere.

**Scheme 1:**
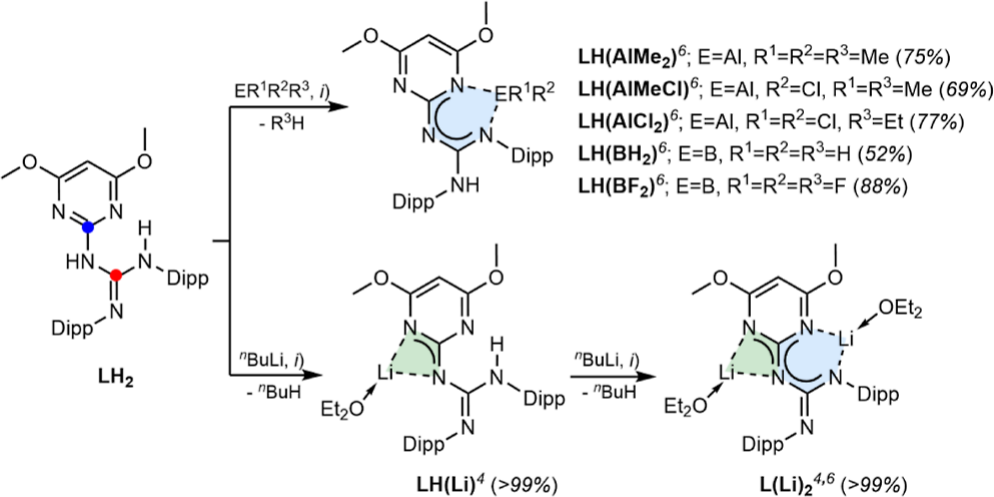
Synthesis of Mononuclear Aluminum, Boron, and Lithium Biguanides and Dilithium Biguanide^a^

a (i) Et_2_O, −80 °C to RT. In **LH**_**2**_, carbon atoms ArC_q_^Prm^ and ArC_q_^Gua^ are marked with blue and red dots, respectively. Isolated yields are given in parentheses

From the comparison of indicative signals (for ArC_q_^Prm^ and ArC_q_^Gua^) in ^13^C NMR spectra of **LH­(Li)** ^*4*^ and **LH**_**2**_, one can conclude the structure of **LH­(Li)** ^*4*^ comprises of the lithium ion coordinated by two nitrogen atoms (pyrimidine N1 and central biguanide N3 nitrogen) in a four-membered chelate ring, as drawn in Scheme 1. Additionally, the lithium is extra-coordinated by the solvent molecule(s)—diethyl ether in the reaction media or THF-*d*_8_ in the NMR tube and resonates at 0.2 ppm (THF-*d*_8_) in the ^7^Li NMR spectrum. The nature of the solvate precludes its crystallization from polar solvents and low solubility in nonpolar solvents prevents the formation of suitable crystalline materials. After recrystallization from ^*n*^hexane, the *sc*-XRD analysis of **LH­(Li)** ^*4*^ was possible and showed a dimeric species (Figure 2) with both lithium atoms chelated and doubly bridged by two biguanide ligands in a κ^1^-*N*-κ^2^-*N*,*N*′-type (ladder-type)^37^ fashion. The N–Li–N “bite angles” range between 64–66°, and the metals are coordinated isobidentately with Li–N distances of 2.02–2.23 Å. The Li···Li separations (∼2.81–2.84 Å) suggest contacted Li pairs. To achieve the dimeric structure, the biguanide backbone was misshaped considerably *via* a rotation along the central N1–C1 (N6–C32) bond about *ca* 80°. This geometry of the ligand is not suitable for the intramolecular NH···N bonding, present in **LH**_**2**_ and probably in **LH­(Li)** ^*4*^ THF-*d*_8_ solutions. Despite that, still prominent strong n-π conjugation reflects in the 1.33–1.38 Å range of values for the C–N distances in the biguanide skeleton, with the exception of the shorter (1.29–1.33 Å) C1–N2 (C32–N7) imine-like bond.

**Figure 2:**
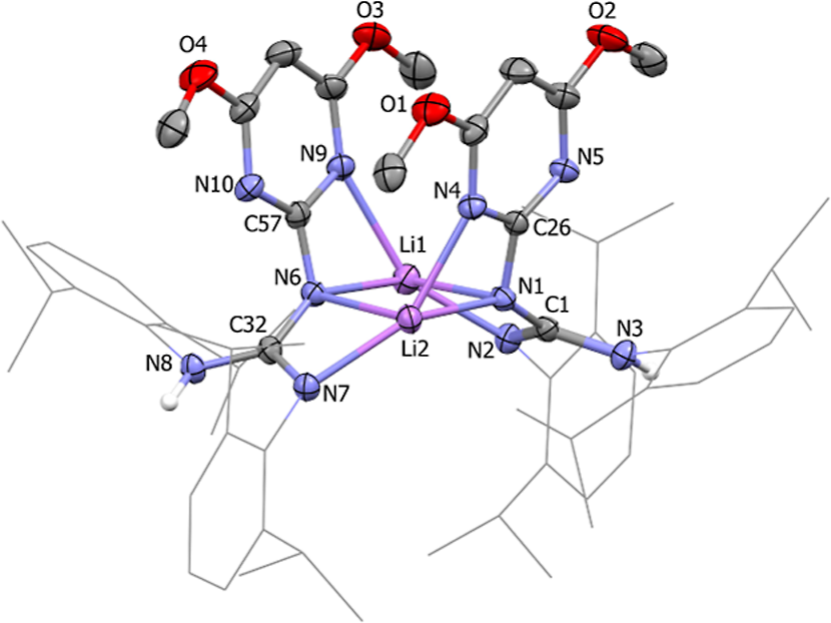
Side view (ORTEP 35% probability) of the molecular structure of dimeric **LH­(Li)** ^*4*^. Hydrogen atoms, except for NH groups, are omitted for clarity. A second independent molecule and the full list of interatomic distances and bond angles are listed in Supporting Information, p. S8.

In order to evaluate whether sequential reactivity of **LH**_**2**_ works similarly for other reagents, commercially available Me_3_Al, Me_2_AlCl, and EtAlCl_2_ and boron species BH_3_·Me_2_S and BF_3_·Et_2_O were tested, giving appropriate **LH­(AlMe**_**2**_ **)** ^*6*^, **LH­(AlMeCl)** ^*6*^, **LH­(AlCl**_**2**_ **)** ^*6*^, **LH­(BH**_**2**_ **)** ^*6*^, and **LH­(BF**_**2**_ **)** ^*6*^ in good yields (Scheme 1—number 6 in superscript means coordination in a six-membered chelate). The boron complexes turned out to be air-stable and thus additional recrystallization (for **LH­(BH**_**2**_ **)** ^*6*^) and liquid–liquid extraction (toluene/H_2_O for **LH­(BF**_**2**_ **)** ^*6*^) were performed to purify the compounds. In the cases of **LH­(AlMe**_**2**_ **)** ^*6*^, **LH­(AlCl**_**2**_ **)** ^*6*^, and **LH­(BF**_**2**_ **)** ^*6*^, an alternative synthetic approach employed **LH­(Li)** ^*4*^ as the starting material, which was treated with aluminum- or boron halide (Me_2_AlCl, AlCl_3_, or BF_3_·Et_2_O) upon lithium halide elimination (Scheme S1 in the Supporting Information). The obvious target molecules are the iodinated aluminum species, which can be reduced to low-valent compounds more easily than other halides.^9^ Treatment of **LH­(AlMe**_**2**_ **)** ^*6*^ with one equiv of I_2_ at 60 °C yielded **LH­(AlMeI)** ^*6*^ after 2 h while the second exchange to **LH­(AlI**_**2**_ **)** ^*6*^ with two equiv of I_2_ was finished after 72 h (Scheme S2 in the Supporting Information).

The NMR analysis of monoanionic aluminum or boron biguanides in C_6_D_6_ (or THF-*d*_8_ for **LH­(AlCl**_**2**_ **)** ^*6*^) revealed the presence of only one NH resonance, in agreement to a deprotonation to the first step, at ∼5–6 ppm (opposed to 9.16 ppm for **LH­(Li)** ^*4*^ in THF-*d*_8_). This value, the spectral pattern, and the similar chemical shift of both Ar*C*_q_^Gua^ and Ar*C*_q_^Prm^ in ^13^C NMR spectra (160.1–161.7 ppm for **LH­(AlX**_**2**_ **)** or 158.2–160.6 ppm for **LH­(BX**_**2**_ **)** analogues) correspond to the previously reported^15,16^ arrangement with a 6-membered chelate. The electron-donating and electron-withdrawing characters of the methyl and halide substituents at the Al center is reflected, respectively, in the ^1^H chemical shift of N*H* ^Dipp^ and the ^13^C chemical shift of Ar*C*H^Prm^ (Figure 3). Due to dynamic behavior in solution, the signal in ^11^B NMR spectra for **LH­(BH**_**2**_ **)** ^*6*^ is very broad at −8.50 ppm (C_6_D_6_) and no H-coupling can be observed. The electron-withdrawing fluorine substituents in **LH­(BF**_**2**_ **)** ^*6*^ cause deshielding of the boron nucleus (1.15 ppm in C_6_D_6_, 0.35 ppm in THF-*d*_8_) and splitting to a dd, appearing as a triplet with ^1^ *J* = 26.5 Hz. The corresponding ^19^F resonance is found as a nonbinomial quartet at −132.2 (C_6_D_6_) or −133.5 ppm (THF-*d*_8_). Additionally, a very weak C–F splitting constant was found in ^13^C NMR spectra for Ar*C*_q_^OMe^ (^4^ *J*_C,F_ = 2.6 Hz) and *C*H_3_^Dipp^ (^*6*^ *J*_C,F_ = 4.0 Hz) due to the through-space interaction of the fluorine atom with C–H moieties attached to the affected carbons.

**Figure 3:**
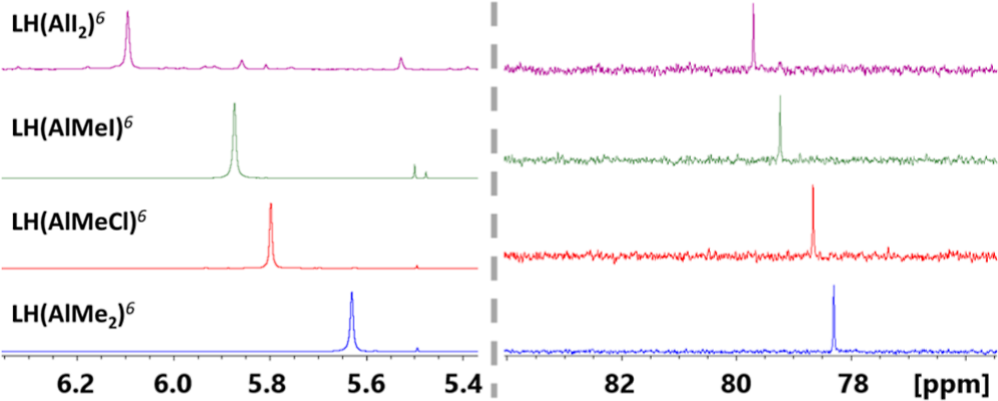
Detail of the N*H* ^Dipp^ signal in ^1^H (left) and Ar*C*H^Prm^ in ^13^C (right) NMR spectra of monometallic isostructural aluminum biguanides (C_6_D_6_, 295 K).

In the solid state of the isostructural monoanionic **LH­(AlX**_**2**_ **)** ^*6*^ and **LH­(BX**_**2**_ **)** ^*6*^ (X = Me, H, F, Cl, or I—see examples in Figure 4), the position of the NH group changed (relative to **LH­(Li)** ^*4*^). The neighboring pyrimidine ring and metallacycle are planar, with the aluminum atom slightly misplaced from the plane by *ca* 0.2–0.8 Å, whereas the boron atom lies in the biguanide plane due to the smaller ionic radius. The aluminum/boron atom is coordinated in a deformed tetrahedral geometry almost isobidentately to the biguanide ligand (the difference of N–Al lengths is *ca* 0.05 Å and even less for N–B), and the N–Al distances 1.85–1.96 Å slightly elongate with less electronegative Al-atom substituents in a series **LH­(AlCl**_**2**_ **)** ^*6*^ < **LH­(AlI**_**2**_ **)** ^*6*^ < **LH­(AlMeI)** ^*6*^ ≈ **LH­(AlMeCl)** ^*6*^ < **LH­(AlMe**_**2**_ **)** ^*6*^. The computed APT charges at Al atoms of **LH­(AlX**_**2**_ **)** ^*6*^ species (Figure S35) differ significantly with substitution by negative atoms, which correlates with chemical shift values for these species presented in Figure 3 and Lewis acidity of the Al atom. More negative charges (∼0.1 e) were obtained for Al atoms chelated by a four membered system. The same trend is observable for the N–B lengths, which are shorter for **LH­(BF**_**2**_ **)** ^*6*^ (1.54–1.58 Å) than for **LH­(BH**_**2**_ **)** ^*6*^ (1.57–1.58 Å). The interatomic C–N distance values in the range of 1.33–1.38 Å lie between the standard single and double bond lengths (1.46 Å and 1.27 Å)^38^ including the exocyclic C–NH bond (e.q. C1–N2 in **LH­(BH**_**2**_ **)** ^*6*^ in Figure 4). A strong parallel offset π−π stacking^39^ of 3.3–3.4 Å is observed for the pyrimidine rings in all **LH­(Al)** ^*6*^ species in the solid state (see Figure S1 in the Supporting Information), whereas the related distance between parallel pyrimidine units in boron analogue **LH­(BH**_**2**_ **)** ^*6*^ is around 7.5 Å. **LH­(BH**_**2**_ **)** ^*6*^ packs differently and there is a H-bond between the exocyclic NH^Dipp^ and the oxygen atom from a tetrahydrofuran molecule. A weak halogen interaction in **LH­(AlI**_**2**_ **)** ^*6*^ (3.877 Å at 133.24°) is also present.

**Figure 4:**
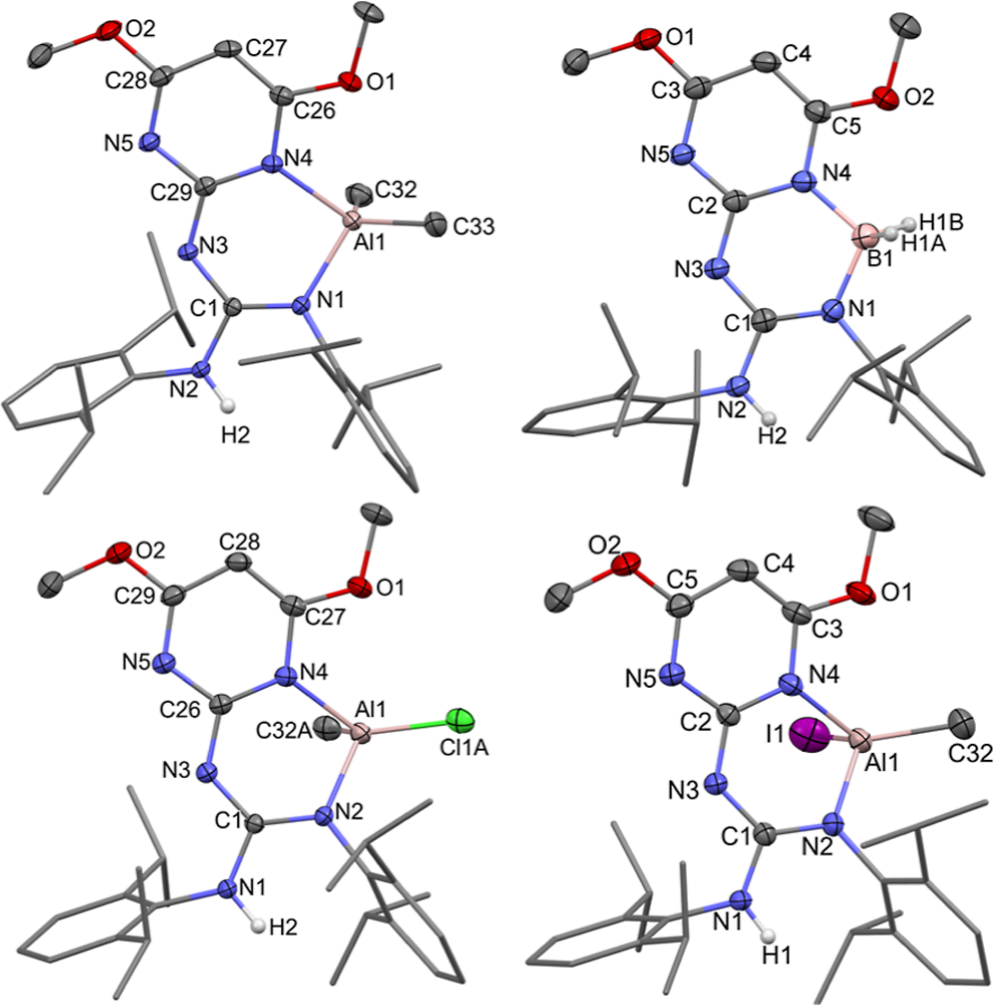
Molecular structures of **LH­(AlMe**_**2**_ **)** ^*6*^, **LH­(BH**_**2**_ **)** ^*6*^, **LH­(AlMeCl)** ^*6*^, and **LH­(AlMeI)** ^*6*^. ORTEP view with 50% probability and capped sticks representation of the Dipp substituents. Hydrogen atoms, except for NH group, are omitted for clarity. Full list of interatomic distances and bond angles is listed in Supporting Information, pp. S9–S11.

To prove the idea of sequential deprotonation of ligands and possible formation of biguanides(2−), the dinuclear compounds were attempted and successfully prepared *via* treatment of **LH**_**2**_ with 2 equiv of the same reagents as the mononuclear ones. Using ^*n*^BuLi, **L­(Li)**_**2**_^*4*,*6*^ was obtained in the form of a waxy solid in quantitative yield. NMR analysis in THF-*d*_8_ showed two distinct resonances in ^7^Li NMR spectra at 1.4 and 0.3 ppm, the latter being similar to 0.2 ppm in **LH­(Li)** ^*4*^. Thus, a nonsymmetric arrangement can be expected with one Li atom coordinated in a 4-membered chelate like in **LH­(Li)** ^*4*^, further supported by the ^1^H NMR spectral pattern. This leads us to propose a structure with a six-membered and a four-membered chelate, both joined to the pyrimidine ring (Scheme 1).

When employing organoaluminum reagents, the double deprotonation reaction of **LH**_**2**_ by 2 equiv of Me_3_Al or Me_2_AlCl in Et_2_O caused the formation of well soluble **L­(AlMe**_**2**_ **)**_**2**_^*6*,*4*^ and **L­(AlMeCl)**_**2**_^*6*,*4*^. Both species exhibit high similarity in the NMR spectral patterns. In ^13^C NMR spectra, the ArC_q_^Gua^ (δ = 164.7/166.0 ppm) is significantly downfield shifted compared to ArC_q_^Prm^ (δ = 158.2/157.2 ppm) in C_6_D_6_ for **L­(AlMe**_**2**_ **)**_**2**_^*6*,*4*^ and **L­(AlMeCl)**_**2**_^*6*,*4*^, respectively. This situation is quite different to the dilithium system with two chelates in **L­(Li)**_**2**_^*4*,*6*^, where the signal position is reversed, because ArC_q_^Prm^ is a part of both metallacycles and is therefore less shielded than ArC_q_^Gua^. Hence, the guanidinate carbon atom in **L­(AlMe**_**2**_ **)**_**2**_^*4*,*6*^ and **L­(AlMeCl)**_**2**_^*4*,*6*^ is expected to be a part of both chelate rings. This observation brings the suggestion of the “6,4” structural pattern, as drawn in Scheme 2, further confirmed by *sc-*XRD analysis (Figure 5). The “6,4” structural pattern in the solid state for **L­(AlMe**_**2**_ **)**_**2**_^*6*,*4*^ is analogous to the previously described bis­(zinc-amido)-biguanide complex,^15^ with the two chelate rings sharing the strongly deshielded (δ = 164.7 ppm, C_6_D_6_) guanidinate carbon C1 (Figure 5). The C–N interatomic distances in the biguanide moiety are related to the strong n-π conjugation, with values between 1.33 and 1.39 Å. The greatest is the distance C1–N3 between the two atoms shared by both chelate rings. The repulsion between both Dipp substituents, forced into coplanarity by the position of the Al2 atom, causes the displacement of the N1 atom from the otherwise planar biguanide skeleton.

**Scheme 2:**
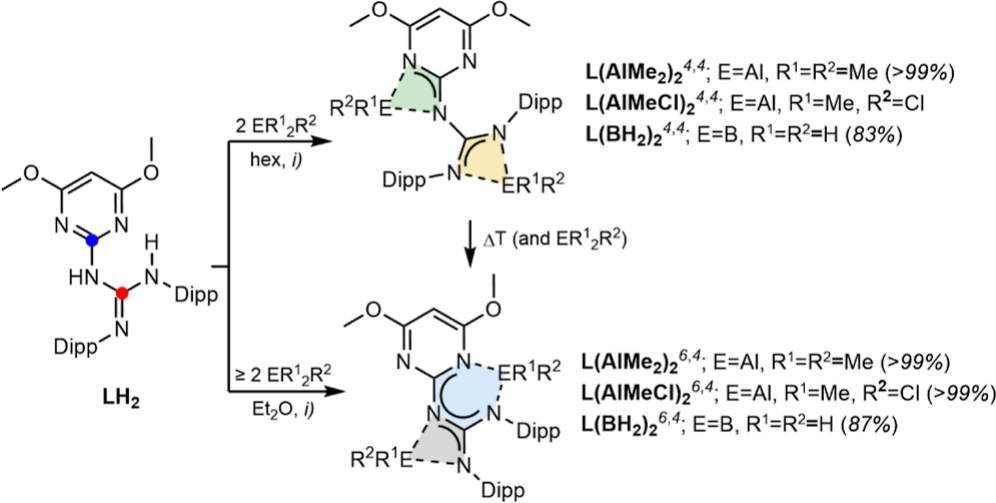
Synthesis of Dinuclear “4,4 and 6,4” Aluminum and Boron Biguanides^a^

a (i) −80 °C or RT, -R^1^H. In **LH**_**2**_, carbon atoms ArC_q_^Prm^ and ArC_q_^Gua^ are marked with blue and red dots, respectively. Isolated yields are given in parentheses

**Figure 5:**
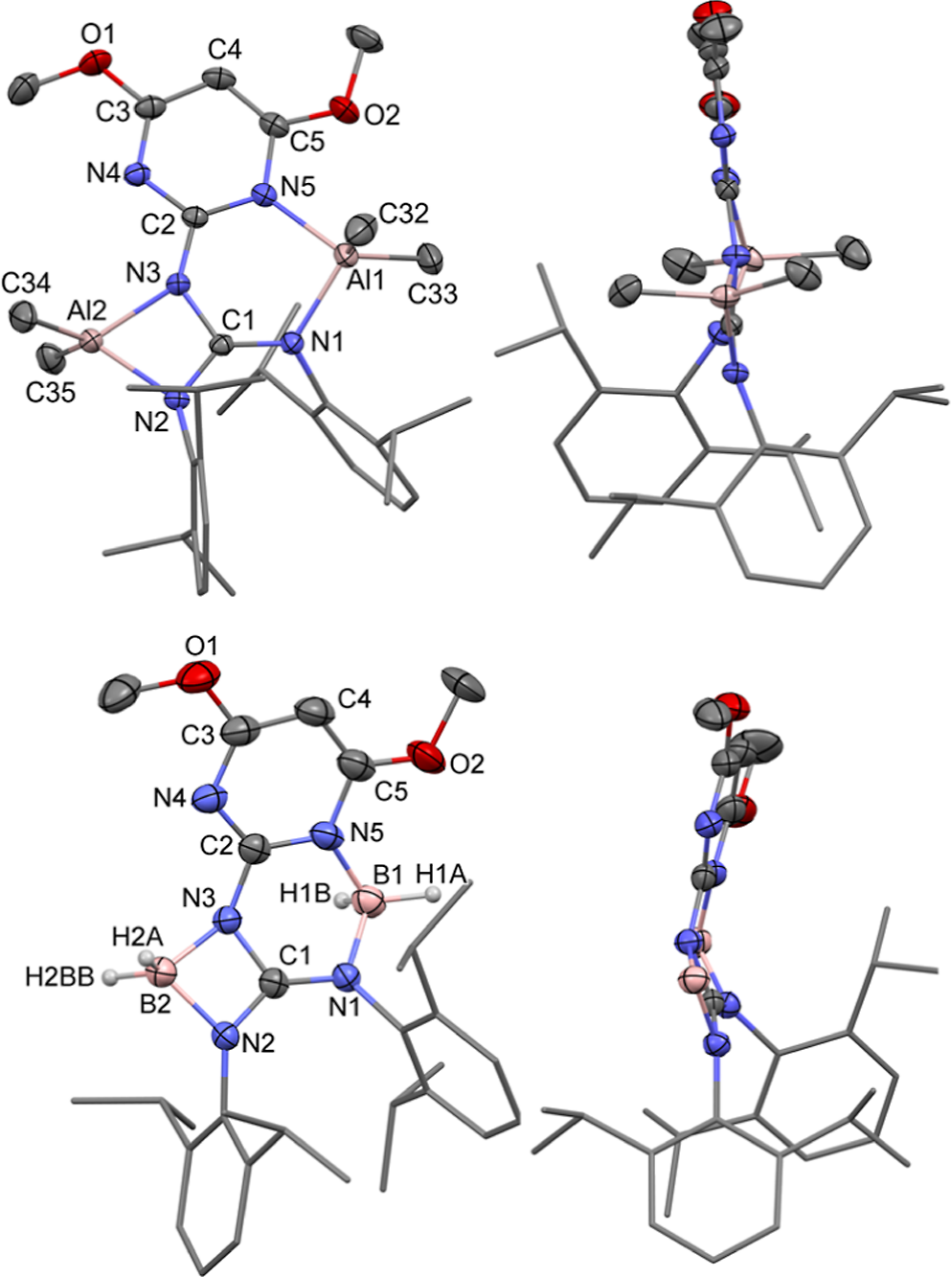
Molecular structure of **L­(AlMe**_**2**_ **)**_**2**_^*6*,*4*^ (upper—left), **L­(BH**_**2**_ **)**_**2**_^*6*,*4*^ (lower—left) and side views, showcasing the planar and concave nature of the joined pyrimidine ring and heterocycles (right). ORTEP view with 50% probability and capped sticks representation of the Dipp substituents. Hydrogen atoms are omitted for clarity. The full list of interatomic distances and bond angles is listed in Supporting Information, pp. S13–S14.

Treatment of **LH**_**2**_ with (even a slight) excess than 2 equiv of BH_3_·Me_2_S yields **L­(BH**_**2**_ **)**_**2**_^*6*,*4*^ (Scheme 2). The patterns of ^1^H and ^13^C NMR spectra of the bis­(boron) species are comparable to bis­(aluminum), in addition very broad BH_2_ group signals were observed at 4.02/3.48 ppm in C_6_D_6_. Surprisingly, close chemical shifts at −8.9/–11.5 ppm for **L­(BH**_**2**_ **)**_**2**_^*6*,*4*^ were observed in the ^11^B NMR spectrum. Unlike the almost planar geometry of the pyrimidine and chelate rings in the Al species, the biguanide moiety in **L­(BH**_**2**_ **)**_**2**_^*6*,*4*^ is bent (Figure 5, right lower part), most probably due to the smaller atomic radius of boron. The angle between N_3_C planes around C1 and C2 is 28.67°.

Next, the introduction of one equivalent of I_2_ to the Et_2_O solution of **L­(AlMe**_**2**_ **)**_**2**_^*6*,*4*^ led to the selective formation of **L­(AlMe**_**2**_ **)** ^*6*^ **(AlMeI)** ^*4*^ in quantitative yields (Scheme 3). The position of the iodine atom was determined on the basis of ^1^H 1D-selective gradient NOESY NMR experiments, as well as the *sc*-XRD analysis, as a substituent of the Al2 atom, chelated in a 4-membered metallacycle (Figure 6). The structural parameters differ only negligibly from the respective parameters for **L­(AlMe**_**2**_ **)**_**2**_^*6*,*4*^. When the monometallic **LH­(AlMeI)** ^*6*^ was deprotonated with Me_3_Al, **L­(AlMeI)** ^*6*^ **(AlMe**_**2**_ **)** ^*4*^ was isolated in an 87% yield after washing with hexane (Scheme 3). The structure was once again corroborated by ^1^H-NOESY NMR experiments. Thus, two bimetallic aluminum species were synthesized by the modification of the reaction procedure (order of reagents) with a controlled position of the halide substitution.

**Scheme 3:**
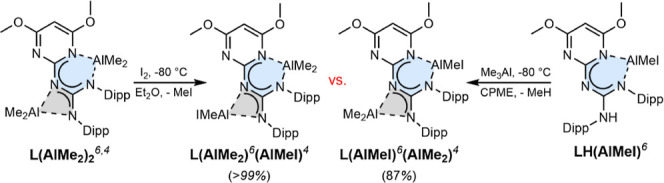
Synthetic routes to Dinuclear “6,4” Complexes **L­(AlMe**_**2**_ **)** ^*6*^ **(AlMeI)** ^*4*^ and **L­(AlMeI)** ^*6*^ **(AlMe**_**2**_ **)** ^*4*^ ^a^

a Isolated yields are given in parentheses

**Figure 6:**
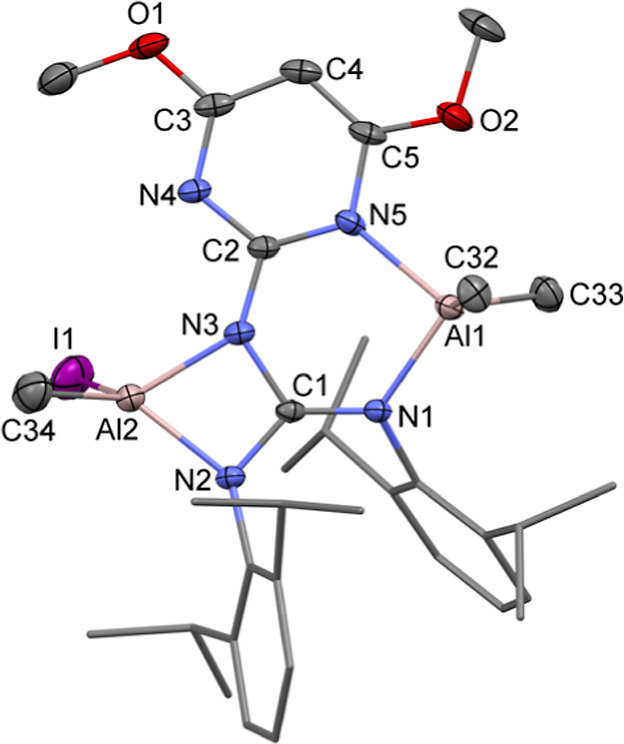
Molecular structure of **L­(AlMe**_**2**_ **)** ^*6*^ **(AlMeI)**.^*4*^ ORTEP view with 50% probability and capped sticks representation of the Dipp substituents. Hydrogen atoms are omitted for clarity. The full list of interatomic distances and bond angles is listed in the Supporting Information, p. S14 and S15.

Although the conversions of the starting material to **L­(AlMe**_**2**_ **)**_**2**_^*6*,*4*^ are essentially quantitative, the preparation of larger quantities of material needed for reactivity studies was negatively influenced by the challenging crystallization of the very well soluble **L­(AlMe**_**2**_ **)**_**2**_^*6*,*4*^. Thus, the modified procedure of **LH**_**2**_ deprotonation by 2 equiv of Me_3_Al carried out in hexane was attempted. Surprisingly enough, the material obtained was pure but exhibited totally different NMR spectral patterns than **L­(AlMe**_**2**_ **)**_**2**_^*6*,*4*^. The crystalline product, however, was identified as **L­(AlMe**_**2**_ **)**_**2**_^*4*,*4*^ by NMR spectroscopy as well as the *sc-*XRD technique. Subsequently, the same phenomenon was identified for the deprotonation of **LH**_**2**_ by 2 equiv of Me_2_AlCl, which yielded **L­(AlMeCl)**_**2**_^*4*,*4*^. The ^1^H and ^13^C NMR spectra of the two species are similar and indicate a higher degree of molecular symmetry, as evidenced by the chemical equivalence of the Dipp substituents, Al–methyl and methoxy groups (see the Supporting Information). In the ^13^C NMR spectra recorded in C_6_D_6_, the ArC_q_^Prm^ and ArC_q_^Gua^ resonances are found at closely comparable chemical shifts (δ = 161.4/160.2 ppm for **L­(AlMe**_**2**_ **)**_**2**_^*4*,*4*^ and δ = 161.6/161.2 ppm for **L­(AlMeCl)**_**2**_^*4*,*4*^, respectively). In the solid state, both species are isostructural, with the aluminum atoms coordinated in separated 4-membered chelates (Figure 7), each including one biguanide carbon atom, which is in correspondence to the similar ^13^C chemical shift of ArC_q_^Gua/Prm^. The chelate planes form an angle of approximately 34°. This decrease of planarity of the biguanide moiety is mainly caused by the displacement of the C1 atom through the change of the N5–C2–N3–C1 torsion angle and a rotation of the N3–C1 bond. These changes in the geometry, however, have no effect on the high degree of electron density delocalization, as all the C–N distances range between 1.32 and 1.38 Å. Also, both methoxy groups are facing the same direction (contrary to the solid-state structures of [**L­(Li)**_**2**_^*4*^, **LH­(AlX**_**2**_ **)** ^*6*^, **LH­(BX**_**2**_ **)** ^*6*^, and **L­(AlMe**_**2**_ **)**_**2**_^*6*,*4*^], probably due to crystal packing. When comparing the interatomic Al–N distances (1.89–2.00 Å) and bite angles (67.3–70.3°) of the four-membered chelates in **L­(AlMe**_**2**_ **)**_**2**_^*4*,*4*^, **L­(AlMeCl)**_**2**_^*4*,*4*^, and **L­(AlMe**_**2**_ **)**_**2**_^*6*,*4*^, only negligible difference can be observed (see full data in the Supporting Information).

**Figure 7:**
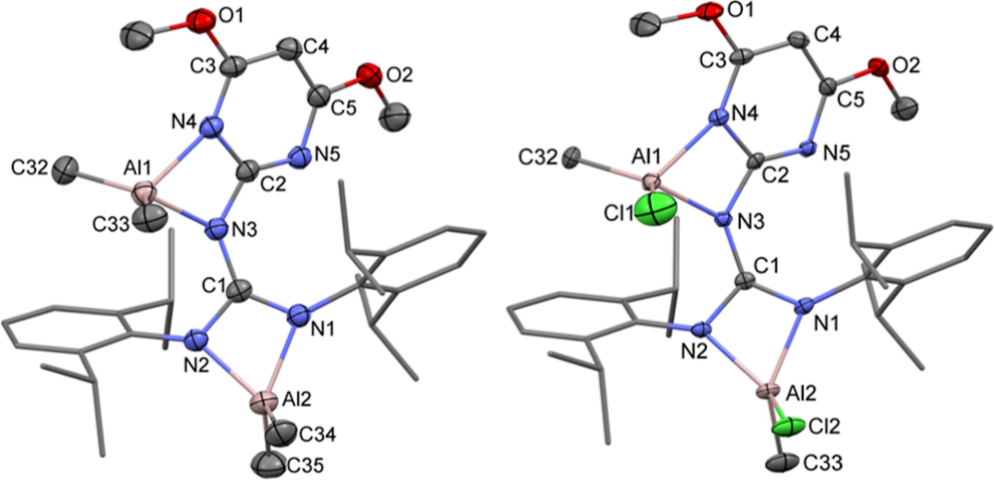
Molecular structure of **L­(AlMe**_**2**_ **)**_**2**_^*4*,*4*^ (left) and **L­(AlMeCl)**_**2**_^*4*,*4*^ (right). ORTEP view with 50% probability and capped sticks representation of the Dipp substituents. Hydrogen atoms are omitted for clarity. The full list of interatomic distances and bond angles is listed in the Supporting Information, p. S12 and S13.

The second coordination isomer can be prepared also in the reaction of **LH**_**2**_ with 2 equiv of BH_3_·Me_2_S, which yields **L­(BH**_**2**_ **)**_**2**_^*4*,*4*^, however, this process is solvent-independent (Scheme 2). To achieve **L­(BH**_**2**_ **)**_**2**_^*4*,*4*^, a slightly substoichiometric amount of borane complex (less than 2 equiv) needs to be used, while an excess promotes the formation of **L­(BH**_**2**_ **)**_**2**_^*6*,*4*^ (Scheme 2). The ^1^H and ^13^C NMR spectra of the bis­(boron) isomers showed higher molecular symmetry of **L­(BH**_**2**_ **)**_**2**_^*4*,*4*^, compared to **L­(BH**_**2**_ **)**_**2**_^*6*,*4*^, and very broad BH_2_ group signals at 4.51/3.79 ppm in C_6_D_6_. Also, the well-separated broad peaks in ^11^B NMR spectra of **L­(BH**_**2**_ **)**_**2**_^*4*,*4*^ at 1.8/–7.1 ppm (C_6_D_6_) are observed contrasting to the close chemical shifts at −8.9/–11.5 ppm for **L­(BH**_**2**_ **)**_**2**_^*6*,*4*^. The IR and Raman spectra of these complexes exhibited (Figures S46–S52) specific signals in expected range ∼2,300–2,700 cm^–1^ attributable to vibrations of B–H bonds, but not much differences in patterns for each individual isomer.

The relationship between the “6,4” and “4,4” coordination isomers was studied in detail for **L­(AlMe**_**2**_ **)**_**2**_^*6*,*4*^ and **L­(AlMe**_**2**_ **)**_**2**_^*4*,*4*^ with additional catalytic, stoichiometric, and computational experiments. The stoichiometric reaction of the monoanionic **LH­(AlMe**_**2**_ **)** ^*6*^ with Me_3_Al led to the exclusive formation of the “6,4” form, **L­(AlMe**_**2**_ **)**_**2**_^*6*,*4*^ in polar (Et_2_O, THF) or nonpolar (^*n*^hexane, ^*n*^pentane) solvents alike. The “4,4” isomer is therefore thought to arrive *via* another monoanionic intermediate, possibly with the metal coordinated in a 4-membered chelate. Attempts to prepare such a species, where **LH**_**2**_ was mixed with one equiv of Me_3_Al in ^*n*^hexane or ^*n*^pentane, resulted in the 1:1 mixture of **LH**_**2**_ with the dianionic **L­(AlMe**_**2**_ **)**_**2**_^*4*,*4*^.

According to DFT computations (B3LYP/6-311 + G­(d,p)), the Δ*G* energy of the optimized structures of **L­(AlMe**_**2**_ **)**_**2**_^*4*,*4*^ and **L­(AlMe**_**2**_ **)**_**2**_^*6*,*4*^ is 10 kcal·mol^–1^ in favor of the “6,4” species. Therefore, the thermally conducted transformation of **L­(AlMe**_**2**_ **)**_**2**_^*4*,*4*^ into **L­(AlMe**_**2**_ **)**_**2**_^*6*,*4*^ was investigated in benzene and toluene at various temperatures. At 130 °C in toluene, the decomposition of the complex to carbodiimide can be observed, while at 110 °C, the isomerization takes place during 160 h with no side products (see Figure 8). The same process proceeds at a lower temperature (80 °C) in C_6_D_6_ over the period of 146 days (Figure S5 in the Supporting Information). A kinetically conducted experiment involved the introduction of a 0.5 equiv of Me_3_Al to the Tol-d_8_ solution of **L­(AlMe**_**2**_ **)**_**2**_^*4*,*4*^, which was heated to 80 °C simultaneously against a reference sample without added Me_3_Al and no conversion (Figure 9), where Me_3_Al accelerated the isomerization process to *ca*. 50% conversion to **L­(AlMe**_**2**_ **)**_**2**_^*6*,*4*^ after 20 h.

**Figure 8:**
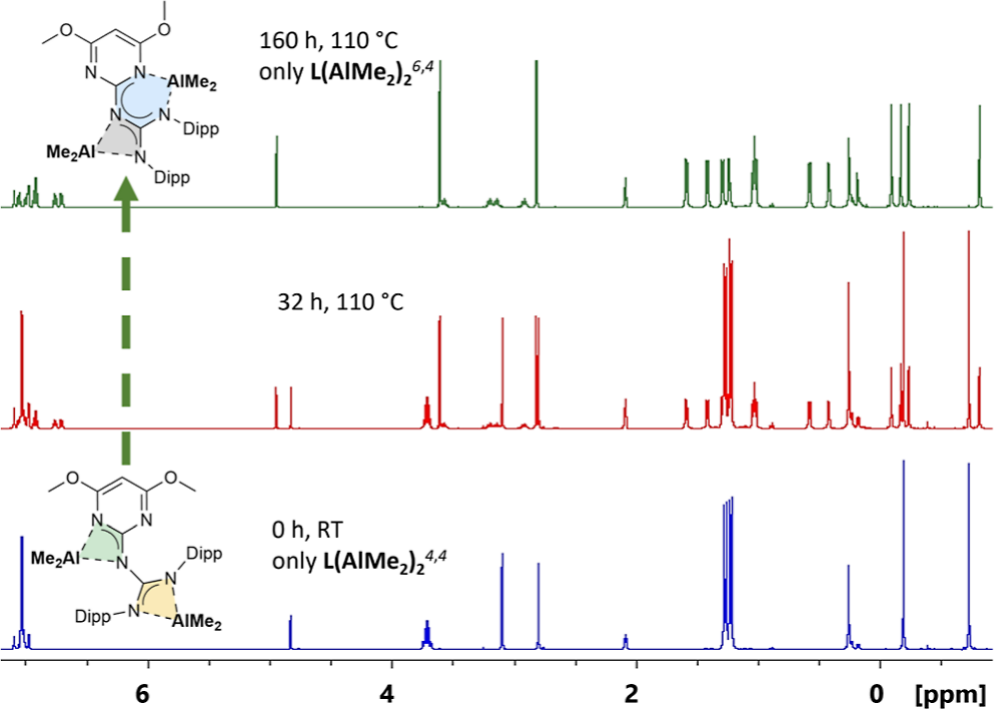
Progress of a thermally induced isomerization of **L­(AlMe**_**2**_ **)**_**2**_^*4*,*4*^ to **L­(AlMe**_**2**_ **)**_**2**_^*6*,*4*^ in Tol-*d*_8_ solution monitored by ^1^H NMR spectroscopy in a sealed NMR tube.

**Figure 9:**
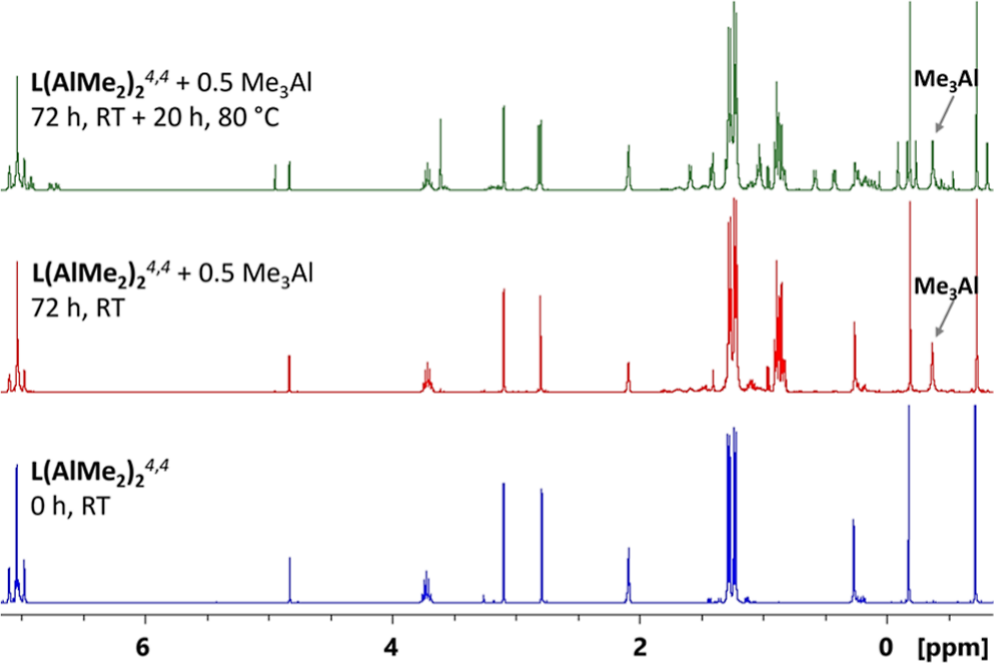
Progress of a thermally induced isomerization of **L­(AlMe**_**2**_ **)**_**2**_^*4*,*4*^ to **L­(AlMe**_**2**_ **)**_**2**_^*6*,*4*^ accelerated by the addition of 0.5 equiv of Me_3_Al. Monitored in Tol-*d*_8_ solution by ^1^H NMR spectroscopy in a sealed NMR tube.

Noteworthy, **L­(BH**_**2**_ **)**_**2**_^*4*,*4*^ could also be isomerized to **L­(BH**_**2**_ **)**_**2**_^*6*,*4*^ with a stoichiometric amount of BH_3_·Me_2_S in 1 h at 80 °C.

The relative Gibbs free energies for optimized structures of experimentally obtained as well as hypothetical molecules of **LH­(AlX**_**2**_ **)**, **L­(AlX**_**2**_ **)**_**2**_, and **L­(BX**_**2**_ **)**_**2**_ (Figures S36–S41) exhibit higher thermodynamic stability for all “6,4” species by 5–20 kcal/mol except the **L­(AlMeCl)**_**2**_ system, where both isomers have almost the same energies.

The aluminum or boron halide biguanides **LH­(AlMeCl)** ^*6*^, **LH­(BF**_**2**_ **)** ^*6*^, **L­(AlMeCl)**_**2**_^*6*,*4*^, and **L­(AlMeI)** ^*6*^ **(AlMe**_**2**_ **)** ^*4*^ were tested for halide abstraction (metal reduction) with potassium metal but only resulted in mixtures of several products (Scheme S3 in the Supporting Information). Attempts of **L­(AlMe**_**2**_ **)**_**2**_^*6*,*4*^ additions to unsaturated C–N and C–O bonds were unsuccessful, even when heated to 80 °C (Scheme S3 in the Supporting Information), the same as for the triple CC bond in acetylene and **L­(AlMe**_**2**_ **)** ^*6*^ **(AlMeI)** ^*4*^ (Scheme S5 in the Supporting Information). Also, the introduction of reducing agents (K, KC_8_ or K­[B­(Et)_3_H]) yielded either no result, or an inseparable mixture of many products.

### ROP Catalysis

Our previous findings showed that magnesium and zinc complexes coordinated by a **L-** or **LH-**biguanide system exhibit extraordinary activity toward the ring-opening polymerization of lactide.^15,16^ To the best of our knowledge, there is no report on ROP by biguanide Group 13 complexes. Hence, several prepared mono- or dianionic complexes with the -AlMe_2_ part, namely **LH­(AlMe**_**2**_ **)** ^*6*^, **L­(AlMe**_**2**_ **)**_**2**_^*4*,*4*^, and **L­(AlMe**_**2**_ **)**_**2**_^*6*,*4*^, and both monoanionic biguanide boron species **LH­(BH**_**2**_ **)** ^*6*^ and **LH­(BF**_**2**_ **)** ^*6*^ were investigated as initiators in the *rac*-lactide ROP (Table 1). The aluminum species **LH­(AlMe**_**2**_ **)** ^*6*^, **L­(AlMe**_**2**_ **)**_**2**_^*4*,*4*^, and **L­(AlMe**_**2**_ **)**_**2**_^*6*,*4*^ exhibit a lower catalytic activity than the corresponding Mg and Zn complexes.^15,16^ In contrast, the biguanide Al complexes outperform the structurally related β-diketiminate complexes (TOF of up to 11 h^–1^ at 90 °C vs 0.5 h^–1^ at 80 °C).^40^ They afford comparable performance to that of amidinate aluminum complexes (TOF of 7.8 h^–1^ at 70 °C, without a co-initiator)^41^ or to mononuclear phenoxy–imine aluminum complexes (TOF of up to 6 h^–1^ at 100 °C).^42–44^ The experiment conducted using the known complex Al­(Me)_2_(OC_6_H_4_(2-(C­(H)­N­(*^n^* Pr)))) under the conditions described in Table 1 (entry 16) afforded also comparable activity, albeit with reduced control.^45^ Nevertheless, biguanide Al complexes remain significantly less active than the most efficient aluminum catalysts reported to date, including those described by Carpentier and Kirillov^46^ (TOF = 82 h^–1^ at 90 °C, bis­(phenoxy–imine)­Al_2_), Jones^47^ (TOF = 194 h^–1^ at 80 °C, (catalen)­Al), Chen^48^ (TOF = 75 h^–1^ at 70 °C, (thiophenoxy–imine)­Al), and Li^49^ (TOF = 147 h^–1^ at 80 °C, (bis­(β-ketoiminate))­Al).

**Table 1:** ROP of *rac*-Lactide Mediated by Complexes **LH­(AlMe**_**2**_ **)** ^*6*^, **L­(AlMe**_**2**_ **)**_**2**_^*4*,*4*^, **L­(AlMe**_**2**_ **)**_**2**_^*6*,*4*^, **LH­(BH**_**2**_ **)** ^*6*^, and **LH­(BF**_**2**_ **)** ^*6*^ ^a^

entry	cat.	[mon.]/[cat]_0_/[^*i*^PrOH]	*T* [°C]	solvent	time [h]	conv.^b^ [ %]	*M*_*n*,theo_ ^c^ [g mol^–1^]	*M*_*n*,exp_ ^d^ [g mol^–1^]	*D̵* ^*d*^	TOF [h^–1^]
1	**LH(AlMe**_**2**_ **)** ^*6*^	100:1:1	20	DCM	8	0	-	-	-	-
2	**LH(AlMe**_**2**_ **)** ^*6*^	100:1:1	90	Tol	8	90	13,000	12,200	1.08	11.25
3	**LH(AlMe**_**2**_ **)** ^*6*^	100:1:1	90	Tol	24	95	13,700	12,500	1.24	3.96
4	**L(AlMe**_**2**_ **)**_**2**_^*4*,*4*^	100:1:1	90	Tol	8	71	10,200	5,900	1.11	4.44
5	**L(AlMe**_**2**_ **)**_**2**_^*4*,*4*^	100:1:2	90	Tol	8	89	6,400	4,400	1.09	5.56
6	**L(AlMe**_**2**_ **)**_**2**_^*4*,*4*^	200:1:2	90	Tol	8	83	12,000	5,900	1.10	10.38
7	**L(AlMe**_**2**_ **)**_**2**_^*4*,*4*^	200:1:2	60	Tol	8	10	n.d.	n.d.	n.d.	1.25
8	**L(AlMe**_**2**_ **)**_**2**_^*6*,*4*^	100:1:1	90	Tol	8	71	10,200	7,300	1.09	4.44
9	**L(AlMe**_**2**_ **)**_**2**_^*6*,*4*^	100:1:2	90	Tol	8	93	6,700	4,900	1.09	5.81
10	**L(AlMe**_**2**_ **)**_**2**_^*6*,*4*^	200:1:2	90	Tol	8	78	11,200	7,400	1.13	9.75
11	**L(AlMe**_**2**_ **)**_**2**_^*6*,*4*^	200:1:2	60	Tol	8	9	n.d.	n.d.	n.d.	1.13
12	**LH(BH**_**2**_ **)** ^*6*^	100:1:1	20	DCM	3	0	-	-	-	-
13	**LH(BH**_**2**_ **)** ^*6*^	100:1:1	90	Tol	2	0	-	-	-	-
14	**LH(BF**_**2**_ **)** ^*6*^	100:1:1	20	DCM	16	0	-	-	-	-
15	**LH(BF**_**2**_ **)** ^*6*^	100:1:1	90	Tol	4	0	-	-	-	-
16	**Al(Me)**_**2**_ **(OC**_**6**_ **H**_**4**_ **(2-(C(H)** **N(** *^n^* **Pr))))**	100:1:1	90	Tol	8	96	13,800	8,500	2.2	12

a All experiments were duplicated. Polymerization conditions: [mon]_0_ = 1.0 M. Reactions performed with a batch of recrystallized and sublimated LA.

b Monomer conversion was calculated from ^1^H NMR spectra in CDCl_3_.

c Calculated using *M*_*n*,theo_ = [mon.]_0_/([^*i*^PrOH]_0_) × M_mon._ × conversion.

d Measured by GPC in THF (45 °C) using PS standards and corrected by applying the appropriate correcting factor (0.58).

The monoanionic **LH­(AlMe**_**2**_ **)** ^*6*^ was tested in 1 mol % loading for the DCM solution of *rac*-lactide at 20 °C, co-initiated by one equivalent of ^*i*^PrOH. These conditions, effective for biguanide Mg- or Zn-systems, did not lead to any polymerization. In conditions commonly used for Al-based ROP, e.q. elevated temperature (90 °C) in toluene, 90% of conversion was measured after 8 h, corresponding to the turnover frequency of 11.25 h^–1^ (Table 1, entry 2). The number-average molar mass (*M*_*n*_) of the obtained PLA (12,200 g·mol^–1^) was in good agreement to the theoretical value (13,000 g·mol^–1^) with a very narrow dispersity of 1.08. The prolongation of the reaction time caused a slight broadening of the molecular weight distribution (*D̵* = 1.24), attributed to probable transesterification reactions occurring after longer heating (Table 1, entry 3). From MALDI-TOF spectra, obtained from a smaller polymeric chain (reaction ratio 25:1:1), it can be concluded that the transesterification products are present in an almost equimolar ratio (Figure S28 in the Supporting Information) and the polymer chain exhibited the ^*i*^PrO- end group.

The bimetallic isomers **L­(AlMe**_**2**_ **)**_**2**_^*4*,*4*^ and **L­(AlMe**_**2**_ **)**_**2**_^*6*,*4*^ were compared in 1 mol % loading using 1 or 2 equiv of ^*i*^PrOH. In a [100:1:1] ratio, both complexes yielded 71% of PLA after 8 h, with a similar mass and dispersity (Table 1, entries 4 and 8). The chain produced by **L­(AlMe**_**2**_ **)**_**2**_^*4*,*4*^ was slightly shorter (5,900 g mol^–1^) than the one produced by **L­(AlMe**_**2**_ **)**_**2**_^*6*,*4*^ (7,300 g mol^–1^), but both were considerably smaller than the theoretical value of 10,200 g mol^–1^. No significant difference in behavior in LA ROP between the isomers was, therefore, observed. The MALDI-TOF analysis revealed a similar profile to the PLA made by monometallic catalyst **LH­(AlMe**_**2**_ **)** ^*6*^ (Figure S29 in the Supporting Information). An increase of the amount of ^*i*^PrOH to be equal to aluminum centers in the [100:1:2] ratio followed with better activity and control for both bimetallic species, with 89% yield of PLA for **L­(AlMe**_**2**_ **)**_**2**_^*4*,*4*^ (Table 1, entry 5) and 93% yield for **L­(AlMe**_**2**_ **)**_**2**_^*6*,*4*^ (Table 1, entry 9). In the [200:1:2] ratio, **L­(AlMe**_**2**_ **)**_**2**_^*4*,*4*^ converted 83% of the lactide in 8 h (Table 1, entry 6), whereas **L­(AlMe**_**2**_ **)**_**2**_^*6*,*4*^ lagged behind with 78% conversion (Table 1, entry 10). In both cases, the produced polymer had a smaller mass (5,900 and 7,400 g mol^–1^, respectively) than the expected value (12,000 and 11,200 g mol^–1^, respectively).

To better understand the difference between **L­(AlMe**_**2**_ **)**_**2**_^*4*,*4*^ and **L­(AlMe**_**2**_ **)**_**2**_^*6*,*4*^, the reactions were performed at a lower temperature (60 °C), but the result was again very similar −10% and 9% of conversion after 8 h, respectively (Table 1, entries 7 and 11). Therefore, in Table 1, entries 4–11, only negligible differences between the performance of **L­(AlMe**_**2**_ **)**_**2**_^*4*,*4*^ and **L­(AlMe**_**2**_ **)**_**2**_^*6*,*4*^ were observed. Moreover, when considering the same lactide/metal/^*i*^PrOH ratio (100:1:1; entries 2, 6, and 10 in Table 1), the activity of all aluminum co-initiators is comparable with the TOF value of 9.75–11.25 h^–1^, meaning that the position of the -AlMe_2_ fragment has only a small effect on the activity of the system. Out of the aluminum species, the monometallic **LH­(AlMe**_**2**_ **)** ^*6*^ controlled the polymer mass the best with an excellent match of the experimental to theoretical value. The tacticity values *P*_r_ = 0.45–0.65 for all polymeric materials was consistent with the low stereoselectivity of the catalysts, caused by the planar nature of the complexes. The boron complexes were inactive in *rac*-lactide ROP both at room temperature (Table 1, entries 12 and 14) and at higher temperatures, under the conditions used for aluminum complexes (Table 1, entries 13 and 15).

### Hydroboration Catalysis

To test the versatility of the obtained catalysts in other reactions, we applied the complexes **LH­(AlMe**_**2**_ **)** ^*6*^, **LH­(BH**_**2**_ **)** ^*6*^, **LH­(BF**_**2**_ **)** ^*6*^, **L­(BH**_**2**_ **)**_**2**_^*6*,*4*^, and **L­(BH**_**2**_ **)**_**2**_^*4*,*4*^ as potential catalysts in model hydroboration reactions of unsaturated C–C bonds (Table S1). Aluminum complexes have already been reported in the literature as catalysts for hydroboration reactions;^14,21,22,50^ therefore, the choice of this transformation was justified. The results obtained for reactions carried out at room temperature were rather similar for all of the selected complexes and non-satisfactory. Taking into the account the presence of borohydrides in the structures of **LH­(BH**_**2**_ **)** ^*6*^, **L­(BH**_**2**_ **)**_**2**_^*6*,*4*^, and **L­(BH**_**2**_ **)**_**2**_^*4*,*4*^, and risk of their release at higher temperatures, we decided that **LH­(AlMe**_**2**_ **)** ^*6*^ was the most suitable candidate for further tests.

Since borohydride itself is known to catalyze hydroboration, we sought to ensure that the observed catalysis was driven by the synthesized complexes rather than by in situ generated hidden boron species.^51–54^ Moreover, control hydroboration reactions with **LH­(AlMe**_**2**_ **)** ^*6*^ and TMEDA excluded the possibility of the formation of a hidden boron catalyst during processes with the utilization of the aluminum complex. The best result was obtained for the addition of pinacolborane (HBpin) to phenylacetylene at 100 °C with 10% catalyst loading, leading to a β-boryl-substituted product in 87% yield (Scheme 4). However, changing the substrate to styrene under the same reaction conditions resulted in a lower conversion to the hydroboration product (55%, Scheme 4). Interestingly, when chalcone was tested, despite the presence of two unsaturated bonds (C=C and C=O) in its structure, pinacolborane did not attach to either of them; instead, the hydrogenation of the C=C bond occurred (Scheme 4). Although **LH­(AlMe**_**2**_ **)** ^*6*^ exhibited catalytic activity in the addition reaction of HBpin to the selected alkene and alkyne, these results do not represent an alternative to catalysts already reported in the literature due to the relatively high catalyst loading and reaction temperature.^55,56^

**Scheme 4:**
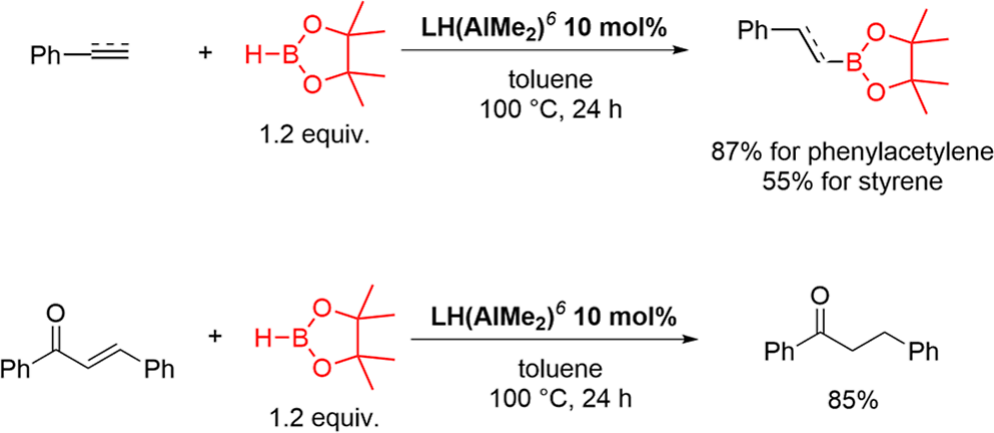
Hydroboration of Phenylacetylene and Styrene in the Presence of **LH­(AlMe**_**2**_ **)** ^*6*^ (Top) and Hydrogenation of Chalcone in the Presence of Pinacolborane and **LH­(AlMe**_**2**_ **)** ^*6*^

## Conclusions

Doubly deprotonable biguanide ligand **LH**_**2**_ was used to prepare several monoanionic complexes, which were found in either a six-membered (azadiketiminate-like) or a four-membered (guanidinate-like) chelating arrangement depending on the coordinated element. Dianionic complexes were also prepared, and these exhibited an even greater variety of structural motifs, combining the six- and four-membered architectures. In the structure of dilithium **L­(Li)**_**2**_^*4*,*6*^, the four-membered chelate is adjacent to the pyrimidine ring, whereas in the diboron **L­(BH**_**2**_ **)**_**2**_^*6*,*4*^ and dialuminum **L­(AlMe**_**2**_ **)**_**2**_^*6*,*4*^ and **L­(AlMeCl)**_**2**_^*6*,*4*^ the four-membered chelate includes the guanidinate carbon atom. Structural isomerism was found for bis­(boron) and bis­(aluminum) species bearing the dianionic ligand, where by opting for different reaction conditions, two distinct isomers can be prepared. This phenomenon was extensively experimentally and theoretically studied, exhibiting both thermodynamic and kinetic consequences. Regarding lactide polymerization activity, both mono- and bimetallic biguanide-AlMe_2_ complexes, in combination with ^*i*^PrOH, were found to be active catalysts for lactide ROP, affording atactic PLA. The highest activity and best control were achieved with the monometallic complex **LH­(AlMe**_**2**_ **)** ^*6*^. Selected aluminum complexes also showed activity in the hydroboration of the unsaturated C–C bonds, confirming the versatility of the synthesized catalytic systems.

## Materials and Methods

Synthesis of **LH**_**2**_ was described elsewhere.^15^ All solvents and chemical reagents were purchased from commercial sources and used without further purification except for 4,4,5,5-tetramethyl-1,3,2-dioxaborolane, which was purified by vacuum distillation. Most synthetic procedures were performed using the standard Schlenk techniques under an inert argon atmosphere (99.999%) (inert gas was passed through the oxygen/moisture trap Supelco before entering the vacuum/inert line) and solvents were dried with the help of solvent purification system PureSolv MD 7 supplied by Innovative Technology, Inc., degassed, and then stored under an argon atmosphere over a potassium or sodium mirror, if needed. The exceptions were air-stable monoanionic boron complexes **LH­(BH**_**2**_ **)** ^*6*^ and **LH­(BF**_**2**_ **)** ^*6*^. Synthetic protocols and characterization of all complexes are given in detail in Supporting Information file pp. S28–S37. Single crystals suitable for X-ray analyses were obtained from the corresponding saturated solutions of products in organic solvent(s) (Et_2_O, hexane, Et_2_O/hexane (1:1) mixture, toluene, THF) at room temperature or cooled to 7 or −30 °C. Deuterated solvents for NMR spectroscopy were distilled, degassed, and stored over a K or Na-mirror under an argon atmosphere.

Mass spectra of PLA samples were acquired on a time-of-flight mass spectrometer (MALDI-ToF-Microflex LRF, Bruker Daltonics). An external quadratic multipoint calibration was carried out before each measurement using polyethylene glycol (PEG) mixed in THF with dithranol (DIT). Analysis was performed with DIT as the matrix (10 mg/mL) and sodium trifluoroacetate (10 mg/mL) as an additive. The polymer (10 mg/mL), the matrix, and the additive were mixed in a volumetric ratio of 1/1/0.5 in THF. All of the analyses were performed in the positive reflectron mode.

GC analyses of crude mixture of hydroboration reactions were performed on a Bruker Scion 436-GC with a Megabore column (30 m Agilent VF5 ms 0.53 mm) and TCD (temperature program: 60 °C (3 min), 60 to 250 °C (10 °C/min), 250 °C (5 min) using decane as an internal standard. The mass spectra were obtained by GC–MS analysis on a Varian 431-GC with a 30 m Agilent J&W VF-200 ms 0.25 mm capillary column and a Varian 220-MS mass spectrometry detector temperature program: 60 °C (3 min), 60–280 °C (10 °C/min), 280 °C (8 min).

### NMR Spectroscopy

NMR spectra were recorded from solutions of appropriate compounds in deuterated solvent(s) on a Bruker Avance 500 spectrometer (equipped with a Z-gradient 5 mm Prodigy cryoprobe) at frequencies for ^1^H (500.13 MHz), ^13^C­{^1^H} (125.76 MHz), ^7^Li­{^1^H} (194.34 MHz), ^11^B and ^11^B­{^1^H} (160.43 MHz) and ^19^F­{^1^H} (470.93 MHz) or a Bruker UltraShield 400 spectrometer at frequencies for ^1^H (400.13 MHz), ^13^C­{^1^H} (100.62 MHz), ^7^Li­{^1^H} (155.49 MHz), ^11^B (128.34 MHz), ^11^B­{^1^H} (128.34 MHz), and ^19^F­{^1^H} (376.75 MHz) at 295 K or in some cases at various temperatures. Variable-temperature NMR experiments were performed on a Bruker UltraShield 400 spectrometer with a triple resonance broad-band probe (5 mm TBO BB-1H/D Z-GRD) operating at 400.16 MHz for ^1^H. Solutions were obtained by dissolving approximately 40 mg of each compound in approximately 0.6 mL of deuterated solvents. Values of ^1^H chemical shifts were referenced to residual signals of benzene (δ­(^1^H) = 7.16), tetrahydrofuran (δ­(^1^H) = 1.73) or toluene (δ­(^1^H) = 2.09). Values of ^13^C chemical shifts were calibrated to signals of benzene (δ­(^13^C) = 128.4), tetrahydrofuran (δ­(^13^C) = 67.6), or toluene (δ­(^13^C) = 20.4). All ^13^C NMR spectra were measured using a standard proton-decoupled experiment and CH/CH_3_ vs C_q_/CH_2_ were sometimes differentiated with the APT method’s help.^57^ NMR signal assignment was supported by selective 1D 1H selective gradient NOESY, ^1^H,^1^H-NOESY, ^1^H,^13^C-HSQC or ^1^H,^13^C-HMBC 2D spectra. The ^1^H and ^13^C NMR spectra of products of hydroboration were recorded on a Bruker Ultrashield 300 MHz or Bruker Ascend 400 MHz spectrometers. Chemical shifts were referenced to the residual chloroform peak.

Infrared (single-bounce diamond ATR) and Raman (vacuum-sealed capillary excitation laser at 1064 nm) spectra were recorded on a Nicolet iS50 FTIR spectrometer equipped with the iS50 Raman module.

## Crystallography

The X-ray data for colorless crystals of all complexes were obtained at 150 K using an Oxford Cryostream low-temperature device with a Bruker D8-Venture diffractometer equipped with a Mo (Mo/K_α_ radiation; λ = 0.71073 Å) microfocus X-ray (IμS) source, photon CMOS detector, and Oxford Cryosystems cooling device. Obtained data were treated by XT-version 2014/5 and SHELXL-2017/1 software implemented in an APEX4 v2019.4–0 (Bruker AXS) system.^58^ *R*_int_ = ∑|*F*_o_^2^ – *F*_o,mean_^2^|/∑*F*_o_^2^, S = [∑(*w*(*F*_o_^2^ – *F*_c_^2^)^2^)/(*N*_diffrs_ – *N*_params_)]^1/2^ for all data, *R*(*F*) = ∑||*F*_o_| – |*F*_c_||/∑|*F*_o_| for observed data, *wR*(*F* ^2^) = [∑(*w*(*F*_o_^2^ – *F*_c_^2^)^2^)/(∑*w*(*F*_o_^2^)^2^)]^1/2^ for all data. Crystallographic data for all structural analysis has been deposited with the Cambridge Crystallographic Data Centre, CCDC nos. 2526577–2526589.

Copies of this information may be obtained free of charge from The Director, CCDC, 12 Union Road, Cambridge CB2 1EY, UK (fax: +44-1223-336033; e-mail: deposit@ccdc.cam.ac.uk; Web site: http://www.ccdc.cam.ac.uk). Data were corrected for absorption effects using the Multi-Scan method (SADABS). The structures were solved and refined using the Bruker SHELXTL software package.

Hydrogen atoms were mostly localized on a difference Fourier map, however, to ensure uniformity of treatment of crystal, most of the hydrogen atoms were recalculated into idealized positions (riding model) and assigned temperature factors H_iso_(H) = 1.2 U_eq_ (pivot atom) or of 1.5U_eq_ (methyl). H atoms in methyl, methylene, methine moieties, and C–H in aromatic rings were placed with C–H distances of 0.96, 0.97, 0.98, and 0.93 Å. Hydrogen atoms in NH groups were added freely according the maxima on the difference Fourier map. Minor disorders were treated by standard methods.

## Computation

All the calculations were performed with the Gaussian 16 program.^59^ The geometries of all compounds and their selected isomers as well as byproducts and starting materials were fully optimized at B3LYP/6-311 + G­(d,p) or B3LYP/def2-TZVP for APT charges, levels of theory^60–63^ without any structure simplifications. The structure of complexes obtained by X-ray diffraction were mostly used as the input structures. The conductor-like polarizable continuum model (CPCM)^64^ was employed for the solvation effects (THF, benzene). The empirical dispersion correction D3 was applied for all structures.^65^ All the structures are minima on the potential energy surface, as confirmed by the frequency calculations at the same level of theory and transition states by only one imaginary frequency.

### Typical PLA Synthesis Procedure in Solution

Under an argon atmosphere, to a solution of *rac*-lactide (288.3 mg; 100 equiv; 2.00 mmol) in the chosen solvent (1.3 mL; [LA]_0_ = 1.00 M), 1 equiv of catalyst, and 1 equiv of co-initiator ^*i*^PrOH (1.53 μL; 0.02 mmol) were added. The vial was crimped and let under stirring at the desired temperature. The reaction vial was opened to air in order to quench the reaction. The conversion was determined by ^1^H NMR spectroscopy and the molar mass and dispersity by gel permeation chromatography (GPC). The PLA provided can be washed with methanol in order to remove the residual monomer and catalyst.

### Typical Hydroboration Synthesis Procedure in Solution

Under an argon atmosphere, to a solution of olefin or alkyne (0.125 mmol) in the chosen solvent (neat or toluene), 1.2 equiv of HBpin (0.021 mL; 0.150 mmol) and the chosen amount of catalyst (0.013 mmol) were introduced. The reaction was allowed to stir at the desired temperature. The conversion and yield were determined by GC–MS analysis.

## Supplementary Material







## Notes

Synthetic procedures and structural issues of the complexes, reactivity studies, polymerization results, hydroboration results, crystallographic parameters and computational methods, and NMR spectra of prepared compounds (PDF)
CIF and MOL files (ZIP)
FIDs of NMR measurements (PDF)
