# Supplementary material for: Biguanide Complexes of Boron and Aluminum
Source: Inorg Chem. 2026 Jun 4;65(24):13350–61. doi: 10.1021/acs.inorgchem.6c00595 (PMC13292212; doi:10.1021/acs.inorgchem.6c00595)
Supplement: Supplementary file 1 [file ic6c00595_si_001.pdf]

Biguanide Complexes of Boron and Aluminum

Lukáš Vlk,† Tomáš Chlupatý,†* Alena Hoffmannová,† Zdeňka Růžičková,† Aleksandra Szymańska,#§ Benjamin Théron,‡ Raluca Malacea-Kabbara,‡ Pierre Le Gendre,‡* Jędrzej Walkowiak,# Aleš Růžička†*

†Department of General and Inorganic Chemistry, Faculty of Chemical Technology, University of Pardubice, Studentská 573, Pardubice 532 10, Czech Republic

‡Univ. Bourgogne Europe, Institut de Chimie Moléculaire de l'Université de Bourgogne (ICMUB), UMR CNRS 6302, 9 Avenue Alain Savary, 21078 Dijon, France

#Center for Advanced Technologies, Adam Mickiewicz University, Uniwersytetu Poznańskiego 10, 61-614 Poznań, Poland

§Faculty of Chemistry, Adam Mickiewicz University, Uniwersytetu Poznańskiego 8, 61-614 Poznań, Poland

*tomas.chlupaty@upce.cz, pierre.le-gendre@u-bourgogne.fr, ales.ruzicka@upce.cz

TABLE OF CONTENTS

SYNTHETIC ASPECTS AND STRUCTURAL BEHAVIOR....pages S2-S17
- Alternative synthesis of **LH(AlCl$_2$)**^{6} and **LH(BF$_2$)**^{6} from **LH(Li)**^{4} - Scheme S1....page S2
- Synthesis of **LH(AlMeI)**^{6} and **LH(AlI$_2$)**^{6} - Scheme S2....page S2
- Reactivity of mono- and dialuminium complexes - Scheme S3....page S2
- View of the paralell offset π-π stacking for **LH(AlX$_2$)**^{6} and **LH(BX$_2$)**^{6} - Figure S1....page S3
- VT ^{1}H NMR spectra - Figures S2-S4....pages S4-S6
- Temperature-dependent isomerization of **L(AlMe$_2$)$_2$**^{4,4} to **L(AlMe$_2$)$_2$**^{6,4} -Figure S5....page S7
- Molecular structures - Figures S6-S18....pages S8-S15

POLYMERIZATION....pages S16–S21
- Representative ^{1}H NMR spectrum of the methine region of crude PLA – Figure S19....page S16
- Representative GPC spectra of crude PLA – Figures S20-S27....pages S16-S19
- Representative MALDI-TOF spectra of PLA – Figures S28-S30....pages S20-S21

HYDROBORATION....pages S22-S27
- General procedure for tests of catalytic activity....page S22
- Hydroboration of PhC≡CH and styrene – Table S1....page S22
- Characterization of synthesized products....page S23
- NMR spectra of synthesized products – Figures S31-S34....pages S24-S27

SYNTHESIS§§....pages S28–S37

CRYSTALLOGRAPHY....pages S38-S50
- Crystal data and structure refinement – Tables S2-S14....pages S38-S50

THEORY....pages S51-S54
- Optimized structures and calculated APT charges for **LH(AlX$_2$)** – Figure S35....page S51
- Optimized structures and calculated relative Gibbs' free energies – Figures S36-S41....pages S52-S54

INFRARED AND RAMAN SPECTRA....pages S55-S59
- Spectra of **LH(BH$_2$)**^{6}, **L(BH$_2$)$_2$**^{6,4} and **L(BH$_2$)$_2$**^{4,4} – Figures S42-S50.... pages S55-S59

§§ NMR spectra of all prepared compounds are given in separate pdf. file

SYNTHETIC ASPECTS AND STRUCTURAL BEHAVIOR

Additionally, to the deprotonation reaction of **LH_2**, **$LH(AlCl_2)$**[6] and **$LH(BF_2)$**[6] were also prepared from **LH(Li)**[4] *via* transmetallation (Scheme S1) with the help of small LiX molecule elimination.

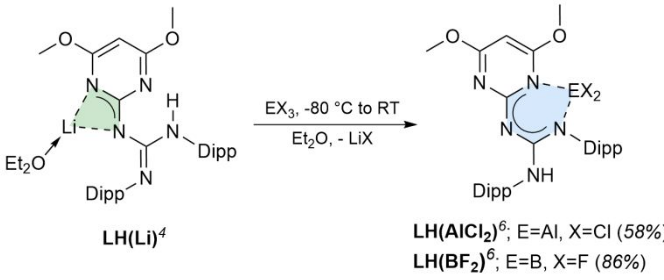

Scheme S1. Synthesis of **$LH(AlCl_2)$**[6] and **$LH(BF_2)$**[6] from **LH(Li)**[4]. Isolated yields are given in parentheses.

The reaction of **$LH(AlMe_2)$**[6] with 1 equivalent of I_2 was performed in toluene for 2 hours at 60°C (Scheme S2). The reaction mixture was filtered off, filtrate was slowly evaporated to crystallize crude product, which after washing with Et_2O yielded **LH(AlMeI)**[6]. Substitution of second Me group was performed sequentially from isolated **LH(AlMeI)**[6]. Alternatively, direct substitution from **LH_2** with 2 eq. of iodine (Scheme S2) was also investigated. In both cases, reaction mixture needs to be heated to 60 °C for 72 hours with subsequent solvent evaporation to get yellow crystalline solid of **$LH(AlI_2)$**[6].

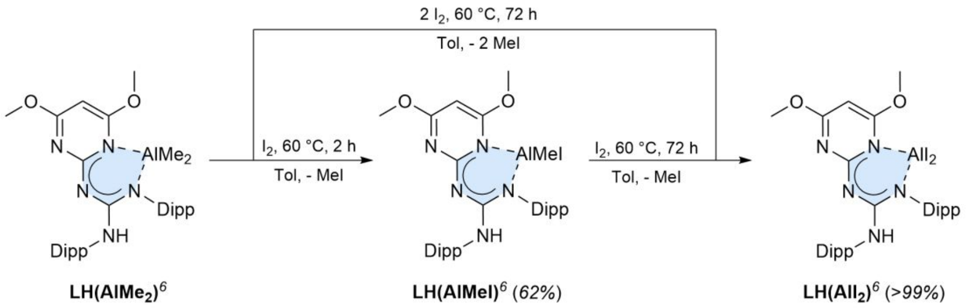

Scheme S2. Synthesis of **LH(AlMeI)**[6] and **$LH(AlI_2)$**[6]. Isolated yields are given in parentheses.

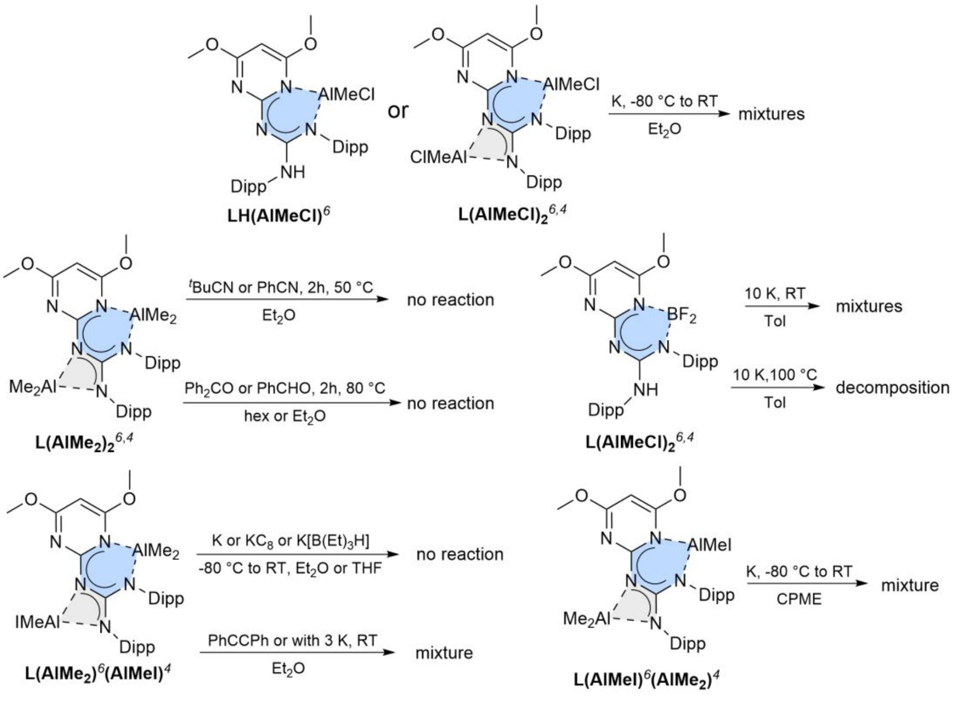

Scheme S3. Reactivity of prepared mono- and dialuminium complexes towards reduction agents and (or) unsaturated CC, CN, CO bonds.

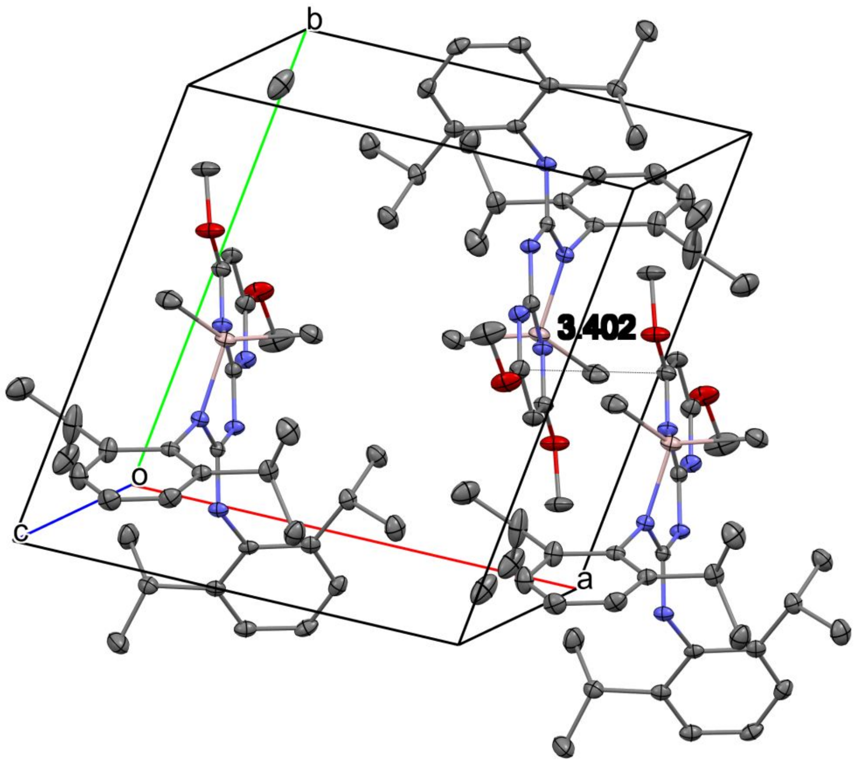

Figure S1. View of the paralell offset π-π stacking present in the solid state for **LH(AlMe$_2$)**[6]. The same properties are observed for **LH(AlMeCl)**[6], **LH(AlCl$_2$)**[6], **LH(AlMeI)**[6] and **LH(AlI$_2$)**[6].

The solid-state parameters of the molecules of monoanionic **LH(AlX$_2$)**[6] and **LH(BX$_2$)**[6] type compounds are very similar. Only negligible change in the geometry of the biguanide moiety can be detected. In terms of intramolecular interactions, a strong parallel offset π-π stacking (3.3–3.4 Å) is observed for the pyrimidine rings in all **LH(AlX$_2$)**[6] species in solid state (Figure S1), whereas the related distance between parallel pyrimidine units in boron analogue **LH(BH$_2$)**[6] is around 7.5 Å due to additional BH- interactions to CH_3 group of the Dipp substituent. In **LH(BF$_2$)**[6], the halide atoms interact with methoxy- group CH_3 hydrogen from a neighboring molecule.

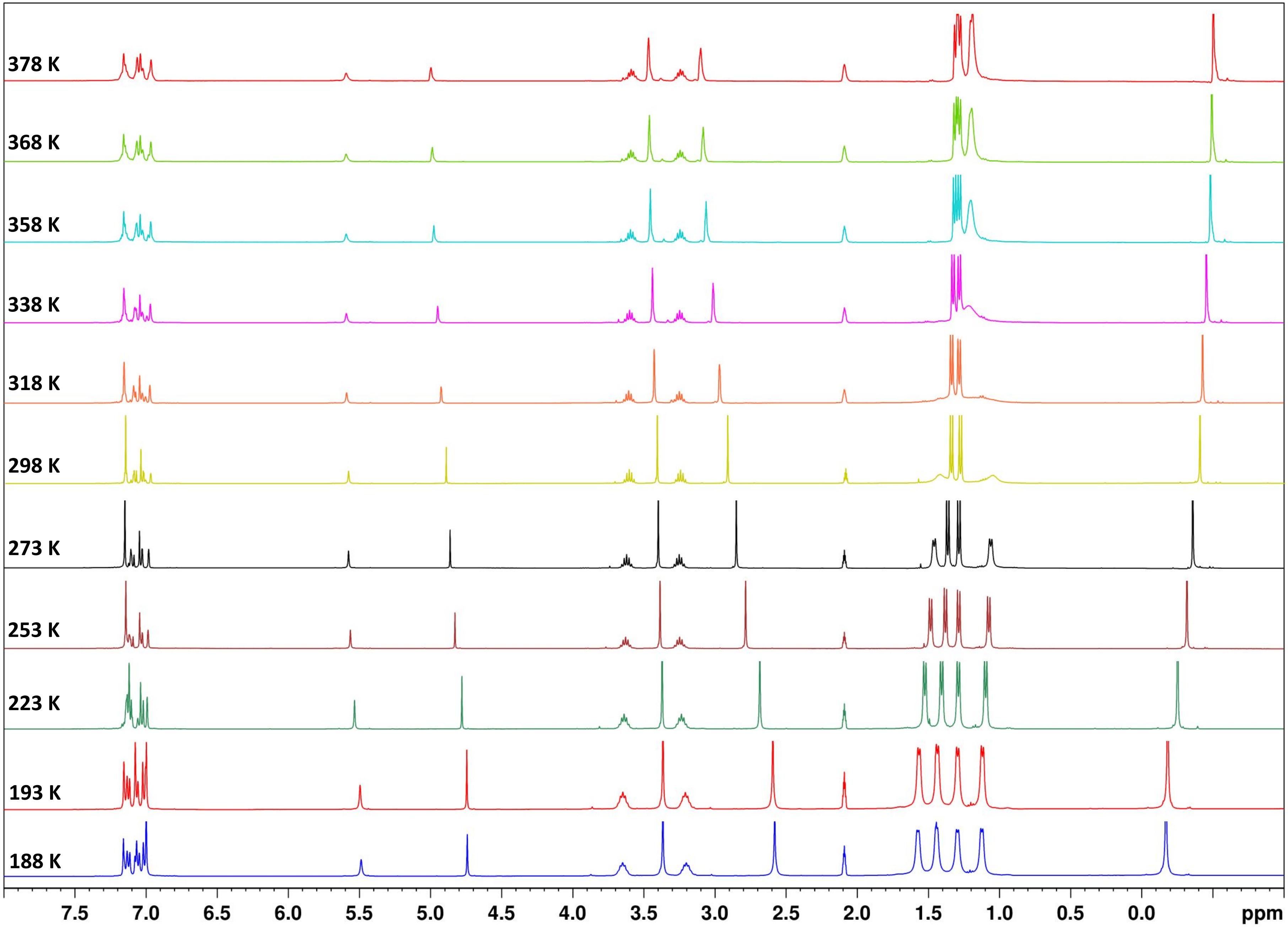

Figure S2. VT ^{1}H NMR spectra of **LH(AlMe$_2$)**[6] in Tol-d$_8$, 188–378 K.

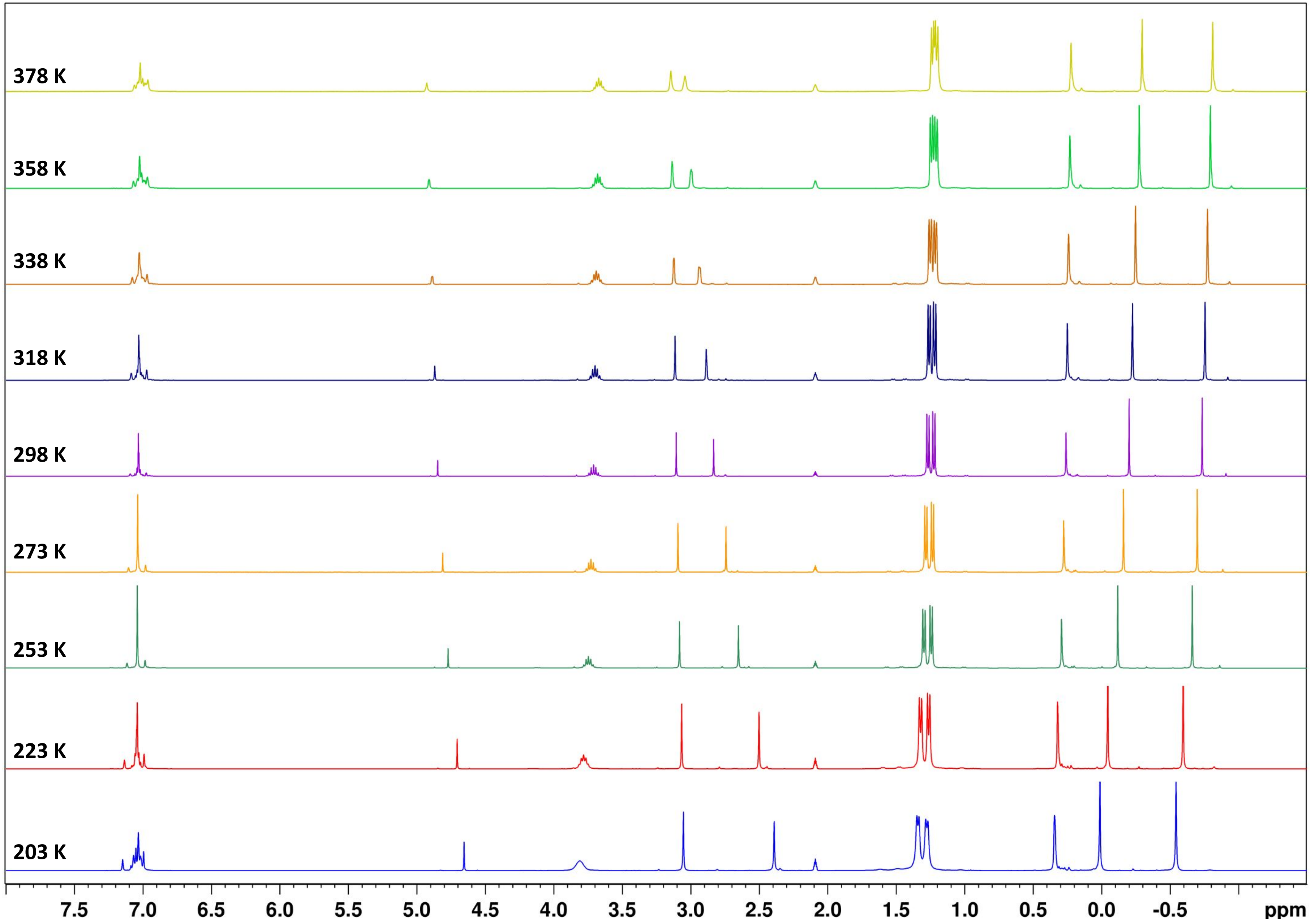

Figure S3. VT ^{1}H NMR spectra of **L(AlMe$_2$)$_2$**^{4,4} in Tol-d$_8$, 203–378 K.

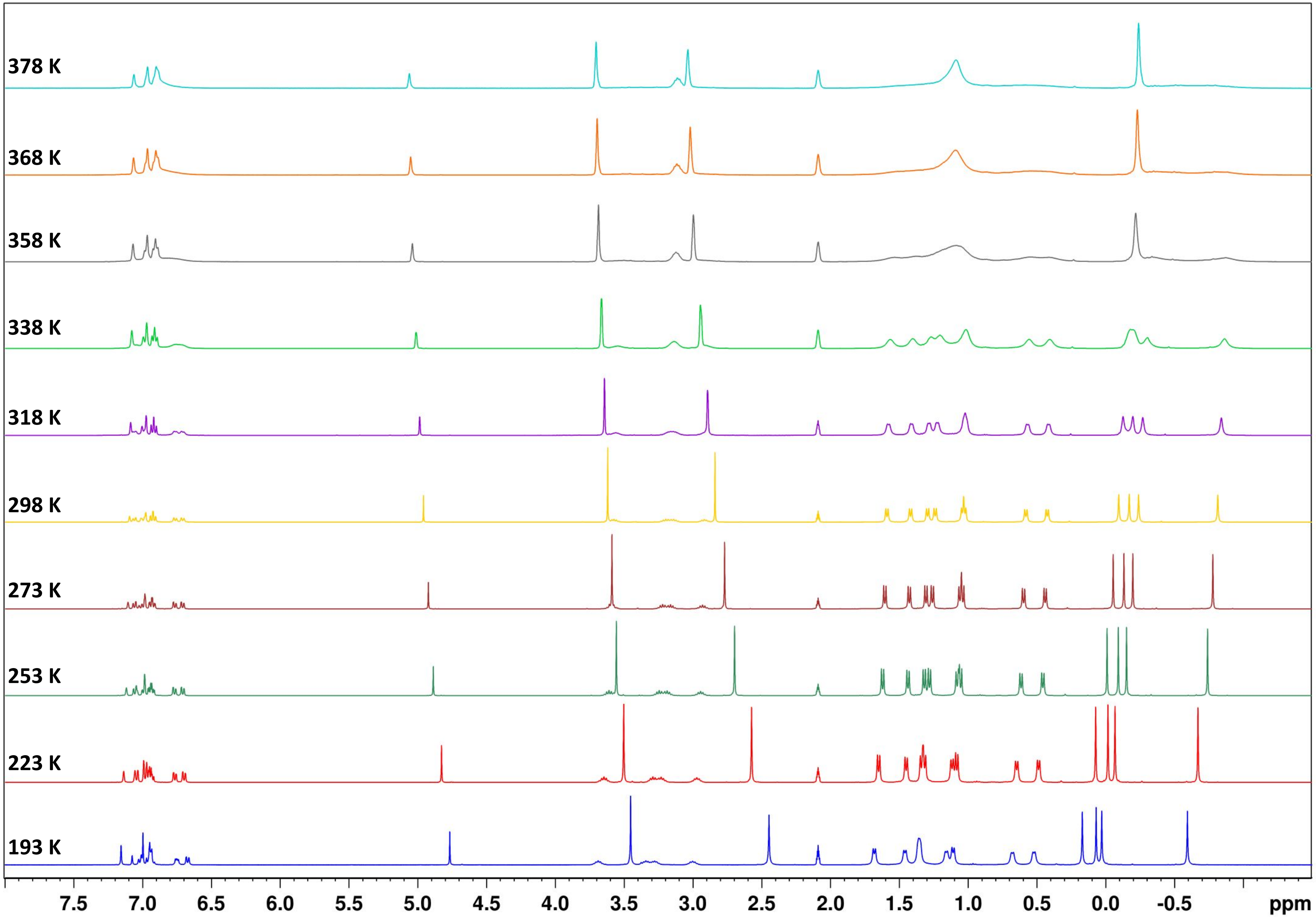

Figure S4. VT ^{1}H NMR spectra of **L(AlMe$_2$)$_2$***[6,4]* in Tol-d$_8$, 193–378 K.

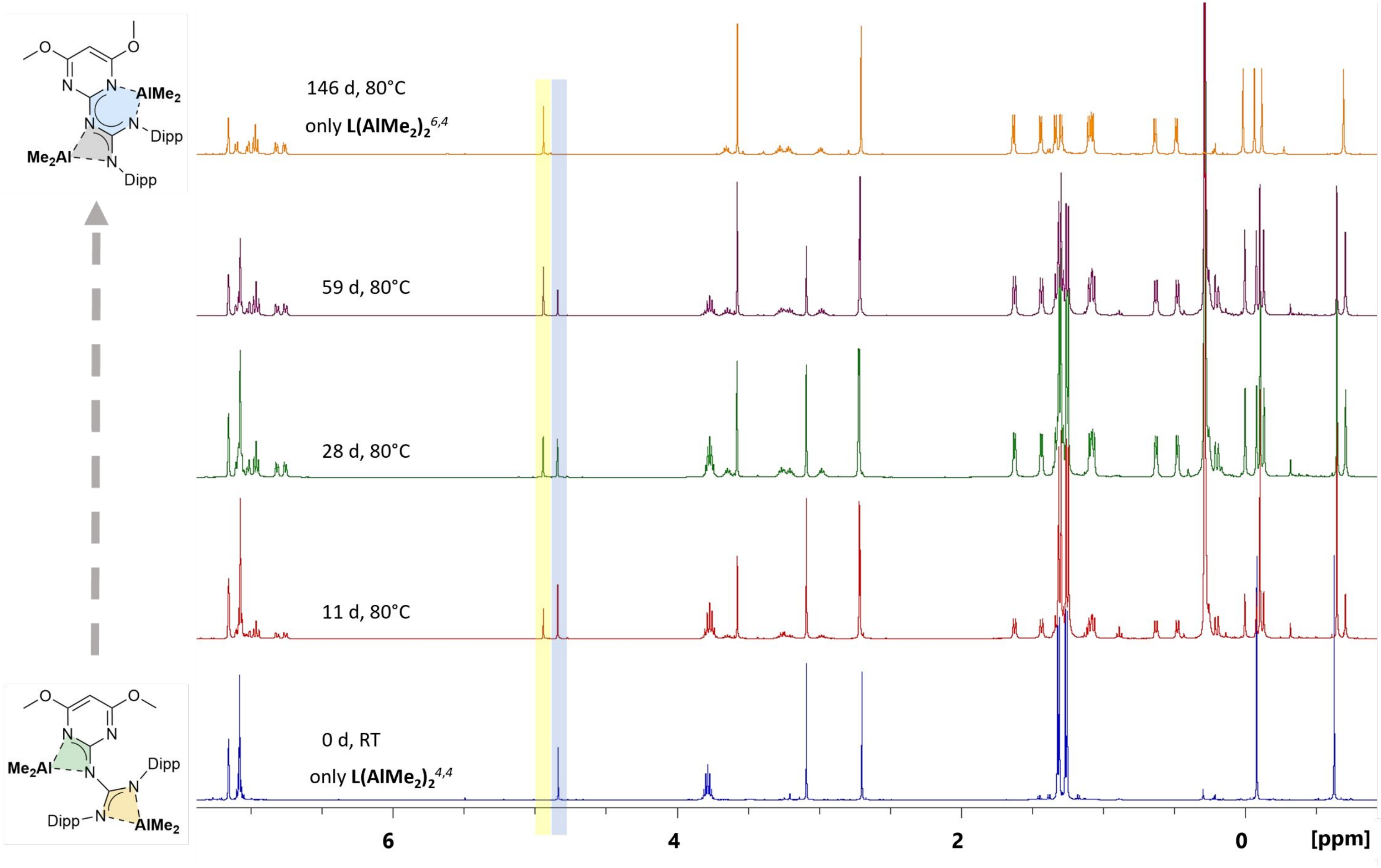

Figure S5. Progress of temperature-dependent isomerization process of **$L(AlMe_2)_2$**^{4,4} (blue box for ArH^{Prm} signal) to **$L(AlMe_2)_2$**^{6,4} (yellow box for ArH^{Prm} signal) in C_6D_6 solution monitored by ^1H NMR spectroscopy in sealed NMR tube.

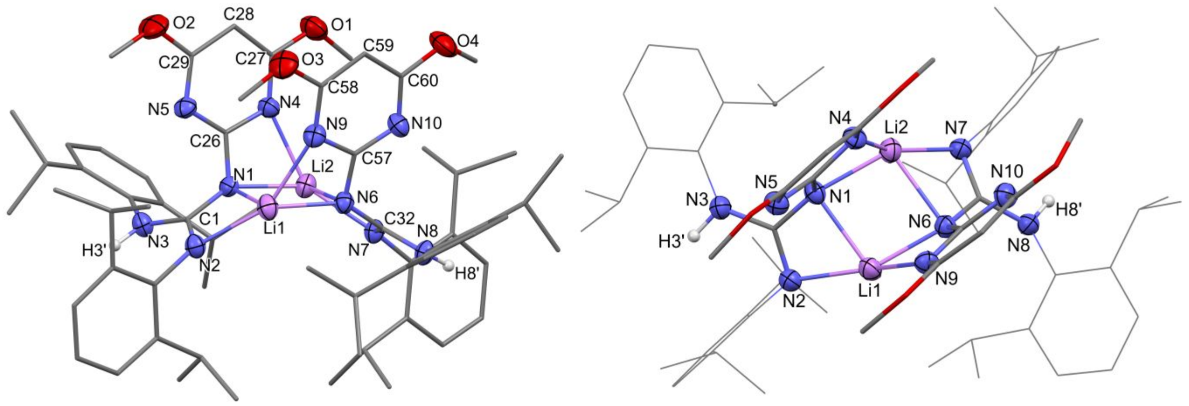

Figure S6. The molecular structure of **[LH(Li)^{4}]$_2$**, ORTEP view 50% probability level, one of two independent molecules (left) and the detail of the ladder-type coordination of **[LH(Li)^{4}]$_2$** (bottom). Hydrogen atoms, except for NH groups, are omitted for clarity. Selected bond lengths (Å) and angles (°): Li1–N1 2.190(9), Li1–N2 2.078(9), Li1–N6 2.022(9), Li1–N9 2.234(9), Li2–N1 2.035(9), Li2–N4 2.162(9), Li2–N6 2.182(9), Li2–N7 2.054(9), Li3–N11 2.215(9), Li3–N12 2.037(9), Li3–N16 2.041(9), Li3–N19 2.180(9), Li4–N11 2.017(9), Li4–N14 2.162(9), Li4–N16 2.186(8), Li4–N17 2.074(9), N1–Li1–N2 64.6(3), N1–Li2–N4 64.9(3), N6–Li1–N9 64.0(3), N6–Li2–N7 65.4(3), N11–Li3–N12 65.5(3), N11–Li4–N14 64.9(3), N16–Li3–N19 64.8(3), N16–Li4–N17 65.4(3), N1–Li1–N6 96.4(3), N1–Li1–N9 107.6(4), N1–Li2–N6 96.2(3), N1–Li2–N7 142.8(4), N11–Li3–N16 94.8(3), N11–Li3–N19 108.9(4), N11–Li4–N16 96.4(3), N11–Li4–N17 142.1(4), N2–Li1–N6 141.3 (4) N2–Li1–N9 151.7(5), N4–Li2–N6 106.1(4), N4–Li2–N7 149.0(4), N12–Li3–N16 140.5(5), N12–Li3–N19 152.3(5), N14–Li4–N16 105.7(4), N14–Li4–N17 149.6(4), C1–N2 1.294(6), C1–N3 1.369(6), N1–C1 1.378(6), N1–C26 1.350(6), N4–C26 1.371(6), N5–C26 1.352(6), N7–C32 1.299(5), N8–C32 1.369(6), N6–C32 1.383(6), N6–C57 1.342(6), N9–C57 1.370(6), N10–C57 1.358(6), N12–C101 1.297(5), N13–C101 1.365(6), N11–C101 1.392(5), N11–C126 1.335(5), N14–C126 1.367(5), N15–C126 1.348(6), N17–C132 1.309(6), N18–C132 1.367(6), N16–C132 1.377(6), N16–C157 1.346(6), N19–C157 1.368(6), N20–C157 1.348(6), N2–C1–N1 117.4(4), N3–C1–N1 119.6(4), N3–C1–N2 122.8(4), C26–N1–C1 120.9(4), N1–C26–N5 122.2(4), N1–C26–N4 112.0(4), N5–C26–N4 125.7(4), N6–C32–N7 117.4(4), N6–C32–N8 118.6(4), N7–C32–N8 123.8(4), C32–N6–C57 119.6(4), N6–C57–N9 113.0(4), N6–C57–N10 121.6(4), N9–C57–N10 125.1(4), N12–C101–N11 117.9(4), N13–C101–N11 118.2(4), N13–C101–N12 123.6(4), C126–N11–C101 118.9(4), N11–C126–N15 121.8(4), N11–C126–N14 112.4(4), N15–C126–N14 125.6(4), N16–C132–N17 118.2(4), N16–C132–N18 119.1(4), N17–C132–N18 122.4(4), C132–N16–C157 120.8(4), N16–C157–N19 113.0(4), N16–C157–N20 121.6(4), N19–C157–N20 125.3(4)., N5–C29 1.324(6), N4–C27 1.323(6), N9–C58 1.338(6), N10–C60 1.325(6), N14–C127 1.328(6), N15–C129 1.334(6), N19–C158 1.305(7), N20–C160 1.334(7), C27–C28 1.386(7), C28–C29 1.372(7), C58–C59 1.372(7), C59–C60 1.380(8), C127–C128 1.391(7), C128–C129 1.377(7), C158–C159 1.385(8), C159–C160 1.373(8), O1–C27 1.351(6), O2–C29 1.359(6), O3–C58 1.342(6), O4–C60 1.362(6), O5–C127 1.347(6), O6–C129 1.383(6), O7–C161 1.258(8), O8–C160 1.345(7).

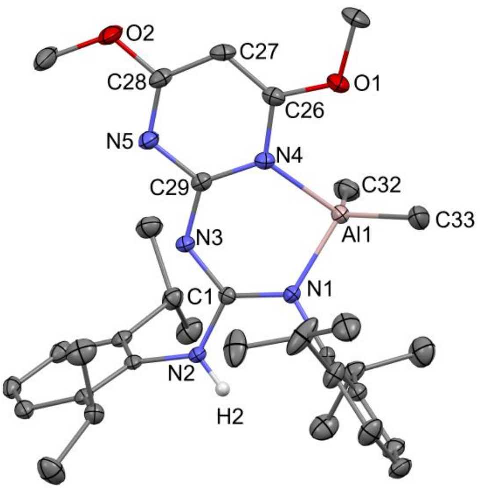

Figure S7. The molecular structure of **LH(AlMe₂)**[6] (ORTEP view 50% probability level). Hydrogen atoms, except for NH groups, are omitted for clarity. Selected bond lengths (Å) and angles (°): Al1–N1 1.9045(13), Al1–N4 1.9556(13), N1–Al1–N4 91.55(5), Al1–C32 1.9720(18), Al1–C33 1.9485(18), C1–N1 1.3379(19), C1–N2 1.3628(19), C1–N3 1.3420(19), C29–N3 1.3365(19), C29–N4 1.367(2), C29–N5 1.3623(19), N2–C1–N1 119.37(13), N3–C1–N1 126.88(13), N3–C1–N2 113.75(13), C29–N3–C1 124.89(13), N3–C29–N5 113.71(13), N3–C29–N4 124.49(13), N5–C29–N4 121.80(13), C26–N4 1.3590(19), C26–C27 1.362(2), C27–C28 1.396(2), C28–N5 1.316(2), C26–O1 1.341(2), C28–O2 1.3394(19), N2–N4 2.766(2).

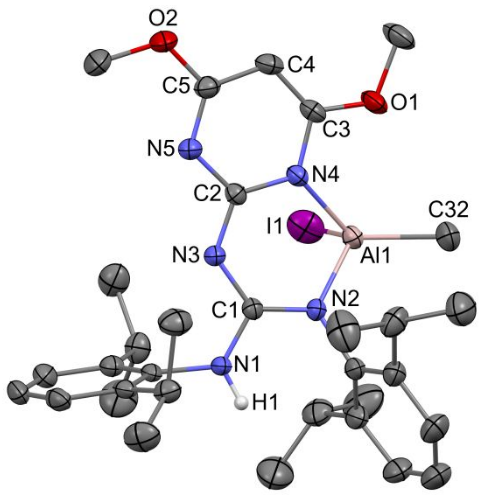

Figure S8. The molecular structure of **LH(AlMeI)**[6] (ORTEP view 50% probability level). Hydrogen atoms, except for NH groups, are omitted for clarity. Selected bond lengths (Å) and angles (°): Al1–N2 1.8705(19), Al1–N4 1.9141(19), N2–Al1–N4 94.14(8), Al1–C32 1.955(3), Al1–I1 2.5739(7), C1–N1 1.357(3), C1–N2 1.344(3), C1–N3 1.341(3), C2–N3 1.335(3), C2–N4 1.368(3), C2–N5 1.354(3), N2–C1–N1 119.45(18), N3–C1–N1 114.13(18), N3–C1–N2 126.42(19), C2–N3–C1 125.32(19), N3–C2–N5 114.23(19), N3–C2–N4 124.50(19), N5–C2–N4 121.27(19), C3–N4 1.361(3), C3–C4 1.357(3), C4–C5 1.396(3), C5–N5 1.315(3), C3–O1 1.333(3), C5–O2 1.337(3), N2–N4 2.771(2).

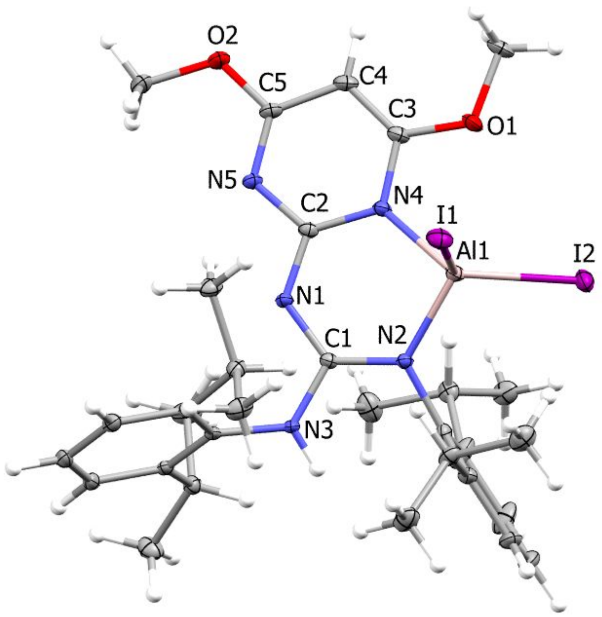

Figure S9. The molecular structure of **LH(AlI$_2$)**[6] (ORTEP view 50% probability level). Hydrogen atoms, except for NH groups, are omitted for clarity. Selected bond lengths (Å) and angles (°): Al1–N2 1.862(2), Al1–N4 1.905(2), N2–Al1–N4 94.49(10), Al1–I1 2.5525(9), Al1–I2 2.4145(10), C1–N1 1.340(3), C1–N2 1.351(3), C1–N3 1.351(3), C2–N1 1.341(3), C2–N4 1.370(3), C2–N5 1.357(3), N2–C1–N1 126.3(2), N3–C1–N1 114.6(2), N3–C1–N2 119.2(2), C2–N1–C1 125.5(2), N1–C2–N4 123.9(2), N1–C2–N5 114.0(2), N4–C2–N5 122.1(2), C3–N4 1.370(3), C3–C4 1.349(4), C4–C5 1.419(4), C5–N5 1.314(3), C3–O1 1.338(4), C5–O2 1.325(3), N2–N4 2.766(3).

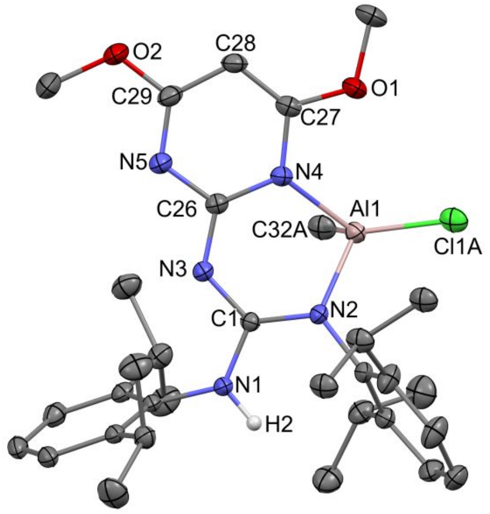

Figure S10. The molecular structure of **LH(AlMeCl)**[6] (ORTEP view 50% probability level). Hydrogen atoms, except for NH groups, are omitted for clarity. Selected bond lengths (Å) and angles (°): Al1–N2 1.8752(16), Al1–N4 1.9172(17), N2–Al1–N4 94.03(7), Al1–C32A 2.17(2), Al1–Cl1 2.1484 (13), C1–N1 1.355(2), C1–N2 1.346(2), C1–N3 1.339(2), C26–N3 1.335(2), C26–N4 1.376(2), C26–N5 1.358(2), N2–C1–N1 119.01 (15), N3–C1–N1 114.26 (15), N3–C1–N2 126.73 (16), C26–N3–C1 125.53 (16), N3–C26–N5 114.19 (16), N3–C26–N4 124.23 (16), N5–C26–N4 121.57 (16), C27–N4 1.361(2), C27–C28 1.359(3), C28–C29 1.405(3), C29–N5 1.316(2), C27–O1 1.341(2), C29–O2 1.335(2), N2–N4 2.774(2).

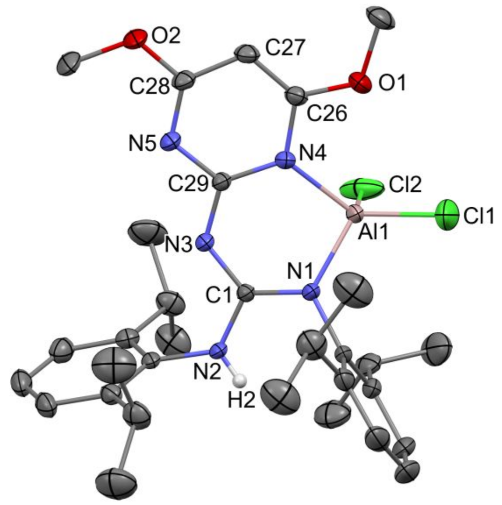

Figure S11. The molecular structure of **LH(AlCl$_2$)**[6] (ORTEP view 50% probability level). Hydrogen atoms, except for NH groups, are omitted for clarity. Selected bond lengths (Å) and angles (°): Al1–N1 1.8541(18), Al1–N4 1.8988(19), N1–Al1–N4 94.47(8), Al1–Cl1 2.1114(11), Al1–Cl2 2.1447(12), C1–N1 1.356(3), C1–N2 1.348(3), C1–N3 1.332(3), C29–N3 1.341(3), C29–N4 1.375(3), C29–N5 1.351(3), N2–C1–N1 117.96(18), N3–C1–N1 126.46(18), N3–C1–N2 115.57(18), C29–N3–C1 125.06(18), N3–C29–N5 114.85(18), N3–C29–N4 123.53(18), N5–C29–N4 121.59(18), C26–N4 1.370(3), C26–C27 1.355(3), C27–C28 1.406(3), C28–N5 1.320(3), C26–O1 1.334(3), C28–O2 1.326(3), N2–N4 2.755(2).

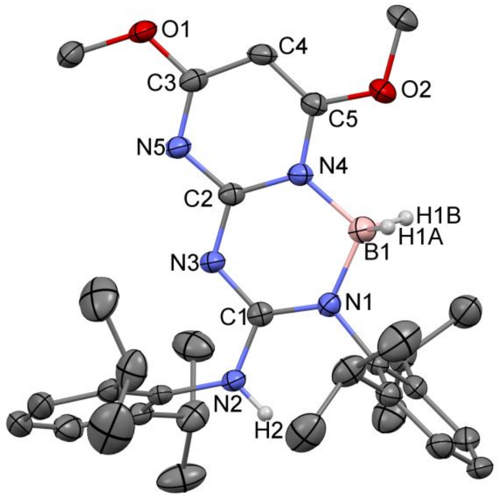

Figure S12. The molecular structure of **LH(BH$_2$)**[6] (ORTEP view 50% probability level). Hydrogen atoms, except for NH and BH$_2$ groups, are omitted for clarity. Selected bond lengths (Å) and angles (°): B1–N1 1.567(2), B1–N4 1.581(2), N1–B1–N4 106.69(12), C1–N1 1.3273(18), C1–N2 1.3557(19), C1–N3 1.3405(19), C2–N3 1.3394(18), C2–N4 1.3679(18), C2–N5 1.3478(19), N2–C1–N1 119.96(13), N3–C1–N1 124.71(13), N3–C1–N2 115.33(12), C2–N3–C1 121.06(12), N3–C2–N5 114.95(12), N3–C2–N4 122.39(13), N5–C2–N4 122.65(13), C5–N4 1.3600(18), C4–C5 1.358(2), C3–C4 1.393(2), C3–N5 1.3136(19), C3–O1 1.3309(18), C5–O2 1.3362(18), N1–N4 2.526(2).

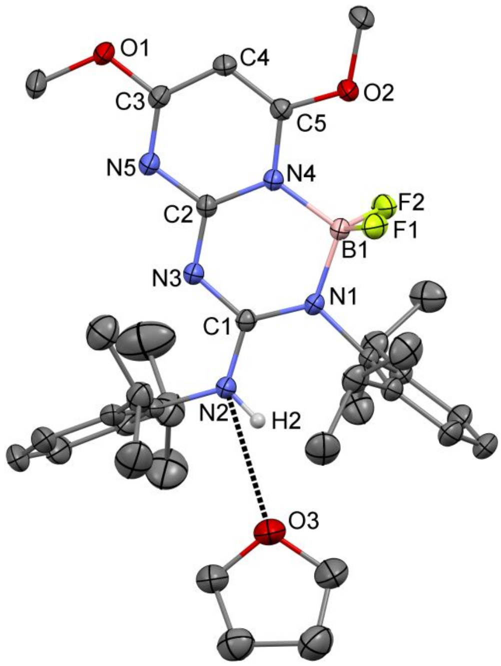

Figure S13. The molecular structure of **LH(BF_2)**[6] (ORTEP view 50% probability level). Hydrogen atoms, except for NH groups, are omitted for clarity. Selected bond lengths (Å) and angles (°): B1–N1 1.5350(16), B1–N4 1.5778(15), N1–B1–N4 107.72(9), B1–F1 1.3866(15), B1–F2 1.3817(15), C1–N1 1.3366(14), C1–N2 1.3440(15), C1–N3 1.3404(14), C2–N3 1.3260(15), C2–N4 1.3770(14), C2–N5 1.3557(14), N2–C1–N1 120.41(10), N3–C1–N1 124.54(10), N3–C1–N2 115.05(10), C2–N3–C1 120.70(10), N3–C2–N5 115.25(10), N3–C2–N4 123.04(10), N5–C2–N4 121.71(10), C5–N4 1.3710(14), C4–C5 1.3655(16), C3–C4 1.4010(16), C3–N5 1.3072(15), C3–O1 1.3404(14), C5–O2 1.3292(14), N1–N4 2.514(1), N2–H2···O3 142.4(14), N2–H2 0.871(16), H2···O3 2.130(16), N2···O3 2.8700(15).

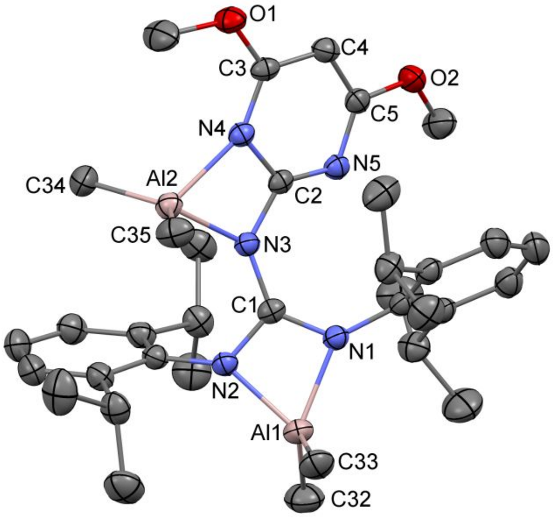

Figure S14. The molecular structure of **L($AlMe_2$)$_2$**[4,4] (ORTEP view 50% probability level). Hydrogen atoms are omitted for clarity. Selected bond lengths (Å) and angles (°): Al1–N1 1.9549(15), Al1–N2 1.9158(15), Al2–N3 1.9523(16), Al2–N4 1.9987(16), N1–Al1–N2 68.91(6), N3–Al2–N4 67.27(6), Al1–C32 1.952(2), Al1–C33 1.965(2), Al2–C34 1.943(2), Al2–C35 1.953(2), C1–N1 1.337(2), C1–N2 1.355(2), C1–N3 1.368(2), C2–N3 1.367(2), C2–N4 1.377(2), C2–N5 1.323(2), N2–C1–N1 108.91(14), N3–C1–N1 132.41(16), N3–C1–N2 118.43(15), C2–N3–C1 131.05(15), N3–C2–

N5 128.54(15), N3–C2–N4 105.80(14), N5–C2–N4 125.53(15), C3–N4 1.335(2), C3–C4 1.374(3), C4–C5 1.385(3), C5–N5 1.336(2), C3–O1 1.336(2), C5–O2 1.340(2), N1–N5 3.360(2).

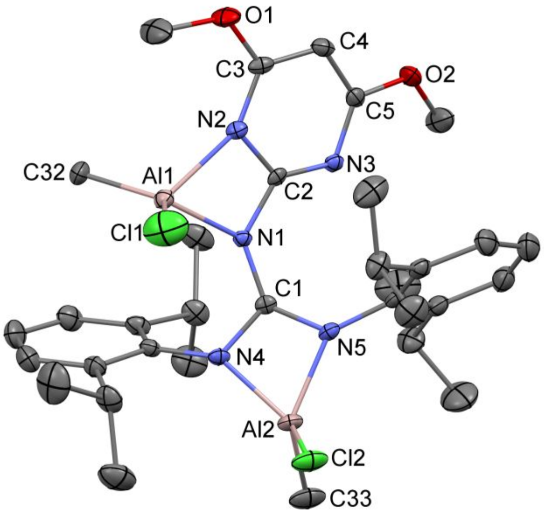

Figure S15. The molecular structure of **L(AlMeCl)$_2$**^{4,4} (ORTEP view 50% probability level). Hydrogen atoms are omitted for clarity. Selected bond lengths (Å) and angles (°): Al1–N1 1.943(3), Al1–N2 1.980(3), Al2–N4 1.887(3), Al2–N5 1.927(3), N1–Al1–N2 68.01(10), N4–Al2–N5 70.25(11), Al1–C32 1.951(3), Al1–Cl1 2.0755(16), Al2–C33 1.970(9), Al2–Cl2 2.097(2), C1–N4 1.363(4), C1–N5 1.337(4), C1–N1 1.366(4), C2–N1 1.376(4), C2–N2 1.382(4), C2–N3 1.321(4), N4–C1–N1 118.6(2), N5–C1–N1 132.4(3), N4–C1–N5 108.7(2), C1–N1–C2 131.0(2), N1–C2–N3 128.3(3), N1–C2–N2 105.4(2), N2–C2–N3 126.1(3), C3–N2 1.345(4), C3–C4 1.373(4), C4–C5 1.394(4), C5–N3 1.348(4), C3–O1 1.332(4), C5–O2 1.328(4), N3–N5 3.356(3).

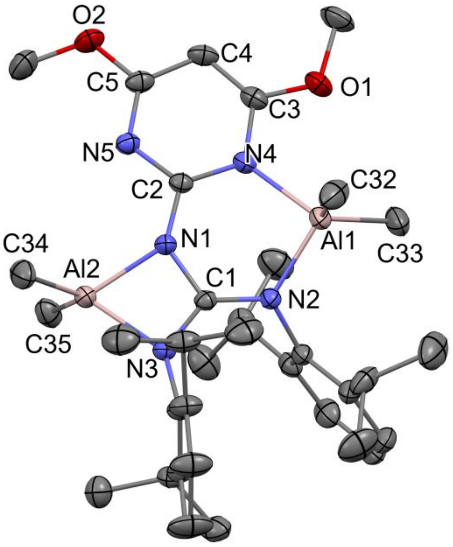

Figure S16. The molecular structure of **L(AlMe$_2$)$_2$**$^{6,4\ 4}$ (ORTEP view 50% probability level). Hydrogen atoms are omitted for clarity. Selected bond lengths (Å) and angles (°): Al1–N2 1.9197(16), Al1–N4 1.9762(16), Al2–N1 1.9310(15), Al2–N3 1.9614(15), N2–Al1–N4 92.74(6), N1–Al2–N3 67.67(6), Al1–C32 1.957(2), Al1–C33 1.966(2), Al2–C34 1.943(2), Al2–C35 1.956(2), C1–N1 1.387(2), C1–N2 1.333(2), C1–N3 1.341(2), C2–N1 1.344(2), C2–N4 1.358(2), C2–N5 1.348(2), N2–C1–N1 122.72(15), N3–C1–N1 105.25(14), N3–C1–N2 131.89(16), C2–N1–

C1 129.40(15), N1–C2–N4 121.99(16), N1–C2–N5 113.75(15), N5–C2–N4 124.21(16), C3–N4 1.362(2), C3–C4 1.365(3), C4–C5 1.399(3), C5–N5 1.318(2), C3–O1 1.334(2), C5–O2 1.333(2), N2–N4 2.820(2).

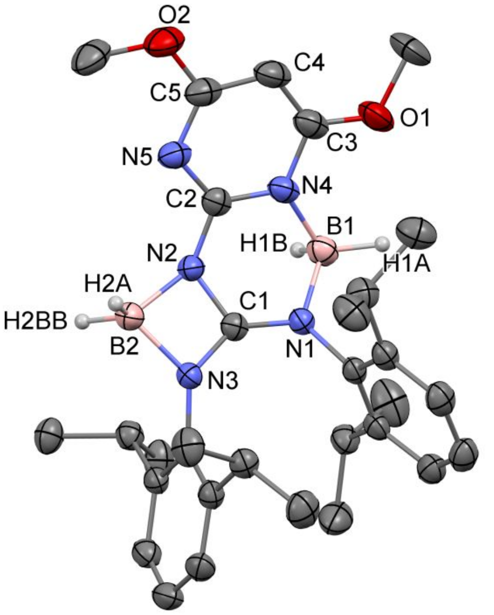

Figure S17. The molecular structure of **$L(BH_2)_2^{6,4}$** (ORTEP view 50% probability level). Hydrogen atoms, except for BH_2 groups, are omitted for clarity. Selected bond lengths (Å) and angles (°): B1–N1 1.569(3), B1–N4 1.603(4), B2–N2 1.600(3), B2–N3 1.605(3), N1–B1–N4 104.96(18), N2–B2–N3 80.96(16), C1–N1 1.313(3), C1–N2 1.380(3), C1–N3 1.330(2), C2–N2 1.355(3), C2–N4 1.356(3), C2–N5 1.330(3), N2–C1–N1 120.91(18), N3–C1–N1 138.6(2), N3–C1–N2 100.35(18), C2–N2–C1 122.19(17), N2–C2–N4 116.2(2), N2–C2–N5 118.20(19), N5–C2–N4 125.6(2), C3–N4 1.368(3), C3–C4 1.363(4), C4–C5 1.387(3), C5–N5 1.328(3), C3–O1 1.332(3), C5–O2 1.337(3), N1–N4 2.515(2).

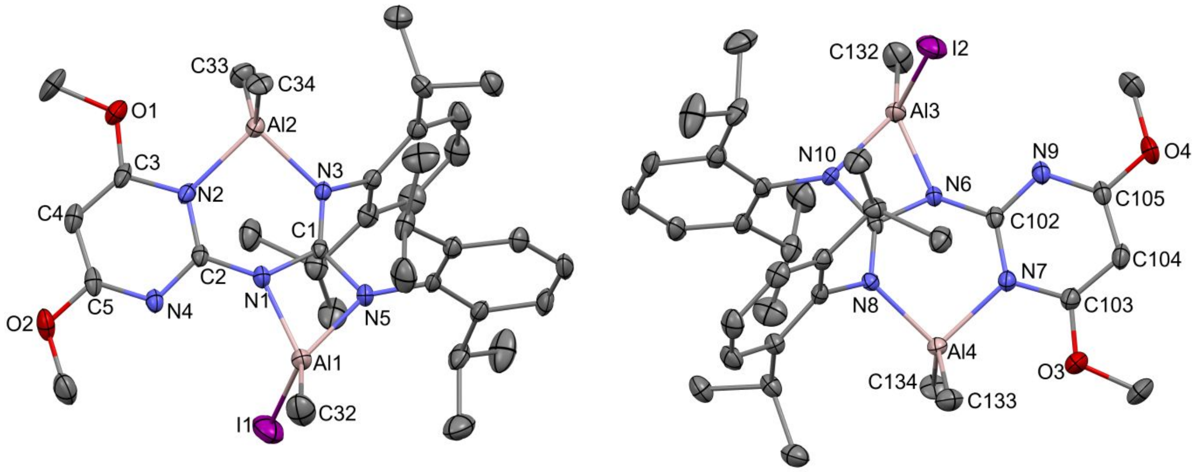

Figure S18. The molecular structure (two independent molecules) of **$L(AlMe_2)^6(AlMeI)^4$** (ORTEP view 50% probability level). Hydrogen atoms are omitted for clarity. Selected bond lengths (Å) and angles (°): Two independent molecules present. Selected bond lengths (Å) and angles (°): Al1–N1 1.900(4), Al1–N5 1.920(3), Al2–N2 1.972(3), Al2–N3 1.937(3), N1–Al1–N5 69.23(14), N2–Al2–N3 93.46(14), Al1–C32 1.982(5), Al1–I1 2.5453(13), Al2–C33 1.956(4), Al2–C34 1.962(4), C1–N1 1.400(4), C1–N3 1.327(5), C1–N5 1.347(5), C2–N1 1.349(5), C2–N2 1.347(5), C2–N4 1.346(5), N3–C1–N1 123.4(3), N5–C1–N1 104.4(3), N3–C1–N5 132.2(3), C2–N1–C1 129.0(3), N1–

C2–N2 121.6(3), N1–C2–N4 113.7(3), N2–C2–N4 124.6(3), C3–N2 1.373(5), C3–C4 1.361(6), C4–C5 1.391(6), C5–N4 1.321(5), C3–O1 1.329(5), C5–O2 1.338(5), N2–N3 2.847(5).

Al3–N6 1.905(3), Al3–N10 1.920(3), Al4–N7 1.976(3), Al4–N8 1.935(3), N6–Al3–N10 69.24(13), N7–Al4–N8 93.66(14), Al3–C132 1.970(5), Al3–I2 2.5547(13), Al4–C133 1.952(4), Al4–C134 1.957(4), C101–N6 1.396(4), C101–N8 1.327(5), C101–N10 1.350(5), C102–N6 1.342(5), C102–N7 1.358(5), C102–N9 1.339(5), N6–C101–N8 123.3(3), N8–C101–N10 132.0(3), N6–C101–N10 104.6(3), C102–N6–C101 129.9(3), N6–C102–N7 120.9(3), N6–C102–N9 114.5(3), N7–C102–N9 124.5(3), C103–N7 1.355(5), C103–C104 1.367(6), C104–C105 1.388(6), C105–N9 1.323(5), C103–O3 1.339(5), C105–O4 1.338(5), N7–N8 2.853(5).

POLYMERIZATION

Polymerization – representative ^{1}H NMR Spectra

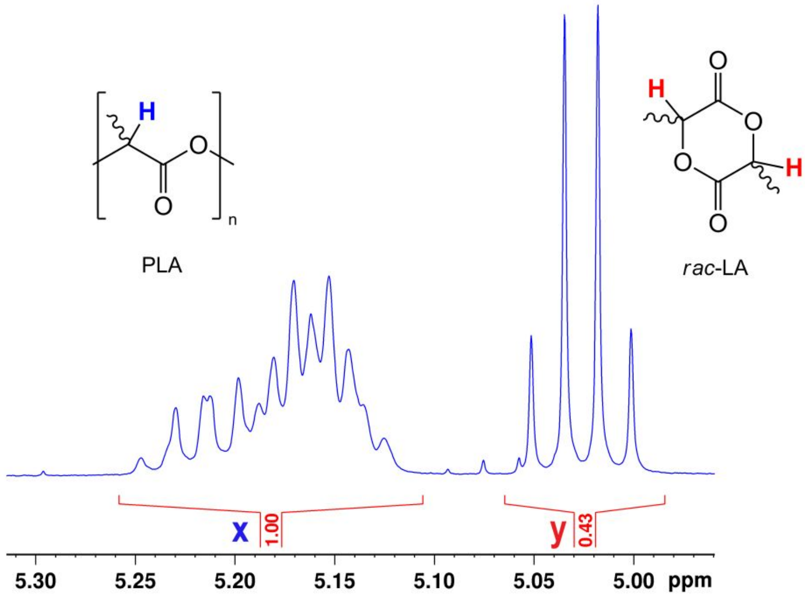

Figure S19. ^{1}H NMR spectrum in $CDCl_3$ of the methine region of crude PLA produced using **L(AlMe$_2$)$_2$**^{4,4} in toluene at 90 °C ([*rac*-LA:**L(AlMe$_2$)$_2$**^{4,4}:^{i}PrOH] = 100:1:1, 8 h) aiming to determine PLA conversion = [x/(x+y)] × 100% (Table 1, entry 4).

Representative GPC Spectra

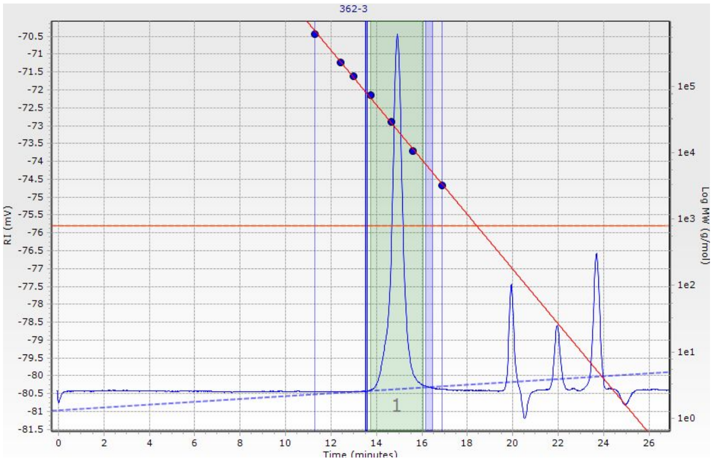

Figure S20. GPC spectrum of crude PLA produced using **LH(AlMe$_2$)**^{6} in toluene at 90°C ([*rac*-LA:cat.:^{i}PrOH] = 100:1:1, 8 h) (Table 1, entry 2).

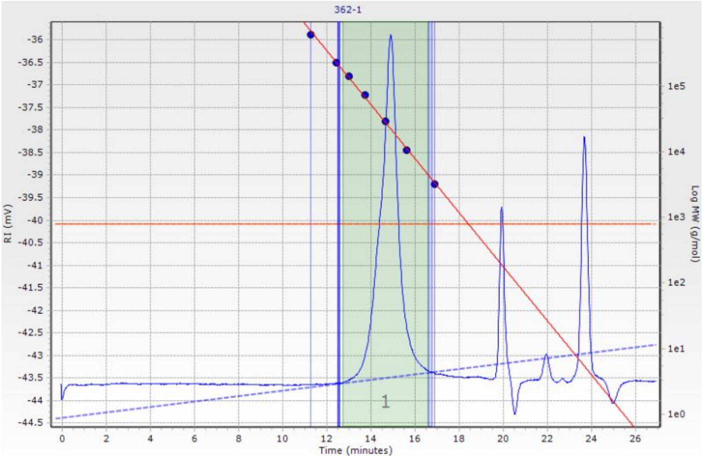

Figure S21. GPC spectrum of crude PLA produced using **LH(AlMe$_2$)**[6] in toluene at 90°C ([*rac*-LA: cat.:^{i}PrOH] = 100:1:1, 24 h) (Table 1, entry 3).

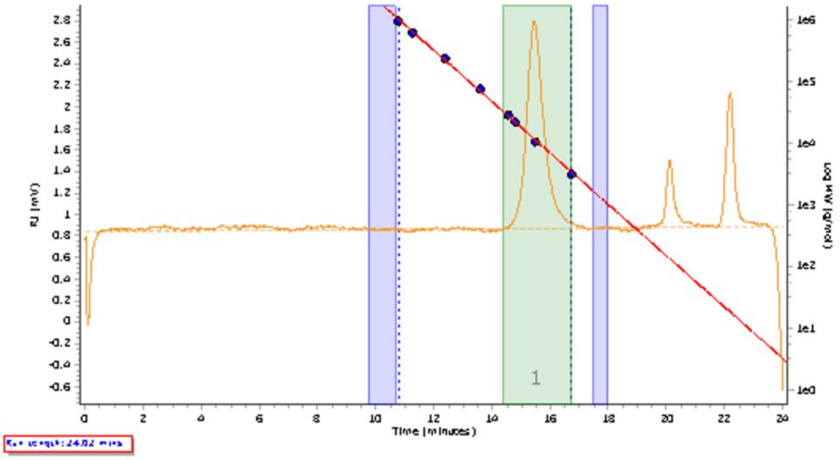

Figure S22. GPC spectrum of crude PLA produced using **L(AlMe$_2$)$_2$**[4,4] in toluene at 90°C ([*rac*-LA: cat.:^{i}PrOH] = 100:1:1, 8 h) (Table 1, entry 4).

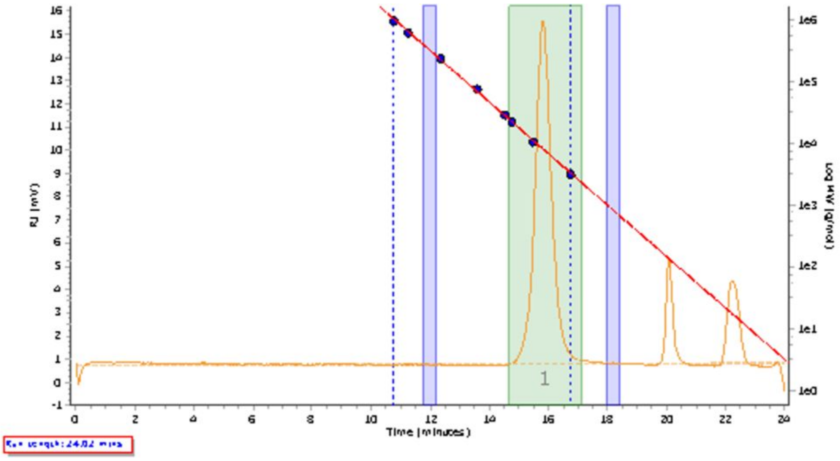

Figure S23. GPC spectrum of crude PLA produced using **L(AlMe$_2$)$_2$**[4,4] in toluene at 90°C ([*rac*-LA: cat.:^{i}PrOH] = 100:1:2, 8 h) (Table 1, entry 5).

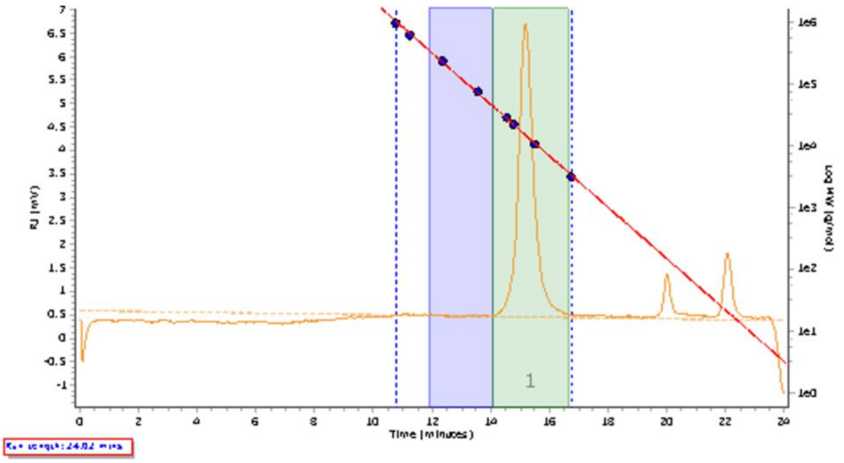

Figure S24. GPC spectrum of crude PLA produced using **L(AlMe$_2$)$_2$**^{4,4} in toluene at 90°C ([*rac*-LA: cat.:^{i}PrOH] = 200:1:2, 8 h) (Table 1, entry 6).

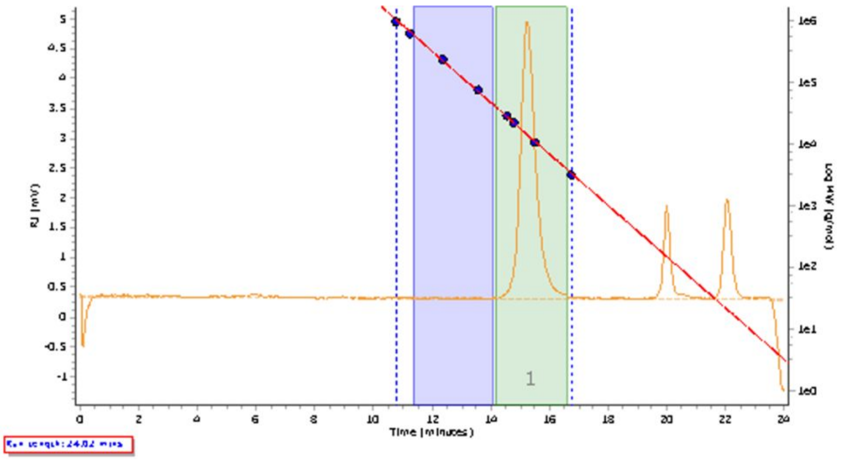

Figure S25. GPC spectrum of crude PLA produced using **L(AlMe$_2$)$_2$**^{6,4} in toluene at 90°C ([*rac*-LA: cat.:^{i}PrOH] = 100:1:1, 8 h) (Table 1, entry 8).

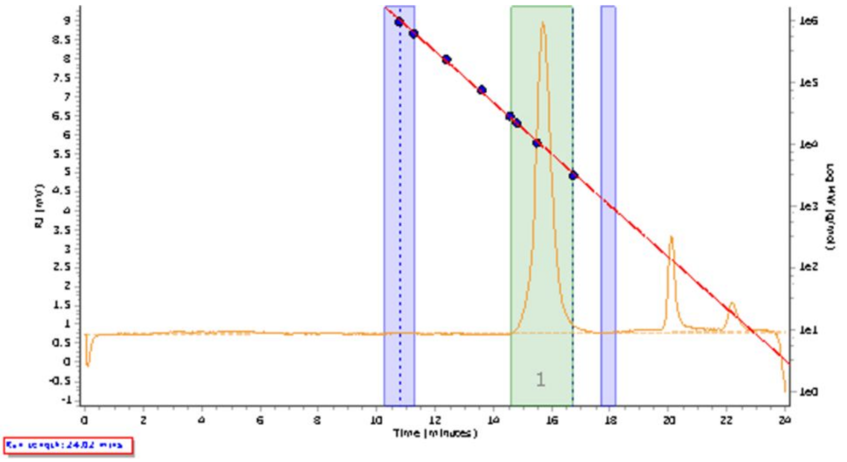

Figure S26. GPC spectrum of crude PLA produced using **L(AlMe$_2$)$_2$**^{6,4} in toluene at 90°C ([*rac*-LA: cat.:^{i}PrOH] = 100:1:2, 8 h) (Table 1, entry 9).

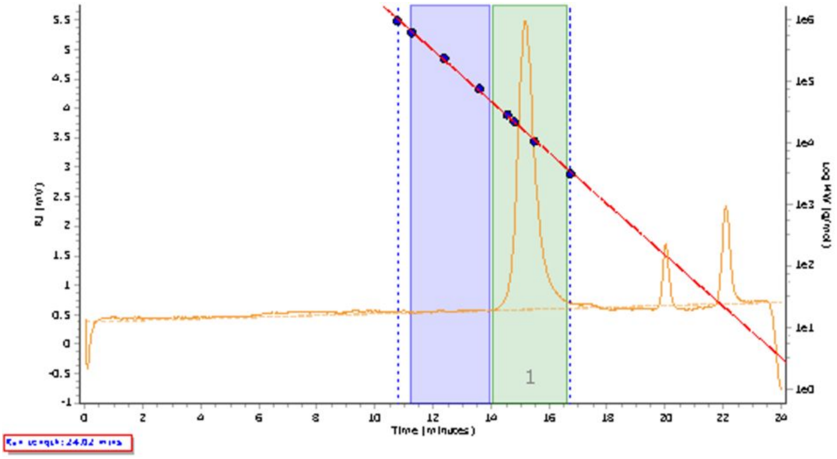

Figure S27. GPC spectrum of crude PLA produced using **L(AlMe$_2$)$_2$**^{6,4} in toluene at 90°C ([*rac*-LA: cat.:^{i}PrOH] = 200:1:2, 8 h) (Table 1, entry 10).

MALDI-TOF Spectra of PLA samples

These analyses were performed to determine the linear or cyclic nature of the PLA chains as well as the end-chain group. The presence or absence of peaks between the majority peaks can be used to assess the degree of transesterification.

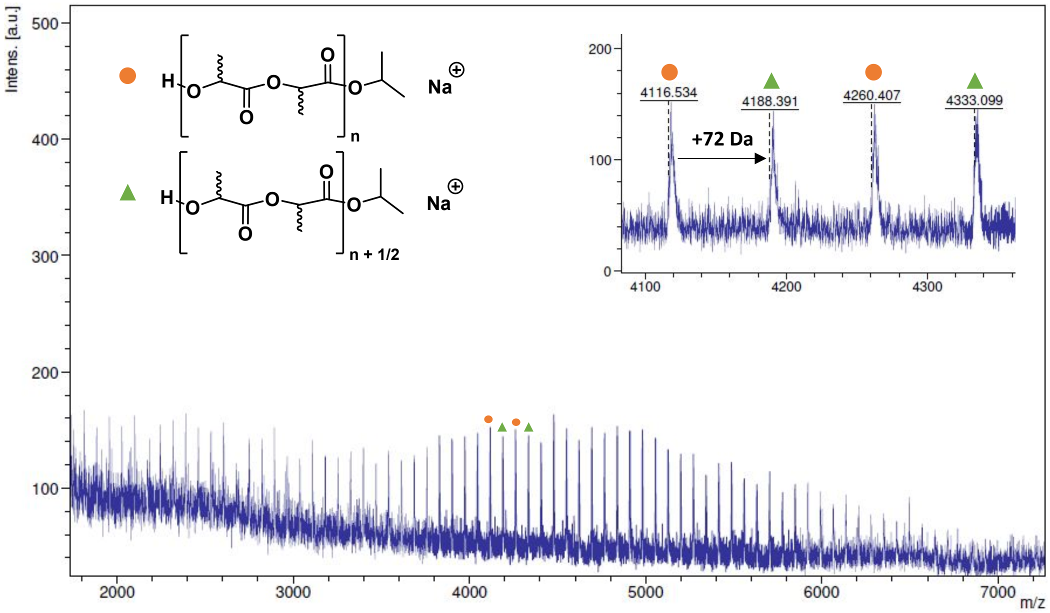

Figure S28. MALDI-TOF spectrum of PLA produced using **LH(AlMe$_2$)**[6] with ^{i}PrOH as co-initiator (90°C, 8 h, 25:1:1). Magnified versions are provided to assist in identifying the repeat unit.

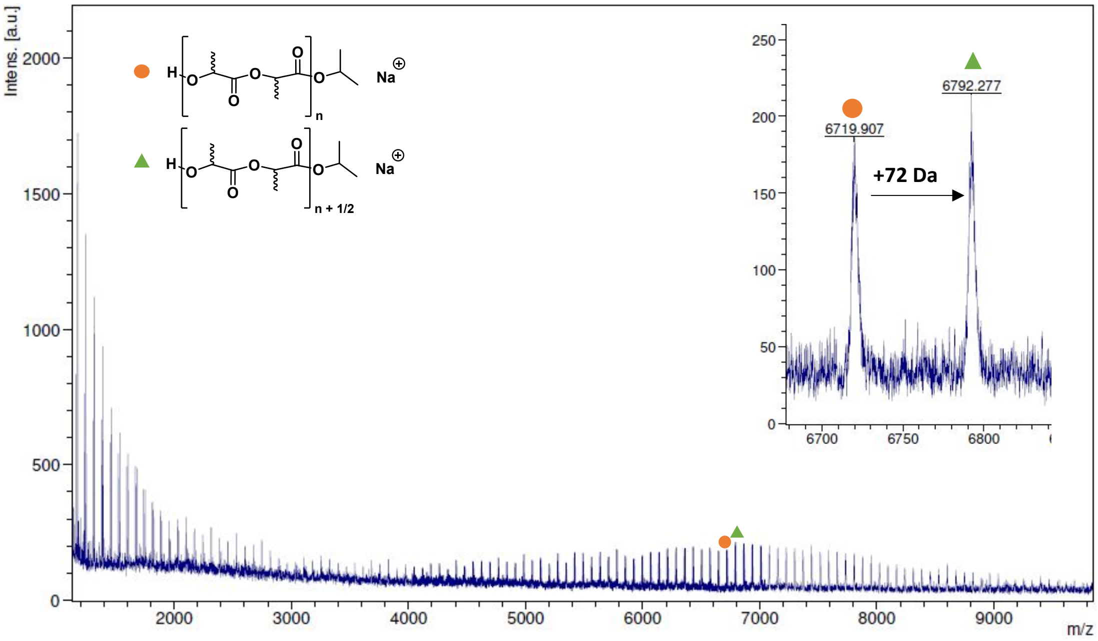

Figure S29. MALDI-TOF spectrum of PLA produced using **L(AlMe$_2$)$_2$**[6,4] with ^{i}PrOH as co-initiator (90°C, 8 h, 50:1:1). Magnified versions are provided to assist in identifying the repeat unit.

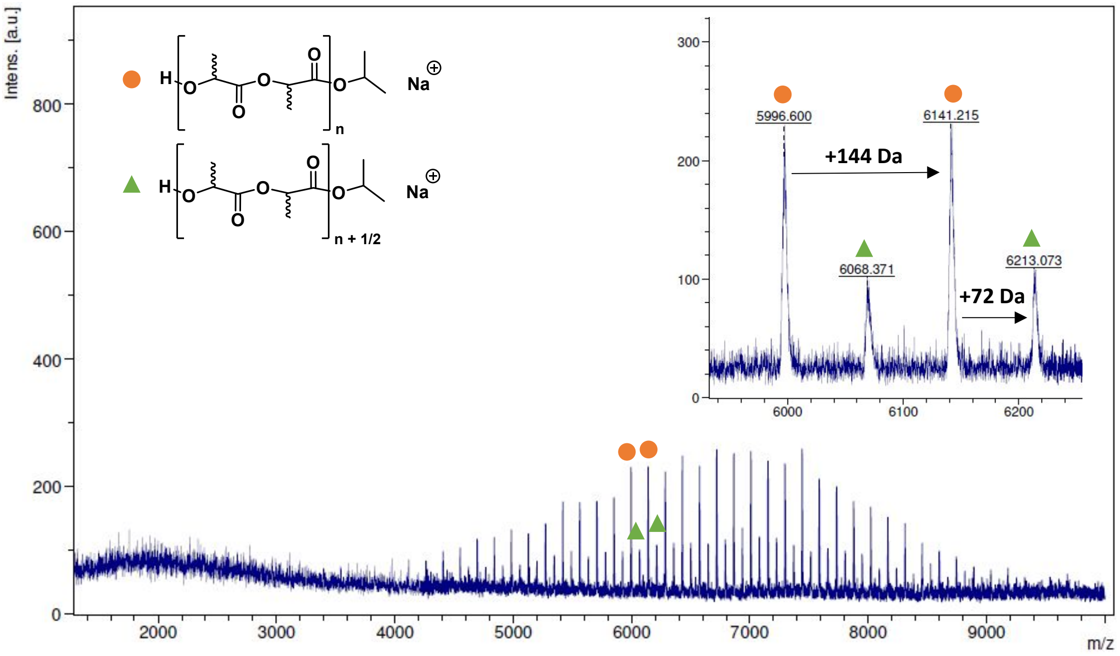

Figure S30. MALDI-TOF spectrum of PLA produced using **L(AlMe$_2$)$_2$**^{6,4} with ^{i}PrOH as co-initiator (90°C, 8 h, 100:1:2). Magnified versions are provided to assist in identifying the repeat unit.

HYDROBORATION

General procedure for tests of catalytic activity of complexes LH(AlMe$_2$)[6], LH(BH$_2$)[6], L(BH$_2$)$_2$[6,4] and LH(BH$_2$)[6] in hydroboration reactions

A crimp capped vial (in case of neat reaction) or Schlenk rotaflo vessel (in case of reaction with solvent) was charged with the catalyst, substrate (phenylacetylene, styrene or chalcone, 0.125 mmol) and pinacolborane (1.2 equiv., 0.15 mmol, 21.8 µL) in a glovebox. The reactions were carried out in the conditions listed in Table S1. In case of reaction with solvent 0.2 mL of toluene was added. After the reaction, crude reaction mixture was analysed by GC and GC–MS analyses. Products were purified using column chromatography.

Table S1. Hydroboration of phenylacetylene and styrene in the presence of **LH(AlMe$_2$)**[6], **LH(BH$_2$)**[6], **LH(BF$_2$)**[6], **L(BH$_2$)$_2$**[6,4] and **L(BH$_2$)$_2$**[4,4].

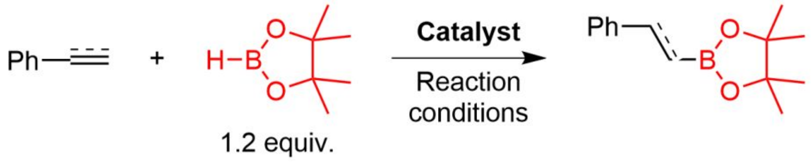

Entry	Substrate	Catalyst	Catalyst loading	Reaction conditions	Solvent	Yield*
1	Phenylacetylene	**LH(BH$_2$)**[6]	3 mol%	20 h, RT	neat	33
2	Phenylacetylene	**L(BH$_2$)$_2$**[6,4]	3 mol%	20 h, RT	neat	30
3	Phenylacetylene	**L(BH$_2$)$_2$**[4,4]	3 mol%	20 h, RT	neat	30
4	Phenylacetylene	**LH(BF$_2$)**[6]	3 mol%	20 h, RT	neat	22
5	Phenylacetylene	**LH(AlMe$_2$)**[6]	3 mol%	20 h, RT	neat	23
6	Phenylacetylene	**LH(AlMe$_2$)**[6]	3 mol%	17 h, 60 °C	neat	38
7	Phenylacetylene	**LH(AlMe$_2$)**[6]	3 mol%	17 h, 100 °C	neat	58
8	Phenylacetylene	**LH(AlMe$_2$)**[6]	10 mol%	24 h, 100 °C	toluene	87
9	Styrene	**LH(BH$_2$)**[6]	3 mol%	20 h, RT	neat	4.5
10	Styrene	**L(BH$_2$)$_2$**[6,4]	3 mol%	20 h, RT	neat	4.5
11	Styrene	**LH(BF$_2$)**[6]	3 mol%	20 h, RT	neat	2.8
12	Styrene	**LH(AlMe$_2$)**[6]	3 mol%	20 h, RT	neat	3.2
13	Styrene	**LH(AlMe$_2$)**[6]	3 mol%	17 h, 60 °C	neat	22
14	Styrene	**LH(AlMe$_2$)**[6]	3 mol%	17 h, 100 °C	neat	45
15	Styrene	**LH(AlMe$_2$)**[6]	10 mol%	24 h, 100 °C	toluene	55

* based on GC and GC-MS analyses

Characterization of synthesized products

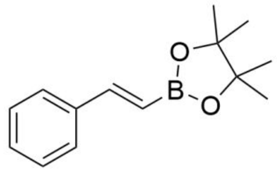

(E)-4,4,5,5-tetramethyl-2-styryl-1,3,2-dioxaborolane

^{1}H NMR (300 MHz, $CDCl_3$, δ, ppm): 7.52 – 7.46 (m, 2H), 7.40 (d, $J_{H\text{-}H}$ = 18.3 Hz, 1H), 7.36 – 7.28 (m, 3H), 6.17 (d, $J_{H\text{-}H}$ = 18.3 Hz, 1H), 1.32 (s, 12H). **^{13}C NMR** (75 MHz, $CDCl_3$, δ, ppm): 149.66, 137.64, 129.04, 128.71, 127.21, 83.51, 24.96. C_α to boron atom was not observed. **MS (EI) [m/z (%)]**:230 (M^+, 44), 215 (20), 144(78), 129 (100), 118(16), 105(31), 85(12), 77(26). Isolated yield: 77% (22 mg). The analytical data are in agreement with the literature.[1]

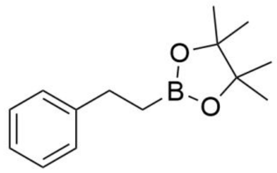

4,4,5,5-tetramethyl-2-phenethyl-1,3,2-dioxaborolane

^{1}H NMR (400 MHz, $CDCl_3$, δ, ppm): 7.29 – 7.20 (m, 4H), 7.19 – 7.11 (m, 1H), 2.75 (t, $J_{H\text{-}H}$ = 8.0 Hz, 2H), 1.22 (s, 12H), 1.15 (t, $J_{H\text{-}H}$ = 8.0 Hz, 2H). **^{13}C NMR** (75 MHz, $CDCl_3$, δ, ppm): 144.54, 128.30, 128.13, 125.62, 83.21, 30.08, 24.93. C_α to boron atom was not observed. **MS (EI) [m/z (%)]**: 232 (M^+, 6), 217 (6), 175(26), 132 (43), 105(29), 91(50), 84(100), 69 (13). Isolated yield: 48% (14 mg). The analytical data are in agreement with the literature.[1]

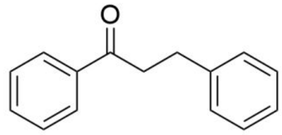

1,3-diphenylpropan-1-one

MS (EI) [m/z (%)]:210(M^+, 32), 105(100), 92(10), 78 (44), 66(4), 51(21).

1. Wu, Y.; Shan, C.; Ying, J.; Su, J.; Zhu, J.; Liu, L. L; Zhao, Y. Catalytic hydroboration of aldehydes, ketones, alkynes and alkenes initiated by NaOH. *Green Chem.*, **2017**, *19*, 4169-4175. doi.org/10.1039/C7GC01632H

NMR spectra of synthesized products

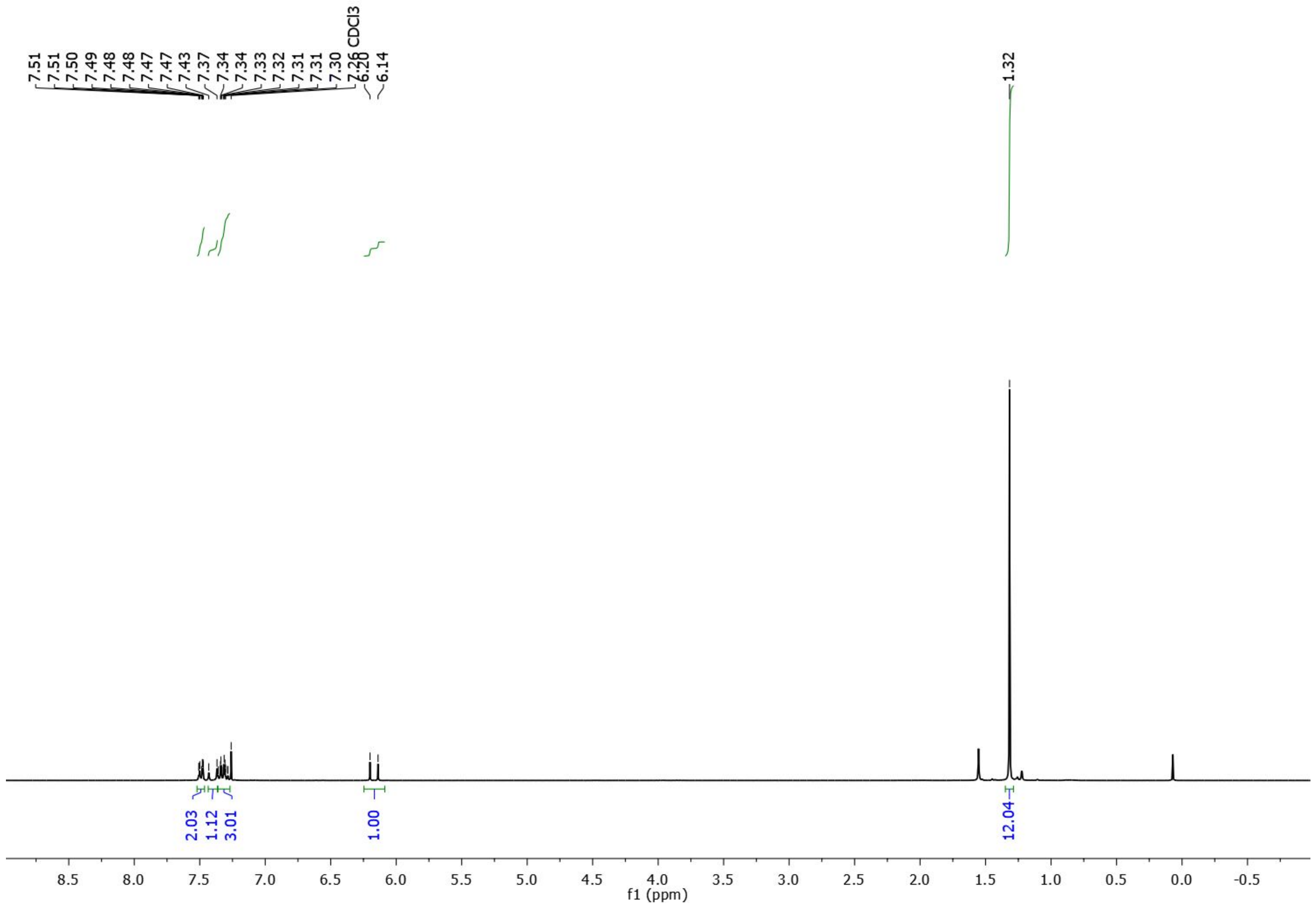

Figure S31. ^{1}H NMR spectrum of (E)-4,4,5,5-tetramethyl-2-styryl-1,3,2-dioxaborolane in $CDCl_3$, 295K.

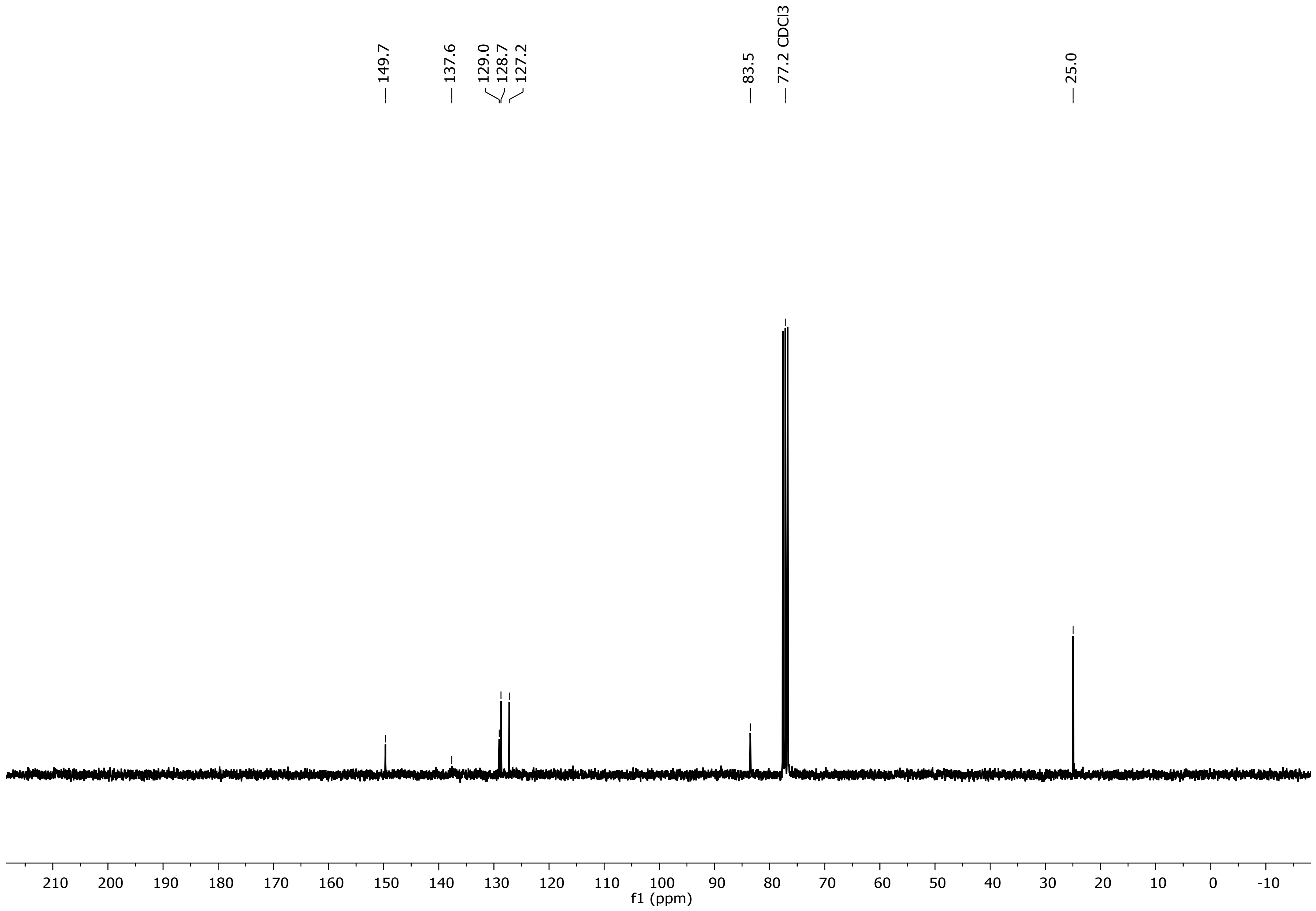

Figure S32. ^{13}C NMR spectrum of (E)-4,4,5,5-tetramethyl-2-styryl-1,3,2-dioxaborolane in $CDCl_3$, 295 K.

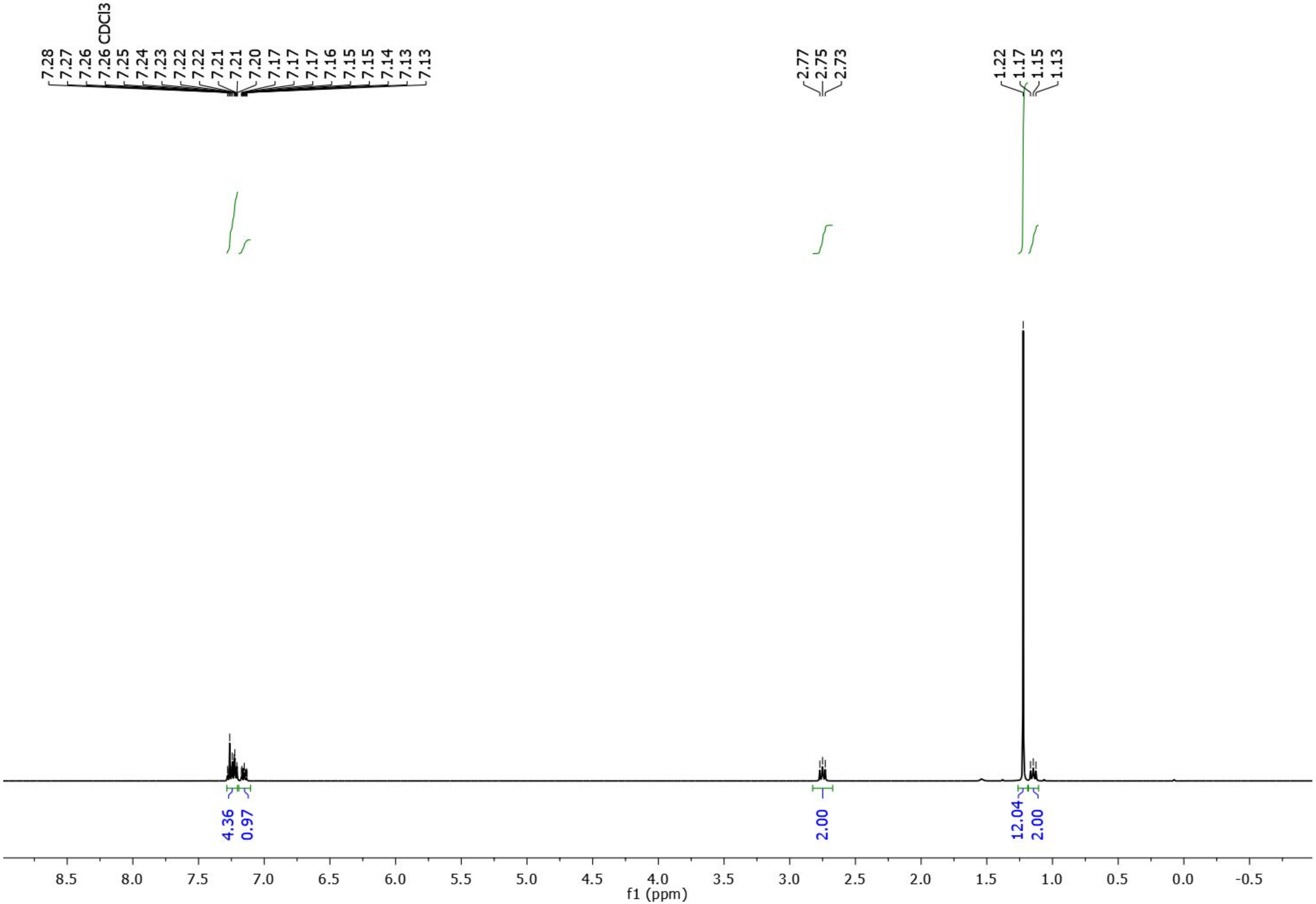

Figure S33 ^{1}H NMR spectrum of 4,4,5,5-tetramethyl-2-phenethyl-1,3,2-dioxaborolane in $CDCl_3$, 295 K.

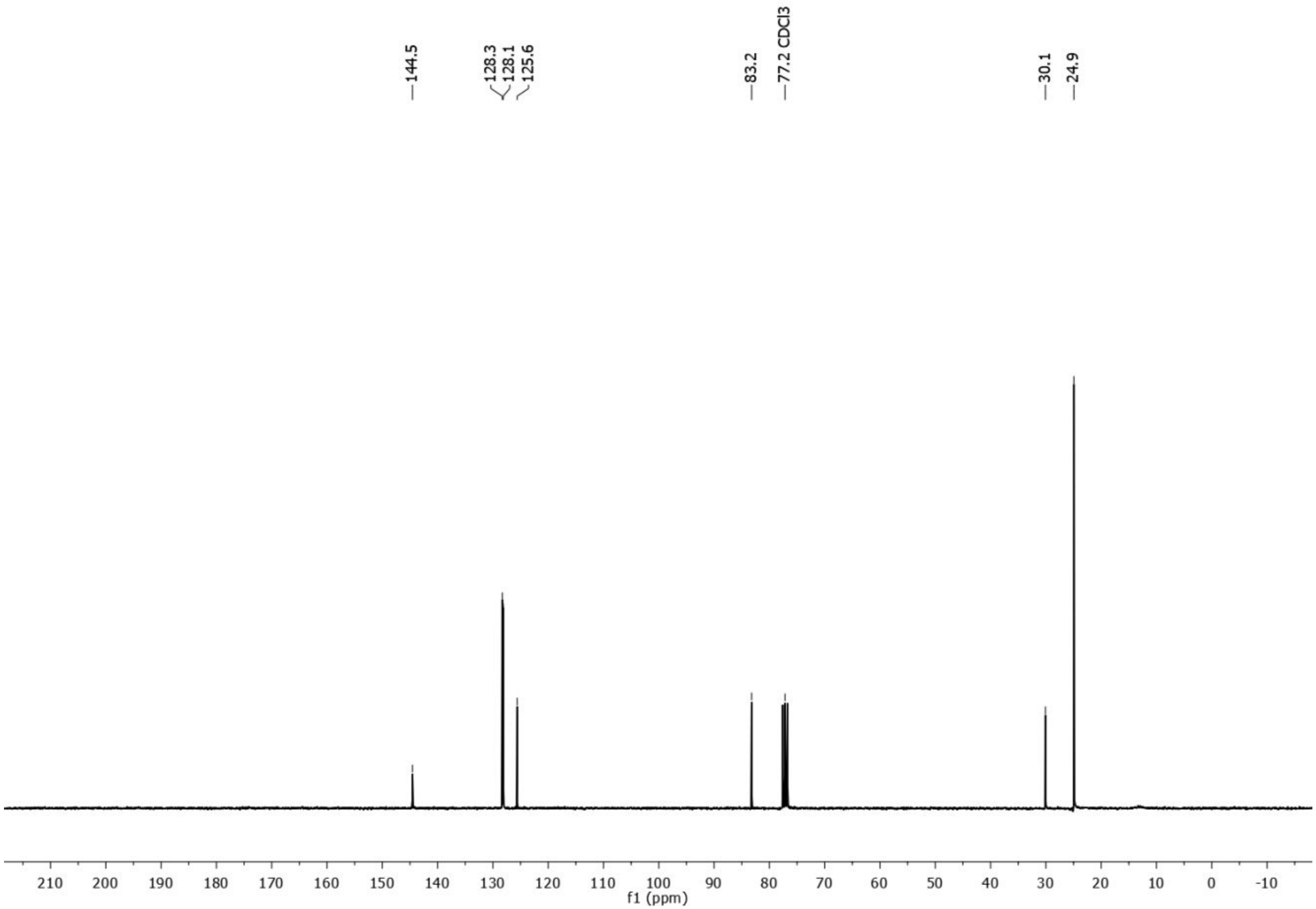

Figure S34. ^{13}C NMR spectrum of 4,4,5,5-tetramethyl-2-phenethyl-1,3,2-dioxaborolane in $CDCl_3$, 295 K.

SYNTHESIS

Preparation and Characterization of **LH(Li)**[4]

To a colorless solution of **LH₂** (1.000 g; 1.93 mmol) in Et_2O (40 mL) cooled to -80 C, a 1.6 M solution of ^{n}BuLi in hexane (1.21 mL; 1.93 mmol) was added. The reaction mixture was allowed to warm to room temperature and stirred for 24 hours. The volatiles were evaporated under vacuum with the yield of 1.001 g (>99 %) of white **LH(Li)**[4]. Single crystals suitable for *sc*XRD analyses were obtained by cooling of a saturated solution of **LH(Li)**[4] in hexane to 7 °C. ^{1}H NMR (THF-d_8, 500.20 MHz, 295 K) δ: 9.16 (s, 1H, NH^{Dipp}); 7.09 (s, 3H, ArH^{Dipp}); 6.87 (d, 2H, ^{3}J = 7.6 Hz, ArH^{Dipp}); 6.67 (t, 1H, $^{3}J_{H,H}$ = 7.5 Hz, ArH^{Dipp}); 5.13 (s, 1H, ArH^{Prm}); 3.78 (br s, 3H, O-CH_3); 3.77 (m, 2H, $^{3}J_{H,H}$ = 6.8 Hz, CH^{Dipp}); 3.64 (br s, 3H, O-CH_3); 3.23 (m, 2H, $^{3}J_{H,H}$ = 6.8 Hz, CH^{Dipp}); 1.30 (br s, 6H, CH_3^{Dipp}); 1.19 (br s, 6H, CH_3^{Dipp}); 1.13 (d, 6H, $^{3}J_{H,H}$ = 6.9 Hz, CH_3^{Dipp}); 0.95 (d, 6H, $^{3}J_{H,H}$ = 6.8 Hz, CH_3^{Dipp}). ^{13}C NMR (THF-d_8, 100.61 MHz, 295 K) δ: 172.5 (br s, 2x ArC_q^{OMe}); 165.8 (ArC_q^{Prm}); 153.6 (ArC_q^{Gua}); 149.9 (ArC_q^{Dipp}); 148.3 (ArC_q^{Dipp}); 140.9 (ArC_q^{Dipp}); 138.6 (ArC_q^{Dipp}); 126.7 (ArCH^{Dipp}); 123.0 (ArCH^{Dipp}); 122.5 (ArCH^{Dipp}); 120.1 (ArCH^{Dipp}); 75.4 (ArCH^{Prm}); 53.5 (br s, 2x O-CH_3); 29.4 (CH^{Dipp}); 28.8 (CH^{Dipp}); 26.3 (br s, CH_3^{Dipp}); 24.6 (CH_3^{Dipp}); 24.2 (CH_3^{Dipp}); 23.5 (br s, CH_3^{Dipp}). ^{7}Li NMR (THF-d_8, 194.40 MHz, 295 K) δ: 0.2.

Preparation and Characterization of **LH(AlMe₂)**[6]

To a colorless solution of **LH₂** (0.867 g; 1.67 mmol) in Et_2O (20 mL) cooled to -80 C, a 2.0 M solution of Me_3Al in hexane (0.84 mL; 1.67 mmol) was added. The reaction mixture was allowed to warm to room temperature and stirred for 24 hours with gradual precipitation of a white solid. The precipitate was separated by filtration and dried under vacuum with the yield of 0.721 g (75 %) of white crystalline **LH(AlMe₂)**[6]. Single crystals suitable for *sc*XRD analyses were obtained by cooling of a saturated solution of **LH(AlMe₂)**[6] in Et_2O to 7 °C. ^{1}H NMR (C_6D_6, 500.13 MHz, 295 K) δ: 7.18 (s, 3H, ArH^{Dipp}); 7.14–7.10 (m, 1H, ArH^{Dipp}); 7.08–7.05 (m, 2H, ArH^{Dipp}); 5.63 (s, 1H, NH^{Dipp}); 4.89 (s, 1H, ArH^{Prm}); 3.68 (m, 2H, $^{3}J_{H,H}$ = 6.8 Hz, CH^{Dipp}); 3.39 (s, 3H, O-CH_3); 3.30 (m, 2H, $^{3}J_{H,H}$ = 6.8 Hz, CH^{Dipp}); 2.80 (s, 3H, O-CH_3); 1.50 (br s, 6H, CH_3^{Dipp}); 1.39 (d, 6H, $^{3}J_{H,H}$ = 6.7 Hz, CH_3^{Dipp}); 1.28 (d, 6H, $^{3}J_{H,H}$ = 6.9 Hz, CH_3^{Dipp}); 1.08 (br s, 6H, CH_3^{Dipp}); -0.26 (s, 6H, AlCH_3). ^{13}C NMR (C_6D_6, 125.78 MHz, 295 K) δ: 173.7 (ArC_q^{OMe}); 169.5 (ArC_q^{OMe}); 161.7 (ArC_q^{Prm}); 161.1 (ArC_q^{Gua}); 146.8 (ArC_q^{Dipp}); 146.5 (ArC_q^{Dipp}); 138.9 (ArC_q^{Dipp}); 133.8 (ArC_q^{Dipp}); 128.7 (ArCH^{Dipp}); 127.9 (ArCH^{Dipp}); 125.4 (ArCH^{Dipp}); 123.9 (ArCH^{Dipp}); 78.3 (ArCH^{Prm}); 55.3 (O-CH_3); 54.3 (O-CH_3); 29.4 (CH^{Dipp}); 28.9 (CH^{Dipp}); 26.1 (CH^{Dipp}); 25.0 (CH^{Dipp}); 24.5 (2x CH_3^{Dipp}); -7.9 (AlCH_3). ^{1}H NMR (THF-d_8, 500.13 MHz, 295 K) δ: 7.26–7.19 (m, 3H, ArH^{Dipp}); 7.17–7.12 (m, 1H, ArH^{Dipp}); 7.08–7.05 (m, 2H, ArH^{Dipp}); 6.18 (s, 1H, NH^{Dipp}); 5.66 (s, 1H, ArH^{Prm}); 3.86 (s, 3H, O-CH_3); 3.77 (s, 3H, O-CH_3); 3.39 (m, 2H, $^{3}J_{H,H}$ = 6.8 Hz, CH^{Dipp}); 3.21 (m, 2H, $^{3}J_{H,H}$ = 6.8 Hz, CH^{Dipp}); 1.32 (d, 6H, $^{3}J_{H,H}$ = 6.9 Hz, CH_3^{Dipp}); 1.26 (br s, 6H, CH_3^{Dipp}); 1.20 (d, 6H, $^{3}J_{H,H}$ = 6.7 Hz, CH_3^{Dipp}); 1.08 (br s, 6H, CH_3^{Dipp}); -0.90 (s, 6H, AlCH_3). ^{13}C NMR (THF-d_8, 125.76 MHz, 295 K) δ: 174.2 (ArC_q^{OMe}); 170.4 (ArC_q^{OMe}); 161.8 (ArC_q^{Prm}); 161.6 (ArC_q^{Gua}); 147.4 (ArC_q^{Dipp}); 146.8 (ArC_q^{Dipp}); 139.6 (ArC_q^{Dipp}); 135.2 (ArC_q^{Dipp}); 128.0 (ArCH^{Dipp}); 127.6 (ArCH^{Dipp}); 125.3 (ArCH^{Dipp}); 123.6 (ArCH^{Dipp}); 78.3 (ArCH^{Prm}); 56.5 (O-CH_3); 54.3 (O-CH_3); 29.4 (CH^{Dipp}); 29.1 (CH^{Dipp}); 26.0 (CH^{Dipp}); 24.9 (CH^{Dipp}); 24.8 (CH_3^{Dipp}); 24.2 (CH_3^{Dipp}); -8.4 (AlCH_3). ^{1}H NMR (Tol-d_8, 500.13 MHz, 295 K) δ: 7.17–7.14 (m, 3H, ArH^{Dipp}); 7.12–7.07 (m, 1H, ArH^{Dipp}); 7.06–7.02 (m, 2H, ArH^{Dipp}); 5.59 (s, 1H, NH^{Dipp}); 4.90 (s, 1H, ArH^{Prm}); 3.61 (m, 2H, $^{3}J_{H,H}$ = 6.8 Hz, CH^{Dipp}); 3.41 (s, 3H,

O-CH_3); 3.25 (m, 2H, $^3J_{H,H}$ = 6.8 Hz, CH^{Dipp}); 2.91 (s, 3H, O-CH_3); 1.43 (br s, 6H, CH_3^{Dipp}); 1.35 (d, 6H, $^3J_{H,H}$ = 6.7 Hz, CH_3^{Dipp}); 1.28 (d, 6H, ^{3}J = 6.9 Hz, CH_3^{Dipp}); 1.06 (br s, 6H, CH_3^{Dipp}); -0.40 (s, 6H, AlCH_3).

Preparation and Characterization of **LH(AlMeI)**[6]

To a colorless solution of **LH(AlMe₂)**[6] (1.498 g; 2.52 mmol) in toluene (40 mL) cooled to -40 C, a solution of I_2 (0.640 g; 2.52 mmol) in toluene (10 mL) was added. The reaction mixture was allowed to warm to room temperature and heated to 60 °C for 2 hours with gradual precipitation of a yellow solid. The mixture was separated by filtration and the filtrate evaporated under vacuum to a volume of 10 mL with a precipitation of an off-white solid. The solvent was filtered off and the solid was washed with Et_2O (15 mL) with the yield of 1.075 g (62 %) of white crystalline **LH(AlMeI)**[6]. Single crystals suitable for *sc*XRD analyses were obtained by cooling of a saturated solution of **LH(AlMeI)**[6] in toluene to -30 °C. ^{1}H NMR (C_6D_6, 500.20 MHz, 295 K) δ: 7.18–7.14 (m, 3H, ArH^{Dipp}); 7.13–7.09 (m, 2H, ArH^{Dipp}); 7.04–6.98 (m, 1H, ArH^{Dipp}); 5.87 (s, 1H, NH^{Dipp}); 4.85 (s, 1H, ArH^{Prm}); 4.34 (m, 1H, $^3J_{H,H}$ = 6.8 Hz, CH^{Dipp}); 3.64–3.53 (m, 1H, CH^{Dipp}); 3.35 (m, 1H, $^3J_{H,H}$ = 6.9 Hz, CH^{Dipp}); 3.33 (s, 3H, O-CH_3); 3.11–3.01 (m, 1H, CH^{Dipp}); 2.81 (s, 3H, O-CH_3); 1.73–1.63 (m, 3H, CH_3^{Dipp}); 1.55 (d, 3H, $^3J_{H,H}$ = 6.6 Hz, CH_3^{Dipp}); 1.37–1.30 (m, 3H, CH_3^{Dipp}); 1.29–1.22 (m, 12H, CH_3^{Dipp}); 0.91–0.84 (m, 3H, CH_3^{Dipp}); -0,01 (s, 3H, AlCH_3). ^{13}C NMR (C_6D_6, 125.78 MHz, 295 K) δ: 174.2 (ArC_q^{OMe}); 168.7 (ArC_q^{OMe}); 161.1 (ArC_q^{Gua}); 160.9 (ArC_q^{Prm}); 147.9 (ArC_q^{Dipp}); 147.7 (ArC_q^{Dipp}); 146.4 (ArC_q^{Dipp}); 145.8 (ArC_q^{Dipp}); 137.5 (ArC_q^{Dipp}); 133.0 (ArC_q^{Dipp}); 128.7 (2x ArCH^{Dipp}); 126.2 (ArCH^{Dipp}); 125.3 (ArCH^{Dipp}); 124.6 (ArCH^{Dipp}); 123.4 (ArCH^{Dipp}); 79.2 (ArCH^{Prm}); 55.7 (O-CH$_3$); 54.6 (O-CH$_3$); 29.8 (CH^{Dipp}); 29.4 (CH^{Dipp}); 29.1 (2x CH^{Dipp}); 28.2 (CH$_3^{Dipp}$); 25.5 (CH$_3^{Dipp}$); 25.4 (CH$_3^{Dipp}$); 25.1 (CH$_3^{Dipp}$); 24.8 (CH$_3^{Dipp}$); 24.3 (CH$_3^{Dipp}$); 24.2 (CH$_3^{Dipp}$); 23.8 (CH$_3^{Dipp}$); -4.3 (AlCH$_3$).

Preparation and Characterization of **LH(AlI₂)**[6]

METHOD A: To a colorless solution of **LH(AlMe₂)**[6] (1.443 g; 2.43 mmol) in toluene (40 mL) cooled to -40 C, a solution of I_2 (1.233 g; 4.86 mmol) in toluene (20 mL) was added. The reaction mixture was allowed to warm to room temperature and heated to 60 °C for 72 hours with gradual precipitation of a yellow solid. The volatiles were evaporated under vacuum with the yield of 2.052 g (>99 %) of yellow crystalline **LH(AlI₂)**[6].

METHOD B: To a suspension of **LH(AlMeI)**[6] (0.564 g; 0.82 mmol) in toluene (20 mL) cooled to -40 C, a solution of I_2 (0.208 g; 0.82 mmol) in toluene (10 mL) was added. The reaction mixture was allowed to warm to room temperature and heated to 60 °C for 72 hours with gradual precipitation of a yellow solid. The volatiles were evaporated under vacuum with the yield of 0.653 g (>99 %) of yellow crystalline **LH(AlI₂)**[6].

Single crystals suitable for *sc*XRD analyses were obtained by cooling of a saturated solution of **LH(AlI₂)**[6] in Et_2O to 7 °C. ^{1}H NMR (C_6D_6, 500.20 MHz, 295 K) δ: 7.19–6.99 (m, 6H, ArH^{Dipp}); 6.10 (s, 1H, NH^{Dipp}); 4.72 (s, 1H, ArH^{Prm}); 3.84 (m, 2H, $^3J_{H,H}$ = 6.8 Hz, CH^{Dipp}); 3.28 (s, 3H, O-CH_3); 3.26 (m, 2H, $^3J_{H,H}$ = 6.9 Hz, CH^{Dipp}); 2.84 (s, 3H, O-CH_3); 1.55 (d, 6H, $^3J_{H,H}$ = 6.8 Hz, CH_3^{Dipp}); 1.46–1.39 (m, 6H, CH_3^{Dipp}); 1.25 (d, 6H, $^3J_{H,H}$ = 6.8 Hz, CH_3^{Dipp}); 1.04–0.98 (m, 6H, CH_3^{Dipp}). ^{13}C NMR (C_6D_6, 125.78 MHz, 295 K) δ: 174.4 (ArC_q^{OMe}); 168.2 (ArC_q^{OMe}); 161.0 (ArC_q^{Gua}); 160.1 (ArC_q^{Prm}); 147.2 (ArC_q^{Dipp}); 146.7 (ArC_q^{Dipp}); 137.2 (ArC_q^{Dipp}); 132.6 (ArC_q^{Dipp}); 129.1 (ArCH^{Dipp}); 128.9 (ArCH^{Dipp}); 126.0 (ArCH^{Dipp}); 124.1 (ArCH^{Dipp}); 79.7 (ArCH^{Prm}); 55.4 (O-CH$_3$); 54.8 (O-CH$_3$); 29.6 (CH^{Dipp}); 29.5 (CH^{Dipp}); 26.6 (CH$_3^{Dipp}$); 25.3 (CH$_3^{Dipp}$); 24.7 (CH$_3^{Dipp}$); 24.3 (CH$_3^{Dipp}$).

Preparation and Characterization of **LH(AlMeCl)**[6]

To a colorless solution of **LH_2** (0.512 g; 0.99 mmol) in Et_2O (20 mL) cooled to -80 C, a 1.0 M solution of Me_2AlCl in hexane (0.99 mL; 0.99 mmol) was added. The reaction mixture was allowed to warm to room temperature and stirred for 3 hours with gradual precipitation of a white solid. The precipitate was separated by filtration and dried under vacuum with the yield of 0.406 g (69 %) of white crystalline **LH(AlMeCl)**[6]. Single crystals suitable for *sc*XRD analyses were obtained by cooling of a saturated solution of **LH(AlMeCl)**[6] in Et_2O/hexane (1:1) mixture to 7 °C. ^1H NMR (C_6D_6, 500.13 MHz, 295 K) δ: 7.18 (m, 3H, ArH^{Dipp}); 7.11 (d, 1H, $^3J_{H,H}$ = 7.4 Hz, ArH^{Dipp}); 7.07 (m, 2H, ArH^{Dipp}); 5.80 (s, 1H, NH^{Dipp}); 4.81 (s, 1H, ArH^{Prm}); 4.23 (m, 1H, $^3J_{H,H}$ = 6.7 Hz, CH^{Dipp}); 3.42 (br s, 1H, CH^{Dipp}); 3.39 (m, 1H, $^3J_{H,H}$ = 6.9 Hz, CH^{Dipp}); 3.35 (s, 3H, O-CH_3); 3.15 (br s, 1H, CH^{Dipp}); 2.76 (s, 3H, O-CH_3); 1.58 (d, 6H, $^3J_{H,H}$ = 6.6 Hz, CH_3^{Dipp}); 1.41 (br s, 3H, CH_3^{Dipp}); 1.28 (d, 9H, $^3J_{H,H}$ = 6.8 Hz, CH_3^{Dipp}); 1.19 (br s, 3H, CH_3^{Dipp}); 0.94 (d, ^{3}H, $^3J_{H,H}$ = 5.5 Hz, CH_3^{Dipp}); -0.20 (s, 3H, AlCH_3). ^{13}C NMR (C_6D_6, 125.76 MHz, 295 K) δ: 174.0 (ArC_q^{OMe}); 169.1 (ArC_q^{OMe}); 161.0 (ArC_q^{Prm}) ; 161.0 (ArC_q^{Gua}); 147.5 (ArC_q^{Dipp}); 147.5 (ArC_q^{Dipp}) ; 146.4 (ArC_q^{Dipp}); 146.1 (ArC_q^{Dipp}); 137.5 (ArC_q^{Dipp}); 133.3 (ArC_q^{Dipp}); 128.7 (ArCH^{Dipp}); 128.5 (ArCH^{Dipp}); 126.0 (ArCH^{Dipp}); 125.3 (ArCH^{Dipp}); 124.3 (ArCH^{Dipp}); 123.6 (ArCH^{Dipp}); 78.7 (ArCH^{Prm}); 55.6 (O-CH_3); 54.5 (O-CH_3); 29.7 (CH^{Dipp}); 29.3 (CH^{Dipp}); 29.1 (CH^{Dipp}); 28.9 (CH^{Dipp}); 27.4 (CH_3^{Dipp}); 25.5 (CH_3^{Dipp}); 25.2 (CH_3^{Dipp}); 25.0 (CH_3^{Dipp}); 24.5 (CH_3^{Dipp}); 24.5 (CH_3^{Dipp}); 24.5 (CH_3^{Dipp}); 24.0 (CH_3^{Dipp}); -8.1 (AlCH_3).

Preparation and Characterization of **LH(AlCl$_2$)**[6]

METHOD A: To a colorless solution of **LH_2** (0.668 g; 1.29 mmol) in Et_2O (40 mL) cooled to -80 C, a 1.0 M solution of $EtAlCl_2$ in hexane (1.29 mL; 1.29 mmol) was added. The reaction mixture was allowed to warm to room temperature and stirred for 24 hours with gradual precipitation of a white solid. The precipitate was separated by filtration and dried under vacuum with the yield of 0.610 g (77 %) of white crystalline **LH(AlCl$_2$)**[6].
METHOD B: To a colorless solution of **LH(Li)**[4] (0.529 g; 1.01 mmol) in Et_2O (55 mL) cooled to -80 C, a solution of $AlCl_3$ (0.135 g; 1.01 mmol) in Et_2O (5 mL) was added. The reaction mixture was allowed to warm to room temperature and stirred for 24 hours with gradual precipitation of a white solid. The precipitate was separated by filtration; the filtrate was recrystallized and dried under vacuum with the yield of 0.360 g (58 %) of white crystalline **LH(AlCl$_2$)**[6]. Single crystals suitable for *sc*XRD analyses were obtained from a saturated solution of **LH(AlCl$_2$)**[6] in Et_2O at room temperature. ^1H NMR (THF-d_8, 500.13 MHz, 295 K) δ: 7.34–7.13 (m, 3H, ArH^{Dipp}); 7.12–7.06 (m, 3H, ArH^{Dipp}); 6.60 (s, 1H, NH^{Dipp}); 5.80 (s, 1H, ArH^{Prm}); 3.94 (s, 3H, O-CH_3); 3.80 (s, 3H, O-CH_3); 3.45 (m, 2H, $^3J_{H,H}$ = 6.7 Hz, CH^{Dipp}); 3.15 (m, 2H, $^3J_{H,H}$ = 6.8 Hz, CH^{Dipp}); 1.33 (d, 6H, $^3J_{H,H}$ = 6.9 Hz, CH_3^{Dipp}); 1.30 (d, 6H, $^3J_{H,H}$ = 6.6 Hz, CH_3^{Dipp}); 1.27–1.21 (m, 6H, CH_3^{Dipp}); 1.12–1.06 (m, 6H, CH_3^{Dipp}).

Preparation and Characterization of **LH(BH$_2$)**[6]

To a colorless solution of **LH_2** (1.624 g; 3.14 mmol) in Et_2O (60 mL) cooled to -80 C, BH_3□Me_2S (0.30 mL; 3.14 mmol) was added under argon atmosphere. The reaction mixture was allowed to warm to room temperature and stirred for 24 hours with gradual precipitation of a white solid. The solid was separated by filtration on air and recrystallized

from Et_2O with the yield of 0.864 g (52 %) of white crystalline **LH(BH$_2$)**^{6}. Single crystals suitable for *sc*XRD analyses were obtained by cooling of a saturated solution of **LH(BH$_2$)**^{6} in Et_2O to 7 °C. ^{1}H NMR (C_6D_6, 500.13 MHz, 295 K) δ: 7.24–7.18 (m, 3H, Ar*H*^{Dipp}); 7.12–7.08 (m, 1H, Ar*H*^{Dipp}); 7.06–7.03 (m, 2H, Ar*H*^{Dipp}); 5.32 (s, 1H, N*H*^{Dipp}); 4.91 (s, 1H, Ar*H*^{Prm}); 3.95 (br s, 2H, B*H*$_2$); 3.73 (m, 2H, $^{3}J_{H,H}$ = 6.8 Hz, C*H*^{Dipp}); 3.42 (s, 3H, O-C*H*$_3$); 3.26 (m, 2H, $^{3}J_{H,H}$ = 6.7 Hz, C*H*^{Dipp}); 2.80 (s, 3H, O-C*H*$_3$); 1.51 (d, 6H, $^{3}J_{H,H}$ = 6.6 Hz, C*H*$_3^{Dipp}$); 1.50 (br s, 6H, C*H*$_3^{Dipp}$); 1.34 (d, 6H, $^{3}J_{H,H}$ = 6.9 Hz, C*H*$_3^{Dipp}$); 1.09 (br s, 6H, C*H*$_3^{Dipp}$). ^{13}C NMR (C_6D_6, 125.76 MHz, 295 K) δ: 172.8 (Ar*C*$_q^{OMe}$); 167.0 (Ar*C*$_q^{OMe}$); 160.6 (Ar*C*$_q^{Prm}$); 158.5 (Ar*C*$_q^{Gua}$); 147.6 (Ar*C*$_q^{Dipp}$); 147.0 (Ar*C*$_q^{Dipp}$); 137.8 (Ar*C*$_q^{Dipp}$); 133.2 (Ar*C*$_q^{Dipp}$); 128.9 (Ar*C*H^{Dipp}); 128.5 (Ar*C*H^{Dipp}); 125.5 (Ar*C*H^{Dipp}); 123.8 (Ar*C*H^{Dipp}); 78.8 (Ar*C*H^{Prm}); 55.8 (O-*C*H$_3$); 54.3 (O-*C*H$_3$); 29.3 (*C*H^{Dipp}); 29.3 (*C*H^{Dipp}); 26.7 (*C*H$_3^{Dipp}$); 24.9 (br s, *C*H$_3^{Dipp}$); 24.5 (*C*H$_3^{Dipp}$); 24.3 (br s, *C*H$_3^{Dipp}$). ^{11}B NMR (C_6D_6, 160.46 MHz, 295 K) δ: -8.5 (br s, *B*H$_2$). ^{1}H NMR (THF-d$_8$, 500.13 MHz, 295 K) δ: 7.26–7.23 (m, 3H, Ar*H*^{Dipp}); 7.18–7.13 (m, 1H, Ar*H*^{Dipp}); 7.10–7.05 (m, 2H, Ar*H*^{Dipp}); 6.28 (s, 1H, N*H*^{Dipp}); 5.61 (s, 1H, Ar*H*^{Prm}); 3.80 (s, 3H, O-C*H*$_3$); 3.75 (s, 3H, O-C*H*$_3$); 3.40 (m, 2H, $^{3}J_{H,H}$ = 6.8 Hz, C*H*^{Dipp}); 3.19 (m, 2H, $^{3}J_{H,H}$ = 6.8 Hz, C*H*^{Dipp}); 3.16 (br s, 2H, B*H*$_2$); 1.33 (d, 6H, $^{3}J_{H,H}$ = 6.9 Hz, C*H*$_3^{Dipp}$); 1.27 (d, 6H, $^{3}J_{H,H}$ = 6.7 Hz, C*H*$_3^{Dipp}$); 1.25 (d, 6H, $^{3}J_{H,H}$ = 6.7 Hz, C*H*$_3^{Dipp}$); 1.10 (d, 6H, $^{3}J_{H,H}$ = 6.4 Hz, C*H*$_3^{Dipp}$).

Preparation and Characterization of **LH(BF$_2$)**^{6}

METHOD A: To a colorless solution of **LH$_2$** (0.428 g; 0.83 mmol) in toluene (20 mL) at room temperature, BF_3□Et_2O (0.10 mL; 0.83 mmol) was added under argon atmosphere. The reaction mixture was stirred for 2 hours with gradual precipitation of a white solid. The solid was separated by filtration on air, dissolved in toluene (40 mL) and extracted by 5 mL of H_2O. The organic phase was separated and the toluene evaporated under vacuum with the yield of 0.411 g (88 %) of white crystalline **LH(BF$_2$)**^{6}.

METHOD B: To a colorless solution of **LH(Li)**^{4} (0.545 g; 1.04 mmol) in Et_2O (20 mL) at room temperature, BF_3□Et_2O (0.17 mL; 1.04 mmol) was added. The reaction mixture was stirred for 24 hours with gradual precipitation of a white solid. The solid was separated by filtration, the filtrate was evaporated under vacuum, dissolved in toluene (40 mL) and extracted by 5 mL of H_2O. The organic phase was separated and the toluene evaporated under vacuum with the yield of 0.474 g (86 %) of white crystalline **LH(BF$_2$)**^{6}.

Single crystals suitable for *sc*XRD analyses were obtained by free evaporation of the solvent from a saturated solution of **LH(BF$_2$)**^{6} in THF at room temperature. ^{1}H NMR (C_6D_6, 500.13 MHz, 295 K) δ: 7.21 (s, 3H, Ar*H*^{Dipp}); 7.11–7.07 (m, 1H, Ar*H*^{Dipp}); 7.05–7.01 (m, 2H, Ar*H*^{Dipp}); 5.55 (s, 1H, N*H*^{Dipp}); 4.83 (s, 1H, Ar*H*^{Prm}); 3.66 (m, 2H, $^{3}J_{H,H}$ = 6.7 Hz, C*H*^{Dipp}); 3.38 (s, 3H, O-C*H*$_3$); 3.20 (m, 2H, $^{3}J_{H,H}$ = 6.8 Hz, C*H*^{Dipp}); 2.82 (s, 3H, O-C*H*$_3$); 1.59 (d, 6H, $^{3}J_{H,H}$ = 6.5 Hz, C*H*$_3^{Dipp}$); 1.43 (d, 6H, $^{3}J_{H,H}$ = 5.4 Hz, C*H*$_3^{Dipp}$); 1.30 (d, 6H, $^{3}J_{H,H}$ = 6.9 Hz, C*H*$_3^{Dipp}$); 1.06 (d, 6H, $^{3}J_{H,H}$ = 5.4 Hz, C*H*$_3^{Dipp}$). ^{13}C NMR (C_6D_6, 125.76 MHz, 295 K) δ: 173.1 (Ar*C*$_q^{OMe}$); 168.3 (t, $^{4}J_{C,F}$ = 2.6 Hz, Ar*C*$_q^{OMe}$); 158.8 (Ar*C*$_q^{Prm}$); 158.2 (Ar*C*$_q^{Gua}$); 149.1 (Ar*C*$_q^{Dipp}$); 147.0 (Ar*C*$_q^{Dipp}$); 132.7 (Ar*C*$_q^{Dipp}$); 132.6 (Ar*C*$_q^{Dipp}$); 129.6 (Ar*C*H^{Dipp}); 128.8 (Ar*C*H^{Dipp}); 125.8 (Ar*C*H^{Dipp}); 124.0 (Ar*C*H^{Dipp}); 79.4 (Ar*C*H^{Prm}); 56.1 (O-*C*H$_3$); 54.5 (O-*C*H$_3$); 29.6 (*C*H^{Dipp}); 29.3 (*C*H^{Dipp}); 26.3 (t, $^{6}J_{C,F}$ = 4.0 Hz, *C*H$_3^{Dipp}$); 25.7 (*C*H$_3^{Dipp}$); 24.9 (br s, *C*H$_3^{Dipp}$); 24.3 (br s, *C*H$_3^{Dipp}$). ^{19}F NMR (C_6D_6, 470.59 MHz, 295 K) δ: -132.2 (non-binomial quartet, 2F, $^{1}J_{F,B}$ = 26.5 Hz, B*F*$_2$). ^{11}B NMR (C_6D_6, 160.46 MHz, 295 K) δ: 1.15 (dd as t, 1B, $^{1}J_{B,F}$ = 26.5 Hz, *B*F$_2$).

^{1}H NMR (THF-d$_8$, 500.20 MHz, 295 K) δ: 7.34–7.27 (m, 3H, Ar*H*^{Dipp}); 7.21–7.17 (m, 1H, Ar*H*^{Dipp}); 7.10 (d, 2H, $^{3}J_{H,H}$ = 7.7 Hz, Ar*H*^{Dipp}); 6.58 (s, 1H, N*H*^{Dipp}); 5.74 (s, 1H, Ar*H*^{Prm}); 3.88 (s, 3H, O-C*H*$_3$); 3.80 (s, 3H, O-C*H*$_3$); 3.29 (m, 2H, $^{3}J_{H,H}$ = 6.7

Hz, CH^{Dipp}); 3.16 (m, 2H, $^3J_{H,H}$ = 6.8 Hz, CH^{Dipp}); 1.31 (d, 6H, $^3J_{H,H}$ = 6.9 Hz, CH_3^{Dipp}); 1.29 (d, 6H, $^3J_{H,H}$ = 6.6 Hz, CH_3^{Dipp}); 1.21 (d, 6H, $^3J_{H,H}$ = 6.6 Hz, CH_3^{Dipp}); 1.11 (d, 6H, $^3J_{H,H}$ = 6.7 Hz, CH_3^{Dipp}). ^{13}C NMR (THF-d$_8$, 125.76 MHz, 295 K) δ: 173.8 (ArC_q^{OMe}); 169.1 (t, $^4J_{C,F}$ = 2.6 Hz, ArC_q^{OMe}); 158.9 (ArC_q^{Prm}); 158.4 (ArC_q^{Gua}); 149.5 (ArC_q^{Dipp}); 147.6 (ArC_q^{Dipp}); 134.2 (ArC_q^{Dipp}); 133.4 (ArC_q^{Dipp}); 129.3 (ArCH^{Dipp}); 128.4 (ArCH^{Dipp}); 125.5 (ArCH^{Dipp}); 123.9 (ArCH^{Dipp}); 79.4 (ArCH^{Prm}); 57.2 (O-CH_3); 54.6 (O-CH_3); 29.7 (CH^{Dipp}); 29.4 (CH^{Dipp}); 25.8 (2x CH_3^{Dipp}); 24.9 (CH_3^{Dipp}); 24.2 (CH_3^{Dipp}). ^{19}F NMR (THF-d$_8$, 470.59 MHz, 295 K) δ: -133.5 (non-binomial quartet, 2F, $^1J_{F,B}$ = 26.6 Hz, BF_2). ^{11}B NMR (THF-d$_8$, 160.46 MHz, 295 K) δ: 0.35 (dd as t, 1B, ^{1}J = 26.6 Hz, BF$_2$).

Preparation and Characterization of $\mathbf{L(Li)_2}^{4,6}$

METHOD A: To a colorless solution of **LH₂** (0.600 g; 1.16 mmol) in Et$_2$O (30 mL) cooled to -80 C, a 1.6 M solution of ^{n}BuLi in hexane (1.45 mL; 2.32 mmol) was added. The reaction mixture was allowed to warm to room temperature and stirred for 24 hours. The volatiles were evaporated under vacuum with the yield of 0.611 g (>99 %) of white $\mathbf{L(Li)_2}^{4,6}$.

METHOD B: To a colorless solution of $\mathbf{LH(Li)}^{4}$ (0.420 g; 0.80 mmol) in Et$_2$O (20 mL) cooled to -80 C, a 1.6 M solution of ^{n}BuLi in hexane (0.50 mL; 0.80 mmol) was added. The reaction mixture was allowed to warm to room temperature and stirred for 24 hours. The volatiles were evaporated under vacuum with the yield of 0.421 g (>99 %) of white $\mathbf{L(Li)_2}^{4,6}$. ^{1}H NMR (THF-d$_8$, 500.20 MHz, 295 K) δ: 6.86 (d, 2H, $^3J_{H,H}$ = 7.3 Hz, ArH^{Dipp}); 6.72 (d, 2H, $^3J_{H,H}$ = 7.3 Hz, ArH^{Dipp}); 6.66 (t, 1H, $^3J_{H,H}$ = 7.4 Hz, ArH^{Dipp}); 6.40 (t, 1H, $^3J_{H,H}$ = 7.3 Hz, ArH^{Dipp}); 4.90 (s, 1H, ArH^{Prm}); 3.78 (m, 2H, $^3J_{H,H}$ = 6.8 Hz, CH^{Dipp}); 3.70 (s, 3H, O-CH_3); 3.60 (s, 3H, O-CH_3); 3.37 (m, 2H, $^3J_{H,H}$ = 6.7 Hz, CH^{Dipp}); 1.18 (d, 6H, $^3J_{H,H}$ = 6.7 Hz, CH_3^{Dipp}); 1.11 (d, 6H, $^3J_{H,H}$ = 7.0 Hz, CH_3^{Dipp}); 1.06 (d, 6H, $^3J_{H,H}$ = 6.7 Hz, CH_3^{Dipp}); 0.88 (d, 6H, $^3J_{H,H}$ = 6.4 Hz, CH_3^{Dipp}). ^{13}C NMR (THF-d$_8$, 125.78 MHz, 295 K) δ: 174.2 (ArC_q^{OMe}); 172.3 (ArC_q^{OMe}); 162.8 (ArC_q^{Prm}); 157.0 (ArC_q^{Gua}); 154.1 (ArC_q^{Dipp}); 153.5 (ArC_q^{Dipp}); 144.2 (ArC_q^{Dipp}); 141.3 (ArC_q^{Dipp}); 122.2 (2x ArCH^{Dipp}); 120.0 (ArCH^{Dipp}); 116.6 (ArCH^{Dipp}); 69.8 (ArCH^{Prm}); 54.3 (O-CH_3); 52.7 (O-CH_3); 28.6 (CH^{Dipp}); 28.6 (CH^{Dipp}); 25.6 (CH_3^{Dipp}); 25.4 (CH_3^{Dipp}); 25.2 (CH_3^{Dipp}); 24.5 (CH_3^{Dipp}). ^{7}Li NMR (THF-d$_8$, 194.40 MHz, 295 K) δ: +1.4 (1Li); +0.3 (1Li)

Preparation and Characterization of $\mathbf{L(AlMe_2)_2}^{4,4}$

To a colorless solution of **LH₂** (0.350 g; 0.68 mmol) in hexane (15 mL) cooled to -80 C, a 2.0 M solution of Me$_3$Al in hexane (0.68 mL; 1.35 mmol) was added. The reaction mixture was allowed to warm to room temperature and stirred for 24 hours with gradual precipitation of a white solid. The volatiles were evaporated under vacuum with the yield of 0.423 g (>99 %) of white crystalline $\mathbf{L(AlMe_2)_2}^{4,4}$. Single crystals suitable for *sc*XRD analyses were obtained by cooling of a saturated solution of $\mathbf{L(AlMe_2)_2}^{4,4}$ in hexane to -30 °C. ^{1}H NMR (Tol-d$_8$, 400.16 MHz, 295 K) δ: 7.04–7.01 (m, 6H, ArH^{Dipp}); 4.83 (s, 1H, ArH^{Prm}); 3.72 (m, 4H, $^3J_{H,H}$ = 6.8 Hz, CH^{Dipp}); 3.10 (s, 3H, O-CH_3); 2.80 (s, 3H, O-CH_3); 1.28 (d, 12H, $^3J_{H,H}$ = 6.7 Hz, CH_3^{Dipp}); 1.23 (d, 12H, $^3J_{H,H}$ = 6.9 Hz, CH_3^{Dipp}); -0.19 (s, 6H, AlCH_3); -0.73 (s, 6H, AlCH_3). ^{1}H NMR (C$_6$D$_6$, 500.20 MHz, 295 K) δ: 7.10–7.05 (m, 6H, ArH^{Dipp}); 4.84 (s, 1H, ArH^{Prm}); 3.77 (m, 4H, $^3J_{H,H}$ = 6.8 Hz, CH^{Dipp}); 3.09 (s, 3H, O-CH_3); 2.72 (s, 3H, O-CH_3); 1.31 (d, 12H, $^3J_{H,H}$ = 6.7 Hz, CH_3^{Dipp}); 1.26 (d, 12H, $^3J_{H,H}$ = 6.8 Hz, CH_3^{Dipp}); -0.10 (s, 6H, AlCH_3); -0.64 (s, 6H, AlCH_3). ^{13}C NMR (C$_6$D$_6$, 125.76 MHz, 295 K) δ: 175.7 (ArC_q^{OMe}); 168.1

(ArC_q^{OMe}); 161.4 (ArC_q^{Prm}); 160.2 (ArC_q^{Gua}); 144.7 (ArC_q^{Dipp}); 139.4 (ArC_q^{Dipp}); 125.9 (ArCH^{Dipp}); 124.4 (ArCH^{Dipp}); 80.7 (ArCH^{Prm}); 56.0 (O-CH_3); 55.2 (O-CH_3); 29.0 (CH^{Dipp}); 26.8 (CH_3^{Dipp}); 23.4 (CH_3^{Dipp}); -8.4 (AlCH_3^{Dipp}); -10.7 (AlCH_3^{Dipp}).

Preparation and Characterization of **L(AlMeCl)$_2$**^{4,4}

This isomer was isolated from the reaction of **LH$_2$** with 2 eq. of Me_2AlCl in hexane only in the form of single crystals when the crude mixture containing *ca* 40 molar % of **L(AlMeCl)$_2$**^{4,4} (rest are some unidentified side product and **L(AlMeCl)$_2$**^{6,4} isomer) was cooled to 7 °C for a couple of days because of difficulties with the stability. Nonetheless, several key NMR parameters from C_6D_6 solution could be accurately determined with the help of 2D NMR techniques - such as $^1H/^{13}C$ (ArH^{Prm}): 4.77/81.7 ppm; ^1H (both AlCH_3): 0.04 and -0.86 ppm; ^1H (both O-CH_3): 3.02 and 2.67 ppm; -0.86 ppm; ^{13}C (both ArC_q^{OMe}): 176.2 and 167.7 ppm; ^{13}C (ArC_q^{Gua}): 161.6 ppm; ^{13}C (ArC_q^{Prm}): 161.2 ppm.

Preparation and Characterization of **L(BH$_2$)$_2$**^{4,4}

To a colorless solution of **LH$_2$** (0.695 g; 1.34 mmol) in Et_2O (30 mL) cooled to -80 C, BH_3□Me_2S (0.25 mL; 2.68 mmol) was added. The reaction mixture was allowed to warm to room temperature and stirred for 24 hours with gradual precipitation of a white solid. The solid was separated by filtration and dried under vacuum with the yield of 0.603 g (83 %) of white crystalline **L(BH$_2$)$_2$**^{4,4}. ^1H NMR (C_6D_6, 500.13 MHz, 295 K) δ: 7.05–6.97 (m, 2H, ArH^{Dipp}); 6.94 (d, 2H, $^3J_{H,H}$ = 7.7 Hz, ArH^{Dipp}); 6.91 (d, 2H, $^3J_{H,H}$ = 7.5 Hz, ArH^{Dipp}); 5.04 (s, 1H, ArH^{Prm}); 4.51 (br s, 2H, BH_2); 3.79 (br s, 2H, BH_2); 3.54 (s, 3H, O-CH_3); 3.41 (m, 2H, $^3J_{H,H}$ = 6.8 Hz, CH^{Dipp}); 3.20 (m, 2H, $^3J_{H,H}$ = 6.8 Hz, CH^{Dipp}); 2.75 (s, 3H, O-CH_3); 1.32 (d, 6H, $^3J_{H,H}$ = 6.8 Hz, CH_3^{Dipp}); 1.21 (d, 6H, $^3J_{H,H}$ = 6.8 Hz, CH_3^{Dipp}); 1.09 (d, 6H, $^3J_{H,H}$ = 6.9 Hz, CH_3^{Dipp}); 0.92 (d, 6H, $^3J_{H,H}$ = 6.8 Hz, CH_3^{Dipp}). ^{13}C NMR (C_6D_6, 125.76 MHz, 295 K) δ: 173.4 (ArC_q^{OMe}); 166.8 (ArC_q^{OMe}); 157.6 (ArC_q^{Gua}); 155.6 (ArC_q^{Prm}); 147.3 (ArC_q^{Dipp}); 146.4 (ArC_q^{Dipp}); 140.3 (ArC_q^{Dipp}); 136.2 (ArC_q^{Dipp}); 128.0 (ArCH^{Dipp}); 127.8 (ArCH^{Dipp}); 124.0 (ArCH^{Dipp}); 124.0 (ArCH^{Dipp}); 80.5 (ArCH^{Prm}); 56.1 (O-CH_3); 55.0 (O-CH_3); 29.8 (CH^{Dipp}); 29.3 (CH^{Dipp}); 27.3 (CH_3^{Dipp}); 26.0 (CH_3^{Dipp}); 22.5 (CH_3^{Dipp}) ; 22.5 (CH_3^{Dipp}). ^{11}B NMR (C_6D_6, 160.46 MHz, 295 K) δ: +1.8 (br s, BH_2); -7.1 (br s, BH_2).

Preparation and Characterization of **L(AlMe$_2$)$_2$**^{6,4}

METHOD A: To a colorless solution of **LH$_2$** (0.805 g; 1.55 mmol) in Et_2O (40 mL) cooled to -80 C, a 2.0 M solution of Me_3Al in hexane (1.55 mL; 3.11 mmol) was added. The reaction mixture was allowed to warm to room temperature and stirred for 24 hours. The volatiles were evaporated under vacuum with the yield of 0.970 g (>99 %) of white crystalline **L(AlMe$_2$)$_2$**^{6,4}.

METHOD B: To a suspension of **LH(AlMe$_2$)**^{6} (0.329 g; 0.57 mmol) in Et_2O (15 mL) cooled to -80 C, a 2.0 M solution of Me_3Al in hexane (0.29 mL; 0.57 mmol) was added. The reaction mixture was allowed to warm to room temperature and stirred for 24 hours with gradual dissolving of the solid. The volatiles were evaporated under vacuum with the yield of 0.358 g (>99 %) of white crystalline **L(AlMe$_2$)$_2$**^{6,4}.

Single crystals suitable for *sc*XRD analyses were obtained by cooling of a saturated solution of **L(AlMe$_2$)$_2$**^{6,4} in hexane to -30 °C.^1H NMR (Tol-d_8, 400.16 MHz, 295 K) δ: 7.06 (d, 1H, $^3J_{H,H}$ = 7.6 Hz, ArH^{Dipp}) 7.01–6.98 (m, 1H, ArH^{Dipp}); 6.92 (m, 2H, ArH^{Dipp}); 6.76 (d, 1H, $^3J_{H,H}$ = 7.3 Hz, ArH^{Dipp}); 6.71 (d, 1H, $^3J_{H,H}$ = 7.4 Hz, ArH^{Dipp}); 4.96 (s, 1H, ArH^{Prm}); 3.62 (s,

3H, O-CH_3); 3.57 (m, 1H, $^3J_{H,H}$ = 6.7 Hz, CH^{Dipp}); 3.20 (m, 1H, $^3J_{H,H}$ = 6.7 Hz, CH^{Dipp}); 3.14 (m, 1H, $^3J_{H,H}$ = 6.7 Hz, CH^{Dipp}); 2.92 (m, 1H, $^3J_{H,H}$ = 6.7 Hz, CH^{Dipp}); 2.84 (s, 3H, O-CH_3); 1.59 (d, 3H, $^3J_{H,H}$ = 6.7 Hz, CH_3^{Dipp}); 1.42 (d, 3H, $^3J_{H,H}$ = 6.6 Hz, CH_3^{Dipp}); 1.29 (d, 3H, $^3J_{H,H}$ = 6.6 Hz, CH_3^{Dipp}); 1.24 (d, 3H, $^3J_{H,H}$ = 6.7 Hz, CH_3^{Dipp}); 1.03 (t, 6H, $^3J_{H,H}$ = 6.4 Hz, CH_3^{Dipp}); 0.58 (d, 3H, $^3J_{H,H}$ = 6.7 Hz, CH_3^{Dipp}); 0.42 (d, 3H, $^3J_{H,H}$ = 6.6 Hz, CH_3^{Dipp}); -0.10 (s, 3H, AlCH_3); -0.17 (s, 3H, AlCH_3) -0.24 (s, 3H, AlCH_3); -0.82 (s, 3H, AlCH_3). ^{1}H NMR (C_6D_6, 500.20 MHz, 295 K) δ: 7.10 (d, 1H, $^3J_{H,H}$ = 7.2 Hz, ArH^{Dipp}) 7.02 (d, 1H, $^3J_{H,H}$ = 6.8 Hz, ArH^{Dipp}); 6.97 (m, 2H, ArH^{Dipp}); 6.82 (d, 1H, $^3J_{H,H}$ = 7.5 Hz, ArH^{Dipp}); 6.76 (d, 1H, $^3J_{H,H}$ = 7.4 Hz, ArH^{Dipp}); 4.94 (s, 1H, ArH^{Prm}); 3.65 (m, 1H, $^3J_{H,H}$ = 6.7 Hz, CH^{Dipp}); 3.58 (s, 3H, O-CH_3); 3.28 (m, 1H, $^3J_{H,H}$ = 6.7 Hz, CH^{Dipp}); 3.22 (m, 1H, $^3J_{H,H}$ = 6.7 Hz, CH^{Dipp}); 2.99 (m, 1H, $^3J_{H,H}$ = 6.7 Hz, CH^{Dipp}); 2.70 (s, 3H, O-CH_3); 1.63 (d, 3H, $^3J_{H,H}$ = 6.7 Hz, CH_3^{Dipp}); 1.44 (d, 3H, $^3J_{H,H}$ = 6.7 Hz, CH_3^{Dipp}); 1.34 (d, 3H, $^3J_{H,H}$ = 6.6 Hz, CH_3^{Dipp}); 1.30 (d, 3H, $^3J_{H,H}$ = 6.8 Hz, CH_3^{Dipp}); 1.10 (d, 3H, $^3J_{H,H}$ = 6.8 Hz, CH_3^{Dipp}); 1.08 (d, 3H, $^3J_{H,H}$ = 6.7 Hz, CH_3^{Dipp}); 0.64 (d, 3H, $^3J_{H,H}$ = 6.8 Hz, CH_3^{Dipp}); 0.49 (d, 3H, $^3J_{H,H}$ = 6.6 Hz, CH_3^{Dipp}); 0.02 (s, 3H, AlCH_3); -0.06 (s, 3H, AlCH_3); -0.12 (s, 3H, AlCH_3); -0.69 (s, 3H, AlCH_3). ^{13}C NMR (C_6D_6, 125.78 MHz, 295 K) δ: 174.0 (ArC_q^{OMe}); 169.8 (ArC_q^{OMe}); 164.7 (ArC_q^{Gua}); 158.2 (ArC_q^{Prm}); 145.9 (ArC_q^{Dipp}); 145.5 (ArC_q^{Dipp}); 145.0 (ArC_q^{Dipp}); 144.4 (ArC_q^{Dipp}); 140.5 (ArC_q^{Dipp}); 138.4 (ArC_q^{Dipp}); 126.8 (ArCH^{Dipp}); 126.6 (ArCH^{Dipp}); 125.2 (ArCH^{Dipp}); 124.7 (ArCH^{Dipp}); 124.6 (ArCH^{Dipp}); 123.4 (ArCH^{Dipp}); 79.3 (ArCH^{Prm}); 55.8 (O-CH$_3$); 54.6 (O-CH$_3$); 29.4 (CH^{iPr}); 29.1 (CH^{iPr}); 28.7 (CH^{iPr}); 28.6 (CH^{iPr}); 28.3 (CH$_3^{iPr}$); 27.7 (CH$_3^{iPr}$); 26.4 (CH$_3^{iPr}$); 25.5 (CH$_3^{iPr}$); 25.1 (CH$_3^{iPr}$); 23.9 (CH$_3^{iPr}$); 23.0 (CH$_3^{iPr}$); 22.4 (CH$_3^{iPr}$); -7.7 (AlCH$_3$); -7.8 (AlCH$_3$); -8.3 (AlCH$_3$); -9.0 (AlCH$_3$). ^{1}H NMR (THF-d_8, 400.13 MHz, 295 K) δ: 7.09 (d, 1H, $^3J_{H,H}$ = 7.5 Hz, ArH^{Dipp}); 7.03 (d, 1H, $^3J_{H,H}$ = 7.6 Hz, ArH^{Dipp}); 6.96 (d, 1H, $^3J_{H,H}$ = 7.8 Hz, ArH^{Dipp}); 6.92 (d, 1H, $^3J_{H,H}$ = 7.8 Hz, ArH^{Dipp}); 6.75 (d, 1H, $^3J_{H,H}$ = 7.6 Hz, ArH^{Dipp}); 6.71 (d, 1H, $^{33}J_{H,H}$ = 7.5 Hz, ArH^{Dipp}); 5.93 (s, 1H, ArH^{Prm}); 4.02 (s, 3H, O-CH_3); 3.93 (s, 3H, O-CH_3); 3.37 (m, 1H, $^3J_{H,H}$ = 6.7 Hz, CH^{Dipp}); 3.03 (m, 1H, $^3J_{H,H}$ = 6.7 Hz, CH^{Dipp}); 2.94 (m, 1H, $^3J_{H,H}$ = 6.7 Hz, CH^{Dipp}); 2.74 (m, 1H, $^3J_{H,H}$ = 6.8 Hz, CH^{Dipp}); 1.43 (d, 3H, $^3J_{H,H}$ = 6.7 Hz, CH_3^{Dipp}); 1.31 (d, 3H, $^3J_{H,H}$ = 6.8 Hz, CH_3^{Dipp}); 1.16 (d, 3H, $^3J_{H,H}$ = 6.7 Hz, CH_3^{Dipp}); 1.03 (d, 3H, $^3J_{H,H}$ = 6.7 Hz, CH_3^{Dipp}); 0.89 (d, 3H, $^3J_{H,H}$ = 6.7 Hz, CH_3^{Dipp}); 0.88 (d, 3H, $^3J_{H,H}$ = 6.5 Hz, CH_3^{Dipp}); 0.34 (d, 3H, $^3J_{H,H}$ = 6.8 Hz, CH_3^{Dipp}); 0.20 (d, 3H, $^3J_{H,H}$ = 6.7 Hz, CH_3^{Dipp}); -0.53 (s, 3H, AlCH_3); -0.62 (s, 3H, AlCH_3); -0.68 (s, 3H, AlCH_3); -1.23 (s, 3H, AlCH_3). ^{13}C NMR (C_6D_6, 100.61 MHz, 295 K) δ: 174.9 (ArC_q^{OMe}); 170.8 (ArC_q^{OMe}); 165.1 (ArC_q^{Gua}); 158.3 (ArC_q^{Prm}); 146.1 (ArC_q^{Dipp}); 146.0 (ArC_q^{Dipp}); 145.3 (ArC_q^{Dipp}); 145.1 (ArC_q^{Dipp}); 141.2 (ArC_q^{Dipp}); 139.6 (ArC_q^{Dipp}); 126.7 (ArCH^{Dipp}); 126.5 (ArCH^{Dipp}); 125.2 (ArCH^{Dipp}); 124.8 (ArCH^{Dipp}); 124.6 (ArCH^{Dipp}); 123.6 (ArCH^{Dipp}); 79.8 (ArCH^{Prm}); 57.2 (O-CH$_3$); 55.1 (O-CH$_3$); 29.4 (CH^{iPr}); 29.1 (CH^{iPr}); 28.9 (CH^{iPr}); 28.8 (CH^{iPr}); 27.9 (CH$_3^{iPr}$); 26.7 (CH$_3^{iPr}$); 26.1 (CH$_3^{iPr}$); 25.5 (CH$_3^{iPr}$); 24.9 (CH$_3^{iPr}$); 24.0 (CH$_3^{iPr}$); 23.0 (CH$_3^{iPr}$); 22.9 (CH$_3^{iPr}$); -8.3 (2x AlCH$_3$); -8.6 (AlCH$_3$); -9.0 (AlCH$_3$).

Preparation and Characterization of **L(AlMeCl)₂**^{6,4}

METHOD A: To a colorless solution of **LH_2** (0.337 g; 0.65 mmol) in Et_2O (15 mL) cooled to -80 C, a 1.0 M solution of Me_2AlCl in hexane (1.30 mL; 1.30 mmol) was added. The reaction mixture was allowed to warm to room temperature and stirred for 24 hours. The volatiles were evaporated under vacuum with the yield of 0.398 g (>99 %) of white crystalline **$L(AlMeCl)_2$**^{6,4}.

METHOD B: To a suspension of **LH(AlMeCl)**^{6} (1.728 g; 2.91 mmol) in Et_2O (40 mL) cooled to -80 C, a 1.0 M solution of Me_2AlCl in hexane (2.91 mL; 2.91 mmol) was added. The reaction mixture was allowed to warm to room

temperature and stirred for 3 hours with gradual dissolving of the solid. The volatiles were evaporated under vacuum with the yield of 1.930 g (>99 %) of white crystalline **$L(AlMeCl)_2$**^{6,4}.

Single crystals suitable for *sc*XRD analyses were obtained by cooling of a saturated solution of **$L(AlMeCl)_2$**^{6,4} in hexane to 7 °C. ^{1}H NMR (C_6D_6, 400.13 MHz, 295 K) δ: 7.09 (d, 1H, $^3J_{H,H}$ = 7.7 Hz, ArH^{Dipp}); 7.03 (d, 1H, ^3J = 7.7 Hz, ArH^{Dipp}); 6.98-6.92 (m, 3H, ArH^{Dipp}); 6.80 (d, 1H, $^3J_{H,H}$ = 7.8 Hz, ArH^{Dipp}); 6.75 (d, 1H, $^3J_{H,H}$ = 7.5 Hz, ArH^{Dipp}); 4.90 (s, 1H, ArH^{Prm}); 3.64 (m, 1H, $^3J_{H,H}$ = 6.7 Hz, CH^{Dipp}); 3.55 (s, 3H, O-CH_3); 3.38 (m, 2H, $^3J_{H,H}$ = 6.8 Hz, CH^{Dipp}); 3.08 (m, 1H, $^3J_{H,H}$ = 6.8 Hz, CH^{Dipp}); 2.71 (s, 3H, O-CH_3); 1.70 (d, 3H, $^3J_{H,H}$ = 6.6 Hz, CH_3^{Dipp}); 1.51 (d, 3H, $^3J_{H,H}$ = 6.6 Hz, CH_3^{Dipp}); 1.35 (d, 3H, $^3J_{H,H}$ = 6.8 Hz, CH_3^{Dipp}); 1.24 (d, 3H, $^3J_{H,H}$ = 6.7 Hz, CH_3^{Dipp}); 1.21 (d, 3H, $^3J_{H,H}$ = 6.8 Hz, CH_3^{Dipp}); 1.00 (d, 3H, $^3J_{H,H}$ = 6.8 Hz, CH_3^{Dipp}); 0.61 (d, 3H, $^3J_{H,H}$ = 6.8 Hz, CH_3^{Dipp}); 0.46 (d, 3H, $^3J_{H,H}$ = 6.8 Hz, CH_3^{Dipp}); 0.04 (s, 3H, AlCH_3); -0.53 (s, 3H, AlCH_3). ^{13}C NMR (C_6D_6, 100.61 MHz, 295 K) δ: 174.4 (ArC_q^{OMe}); 169.3 (ArC_q^{OMe}); 166.0 (ArC_q^{Gua}); 157.2 (ArC_q^{Prm}); 146.5 (ArC_q^{Dipp}); 146.0 (ArC_q^{Dipp}); 145.2 (ArC_q^{Dipp}); 144.8 (ArC_q^{Dipp}); 138.5 (ArC_q^{Dipp}); 136.9 (ArC_q^{Dipp}); 127.9 (ArCH^{Dipp}); 127.3 (ArCH^{Dipp}); 125.3 (ArCH^{Dipp}); 125.0 (2x ArCH^{Dipp}); 123.7 (ArCH^{Dipp}); 80.3 (ArCH^{Prm}); 56.3 (O-CH_3); 55.2 (O-CH_3); 29.4 (2x CH^{Dipp}); 28.8 (CH^{Dipp}); 28.8 (CH^{Dipp}); 28.2 (CH_3^{Dipp}); 28.0 (CH_3^{Dipp}); 26.8 (CH_3^{Dipp}); 25.7 (CH_3^{Dipp}); 25.1 (CH_3^{Dipp}); 23.7 (CH_3^{Dipp}); 23.1 (CH_3^{Dipp}); 22.5 (CH_3^{Dipp}); -7.4 (AlCH_3); -9.0 (AlCH_3).

Preparation and Characterization of **$L(BH_2)_2$**^{6,4}

METHOD A: To a colorless solution of **LH_2** (0.824 g; 1.59 mmol) in Et_2O (35 mL) cooled to -80 C, BH_3□Me_2S (1.51 mL; 15.92 mmol) was added. The reaction mixture was allowed to warm to room temperature and stirred for 24 hours with gradual precipitation of a white solid. The solid was separated by filtration and dried under vacuum with the yield of 0.750 g (87 %) of white crystalline **$L(BH_2)_2$**^{6,4}.

METHOD B: To a suspension of **$LH(BH_2)$**^{6} (0.964 g; 1.82 mmol) in Et_2O (40 mL) cooled to -80 C, BH_3□Me_2S (0.17 mL; 1.82 mmol) was added. The reaction mixture was allowed to warm to room temperature and stirred for 24 hours. The solid was separated by filtration and dried under vacuum with the yield of 0.680 g (69 %) of white crystalline **$L(BH_2)_2$**^{6,4}.

Single crystals suitable for *sc*XRD analyses were obtained by cooling of a saturated solution of **$L(BH_2)_2$**^{6,4} in Et_2O to 7 °C. ^{1}H NMR (C_6D_6, 500.20 MHz, 295 K) δ: 7.18–7.15 (m, 1H, ArH^{Dipp}); 7.04 (t, 1H, $^3J_{H,H}$ = 7.7 Hz, ArH^{Dipp}); 7.00–6.94 (m, 2H, ArH^{Dipp}); 6.84–6.80 (m, 2H, ArH^{Dipp}); 4.99 (s, 1H, ArH^{Prm}); 4.02 (br s, 2H, BH_2); 3.85 (m, 1H, $^3J_{H,H}$ = 6.7 Hz, CH^{Dipp}); 3.67 (s, 3H, O-CH_3); 3.48 (br s, 2H, BH_2); 3.12 (m, 1H, $^3J_{H,H}$ = 6.7 Hz, CH^{Dipp}); 2.99 (m, 1H, $^3J_{H,H}$ = 6.7 Hz, CH^{Dipp}); 2.78 (m, 1H, $^3J_{H,H}$ = 6.7 Hz, CH^{Dipp}); 2.60 (s, 3H, O-CH_3); 1.63 (d, 3H, $^3J_{H,H}$ = 6.7 Hz, CH_3^{Dipp}); 1.55 (d, 3H, $^3J_{H,H}$ = 6.7 Hz, CH_3^{Dipp}); 1.42 (d, 3H, $^3J_{H,H}$ = 6.7 Hz, CH_3^{Dipp}); 1.34 (d, 3H, $^3J_{H,H}$ = 6.7 Hz, CH_3^{Dipp}); 1.31 (d, 3H, $^3J_{H,H}$ = 6.7 Hz, CH_3^{Dipp}); 0.81 (d, 3H, $^3J_{H,H}$ = 6.8 Hz, CH_3^{Dipp}); 0.42 (d, 6H, $^3J_{H,H}$ = 6.7 Hz, CH_3^{Dipp}). ^{13}C NMR (C_6D_6, 125.78 MHz, 295 K) δ: 172.3 (ArC_q^{OMe}); 166.4 (ArC_q^{OMe}); 163.5 (ArC_q^{Gua}); 155.2 (ArC_q^{Prm}); 147.5 (ArC_q^{Dipp}); 147.0 (ArC_q^{Dipp}); 145.1 (ArC_q^{Dipp}); 144.4 (ArC_q^{Dipp}); 144.1 (ArC_q^{Dipp}); 142.6 (ArC_q^{Dipp}); 128.1 (ArCH^{Dipp}); 127.7 (ArCH^{Dipp}); 125.3 (ArCH^{Dipp}); 125.1 (ArCH^{Dipp}); 124.6 (ArCH^{Dipp}); 124.3 (ArCH^{Dipp}); 82.4 (ArCH^{Prm}); 56.3 (O-CH_3); 55.6 (O-CH_3); 29.7 (CH^{Dipp}); 29.6 (CH^{Dipp}); 29.5 (CH^{Dipp}); 29.2 (CH^{Dipp}); 28.0 (CH_3^{Dipp}); 26.2 (CH_3^{Dipp}); 25.9 (CH_3^{Dipp}); 25.8 (CH_3^{Dipp}); 24.4 (CH_3^{Dipp}); 24.0 (CH_3^{Dipp}); 22.7 (CH_3^{Dipp}); 22.0 (CH_3^{Dipp}). ^{11}B NMR (C_6D_6, 160.48 MHz, 295 K) δ: -8.9 (br s, BH_2); -11.5 (br s, BH_2).

Preparation and Characterization of **L(AlMe$_2$)^{6}(AlMeI)^{4}**

To a colorless solution of **L(AlMe$_2$)$_2$**^{6,4} (1.226 g; 1.95 mmol) in Et_2O (30 mL) cooled to -80 C, a solution of I_2 (0.495 g; 1.95 mmol) in Et_2O (20 mL) was added. The reaction mixture was allowed to warm to room temperature and stirred for 24 hours with gradual precipitation of a solid. The volatiles were evaporated under vacuum with the yield of 1.429 g (>99 %) of white crystalline **L(AlMe$_2$)^{6}(AlMeI)^{4}**. Single crystals suitable for *sc*XRD analyses were obtained from a saturated solution of **L(AlMe$_2$)^{6}(AlMeI)^{4}** in Et_2O at room temperature. ^{1}H NMR (C_6D_6, 500.20 MHz, 295 K) δ: 7.10 (d, 1H, $^{3}J_{H,H}$ = 7.7 Hz, Ar*H*^{Dipp}); 7.04 (d, 1H, $^{3}J_{H,H}$ = 7.7 Hz, Ar*H*^{Dipp}); 6.99–6.93 (m, 2H, Ar*H*^{Dipp}); 6.80 (d, 1H, $^{3}J_{H,H}$ = 7.7 Hz, Ar*H*^{Dipp}); 6.77 (d, 1H, $^{3}J_{H,H}$ = 7.8 Hz, Ar*H*^{Dipp}); 4.96 (s, 1H, Ar*H*^{Prm}); 3.75 (s, 3H, O-C*H*$_3$); 3.51 (m, 1H, $^{3}J_{H,H}$ = 6.5 Hz, C*H*^{Dipp}); 3.49 (m, 1H, $^{3}J_{H,H}$ = 6.5 Hz, C*H*^{Dipp}); 3.17 (m, 1H, $^{3}J_{H,H}$ = 6.7 Hz, C*H*^{Dipp}); 3.11 (m, 1H, $^{3}J_{H,H}$ = 6.7 Hz, C*H*^{Dipp}); 2.67 (s, 3H, O-C*H*$_3$); 1.60–1.55 (m, 6H, C*H*$_3^{Dipp}$); 1.27 (t, 6H, $^{3}J_{H,H}$ = 6.9 Hz, C*H*$_3^{Dipp}$); 1.21 (d, 3H, $^{3}J_{H,H}$ = 6.6 Hz, C*H*$_3^{Dipp}$); 1.08 (d, 3H, $^{3}J_{H,H}$ = 6.7 Hz, C*H*$_3^{Dipp}$); 0.62 (d, 3H, $^{3}J_{H,H}$ = 6.7 Hz, C*H*$_3^{Dipp}$); 0.44 (d, 3H, $^{3}J_{H,H}$ = 6.6 Hz, C*H*$_3^{Dipp}$); 0.26 (s, 3H, AlC*H*$_3$); -0.16 (s, 3H, AlC*H*$_3$); -0.72 (s, 3H, AlC*H*$_3$). ^{13}C NMR (C_6D_6, 125.78 MHz, 295 K) δ: 174.1 (Ar*C*$_q^{OMe}$); 169.6 (Ar*C*$_q^{OMe}$); 165.5 (Ar*C*$_q^{Gua}$); 157.6 (Ar*C*$_q^{Prm}$); 146.8 (Ar*C*$_q^{Dipp}$); 145.4 (Ar*C*$_q^{Dipp}$); 145.2 (Ar*C*$_q^{Dipp}$); 144.3 (Ar*C*$_q^{Dipp}$); 139.8 (Ar*C*$_q^{Dipp}$); 137.1 (Ar*C*$_q^{Dipp}$); 127.2 (2x Ar*C*H^{Dipp}); 125.1 (Ar*C*H^{Dipp}); 125.0 (Ar*C*H^{Dipp}); 124.6 (Ar*C*H^{Dipp}); 124.1 (Ar*C*H^{Dipp}); 80.4 (Ar*C*H^{Prm}); 56.0 (O-*C*H$_3$); 55.6 (O-*C*H$_3$); 30.1 (*C*H$_3^{Dipp}$); 29.7 (*C*H^{Dipp}); 29.2 (*C*H^{Dipp}); 28.7 (*C*H^{Dipp}); 28.6 (*C*H^{Dipp}); 28.3 (*C*H$_3^{Dipp}$); 26.3 (*C*H$_3^{Dipp}$); 25.2 (*C*H$_3^{Dipp}$); 25.1 (*C*H$_3^{Dipp}$); 24.0 (*C*H$_3^{Dipp}$); 23.9 (*C*H$_3^{Dipp}$); 22.6 (*C*H$_3^{Dipp}$); -2.6 (Al*C*H$_3$); -7.8 (2x Al*C*H$_3$).

Preparation and Characterization of **L(AlMeI)^{6}(AlMe$_2$)^{4}**

To a colorless solution of **LH(AlMeI)**^{6} (0.776 g; 1.12 mmol) in CPME (30 mL) cooled to -80 C, a 2.0 M solution of Me_3Al in hexane (1.12 mL; 2.14 mmol) was added. The reaction mixture was allowed to warm to room temperature and stirred for 24 hours with gradual precipitation of a white solid. The volatiles were evaporated under vacuum, the crude mixture was washed with hexane (15 mL) and dried under vacuum with the yield of 0.719 g (87 %) of white **L(AlMeI)^{6}(AlMe$_2$)^{4}**. ^{1}H NMR (C_6D_6, 500.20 MHz, 295 K) δ: 7.17–7.13 (m, 1H, Ar*H*^{Dipp}); 6.99–6.92 (m, 3H, Ar*H*^{Dipp}); 6.82 (d, 1H, $^{3}J_{H,H}$ = 7.4 Hz, Ar*H*^{Dipp}); 6.74 (d, 1H, $^{3}J_{H,H}$ = 7.6 Hz, Ar*H*^{Dipp}); 4.88 (s, 1H, Ar*H*^{Prm}); 3.84 (m, 1H, $^{3}J_{H,H}$ = 6.6 Hz, C*H*^{Dipp}); 3.49 (s, 3H, O-C*H*$_3$); 3.38 (m, 1H, $^{3}J_{H,H}$ = 6.6 Hz, C*H*^{Dipp}); 3.14 (m, 1H, $^{3}J_{H,H}$ = 6.7 Hz, C*H*^{Dipp}); 2.90 (m, 1H, $^{3}J_{H,H}$ = 6.6 Hz, C*H*^{Dipp}); 2.70 (s, 3H, O-C*H*$_3$); 1.76 (d, 3H, $^{3}J_{H,H}$ = 6.6 Hz, C*H*$_3^{Dipp}$); 1.41–1.38 (m, 6H, C*H*$_3^{Dipp}$); 1.36 (d, 3H, $^{3}J_{H,H}$ = 6.6 Hz, C*H*$_3^{Dipp}$); 1.07 (d, 3H, $^{3}J_{H,H}$ = 6.6 Hz, C*H*$_3^{Dipp}$); 0.98 (d, 3H, $^{3}J_{H,H}$ = 6.6 Hz, C*H*$_3^{Dipp}$); 0.57 (d, 3H, $^{3}J_{H,H}$ = 6.7 Hz, C*H*$_3^{Dipp}$); 0.47 (d, 3H, $^{3}J_{H,H}$ = 6.5 Hz, C*H*$_3^{Dipp}$); -0.04 (s, 3H, AlC*H*$_3$); -0.07 (s, 3H, AlC*H*$_3$); -0.32 (s, 3H, AlC*H*$_3$). ^{13}C NMR (C_6D_6, 125.78 MHz, 295 K) δ: 174.3 (Ar*C*$_q^{OMe}$); 169.0 (Ar*C*$_q^{OMe}$); 163.8 (Ar*C*$_q^{Gua}$); 157.3 (Ar*C*$_q^{Prm}$); 147.1 (Ar*C*$_q^{Dipp}$); 145.2 (Ar*C*$_q^{Dipp}$); 144.9 (Ar*C*$_q^{Dipp}$); 144.7 (Ar*C*$_q^{Dipp}$); 139.0 (Ar*C*$_q^{Dipp}$); 137.6 (Ar*C*$_q^{Dipp}$); 127.6 (Ar*C*H^{Dipp}); 126.9 (Ar*C*H^{Dipp}); 125.5 (Ar*C*H^{Dipp}); 125.3 (Ar*C*H^{Dipp}); 124.6 (Ar*C*H^{Dipp}); 123.4 (Ar*C*H^{Dipp}); 79.9 (Ar*C*H^{Prm}); 56.2 (O-*C*H$_3$); 54.8 (O-*C*H$_3$); 29.2 (*C*H^{Dipp}); 29.0 (*C*H^{Dipp}); 28.8 (*C*H^{Dipp}); 28.7 (*C*H^{Dipp}); 28.2 (*C*H$_3^{Dipp}$); 28.0 (*C*H$_3^{Dipp}$); 27.6 (*C*H$_3^{Dipp}$); 26.0 (*C*H$_3^{Dipp}$); 25.0 (2x *C*H$_3^{Dipp}$); 23.0 (*C*H$_3^{Dipp}$); 22.5 (*C*H$_3^{Dipp}$); -3.6 (Al*C*H$_3$); -8.1 (Al*C*H$_3$); -9.0 (Al*C*H$_3$).

CRYSTALLOGRAPHY

Table S2. Crystal data and structure refinement for **[LH(Li)*[4]*]**$_2$.

Crystal data	
Chemical formula	$C_{62}H_{84}Li_2N_{10}O_4$
M_r	1047.27
Crystal system, space group	Monoclinic, $P2_1$
Temperature (K)	150
a, *b*, *c* (Å)	11.4047(4), 37.9001(12), 14.1637(5)
β (°)	100.759(1)
V (Å^3)	6014.5(4)
Z	4
Radiation type	Mo*K*α
μ (mm^{-1})	0.07
Crystal size (mm)	0.19 × 0.10 × 0.03
Data collection	
Diffractometer	Bruker D8 - Venture
Absorption correction	Multi-scan<br>*SADABS2016*/2 - Bruker AXS area detector scaling and absorption correction
T_{min}, T_{max}	0.708, 0.745
No. of measured, independent and observed [$I > 2\sigma(I)$] reflections	142199, 24676, 15748
R_{int}	0.120
$(\sin \theta/\lambda)_{max}$ (Å^{-1})	0.625
Refinement	
$R[F^2 > 2\sigma(F^2)]$, $wR(F^2)$, S	0.056, 0.154, 1.01
No. of reflections	24676
No. of parameters	1456
No. of restraints	1496
H-atom treatment	H atoms treated by a mixture of independent and constrained refinement
$\Delta\rho_{max}$, $\Delta\rho_{min}$ (e Å^{-3})	0.73, −0.35

Computer programs: Bruker Instrument Service vV6.2.3, *APEX4* v2022.10-0 (Bruker AXS), *SAINT* V8.37A (Bruker AXS Inc., 2015), XT, VERSION 2014/5, *SHELXL2019*/1 (Sheldrick, 2019), *PLATON* (Spek, 2009).

Table S3. Crystal data and structure refinement for **LH(AlMe$_2$)**[6].

Crystal data	
Chemical formula	$C_{33}H_{48}AlN_5O_2$
M_r	573.74
Crystal system, space group	Triclinic, *P*-1
Temperature (K)	150
a, *b*, *c* (Å)	10.8010(5), 12.2087(6), 12.9314(6)
α, β, γ (°)	74.741(2), 84.050(2), 76.154(2)
V (Å^3)	1595.79(13)
Z	2
Radiation type	Mo*K*α
μ (mm^{-1})	0.10
Crystal size (mm)	0.59 × 0.29 × 0.12
Data collection	
Diffractometer	Bruker D8 - Venture
Absorption correction	Multi-scan<br>*SADABS2016*/2 - Bruker AXS area detector scaling and absorption correction
T_{min}, T_{max}	0.705, 0.746
No. of measured, independent and observed [$I > 2\sigma(I)$] reflections	52164, 7366, 5808
R_{int}	0.065
$(\sin \theta/\lambda)_{max}$ (Å^{-1})	0.652
Refinement	
$R[F^2 > 2\sigma(F^2)]$, $wR(F^2)$, S	0.048, 0.114, 1.04
No. of reflections	7366
No. of parameters	391
No. of restraints	341
H-atom treatment	H atoms treated by a mixture of independent and constrained refinement
$\Delta\rho_{max}$, $\Delta\rho_{min}$ (e Å^{-3})	0.30, −0.40

Computer programs: Bruker Instrument Service vV6.2.3, *APEX3* v2016.5-0 (Bruker AXS), *SAINT* V8.37A (Bruker AXS Inc., 2015), XT, VERSION 2014/5, *SHELXL2019*/1 (Sheldrick, 2019), *PLATON* (Spek, 2009).

Table S4. Crystal data and structure refinement for **LH(AlMeI)**[6].

Crystal data	
Chemical formula	$C_{32}H_{45}AlIN_5O_2{\cdot}C_7H_8$
M_r	777.74
Crystal system, space group	Triclinic, *P*-1
Temperature (K)	220
a, *b*, *c* (Å)	10.9226(4), 13.6190(6), 13.7046(6)
α, β, γ (°)	100.901(2), 95.140(2), 91.009(2)
V ($Å^3$)	1992.54(14)
Z	2
Radiation type	Mo*K*α
μ (mm^{-1})	0.86
Crystal size (mm)	0.37 × 0.23 × 0.18
**Data collection**	
Diffractometer	Bruker D8 - Venture
Absorption correction	Multi-scan<br>*SADABS2016*/2 - Bruker AXS area detector scaling and absorption correction
T_{min}, T_{max}	0.681, 0.746
No. of measured, independent and observed [$I > 2\sigma(I)$] reflections	64901, 9199, 7246
R_{int}	0.059
$(\sin \theta/\lambda)_{max}$ ($Å^{-1}$)	0.651
**Refinement**	
$R[F^2 > 2\sigma(F^2)]$, $wR(F^2)$, S	0.039, 0.086, 1.03
No. of reflections	9199
No. of parameters	437
No. of restraints	426
H-atom treatment	H atoms treated by a mixture of independent and constrained refinement
$\Delta\rho_{max}$, $\Delta\rho_{min}$ (e $Å^{-3}$)	0.48, −0.79

Computer programs: Bruker Instrument Service vV6.2.3, *APEX3* v2016.5-0 (Bruker AXS), *SAINT* V8.37A (Bruker AXS Inc., 2015), XT, VERSION 2014/5, *SHELXL2019*/1 (Sheldrick, 2019), *PLATON* (Spek, 2009).

Table S5. Crystal data and structure refinement for **LH(AlI$_2$)**[6].

Crystal data	
Chemical formula	$0.56(C_{31}H_{42}AlI_2N_5O_2)\cdot 0.44(C_{32}H_{45}AlIN_5O_2)$
M_r	748.25
Crystal system, space group	Triclinic, *P*-1
Temperature (K)	150
a, *b*, *c* (Å)	10.7757(9), 12.5025(11), 13.0002(12)
α, β, γ (°)	75.088(4), 84.481(4), 75.469(4)
V (Å^3)	1637.4(3)
Z	2
Radiation type	Mo*K*α
μ (mm^{-1})	1.57
Crystal size (mm)	0.42 × 0.16 × 0.12
Data collection	
Diffractometer	Bruker D8 - Venture
Absorption correction	Multi-scan<br>*SADABS2016*/2 - Bruker AXS area detector scaling and absorption correction
T_{min}, T_{max}	0.603, 0.746
No. of measured, independent and observed [$I > 2\sigma(I)$] reflections	57968, 7528, 6770
R_{int}	0.044
$(\sin \theta/\lambda)_{max}$ (Å^{-1})	0.652
Refinement	
$R[F^2 > 2\sigma(F^2)]$, $wR(F^2)$, S	0.033, 0.075, 1.22
No. of reflections	7528
No. of parameters	417
No. of restraints	365
H-atom treatment	H-atom parameters constrained
$\Delta\rho_{max}$, $\Delta\rho_{min}$ (e Å^{-3})	0.66, −0.84

Computer programs: Bruker Instrument Service vV6.2.3, *APEX3* v2016.5-0 (Bruker AXS), *SAINT* V8.37A (Bruker AXS Inc., 2015), XT, VERSION 2014/5, *SHELXL2019*/1 (Sheldrick, 2019), *PLATON* (Spek, 2009).

Table S6. Crystal data and structure refinement for **LH(AlMeCl)**[6].

Crystal data	
Chemical formula	$C_{32}H_{45}AlClN_5O_2$
M_r	594.16
Crystal system, space group	Triclinic, *P*-1
Temperature (K)	150
a, *b*, *c* (Å)	10.7717(6), 12.1952(6), 12.9800(7)
α, β, γ (°)	74.612(2), 83.695(2), 75.627(2)
V (Å^3)	1590.75(15)
Z	2
Radiation type	Mo*K*α
μ (mm^{-1})	0.18
Crystal size (mm)	0.59 × 0.42 × 0.36
Data collection	
Diffractometer	Bruker D8 - Venture
Absorption correction	Multi-scan *SADABS2016*/2 - Bruker AXS area detector scaling and absorption correction
T_{min}, T_{max}	0.676, 0.746
No. of measured, independent and observed [$I > 2\sigma(I)$] reflections	59001, 7352, 6175
R_{int}	0.058
$(\sin \theta/\lambda)_{max}$ (Å^{-1})	0.652
Refinement	
$R[F^2 > 2\sigma(F^2)]$, $wR(F^2)$, S	0.054, 0.139, 1.04
No. of reflections	7352
No. of parameters	381
No. of restraints	375
H-atom treatment	H atoms treated by a mixture of independent and constrained refinement
$\Delta\rho_{max}$, $\Delta\rho_{min}$ (e Å^{-3})	0.91, −0.97

Computer programs: Bruker Instrument Service vV6.2.3, *APEX3* v2016.5-0 (Bruker AXS), *SAINT* V8.37A (Bruker AXS Inc., 2015), XT, VERSION 2014/5, *SHELXL2014*/7 (Sheldrick, 2014), *PLATON* (Spek, 2009).

Table S7. Crystal data and structure refinement for **LH(AlCl$_2$)**[6].

Crystal data	
Chemical formula	$C_{31}H_{42}AlCl_2N_5O_2·2(C_4H_{10}O)$
M_r	762.81
Crystal system, space group	Triclinic, *P*-1
Temperature (K)	150
a, *b*, *c* (Å)	10.7168(4), 11.7253(4), 16.8353(6)
α, β, γ (°)	79.014(1), 76.995(2), 66.504(1)
V (Å^3)	1878.25(12)
Z	2
Radiation type	Mo*K*α
μ (mm^{-1})	0.25
Crystal size (mm)	0.59 × 0.33 × 0.19
Data collection	
Diffractometer	Bruker D8 - Venture
Absorption correction	Multi-scan<br>*SADABS2016*/2 - Bruker AXS area detector scaling and absorption correction
T_{min}, T_{max}	0.705, 0.746
No. of measured, independent and observed [$I > 2\sigma(I)$] reflections	57597, 8636, 7154
R_{int}	0.041
$(\sin \theta/\lambda)_{max}$ (Å^{-1})	0.652
Refinement	
$R[F^2 > 2\sigma(F^2)]$, $wR(F^2)$, S	0.066, 0.172, 1.05
No. of reflections	8636
No. of parameters	383
No. of restraints	345
H-atom treatment	H atoms treated by a mixture of independent and constrained refinement
$\Delta\rho_{max}$, $\Delta\rho_{min}$ (e Å^{-3})	1.31, −1.51

Computer programs: Bruker Instrument Service vV6.2.3, *APEX3* v2016.5-0 (Bruker AXS), *SAINT* V8.37A (Bruker AXS Inc., 2015), XT, VERSION 2014/5, *SHELXL2019*/1 (Sheldrick, 2019), *PLATON* (Spek, 2009).

Table S8. Crystal data and structure refinement for **LH(BH$_2$)**[6].

Crystal data	
Chemical formula	$C_{31}H_{44}BN_5O_2$
M_r	529.52
Crystal system, space group	Monoclinic, $P2_1/n$
Temperature (K)	150
a, *b*, *c* (Å)	10.5881(3), 15.4172(5), 18.3764(7)
β (°)	101.921(2)
V (Å^3)	2935.05(17)
Z	4
Radiation type	Mo*K*α
μ (mm^{-1})	0.08
Crystal size (mm)	0.18 × 0.15 × 0.06
Data collection	
Diffractometer	Bruker D8 - Venture
Absorption correction	Multi-scan<br>*SADABS2016*/2 - Bruker AXS area detector scaling and absorption correction
T_{min}, T_{max}	0.635, 0.727
No. of measured, independent and observed [$I > 2\sigma(I)$] reflections	84298, 5745, 4311
R_{int}	0.119
$(\sin \theta/\lambda)_{max}$ (Å^{-1})	0.617
Refinement	
$R[F^2 > 2\sigma(F^2)]$, $wR(F^2)$, S	0.045, 0.116, 0.95
No. of reflections	5745
No. of parameters	371
No. of restraints	309
H-atom treatment	H atoms treated by a mixture of independent and constrained refinement
$\Delta\rho_{max}$, $\Delta\rho_{min}$ (e Å^{-3})	0.21, −0.21

Computer programs: Bruker Instrument Service vV6.2.3, *APEX4* v2022.10-0 (Bruker AXS), *SAINT* V8.37A (Bruker AXS Inc., 2015), XT, VERSION 2014/5, *SHELXL2019*/1 (Sheldrick, 2019), *PLATON* (Spek, 2009).

Table S9. Crystal data and structure refinement for **LH(BF$_2$)**[6].

Crystal data	
Chemical formula	$C_{31}H_{42}BF_2N_5O_2 \cdot C_4H_8O$
M_r	637.61
Crystal system, space group	Monoclinic, $P2_1/n$
Temperature (K)	150
a, *b*, *c* (Å)	10.0942(3), 18.5777(7), 18.8141(7)
β (°)	90.808(1)
V (Å^3)	3527.8(2)
Z	4
Radiation type	Mo*K*α
μ (mm^{-1})	0.08
Crystal size (mm)	0.46 × 0.09 × 0.07
Data collection	
Diffractometer	Bruker D8 - Venture
Absorption correction	Multi-scan<br>*SADABS2016*/2 - Bruker AXS area detector scaling and absorption correction[2]
T_{min}, T_{max}	0.701, 0.746
No. of measured, independent and observed [$I > 2\sigma(I)$] reflections	159368, 8762, 6765
R_{int}	0.096
$(\sin \theta/\lambda)_{max}$ (Å^{-1})	0.667
Refinement	
$R[F^2 > 2\sigma(F^2)]$, $wR(F^2)$, S	0.043, 0.114, 1.02
No. of reflections	8762
No. of parameters	428
No. of restraints	360
H-atom treatment	H atoms treated by a mixture of independent and constrained refinement
$\Delta\rho_{max}$, $\Delta\rho_{min}$ (e Å^{-3})	0.23, −0.22

Hydrogen bond geometry

D—H···*A*	*D*—H	H···*A*	*D*···*A*	*D*—H···*A*
N2—H2···O3	0.871(16)	2.130(16)	2.8700(15)	142.4(14)

Computer programs: Bruker Instrument Service vV6.2.3, *APEX4* v2022.10-0 (Bruker AXS), *SAINT* V8.37A (Bruker AXS Inc., 2015), XT, VERSION 2014/5, *SHELXL2019*/1 (Sheldrick, 2019), *PLATON* (Spek, 2009).

2 Krause, L.; Herbst-Irmer, R.; Sheldrick, G. M.; Stalke, D. *J. Appl. Crystallogr.* **2015**, *48*, 3–10. Comparison of silver and molybdenum microfocus X-ray sources for single-crystal structure determination.DOI:10.1107/S1600576714022985.

Table S10. Crystal data and structure refinement for **L(AlMe$_2$)$_2$**^{4,4}.

Crystal data	
Chemical formula	$C_{35}H_{53}Al_2N_5O_2$
M_r	629.78
Crystal system, space group	Monoclinic, $P2_1/n$
Temperature (K)	150
a, *b*, *c* (Å)	10.7691(12), 20.311(3), 16.993(2)
β (°)	90.809(4)
V (Å^3)	3716.5(8)
Z	4
Radiation type	Mo*K*α
μ (mm^{-1})	0.11
Crystal size (mm)	0.59 × 0.51 × 0.23
Data collection	
Diffractometer	Bruker D8 - Venture
Absorption correction	Multi-scan<br>*SADABS2016*/2 - Bruker AXS area detector scaling and absorption correction
T_{min}, T_{max}	0.645, 0.746
No. of measured, independent and observed [$I > 2\sigma(I)$] reflections	101584, 9237, 6747
R_{int}	0.090
$(\sin \theta/\lambda)_{max}$ (Å^{-1})	0.668
Refinement	
$R[F^2 > 2\sigma(F^2)]$, $wR(F^2)$, S	0.052, 0.159, 1.10
No. of reflections	9237
No. of parameters	411
No. of restraints	378
H-atom treatment	H-atom parameters constrained
$\Delta\rho_{max}$, $\Delta\rho_{min}$ (e Å^{-3})	0.43, −0.37

Computer programs: Bruker Instrument Service vV6.2.3, *APEX4* v2022.10-0 (Bruker AXS), *SAINT* V8.37A (Bruker AXS Inc., 2015), XT, VERSION 2014/5, *SHELXL2019*/1 (Sheldrick, 2019), *PLATON* (Spek, 2009).

Table S11. Crystal data and structure refinement **L(AlMeCl)$_2$**^{4,4}.

Crystal data	
Chemical formula	$C_{33}H_{47}Al_2Cl_2N_5O_2$
M_r	670.61
Crystal system, space group	Monoclinic, $P2_1/n$
Temperature (K)	150
a, *b*, *c* (Å)	10.7714(6), 20.2924(11), 17.0384(17)
β (°)	90.346(4)
V (Å^3)	3724.1(5)
Z	4
Radiation type	Mo*K*α
μ (mm^{-1})	0.26
Crystal size (mm)	0.59 × 0.09 × 0.06
Data collection	
Diffractometer	Bruker D8 - Venture
Absorption correction	Multi-scan<br>*SADABS2016*/2 - Bruker AXS area detector scaling and absorption correction
T_{min}, T_{max}	0.577, 0.746
No. of measured, independent and observed [$I > 2\sigma(I)$] reflections	50865, 8500, 7492
R_{int}	0.044
$(\sin \theta/\lambda)_{max}$ (Å^{-1})	0.651
Refinement	
$R[F^2 > 2\sigma(F^2)]$, $wR(F^2)$, S	0.087, 0.215, 1.07
No. of reflections	8500
No. of parameters	410
No. of restraints	405
H-atom treatment	H-atom parameters constrained
	$w = 1/[\sigma^2(F_o^2) + (0.0792P)^2 + 13.0715P]$<br>where $P = (F_o^2 + 2F_c^2)/3$
$\Delta\rho_{max}$, $\Delta\rho_{min}$ (e Å^{-3})	1.12, −1.60

Computer programs: Bruker Instrument Service vV6.2.3, *APEX3* v2016.5-0 (Bruker AXS), *SAINT* V8.37A (Bruker AXS Inc., 2015), XT, VERSION 2014/5, *SHELXL2019*/1 (Sheldrick, 2019), *PLATON* (Spek, 2009).

Table S12. Crystal data and structure refinement for **$L(AlMe_2)_2$**[6,4].

Crystal data	
Chemical formula	$C_{35}H_{53}Al_2N_5O_2$
M_r	629.78
Crystal system, space group	Orthorhombic, $P2_12_12_1$
Temperature (K)	150
a, *b*, *c* (Å)	11.4942(4), 15.8542(4), 20.0510(5)
V (Å^3)	3653.92(18)
Z	4
Radiation type	Mo*K*α
μ (mm^{-1})	0.12
Crystal size (mm)	0.44 × 0.29 × 0.28
Data collection	
Diffractometer	Bruker D8 - Venture
Absorption correction	Multi-scan<br>*SADABS2016*/2 - Bruker AXS area detector scaling and absorption correction
T_{min}, T_{max}	0.714, 0.746
No. of measured, independent and observed [$I > 2\sigma(I)$] reflections	86970, 9044, 8212
R_{int}	0.061
$(\sin \theta/\lambda)_{max}$ (Å^{-1})	0.667
Refinement	
$R[F^2 > 2\sigma(F^2)]$, $wR(F^2)$, S	0.032, 0.084, 1.05
No. of reflections	9044
No. of parameters	411
No. of restraints	375
H-atom treatment	H-atom parameters constrained
$\Delta\rho_{max}$, $\Delta\rho_{min}$ (e Å^{-3})	0.18, −0.17
Absolute structure	Flack x determined using 3467 quotients [(I+)-(I-)]/[(I+)+(I-)] (Parsons, Flack and Wagner, Acta Cryst. B69 (2013) 249-259).
Absolute structure parameter	0.06(3)

Computer programs: Bruker Instrument Service vV6.2.3, *APEX4* v2022.10-0 (Bruker AXS), *SAINT* V8.37A (Bruker AXS Inc., 2015), XT, VERSION 2014/5, *SHELXL2019*/1 (Sheldrick, 2019), *PLATON* (Spek, 2009).

Table S13. Crystal data and structure refinement for **L(BH₂)₂**[6,4].

Crystal data	
Chemical formula	$C_{31}H_{45}B_2N_5O_2$
M_r	541.34
Crystal system, space group	Monoclinic, $P2_1/c$
Temperature (K)	150
a, *b*, *c* (Å)	17.393(3), 15.651(3), 11.3946(19)
β (°)	91.021(7)
V (Å^3)	3101.2(9)
Z	4
Radiation type	Mo*K*α
μ (mm^{-1})	0.07
Crystal size (mm)	0.59 × 0.33 × 0.17
Data collection	
Diffractometer	Bruker D8 - Venture
Absorption correction	Multi-scan<br>*SADABS2016*/2 - Bruker AXS area detector scaling and absorption correction
T_{min}, T_{max}	0.315, 0.709
No. of measured, independent and observed [$I > 2\sigma(I)$] reflections	58645, 4953, 3193
R_{int}	0.179
$(\sin \theta/\lambda)_{max}$ (Å^{-1})	0.578
Refinement	
$R[F^2 > 2\sigma(F^2)]$, $wR(F^2)$, S	0.054, 0.119, 1.00
No. of reflections	4953
No. of parameters	384
No. of restraints	327
H-atom treatment	H atoms treated by a mixture of independent and constrained refinement
$\Delta\rho_{max}$, $\Delta\rho_{min}$ (e Å^{-3})	0.22, −0.19

Computer programs: Bruker Instrument Service vV6.2.3, *APEX4* v2022.10-0 (Bruker AXS), *SAINT* V8.37A (Bruker AXS Inc., 2015), XT, VERSION 2014/5, *SHELXL2019*/1 (Sheldrick, 2019), *PLATON* (Spek, 2009).

Table S14. Crystal data and structure refinement for **L(AlMe$_2$)^{6}(AlMeI)^{4}**.

Crystal data	
Chemical formula	2($C_{34}H_{50}Al_2IN_5O_2$)·$C_4H_{10}O$
M_r	1557.41
Crystal system, space group	Monoclinic, $P2_1/c$
Temperature (K)	150
a, *b*, *c* (Å)	18.9293(14), 11.8768(9), 35.449(3)
β (°)	96.257(2)
V (Å^3)	7922.1(10)
Z	4
Radiation type	Mo*K*α
μ (mm^{-1})	0.89
Crystal size (mm)	0.59 × 0.38 × 0.07
Data collection	
Diffractometer	Bruker D8 - Venture
Absorption correction	Multi-scan<br>*SADABS2016*/2 - Bruker AXS area detector scaling and absorption correction Reference: Krause, L., Herbst-Irmer, R., Sheldrick G.M. & Stalke D., J. Appl. Cryst. 48 (2015) 3-10.
T_{min}, T_{max}	0.592, 0.746
No. of measured, independent and observed [$I > 2\sigma(I)$] reflections	147854, 18306, 15332
R_{int}	0.048
$(\sin \theta/\lambda)_{max}$ (Å^{-1})	0.652
Refinement	
$R[F^2 > 2\sigma(F^2)]$, $wR(F^2)$, S	0.071, 0.144, 1.21
No. of reflections	18306
No. of parameters	912
No. of restraints	853
H-atom treatment	H-atom parameters constrained
	$w = 1/[\sigma^2(F_o^2) + (0.0216P)^2 + 33.1904P]$<br>where $P = (F_o^2 + 2F_c^2)/3$
$\Delta\rho_{max}$, $\Delta\rho_{min}$ (e Å^{-3})	0.82, −1.37

Computer programs: Bruker Instrument Service vV6.2.3, *APEX4* v2022.10-0 (Bruker AXS), *SAINT* V8.37A (Bruker AXS Inc., 2015), XT, VERSION 2014/5, *SHELXL2019*/1 (Sheldrick, 2019), *PLATON* (Spek, 2009).

THEORY

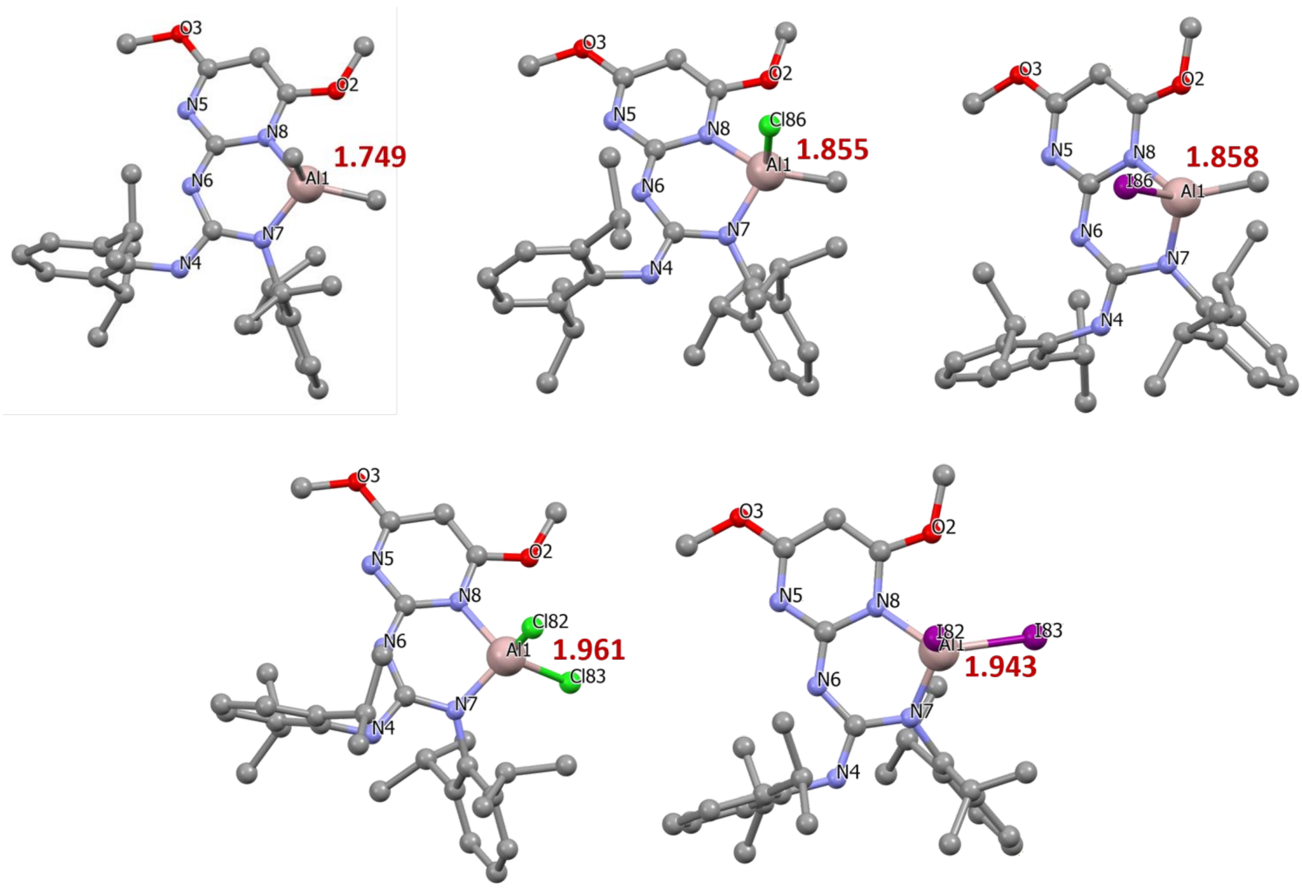

Figure S35. Optimized structures and calculated APT charges for **LH(AlX_2)** [*e*] (B3LYP/D3/CPCM-n-Heptane/def2-TZVP)[3].

3 a) Weigend, F. ; Ahlrichs, R. Balanced basis sets of split valence, triple zeta valence and quadruple zeta valence quality for H to Rn: Design and assessment of accuracy. *Phys. Chem. Chem. Phys.* **2005**, *7*, 3297-3305. doi.org/10.1039/B508541A. b) Zhao, Y.; Truhlar, D. G. The M06 suite of density functionals for main group thermochemistry, thermochemical kinetics, noncovalent interactions, excited states, and transition elements: two new functionals and systematic testing of four M06-class functionals and 12 other functionals. *Theor. Chem. Acc.* **2008**, *120*, 215-241. doi.org/10.1007/s00214-007-0310-x. c) Tomasi, J.; Mennucci, B.; Cammi, R. Quantum mechanical continuum solvation models. *Chem. Rev*. **2005**, *105*, 2999–3093. DOI: 10.1021/cr9904009; d) Grimme, S.; Antony, J.; Ehrlich, S.; Krieg, H. A. Consistent and accurate *ab initio* parametrization of density functional dispersion correction (DFT-D) for the 94 elements H-Pu. *J. Chem. Phys.* **2010**, *132*, 154104–154119. DOI: 10.1063/1.3382344

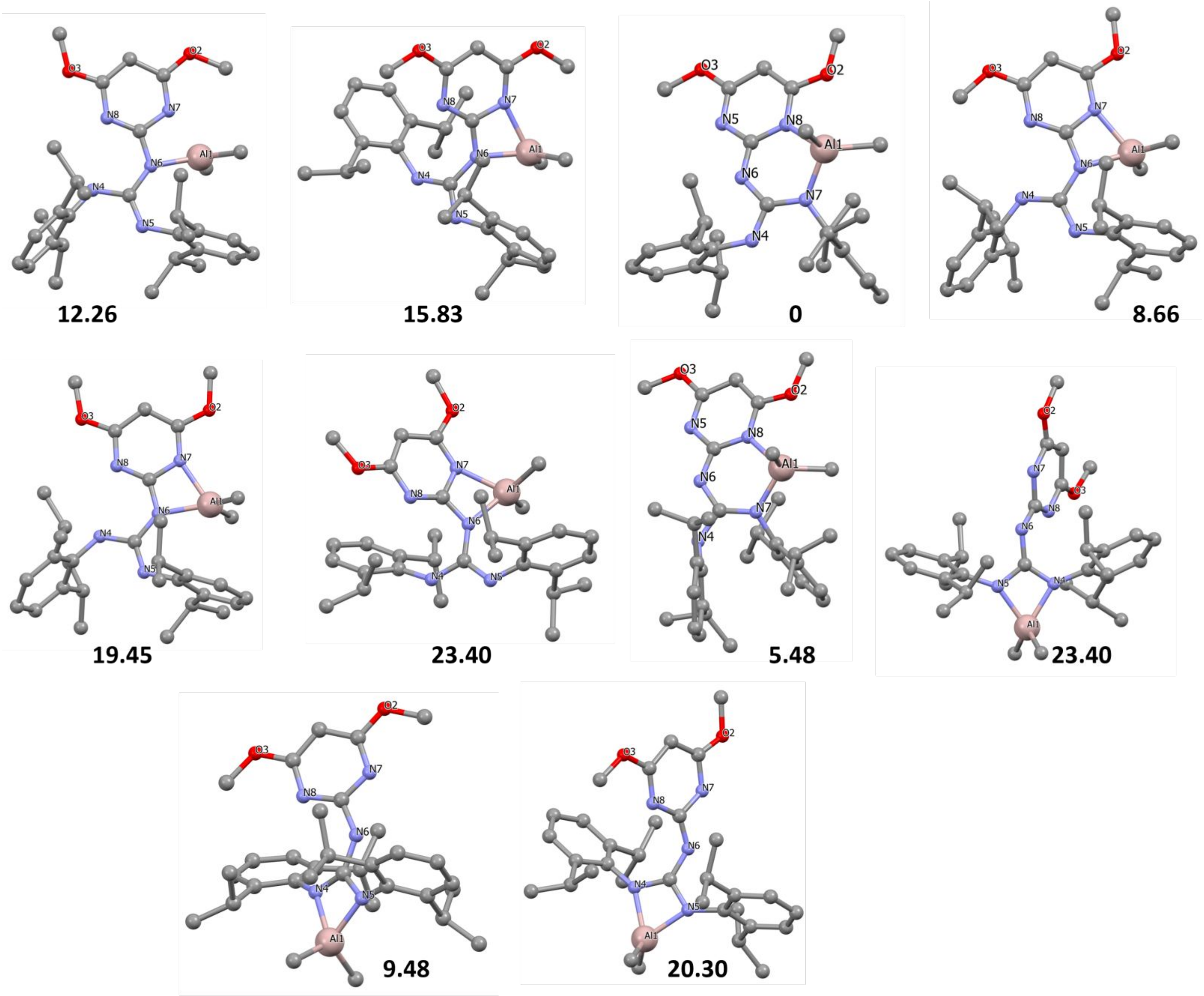

Figure S36. Optimized structures and calculated relative Gibbs' free energies [kcal/mol] (B3LYP/D3/CPCM-n-Heptane/6-311+G(d,p))[3c,3d,4] for **LH(AlMe$_2$)** species.

---

4 a) Becke, A. D. Density-functional thermochemistry. III. The role of exact Exchange. *J. Chem. Phys*. **1993**, *98*, 5648−5652. DOI: 10.1063/1.464913. b) Dunning, T. H. Jr. Gaussian basis sets for use in correlated molecular calculations. I. The atoms boron through neon and hydrogen. *J. Chem. Phys.* **1989**, *90*, 1007−1023. DOI : 10.1063/1.456153.

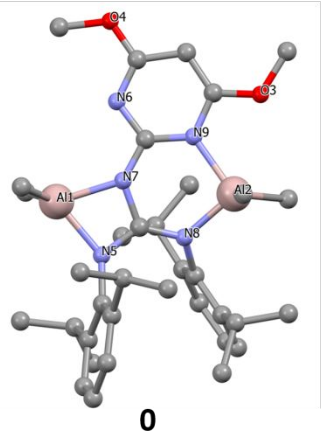

0

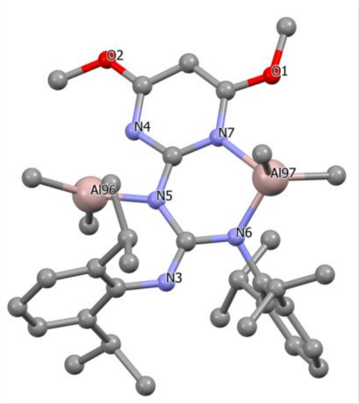

10.04

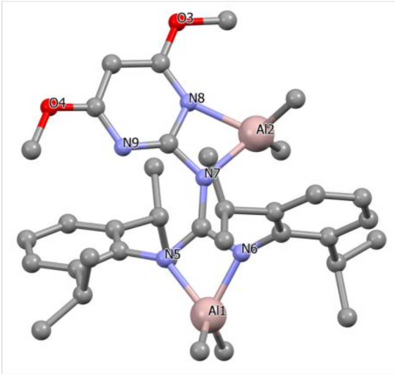

10.08

Figure S37. Optimized structures and calculated relative Gibbs free energies [kcal/mol] (B3LYP/D3/CPCM-n-Heptane/6-311+G(d,p)) [3c,3d,4] for **L(AlMe$_2$)$_2$** species.

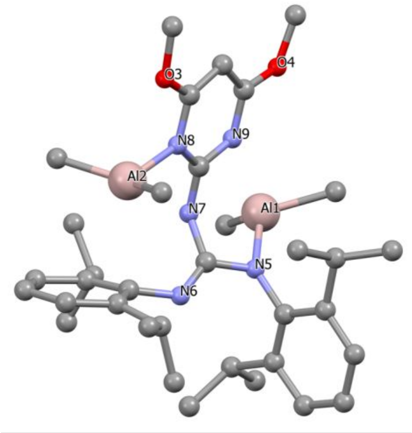

60.32

Figure S38. Optimized structure and calculated relative Gibbs free energy [referred to lowest energy isomer in Figure S37 [kcal/mol] (B3LYP/D3/CPCM-n-Heptane/6-311+G(d,p))[3c,3d,4] for one of transition states of **L(AlMe$_2$)$_2$** species isomerization.

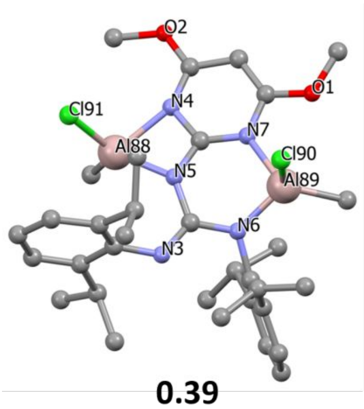

0.39

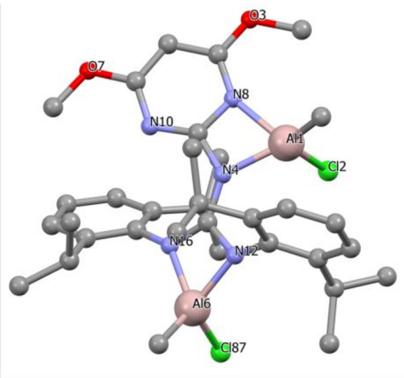

0

Figure S39. Optimized structures and calculated relative Gibbs free energies [kcal/mol] (B3LYP/D3/CPCM-n-Heptane/6-311+G(d,p))[3c,3d,4] for **L(AlMeCl)$_2$** species.

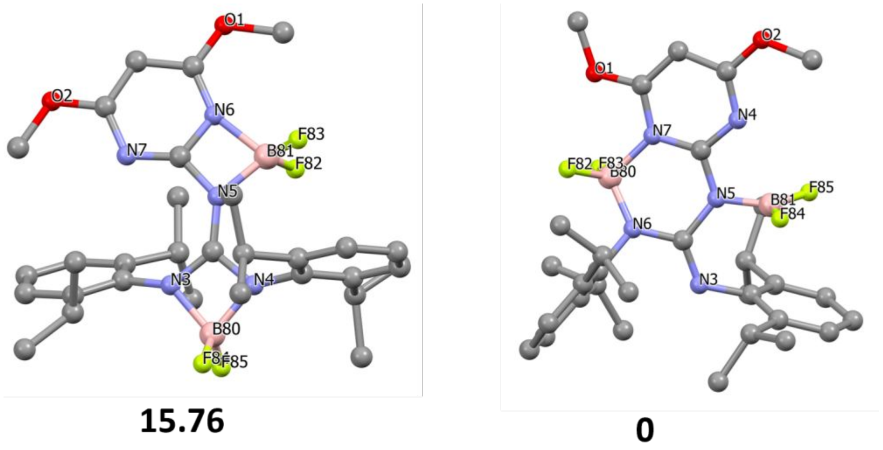

Figure S40. Optimized structures and calculated relative Gibbs free energies [kcal/mol] (B3LYP/D3/CPCM-n-Heptane/6-311+G(d,p))[3c,3d,4] for **L(BF$_2$)$_2$** species.

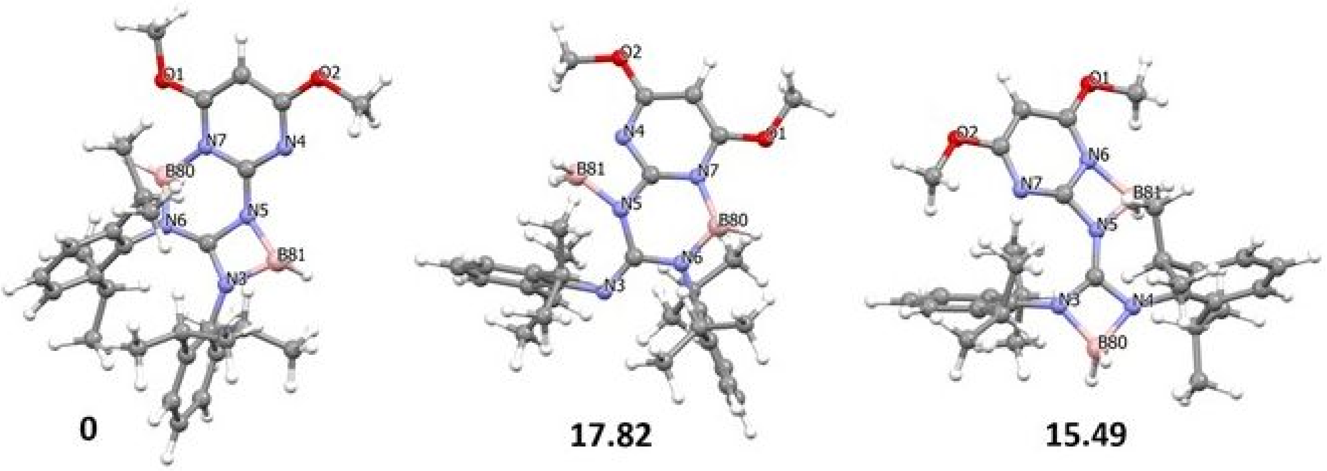

Figure S41. Optimized structures and calculated relative Gibbs free energies [kcal/mol] (B3LYP/D3/CPCM-n-Heptane/6-311+G(d,p))[3c,3d,4] for **L(BH$_2$)$_2$** species.

INFRARED AND RAMAN SPECTRA

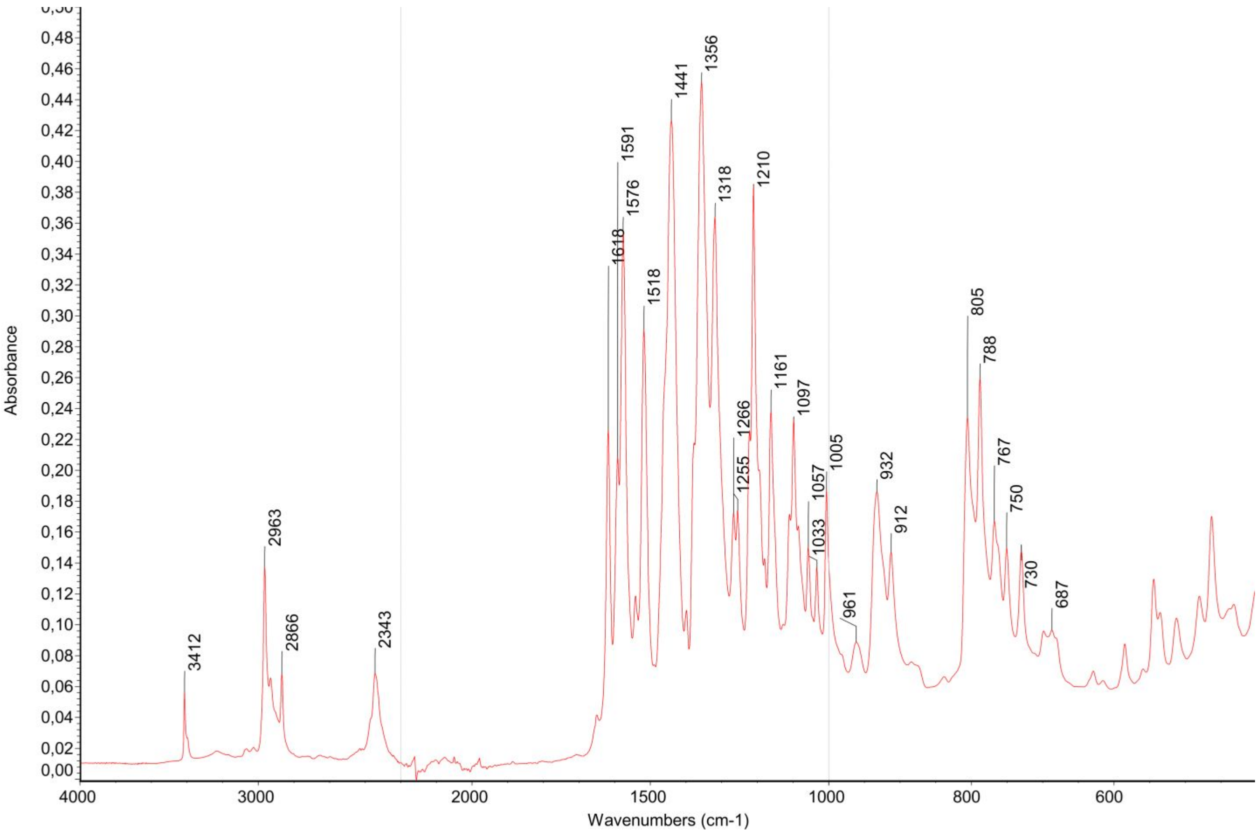

Figure S42. IR spectrum of **LH(BH₂)**[6].

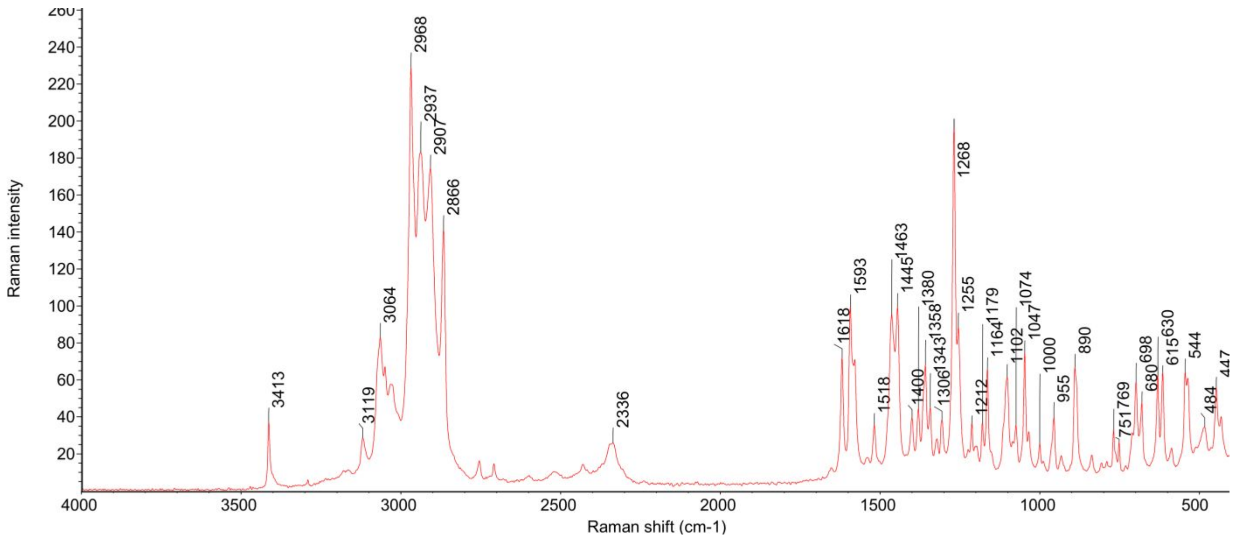

Figure S43. Raman spectrum of **LH(BH₂)**[6].

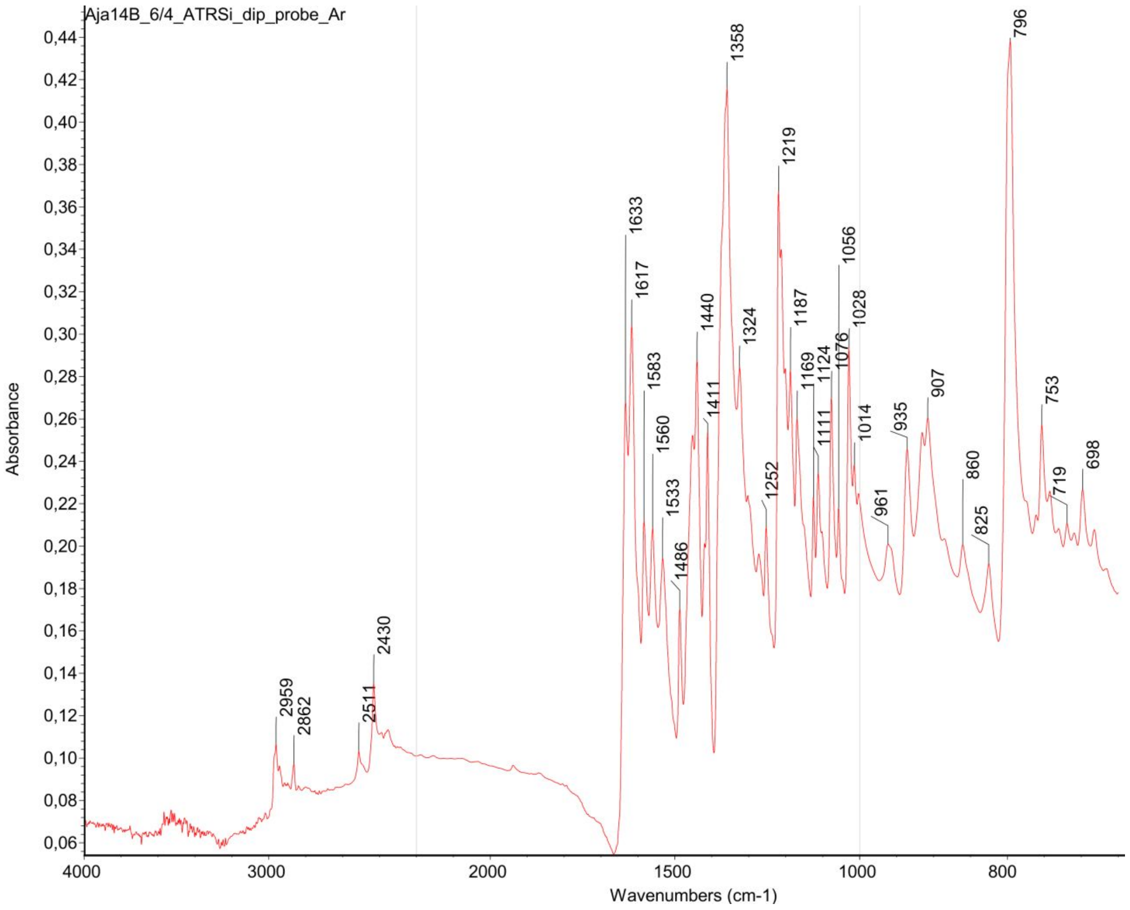

Figure S44. IR spectrum of **$L(BH_2)_2$**^{6,4}.

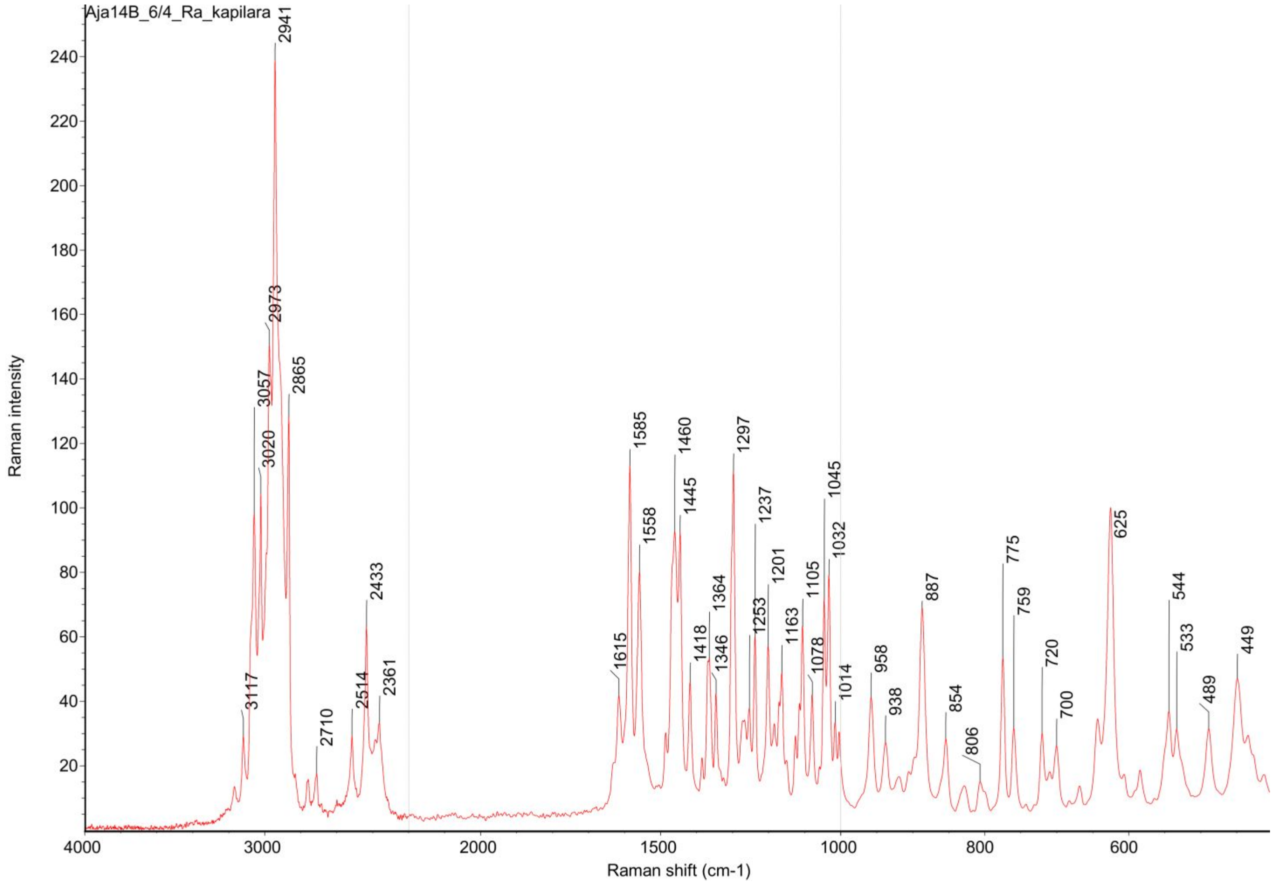

Figure S45. Raman spectrum of **$L(BH_2)_2$**^{6,4}.

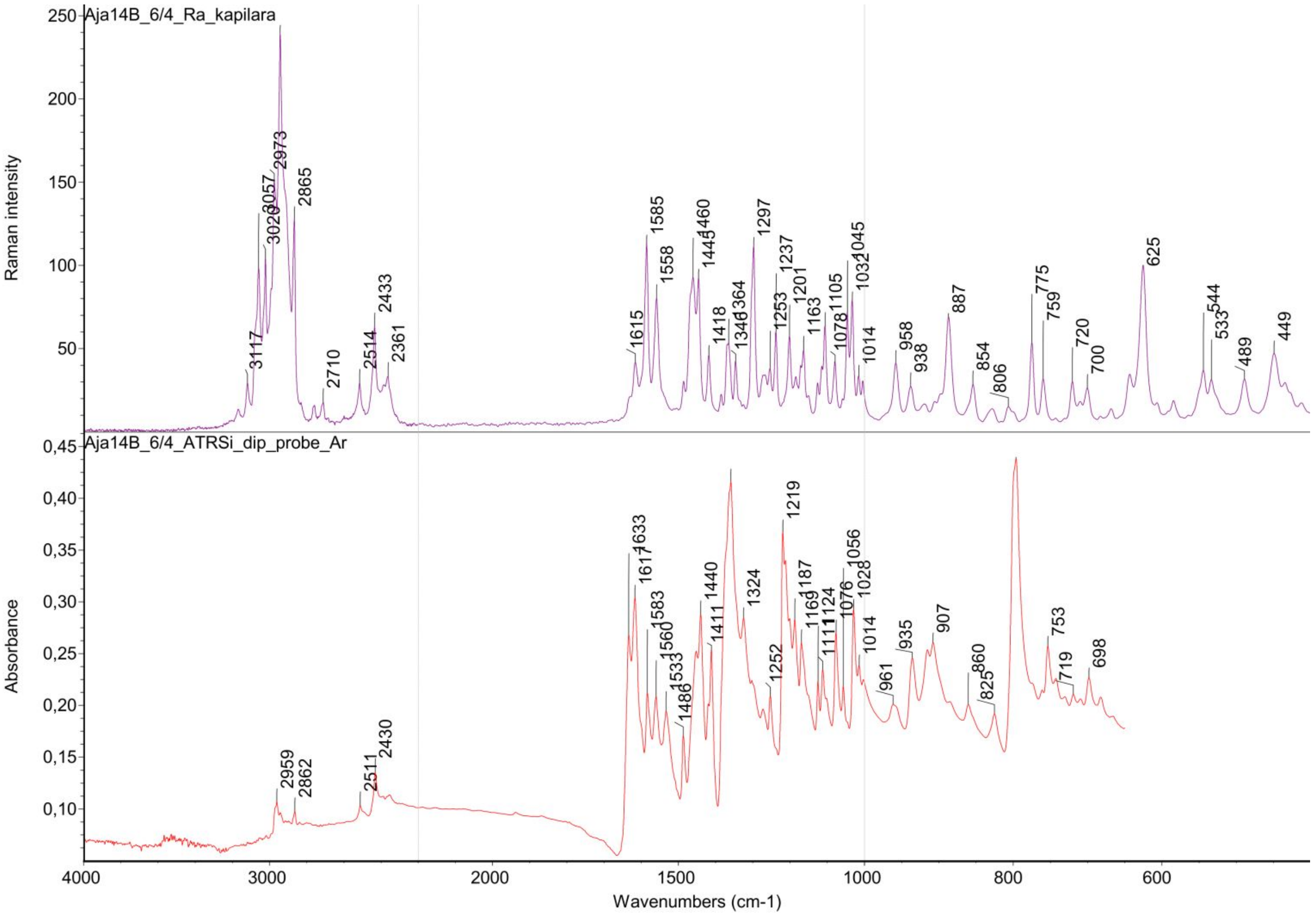

Figure S46. Overlay of Raman and IR spectra of **L(BH$_2$)$_2$**^{6,4}.

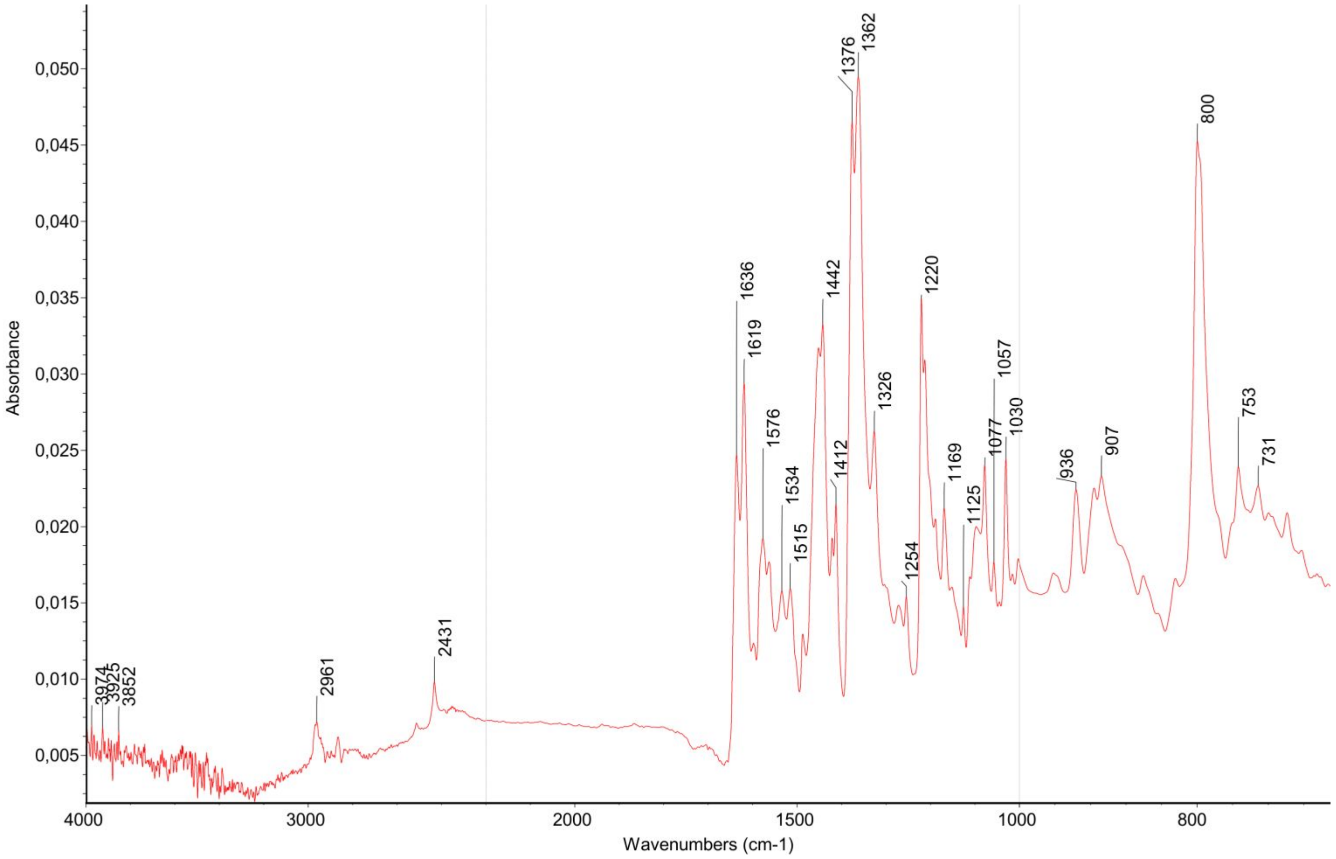

Figure S47. IR spectrum of **L(BH$_2$)$_2$**^{4,4}.

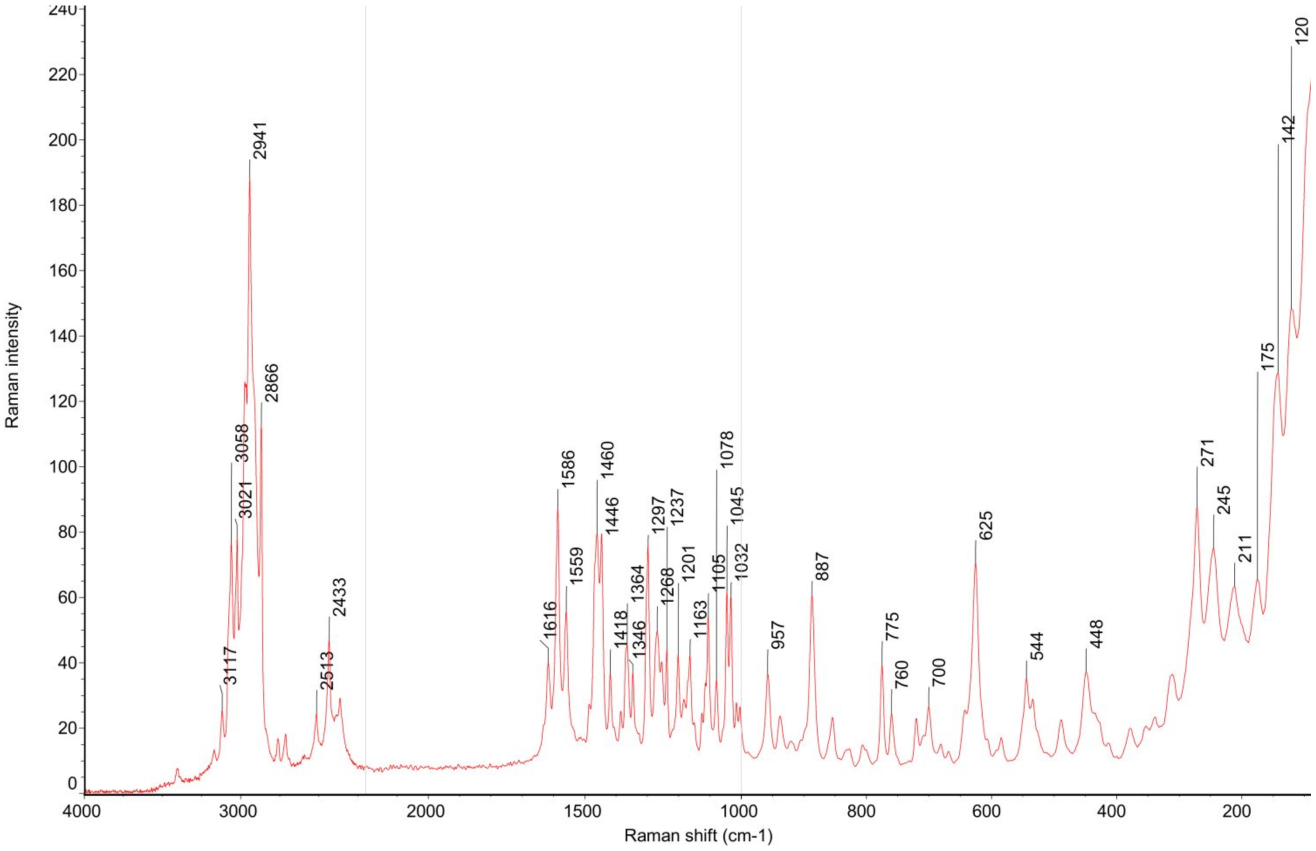

Figure S48. Raman spectrum of **L(BH₂)₂**^{4,4}.

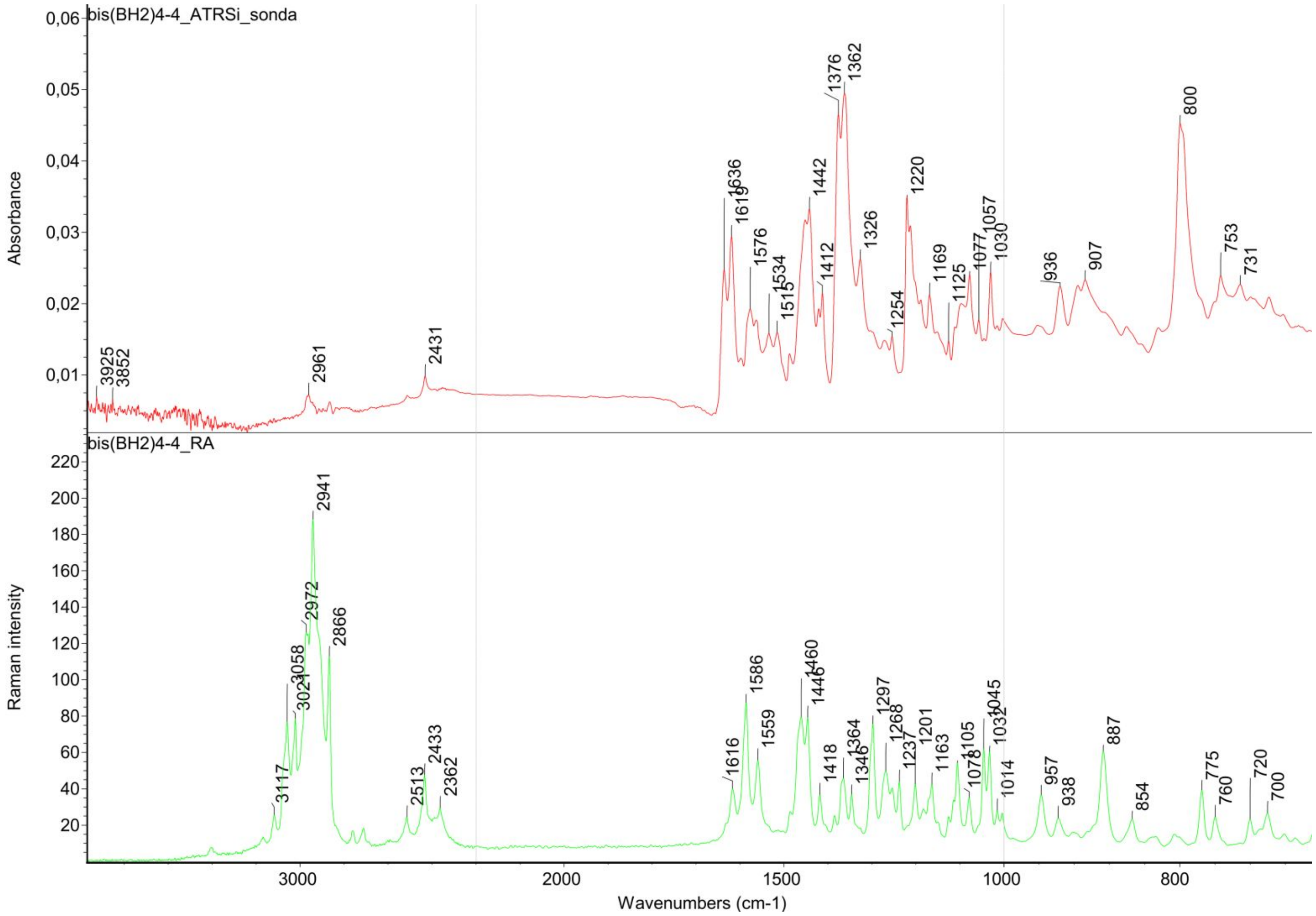

Figure S49. Overlay of Raman and IR spectra of **L(BH₂)₂**^{4,4}.

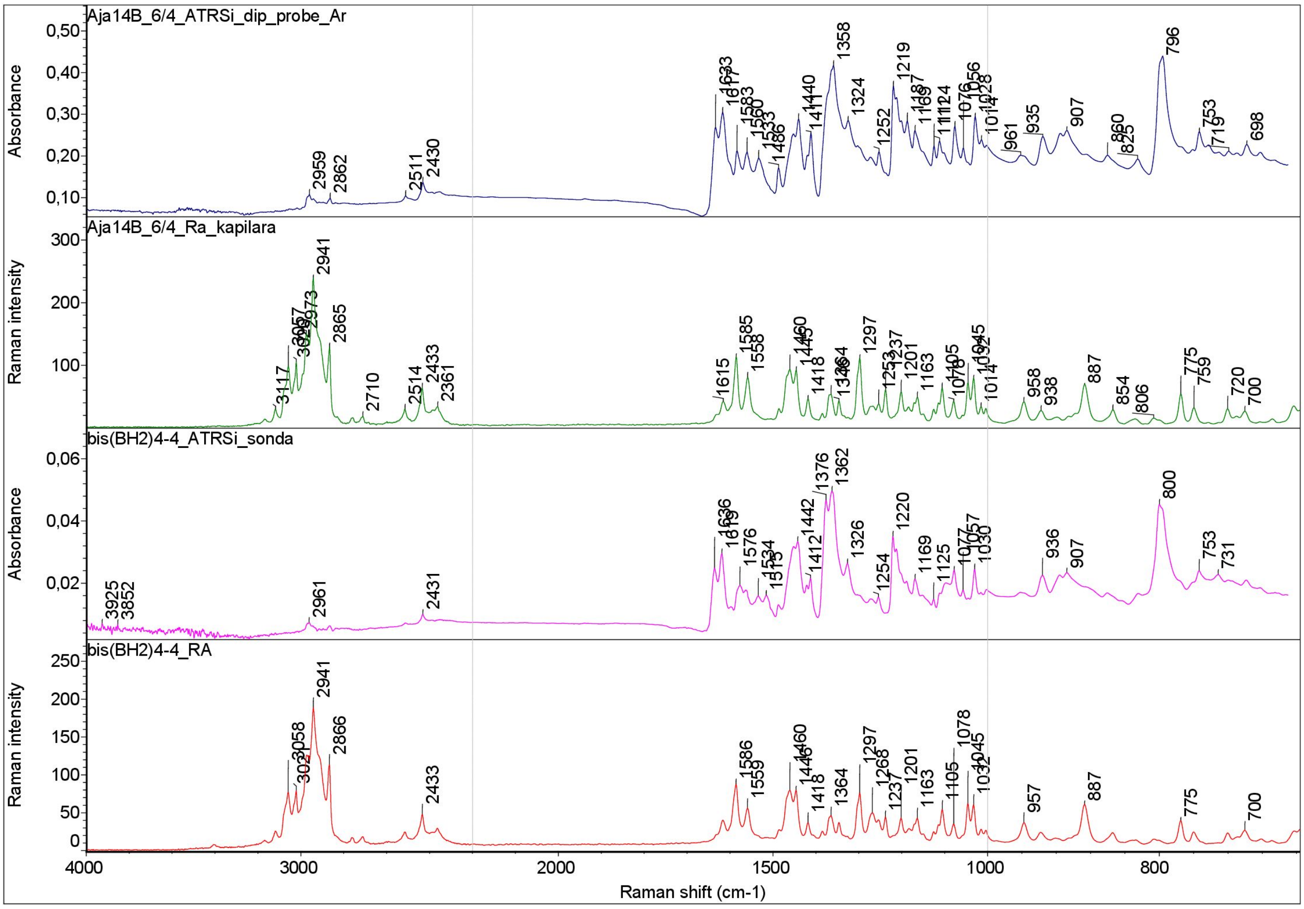

Figure S50. Overlay of Raman and IR spectra of **L(BH$_2$)$_2$**^{6,4} (top) and **L(BH$_2$)$_2$**^{4,4} (bottom).
