# Supplementary material for: Biguanide Complexes of Boron and Aluminum
Source: Inorg Chem. 2026 Jun 4;65(24):13350–61. doi: 10.1021/acs.inorgchem.6c00595 (PMC13292212; doi:10.1021/acs.inorgchem.6c00595)
Supplement: Supplementary file 3 [file ic6c00595_si_003.pdf]

Biguanide Complexes of Boron and Aluminum

Lukáš Vlk,[†] Tomáš Chlupatý,[†*] Alena Hoffmannová,[†] Zdeňka Růžičková,[†] Aleksandra Szymańska,[#§] Benjamin Théron,[‡] Raluca Malacea-Kabbara,[‡] Pierre Le Gendre,[‡*] Jędrzej Walkowiak,[#] Aleš Růžička[†*]

[†]*Department of General and Inorganic Chemistry, Faculty of Chemical Technology, University of Pardubice, Studentská 573, Pardubice 532 10, Czech Republic*

[‡]*Univ. Bourgogne Europe, Institut de Chimie Moléculaire de l'Université de Bourgogne (ICMUB), UMR CNRS 6302, 9 Avenue Alain Savary, 21078 Dijon, France*

[#]*Center for Advanced Technologies, Adam Mickiewicz University, Uniwersytetu Poznańskiego 10, 61-614 Poznań, Poland*

[§]*Faculty of Chemistry, Adam Mickiewicz University, Uniwersytetu Poznańskiego 8, 61-614 Poznań, Poland*

*tomas.chlupaty@upce.cz, pierre.le-gendre@u-bourgogne.fr, ales.ruzicka@upce.cz

TABLE OF CONTENTS

NMR spectra of all compounds

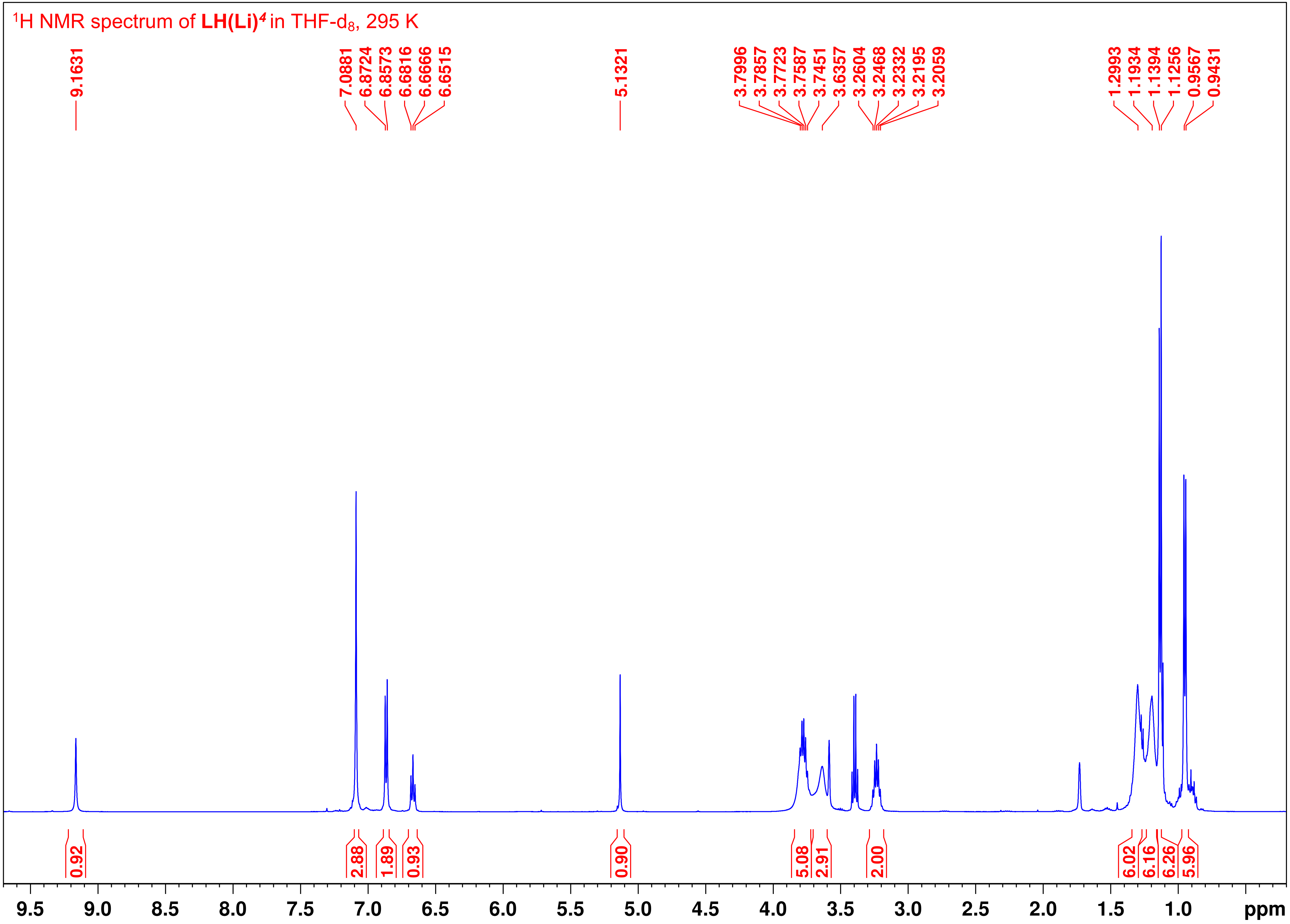

^{1}H NMR spectrum of **LH(Li)^4** in THF-d_8, 295 K
9.1631
7.0881
6.8724
6.8573
6.6816
6.6666
6.6515
5.1321
3.7996
3.7857
3.7723
3.7587
3.7451
3.6357
3.2604
3.2468
3.2332
3.2195
3.2059
1.2993
1.1934
1.1394
1.1256
0.9567
0.9431
0.92
2.88
1.89
0.93
0.90
5.08
2.91
2.00
6.02
6.16
6.26
5.96
9.5
9.0
8.5
8.0
7.5
7.0
6.5
6.0
5.5
5.0
4.5
4.0
3.5
3.0
2.5
2.0
1.5
1.0
ppm

^{13}C NMR spectrum of **LH(Li)**[4] in THF-d_8, 295 K

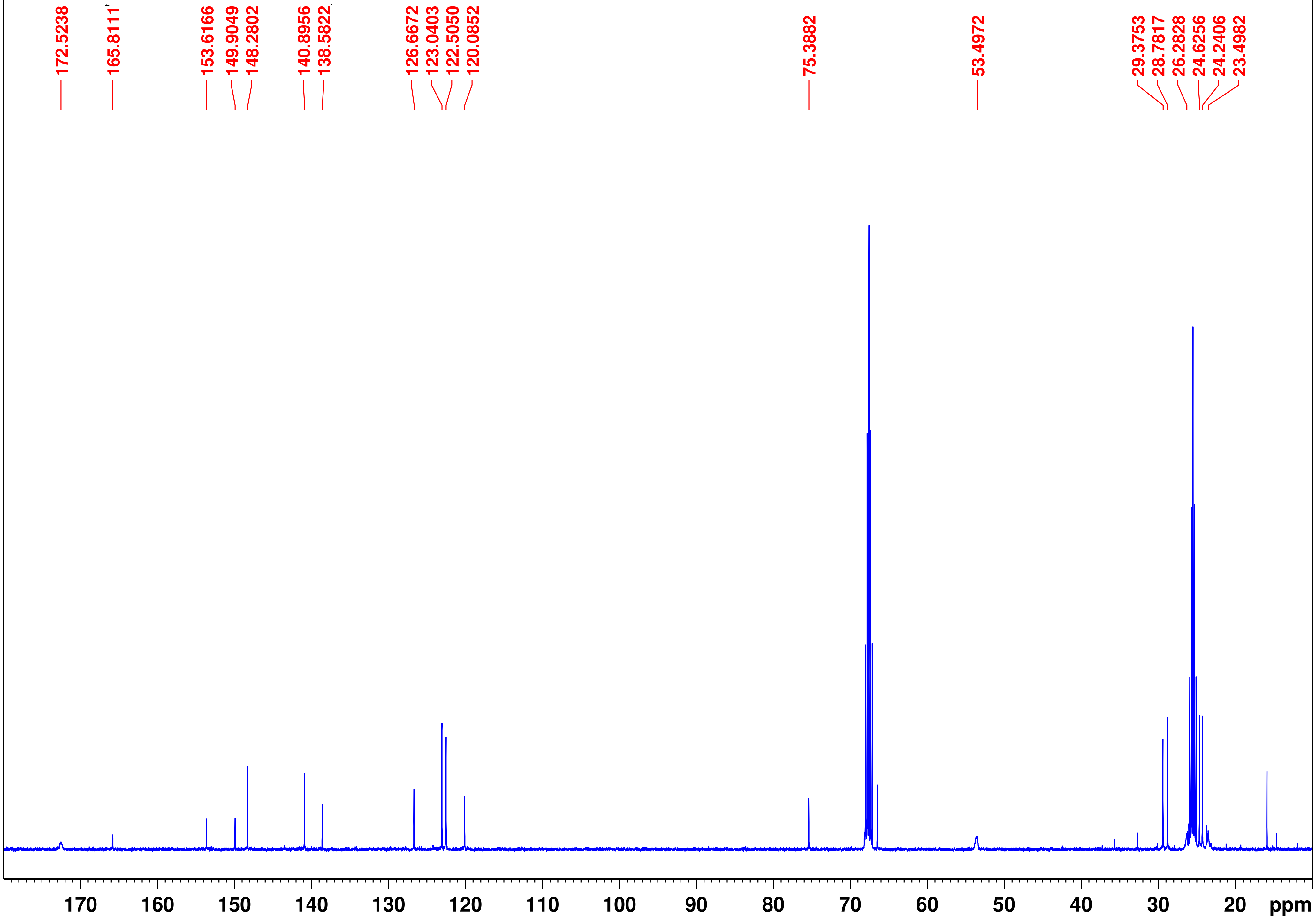

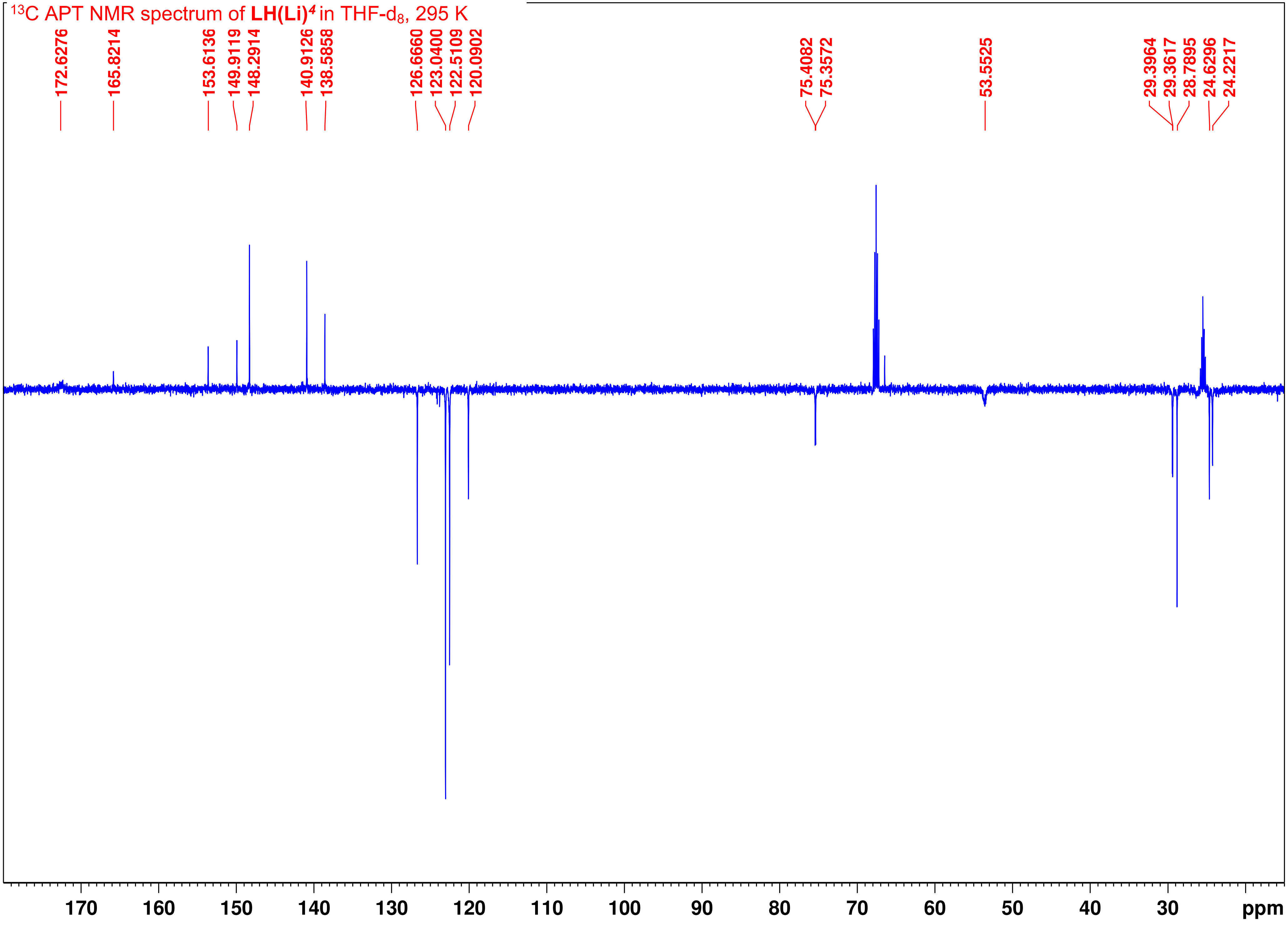

^{13}C APT NMR spectrum of **LH(Li)^4** in THF-d_8, 295 K
172.6276
165.8214
153.6136
149.9119
148.2914
140.9126
138.5858
126.6660
123.0400
122.5109
120.0902
75.4082
75.3572
53.5525
29.3964
29.3617
28.7895
24.6296
24.2217
170
160
150
140
130
120
110
100
90
80
70
60
50
40
30
ppm

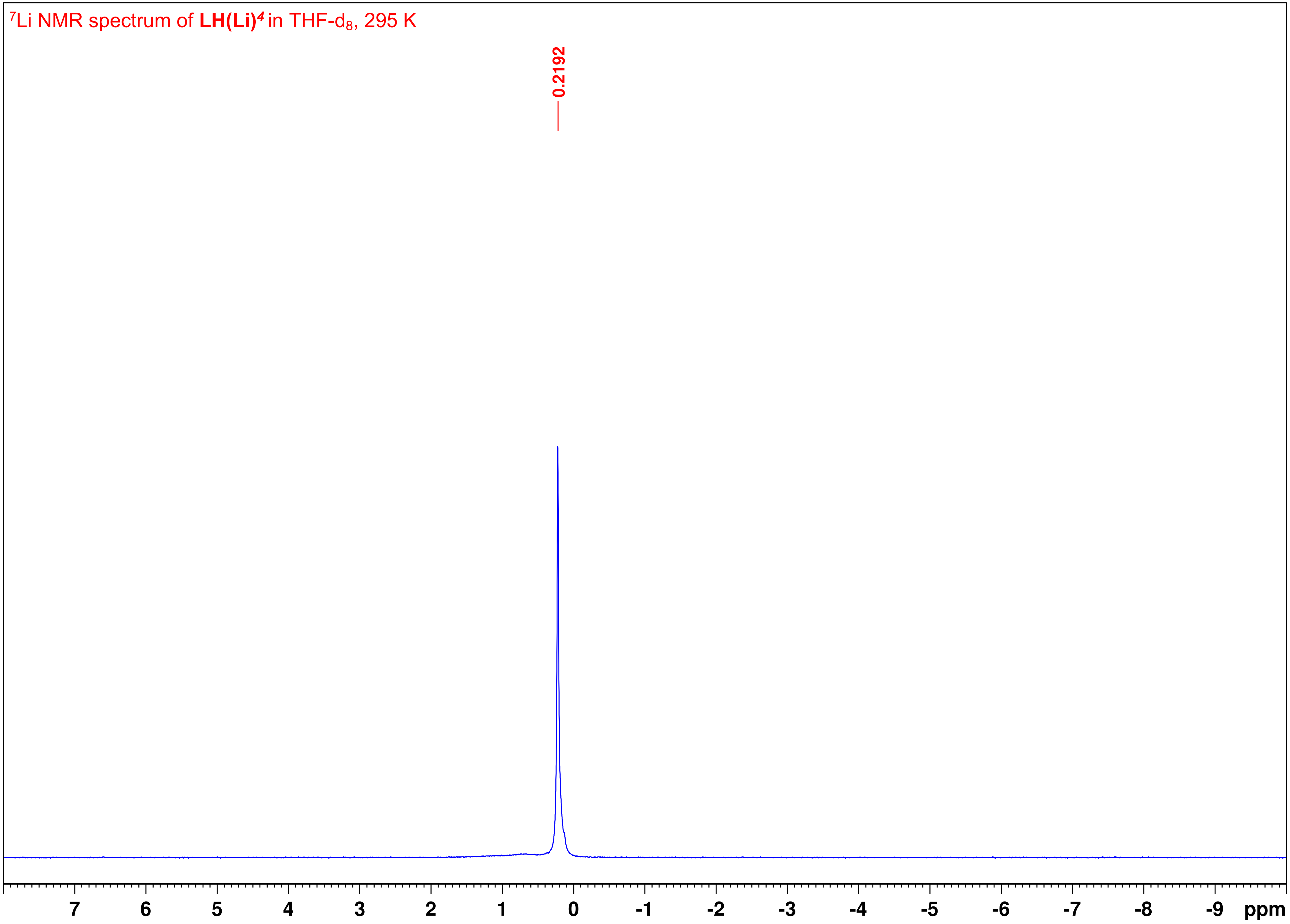
^{7}Li NMR spectrum of **LH(Li)*4*** in THF-d_8, 295 K
0.2192
7
6
5
4
3
2
1
0
-1
-2
-3
-4
-5
-6
-7
-8
-9
ppm

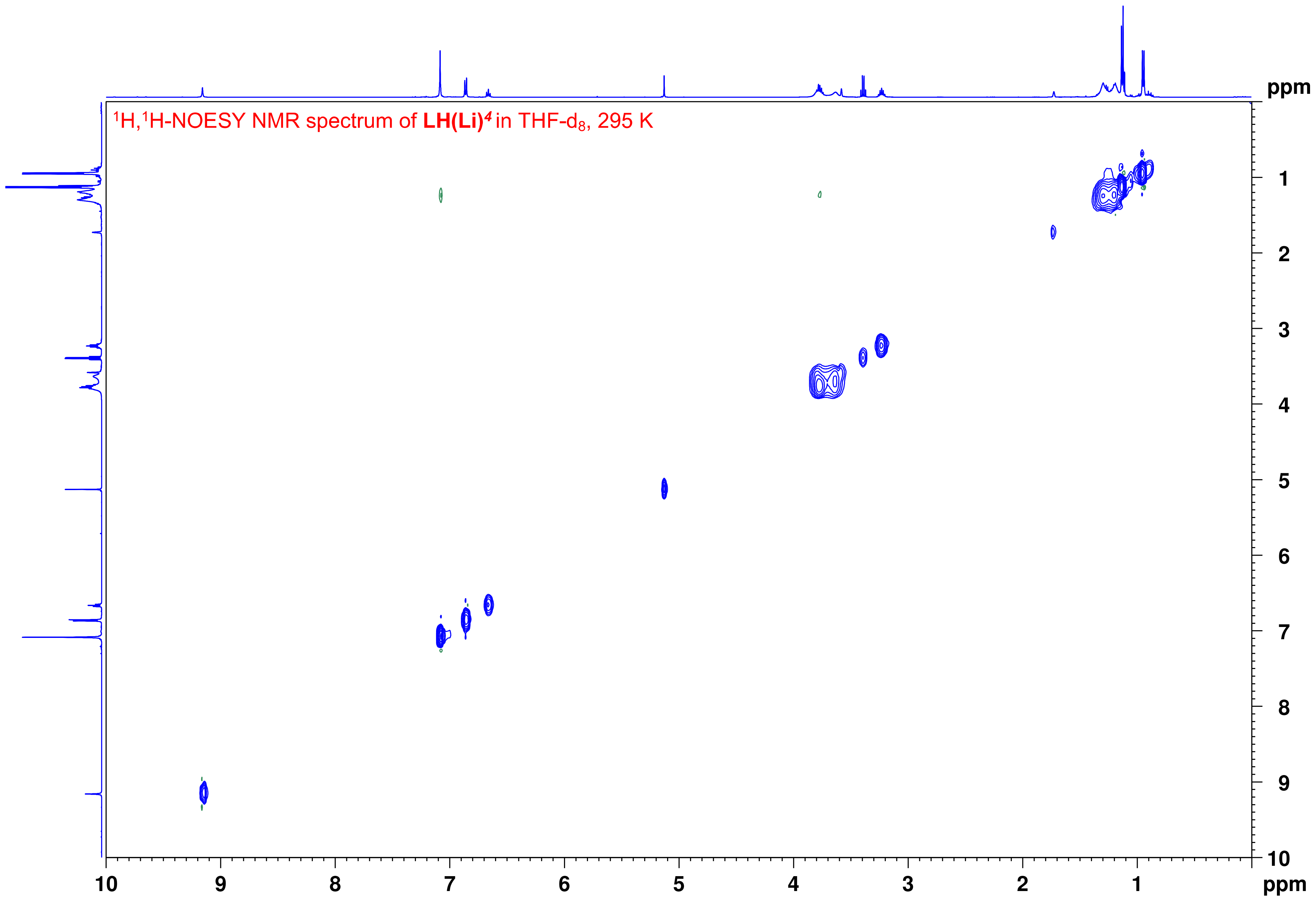
$^1H,^1H$-NOESY NMR spectrum of **LH(Li)*4*** in THF-d_8, 295 K
ppm
1
2
3
4
5
6
7
8
9
10

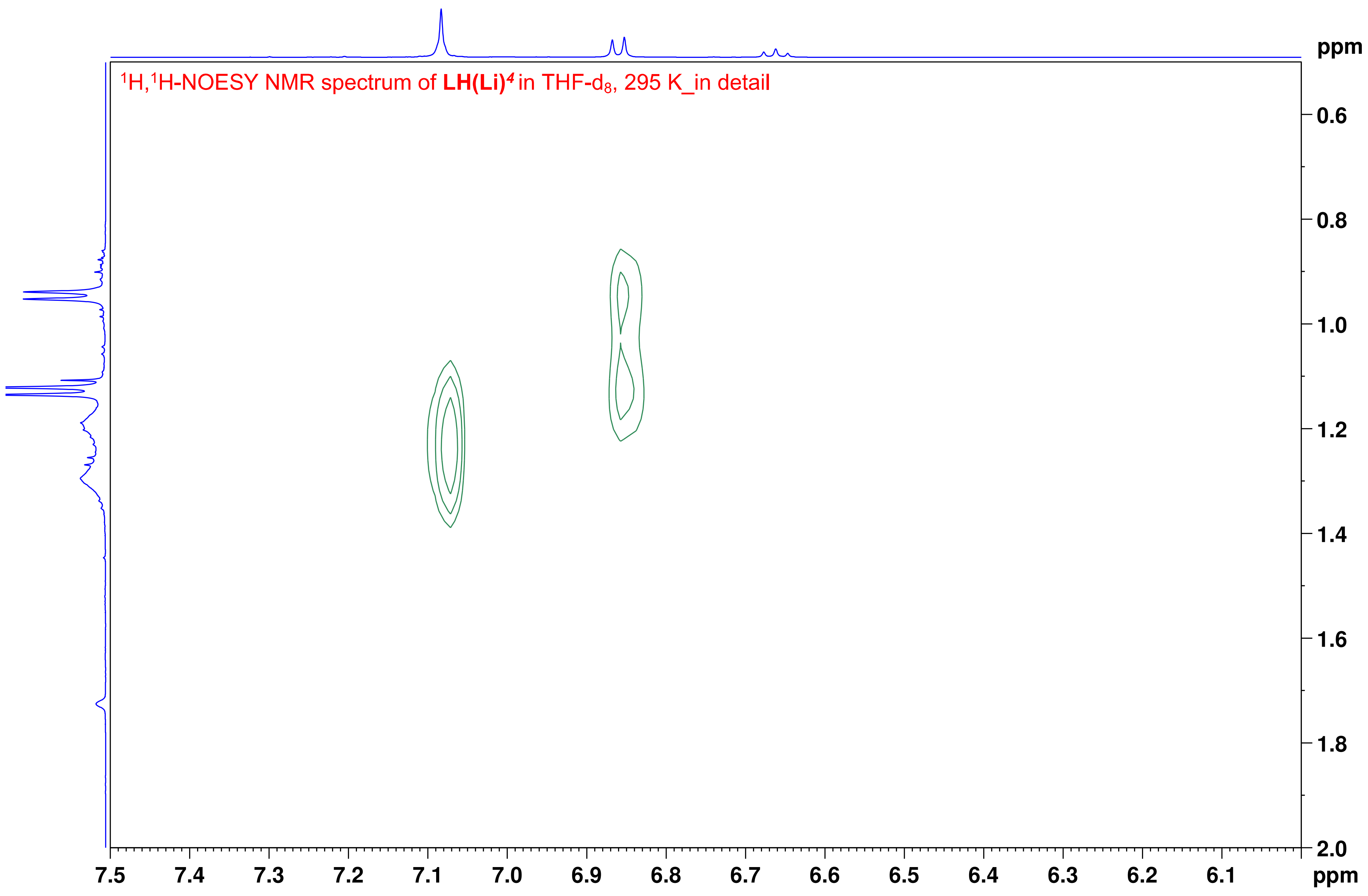

$^{1}H,^{1}H$-NOESY NMR spectrum of **LH(Li)⁴** in THF-d_8, 295 K_in detail
ppm
0.6
0.8
1.0
1.2
1.4
1.6
1.8
2.0
7.5
7.4
7.3
7.2
7.1
7.0
6.9
6.8
6.7
6.6
6.5
6.4
6.3
6.2
6.1
ppm

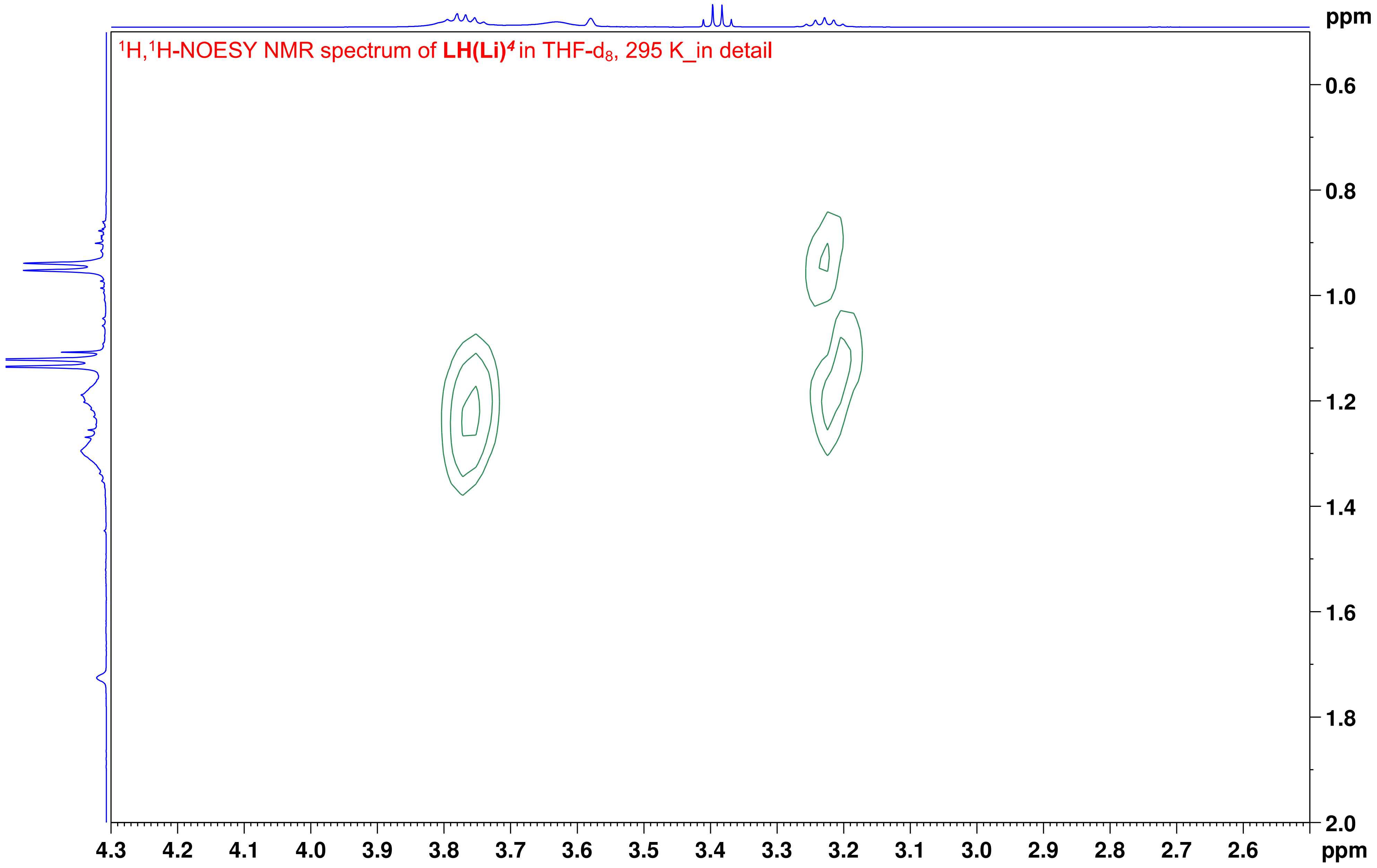
1H,1H-NOESY NMR spectrum of LH(Li)4 in THF-d8, 295 K_in detail
ppm
0.6
0.8
1.0
1.2
1.4
1.6
1.8
2.0
4.3
4.2
4.1
4.0
3.9
3.8
3.7
3.6
3.5
3.4
3.3
3.2
3.1
3.0
2.9
2.8
2.7
2.6
ppm

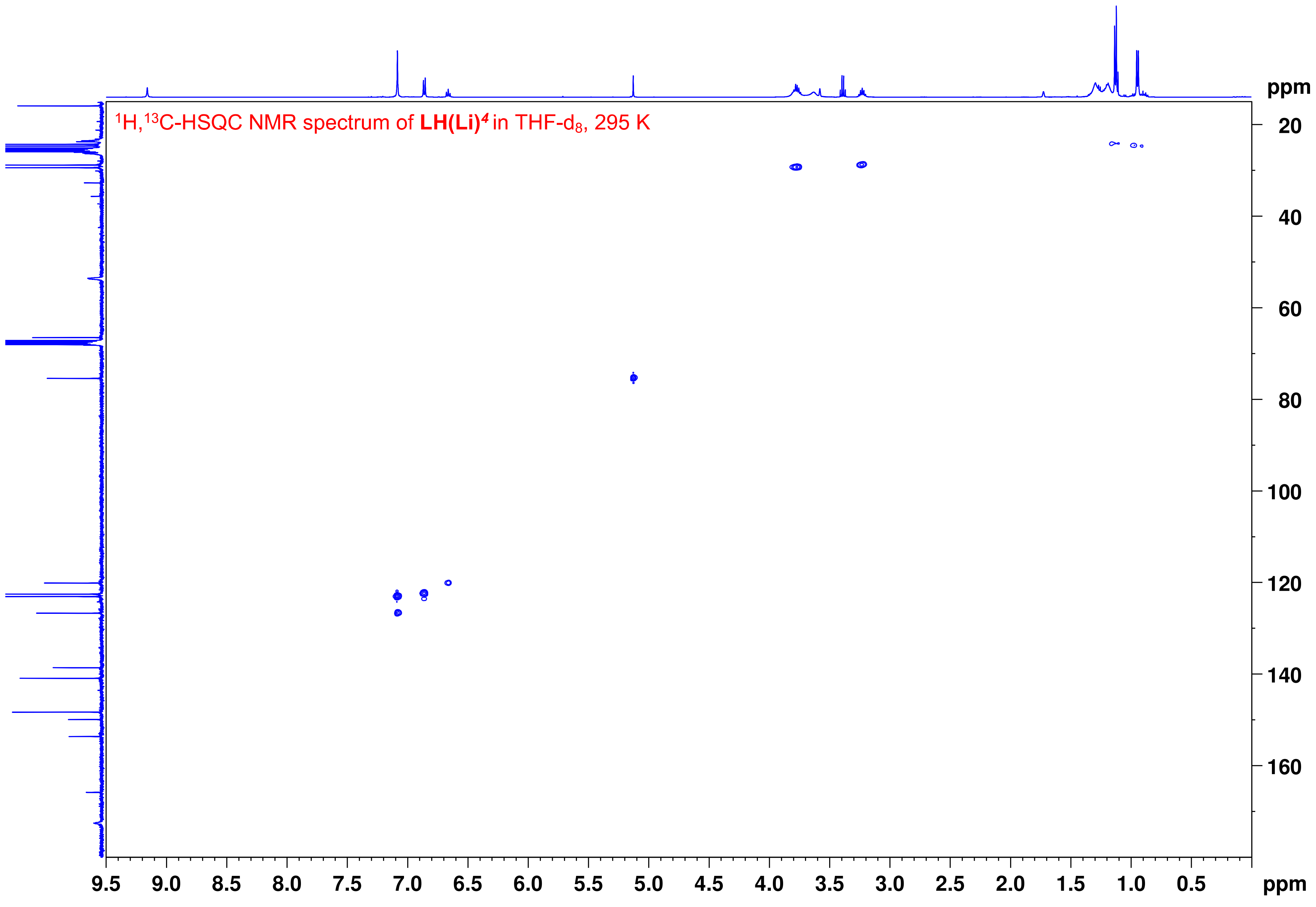

$^{1}H,^{13}C$-HSQC NMR spectrum of **LH(Li)**^4^ in THF-d_8, 295 K
ppm
20
40
60
80
100
120
140
160
9.5
9.0
8.5
8.0
7.5
7.0
6.5
6.0
5.5
5.0
4.5
4.0
3.5
3.0
2.5
2.0
1.5
1.0
0.5
ppm

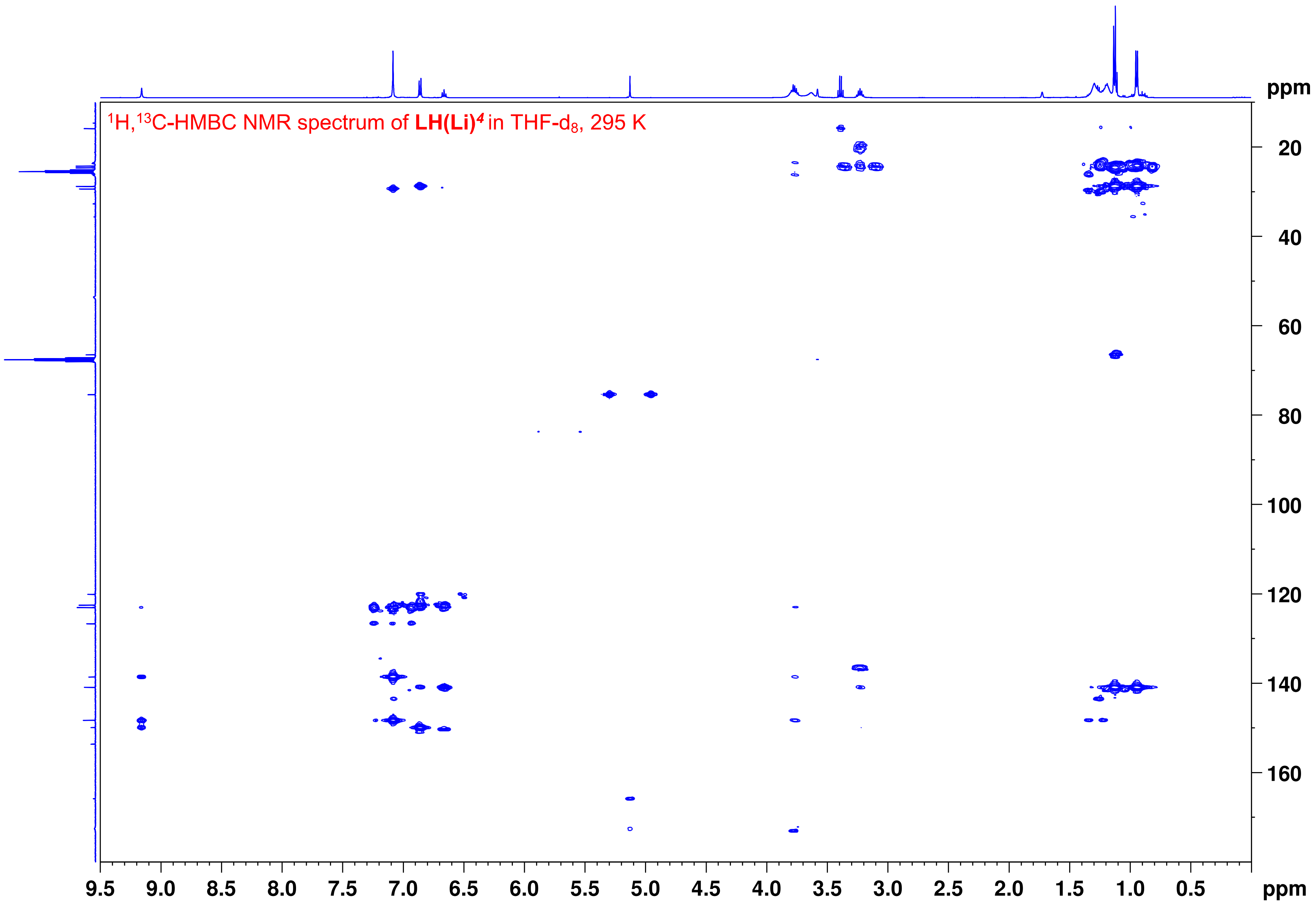
$^{1}H,^{13}C$-HMBC NMR spectrum of **LH(Li)**^{4} in THF-d_8, 295 K
ppm
20
40
60
80
100
120
140
160
9.5
9.0
8.5
8.0
7.5
7.0
6.5
6.0
5.5
5.0
4.5
4.0
3.5
3.0
2.5
2.0
1.5
1.0
0.5
ppm

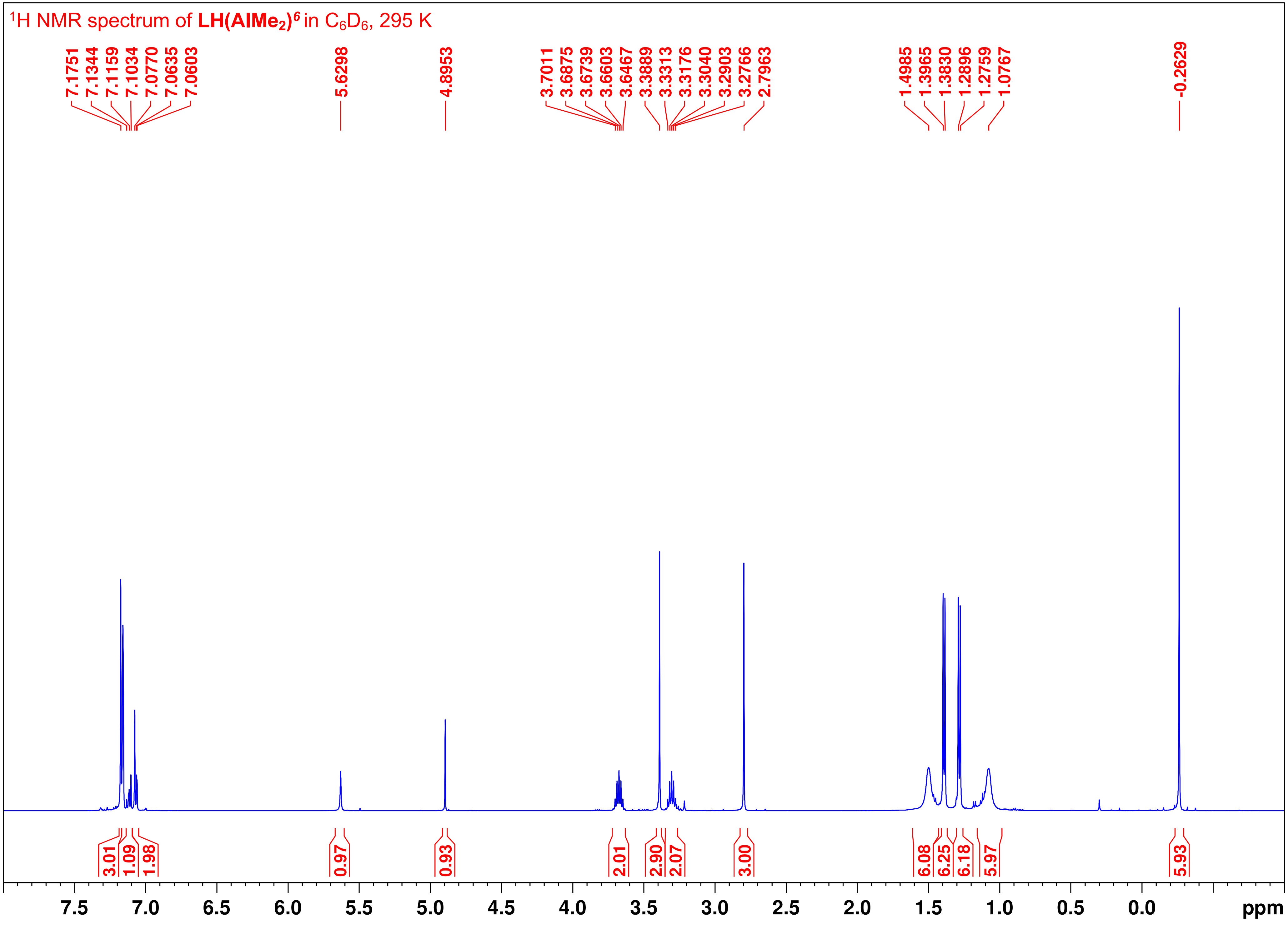
1H NMR spectrum of LH(AlMe2)6 in C6D6, 295 K
7.1751
7.1344
7.1159
7.1034
7.0770
7.0635
7.0603
5.6298
4.8953
3.7011
3.6875
3.6739
3.6603
3.6467
3.3889
3.3313
3.3176
3.3040
3.2903
3.2766
2.7963
1.4985
1.3965
1.3830
1.2896
1.2759
1.0767
-0.2629
3.01
1.09
1.98
0.97
0.93
2.01
2.90
2.07
3.00
6.08
6.25
6.18
5.97
5.93
7.5
7.0
6.5
6.0
5.5
5.0
4.5
4.0
3.5
3.0
2.5
2.0
1.5
1.0
0.5
0.0
ppm

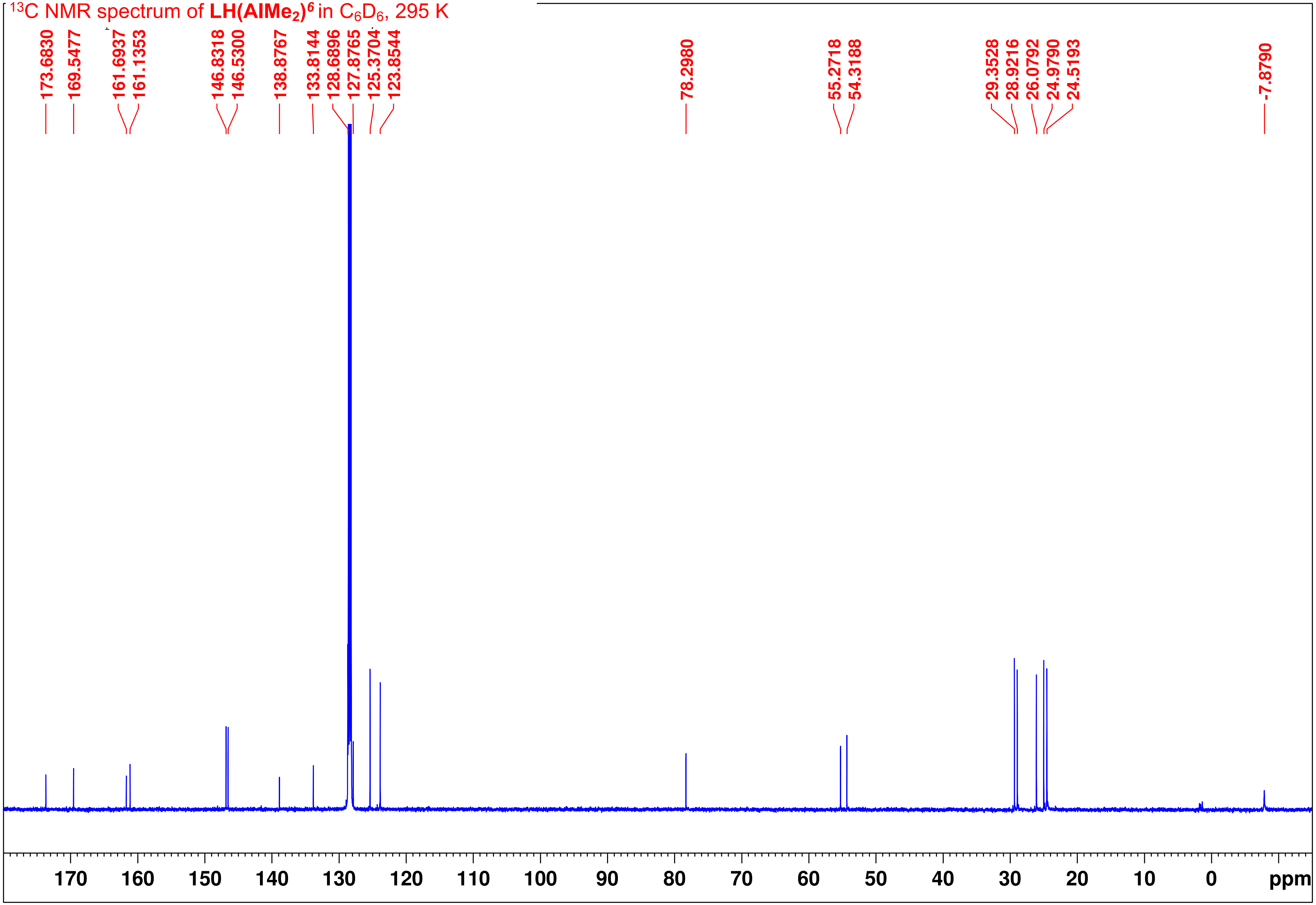
13C NMR spectrum of LH(AlMe2)6 in C6D6, 295 K
173.6830
169.5477
161.6937
161.1353
146.8318
146.5300
138.8767
133.8144
128.6896
127.8765
125.3704
123.8544
78.2980
55.2718
54.3188
29.3528
28.9216
26.0792
24.9790
24.5193
-7.8790
170
160
150
140
130
120
110
100
90
80
70
60
50
40
30
20
10
0
ppm

^{13}C APT NMR spectrum of **LH(AlMe$_2$)**[6] in C_6D_6, 295 K

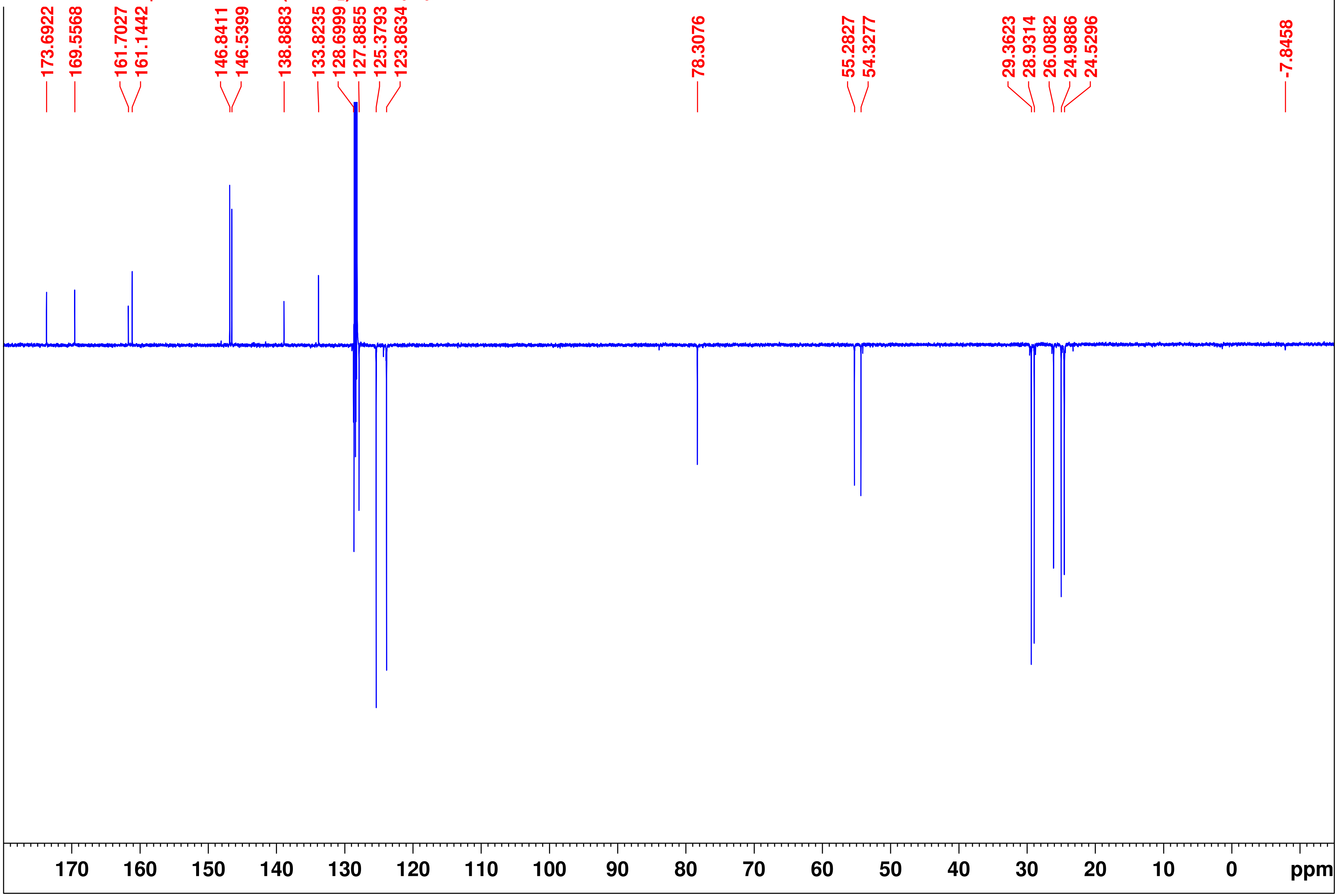

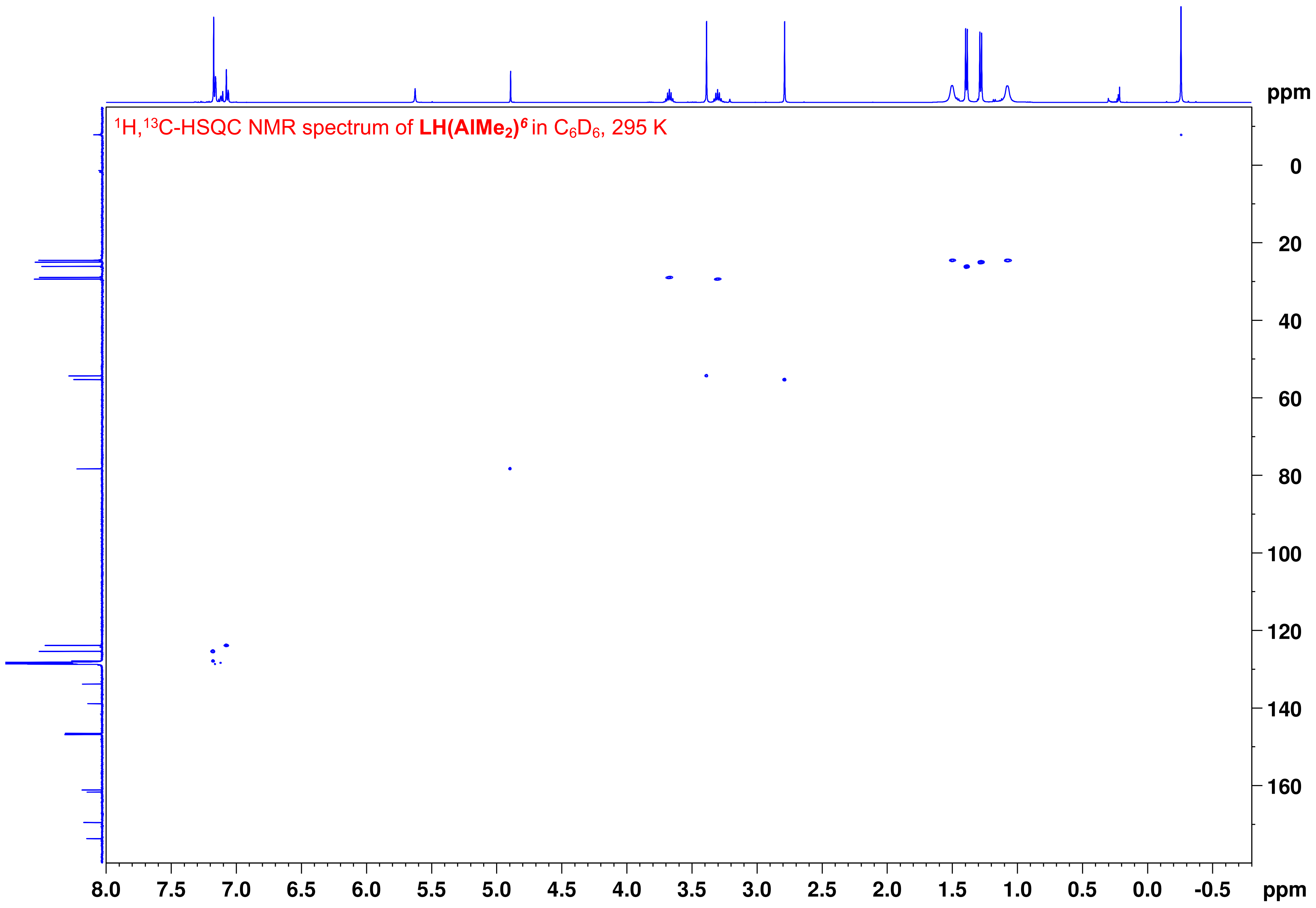
$^{1}H,^{13}C$-HSQC NMR spectrum of **LH(AlMe$_2$)^{6}** in C_6D_6, 295 K
ppm
0
20
40
60
80
100
120
140
160
ppm
8.0
7.5
7.0
6.5
6.0
5.5
5.0
4.5
4.0
3.5
3.0
2.5
2.0
1.5
1.0
0.5
0.0
-0.5
ppm

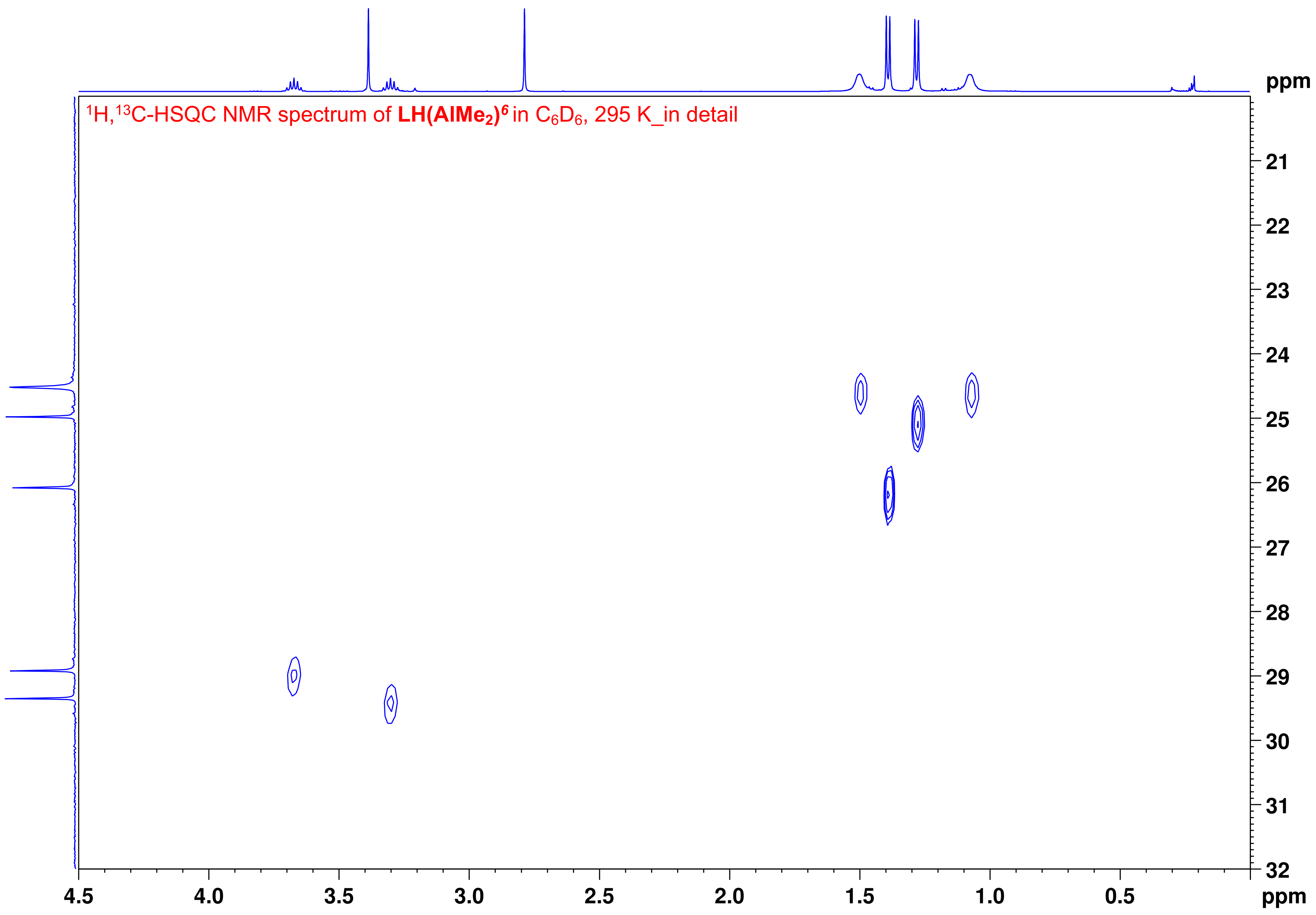

$^{1}H,^{13}C$-HSQC NMR spectrum of **LH(AlMe$_2$)^6** in C_6D_6, 295 K_in detail
ppm
21
22
23
24
25
26
27
28
29
30
31
32
4.5
4.0
3.5
3.0
2.5
2.0
1.5
1.0
0.5
ppm

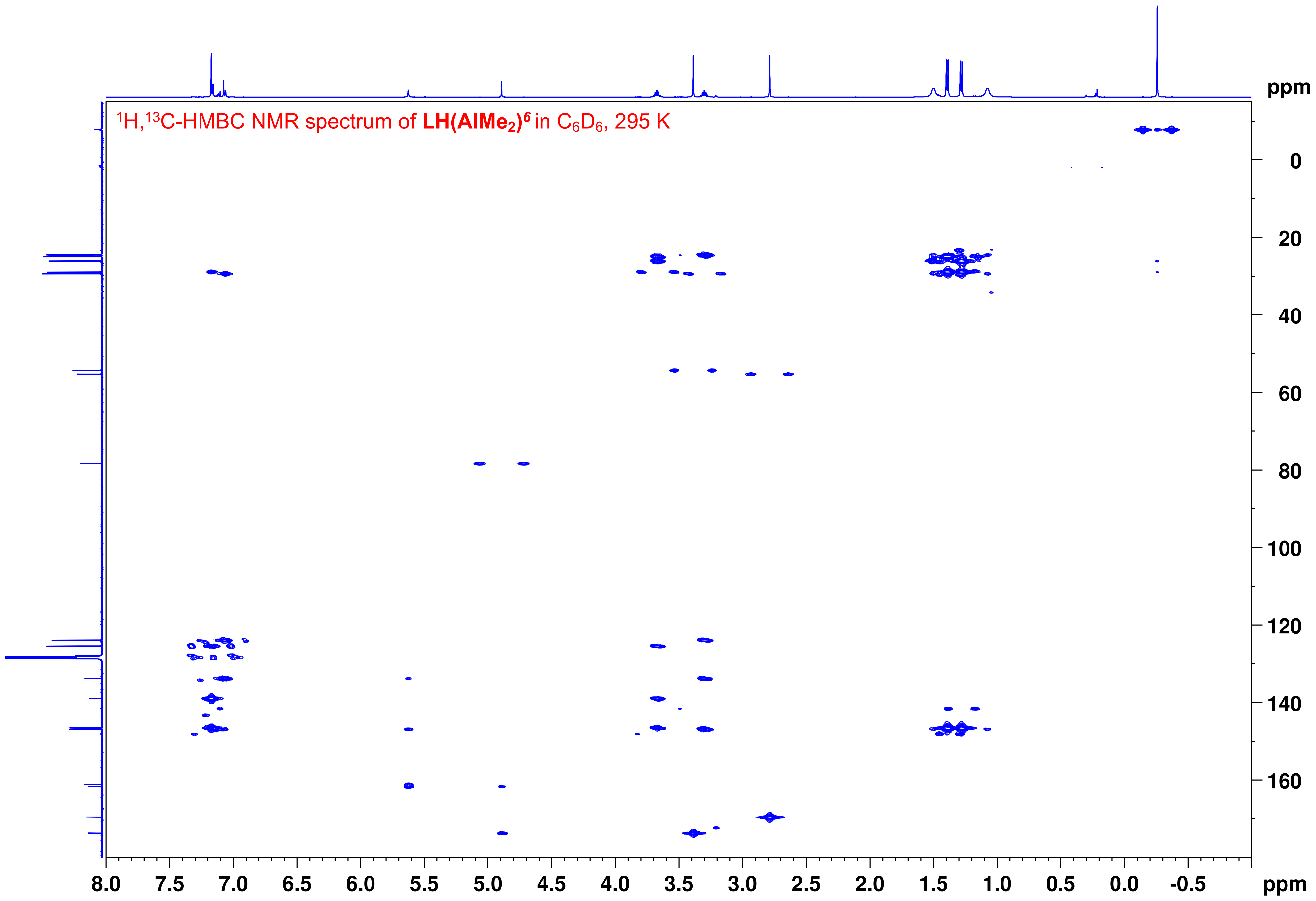
$^1H,^{13}C$-HMBC NMR spectrum of **LH(AlMe$_2$)^6** in C_6D_6, 295 K
ppm
0
20
40
60
80
100
120
140
160
8.0
7.5
7.0
6.5
6.0
5.5
5.0
4.5
4.0
3.5
3.0
2.5
2.0
1.5
1.0
0.5
0.0
-0.5

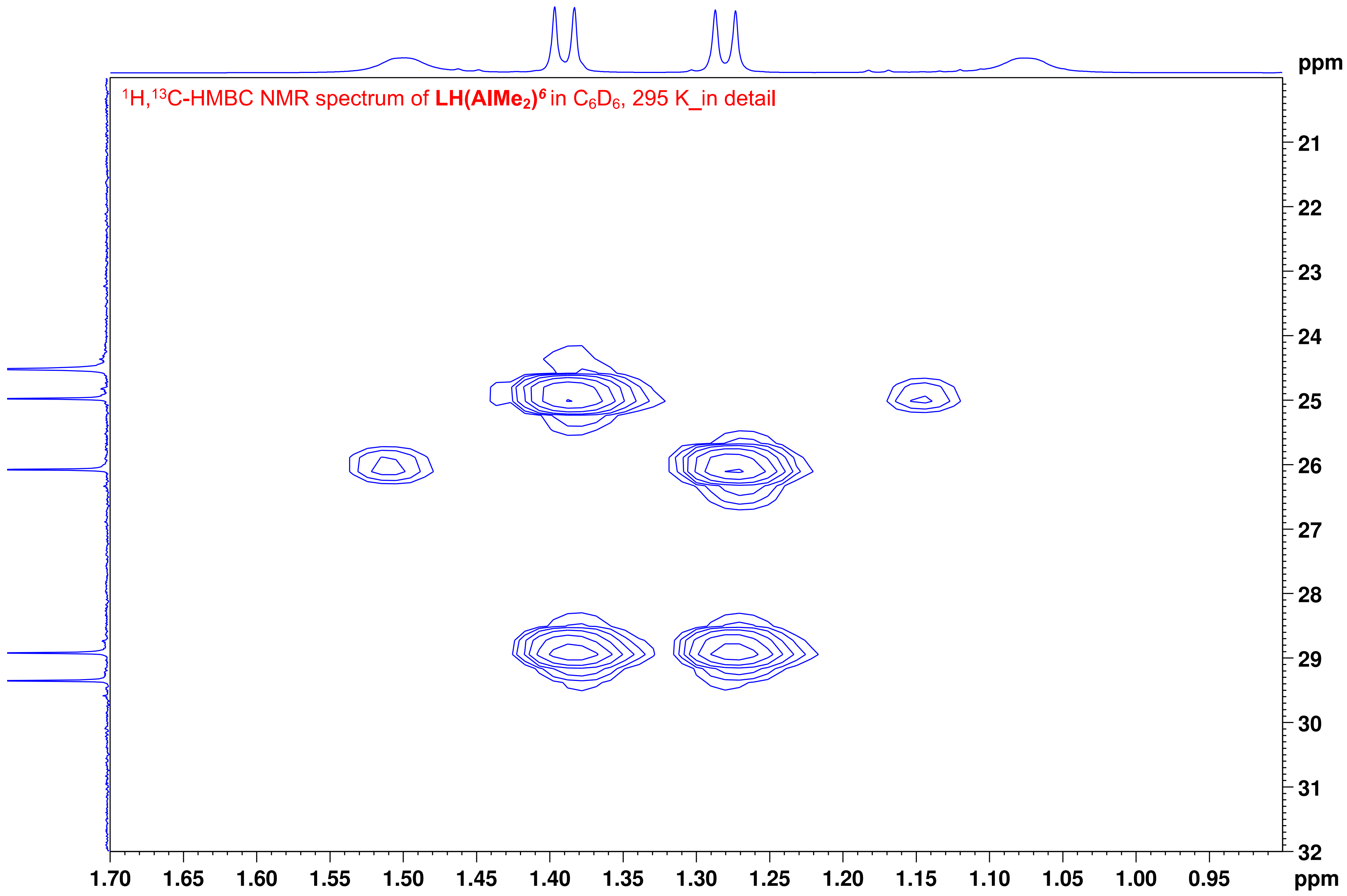

^{1}H,^{13}C-HMBC NMR spectrum of **LH(AlMe$_2$)^6** in C_6D_6, 295 K_in detail
ppm
21
22
23
24
25
26
27
28
29
30
31
32
1.70
1.65
1.60
1.55
1.50
1.45
1.40
1.35
1.30
1.25
1.20
1.15
1.10
1.05
1.00
0.95
ppm

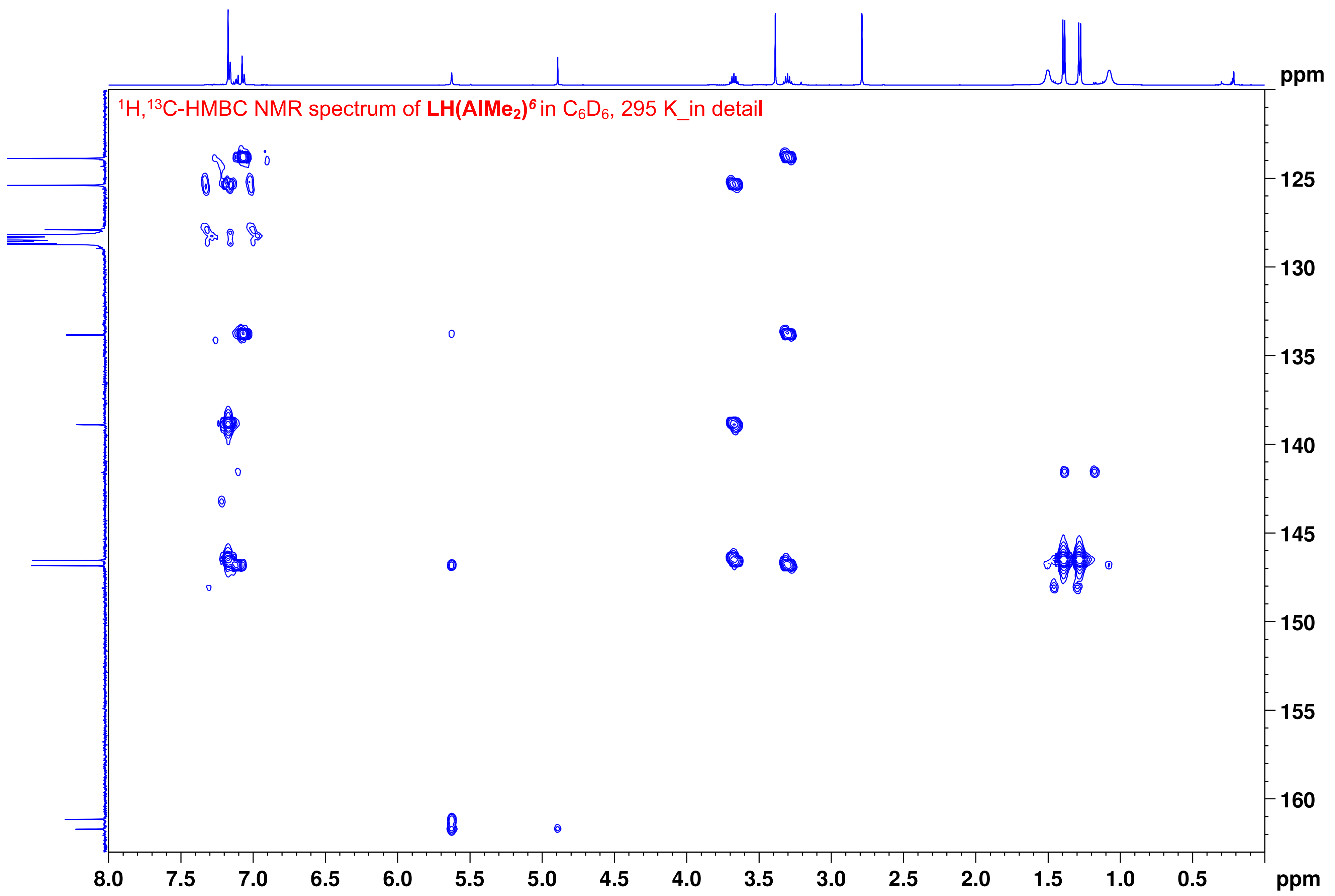

1H,13C-HMBC NMR spectrum of LH(AlMe2)6 in C6D6, 295 K_in detail
ppm
125
130
135
140
145
150
155
160
8.0
7.5
7.0
6.5
6.0
5.5
5.0
4.5
4.0
3.5
3.0
2.5
2.0
1.5
1.0
0.5
ppm

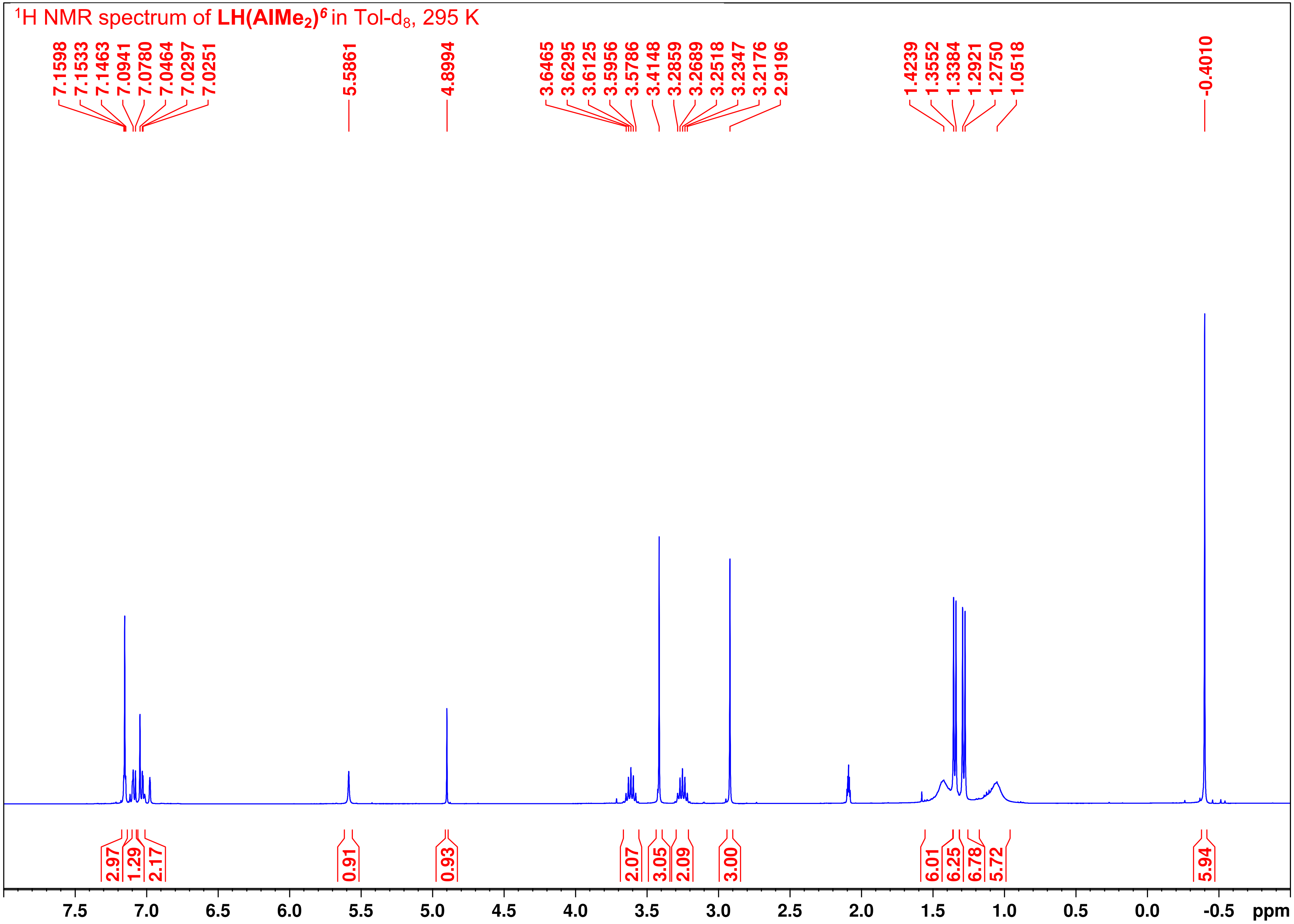

^{1}H NMR spectrum of **LH(AlMe$_2$)^6** in Tol-d$_8$, 295 K
7.1598
7.1533
7.1463
7.0941
7.0780
7.0464
7.0297
7.0251
5.5861
4.8994
3.6465
3.6295
3.6125
3.5956
3.5786
3.4148
3.2859
3.2689
3.2518
3.2347
3.2176
2.9196
1.4239
1.3552
1.3384
1.2921
1.2750
1.0518
-0.4010
2.97
1.29
2.17
0.91
0.93
2.07
3.05
2.09
3.00
6.01
6.25
6.78
5.72
5.94
7.5
7.0
6.5
6.0
5.5
5.0
4.5
4.0
3.5
3.0
2.5
2.0
1.5
1.0
0.5
0.0
-0.5
ppm

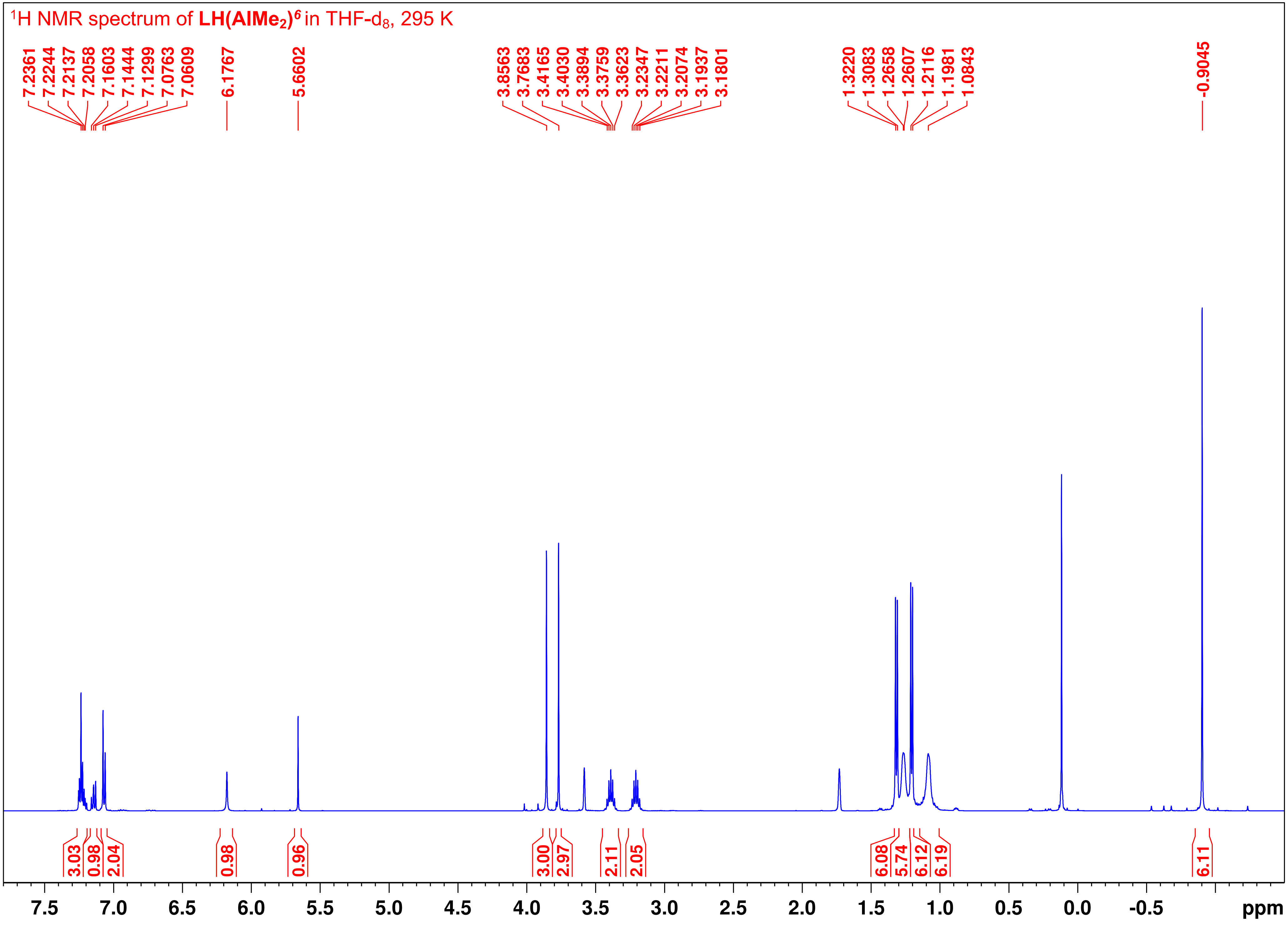

^{1}H NMR spectrum of **LH(AlMe$_2$)^6** in THF-d$_8$, 295 K
7.2361
7.2244
7.2137
7.2058
7.1603
7.1444
7.1299
7.0763
7.0609
6.1767
5.6602
3.8563
3.7683
3.4165
3.4030
3.3894
3.3759
3.3623
3.2347
3.2211
3.2074
3.1937
3.1801
1.3220
1.3083
1.2658
1.2607
1.2116
1.1981
1.0843
-0.9045
3.03
0.98
2.04
0.98
0.96
3.00
2.97
2.11
2.05
6.08
5.74
6.12
6.19
6.11
7.5
7.0
6.5
6.0
5.5
5.0
4.5
4.0
3.5
3.0
2.5
2.0
1.5
1.0
0.5
0.0
-0.5
ppm

^{13}C NMR spectrum of **LH(AlMe$_2$)^6** in THF-d_8, 295 K

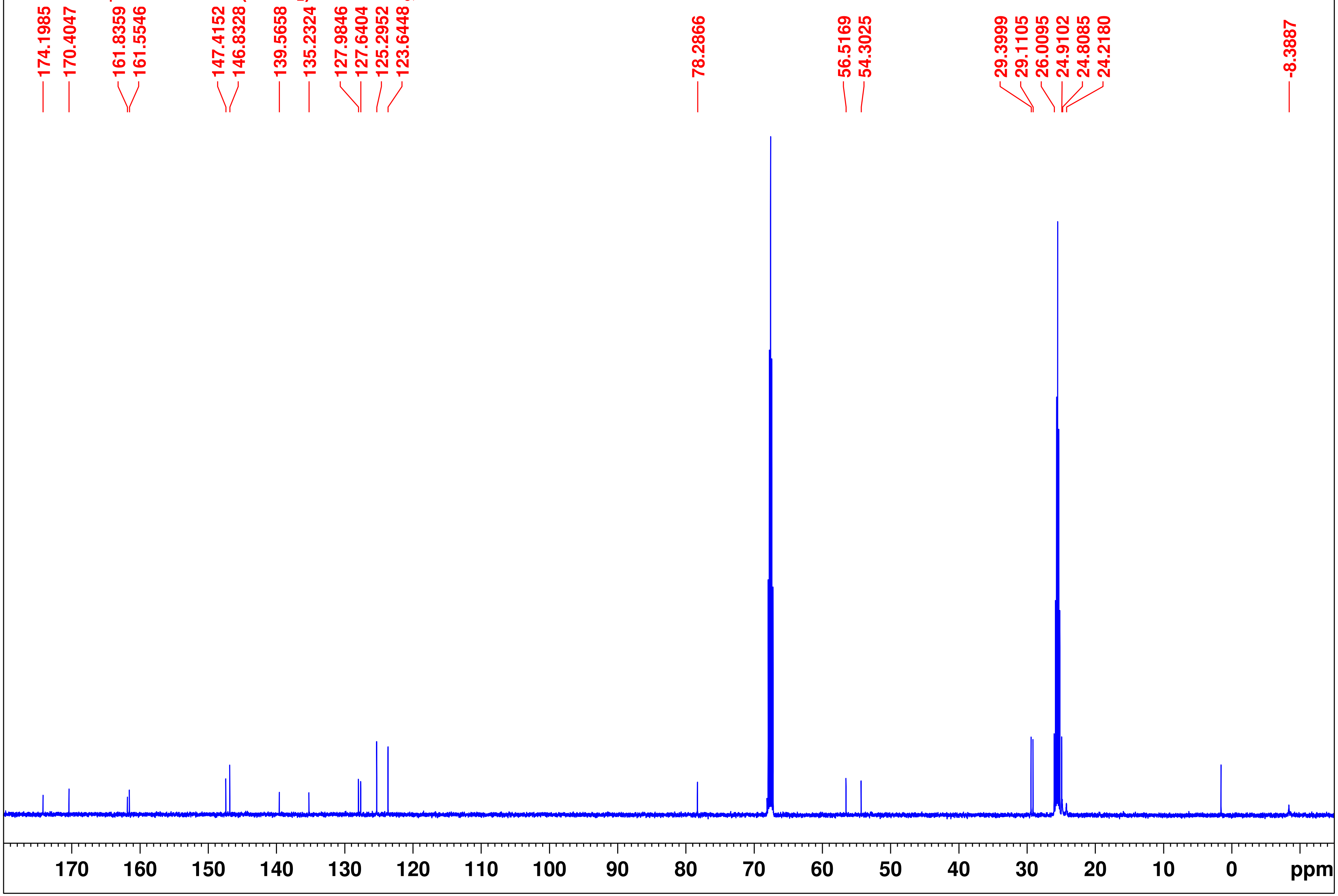

^{13}C NMR spectrum of **LH(AlMe$_2$)^6** in THF-d$_8$, 295 K_in detail

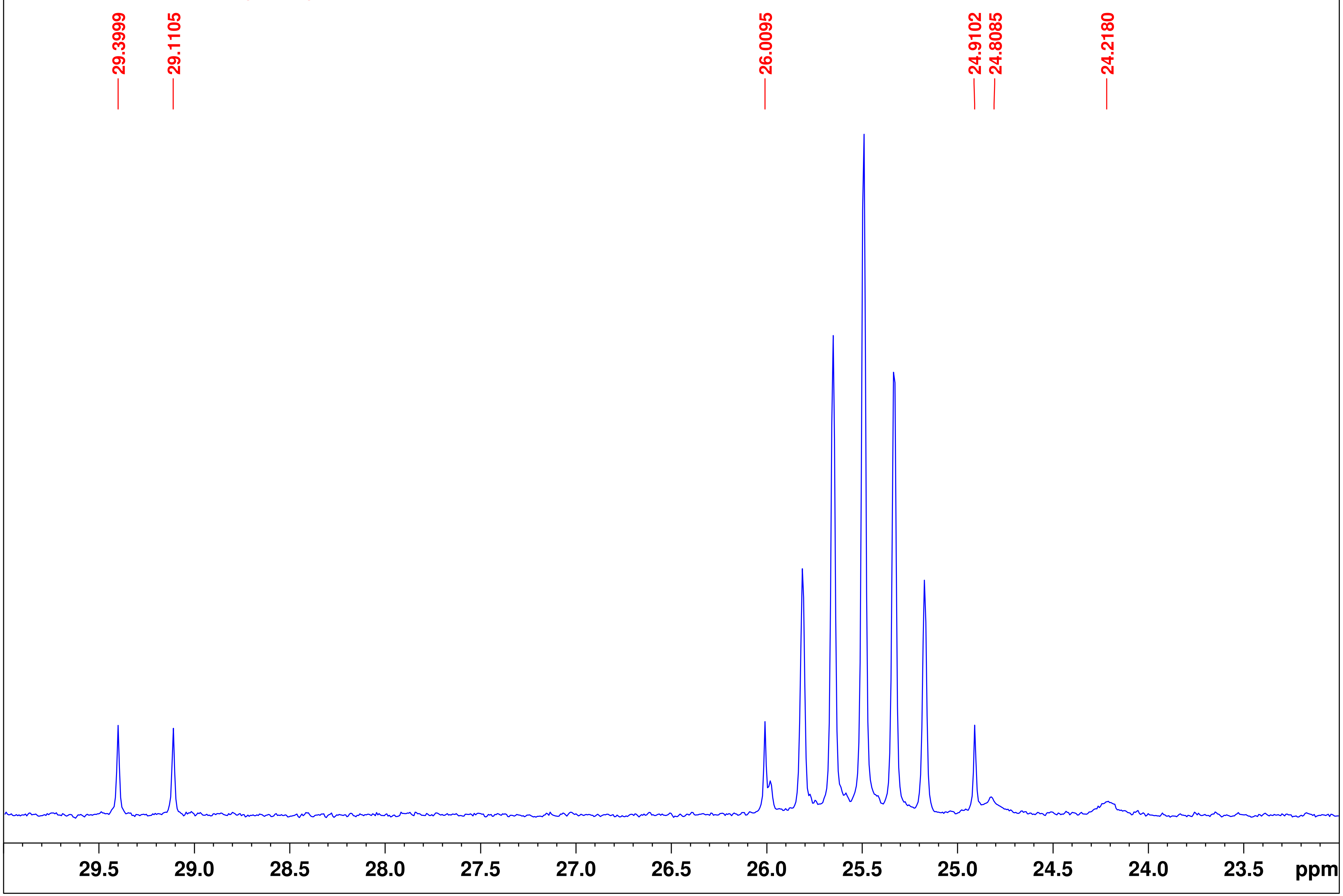

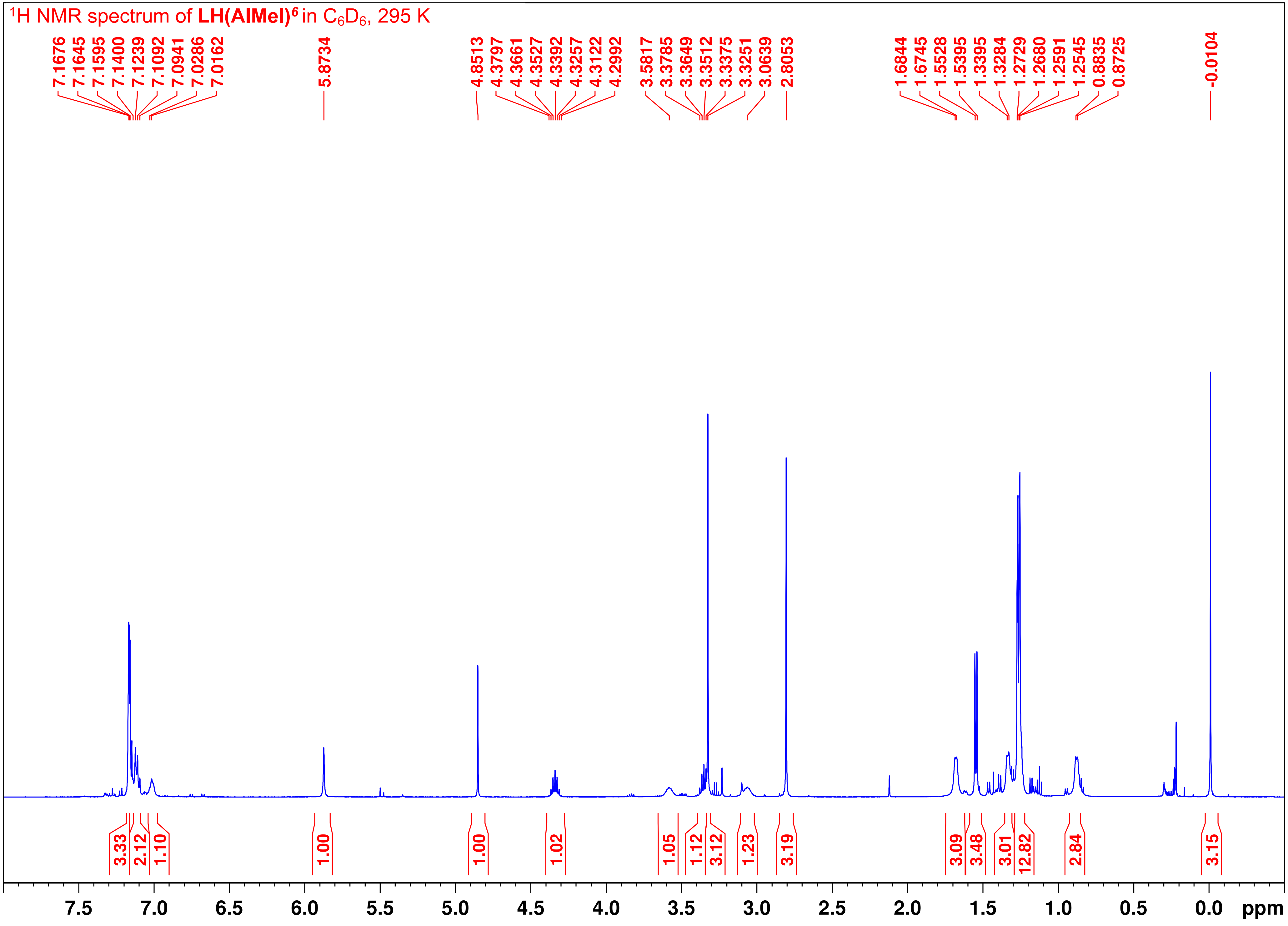

^{1}H NMR spectrum of **LH(AlMeI)^{6}** in C_6D_6, 295 K
7.1676
7.1645
7.1595
7.1400
7.1239
7.1092
7.0941
7.0286
7.0162
5.8734
4.8513
4.3797
4.3661
4.3527
4.3392
4.3257
4.3122
4.2992
3.5817
3.3785
3.3649
3.3512
3.3375
3.3251
3.0639
2.8053
1.6844
1.6745
1.5528
1.5395
1.3395
1.3284
1.2729
1.2680
1.2591
1.2545
0.8835
0.8725
-0.0104
3.33
2.12
1.10
1.00
1.00
1.02
1.05
1.12
3.12
1.23
3.19
3.09
3.48
3.01
12.82
2.84
3.15
7.5
7.0
6.5
6.0
5.5
5.0
4.5
4.0
3.5
3.0
2.5
2.0
1.5
1.0
0.5
0.0
ppm

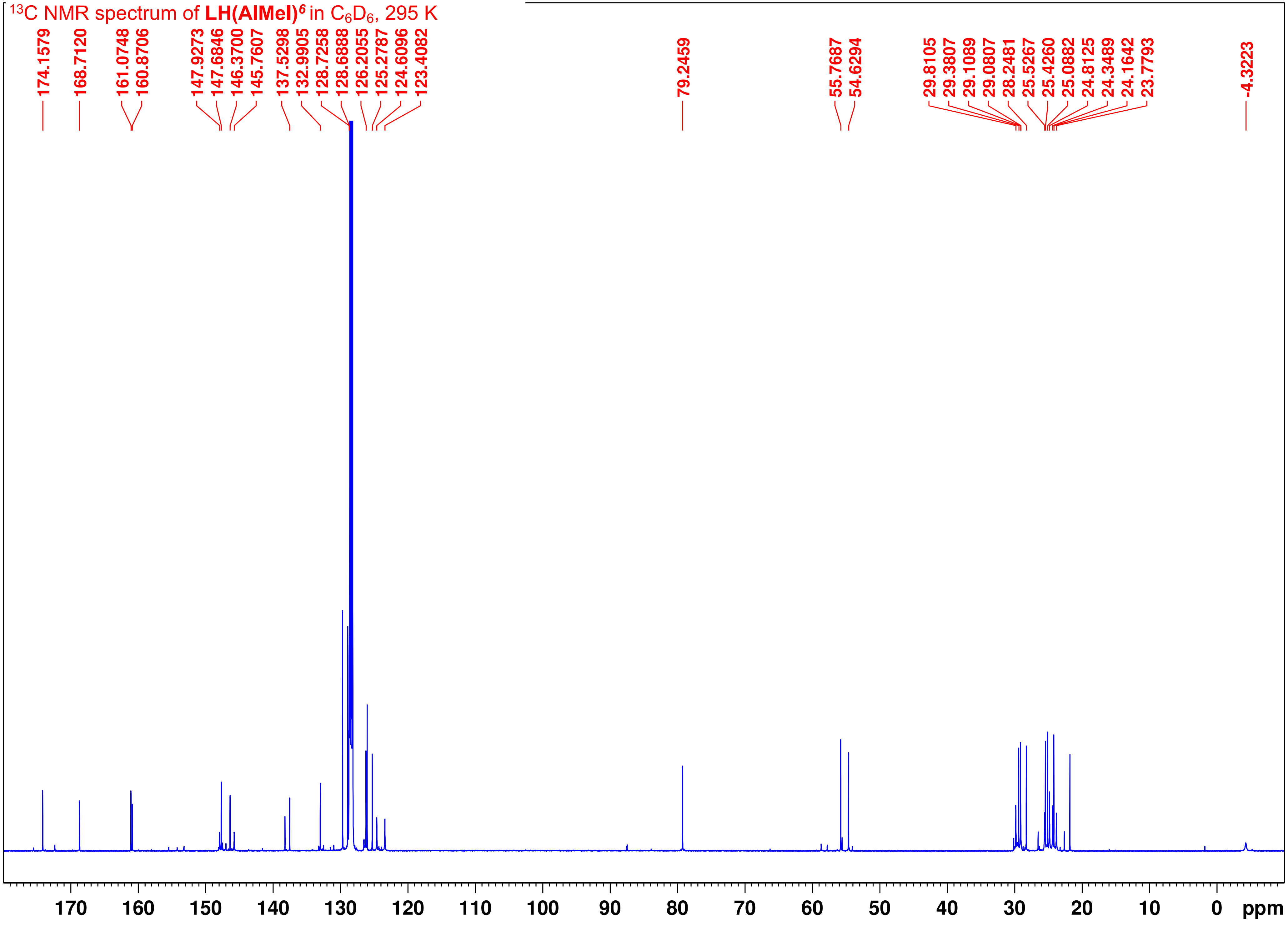

^{13}C NMR spectrum of **LH(AlMeI)**^{6} in C_6D_6, 295 K
174.1579
168.7120
161.0748
160.8706
147.9273
147.6846
146.3700
145.7607
137.5298
132.9905
128.7258
128.6888
126.2055
125.2787
124.6096
123.4082
79.2459
55.7687
54.6294
29.8105
29.3807
29.1089
29.0807
28.2481
25.5267
25.4260
25.0882
24.8125
24.3489
24.1642
23.7793
-4.3223
170
160
150
140
130
120
110
100
90
80
70
60
50
40
30
20
10
0
ppm

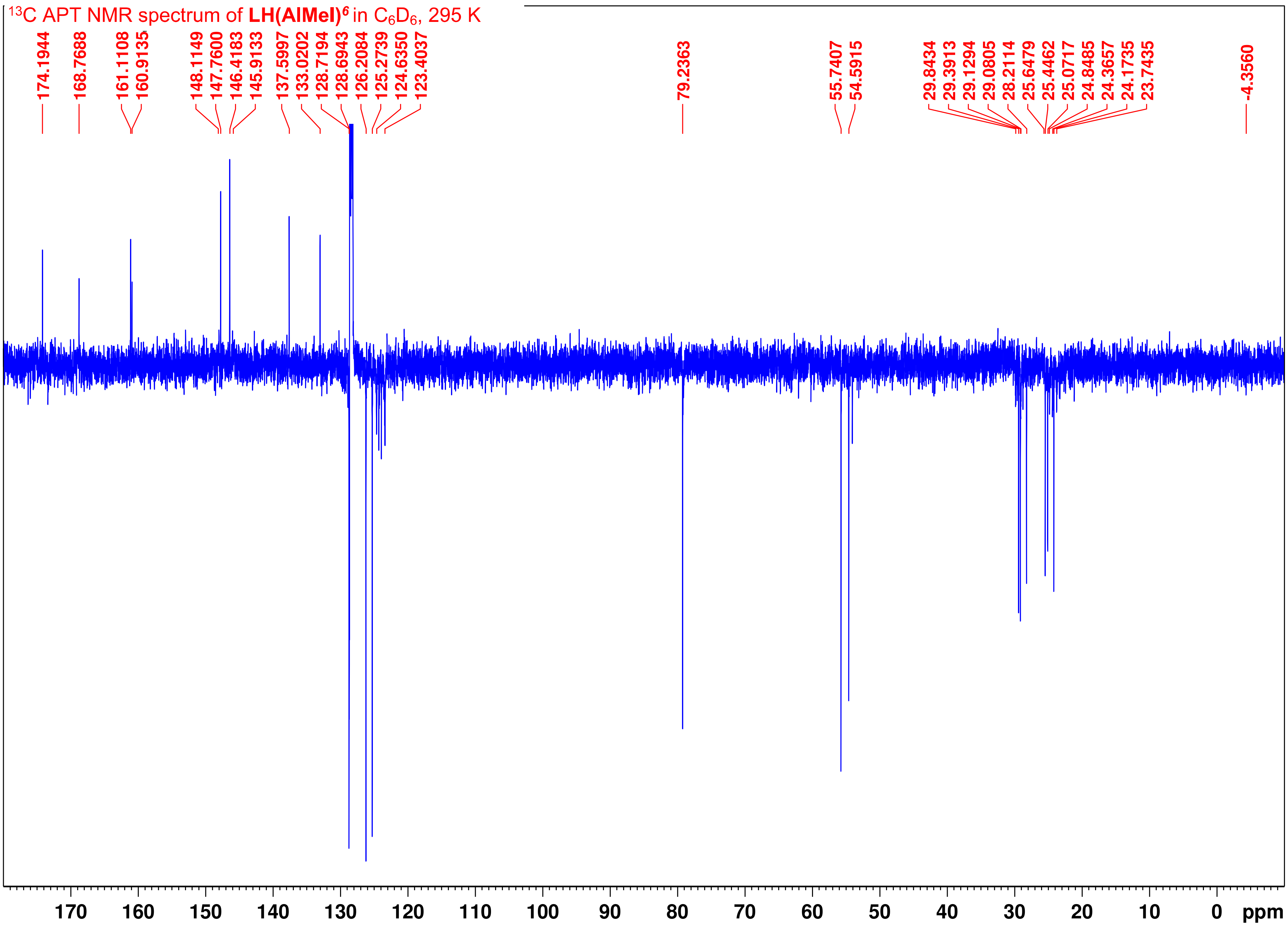

13C APT NMR spectrum of LH(AlMeI)6 in C6D6, 295 K
174.1944
168.7688
161.1108
160.9135
148.1149
147.7600
146.4183
145.9133
137.5997
133.0202
128.7194
128.6943
126.2084
125.2739
124.6350
123.4037
79.2363
55.7407
54.5915
29.8434
29.3913
29.1294
29.0805
28.2114
25.6479
25.4462
25.0717
24.8485
24.3657
24.1735
23.7435
-4.3560
170
160
150
140
130
120
110
100
90
80
70
60
50
40
30
20
10
0
ppm

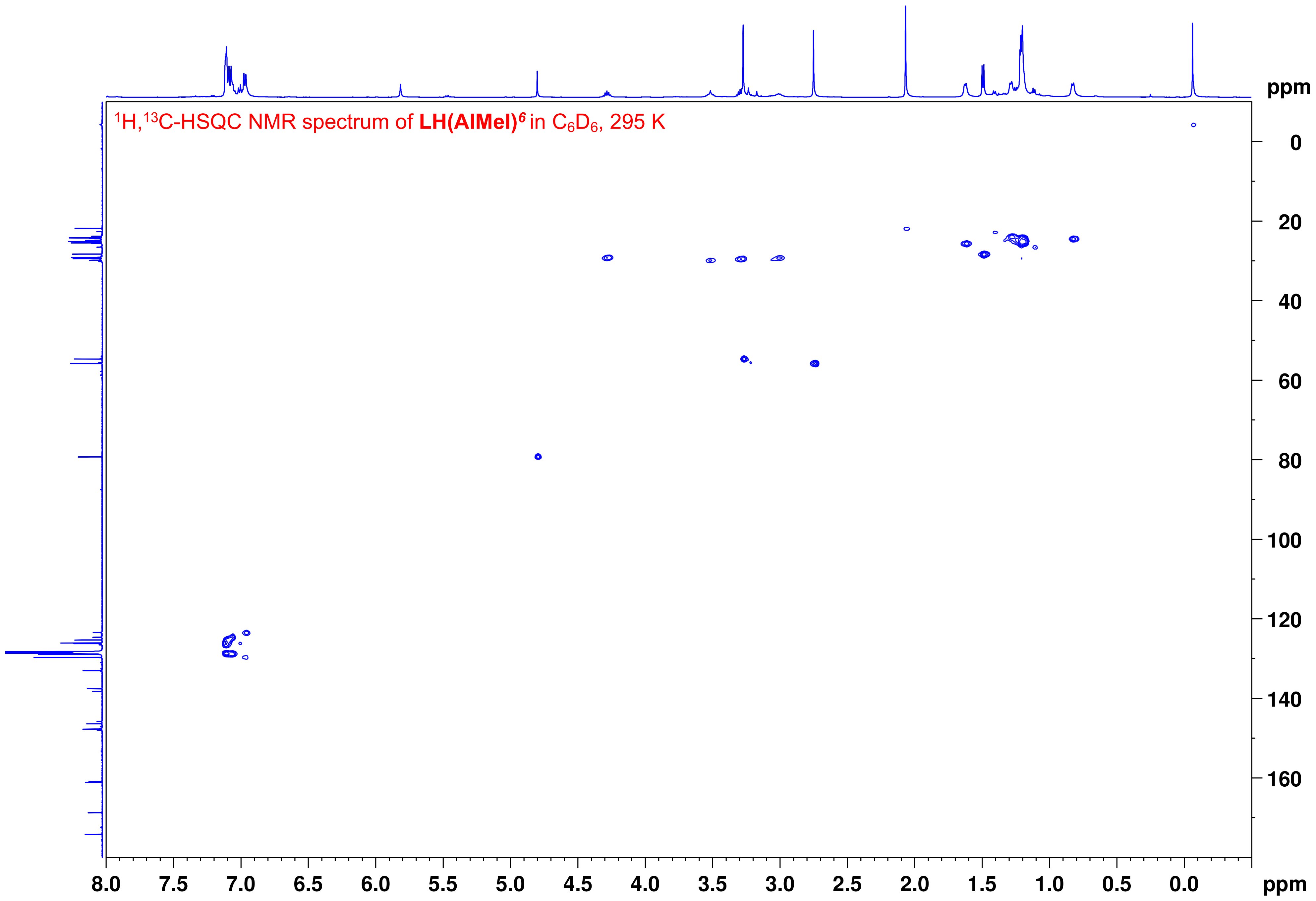

$^1H,^{13}C$-HSQC NMR spectrum of **LH(AlMeI)**^{6} in C_6D_6, 295 K
ppm
0
20
40
60
80
100
120
140
160
8.0
7.5
7.0
6.5
6.0
5.5
5.0
4.5
4.0
3.5
3.0
2.5
2.0
1.5
1.0
0.5
0.0
ppm

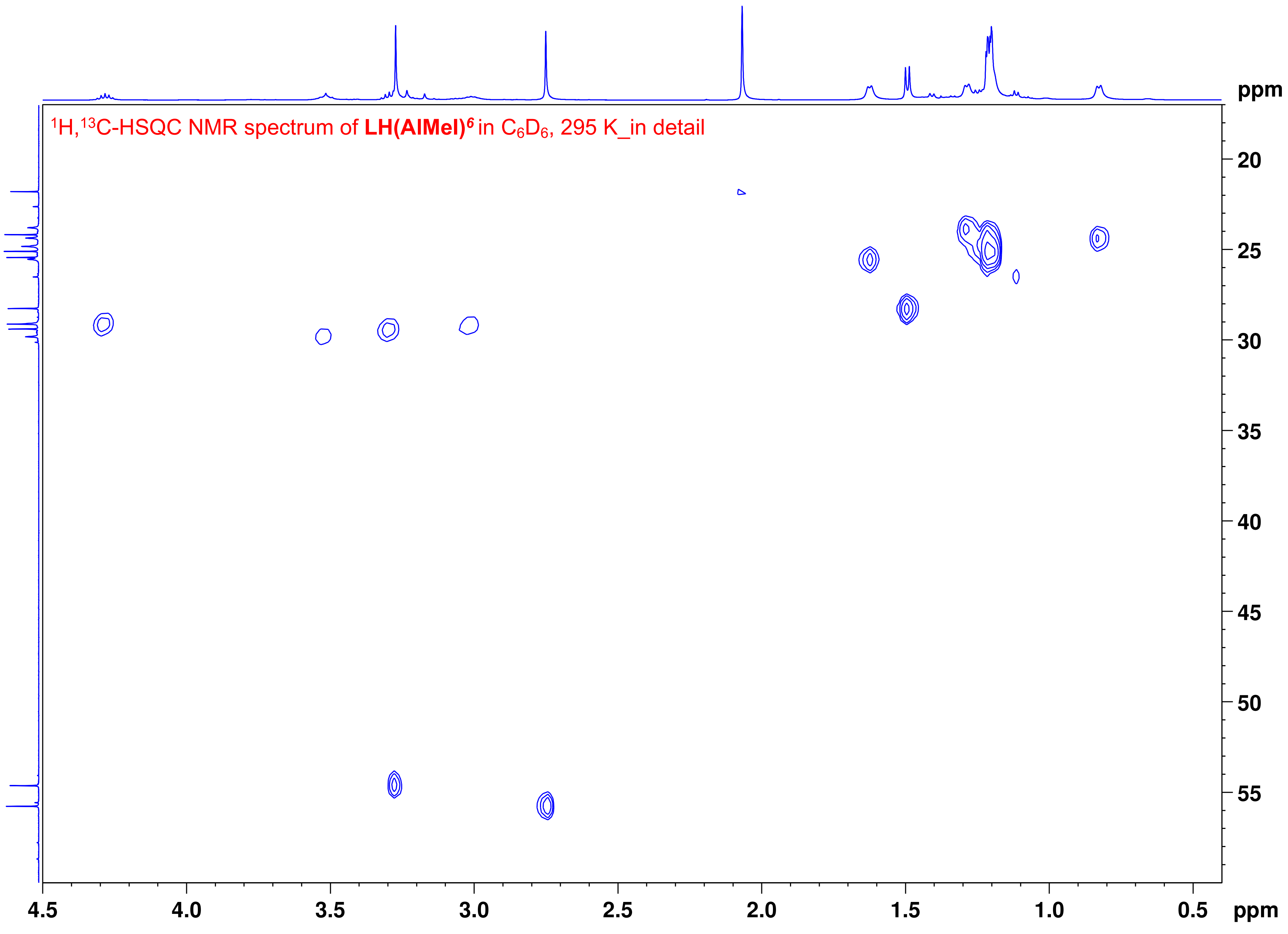

$^{1}H,^{13}C$-HSQC NMR spectrum of **LH(AlMeI)**^{6} in C_6D_6, 295 K_in detail

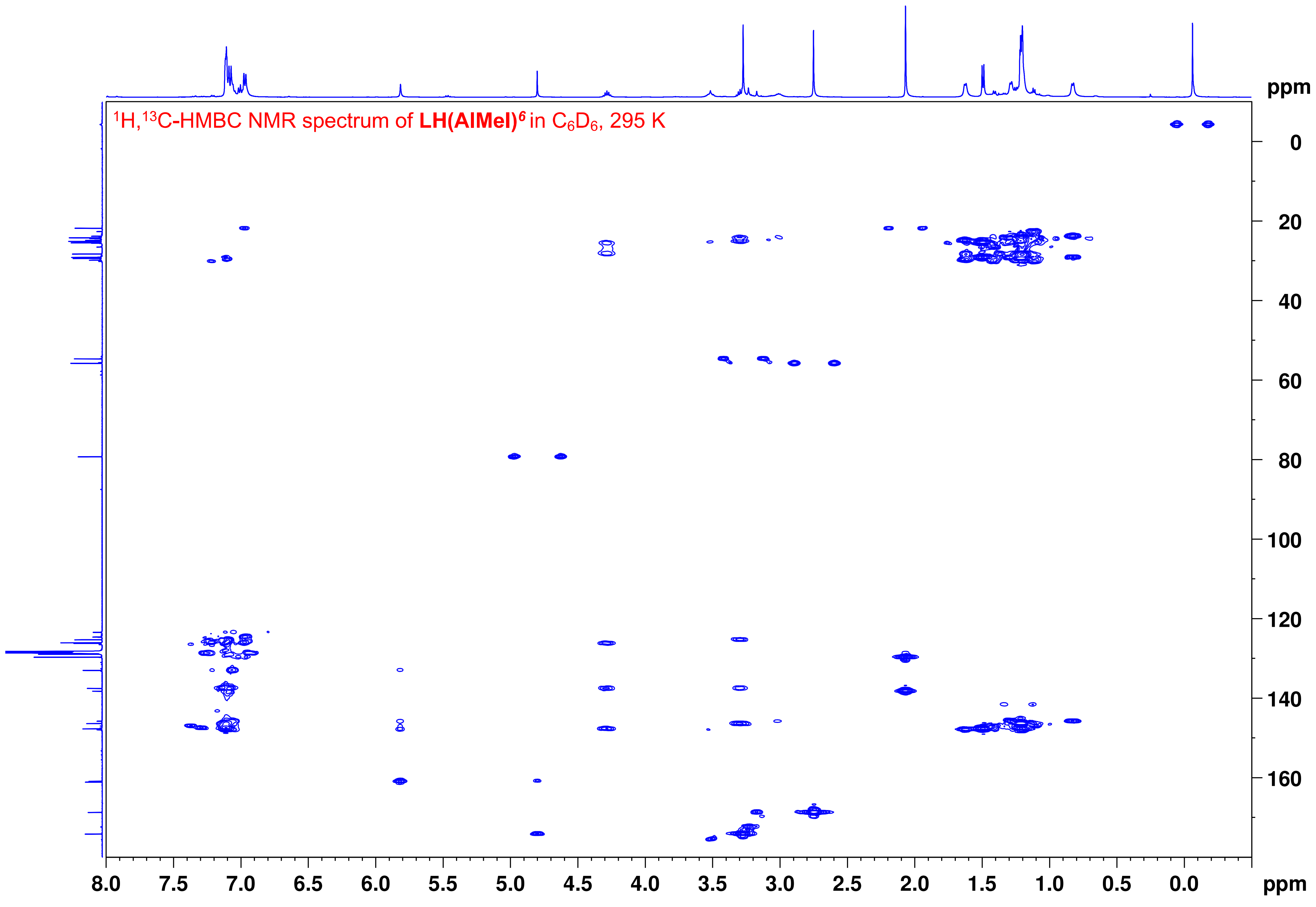

$^{1}H,^{13}C$-HMBC NMR spectrum of **LH(AlMeI)^6** in C_6D_6, 295 K
ppm
0
20
40
60
80
100
120
140
160
8.0
7.5
7.0
6.5
6.0
5.5
5.0
4.5
4.0
3.5
3.0
2.5
2.0
1.5
1.0
0.5
0.0
ppm

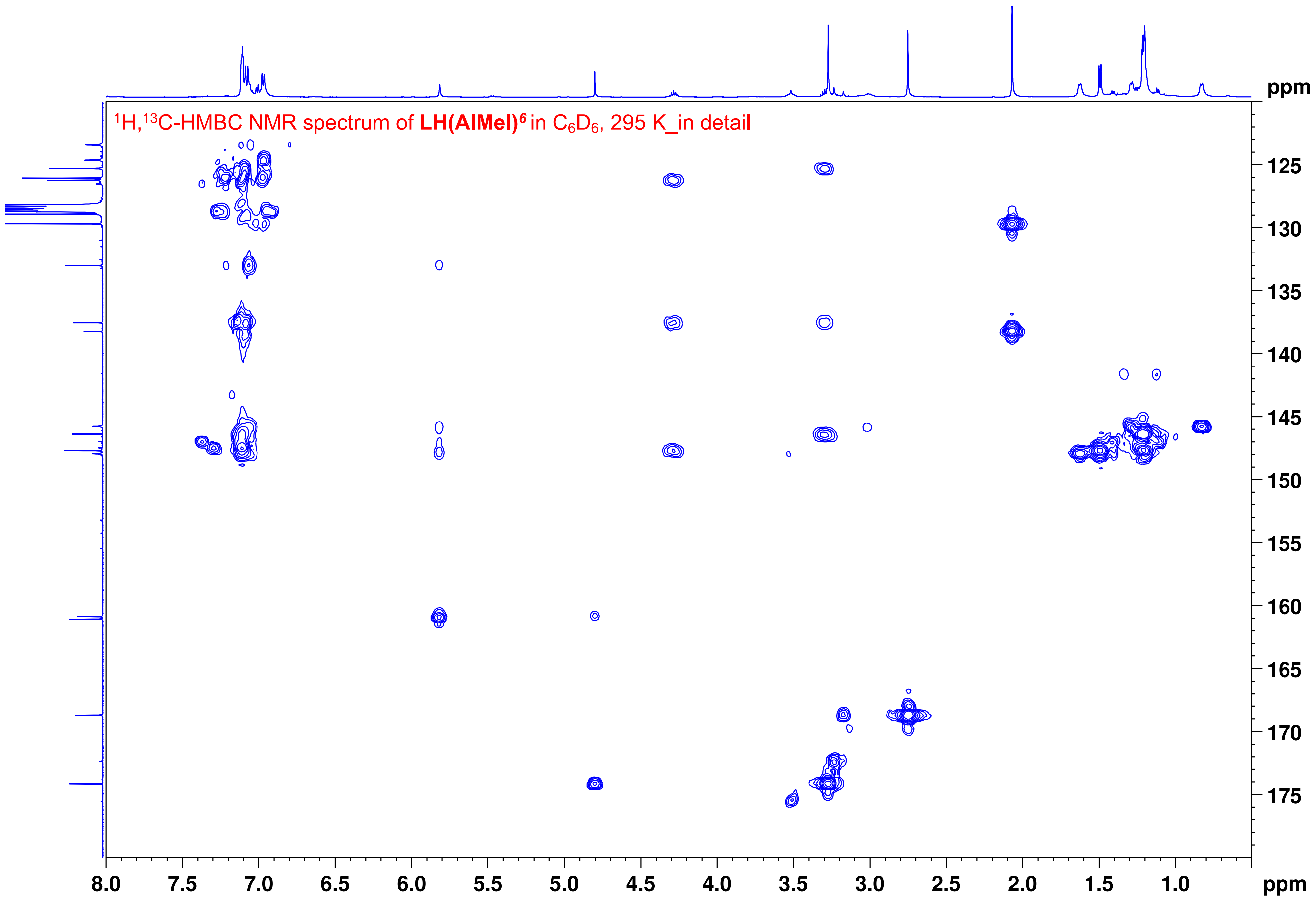

¹H,¹³C-HMBC NMR spectrum of LH(AlMeI)⁶ in C₆D₆, 295 K_in detail
ppm
125
130
135
140
145
150
155
160
165
170
175
ppm
8.0
7.5
7.0
6.5
6.0
5.5
5.0
4.5
4.0
3.5
3.0
2.5
2.0
1.5
1.0
ppm

^{1}H NMR spectrum of **LH(AlI$_2$)^{6}** in C_6D_6, 295 K

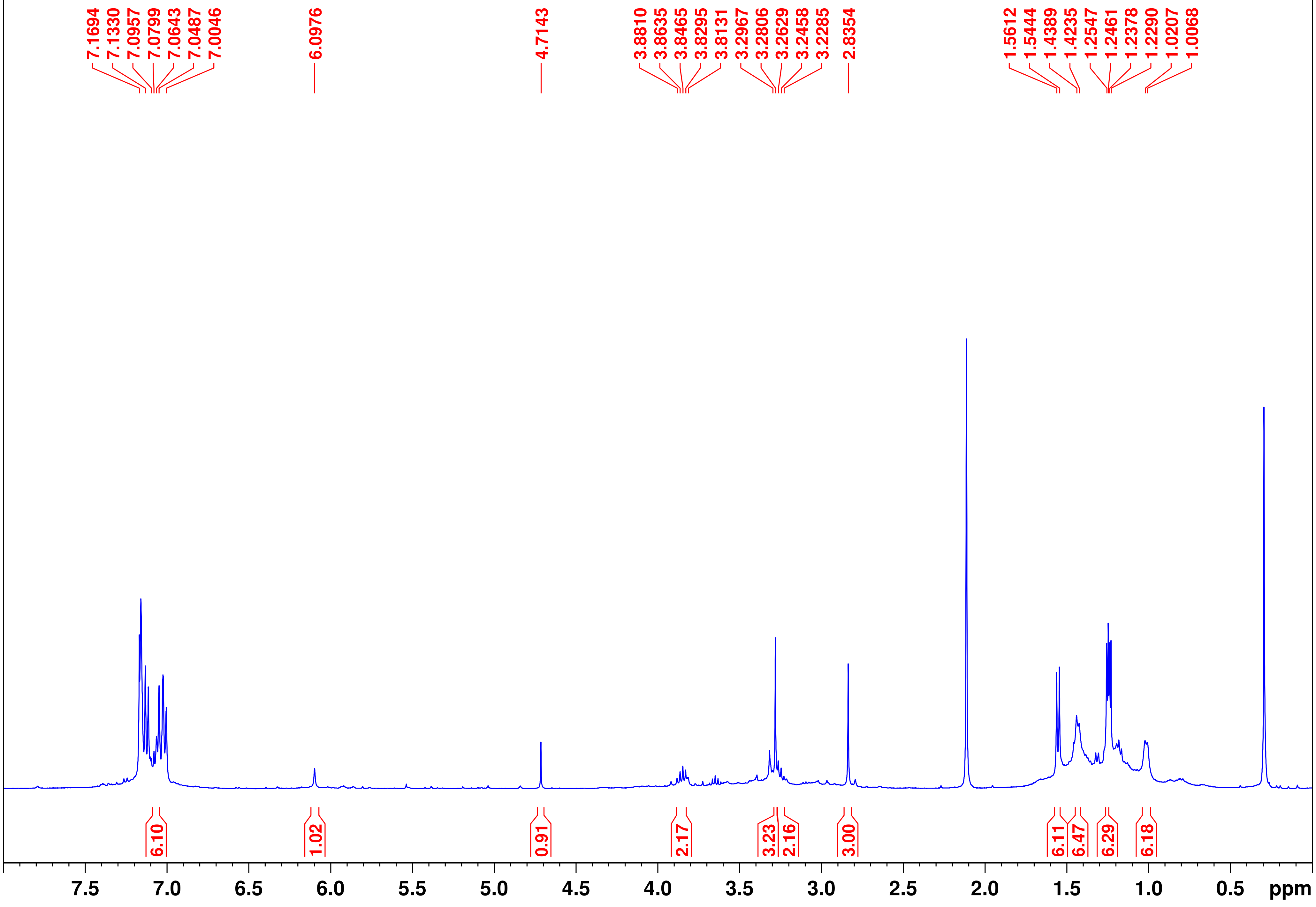

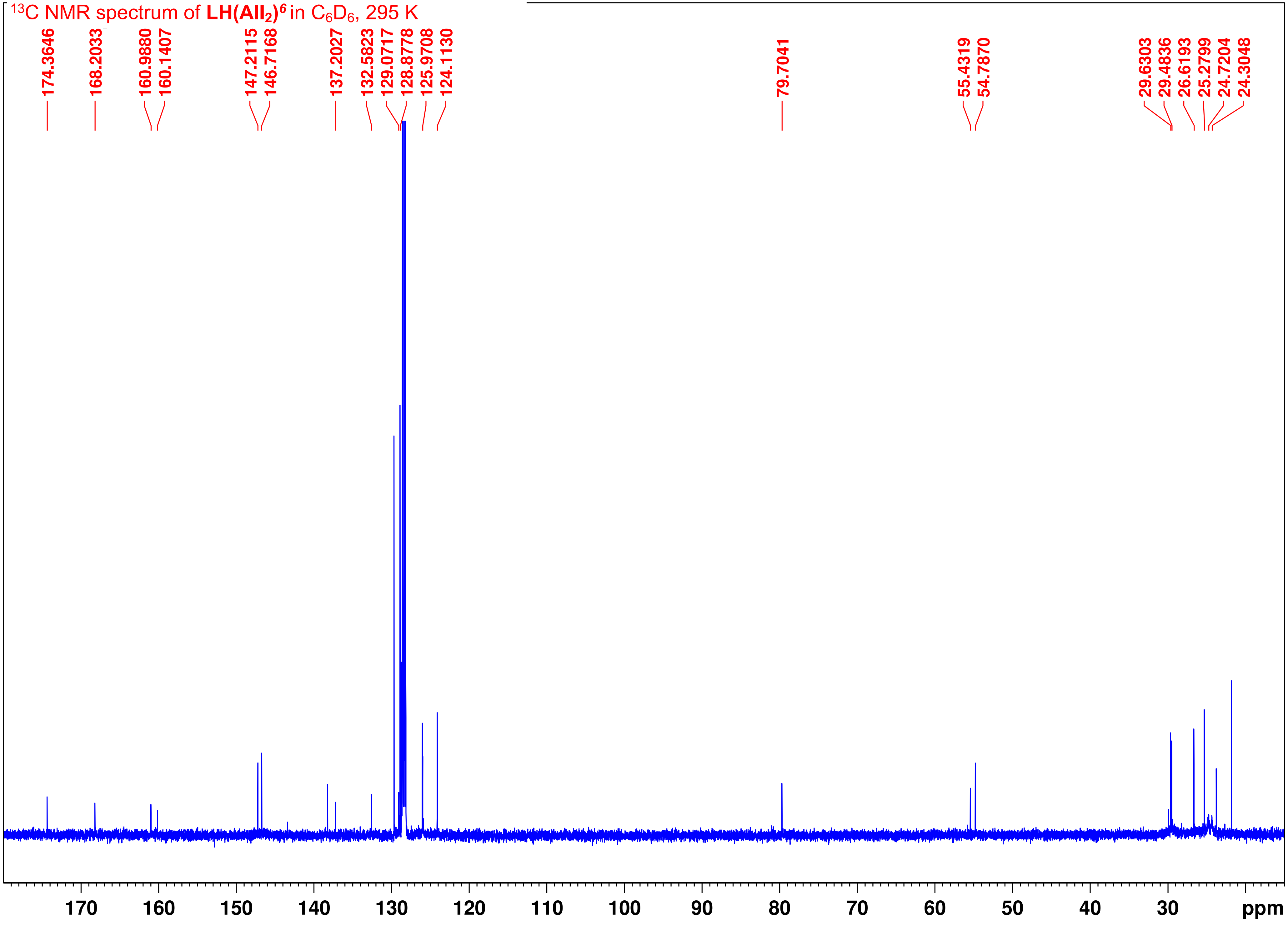

^{13}C NMR spectrum of **LH(AlI$_2$)**^{6} in C_6D_6, 295 K
174.3646
168.2033
160.9880
160.1407
147.2115
146.7168
137.2027
132.5823
129.0717
128.8778
125.9708
124.1130
79.7041
55.4319
54.7870
29.6303
29.4836
26.6193
25.2799
24.7204
24.3048
170
160
150
140
130
120
110
100
90
80
70
60
50
40
30
ppm

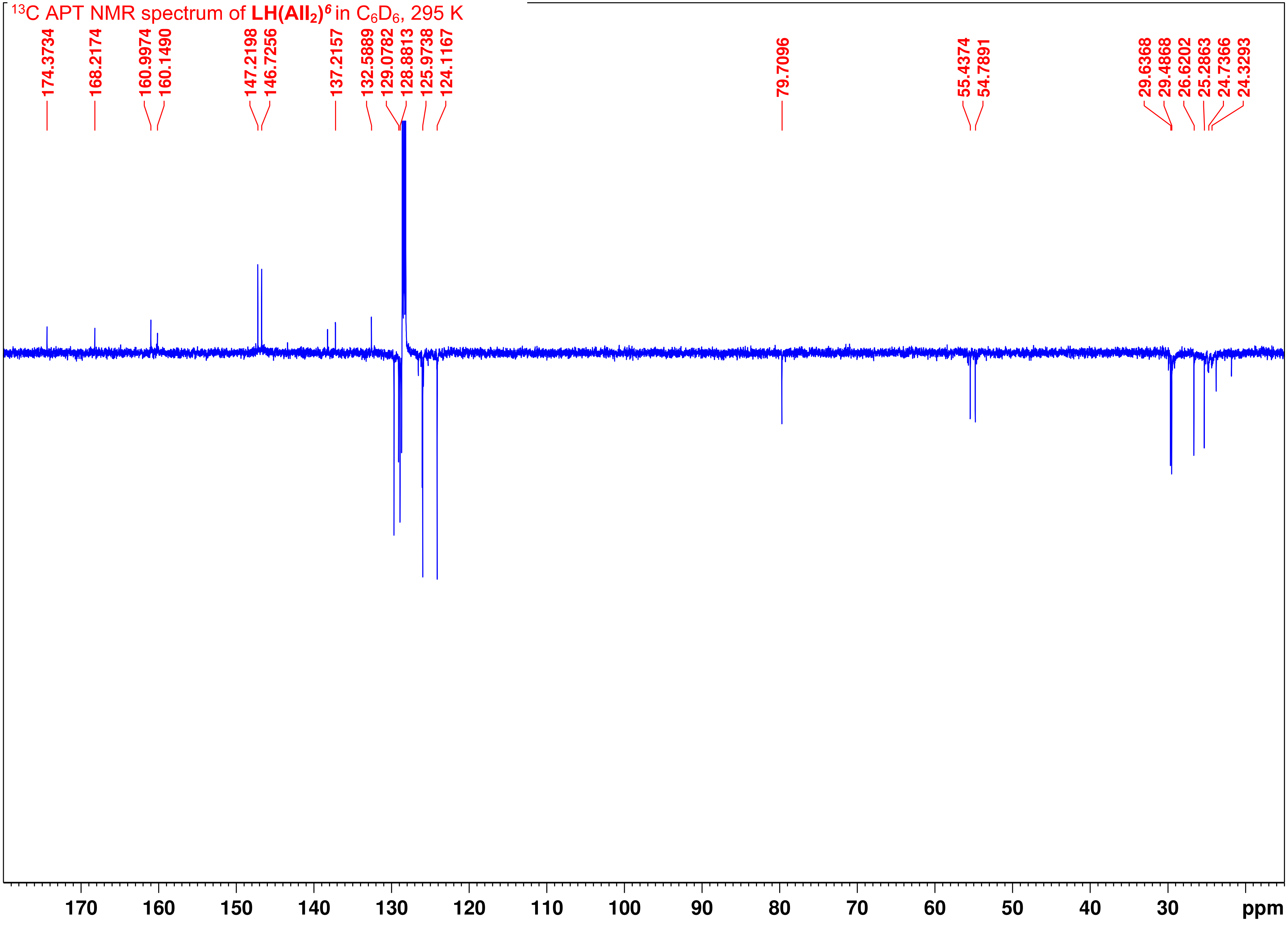
^{13}C APT NMR spectrum of **LH(AlI$_2$)^6** in C_6D_6, 295 K
174.3734
168.2174
160.9974
160.1490
147.2198
146.7256
137.2157
132.5889
129.0782
128.8813
125.9738
124.1167
79.7096
55.4374
54.7891
29.6368
29.4868
26.6202
25.2863
24.7366
24.3293
170
160
150
140
130
120
110
100
90
80
70
60
50
40
30
ppm

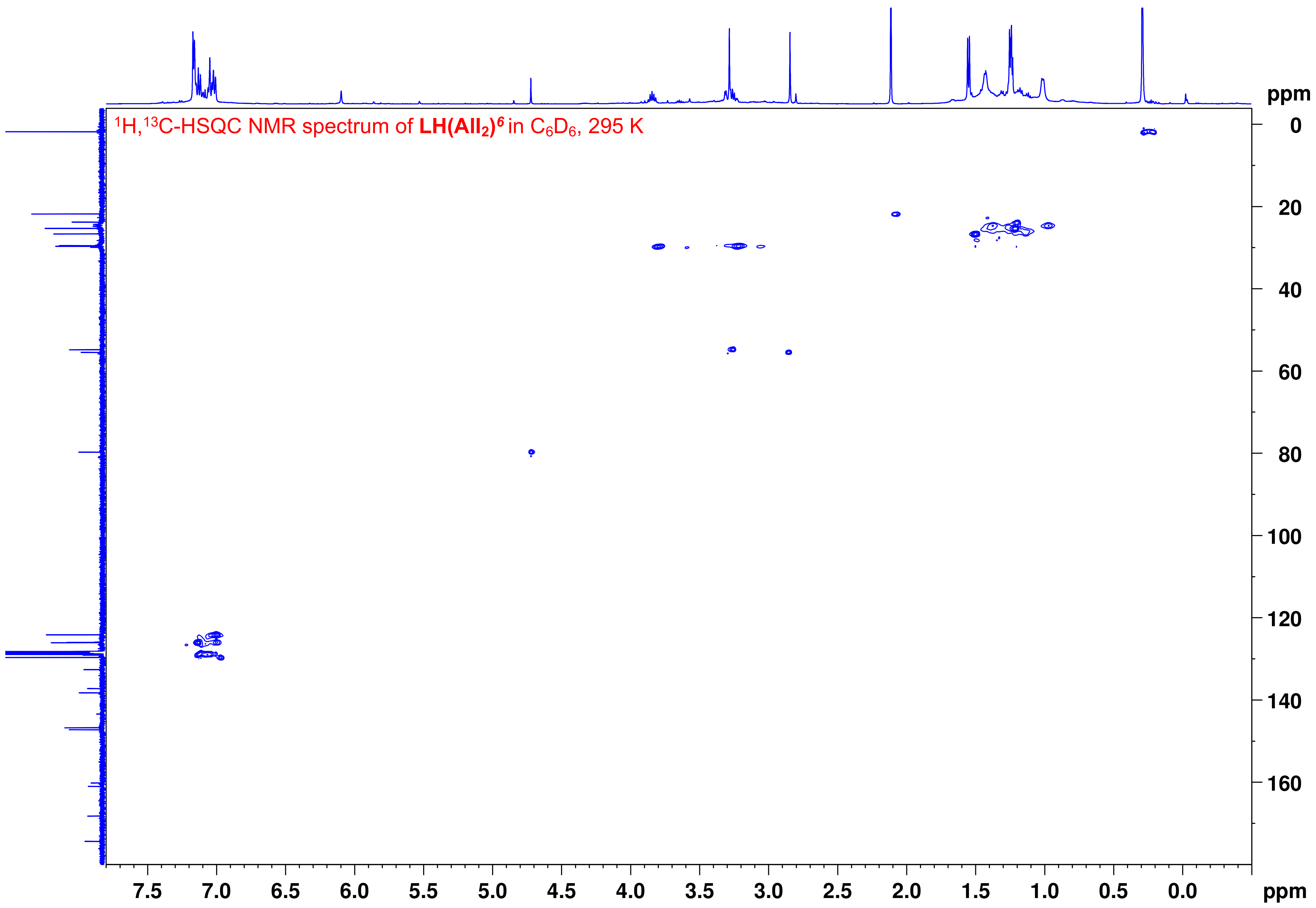

^1H,^{13}C-HSQC NMR spectrum of **LH(AlI$_2$)*6*** in C_6D_6, 295 K
ppm
0
20
40
60
80
100
120
140
160
7.5
7.0
6.5
6.0
5.5
5.0
4.5
4.0
3.5
3.0
2.5
2.0
1.5
1.0
0.5
0.0
ppm

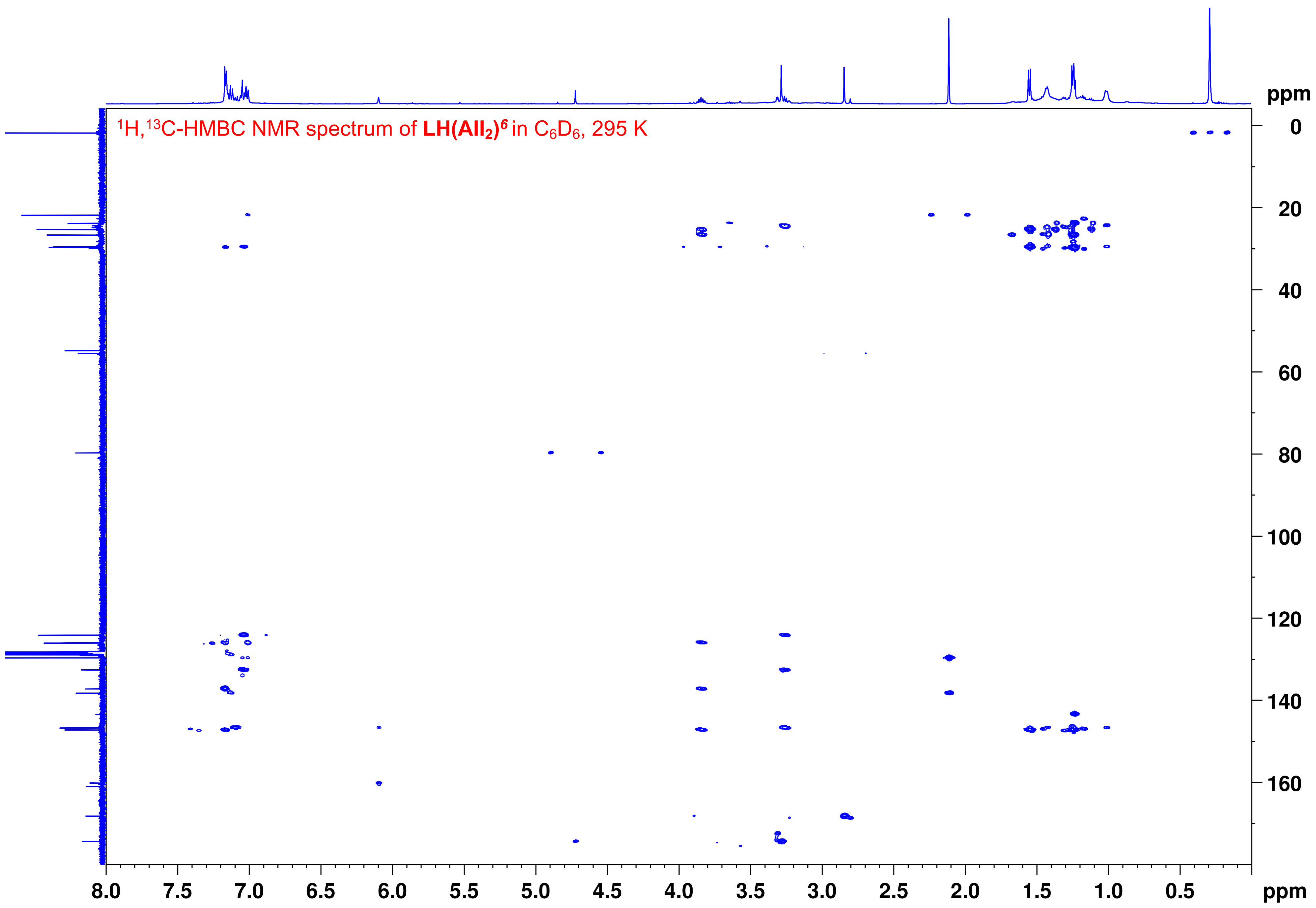
$^{1}H,^{13}C$-HMBC NMR spectrum of **LH(AlI$_2$)^6** in C_6D_6, 295 K
ppm
0
20
40
60
80
100
120
140
160
8.0
7.5
7.0
6.5
6.0
5.5
5.0
4.5
4.0
3.5
3.0
2.5
2.0
1.5
1.0
0.5
ppm

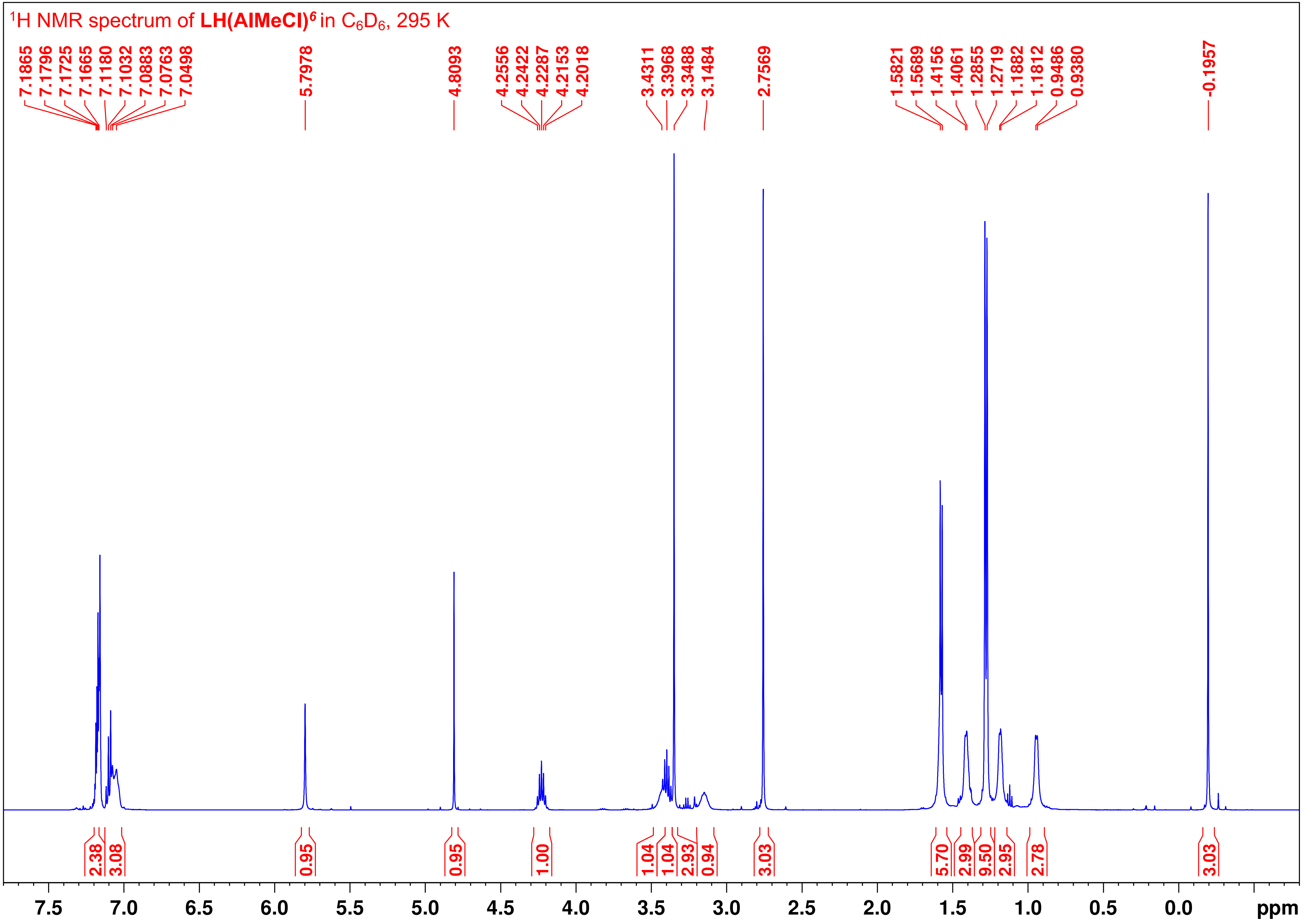

^{1}H NMR spectrum of **LH(AlMeCl)^6** in C_6D_6, 295 K
7.1865
7.1796
7.1725
7.1665
7.1180
7.1032
7.0883
7.0763
7.0498
5.7978
4.8093
4.2556
4.2422
4.2287
4.2153
4.2018
3.4311
3.3968
3.3488
3.1484
2.7569
1.5821
1.5689
1.4156
1.4061
1.2855
1.2719
1.1882
1.1812
0.9486
0.9380
-0.1957
2.38
3.08
0.95
0.95
1.00
1.04
1.04
2.93
0.94
3.03
5.70
2.99
9.50
2.95
2.78
3.03
7.5
7.0
6.5
6.0
5.5
5.0
4.5
4.0
3.5
3.0
2.5
2.0
1.5
1.0
0.5
0.0
ppm

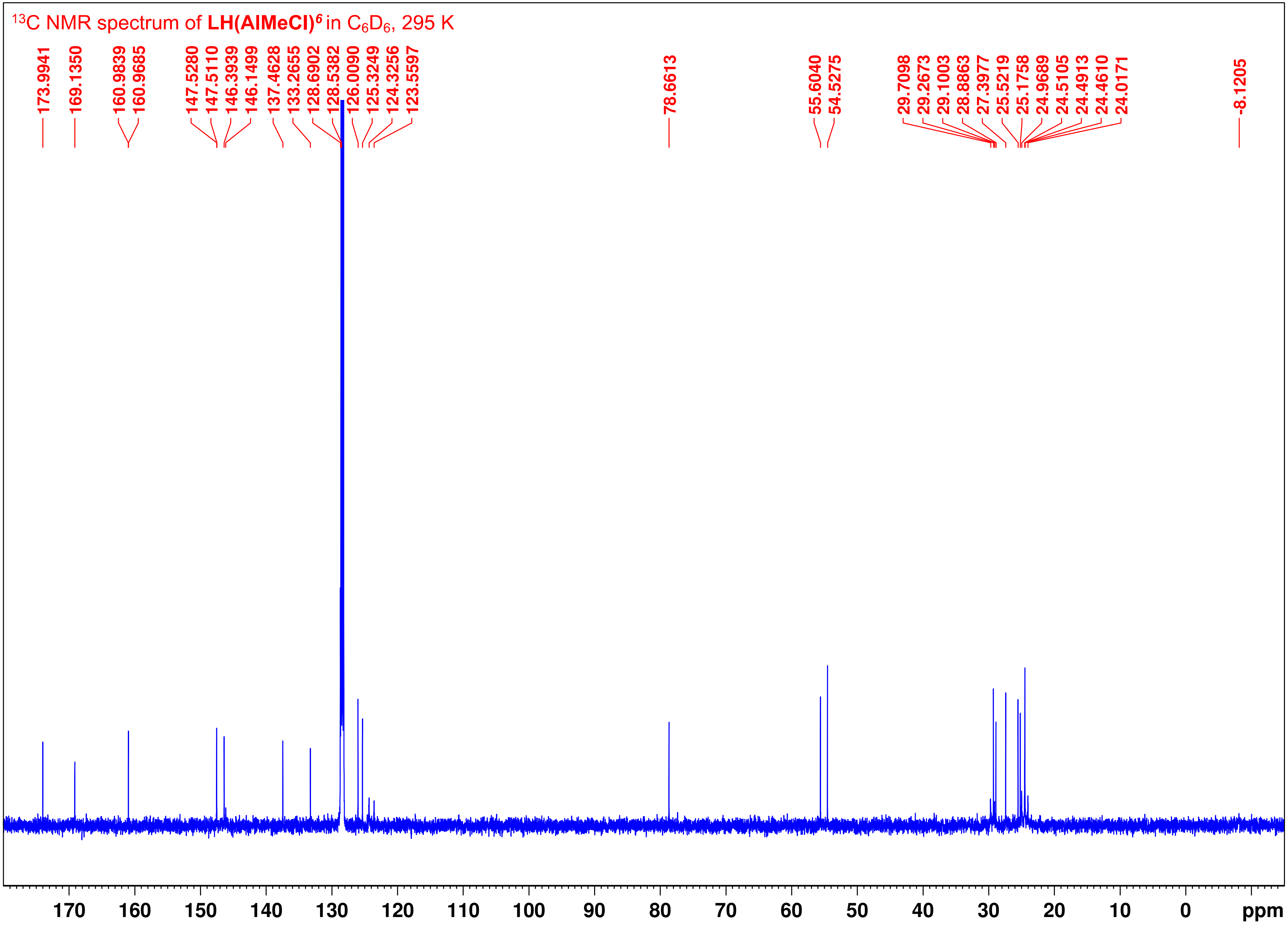
^{13}C NMR spectrum of **LH(AlMeCl)^{6}** in C_6D_6, 295 K
173.9941
169.1350
160.9839
160.9685
147.5280
147.5110
146.3939
146.1499
137.4628
133.2655
128.6902
128.5382
126.0090
125.3249
124.3256
123.5597
78.6613
55.6040
54.5275
29.7098
29.2673
29.1003
28.8863
27.3977
25.5219
25.1758
24.9689
24.5105
24.4913
24.4610
24.0171
-8.1205
170
160
150
140
130
120
110
100
90
80
70
60
50
40
30
20
10
0
ppm

^{13}C NMR spectrum of **LH(AlMeCl)^{6}** in C_6D_6, 295 K_in detail

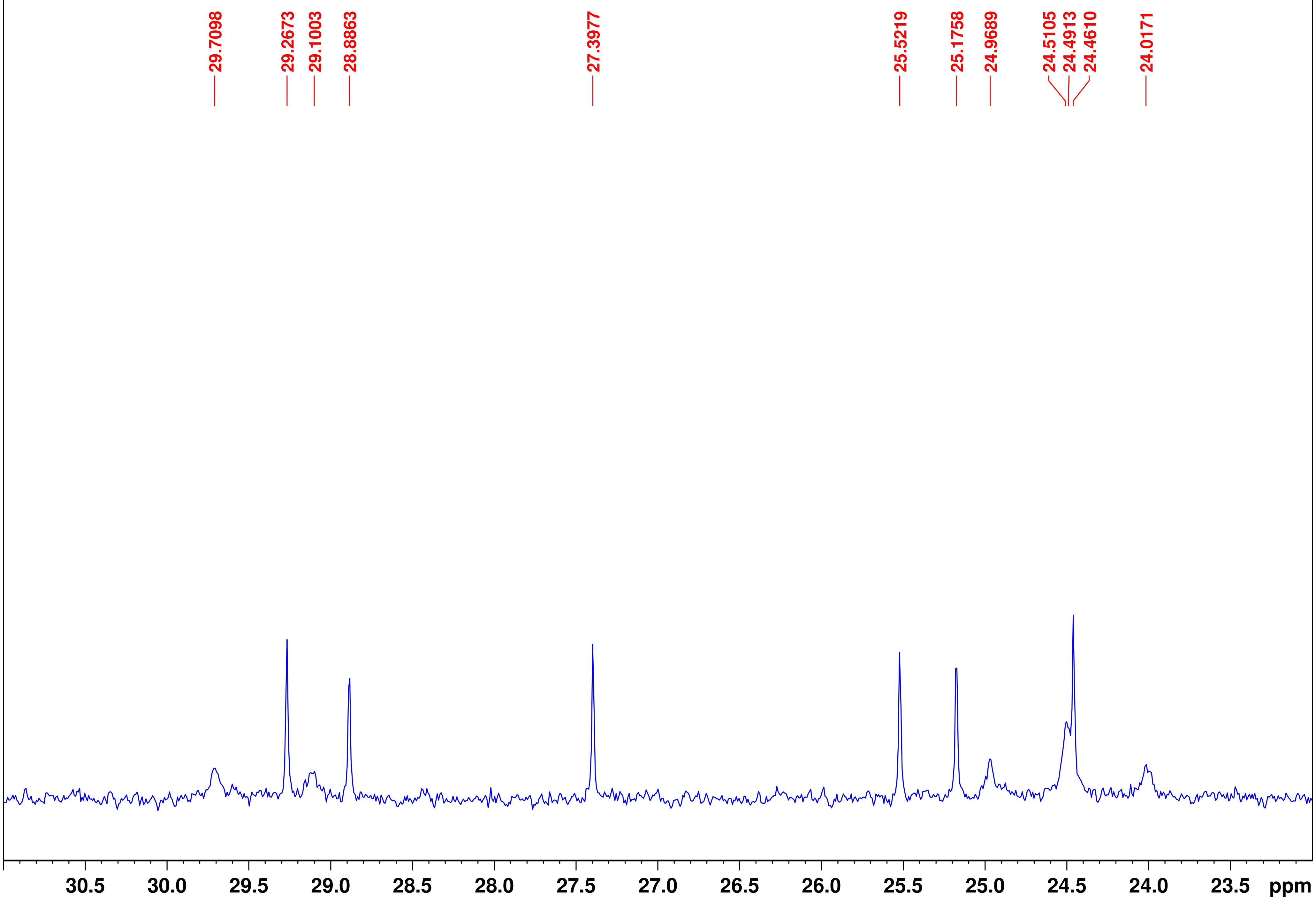

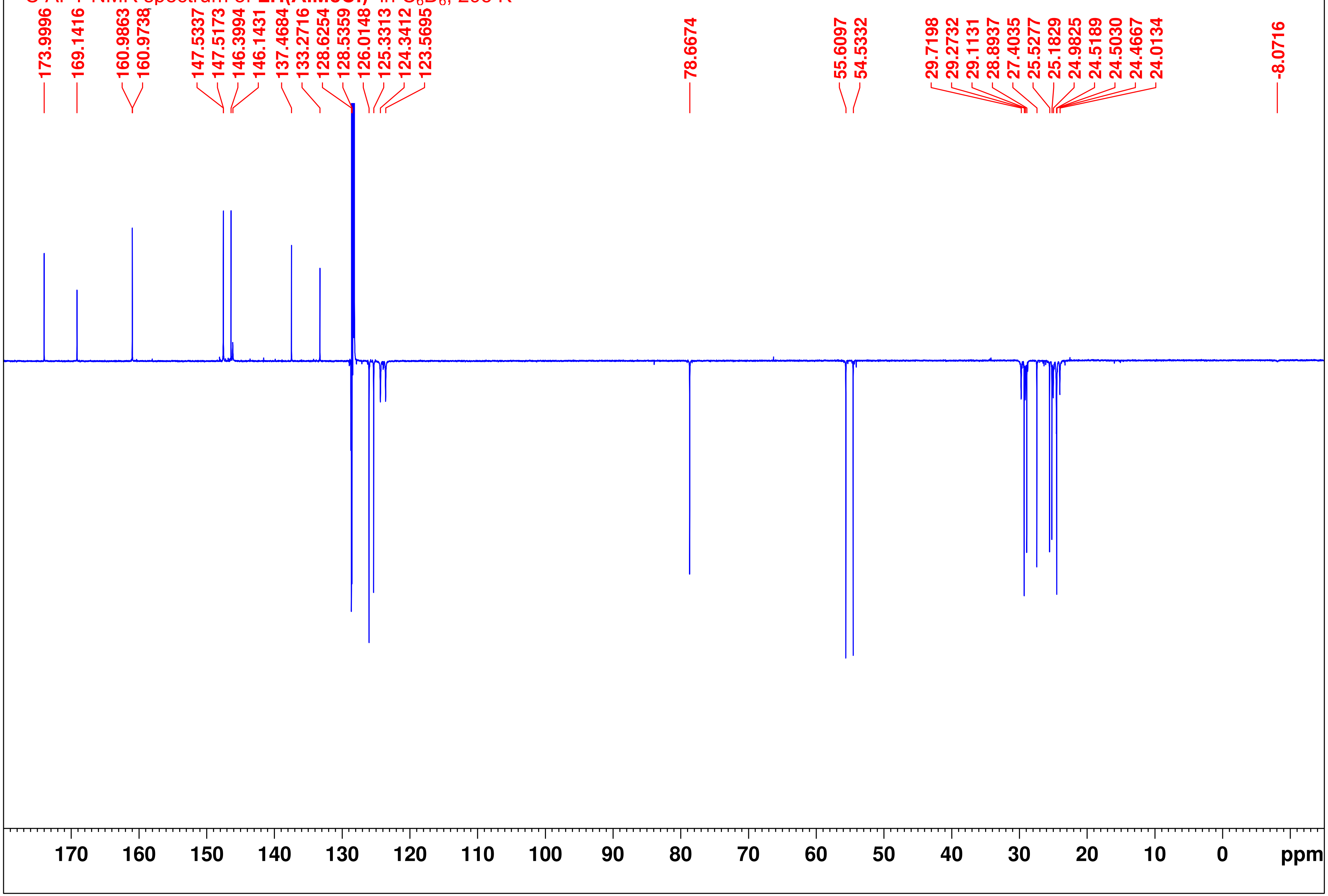

^{13}C APT NMR spectrum of **LH(AlMeCl)*6*** in C_6D_6, 295 K

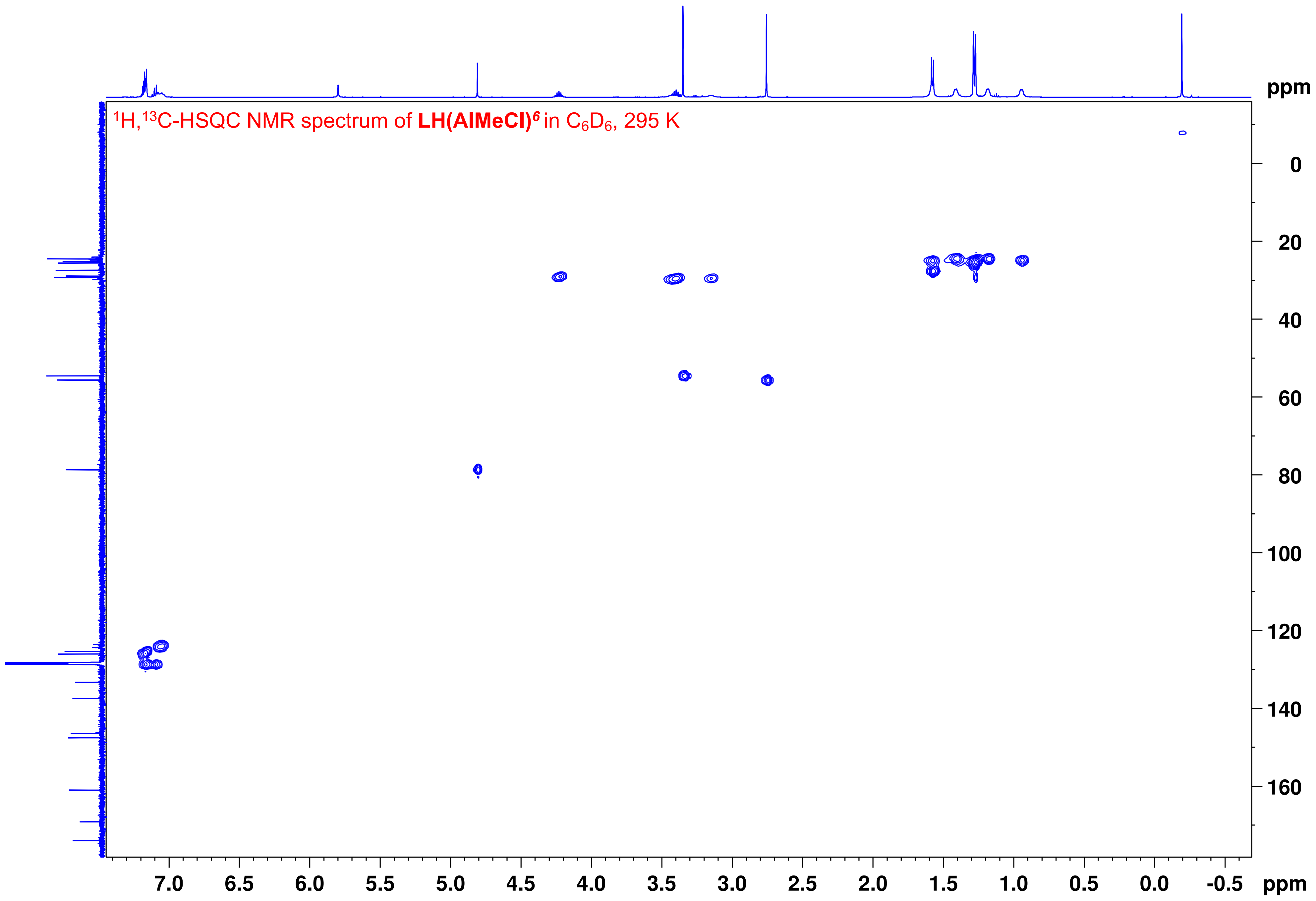

$^{1}H,^{13}C$-HSQC NMR spectrum of **LH(AlMeCl)**^{6} in C_6D_6, 295 K
ppm
0
20
40
60
80
100
120
140
160
7.0
6.5
6.0
5.5
5.0
4.5
4.0
3.5
3.0
2.5
2.0
1.5
1.0
0.5
0.0
-0.5
ppm

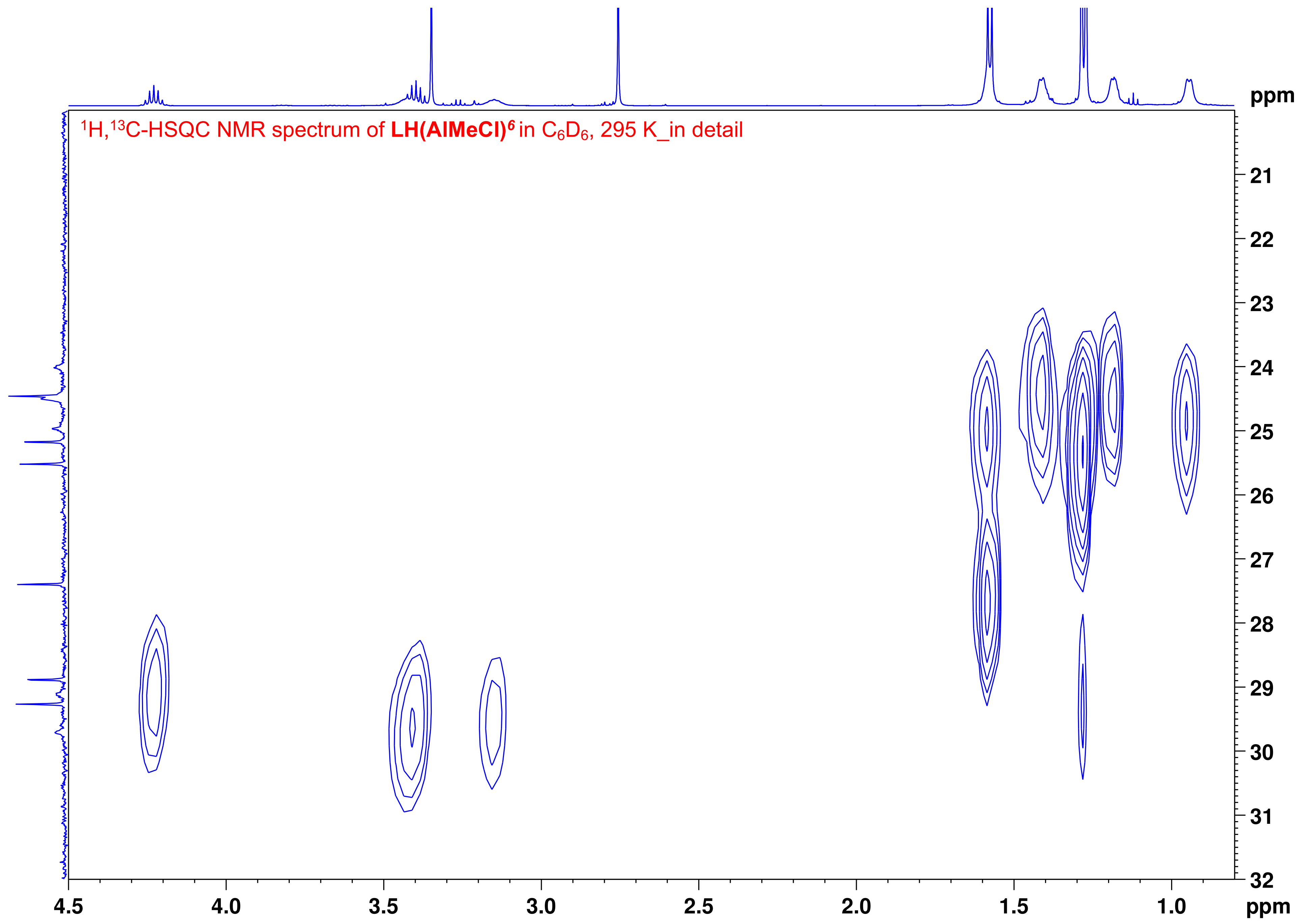

$^{1}H,^{13}C$-HSQC NMR spectrum of **LH(AlMeCl)^{6}** in C_6D_6, 295 K_in detail
ppm
21
22
23
24
25
26
27
28
29
30
31
32
4.5
4.0
3.5
3.0
2.5
2.0
1.5
1.0
ppm

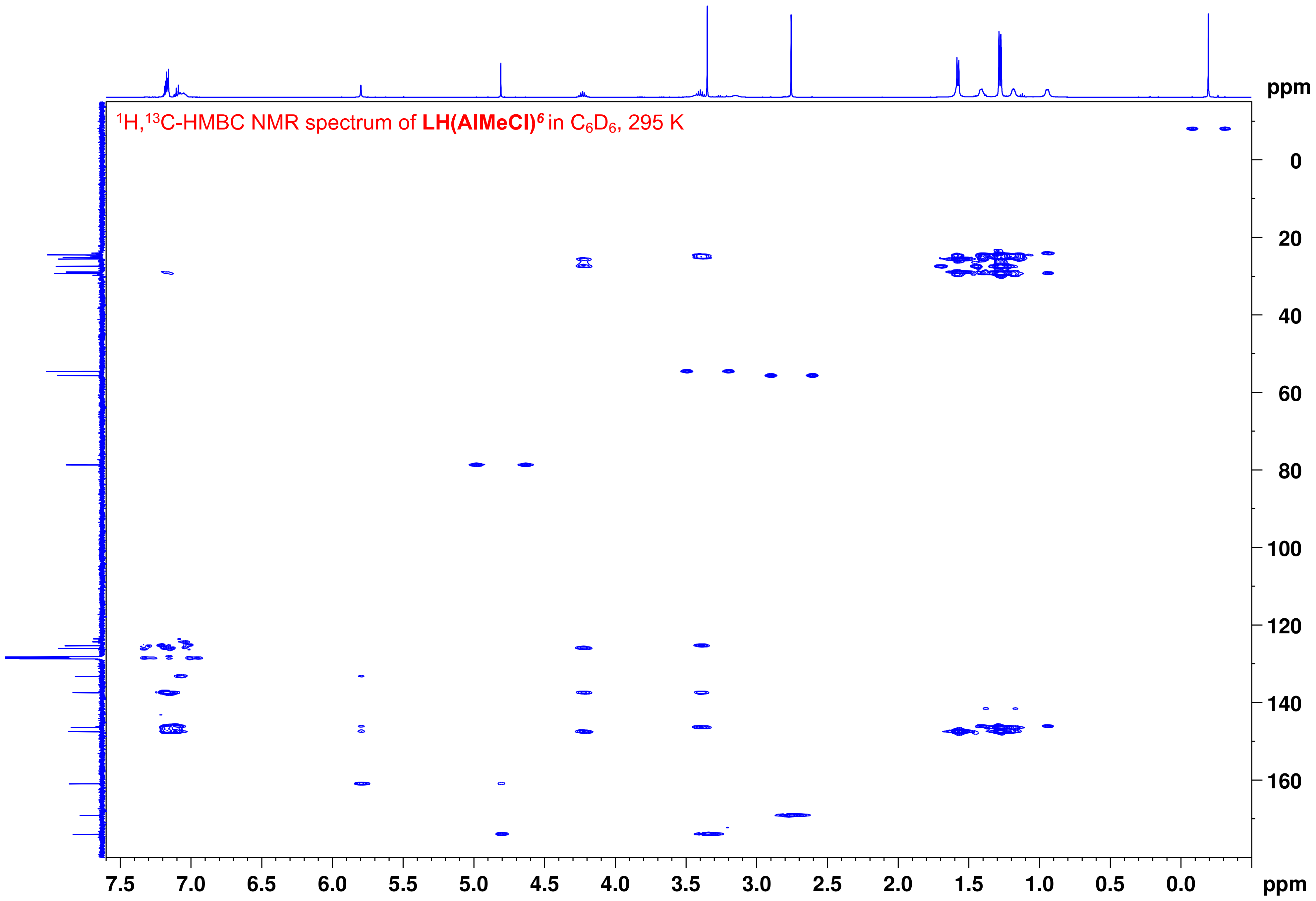

$^{1}H,^{13}C$-HMBC NMR spectrum of **LH(AlMeCl)**[6] in C_6D_6, 295 K
ppm
7.5 7.0 6.5 6.0 5.5 5.0 4.5 4.0 3.5 3.0 2.5 2.0 1.5 1.0 0.5 0.0
0 20 40 60 80 100 120 140 160

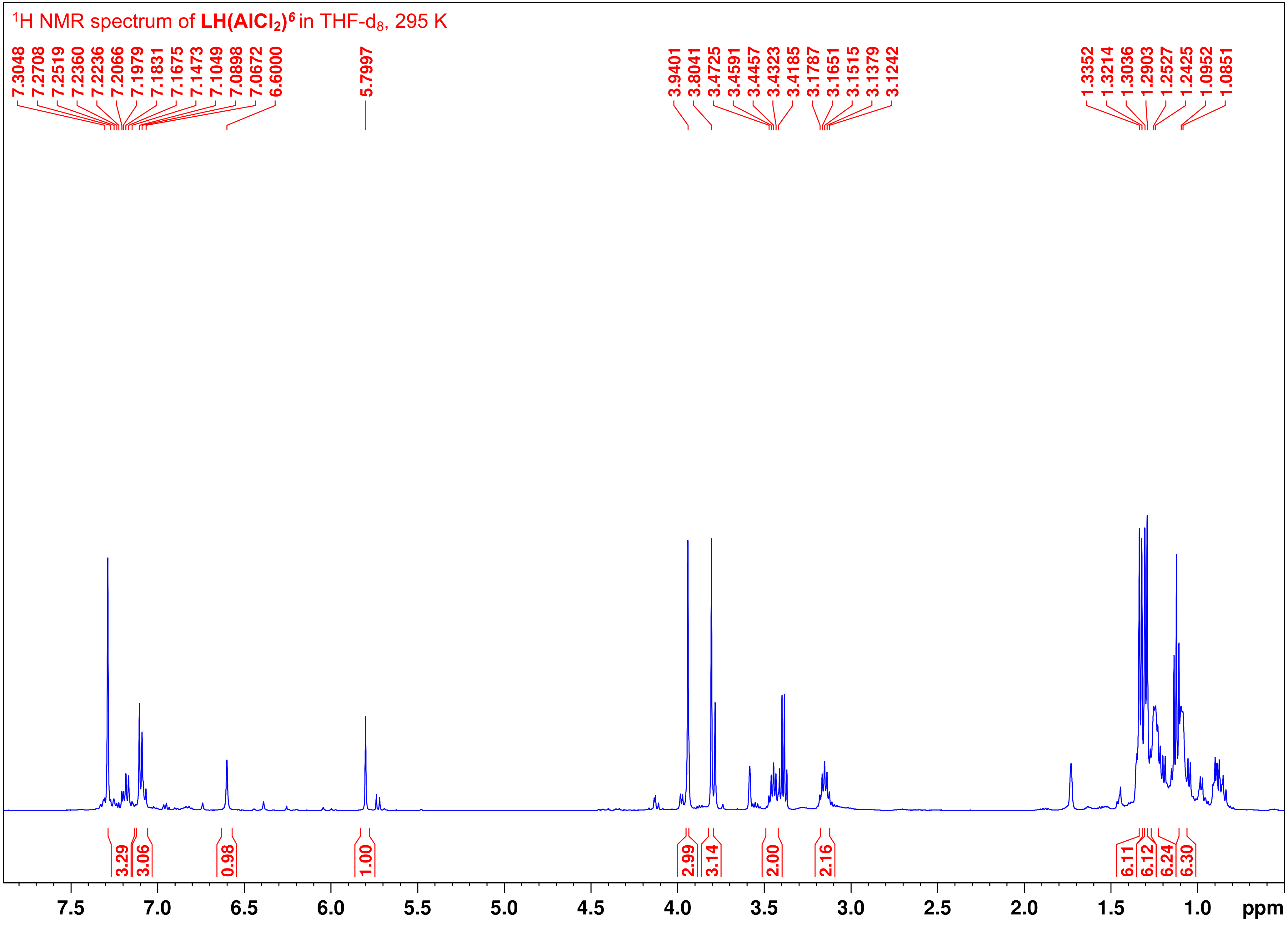

^{1}H NMR spectrum of **LH(AlCl$_2$)^6** in THF-d$_8$, 295 K
7.3048
7.2708
7.2519
7.2360
7.2236
7.2066
7.1979
7.1831
7.1675
7.1473
7.1049
7.0898
7.0672
6.6000
5.7997
3.9401
3.8041
3.4725
3.4591
3.4457
3.4323
3.4185
3.1787
3.1651
3.1515
3.1379
3.1242
1.3352
1.3214
1.3036
1.2903
1.2527
1.2425
1.0952
1.0851
3.29
3.06
0.98
1.00
2.99
3.14
2.00
2.16
6.11
6.12
6.24
6.30
7.5
7.0
6.5
6.0
5.5
5.0
4.5
4.0
3.5
3.0
2.5
2.0
1.5
1.0
ppm

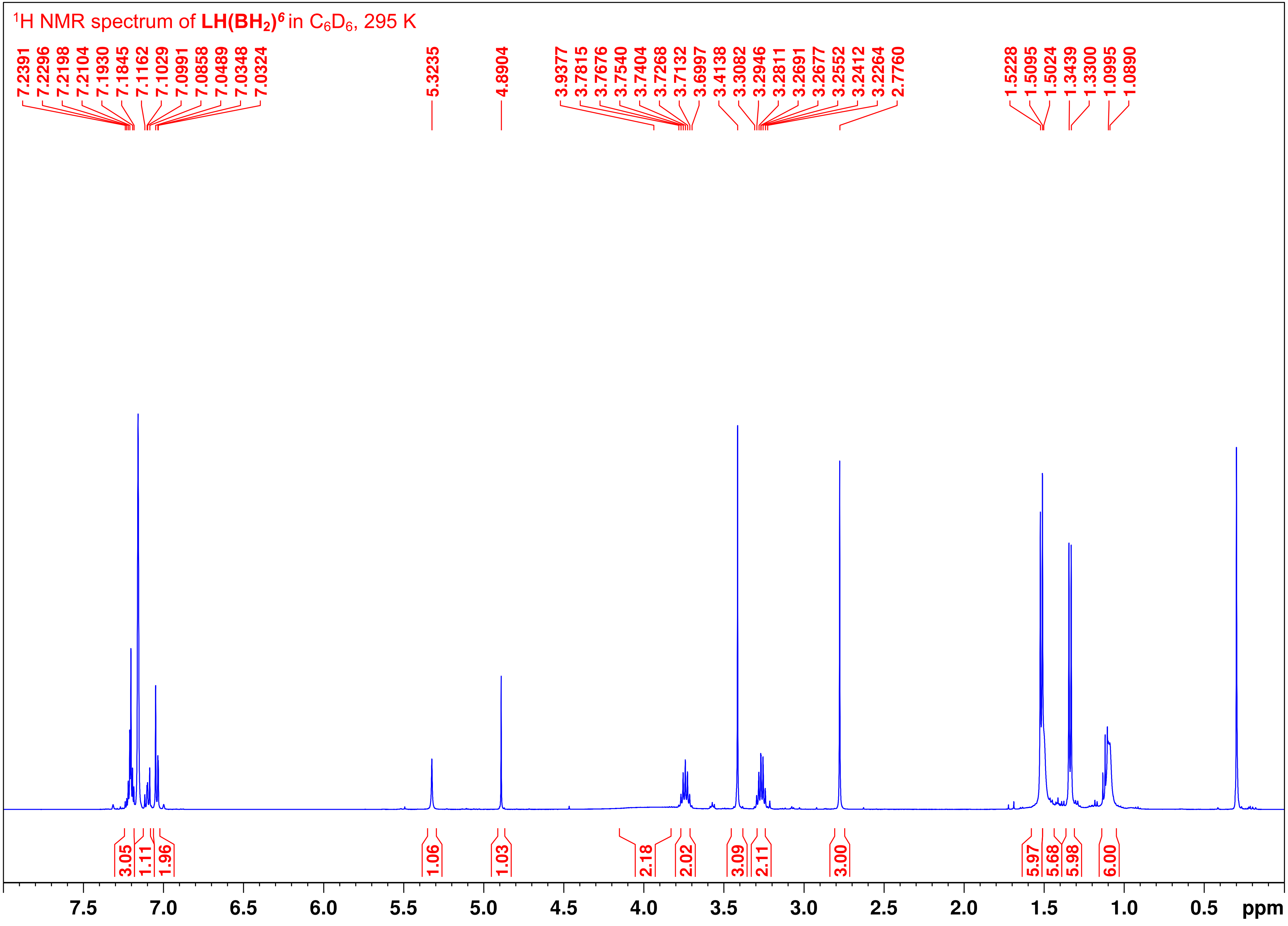

1H NMR spectrum of LH(BH2)6 in C6D6, 295 K
7.2391
7.2296
7.2198
7.2104
7.1930
7.1845
7.1162
7.1029
7.0991
7.0858
7.0489
7.0348
7.0324
5.3235
4.8904
3.9377
3.7815
3.7676
3.7540
3.7404
3.7268
3.7132
3.6997
3.4138
3.3082
3.2946
3.2811
3.2691
3.2677
3.2552
3.2412
3.2264
2.7760
1.5228
1.5095
1.5024
1.3439
1.3300
1.0995
1.0890
3.05
1.11
1.96
1.06
1.03
2.18
2.02
3.09
2.11
3.00
5.97
5.68
5.98
6.00
7.5
7.0
6.5
6.0
5.5
5.0
4.5
4.0
3.5
3.0
2.5
2.0
1.5
1.0
0.5
ppm

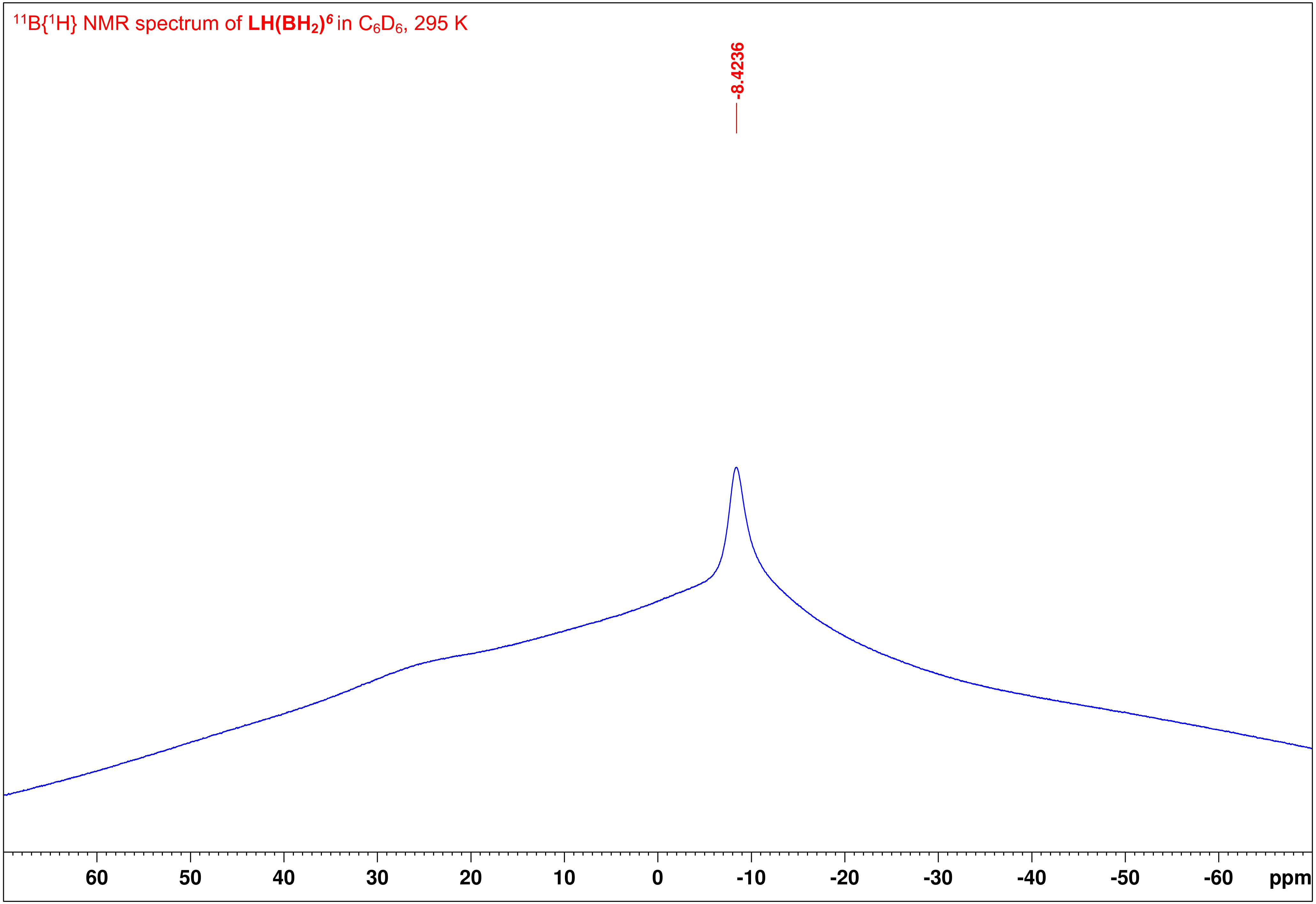

$^{11}B\{^1H\}$ NMR spectrum of **LH(BH$_2$)^6** in C_6D_6, 295 K
-8.4236
60
50
40
30
20
10
0
-10
-20
-30
-40
-50
-60
ppm

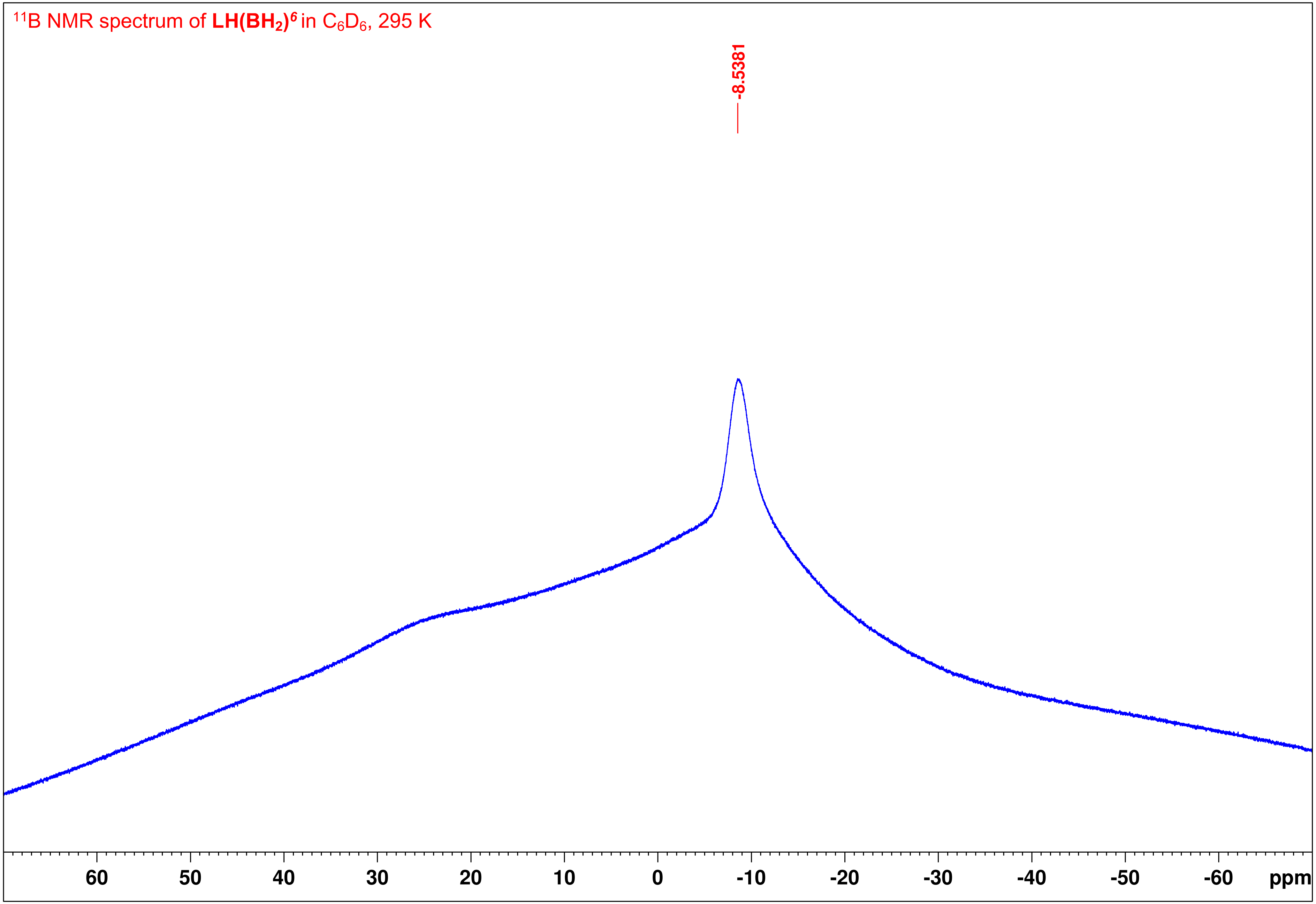

^{11}B NMR spectrum of **LH(BH$_2$)^6** in C_6D_6, 295 K
-8.5381
60
50
40
30
20
10
0
-10
-20
-30
-40
-50
-60
ppm

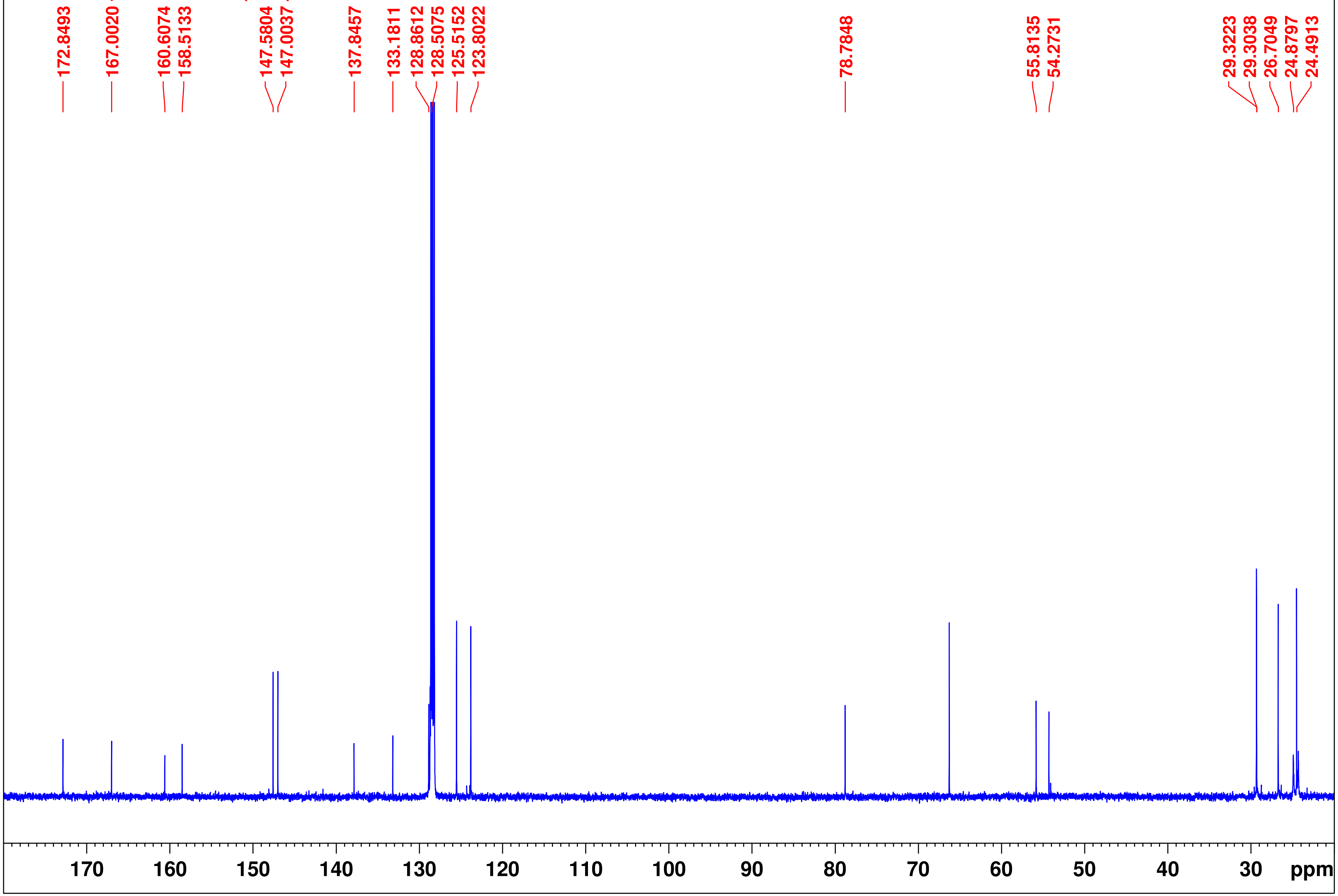

^{13}C NMR spectrum of **LH(BH$_2$)^6** in C_6D_6, 295 K

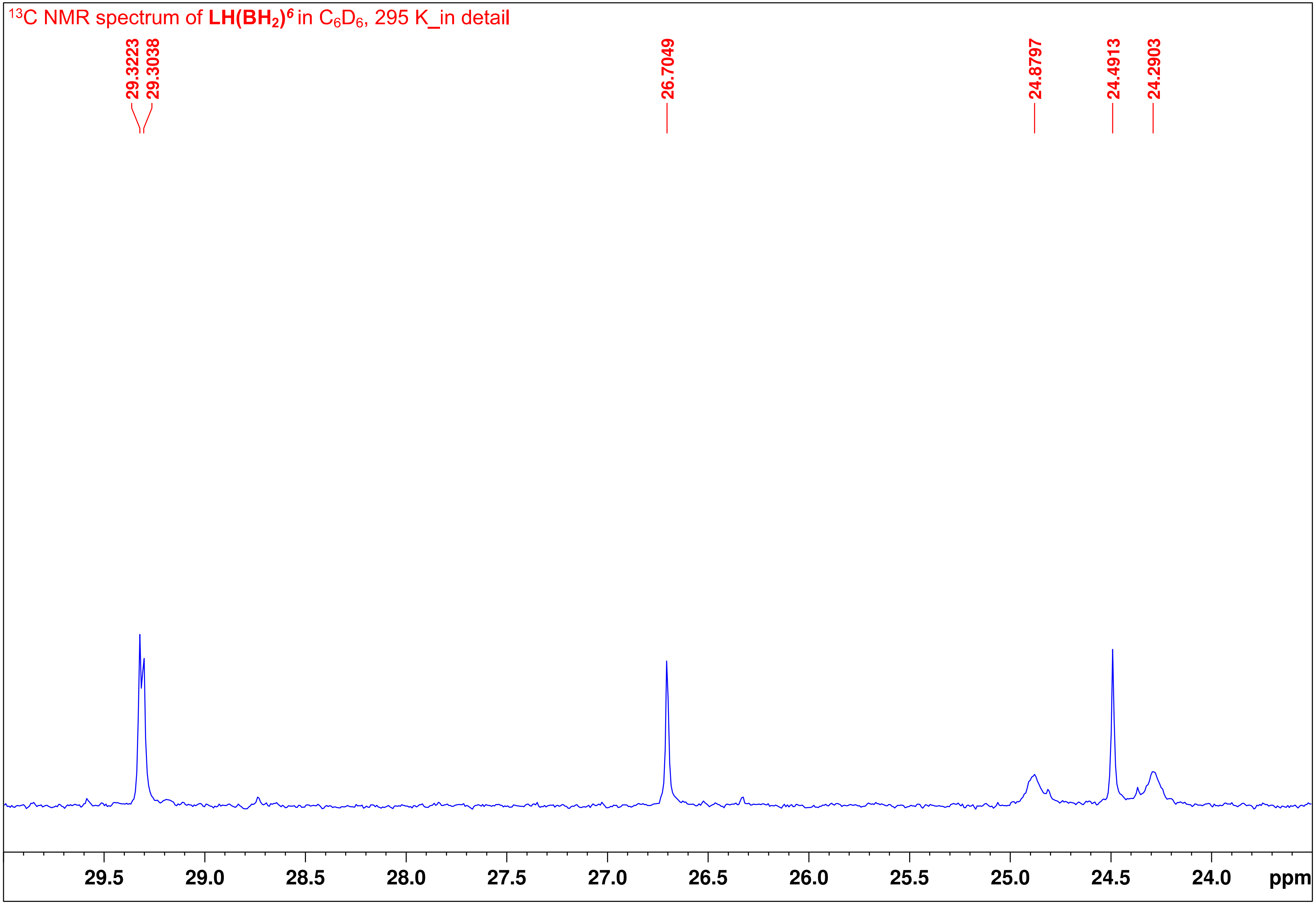

^{13}C NMR spectrum of **LH(BH$_2$)^6** in C_6D_6, 295 K_in detail
29.3223
29.3038
26.7049
24.8797
24.4913
24.2903
29.5
29.0
28.5
28.0
27.5
27.0
26.5
26.0
25.5
25.0
24.5
24.0
ppm

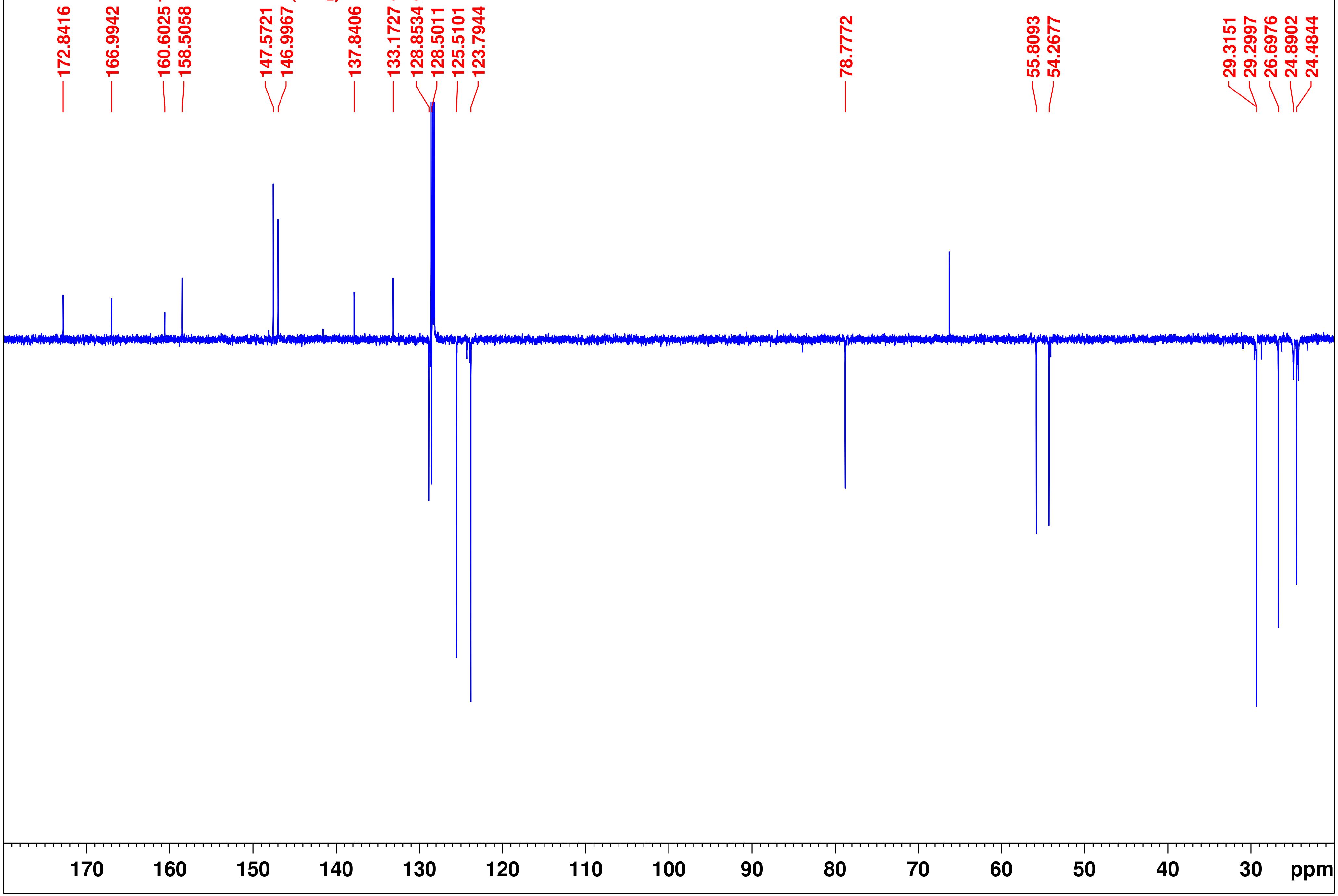

^{13}C APT NMR spectrum of **$LH(BH_2)^6$** in C_6D_6, 295 K

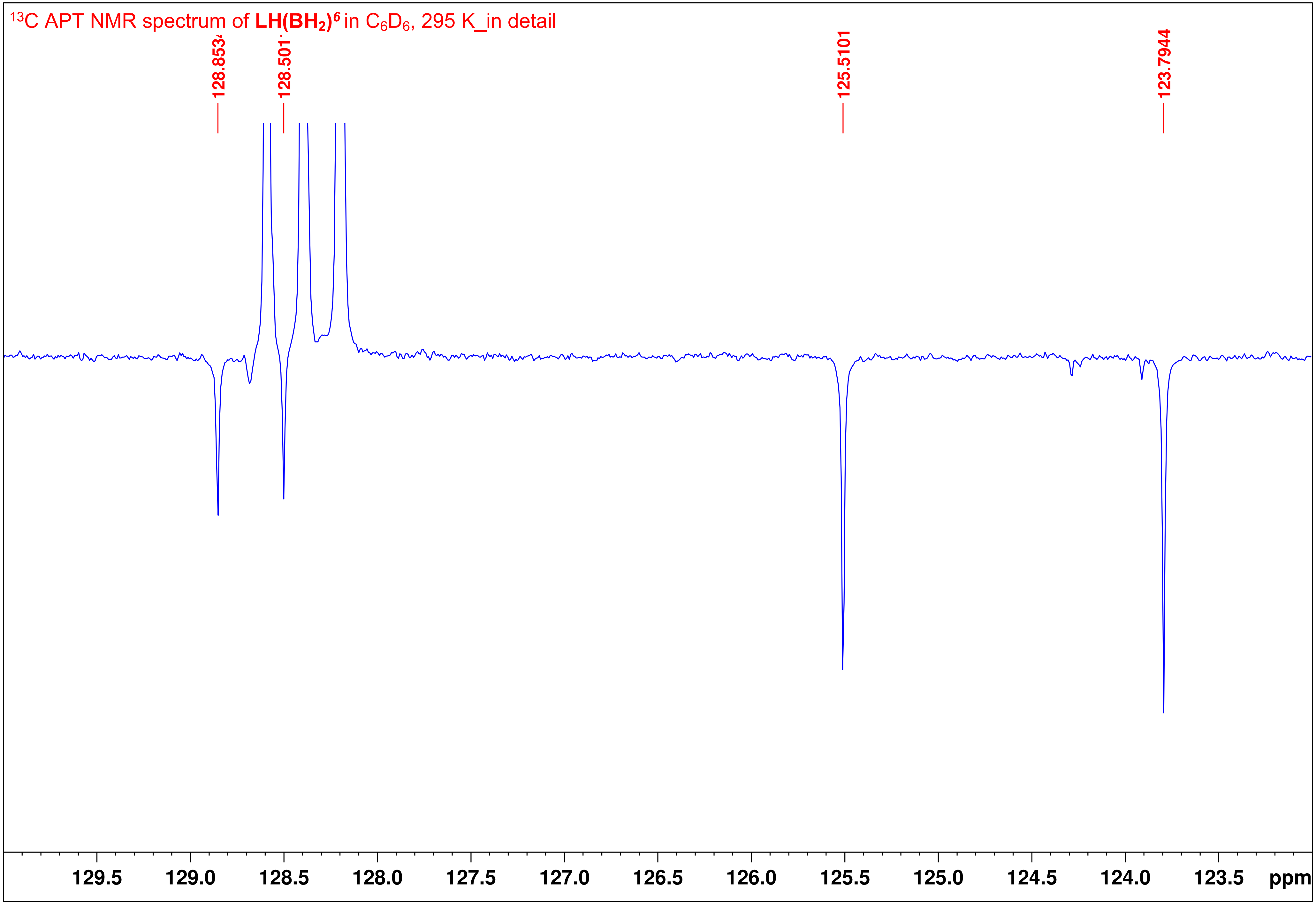

^{13}C APT NMR spectrum of **LH(BH$_2$)^6** in C_6D_6, 295 K_in detail

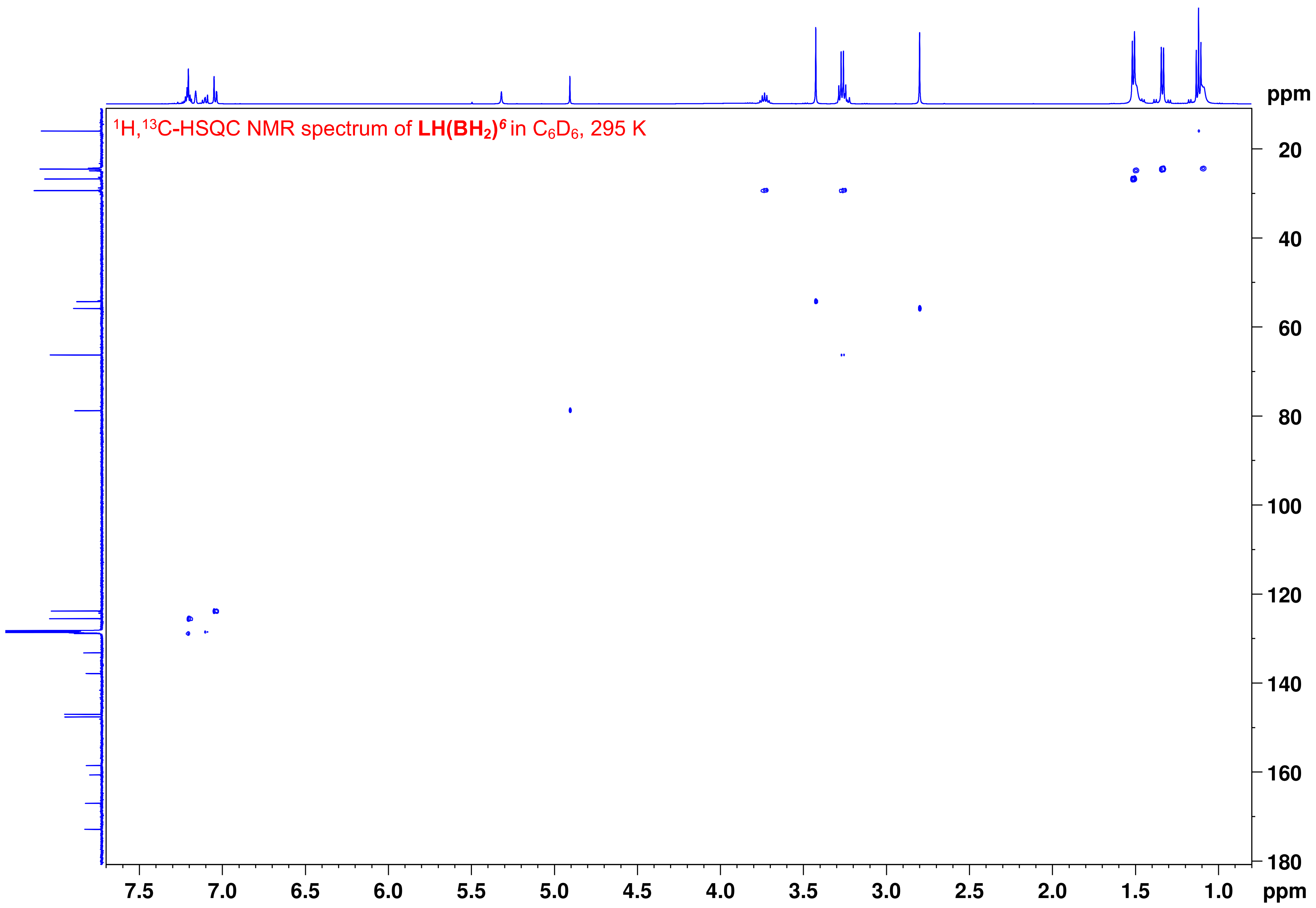
$^1H,^{13}C$-HSQC NMR spectrum of **LH(BH$_2$)^6** in C_6D_6, 295 K
ppm
20
40
60
80
100
120
140
160
180
ppm
7.5
7.0
6.5
6.0
5.5
5.0
4.5
4.0
3.5
3.0
2.5
2.0
1.5
1.0
ppm

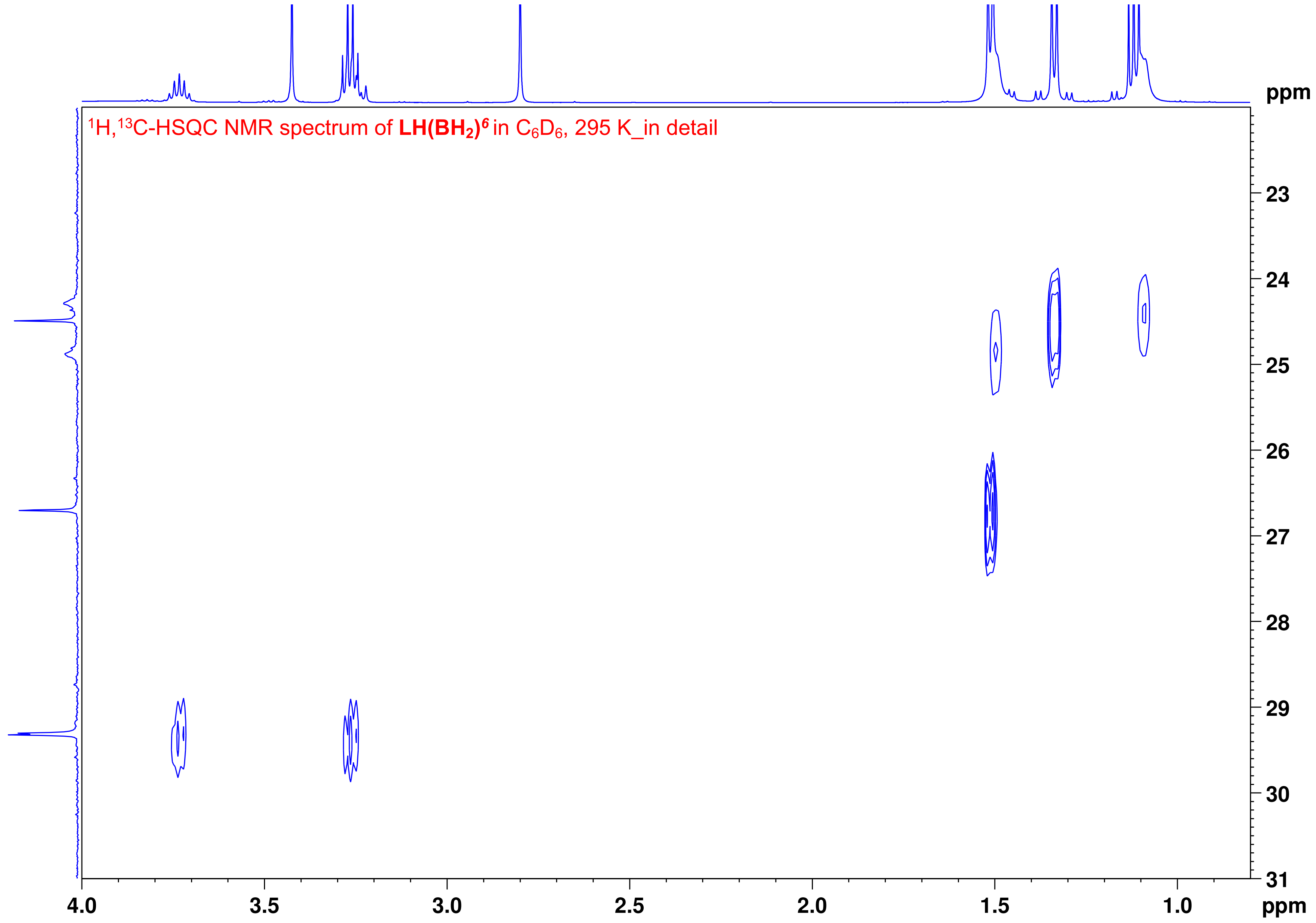

$^{1}H,^{13}C$-HSQC NMR spectrum of **LH(BH$_2$)^6** in C_6D_6, 295 K_in detail
ppm
23
24
25
26
27
28
29
30
31
4.0
3.5
3.0
2.5
2.0
1.5
1.0
ppm

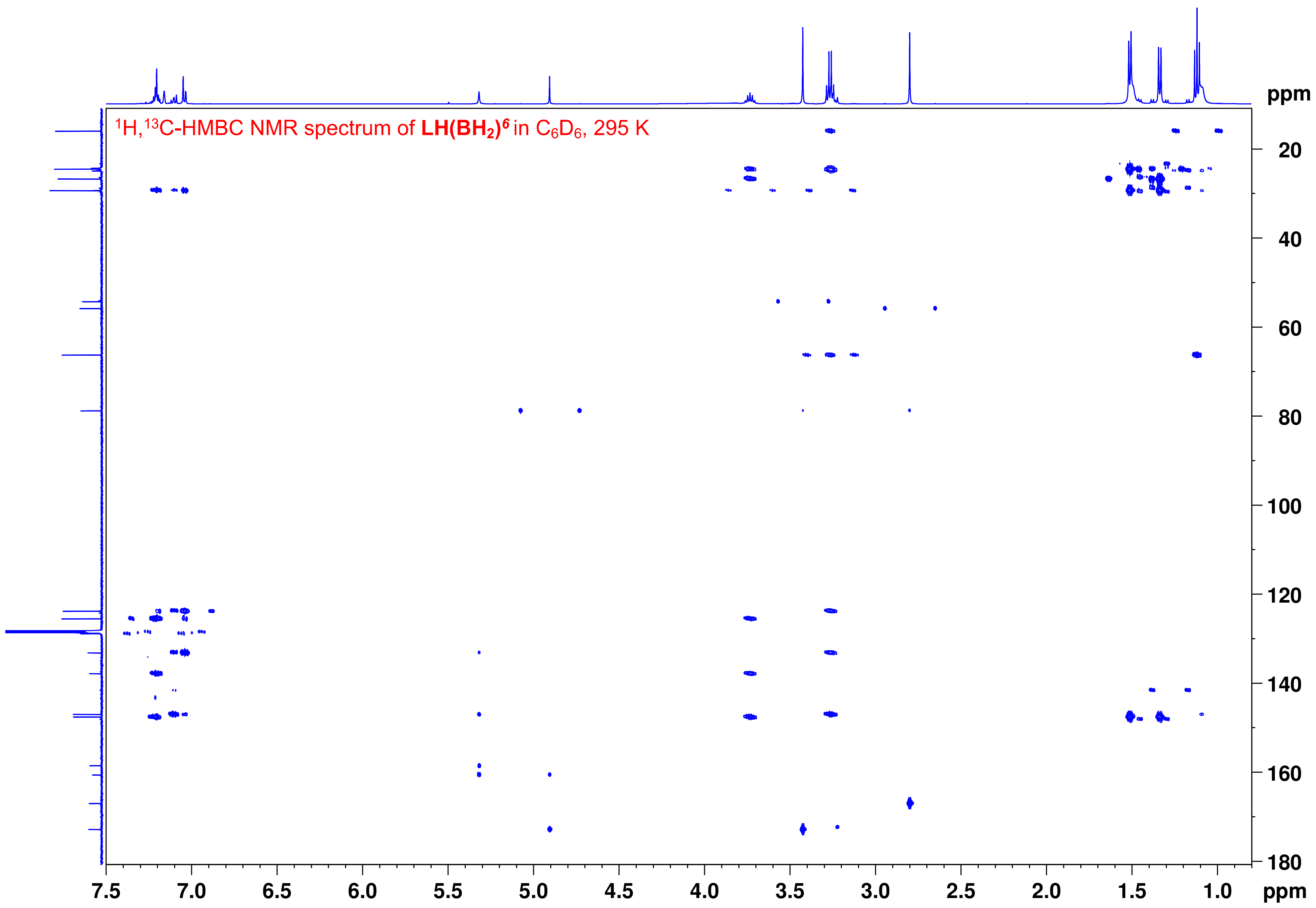
$^1H,^{13}C$-HMBC NMR spectrum of **$LH(BH_2)^6$** in C_6D_6, 295 K
ppm
7.5 7.0 6.5 6.0 5.5 5.0 4.5 4.0 3.5 3.0 2.5 2.0 1.5 1.0 ppm
20 40 60 80 100 120 140 160 180

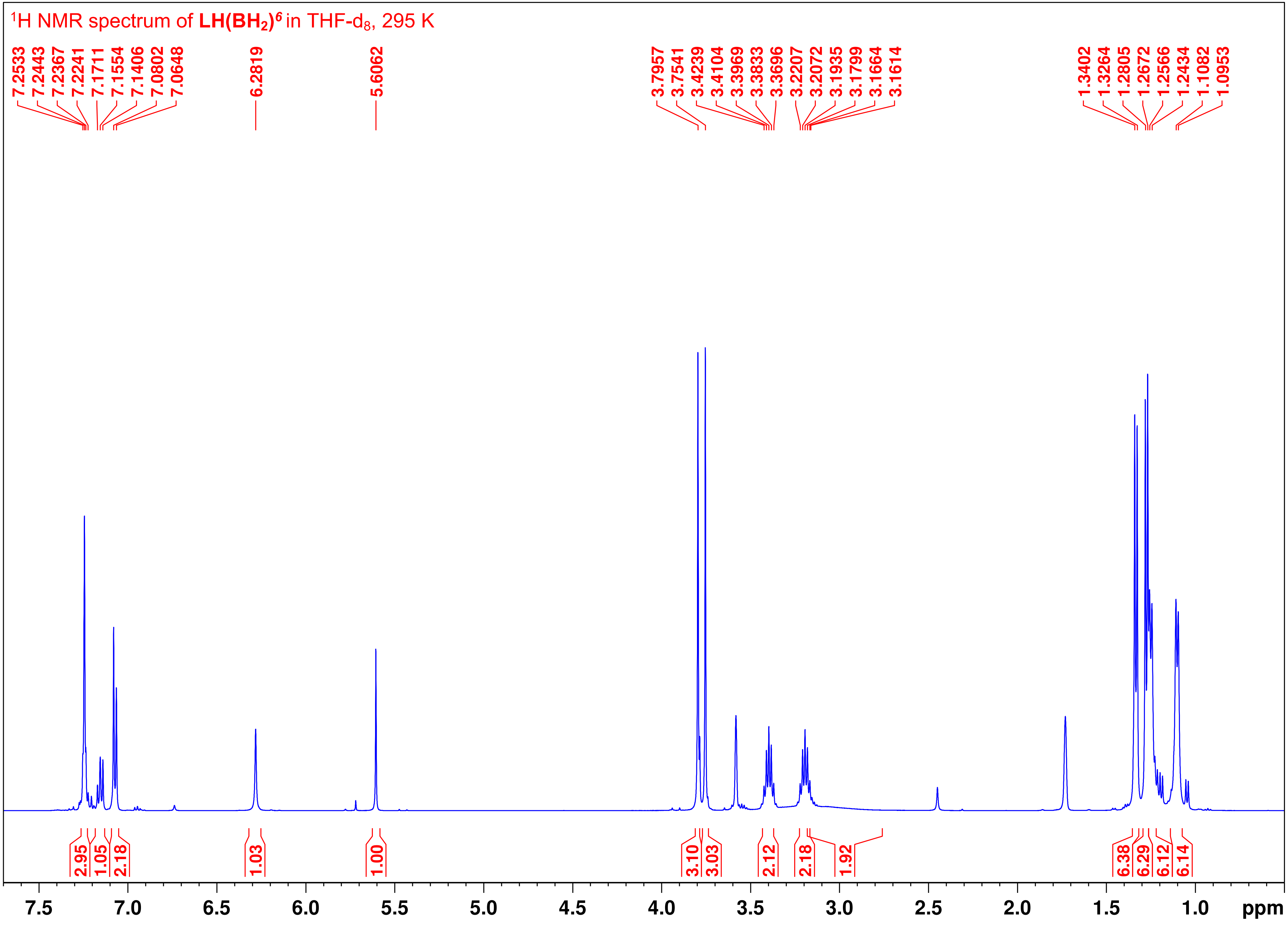

^{1}H NMR spectrum of **LH(BH$_2$)^6** in THF-d$_8$, 295 K
7.2533
7.2443
7.2367
7.2241
7.1711
7.1554
7.1406
7.0802
7.0648
6.2819
5.6062
3.7957
3.7541
3.4239
3.4104
3.3969
3.3833
3.3696
3.2207
3.2072
3.1935
3.1799
3.1664
3.1614
1.3402
1.3264
1.2805
1.2672
1.2566
1.2434
1.1082
1.0953
2.95
1.05
2.18
1.03
1.00
3.10
3.03
2.12
2.18
1.92
6.38
6.29
6.12
6.14
7.5
7.0
6.5
6.0
5.5
5.0
4.5
4.0
3.5
3.0
2.5
2.0
1.5
1.0
ppm

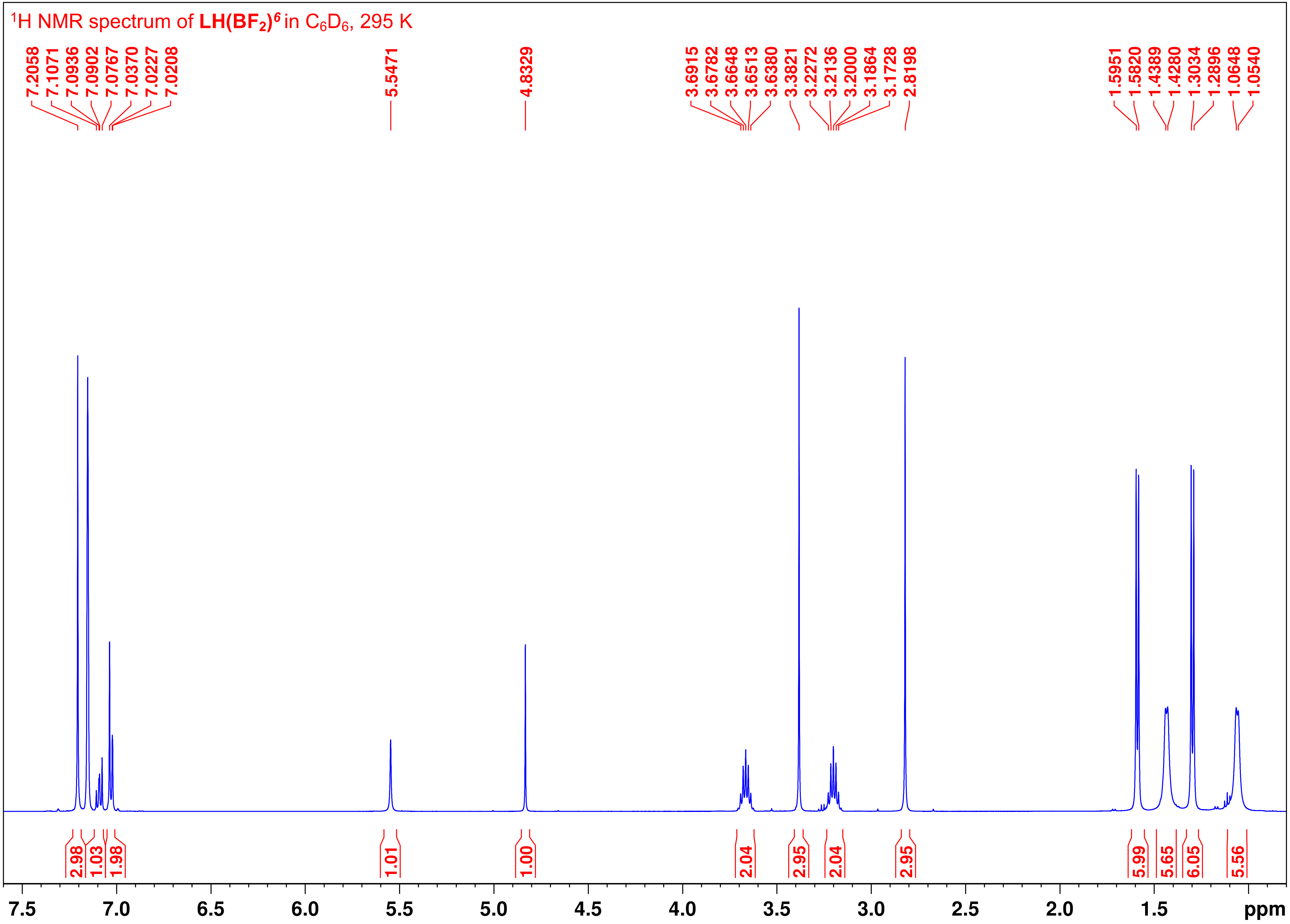

^{1}H NMR spectrum of **LH(BF$_2$)^6** in C_6D_6, 295 K
7.2058
7.1071
7.0936
7.0902
7.0767
7.0370
7.0227
7.0208
5.5471
4.8329
3.6915
3.6782
3.6648
3.6513
3.6380
3.3821
3.2272
3.2136
3.2000
3.1864
3.1728
2.8198
1.5951
1.5820
1.4389
1.4280
1.3034
1.2896
1.0648
1.0540
2.98
1.03
1.98
1.01
1.00
2.04
2.95
2.04
2.95
5.99
5.65
6.05
5.56
7.5
7.0
6.5
6.0
5.5
5.0
4.5
4.0
3.5
3.0
2.5
2.0
1.5
ppm

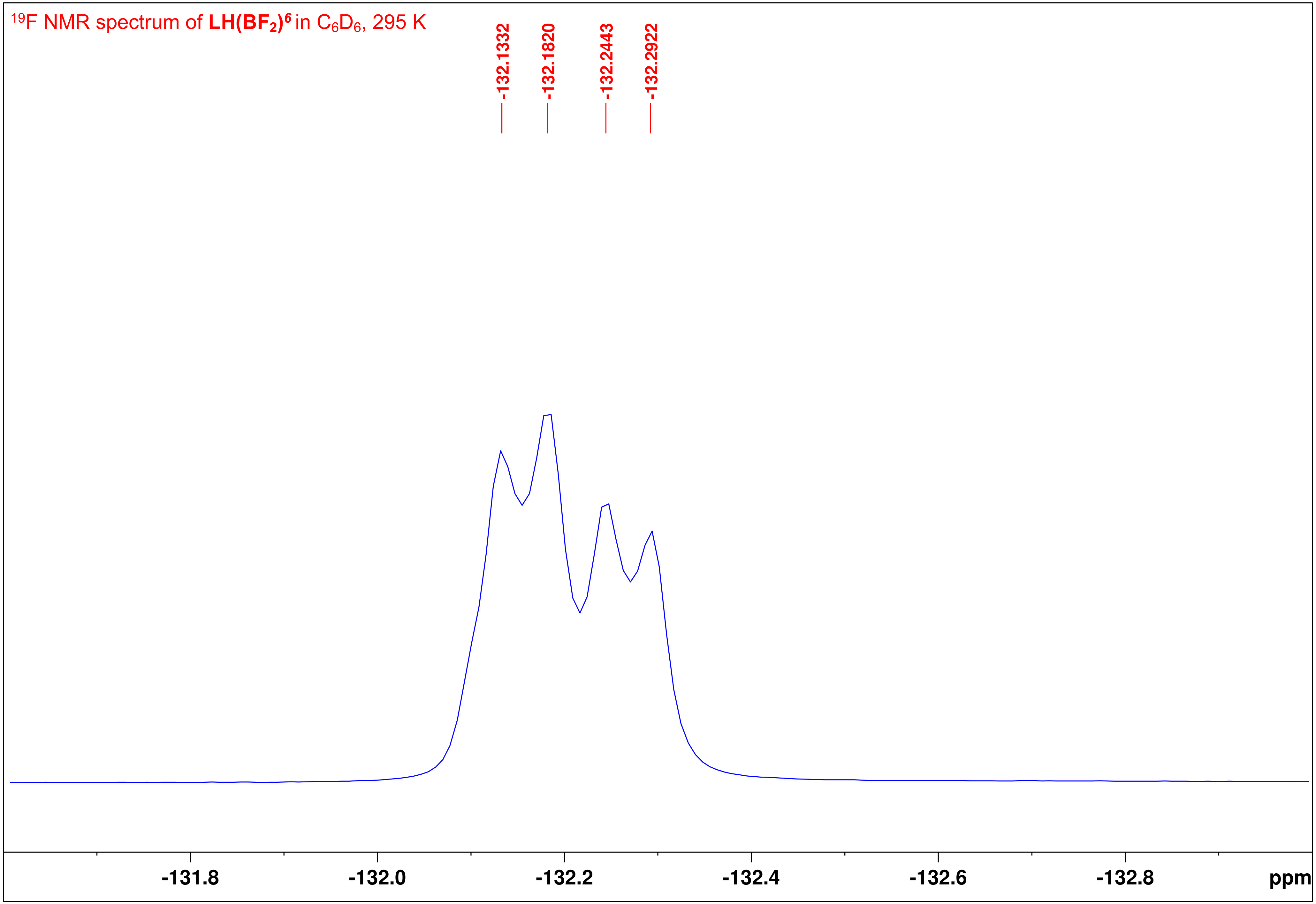

19F NMR spectrum of LH(BF2)6 in C6D6, 295 K
-132.1332
-132.1820
-132.2443
-132.2922
-131.8
-132.0
-132.2
-132.4
-132.6
-132.8
ppm

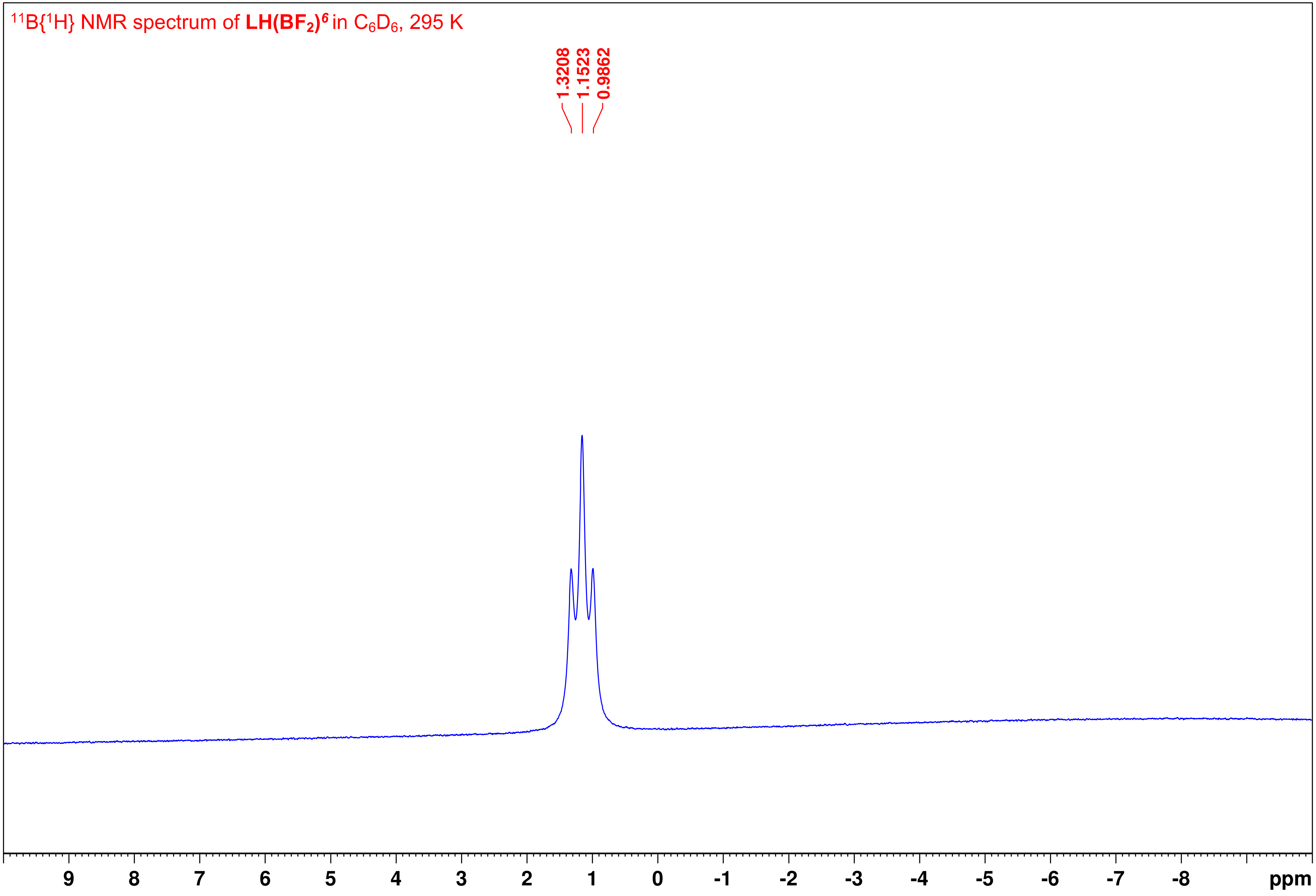

$^{11}B\{^1H\}$ NMR spectrum of **LH(BF$_2$)^6** in C_6D_6, 295 K
1.3208
1.1523
0.9862
9
8
7
6
5
4
3
2
1
0
-1
-2
-3
-4
-5
-6
-7
-8
ppm

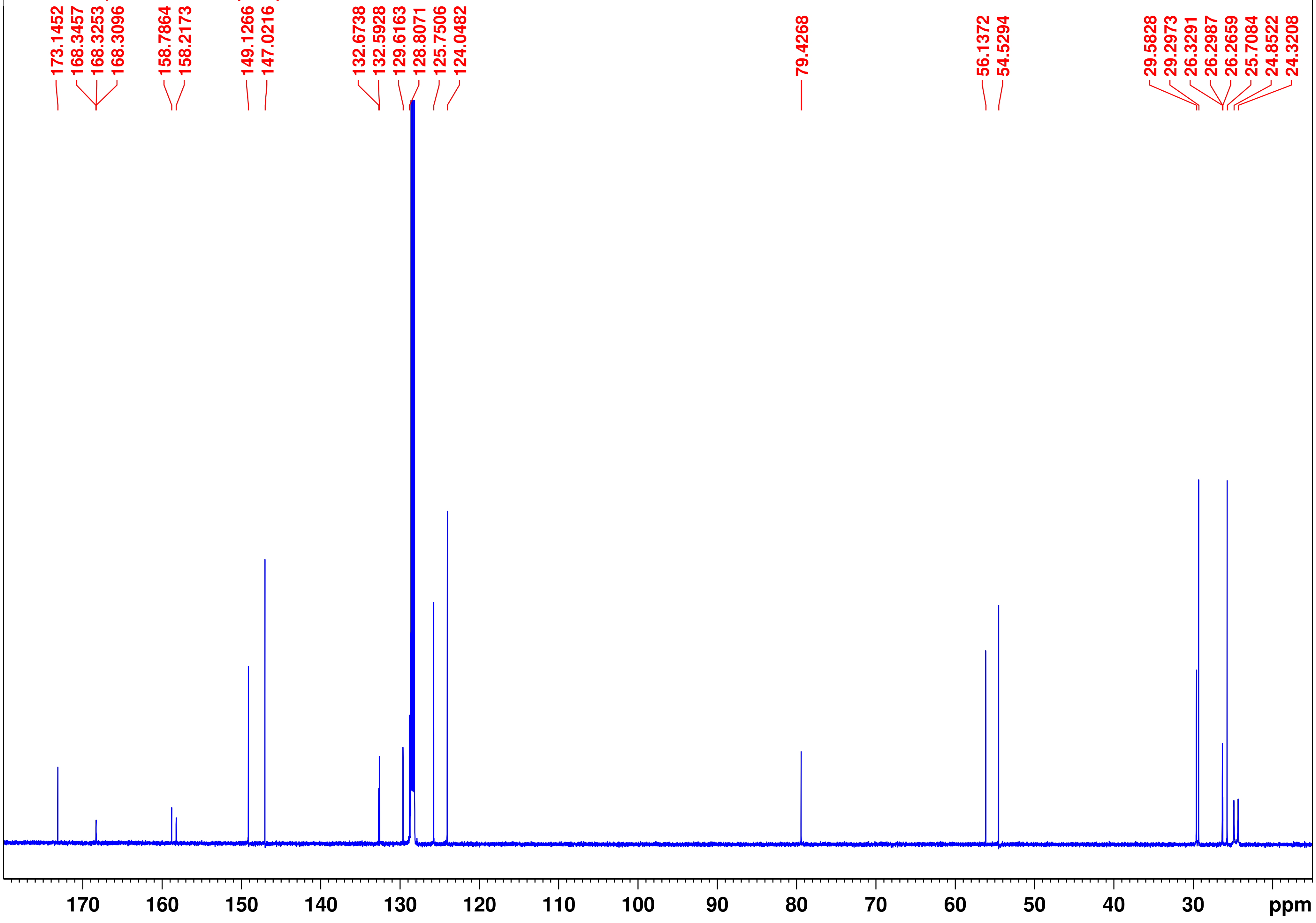

^{13}C NMR spectrum of **LH(BF$_2$)^6** in C_6D_6, 295 K

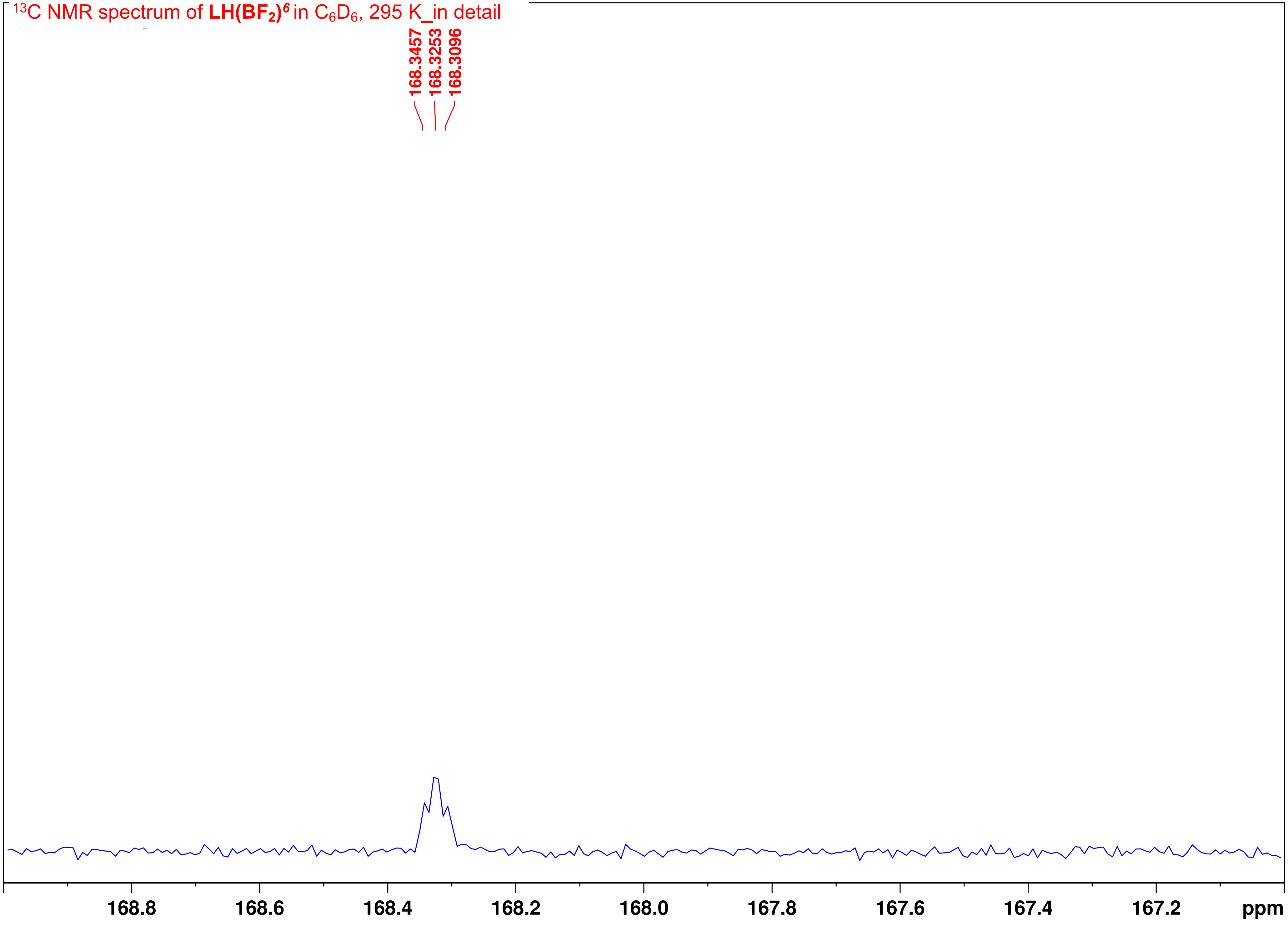
^{13}C NMR spectrum of **LH(BF$_2$)^6** in C_6D_6, 295 K_in detail
168.3457
168.3253
168.3096
168.8
168.6
168.4
168.2
168.0
167.8
167.6
167.4
167.2
ppm

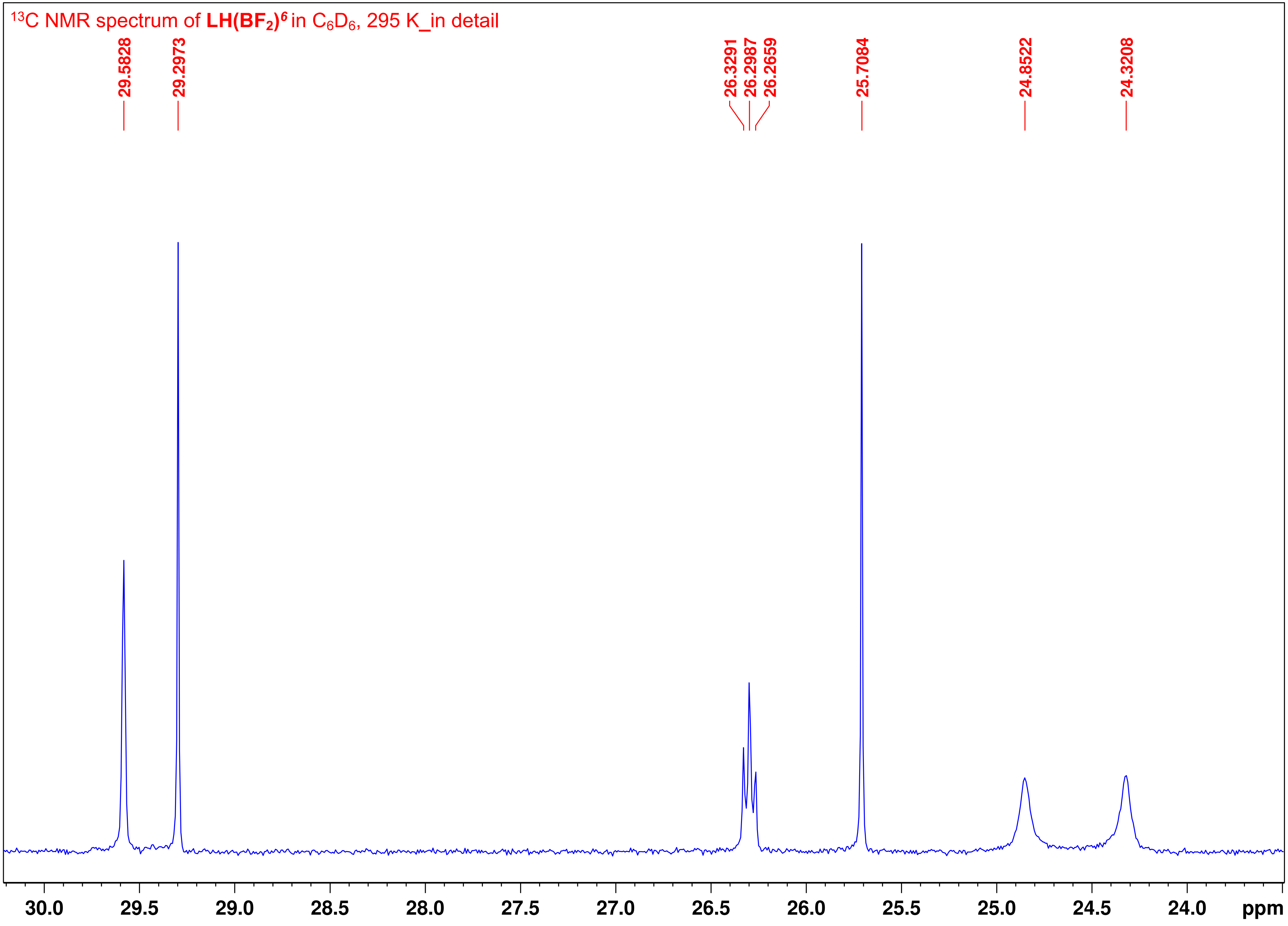
^{13}C NMR spectrum of **LH(BF$_2$)^6** in C_6D_6, 295 K_in detail
29.5828
29.2973
26.3291
26.2987
26.2659
25.7084
24.8522
24.3208
30.0
29.5
29.0
28.5
28.0
27.5
27.0
26.5
26.0
25.5
25.0
24.5
24.0
ppm

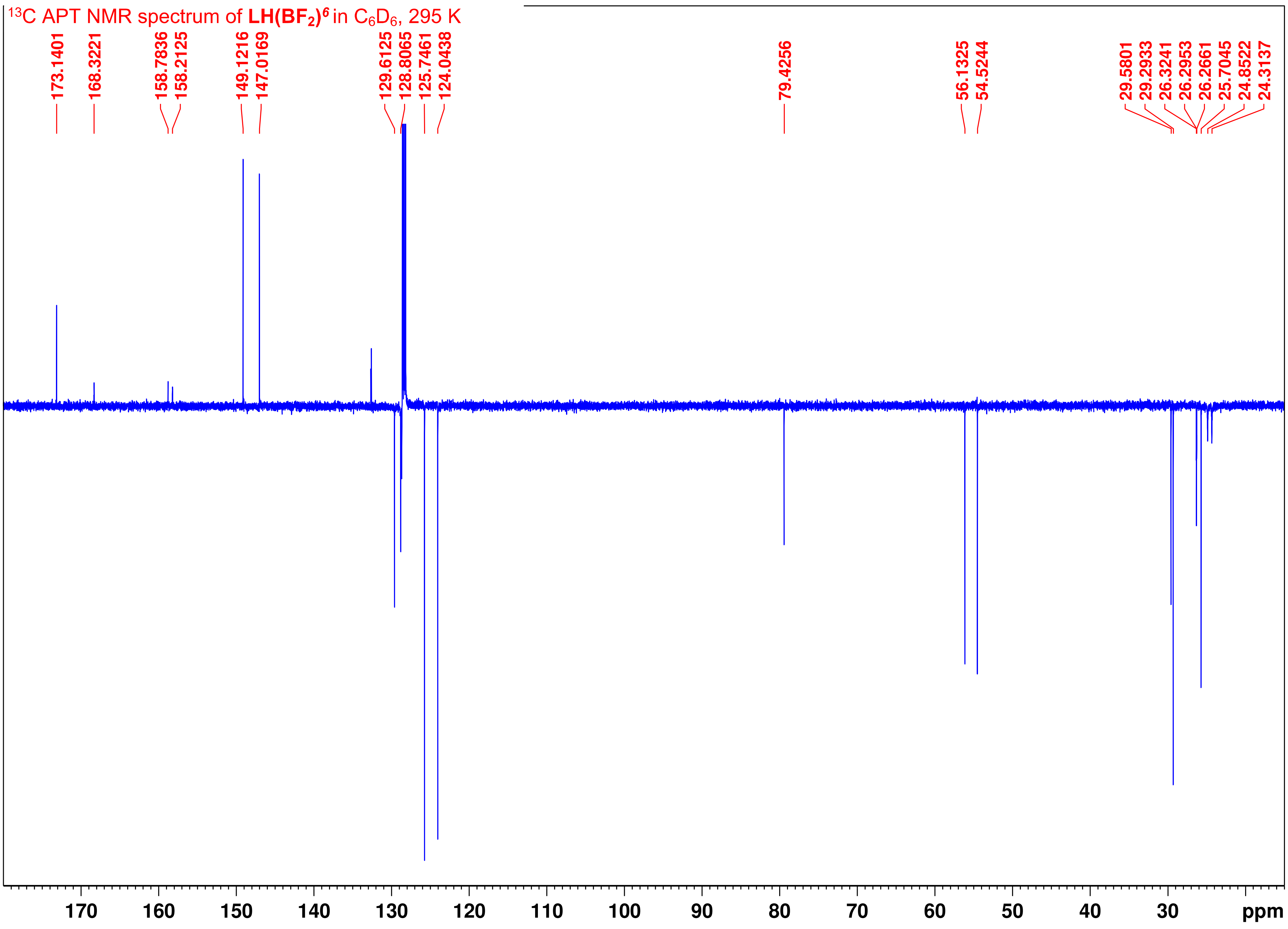

^{13}C APT NMR spectrum of **LH(BF$_2$)^6** in C_6D_6, 295 K
173.1401
168.3221
158.7836
158.2125
149.1216
147.0169
129.6125
128.8065
125.7461
124.0438
79.4256
56.1325
54.5244
29.5801
29.2933
26.3241
26.2953
26.2661
25.7045
24.8522
24.3137
170
160
150
140
130
120
110
100
90
80
70
60
50
40
30
ppm

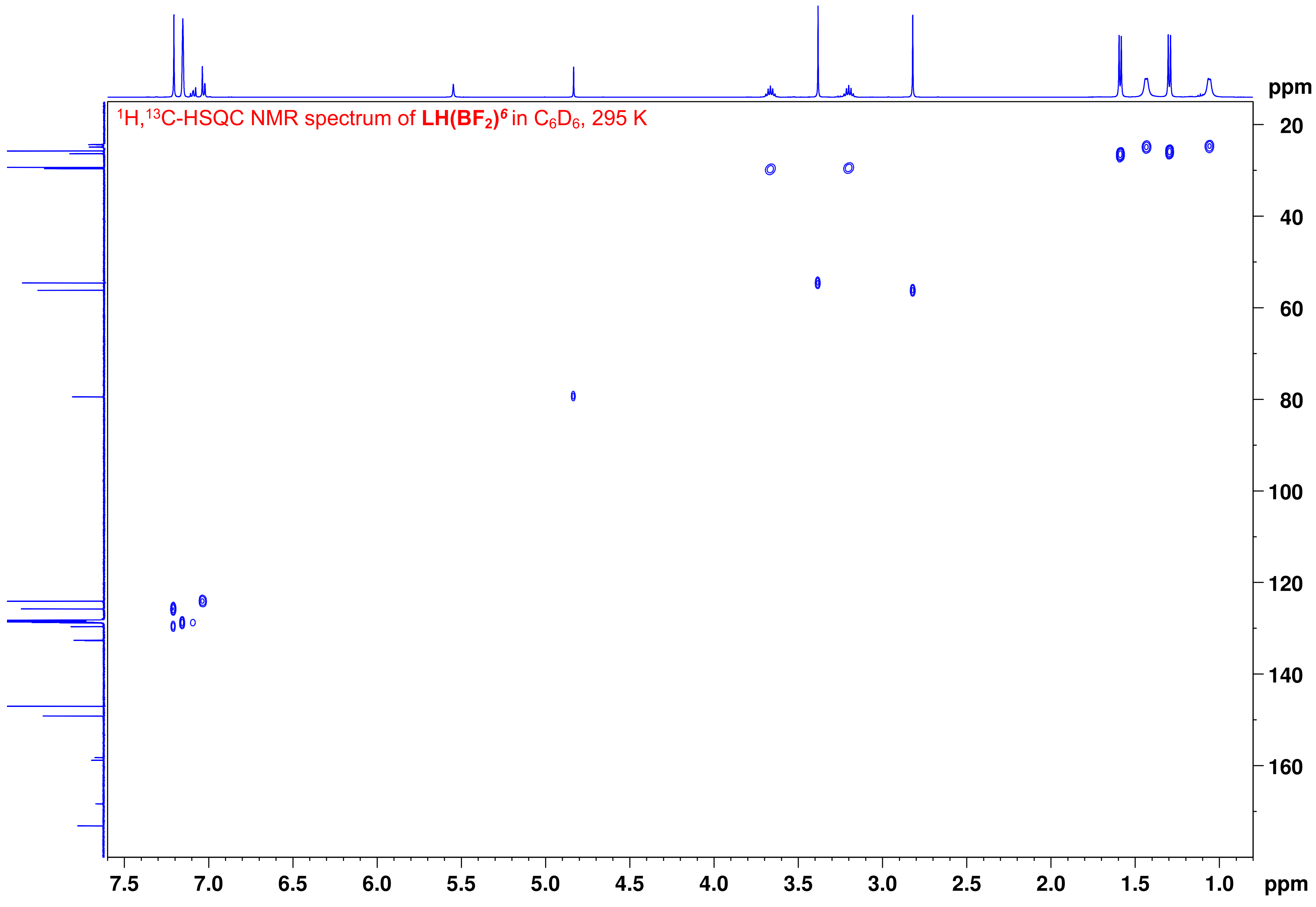

$^{1}H,^{13}C$-HSQC NMR spectrum of **LH(BF$_2$)^6** in C_6D_6, 295 K
ppm
20
40
60
80
100
120
140
160
7.5
7.0
6.5
6.0
5.5
5.0
4.5
4.0
3.5
3.0
2.5
2.0
1.5
1.0
ppm

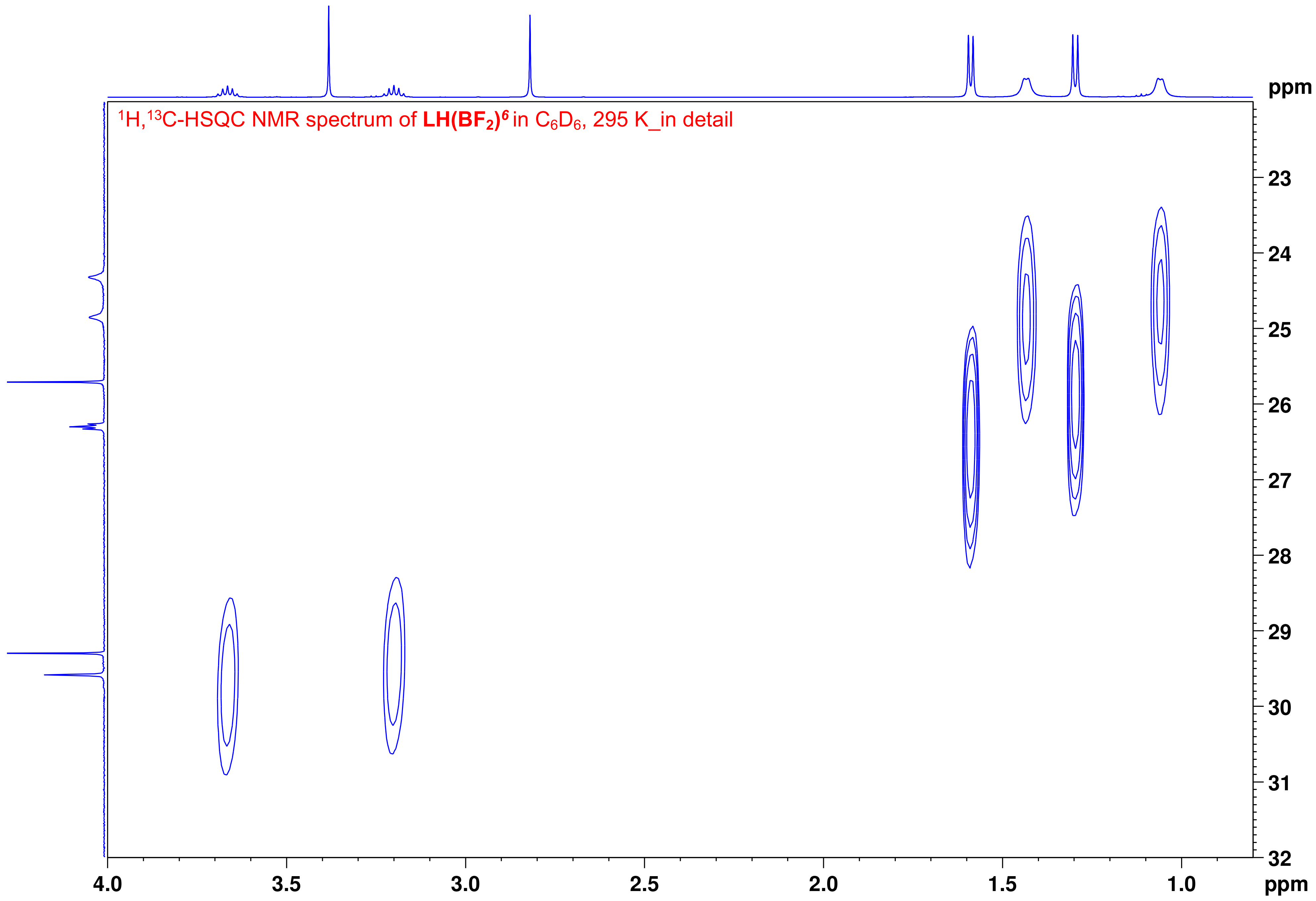

$^{1}H,^{13}C$-HSQC NMR spectrum of **LH(BF$_2$)^6** in C_6D_6, 295 K_in detail

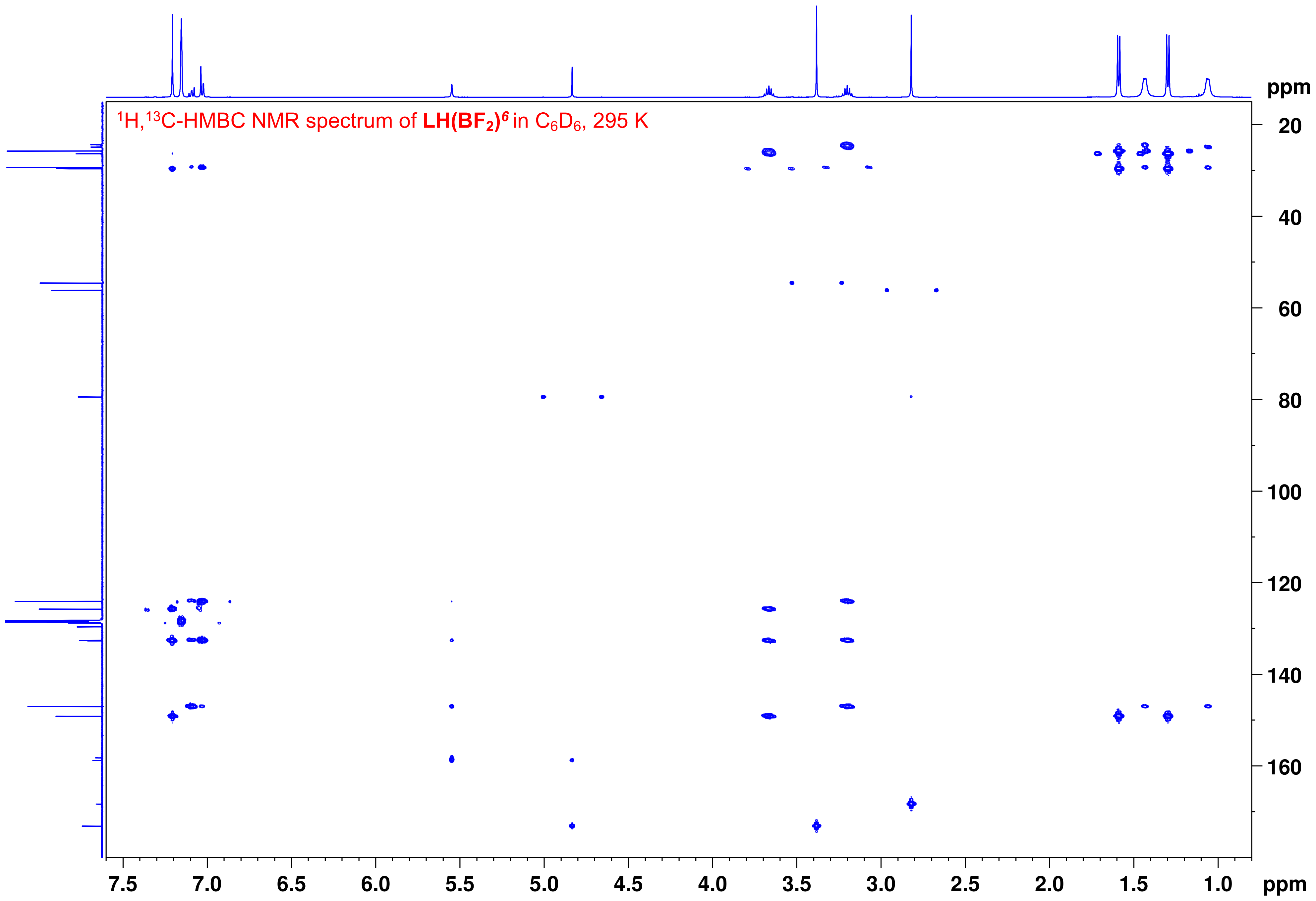

$^{1}H,^{13}C$-HMBC NMR spectrum of **LH(BF$_2$)^6** in C_6D_6, 295 K
ppm
20
40
60
80
100
120
140
160
ppm
7.5
7.0
6.5
6.0
5.5
5.0
4.5
4.0
3.5
3.0
2.5
2.0
1.5
1.0

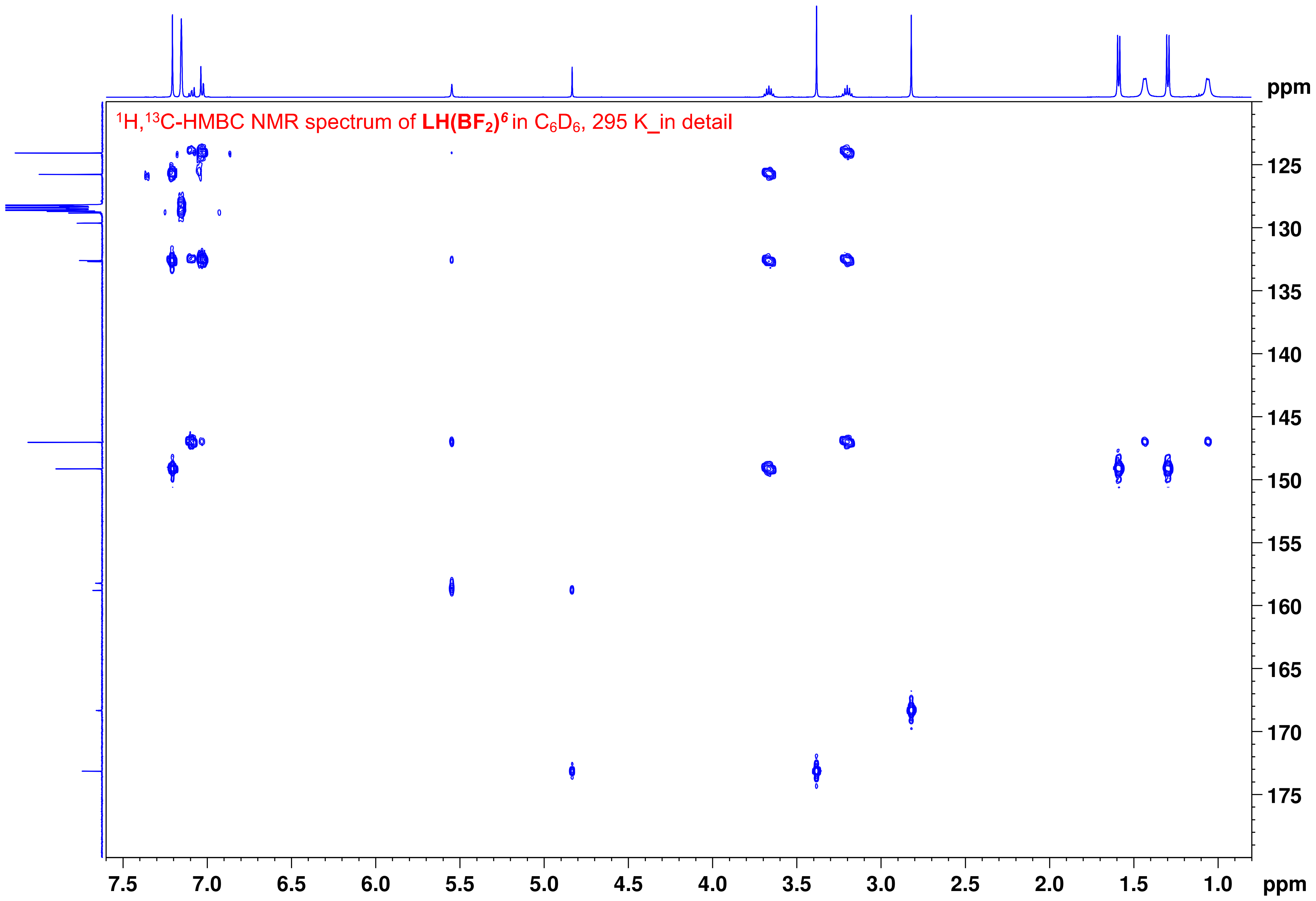

$^{1}H,^{13}C$-HMBC NMR spectrum of **LH(BF$_2$)^6** in C_6D_6, 295 K_in detail
ppm
125
130
135
140
145
150
155
160
165
170
175
7.5
7.0
6.5
6.0
5.5
5.0
4.5
4.0
3.5
3.0
2.5
2.0
1.5
1.0
ppm

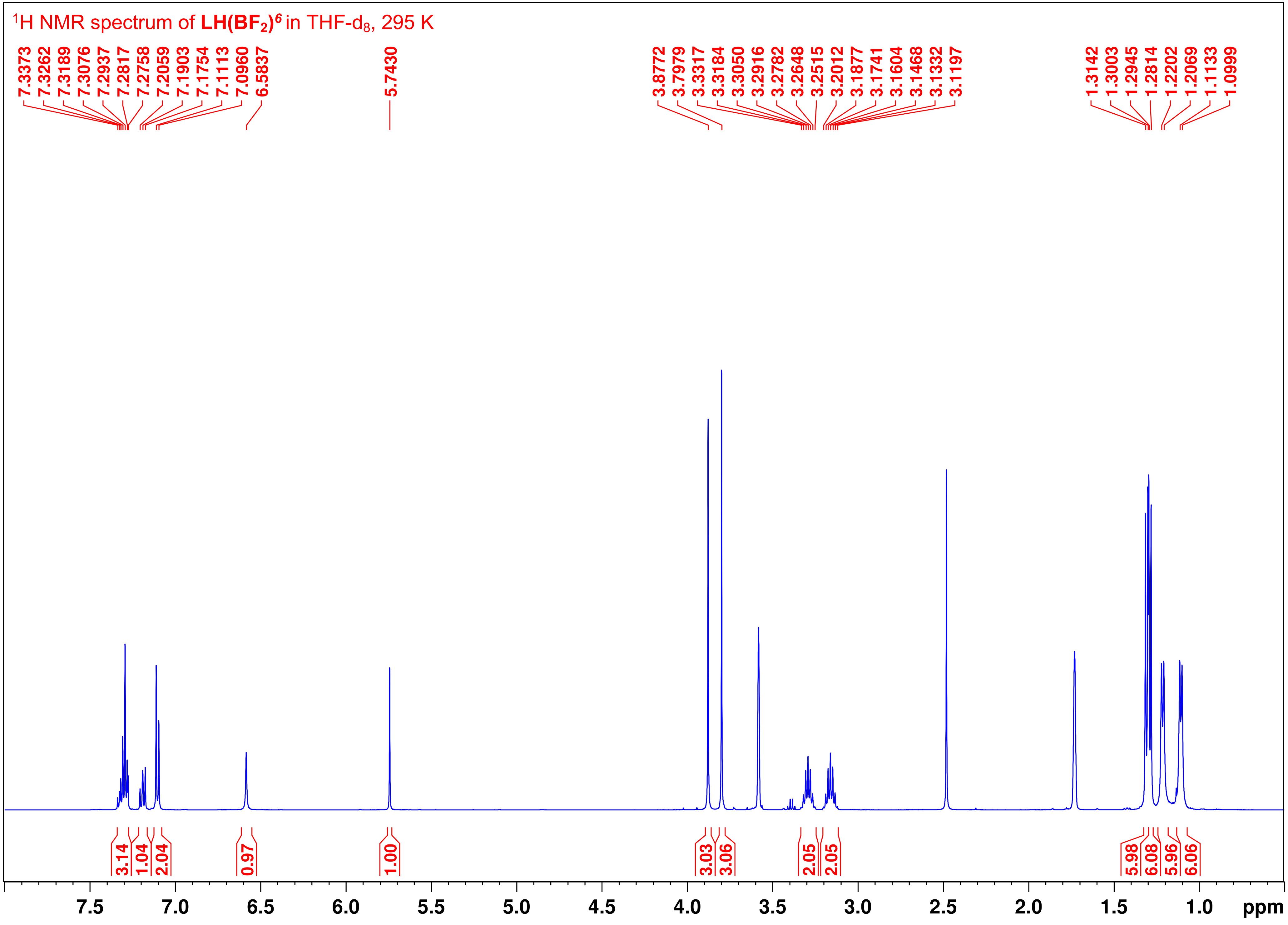

^{1}H NMR spectrum of **LH(BF$_2$)^6** in THF-d$_8$, 295 K

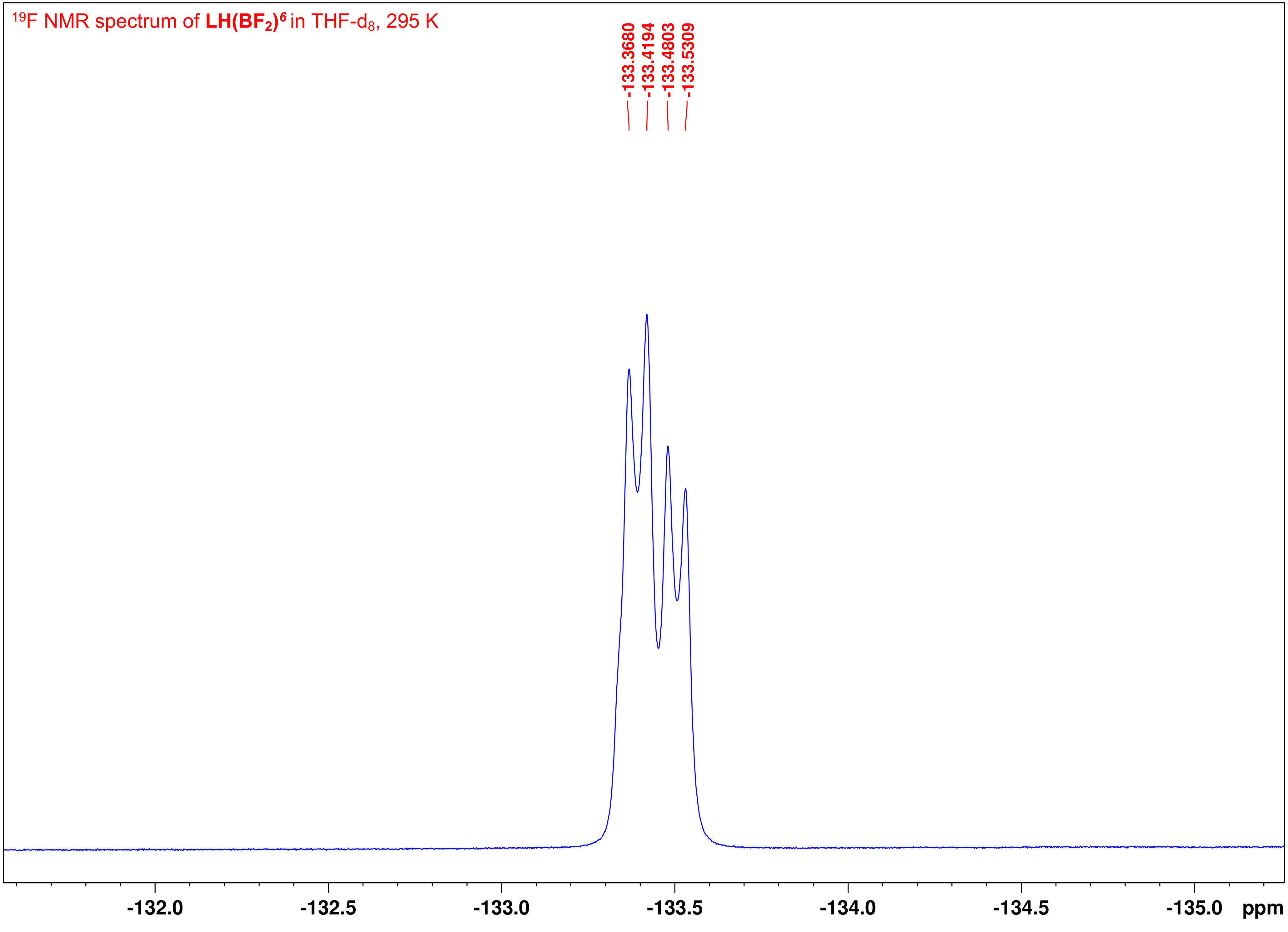

^{19}F NMR spectrum of **LH(BF$_2$)^6** in THF-d_8, 295 K
-133.3680
-133.4194
-133.4803
-133.5309
-132.0
-132.5
-133.0
-133.5
-134.0
-134.5
-135.0
ppm

$^{11}B\{^{1}H\}$ NMR spectrum of **LH(BF$_2$)^6** in THF-d$_8$, 295 K

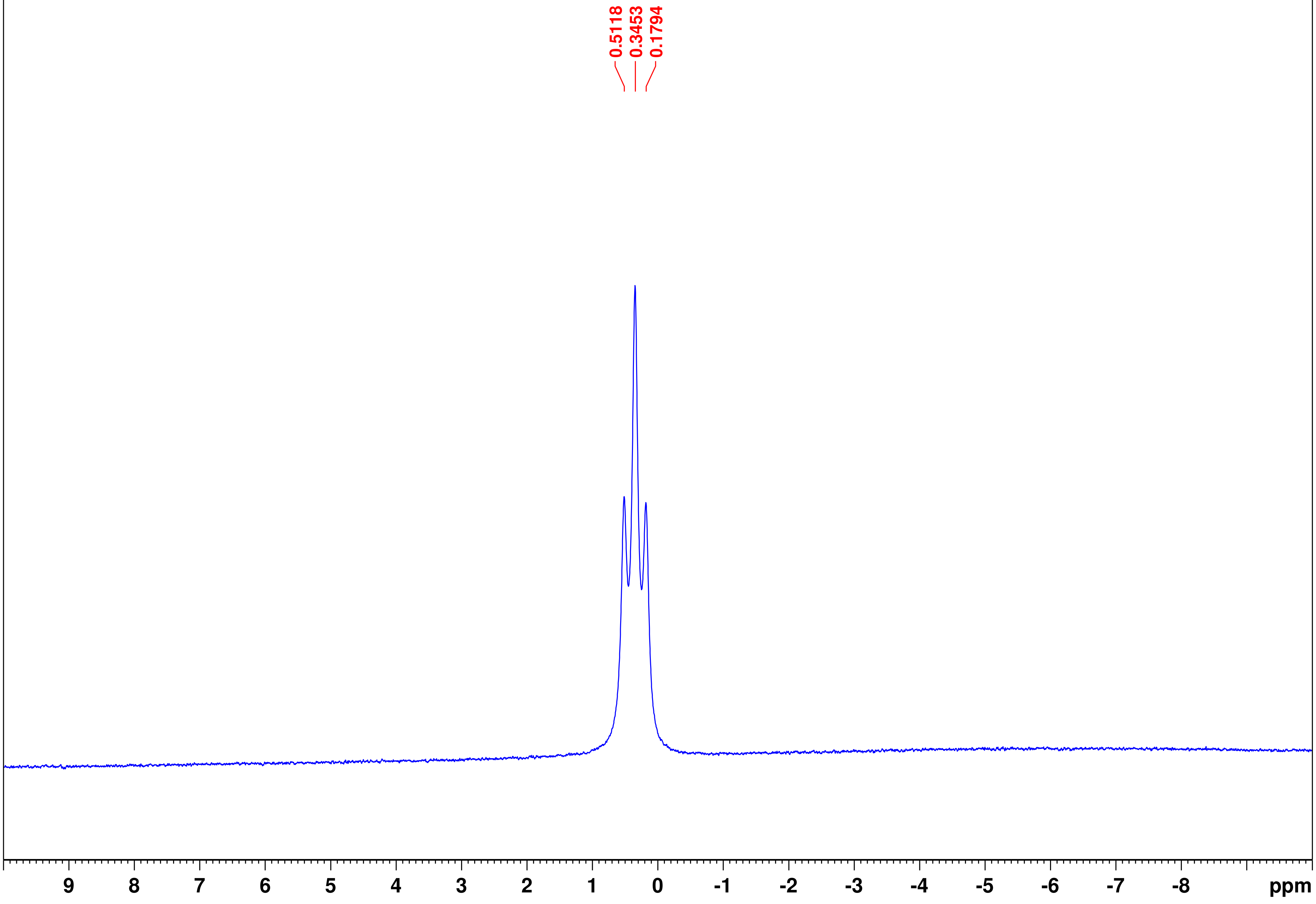

^{13}C NMR spectrum of **LH(BF$_2$)^6** in THF-d_8, 295 K

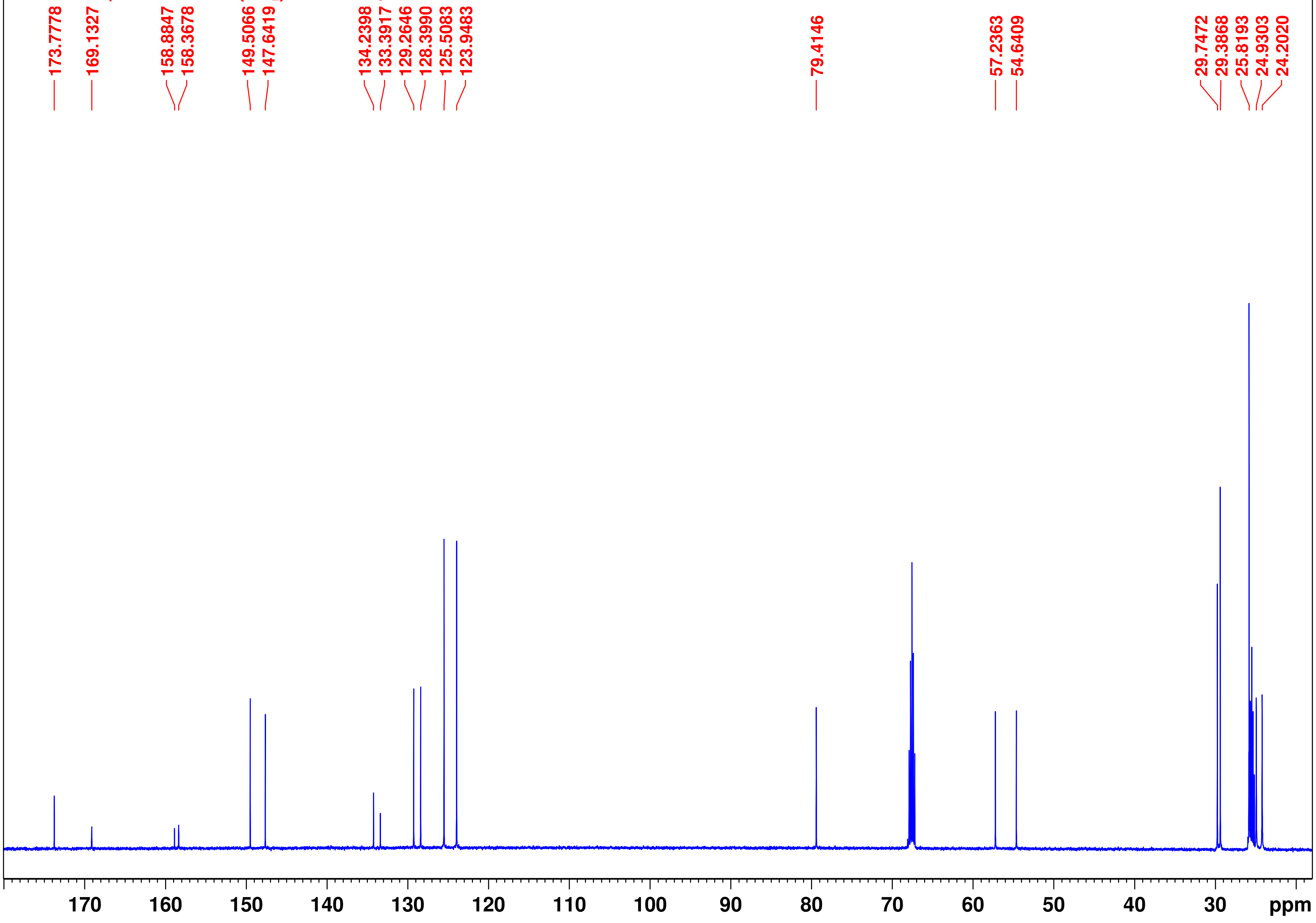

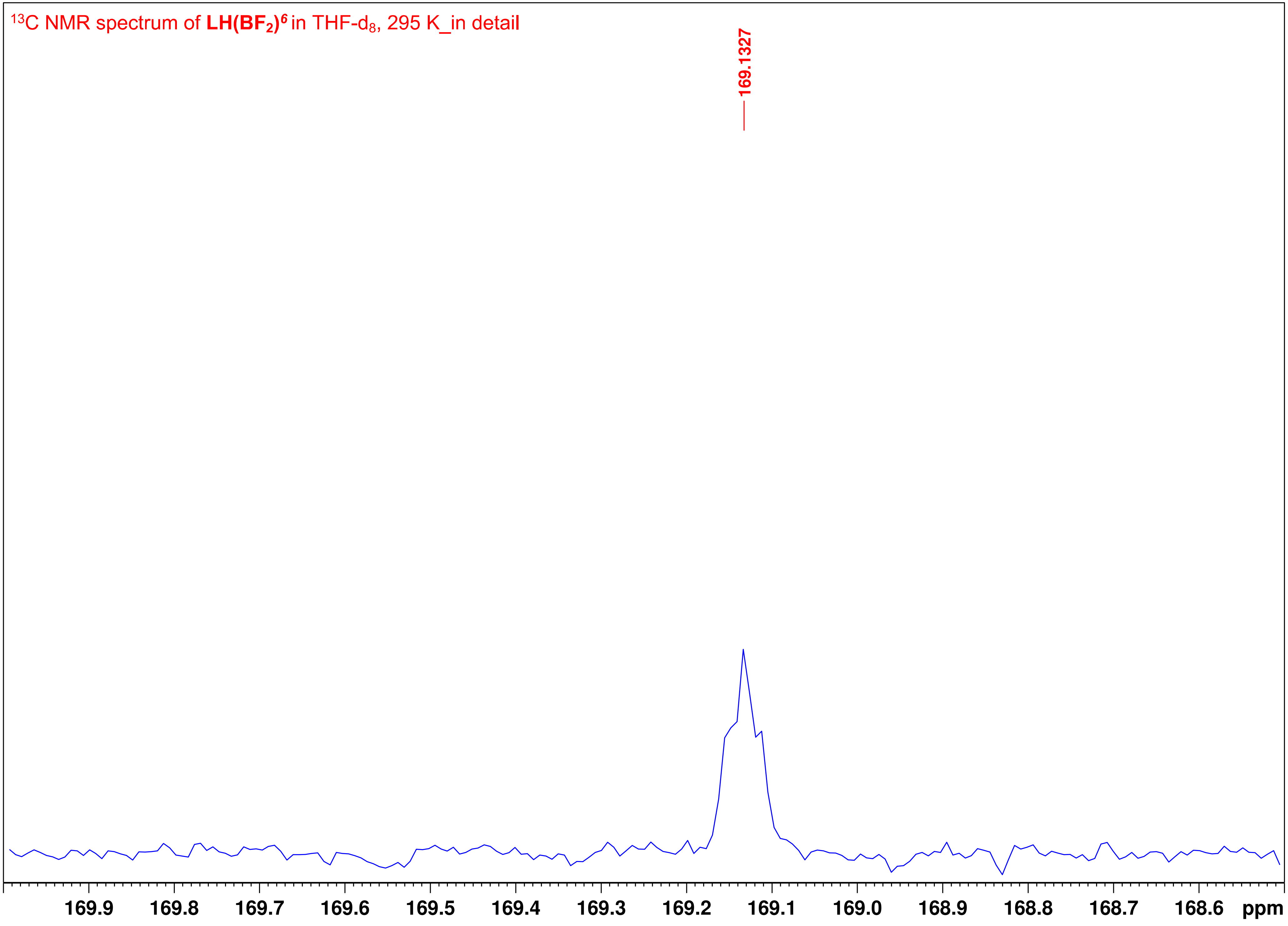
^{13}C NMR spectrum of **LH(BF$_2$)^6** in THF-d$_8$, 295 K_in detail
169.1327
169.9 169.8 169.7 169.6 169.5 169.4 169.3 169.2 169.1 169.0 168.9 168.8 168.7 168.6 ppm

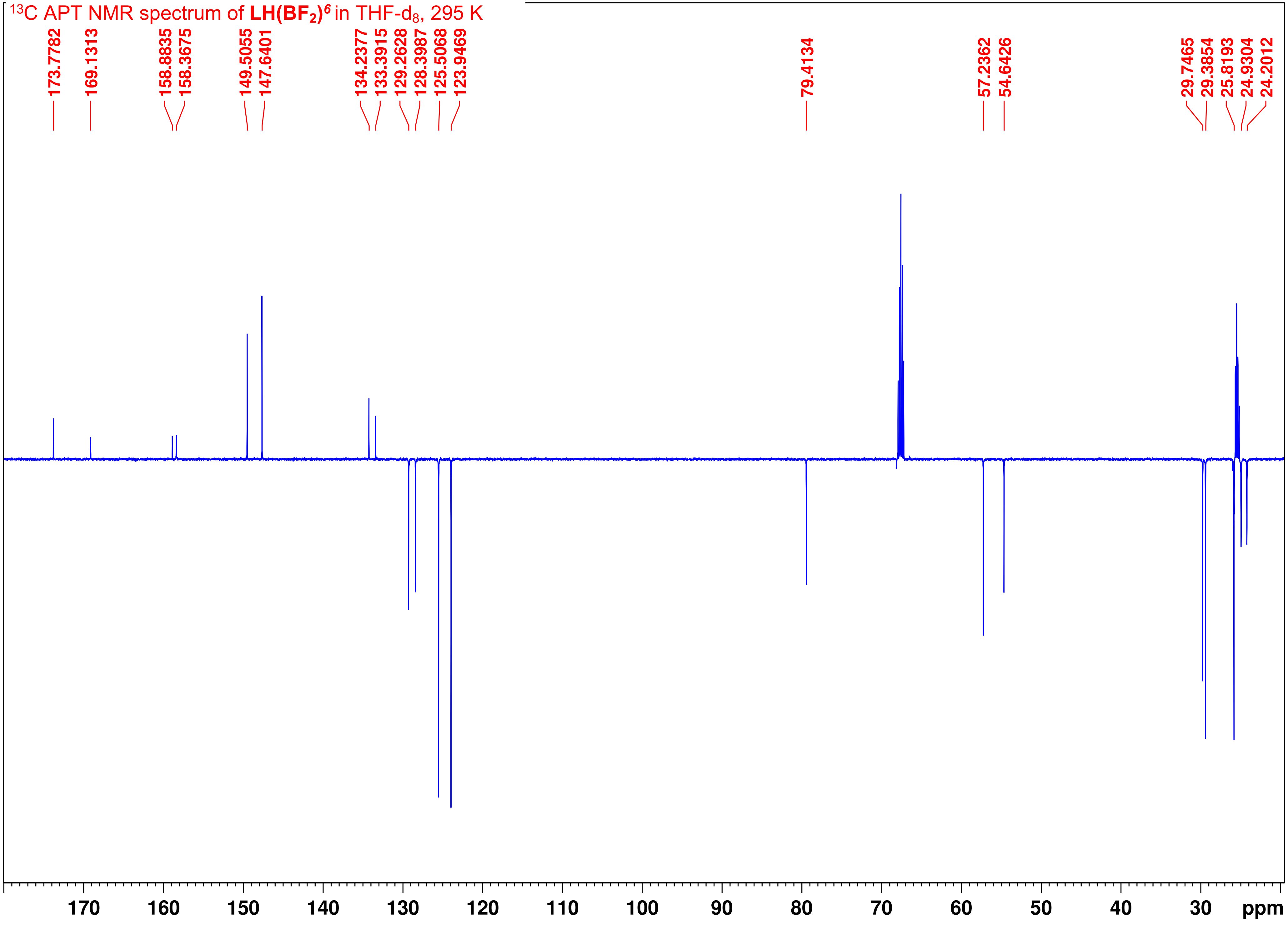

^{13}C APT NMR spectrum of **LH(BF$_2$)^6** in THF-d_8, 295 K
173.7782
169.1313
158.8835
158.3675
149.5055
147.6401
134.2377
133.3915
129.2628
128.3987
125.5068
123.9469
79.4134
57.2362
54.6426
29.7465
29.3854
25.8193
24.9304
24.2012
170
160
150
140
130
120
110
100
90
80
70
60
50
40
30
ppm

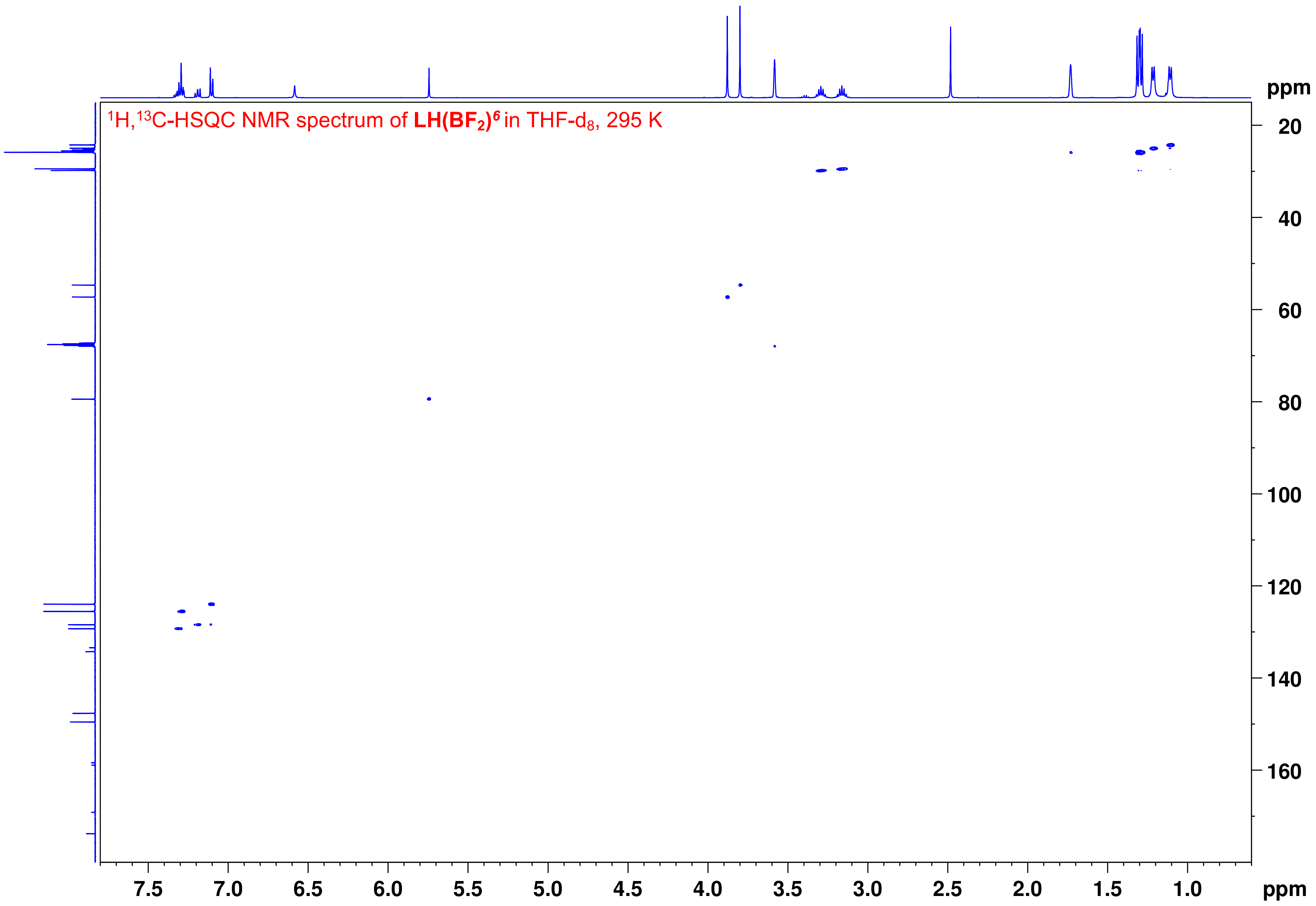

$^{1}H,^{13}C$-HSQC NMR spectrum of **LH(BF$_2$)^6** in THF-d_8, 295 K
ppm
20
40
60
80
100
120
140
160
7.5
7.0
6.5
6.0
5.5
5.0
4.5
4.0
3.5
3.0
2.5
2.0
1.5
1.0

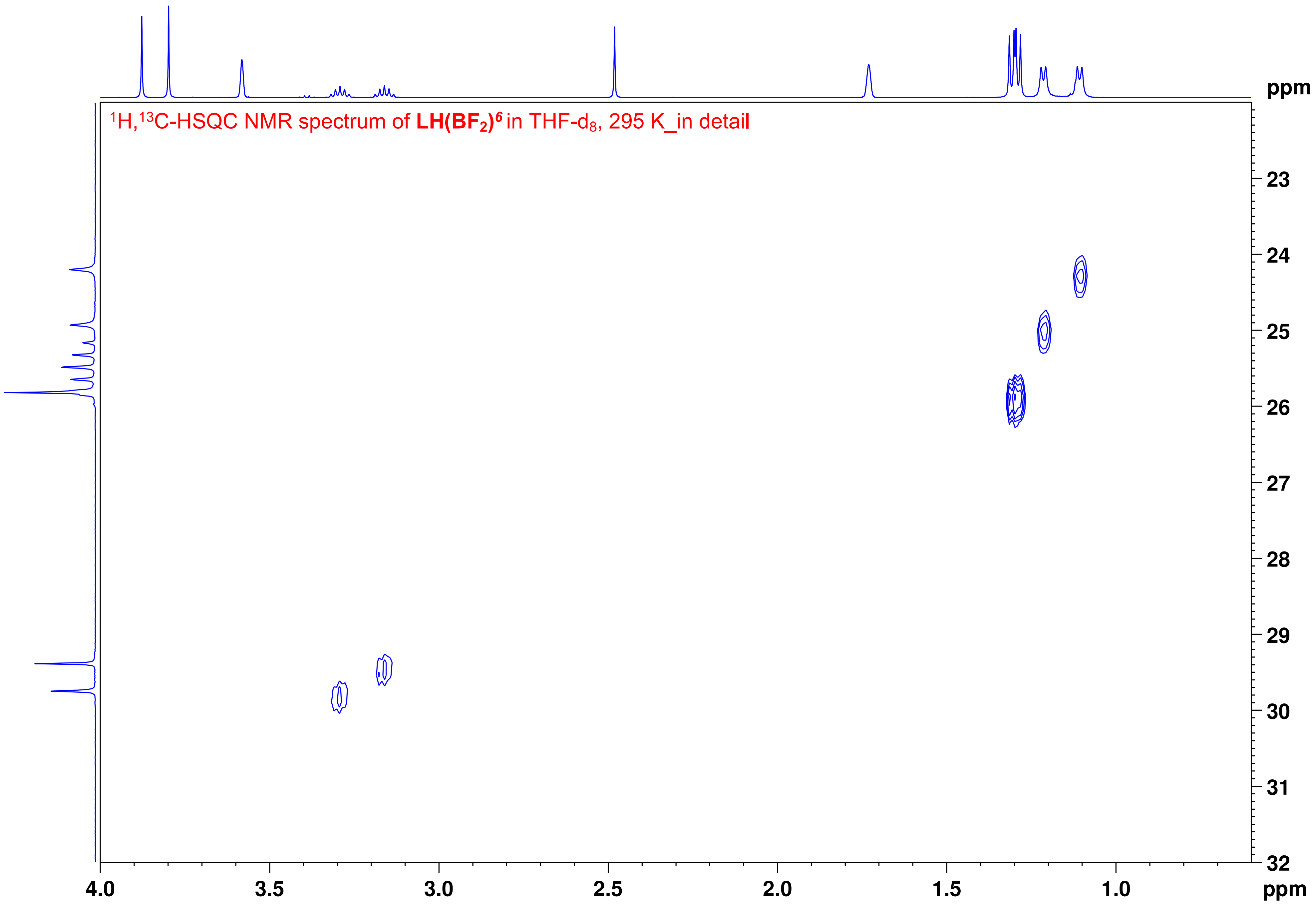

$^{1}H,^{13}C$-HSQC NMR spectrum of **LH(BF$_2$)^6** in THF-d_8, 295 K_in detail

^1H,^{13}C-HSQC NMR spectrum of **$LH(BF_2)^6$** in THF-d_8, 295 K_in detail

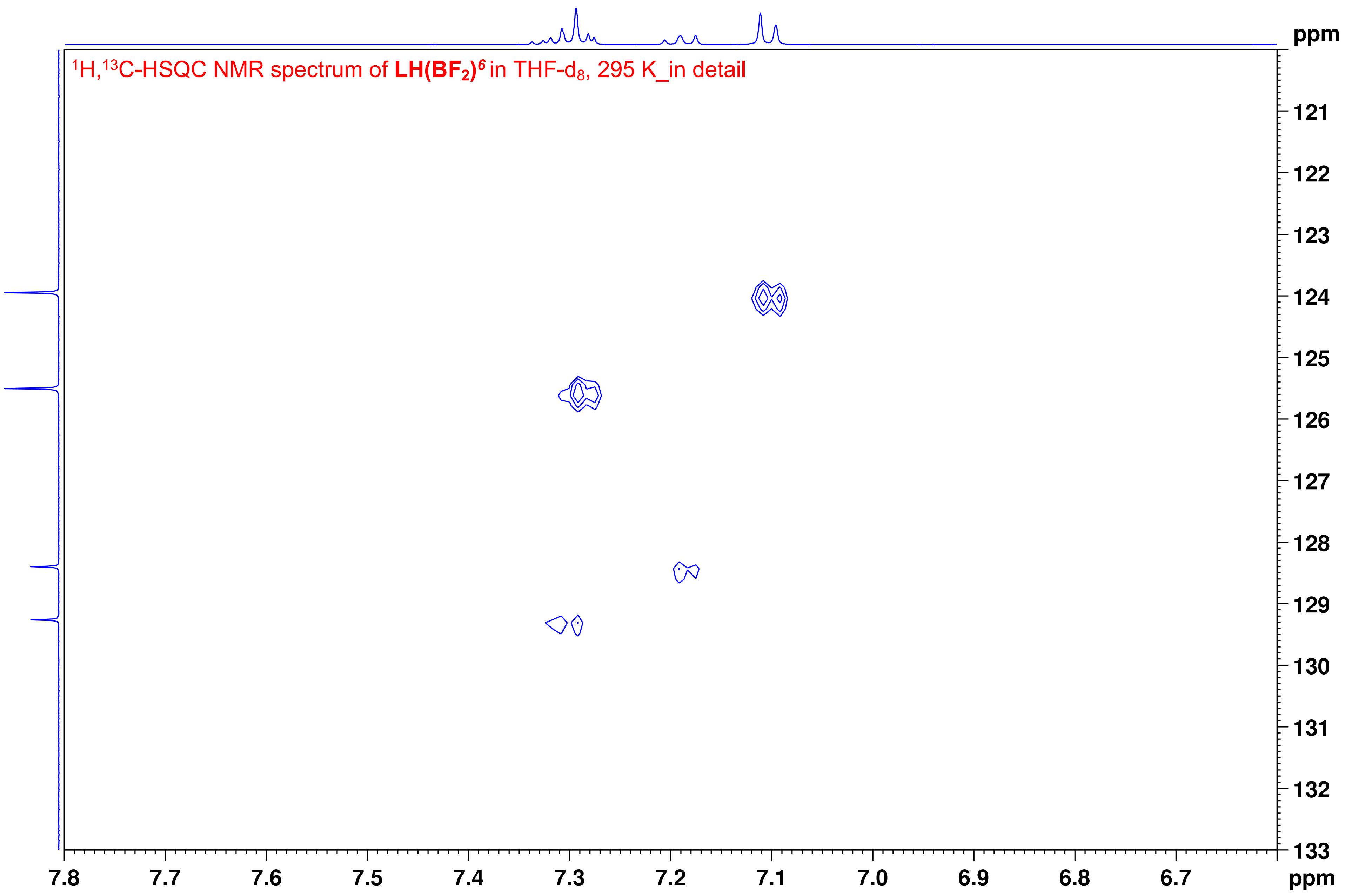

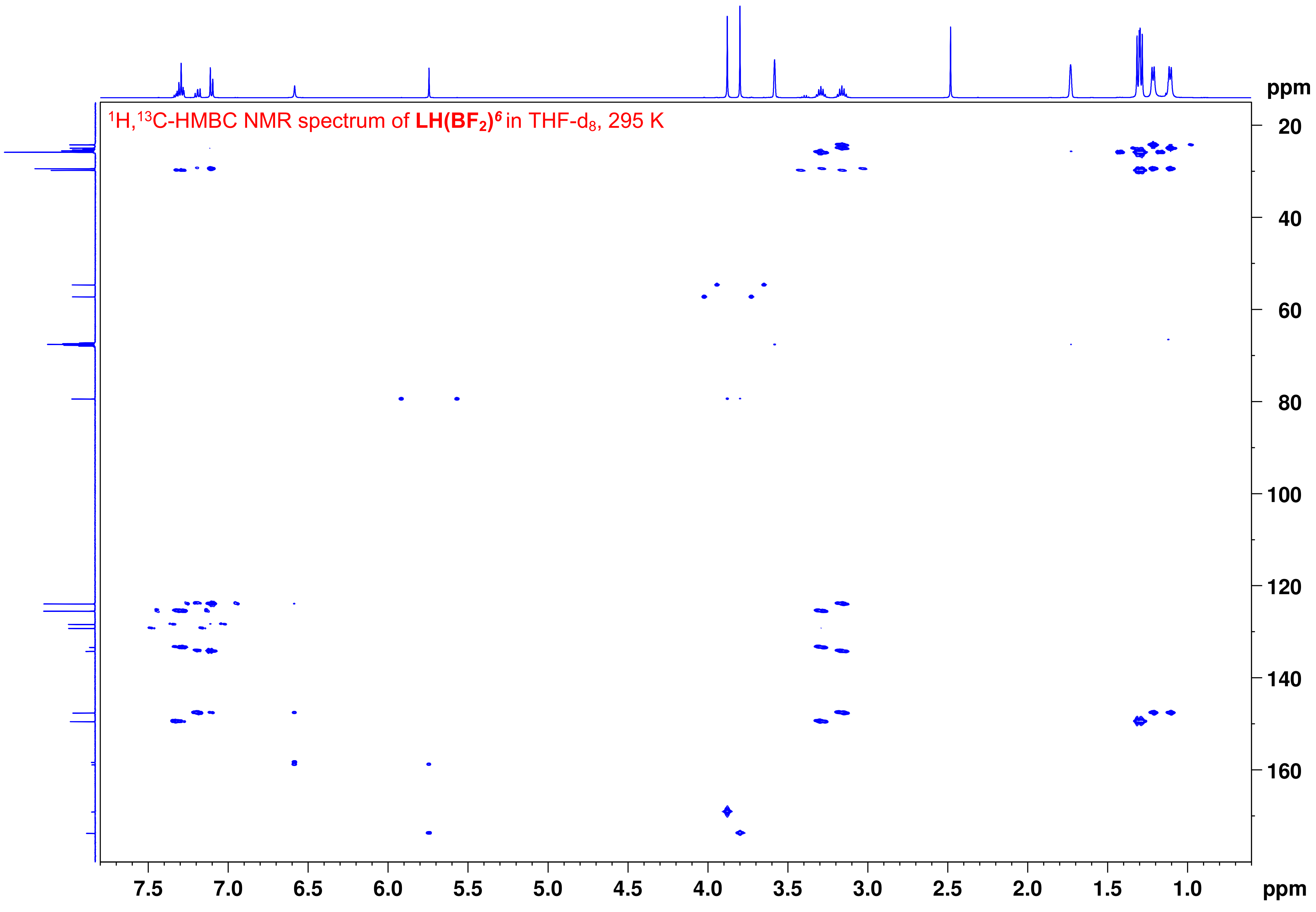

^{1}H,^{13}C-HMBC NMR spectrum of **LH(BF$_2$)^6** in THF-d$_8$, 295 K
ppm
20
40
60
80
100
120
140
160
7.5
7.0
6.5
6.0
5.5
5.0
4.5
4.0
3.5
3.0
2.5
2.0
1.5
1.0
ppm

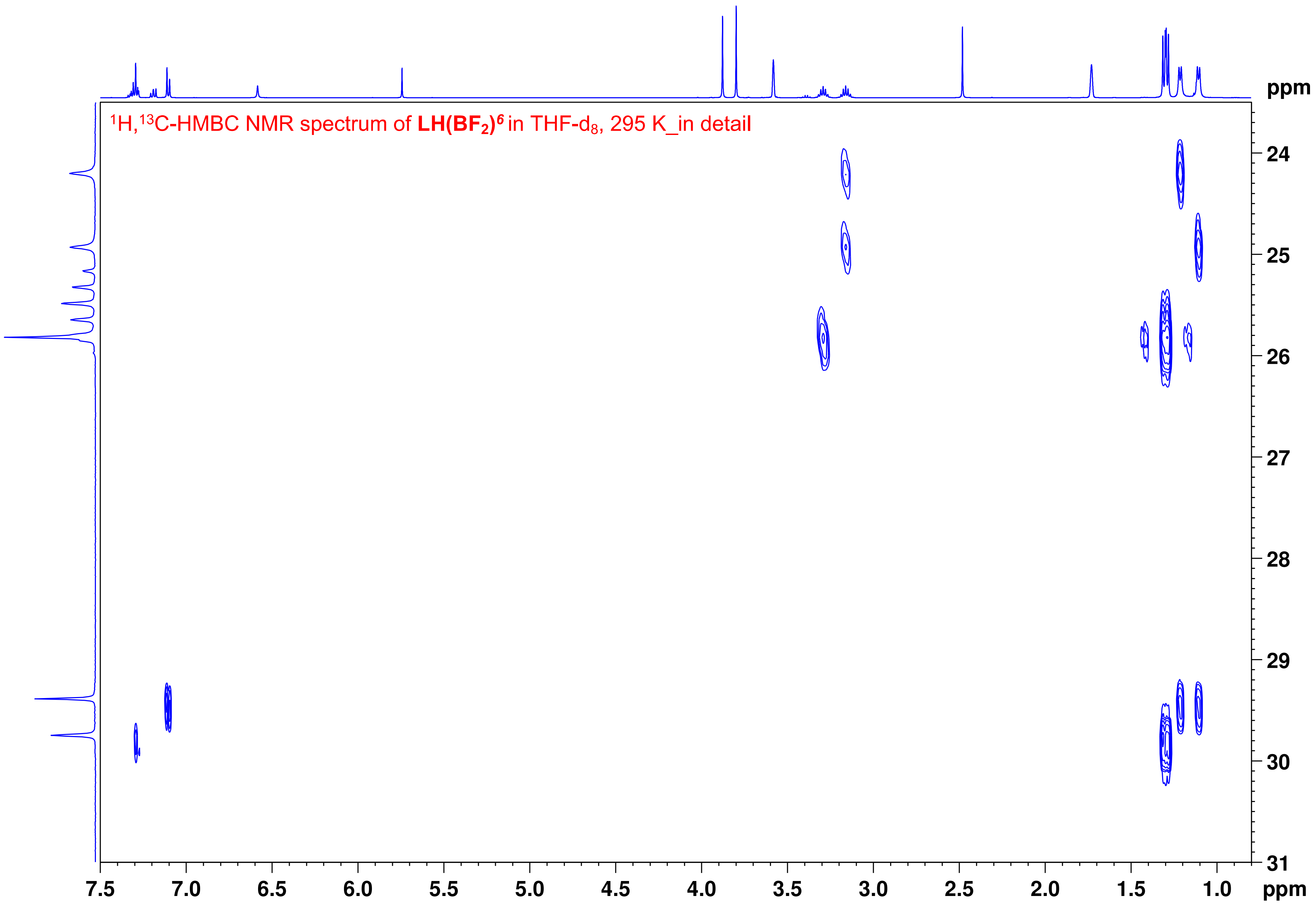

$^{1}H,^{13}C$-HMBC NMR spectrum of **LH(BF$_2$)^6** in THF-d$_8$, 295 K_in detail
ppm
7.5
7.0
6.5
6.0
5.5
5.0
4.5
4.0
3.5
3.0
2.5
2.0
1.5
1.0
24
25
26
27
28
29
30
31

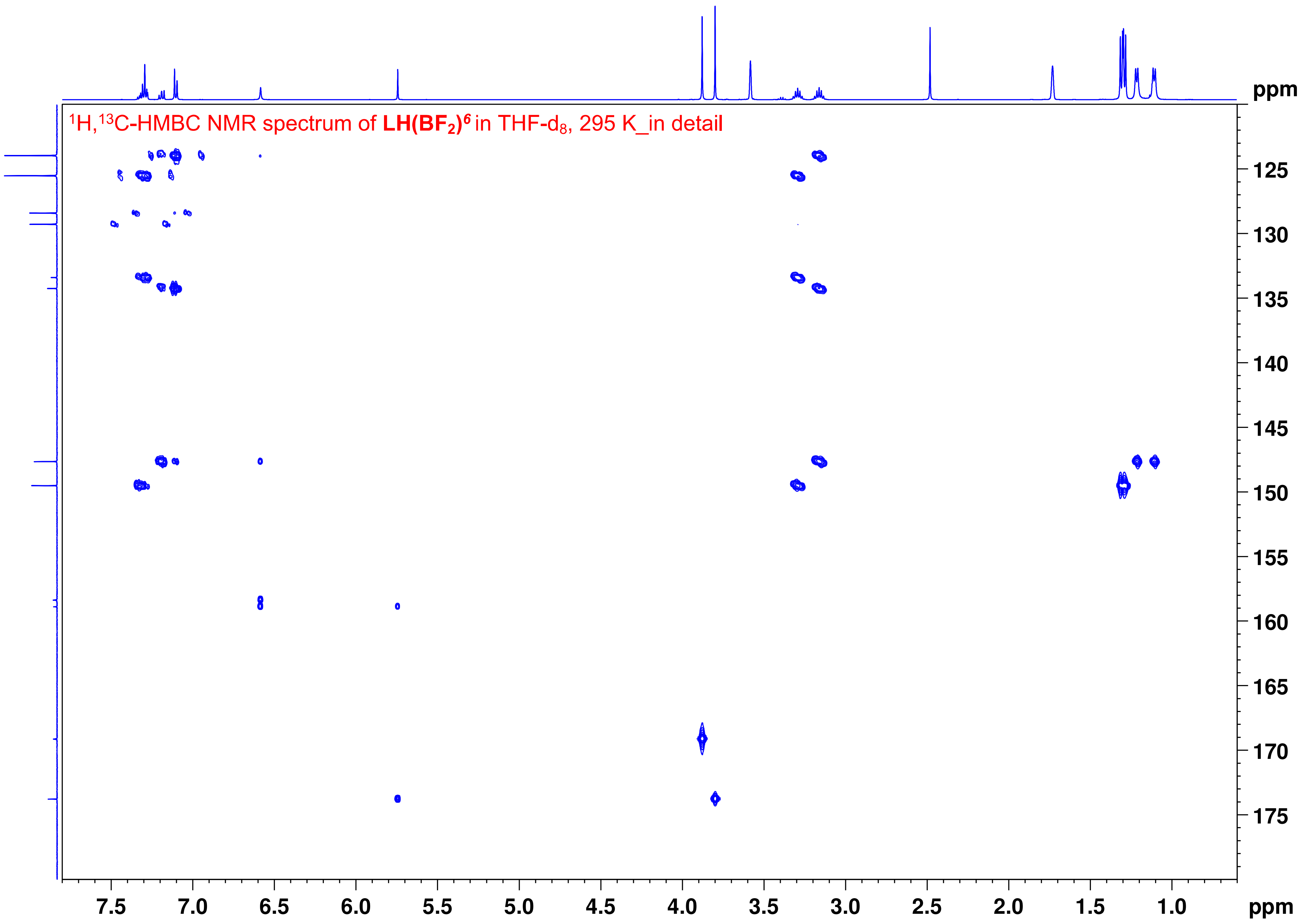
$^{1}H,^{13}C$-HMBC NMR spectrum of **LH(BF$_2$)^6** in THF-d_8, 295 K_in detail
ppm
125
130
135
140
145
150
155
160
165
170
175
ppm
7.5
7.0
6.5
6.0
5.5
5.0
4.5
4.0
3.5
3.0
2.5
2.0
1.5
1.0
ppm

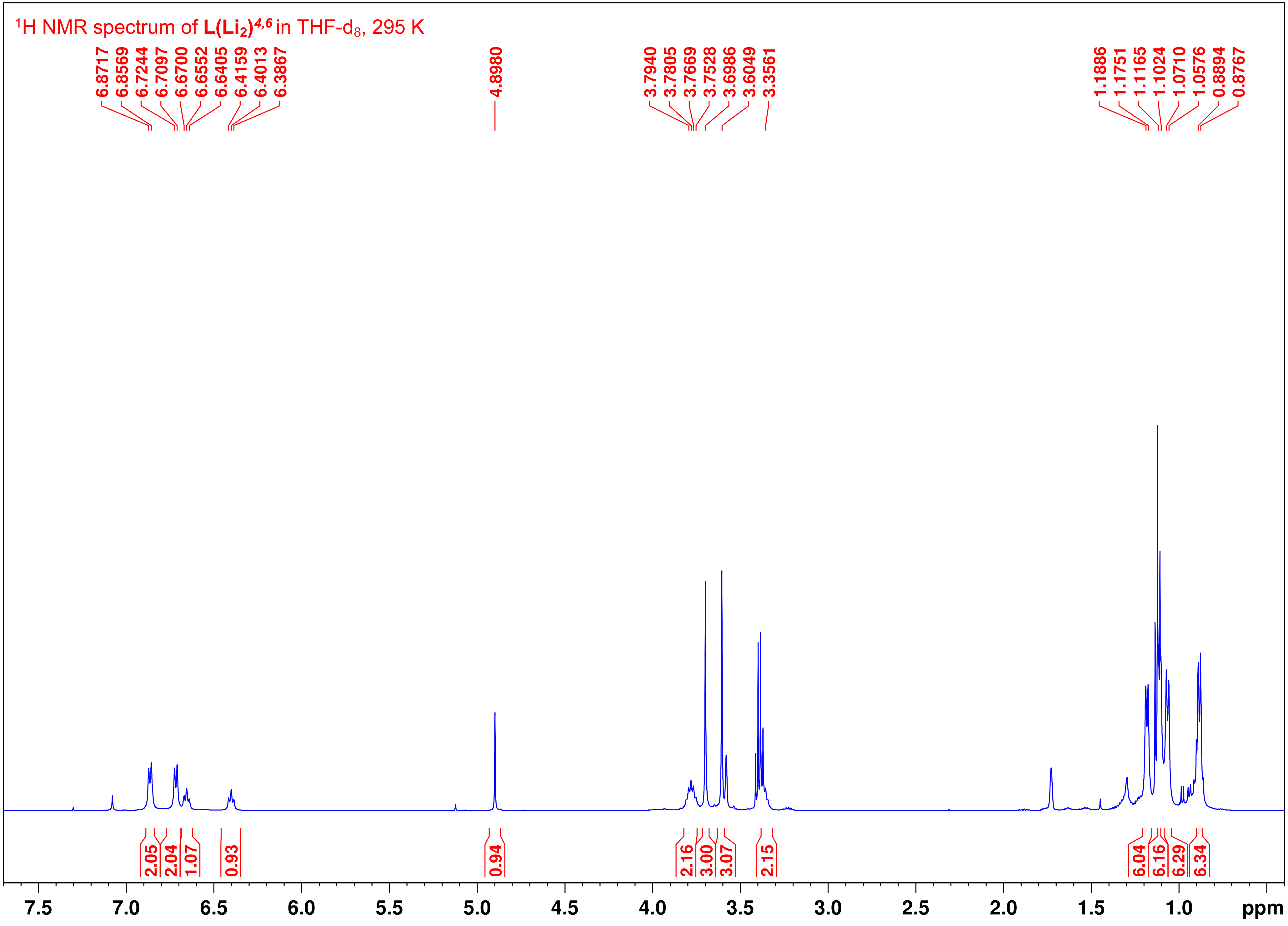

¹H NMR spectrum of L(Li₂)4,6 in THF-d8, 295 K
6.8717
6.8569
6.7244
6.7097
6.6700
6.6552
6.6405
6.4159
6.4013
6.3867
4.8980
3.7940
3.7805
3.7669
3.7528
3.6986
3.6049
3.3561
1.1886
1.1751
1.1165
1.1024
1.0710
1.0576
0.8894
0.8767
2.05
2.04
1.07
0.93
0.94
2.16
3.00
3.07
2.15
6.04
6.16
6.29
6.34
7.5
7.0
6.5
6.0
5.5
5.0
4.5
4.0
3.5
3.0
2.5
2.0
1.5
1.0
ppm

^7Li NMR spectrum of **L(Li$_2$)**^{4,6} in THF-d_8, 295 K

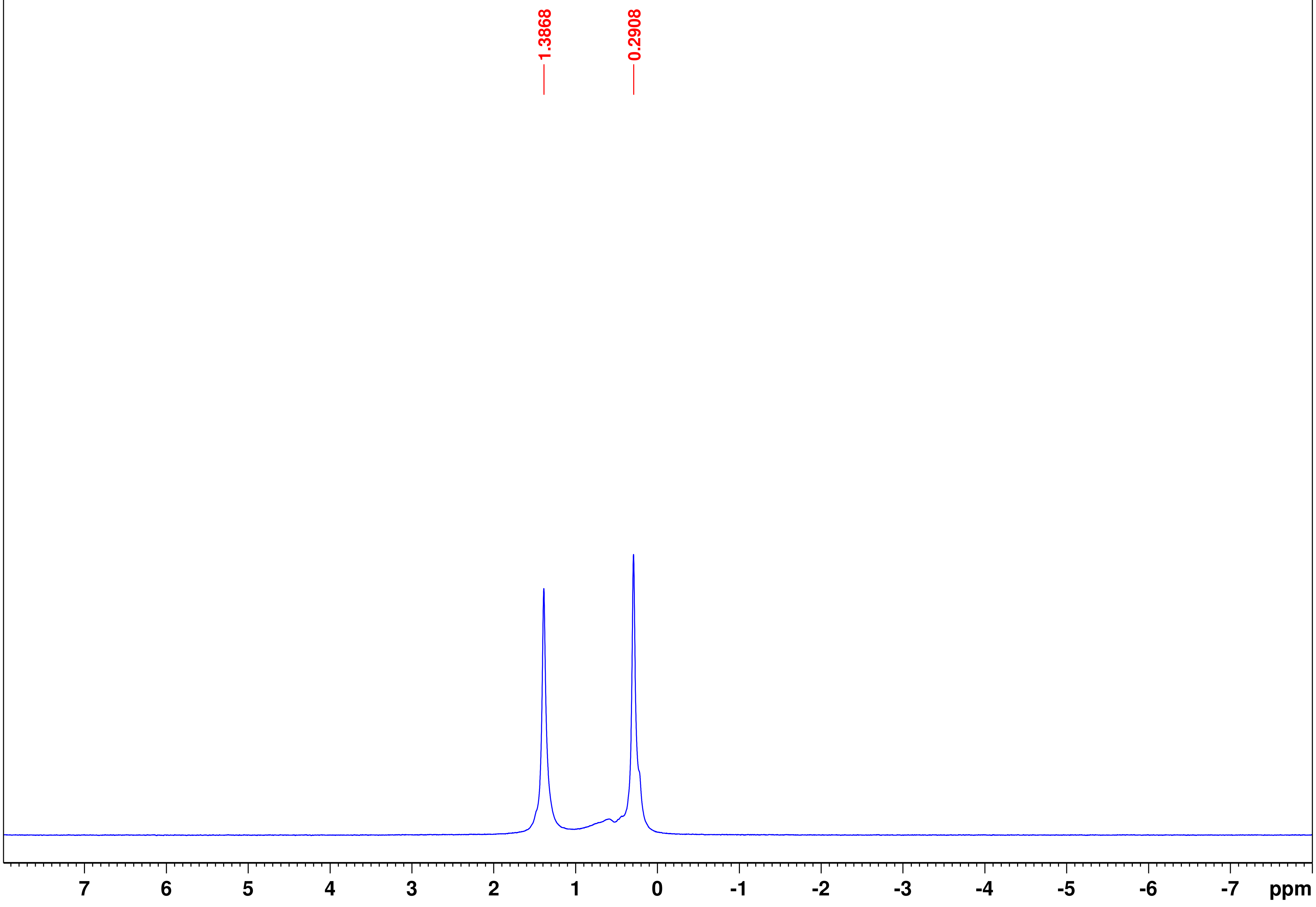

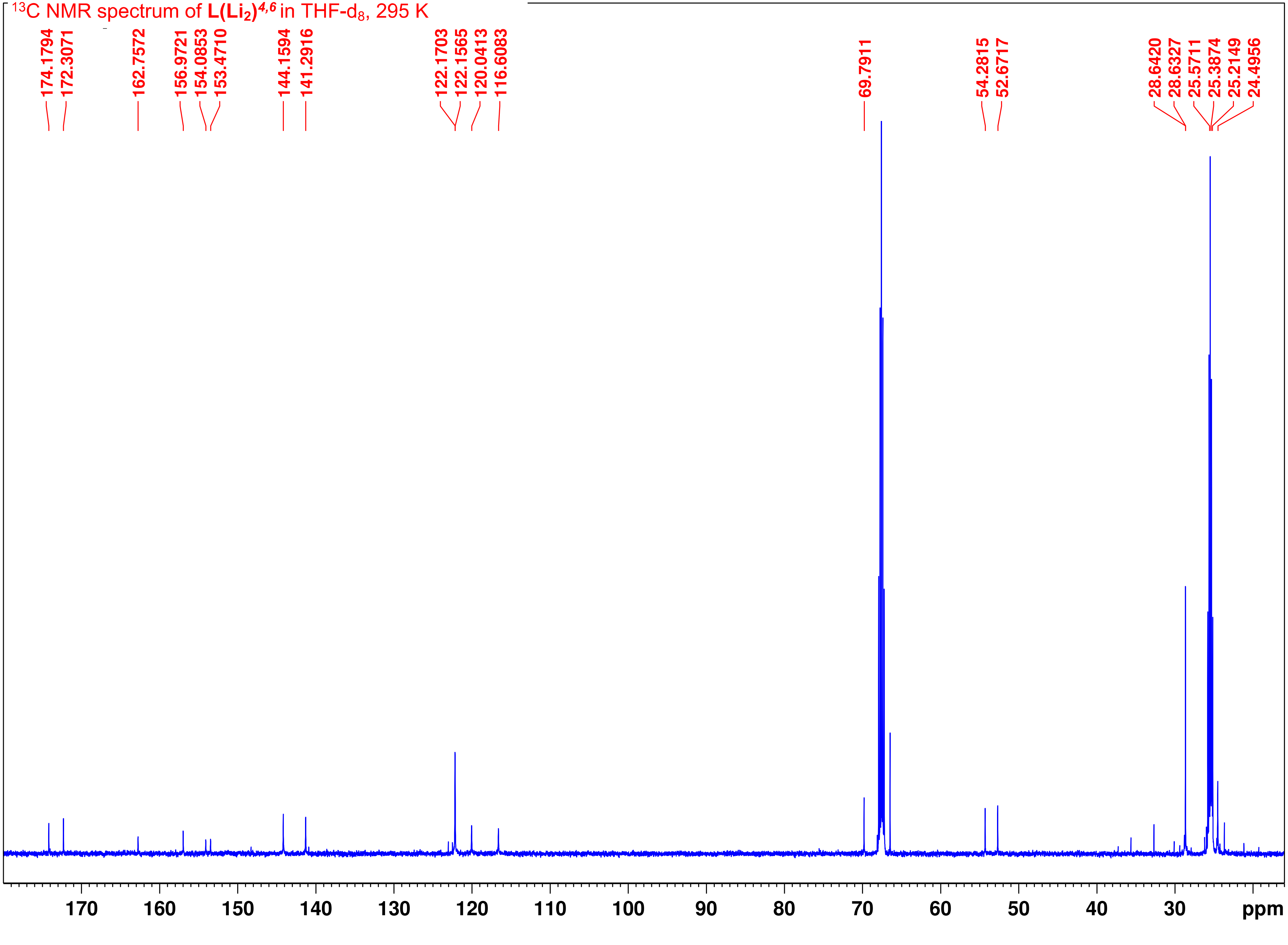
^{13}C NMR spectrum of **L(Li$_2$)**^{4,6} in THF-d$_8$, 295 K
174.1794
172.3071
162.7572
156.9721
154.0853
153.4710
144.1594
141.2916
122.1703
122.1565
120.0413
116.6083
69.7911
54.2815
52.6717
28.6420
28.6327
25.5711
25.3874
25.2149
24.4956
170 160 150 140 130 120 110 100 90 80 70 60 50 40 30 ppm

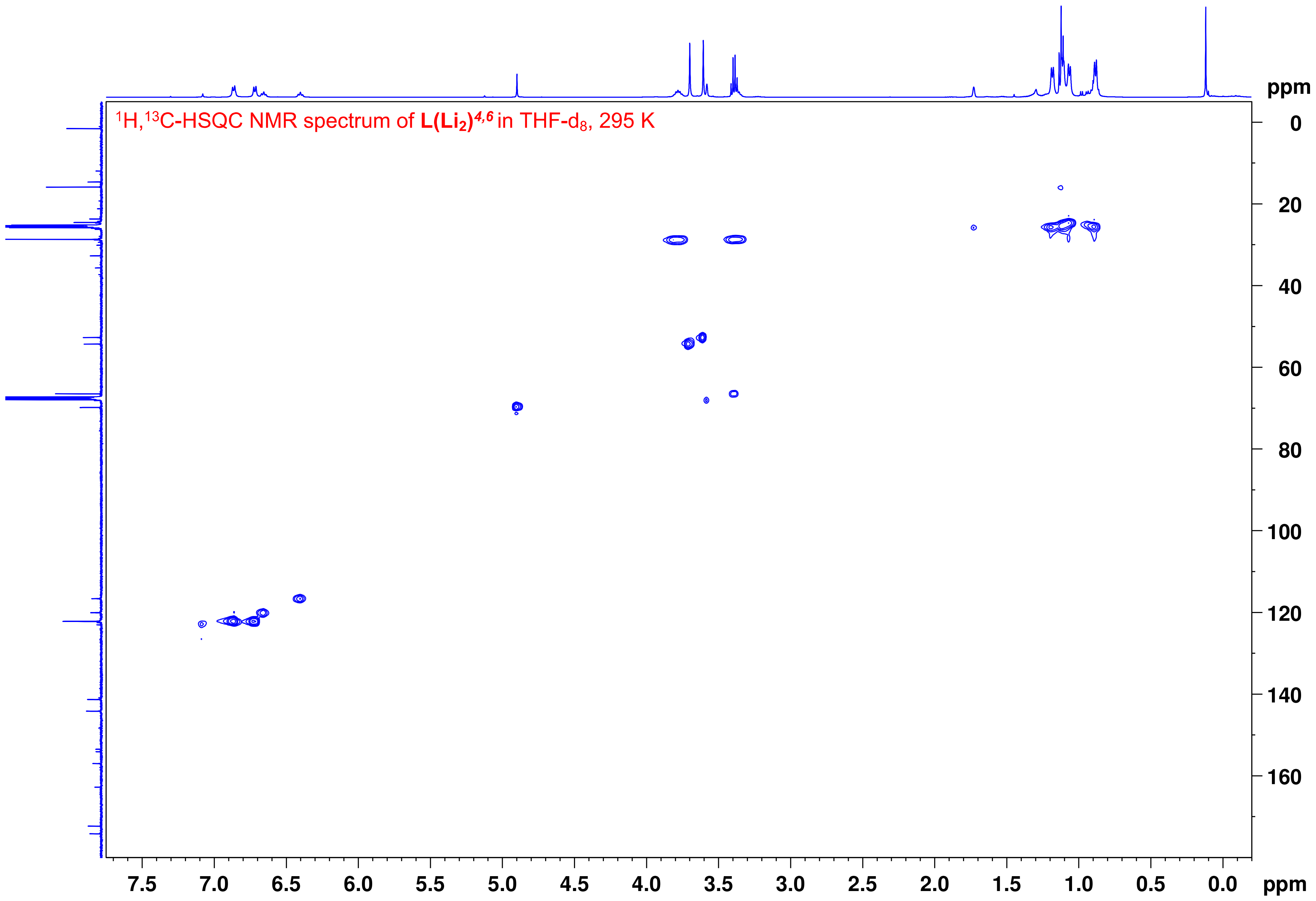

^{1}H,^{13}C-HSQC NMR spectrum of **L(Li$_2$)*4,6*** in THF-d_8, 295 K
ppm
0
20
40
60
80
100
120
140
160
7.5
7.0
6.5
6.0
5.5
5.0
4.5
4.0
3.5
3.0
2.5
2.0
1.5
1.0
0.5
0.0
ppm

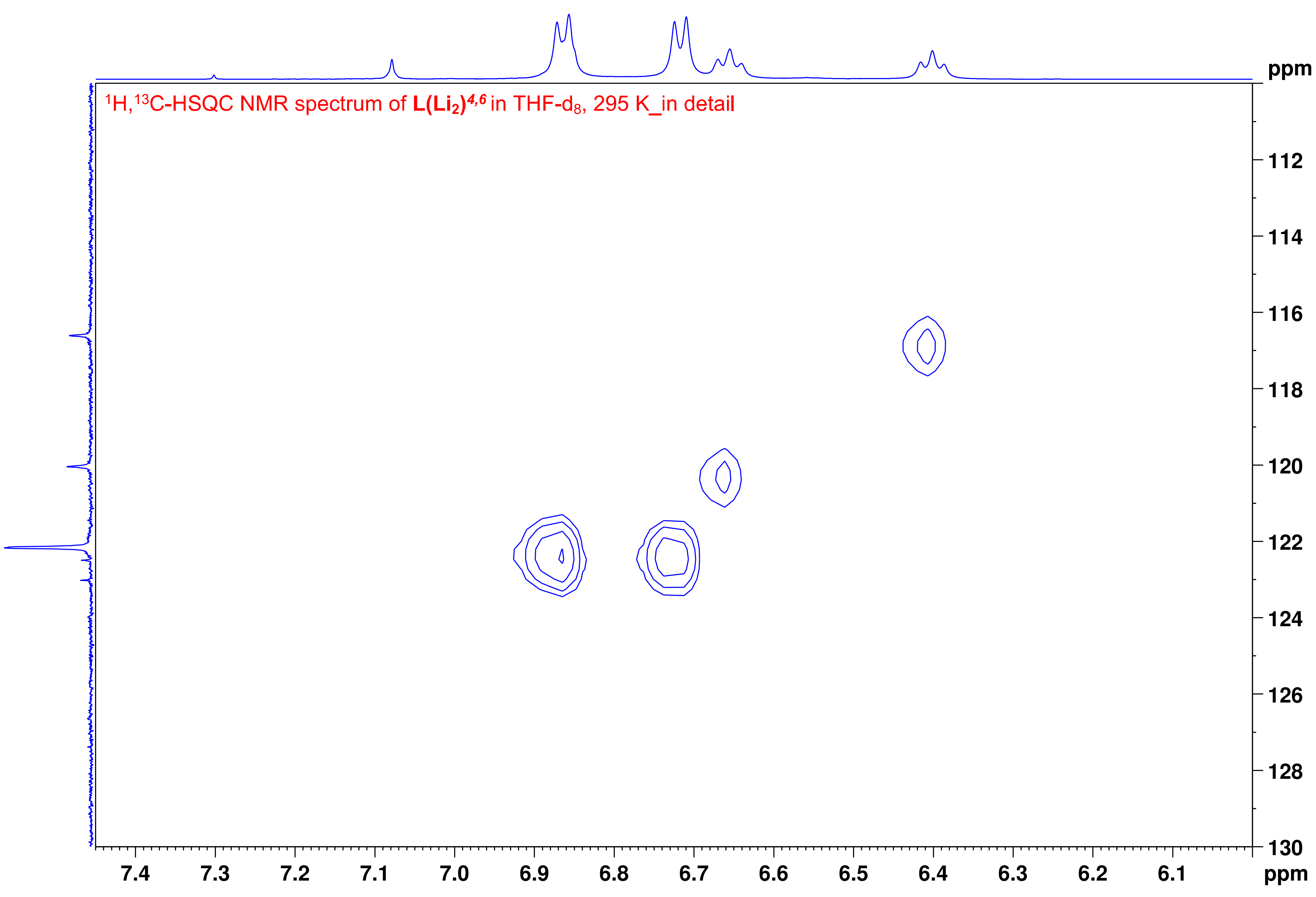

$^{1}H,^{13}C$-HSQC NMR spectrum of **L(Li$_2$)**^{4,6} in THF-d$_8$, 295 K_in detail
ppm
112
114
116
118
120
122
124
126
128
130
ppm
7.4
7.3
7.2
7.1
7.0
6.9
6.8
6.7
6.6
6.5
6.4
6.3
6.2
6.1
ppm

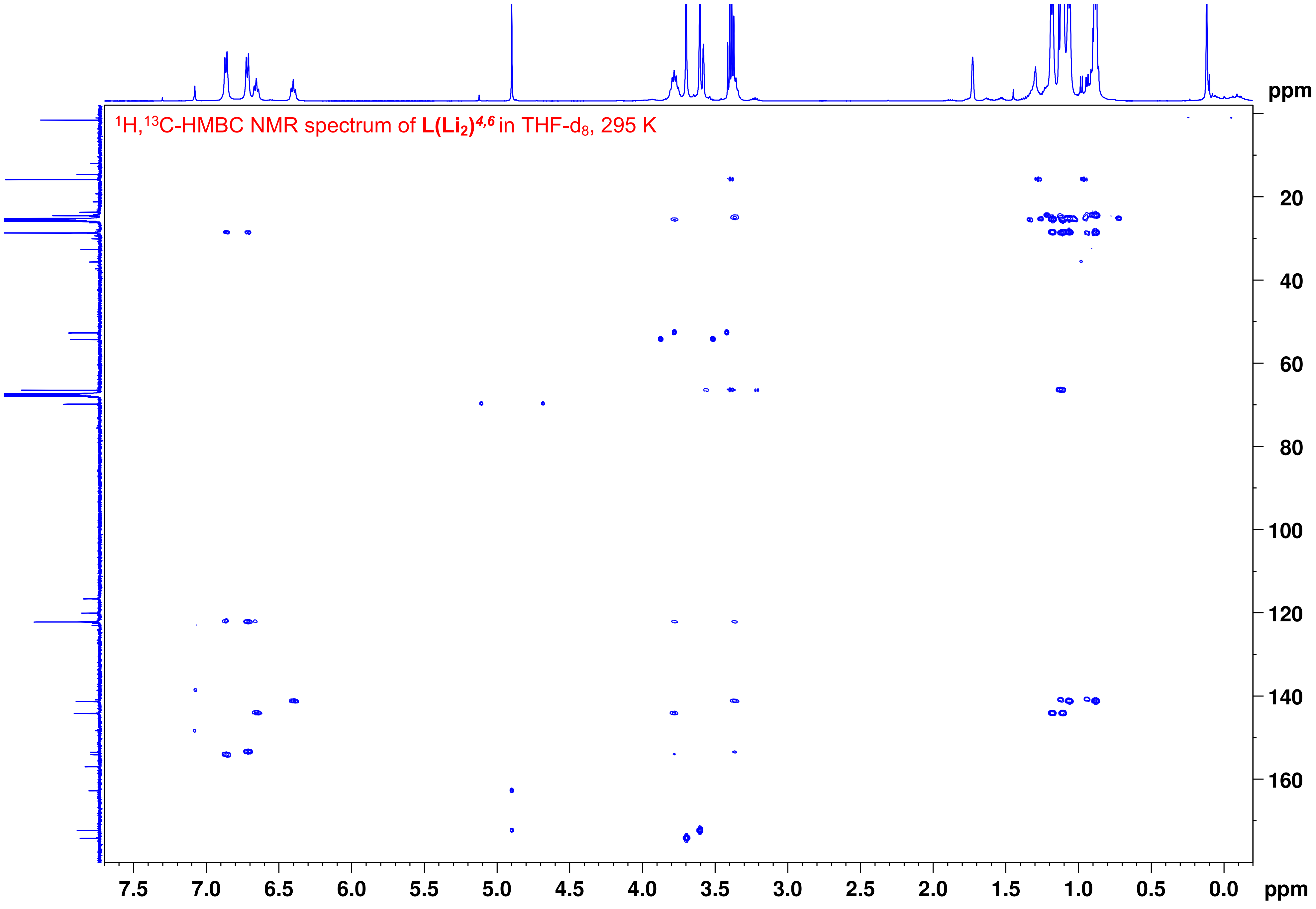

1H,13C-HMBC NMR spectrum of L(Li2)4,6 in THF-d8, 295 K
ppm
20
40
60
80
100
120
140
160
7.5
7.0
6.5
6.0
5.5
5.0
4.5
4.0
3.5
3.0
2.5
2.0
1.5
1.0
0.5
0.0

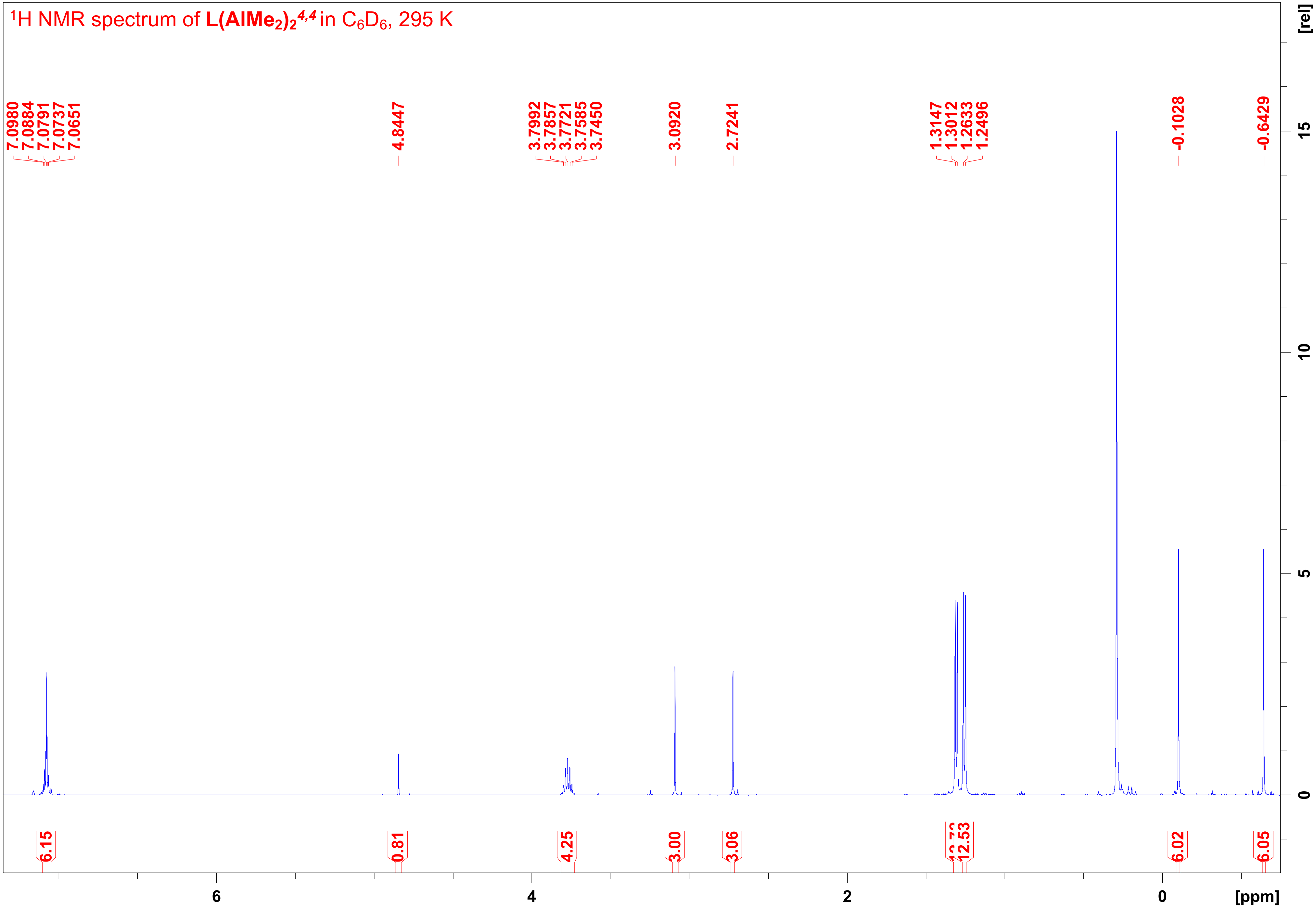

^{1}H NMR spectrum of $\mathbf{L(AlMe_2)_2}^{4,4}$ in C_6D_6, 295 K
7.0980
7.0884
7.0791
7.0737
7.0651
4.8447
3.7992
3.7857
3.7721
3.7585
3.7450
3.0920
2.7241
1.3147
1.3012
1.2633
1.2496
-0.1028
-0.6429
6.15
0.81
4.25
3.00
3.06
12.53
6.02
6.05
[rel]
[ppm]

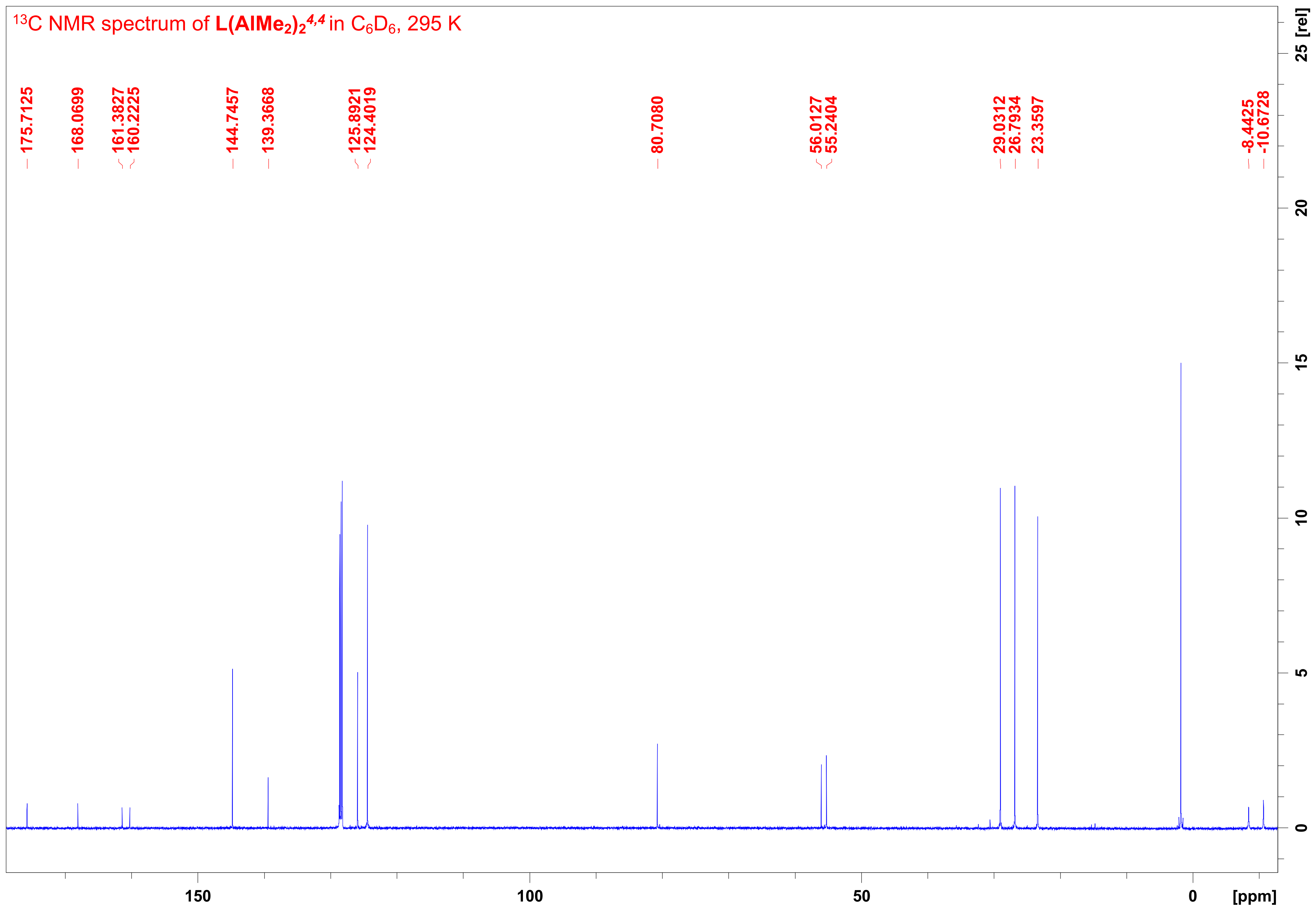

13C NMR spectrum of L(AlMe2)2 4,4 in C6D6, 295 K
175.7125
168.0699
161.3827
160.2225
144.7457
139.3668
125.8921
124.4019
80.7080
56.0127
55.2404
29.0312
26.7934
23.3597
-8.4425
-10.6728
150
100
50
0
[ppm]
25 [rel]
20
15
10
5
0

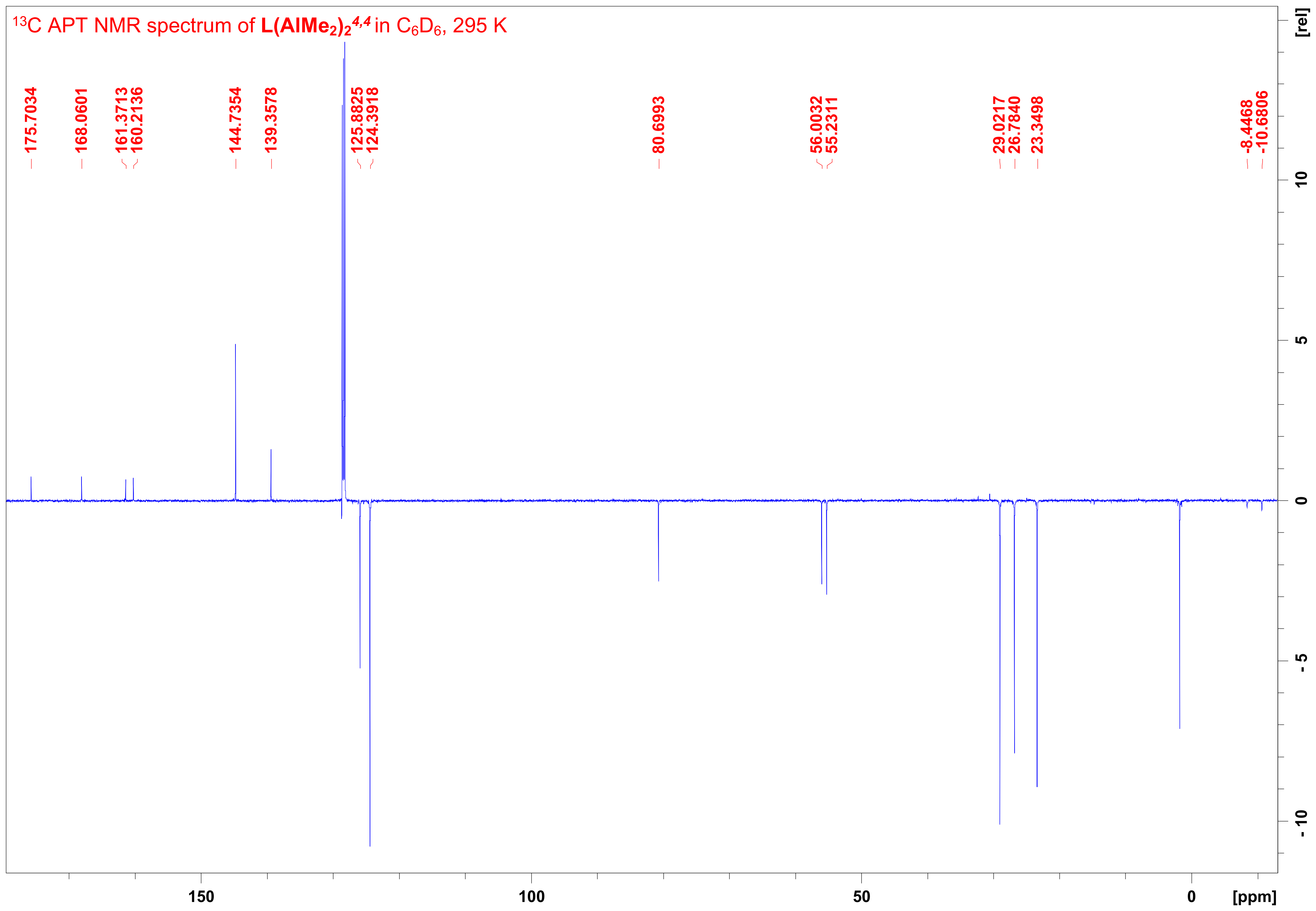

^{13}C APT NMR spectrum of **$L(AlMe_2)_2$**^{4,4} in C_6D_6, 295 K
175.7034
168.0601
161.3713
160.2136
144.7354
139.3578
125.8825
124.3918
80.6993
56.0032
55.2311
29.0217
26.7840
23.3498
-8.4468
-10.6806
[rel]
10
5
0
- 5
- 10
150
100
50
0
[ppm]

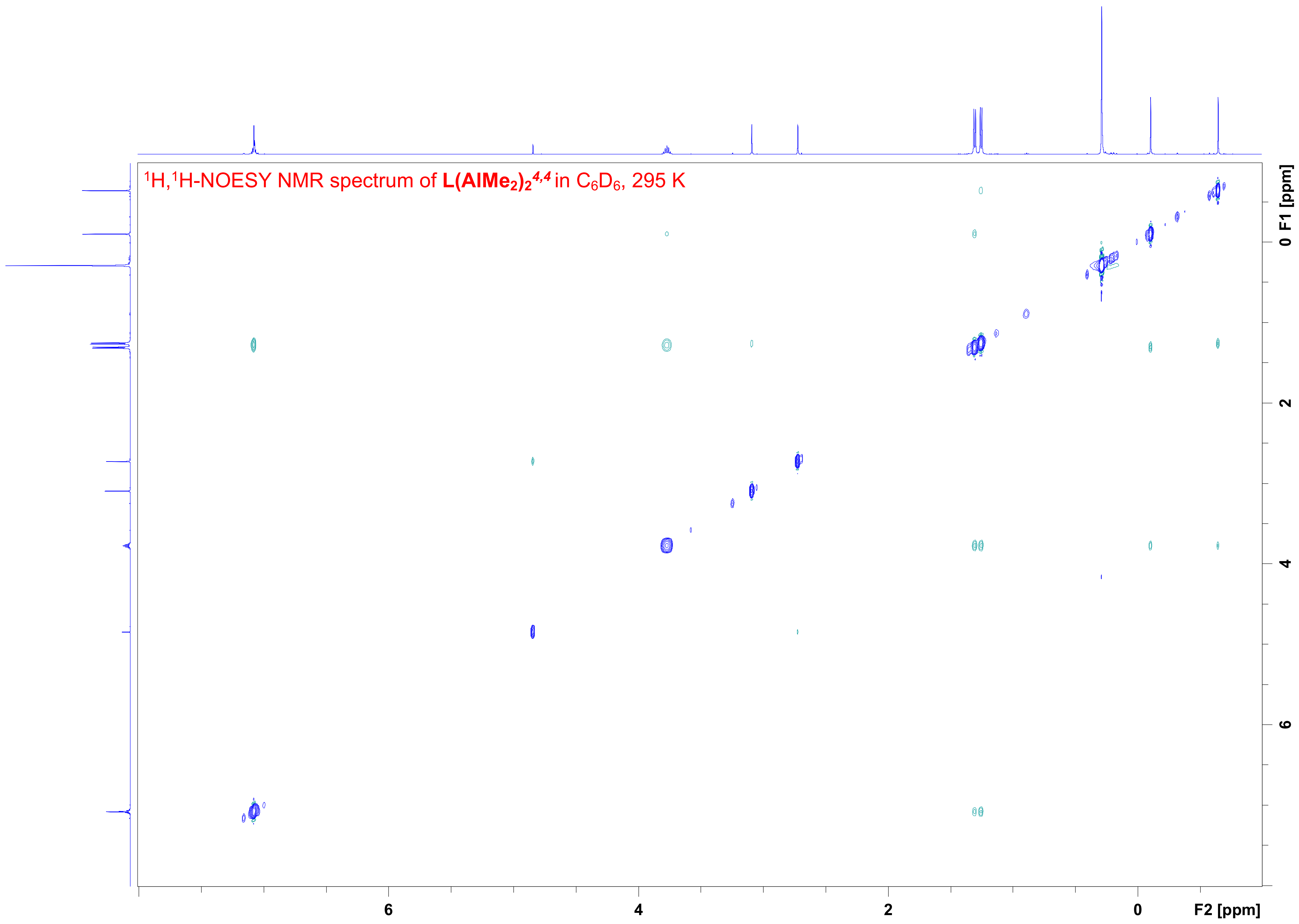

$^1H,^1H$-NOESY NMR spectrum of **L(AlMe$_2$)$_2$**^{4,4} in C_6D_6, 295 K
F1 [ppm]
F2 [ppm]

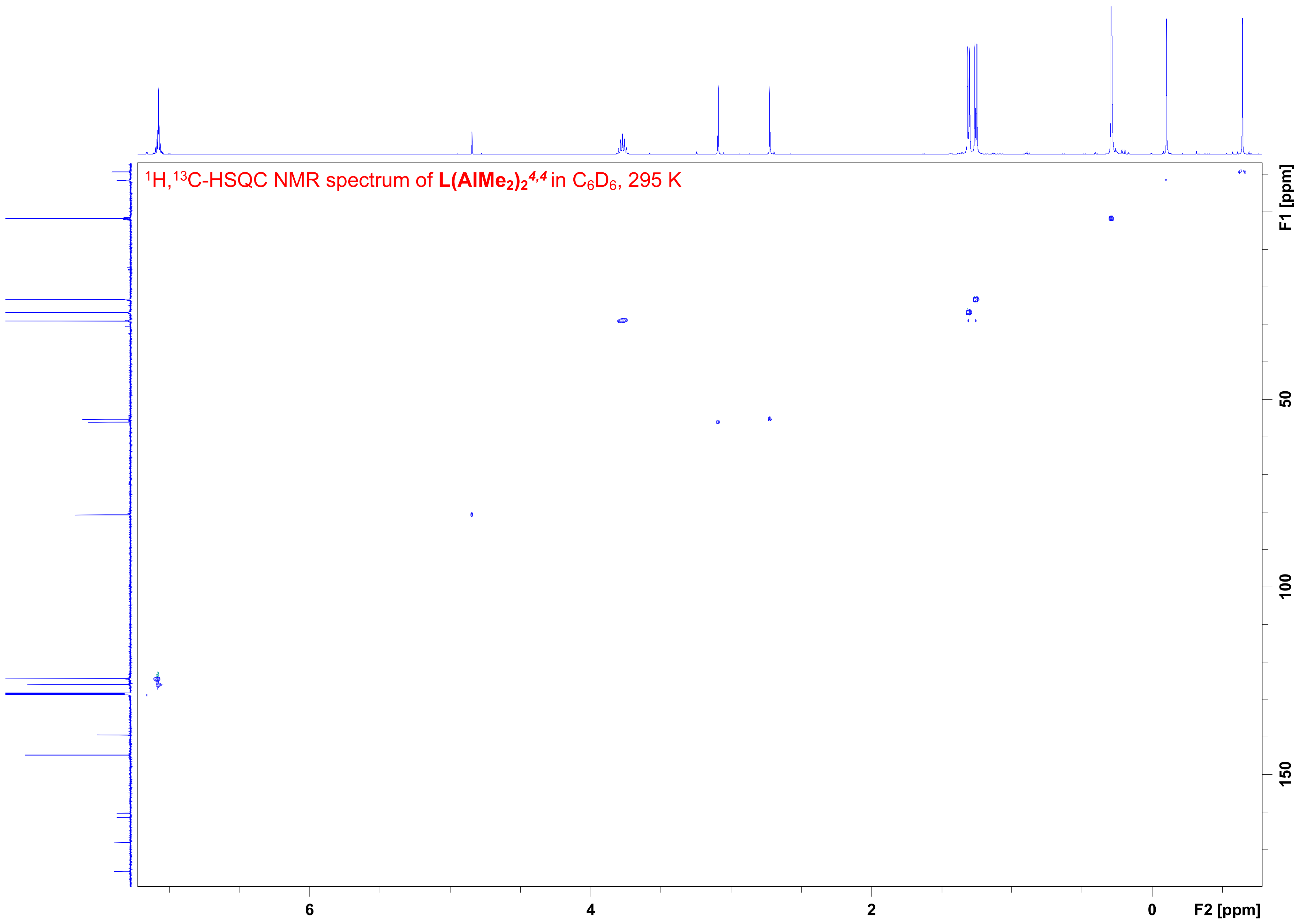

$^{1}H,^{13}C$-HSQC NMR spectrum of **L(AlMe$_2$)$_2$**^{4,4} in C_6D_6, 295 K
F1 [ppm]
50
100
150
6
4
2
0
F2 [ppm]

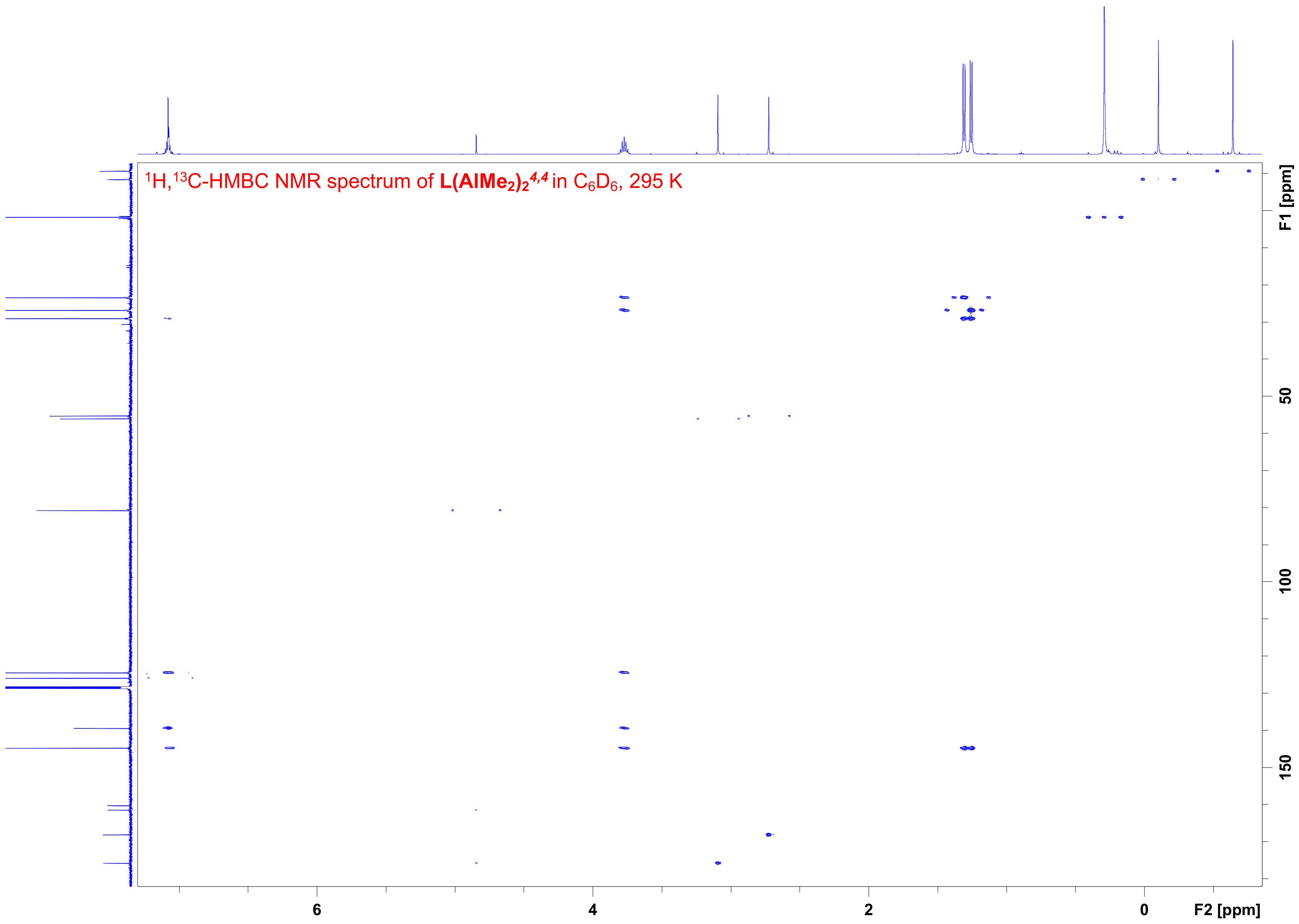

^{1}H,^{13}C-HMBC NMR spectrum of **L(AlMe$_2$)$_2$**^{4,4} in C_6D_6, 295 K
F1 [ppm]
50
100
150
6
4
2
0
F2 [ppm]

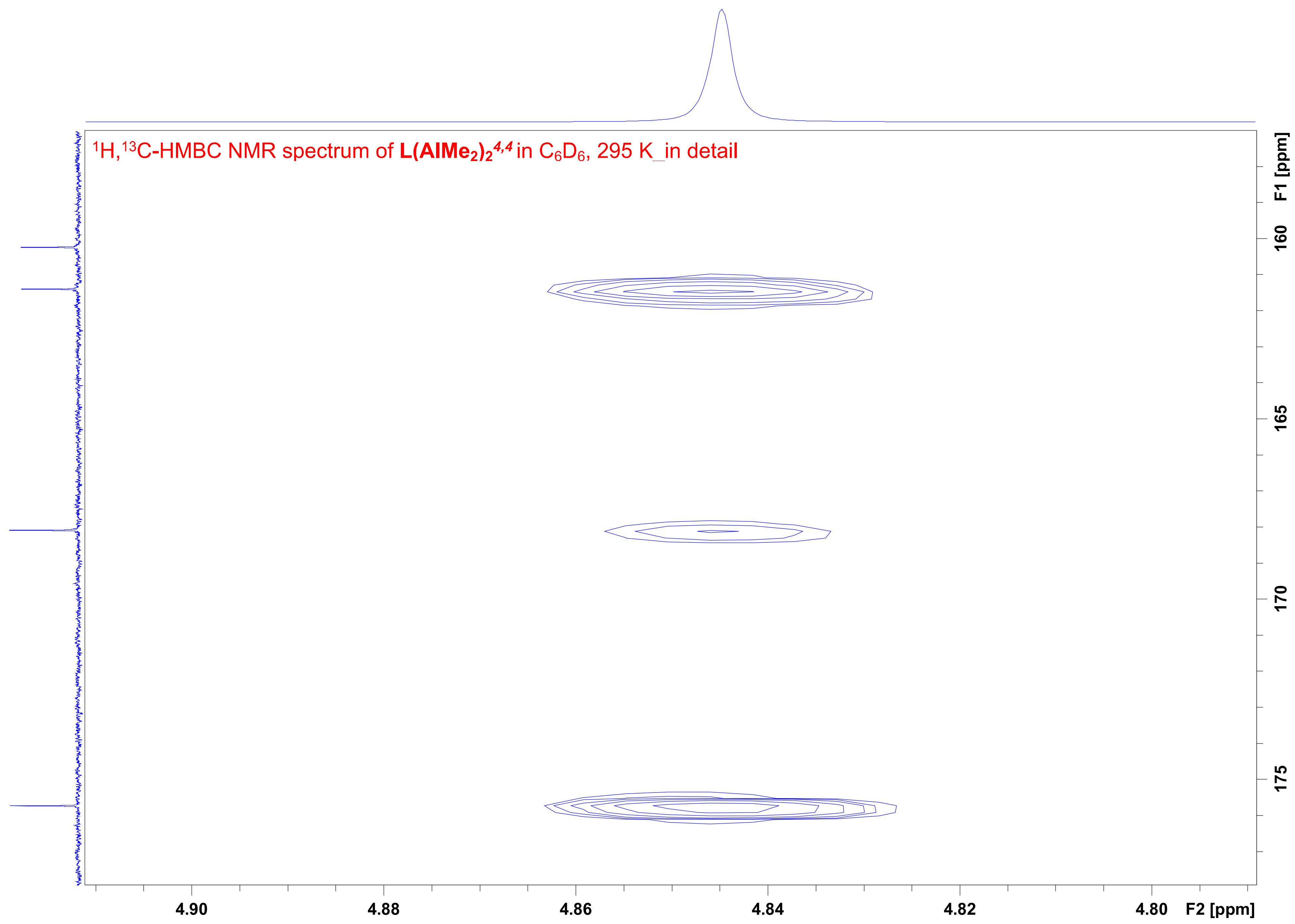
1H,13C-HMBC NMR spectrum of L(AlMe2)2 4,4 in C6D6, 295 K_in detail
F1 [ppm]
160
165
170
175
4.90
4.88
4.86
4.84
4.82
4.80
F2 [ppm]

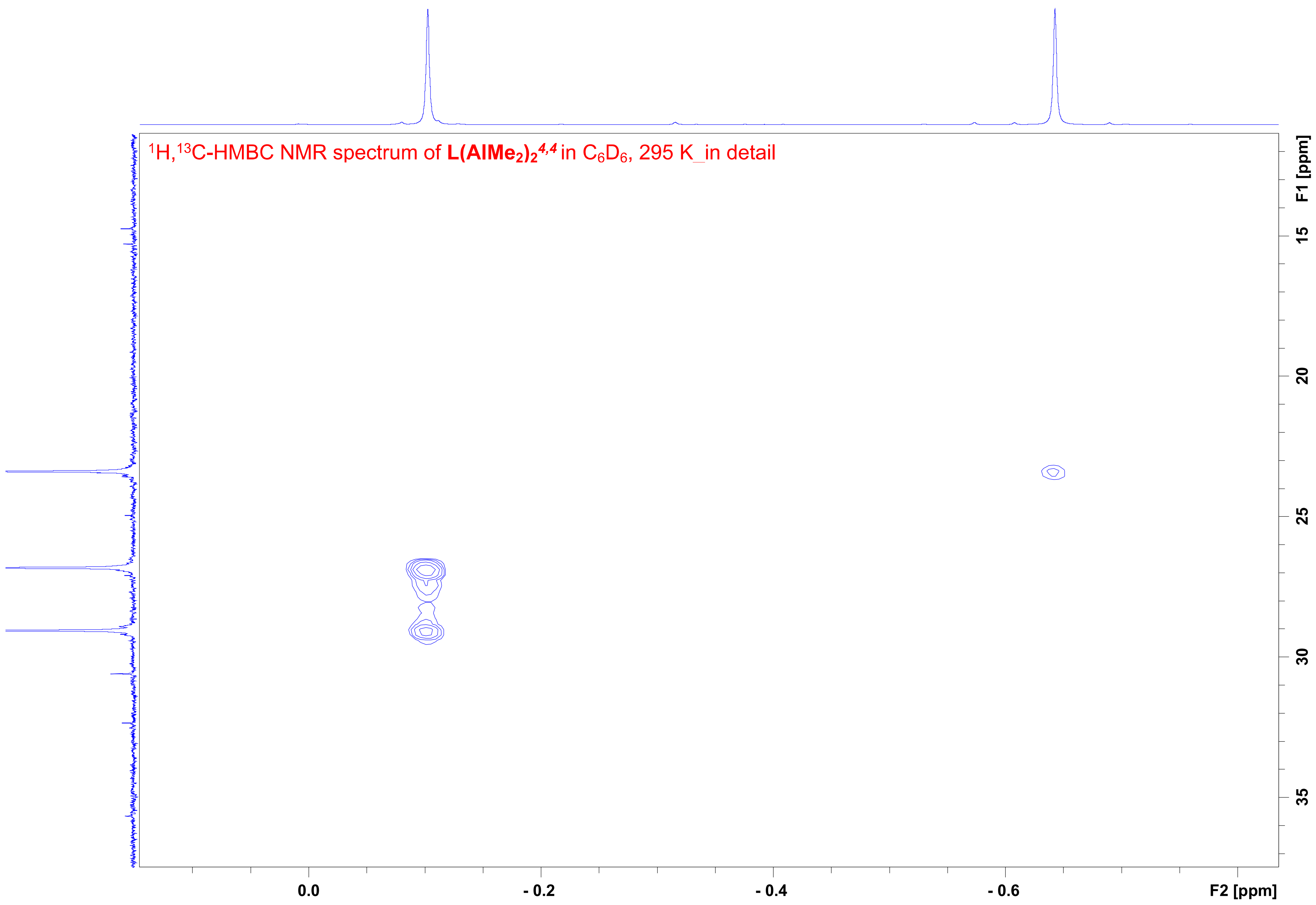

^{1}H,^{13}C-HMBC NMR spectrum of **L(AlMe$_2$)$_2$*^{4,4}*** in C_6D_6, 295 K_in detail
F1 [ppm]
15
20
25
30
35
0.0
- 0.2
- 0.4
- 0.6
F2 [ppm]

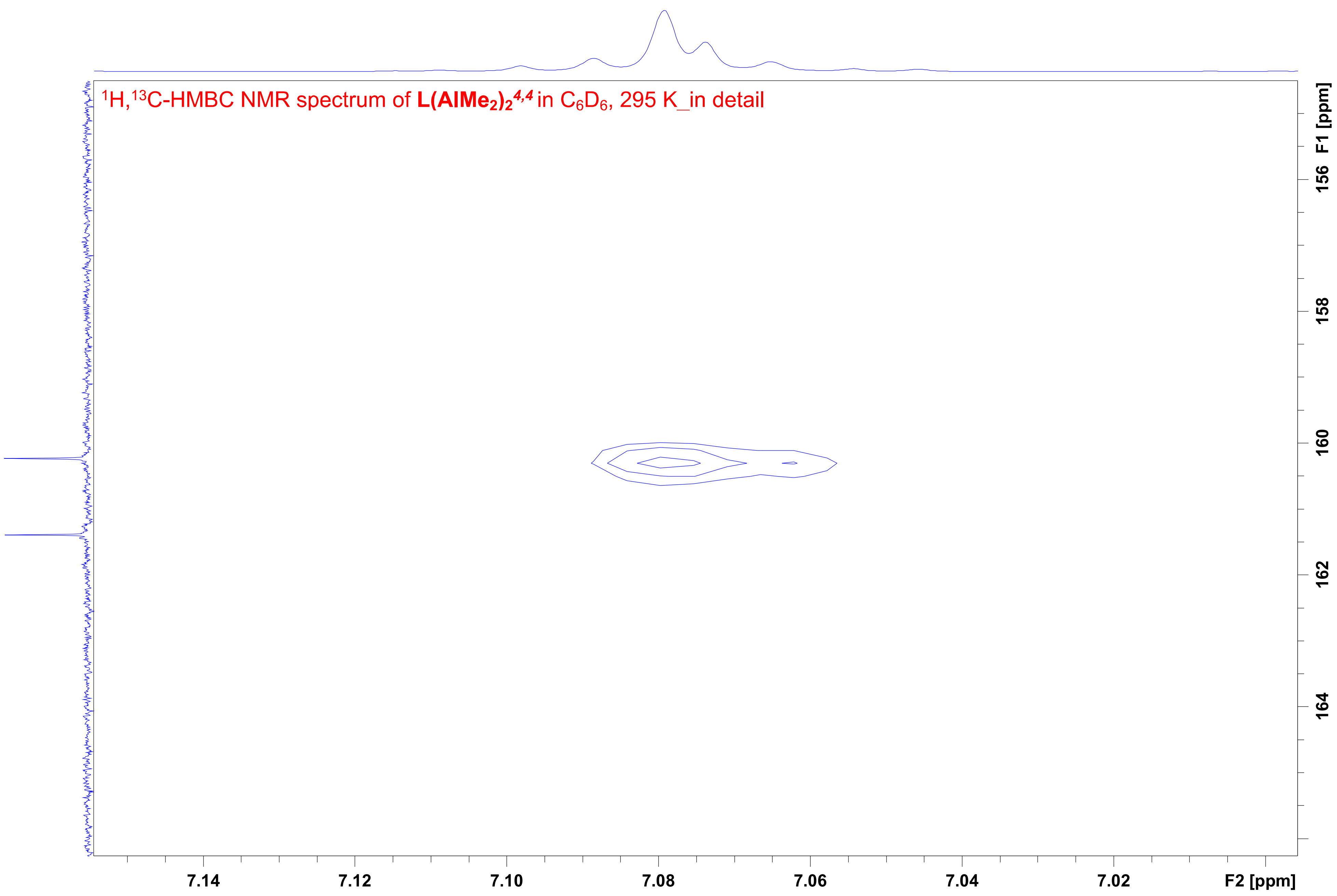

$^{1}H,^{13}C$-HMBC NMR spectrum of **$L(AlMe_2)_2$**[4,4] in C_6D_6, 295 K_in detail
F1 [ppm]
156
158
160
162
164
7.14
7.12
7.10
7.08
7.06
7.04
7.02
F2 [ppm]

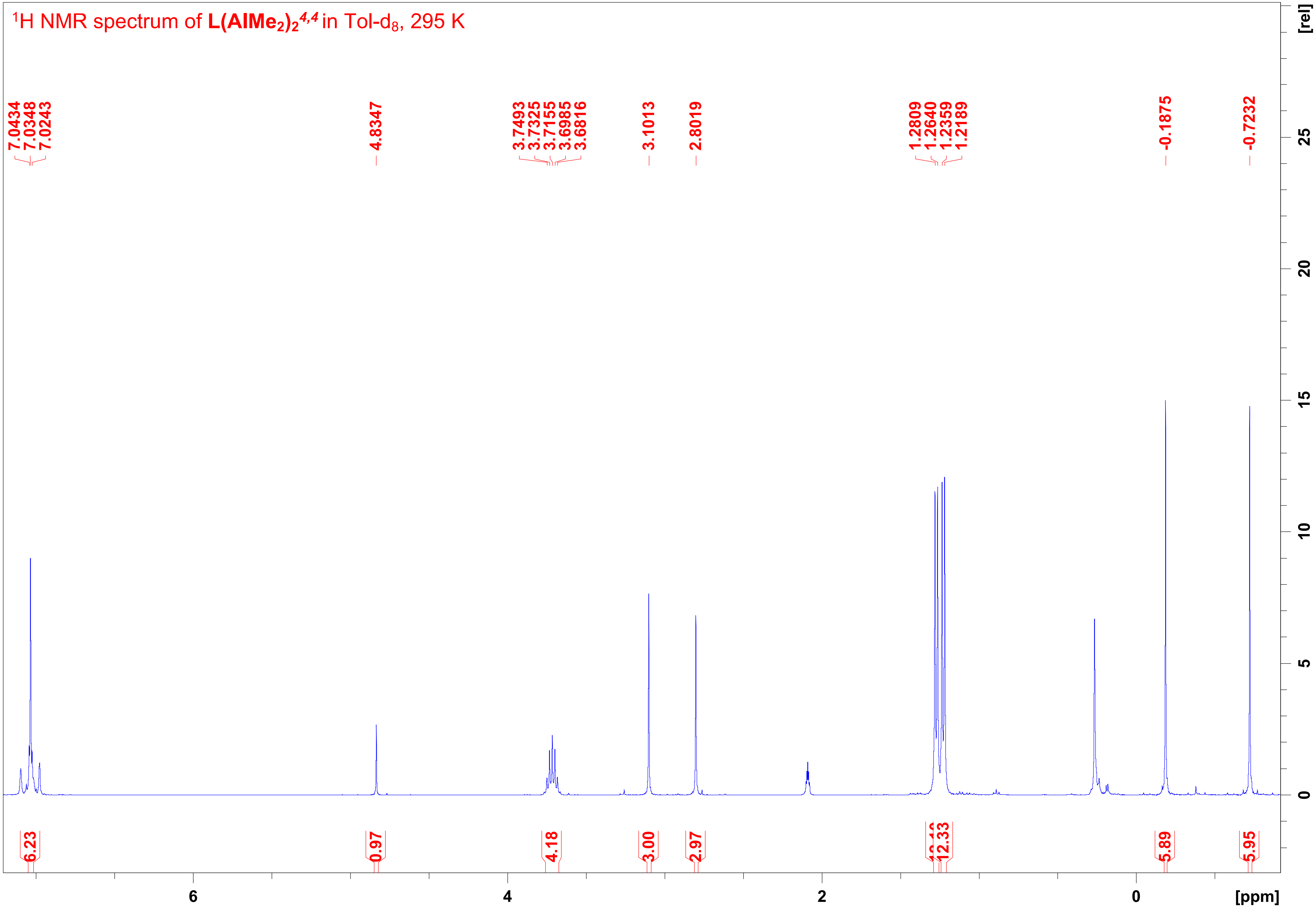

^{1}H NMR spectrum of **$L(AlMe_2)_2$**^{4,4} in Tol-d_8, 295 K

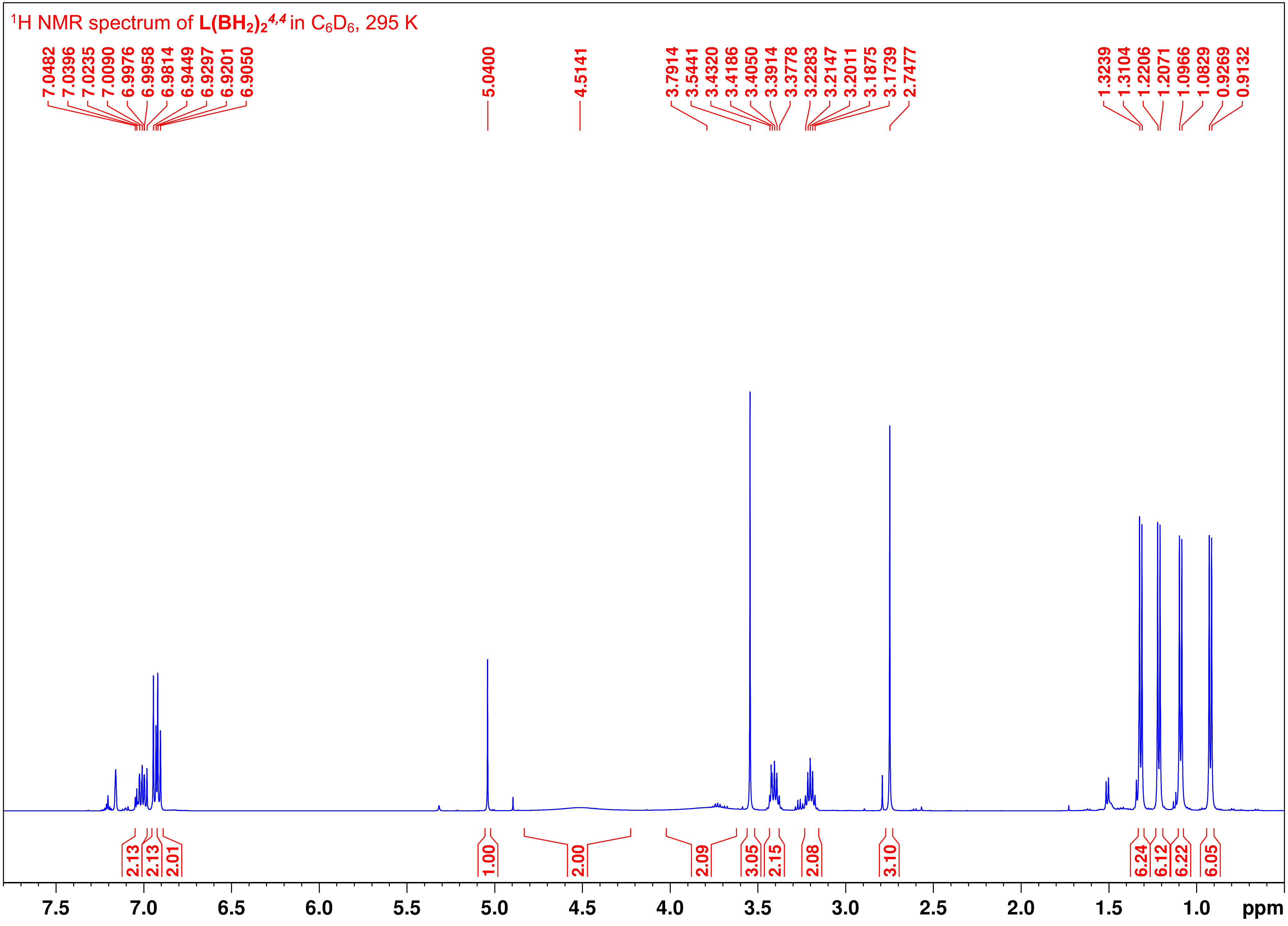

^{1}H NMR spectrum of **$L(BH_2)_2$**^{4,4} in C_6D_6, 295 K
7.0482
7.0396
7.0235
7.0090
6.9976
6.9958
6.9814
6.9449
6.9297
6.9201
6.9050
5.0400
4.5141
3.7914
3.5441
3.4320
3.4186
3.4050
3.3914
3.3778
3.2283
3.2147
3.2011
3.1875
3.1739
2.7477
1.3239
1.3104
1.2206
1.2071
1.0966
1.0829
0.9269
0.9132
2.13
2.13
2.01
1.00
2.00
2.09
3.05
2.15
2.08
3.10
6.24
6.12
6.22
6.05
7.5
7.0
6.5
6.0
5.5
5.0
4.5
4.0
3.5
3.0
2.5
2.0
1.5
1.0
ppm

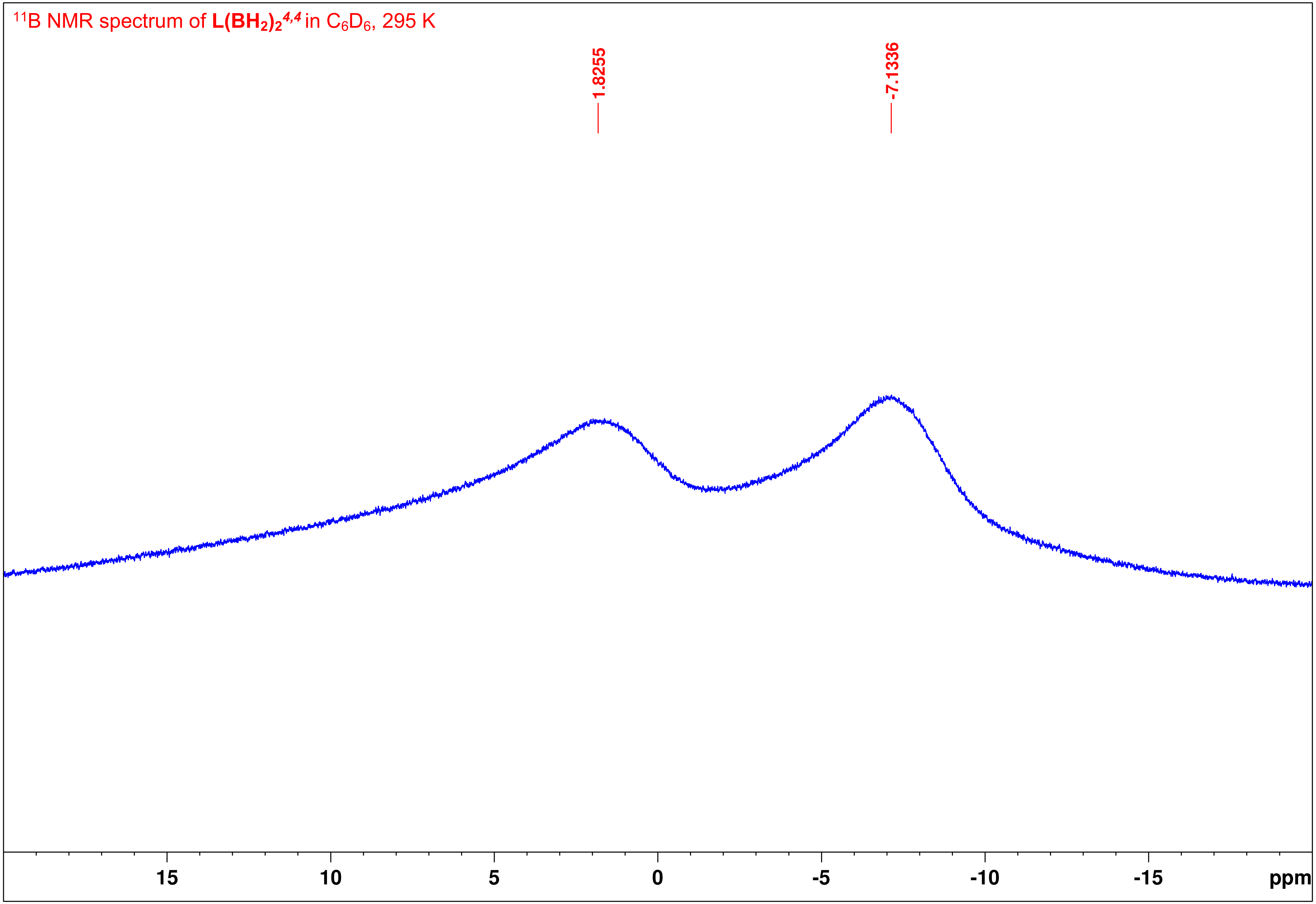

^{11}B NMR spectrum of **$L(BH_2)_2$**^{4,4} in C_6D_6, 295 K
1.8255
-7.1336
15
10
5
0
-5
-10
-15
ppm

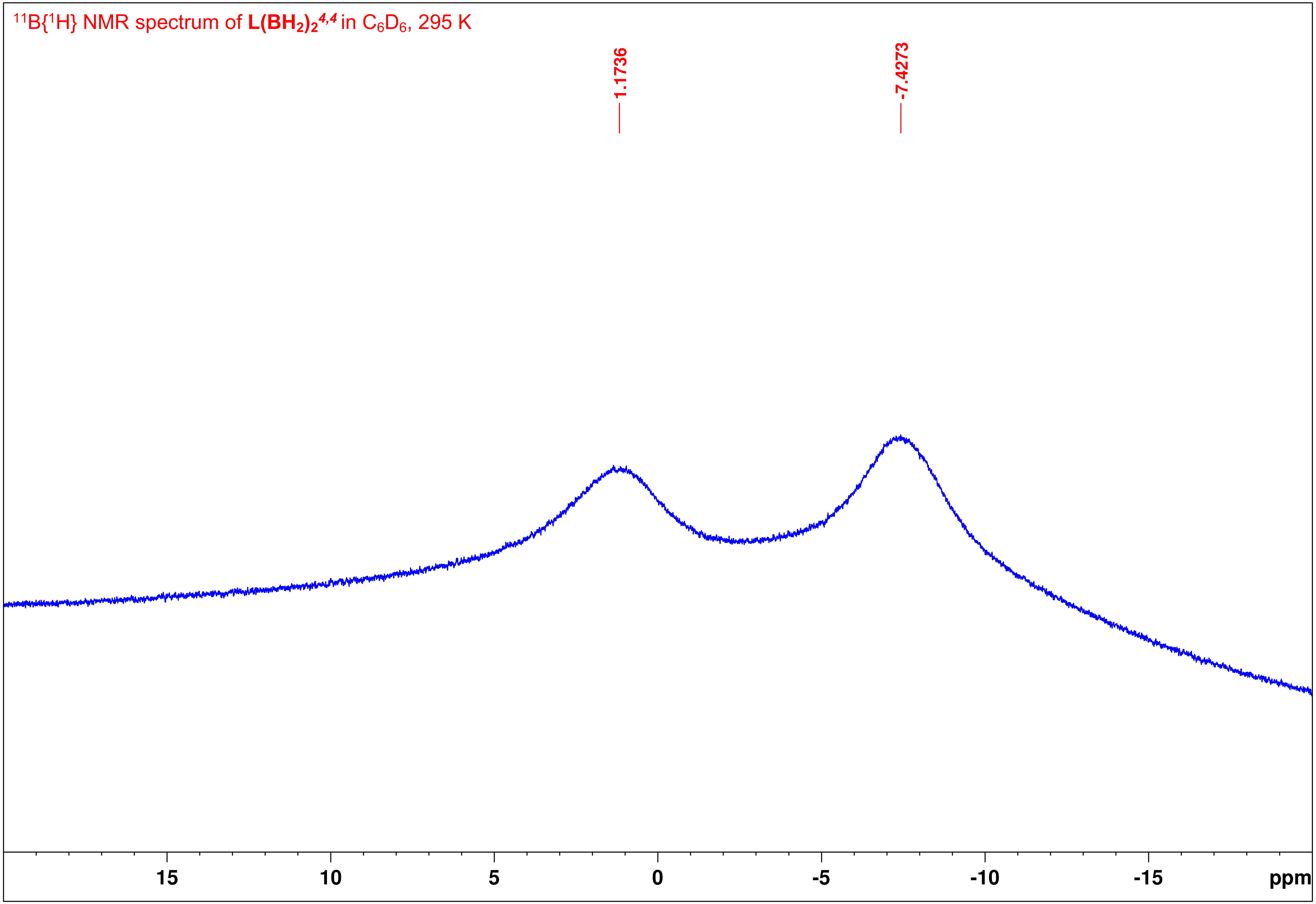
$^{11}B\{^{1}H\}$ NMR spectrum of $\mathbf{L(BH_2)_2}^{\mathbf{\mathit{4,4}}}$ in C_6D_6, 295 K
1.1736
-7.4273
15
10
5
0
-5
-10
-15
ppm

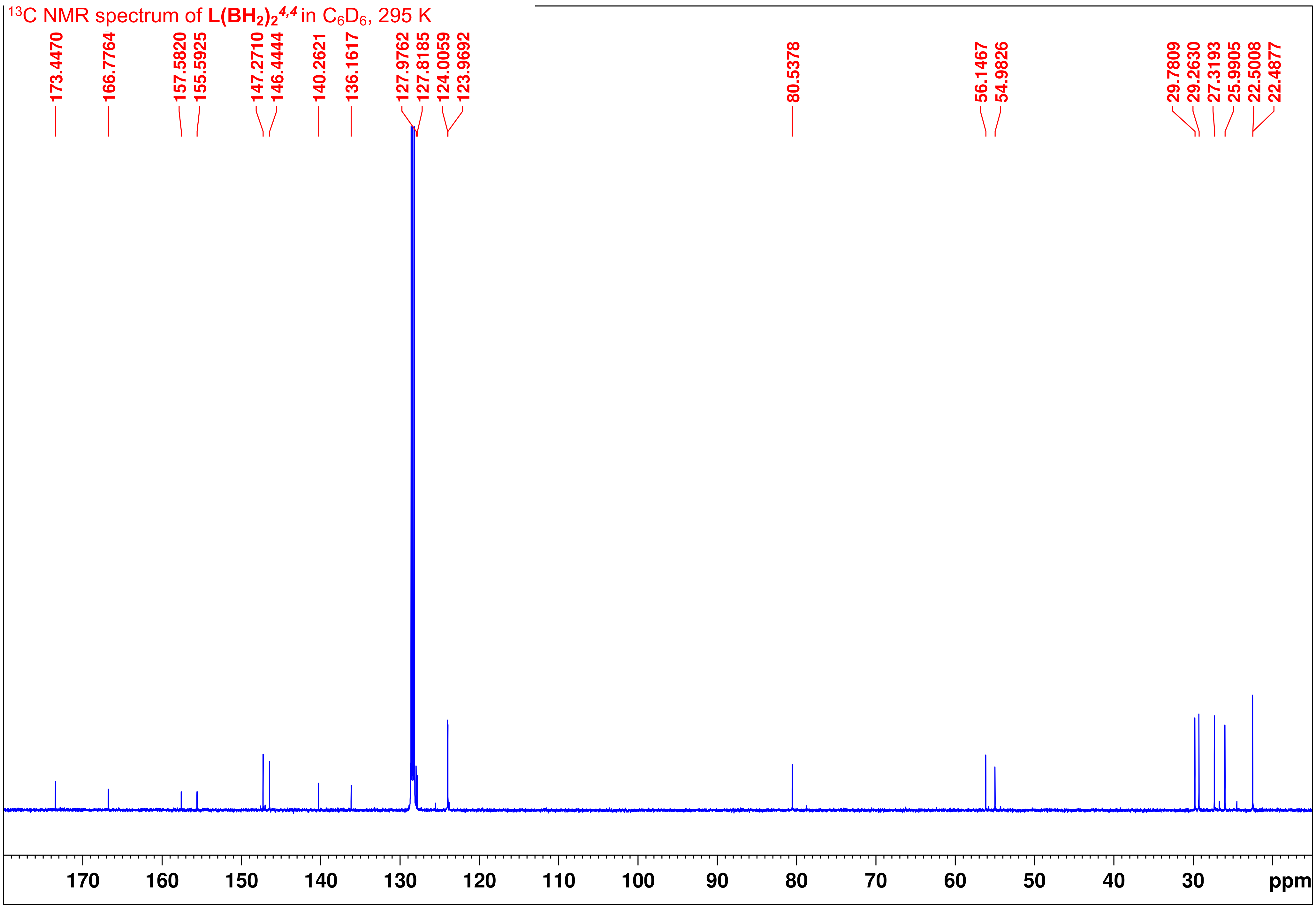
^{13}C NMR spectrum of **L(BH$_2$)$_2$**^{4,4} in C_6D_6, 295 K
173.4470
166.7764
157.5820
155.5925
147.2710
146.4444
140.2621
136.1617
127.9762
127.8185
124.0059
123.9692
80.5378
56.1467
54.9826
29.7809
29.2630
27.3193
25.9905
22.5008
22.4877
170
160
150
140
130
120
110
100
90
80
70
60
50
40
30
ppm

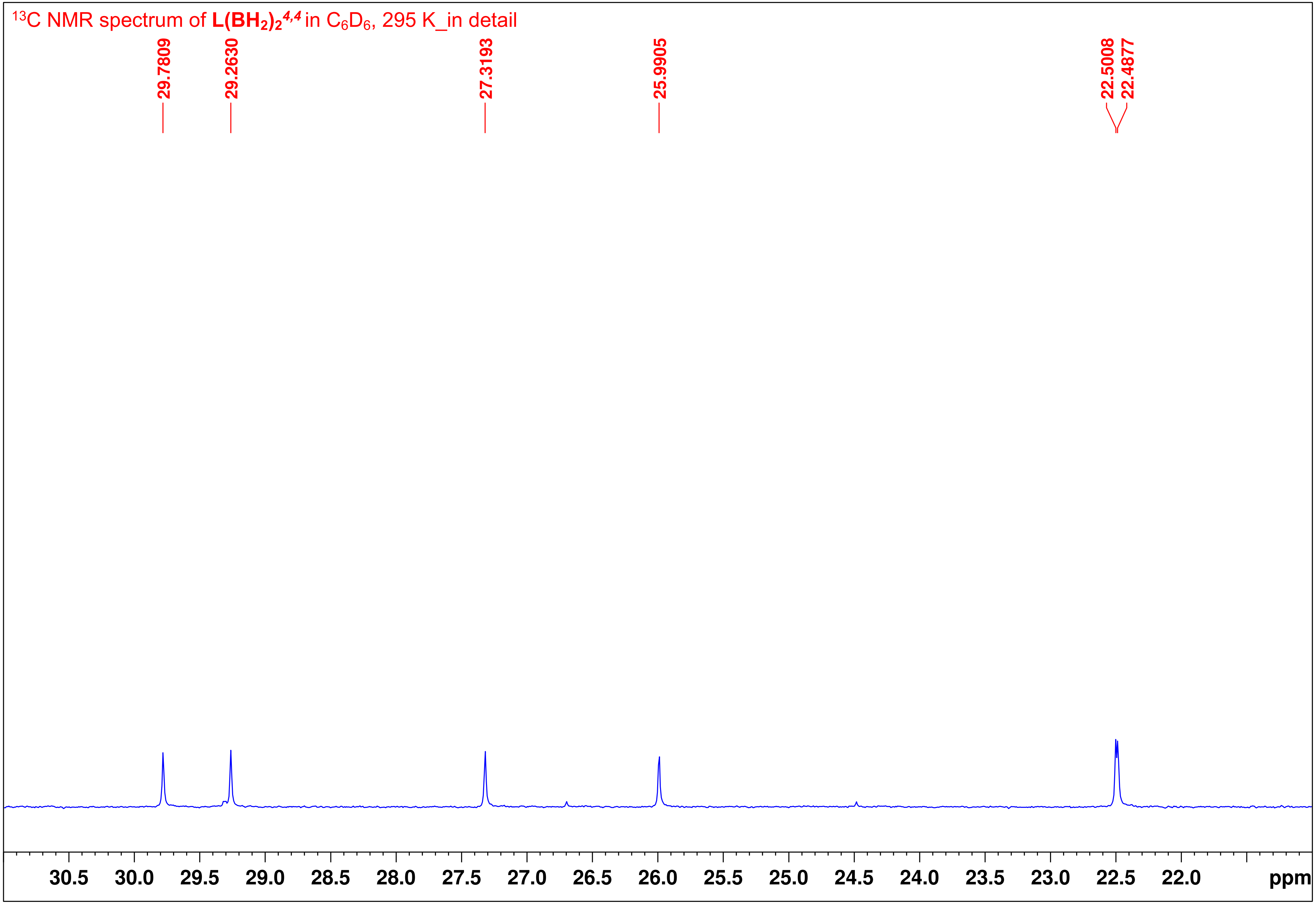

^{13}C NMR spectrum of **$L(BH_2)_2$**^{4,4} in C_6D_6, 295 K_in detail
29.7809
29.2630
27.3193
25.9905
22.5008
22.4877
30.5
30.0
29.5
29.0
28.5
28.0
27.5
27.0
26.5
26.0
25.5
25.0
24.5
24.0
23.5
23.0
22.5
22.0
ppm

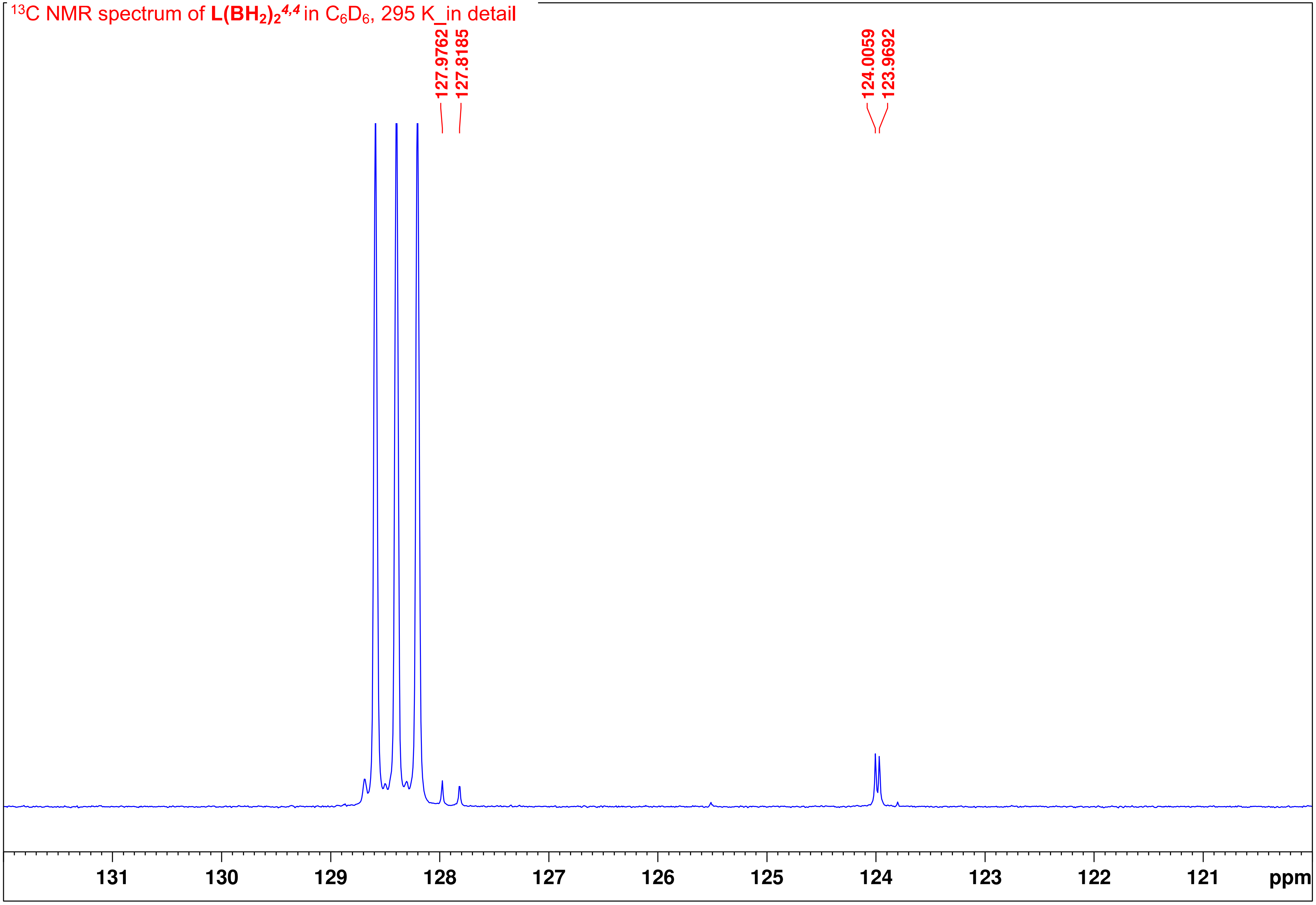

^{13}C NMR spectrum of **L(BH$_2$)$_2$**^{4,4} in C_6D_6, 295 K_in detail
127.9762
127.8185
124.0059
123.9692
131
130
129
128
127
126
125
124
123
122
121
ppm

^{13}C APT NMR spectrum of **$L(BH_2)_2$**^{4,4} in C_6D_6, 295 K

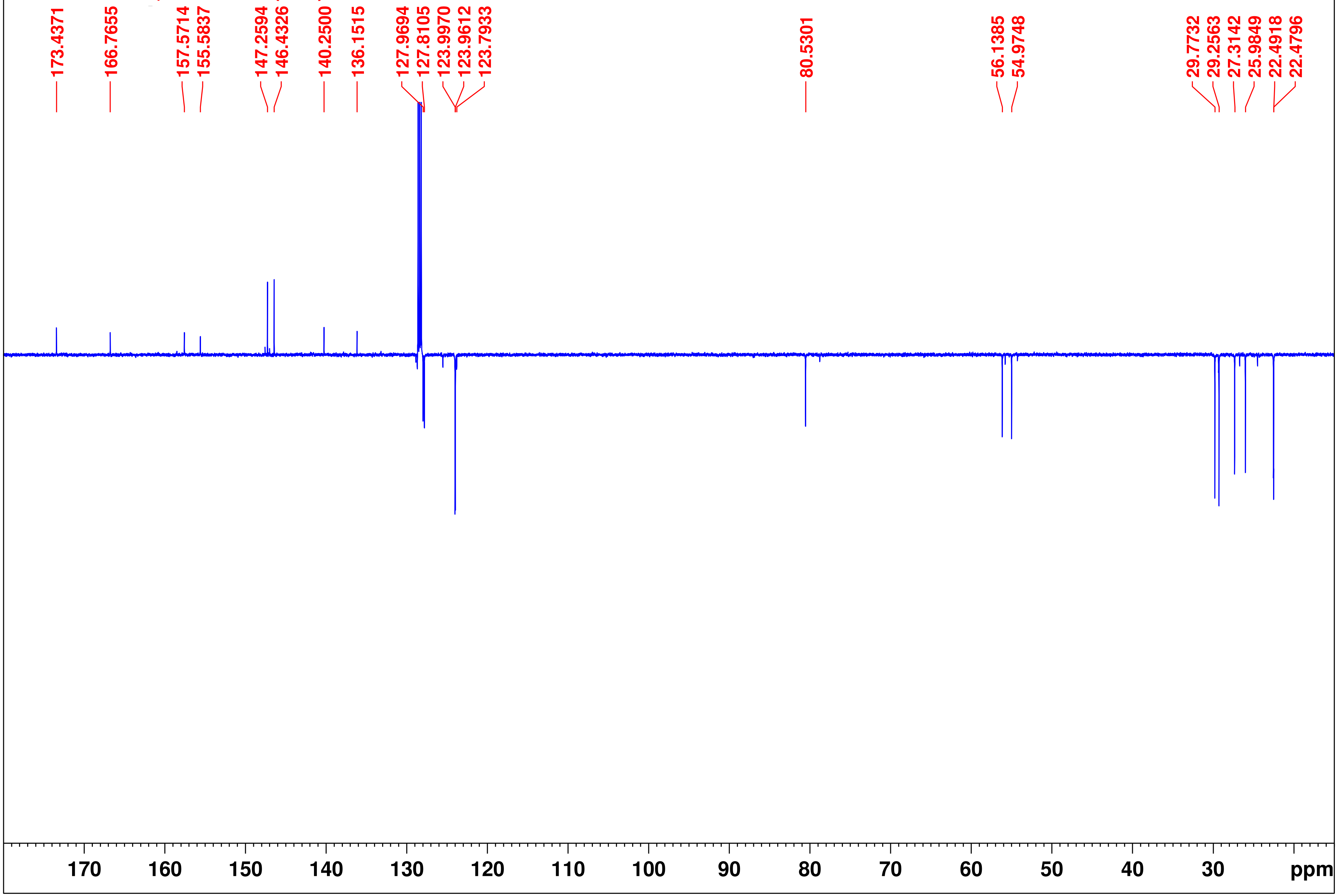

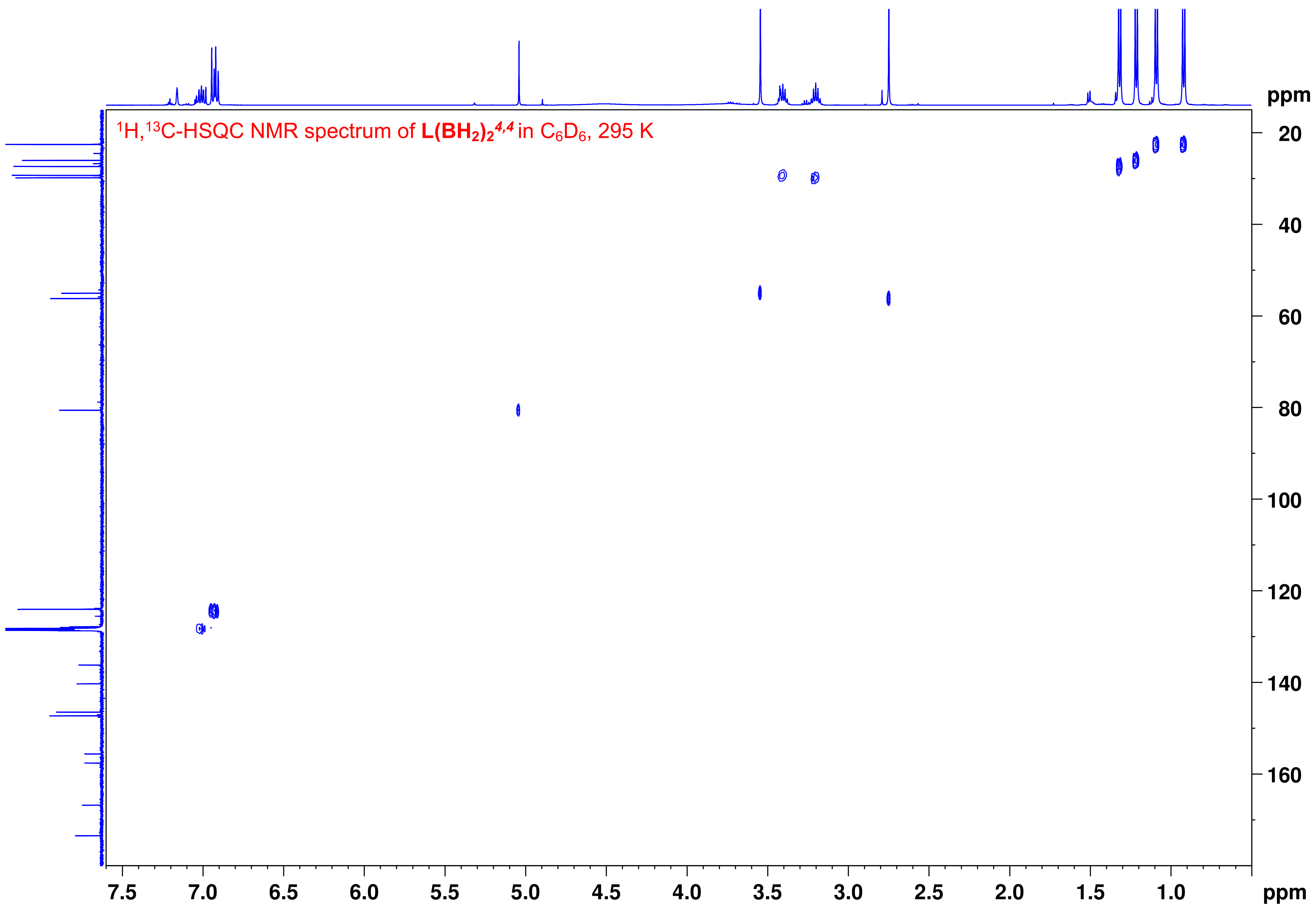
$^1H,^{13}C$-HSQC NMR spectrum of $\mathbf{L(BH_2)_2}^{4,4}$ in C_6D_6, 295 K
ppm
20
40
60
80
100
120
140
160
7.5
7.0
6.5
6.0
5.5
5.0
4.5
4.0
3.5
3.0
2.5
2.0
1.5
1.0
ppm

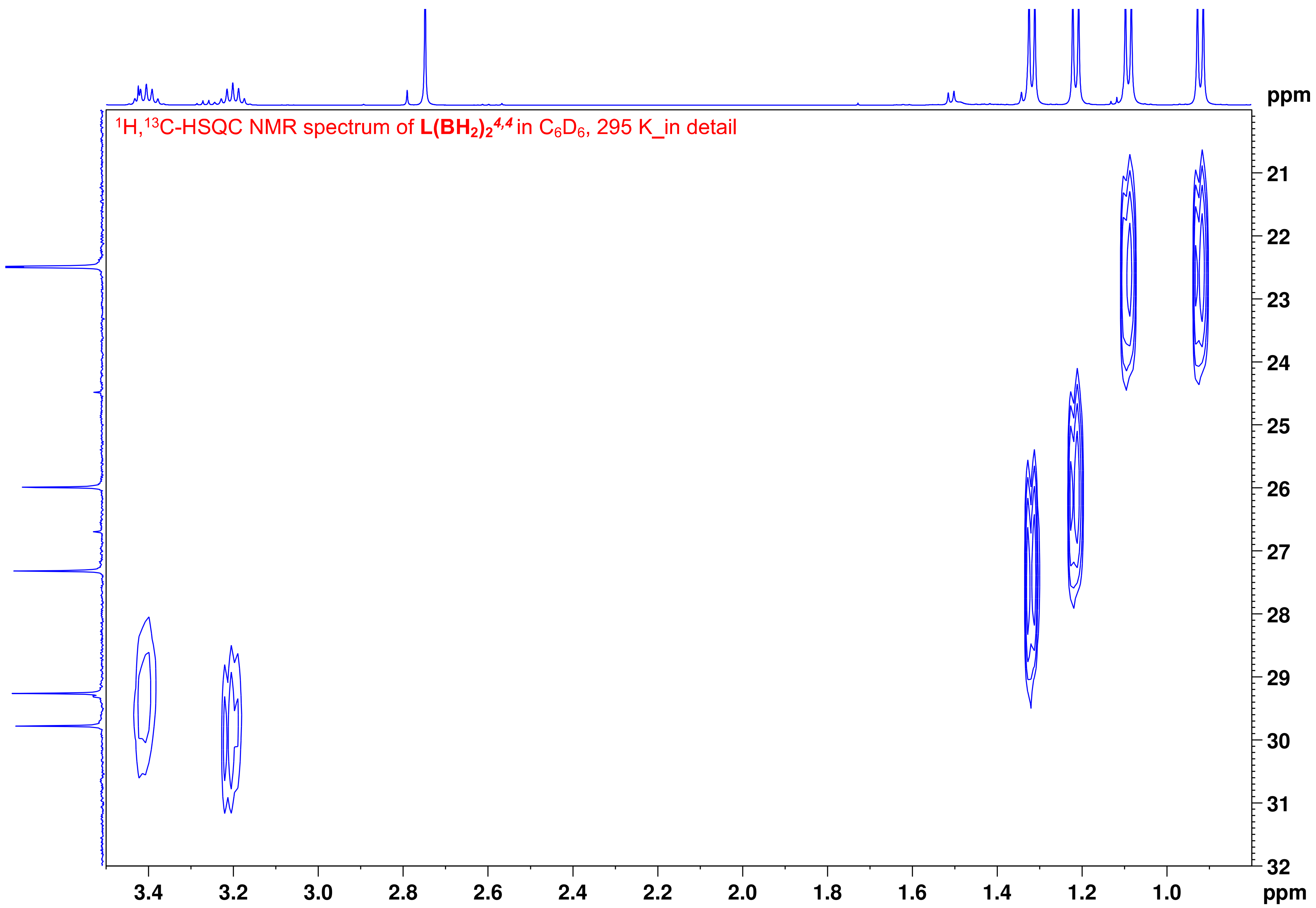
$^{1}H,^{13}C$-HSQC NMR spectrum of **$L(BH_2)_2$**^{4,4} in C_6D_6, 295 K_in detail
ppm
3.4
3.2
3.0
2.8
2.6
2.4
2.2
2.0
1.8
1.6
1.4
1.2
1.0
ppm
21
22
23
24
25
26
27
28
29
30
31
32

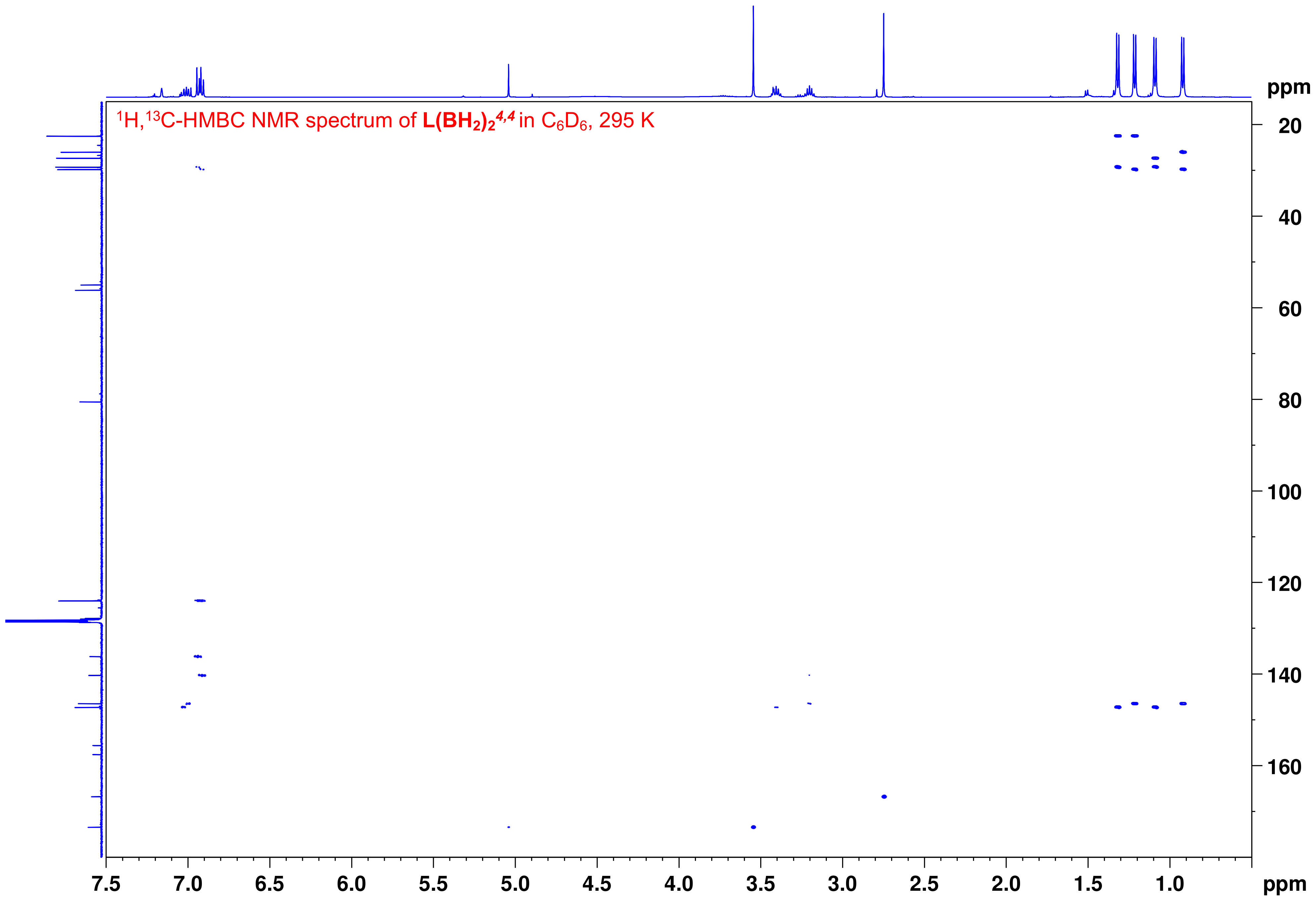

$^{1}H,^{13}C$-HMBC NMR spectrum of **$L(BH_2)_2$*4,4*** in C_6D_6, 295 K

^{1}H NMR spectrum of **L(AlMe$_2$)$_2$^{6,4}** in C_6D_6, 295 K

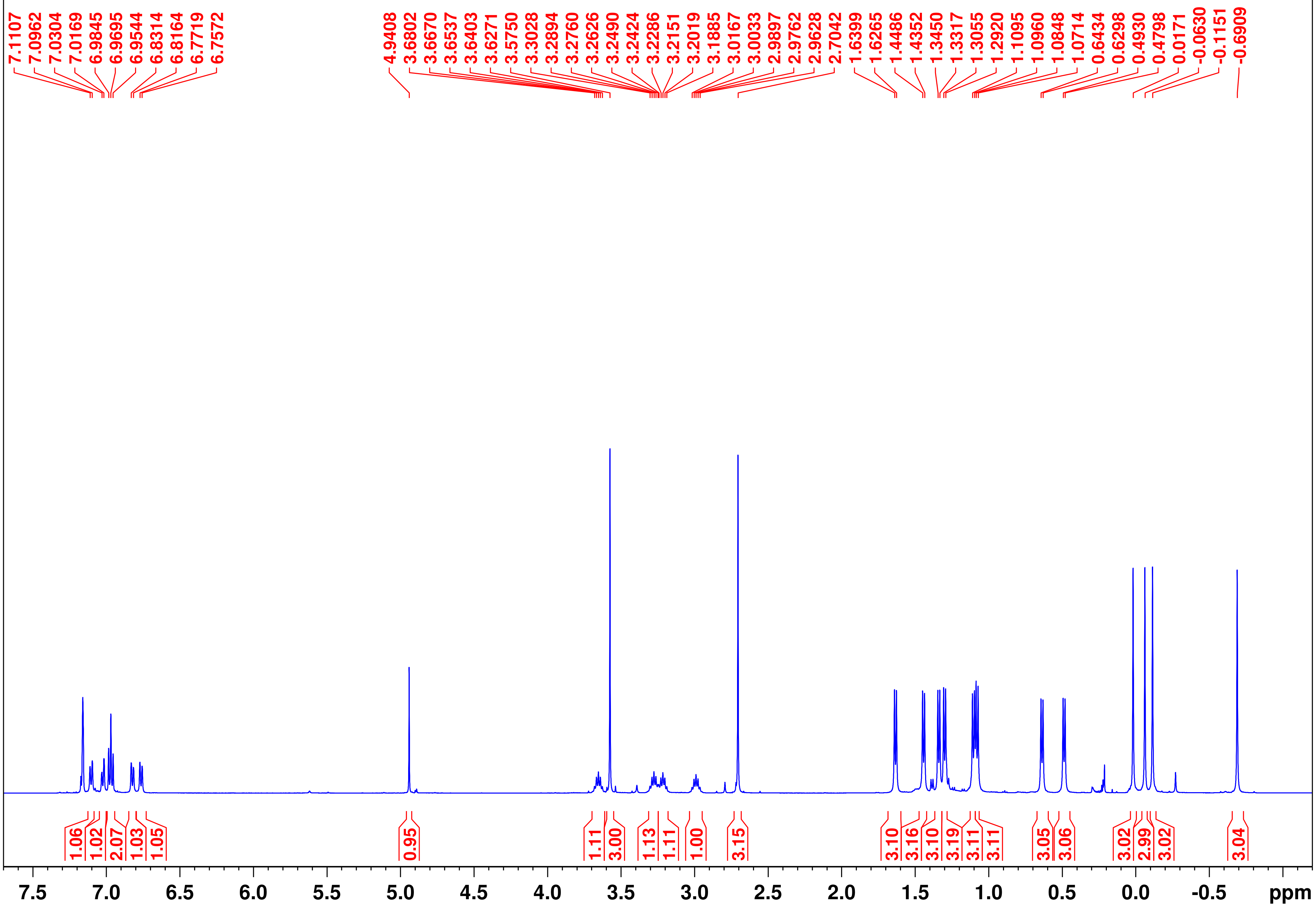

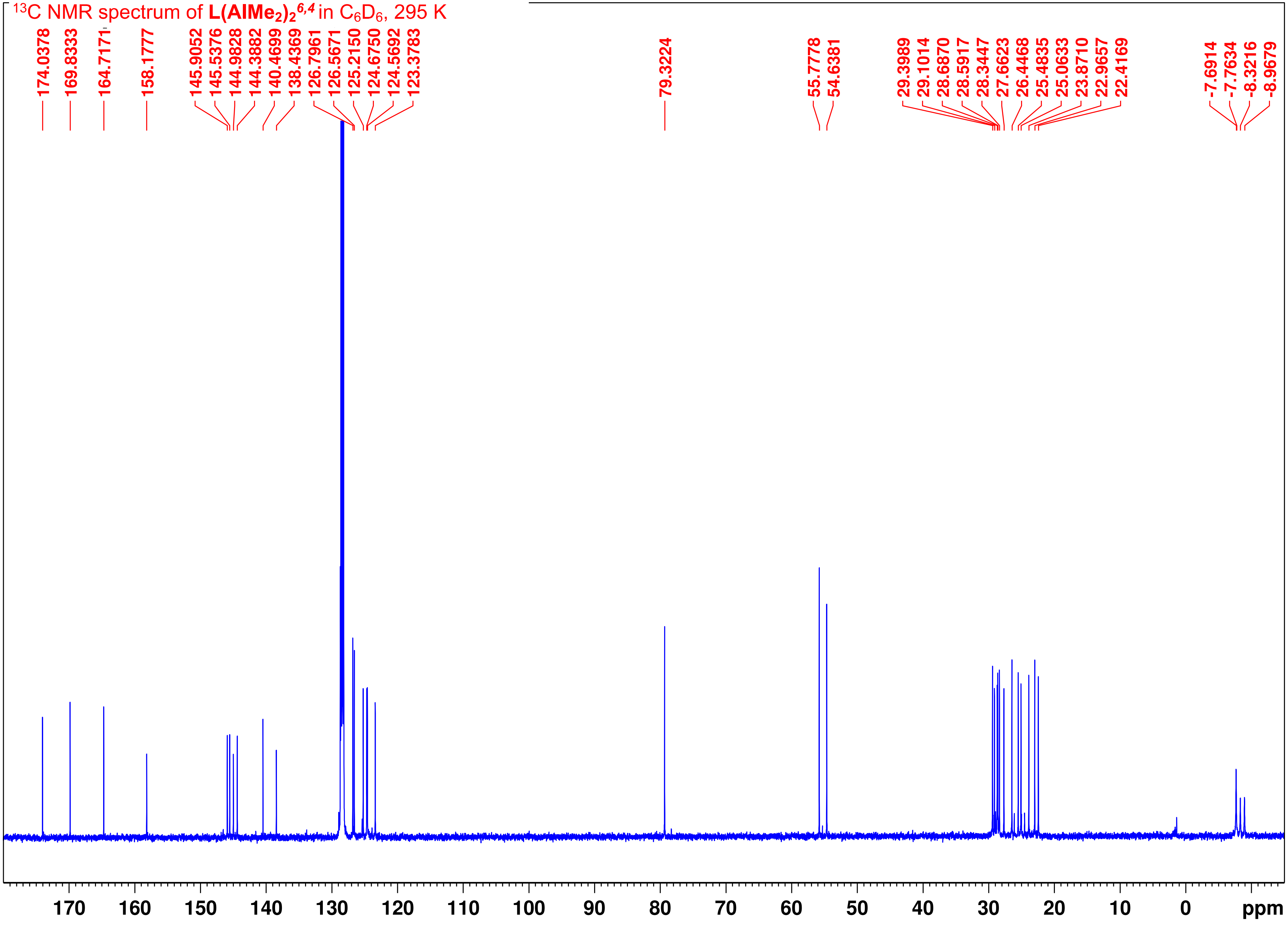

^{13}C NMR spectrum of $\mathbf{L(AlMe_2)_2}^{6,4}$ in C_6D_6, 295 K
174.0378
169.8333
164.7171
158.1777
145.9052
145.5376
144.9828
144.3882
140.4699
138.4369
126.7961
126.5671
125.2150
124.6750
124.5692
123.3783
79.3224
55.7778
54.6381
29.3989
29.1014
28.6870
28.5917
28.3447
27.6623
26.4468
25.4835
25.0633
23.8710
22.9657
22.4169
-7.6914
-7.7634
-8.3216
-8.9679
170
160
150
140
130
120
110
100
90
80
70
60
50
40
30
20
10
0
ppm

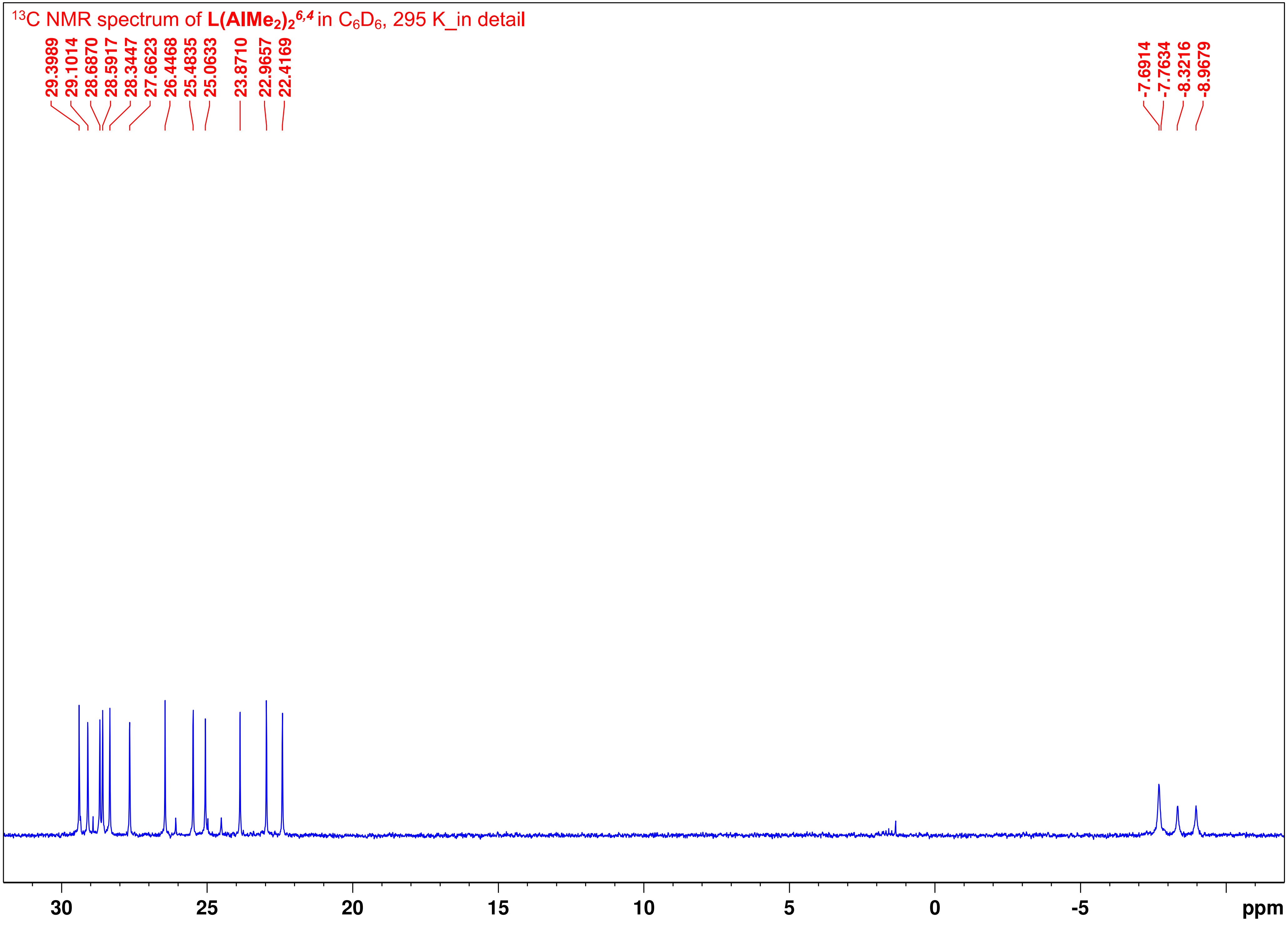

13C NMR spectrum of L(AlMe2)2 6,4 in C6D6, 295 K_in detail
29.3989
29.1014
28.6870
28.5917
28.3447
27.6623
26.4468
25.4835
25.0633
23.8710
22.9657
22.4169
-7.6914
-7.7634
-8.3216
-8.9679
30
25
20
15
10
5
0
-5
ppm

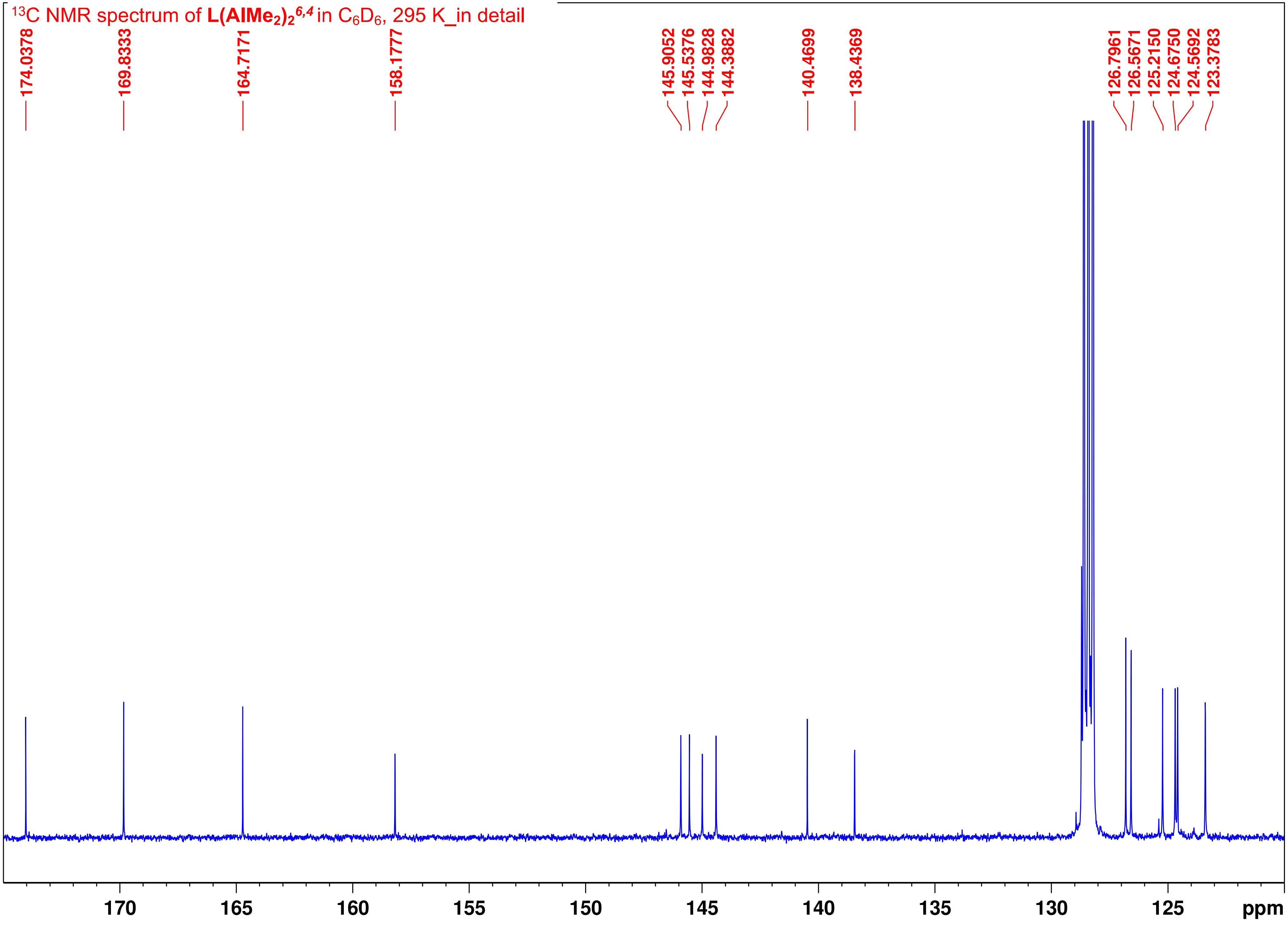

13C NMR spectrum of L(AlMe2)2 6,4 in C6D6, 295 K_in detail
174.0378
169.8333
164.7171
158.1777
145.9052
145.5376
144.9828
144.3882
140.4699
138.4369
126.7961
126.5671
125.2150
124.6750
124.5692
123.3783
170
165
160
155
150
145
140
135
130
125
ppm

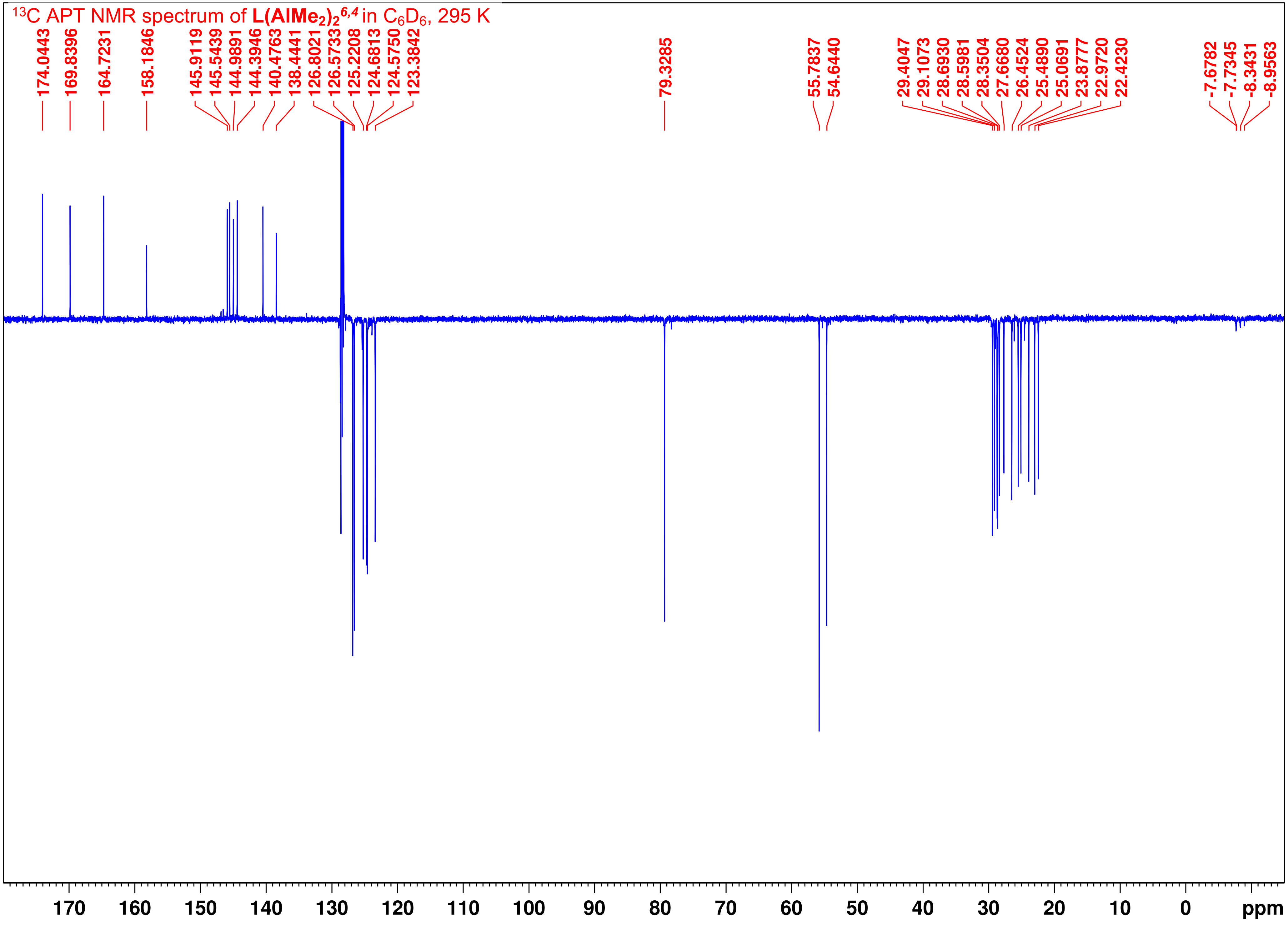

^{13}C APT NMR spectrum of **L(AlMe$_2$)$_2$**^{6,4} in C_6D_6, 295 K
174.0443
169.8396
164.7231
158.1846
145.9119
145.5439
144.9891
144.3946
140.4763
138.4441
126.8021
126.5733
125.2208
124.6813
124.5750
123.3842
79.3285
55.7837
54.6440
29.4047
29.1073
28.6930
28.5981
28.3504
27.6680
26.4524
25.4890
25.0691
23.8777
22.9720
22.4230
-7.6782
-7.7345
-8.3431
-8.9563
170
160
150
140
130
120
110
100
90
80
70
60
50
40
30
20
10
0
ppm

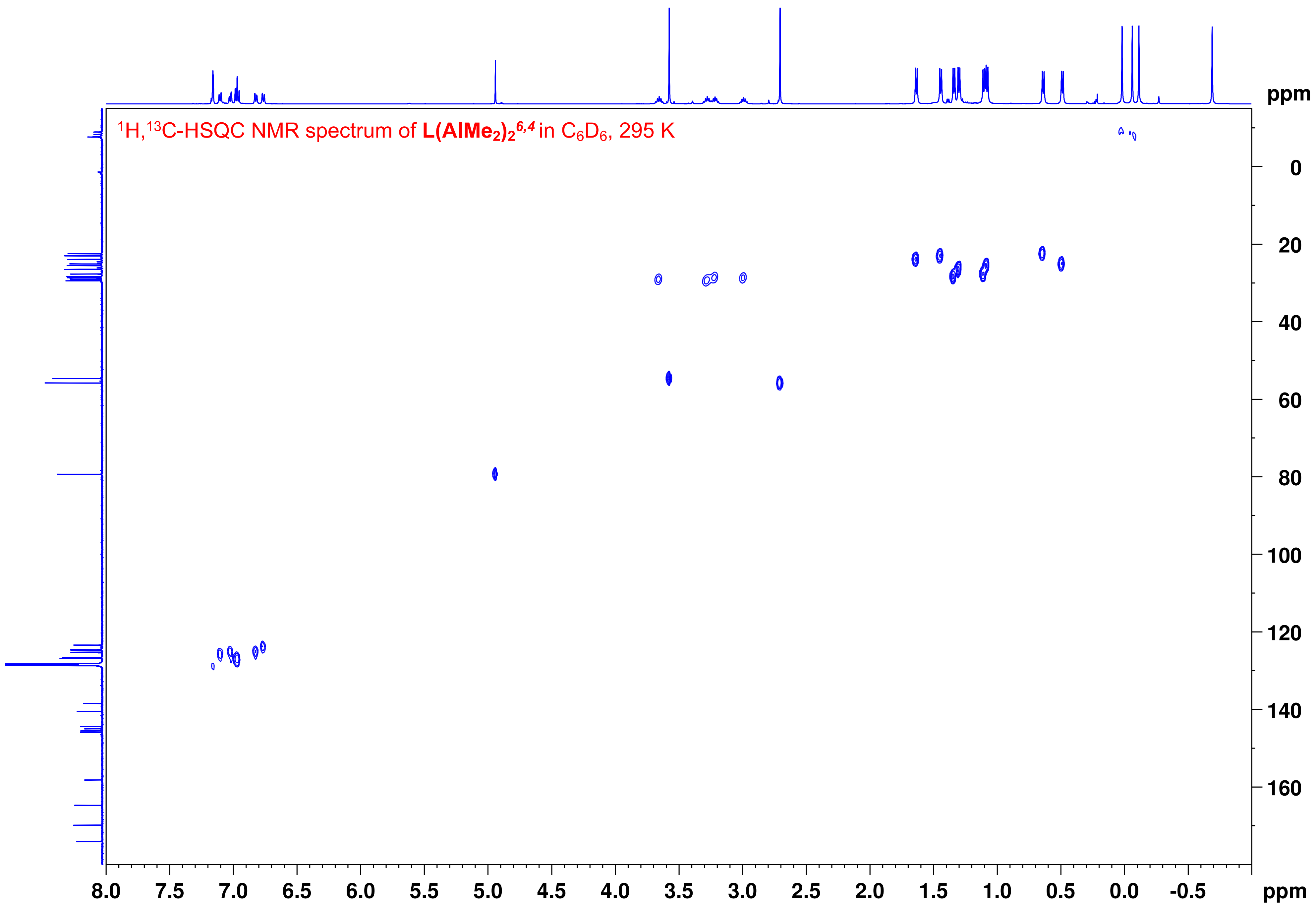
$^1H,^{13}C$-HSQC NMR spectrum of $\mathbf{L(AlMe_2)_2}^{6,4}$ in C_6D_6, 295 K
ppm
0
20
40
60
80
100
120
140
160
8.0
7.5
7.0
6.5
6.0
5.5
5.0
4.5
4.0
3.5
3.0
2.5
2.0
1.5
1.0
0.5
0.0
-0.5
ppm

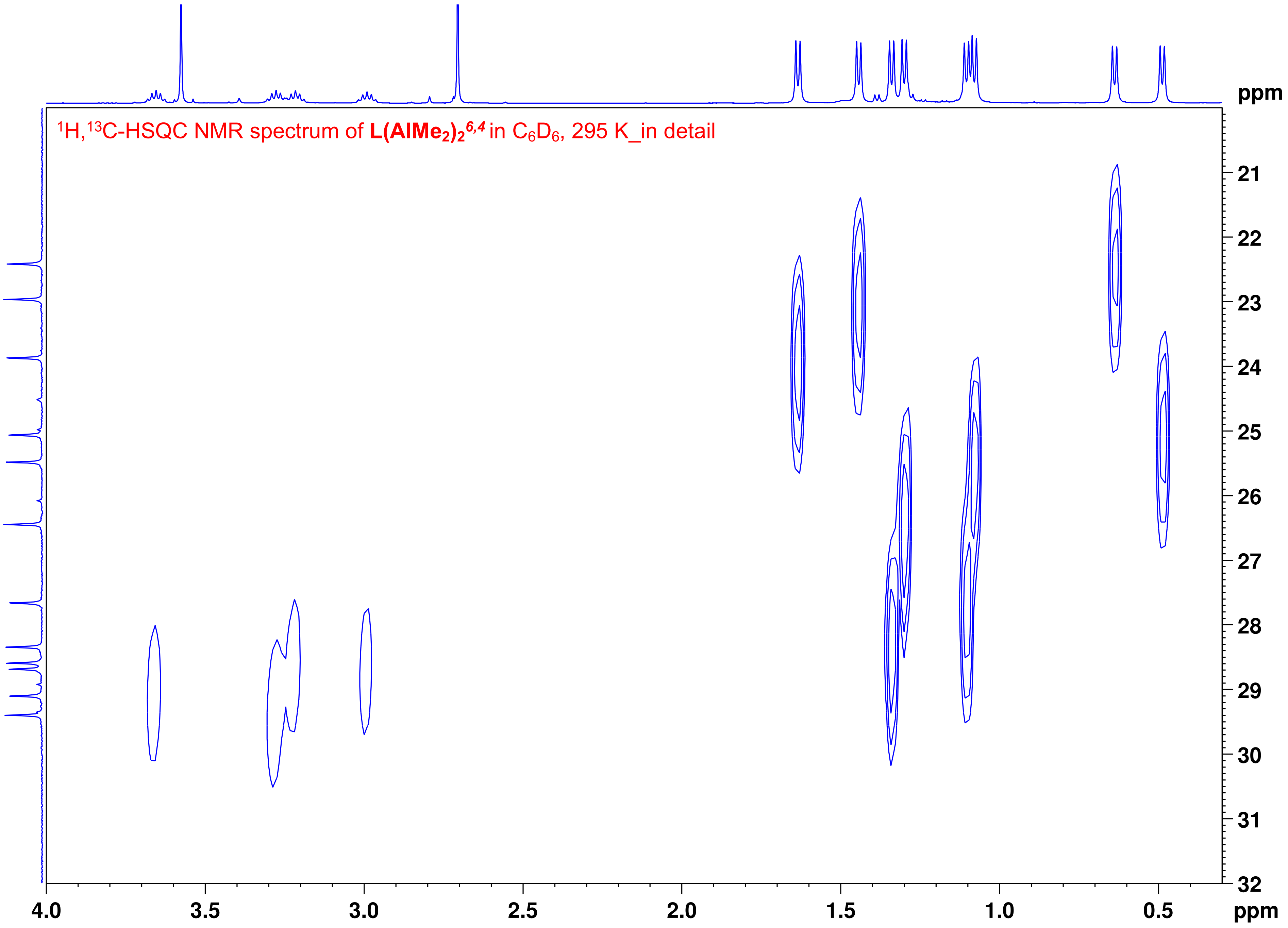

$^{1}H,^{13}C$-HSQC NMR spectrum of **$L(AlMe_2)_2$*^{6,4}*** in C_6D_6, 295 K_in detail
ppm
21
22
23
24
25
26
27
28
29
30
31
32
4.0
3.5
3.0
2.5
2.0
1.5
1.0
0.5
ppm

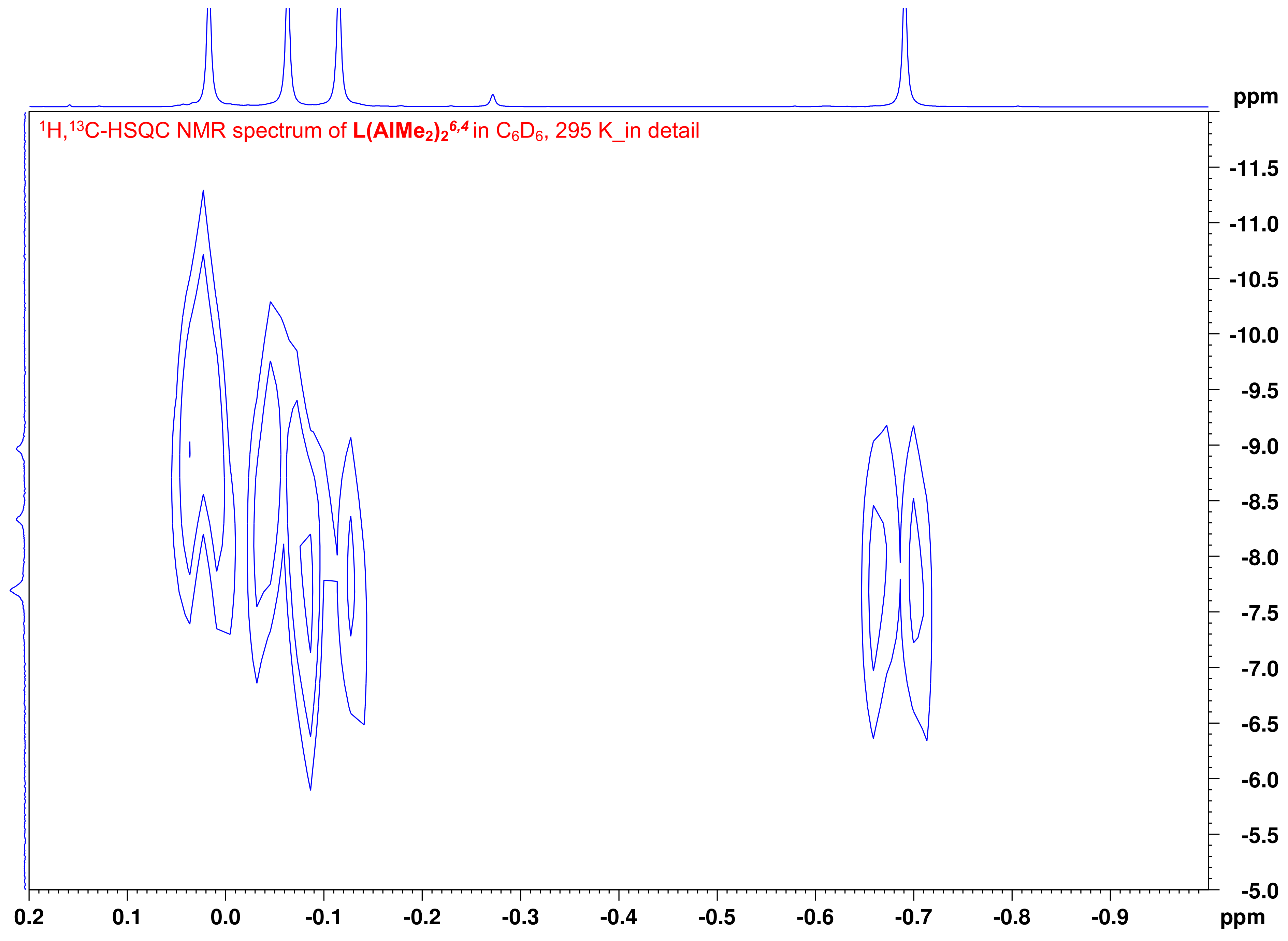

$^{1}H,^{13}C$-HSQC NMR spectrum of $\mathbf{L(AlMe_2)_2}^{6,4}$ in C_6D_6, 295 K_in detail
ppm
-11.5
-11.0
-10.5
-10.0
-9.5
-9.0
-8.5
-8.0
-7.5
-7.0
-6.5
-6.0
-5.5
-5.0
ppm
0.2
0.1
0.0
-0.1
-0.2
-0.3
-0.4
-0.5
-0.6
-0.7
-0.8
-0.9
ppm

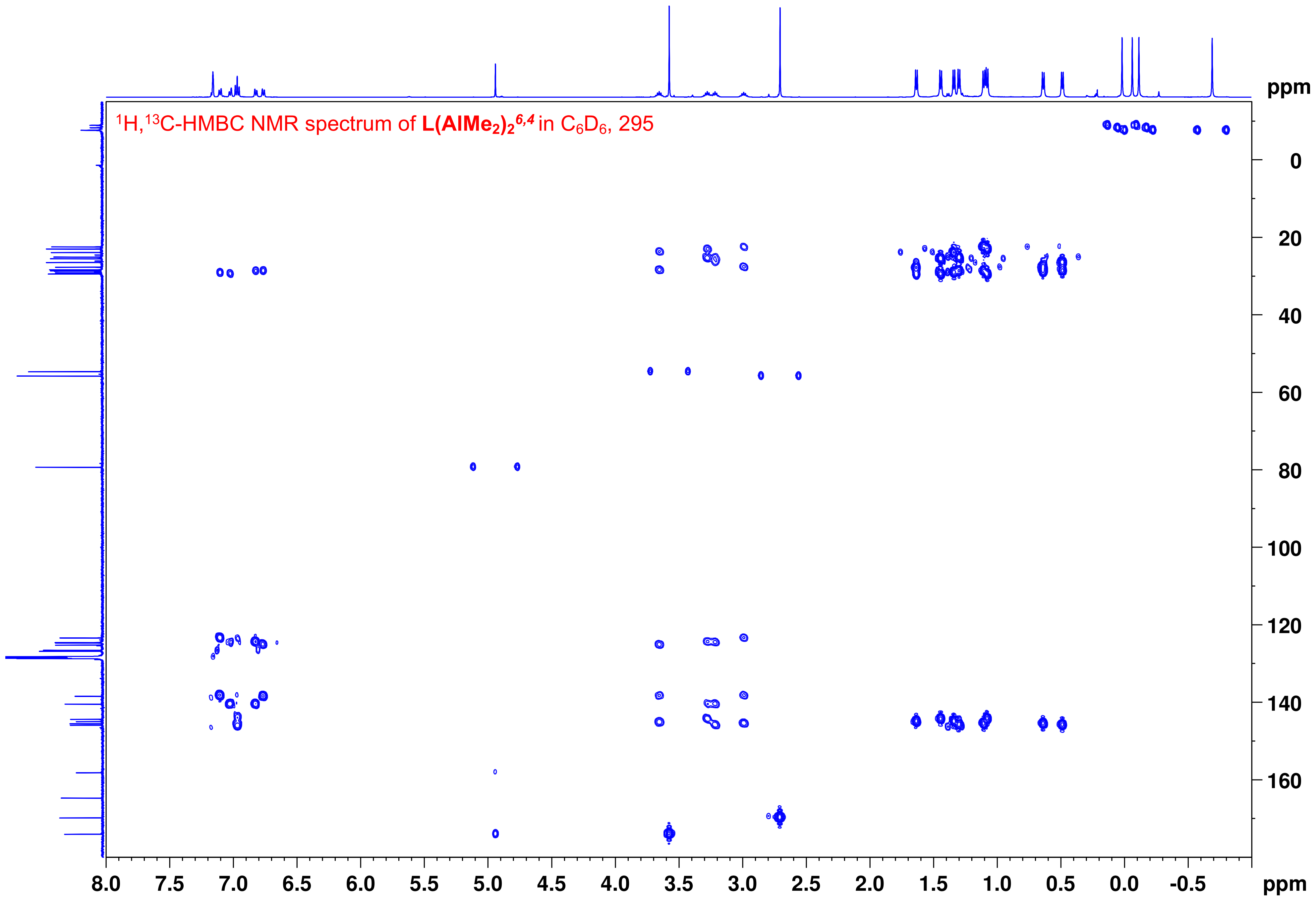

$^1H,^{13}C$-HMBC NMR spectrum of **$L(AlMe_2)_2$**^{6,4} in C_6D_6, 295
ppm
0
20
40
60
80
100
120
140
160
8.0
7.5
7.0
6.5
6.0
5.5
5.0
4.5
4.0
3.5
3.0
2.5
2.0
1.5
1.0
0.5
0.0
-0.5
ppm

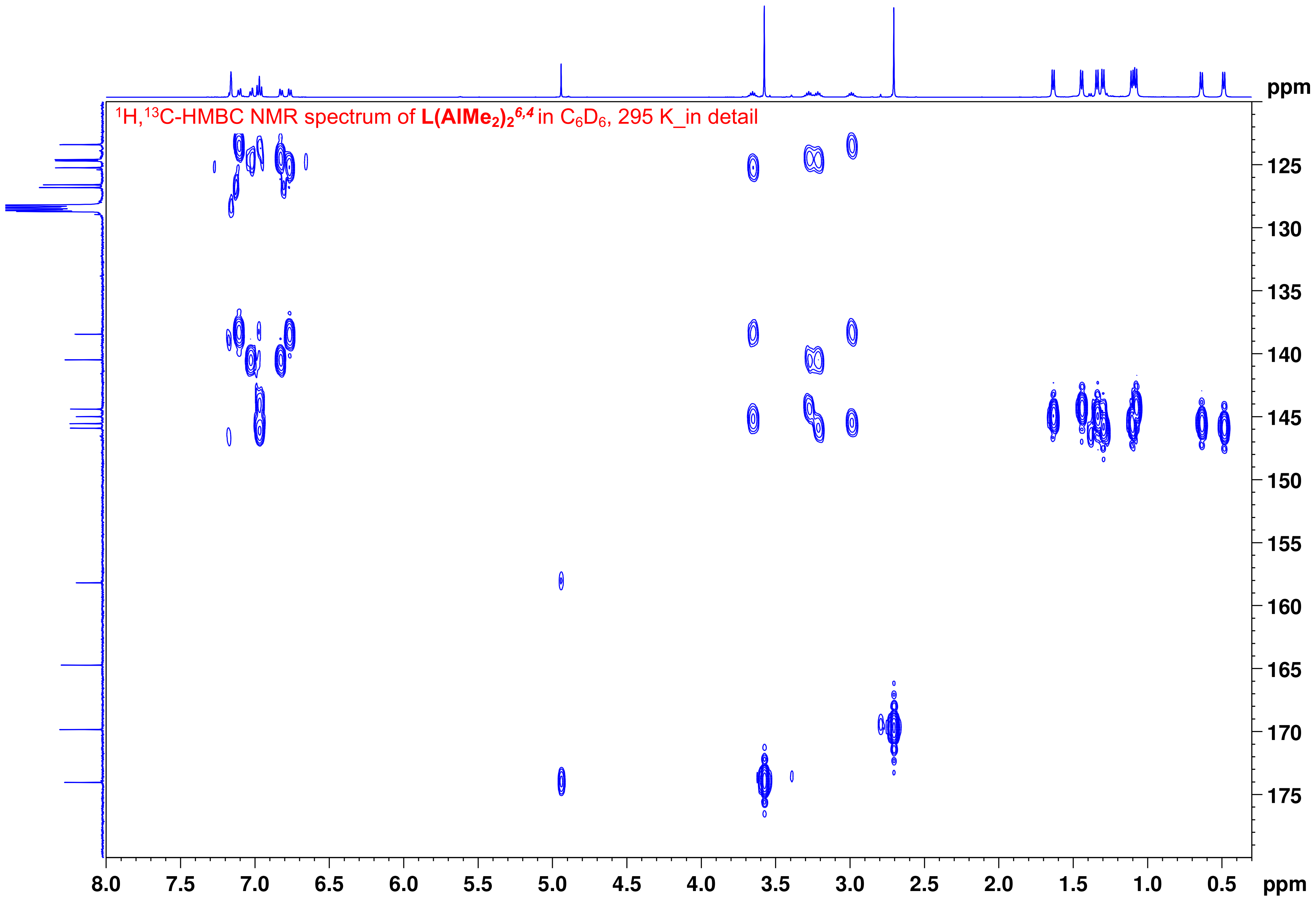

$^{1}H,^{13}C$-HMBC NMR spectrum of **$L(AlMe_2)_2$**^{6,4} in C_6D_6, 295 K_in detail
ppm
125
130
135
140
145
150
155
160
165
170
175
ppm
8.0
7.5
7.0
6.5
6.0
5.5
5.0
4.5
4.0
3.5
3.0
2.5
2.0
1.5
1.0
0.5
ppm

^{1}H NMR spectrum of **L(AlMe$_2$)$_2$**^{6,4} in Tol-d$_8$, 295 K

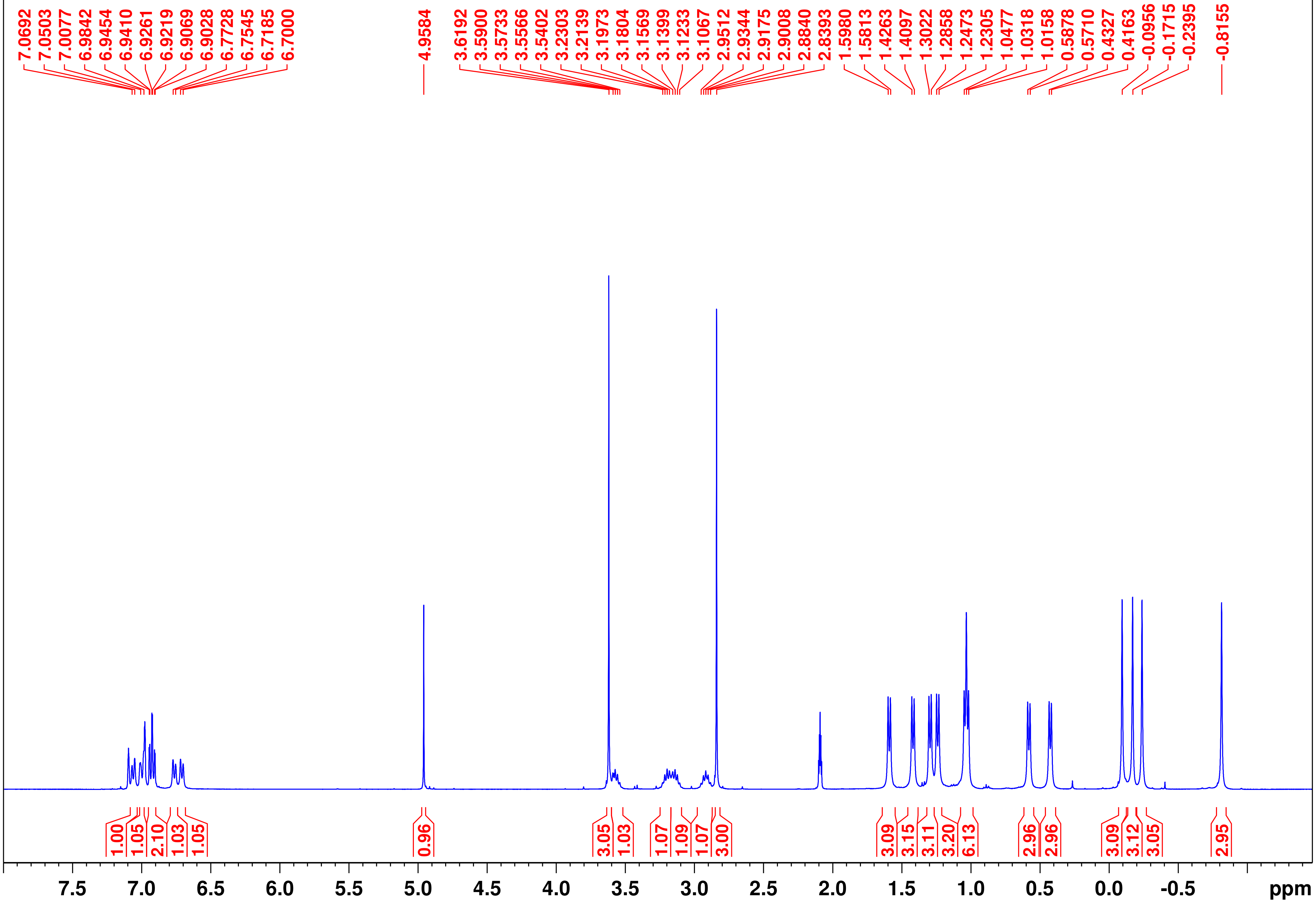

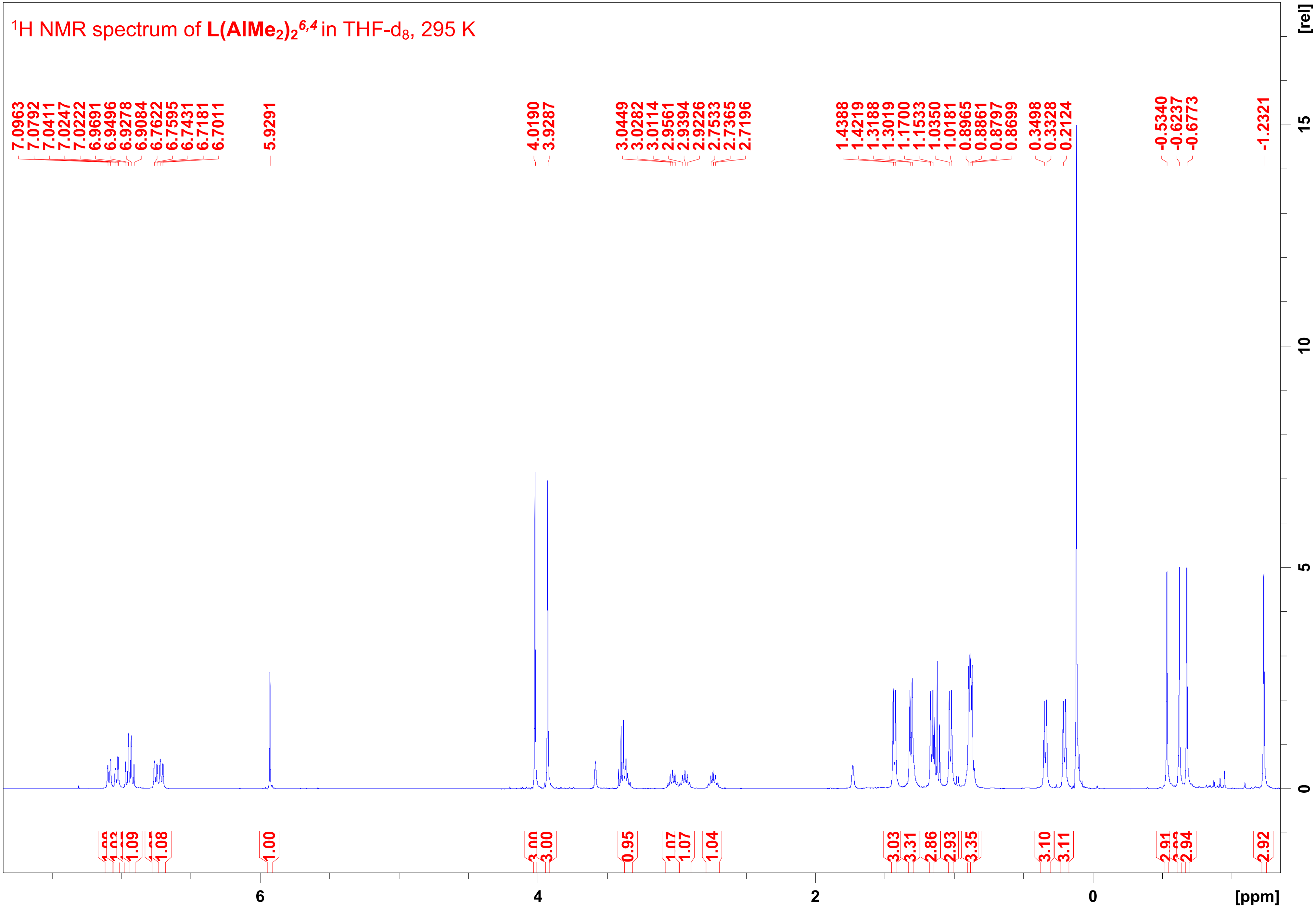

1H NMR spectrum of L(AlMe2)2 6,4 in THF-d8, 295 K
7.0963
7.0792
7.0411
7.0247
7.0222
6.9691
6.9496
6.9278
6.9084
6.7622
6.7595
6.7431
6.7181
6.7011
5.9291
4.0190
3.9287
3.0449
3.0282
3.0114
2.9561
2.9394
2.9226
2.7533
2.7365
2.7196
1.4388
1.4219
1.3188
1.3019
1.1700
1.1533
1.0350
1.0181
0.8965
0.8861
0.8797
0.8699
0.3498
0.3328
0.2124
-0.5340
-0.6237
-0.6773
-1.2321
1.09
1.08
1.00
3.00
0.95
1.07
1.07
1.04
3.03
3.31
2.86
2.93
3.35
3.10
3.11
2.91
2.94
2.92
[rel]
[ppm]

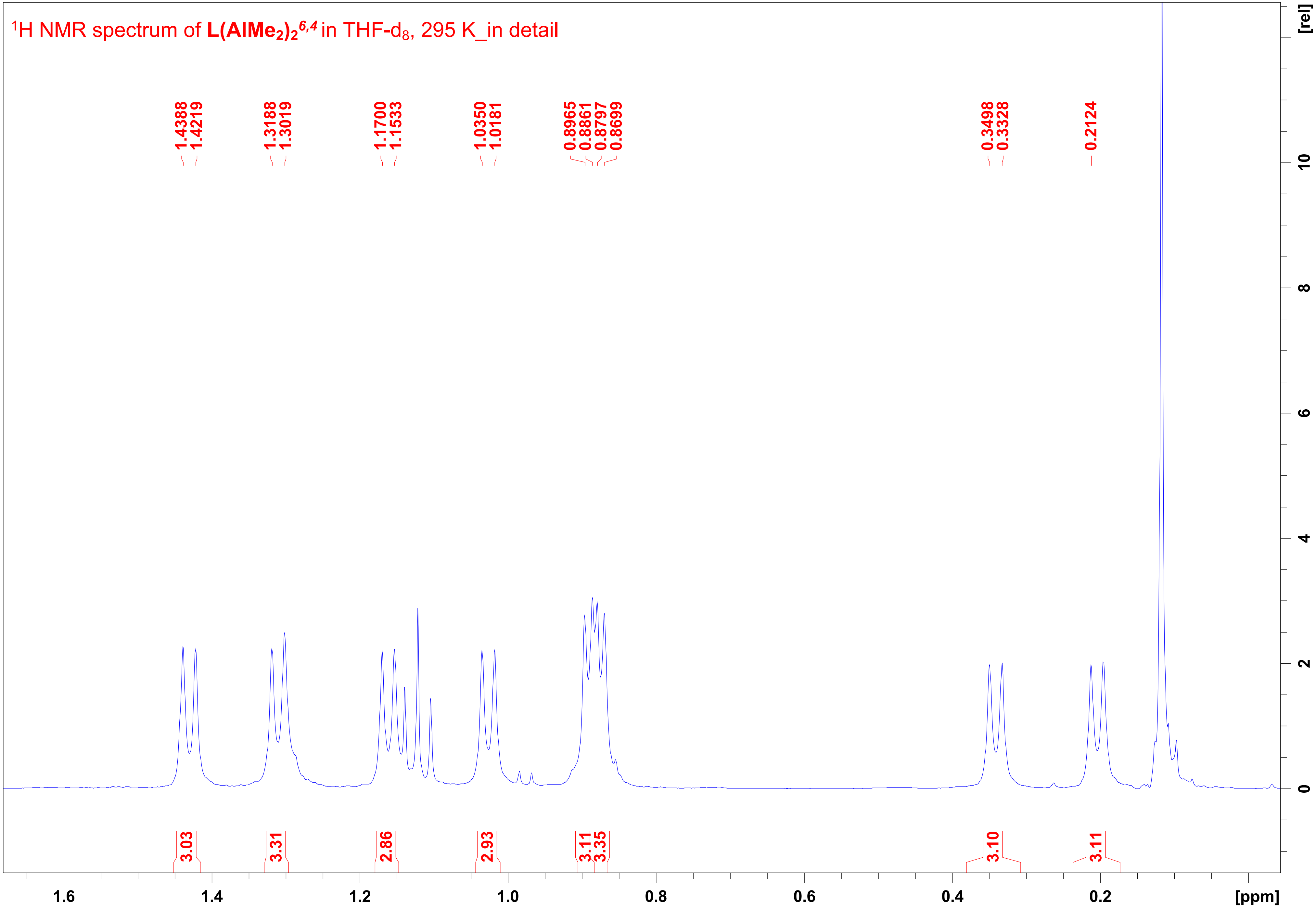
1H NMR spectrum of L(AlMe2)2^6,4 in THF-d8, 295 K_in detail
1.4388
1.4219
1.3188
1.3019
1.1700
1.1533
1.0350
1.0181
0.8965
0.8861
0.8797
0.8699
0.3498
0.3328
0.2124
3.03
3.31
2.86
2.93
3.11
3.35
3.10
3.11
[rel]
[ppm]

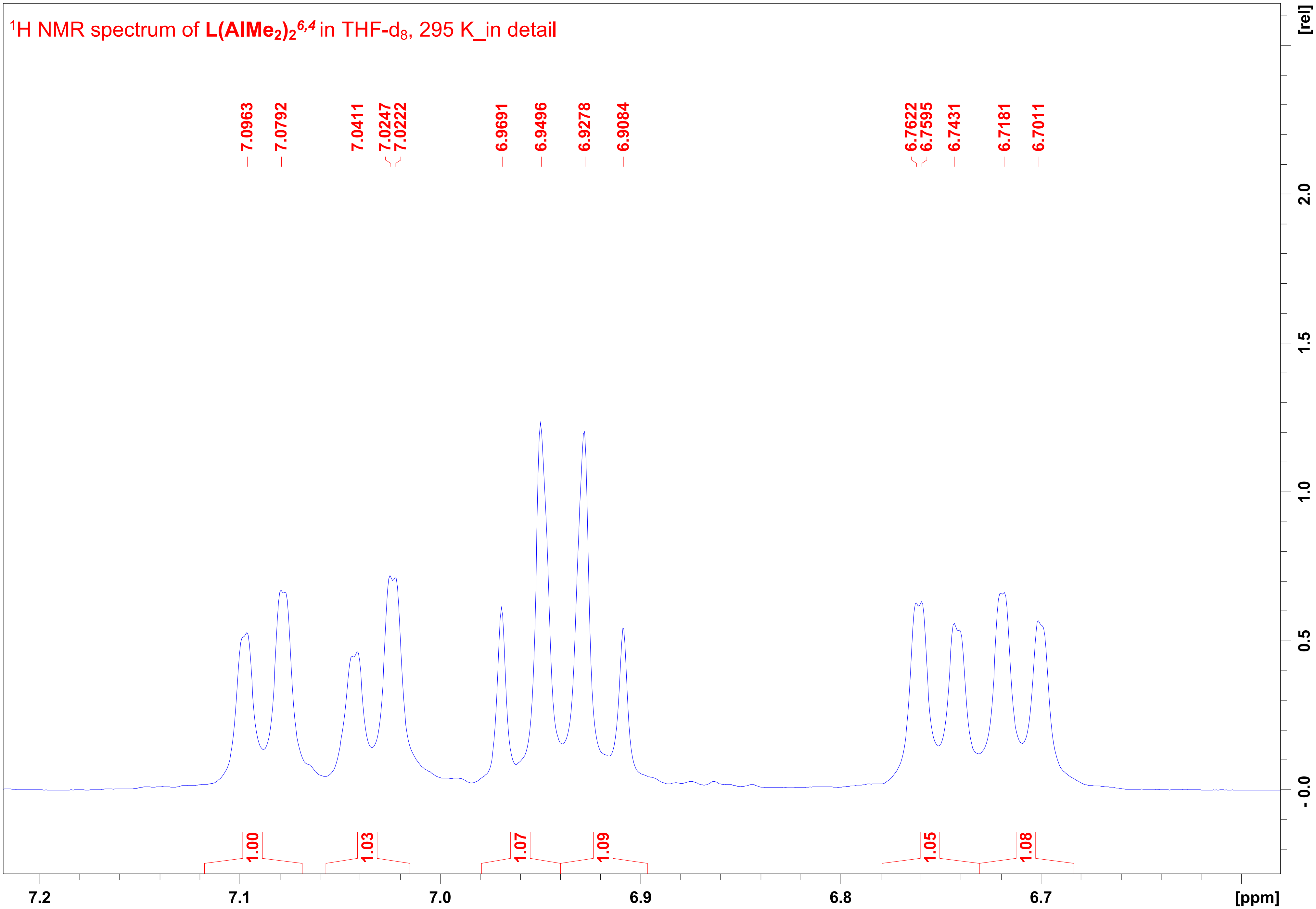

^{1}H NMR spectrum of **L(AlMe$_2$)$_2$^{6,4}** in THF-d$_8$, 295 K_in detail
7.0963
7.0792
7.0411
7.0247
7.0222
6.9691
6.9496
6.9278
6.9084
6.7622
6.7595
6.7431
6.7181
6.7011
1.00
1.03
1.07
1.09
1.05
1.08
[rel]
2.0
1.5
1.0
0.5
-0.0
7.2
7.1
7.0
6.9
6.8
6.7
[ppm]

^{13}C NMR spectrum of **L(AlMe$_2$)$_2$*6,4*** in THF-d$_8$, 295 K

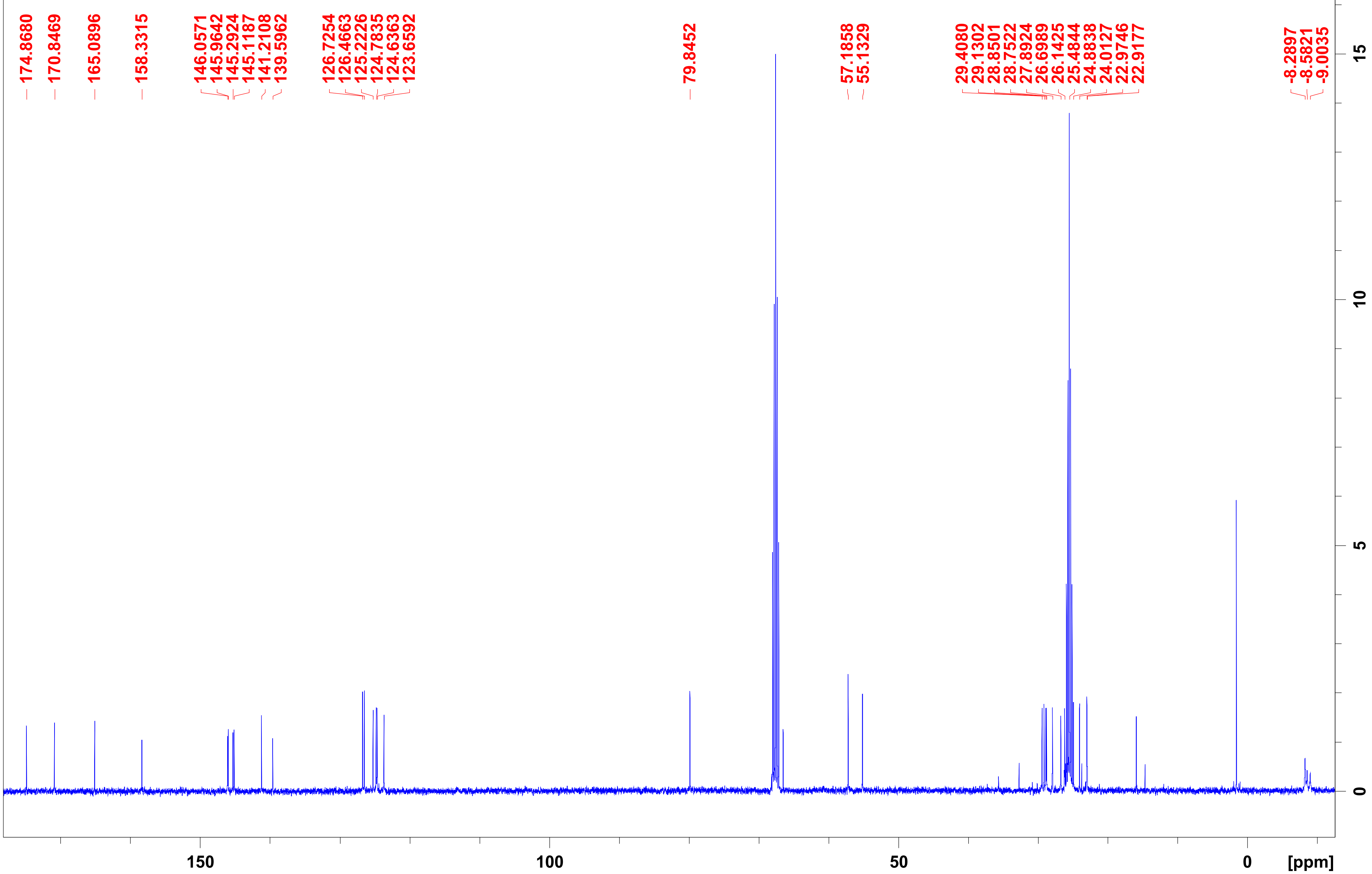

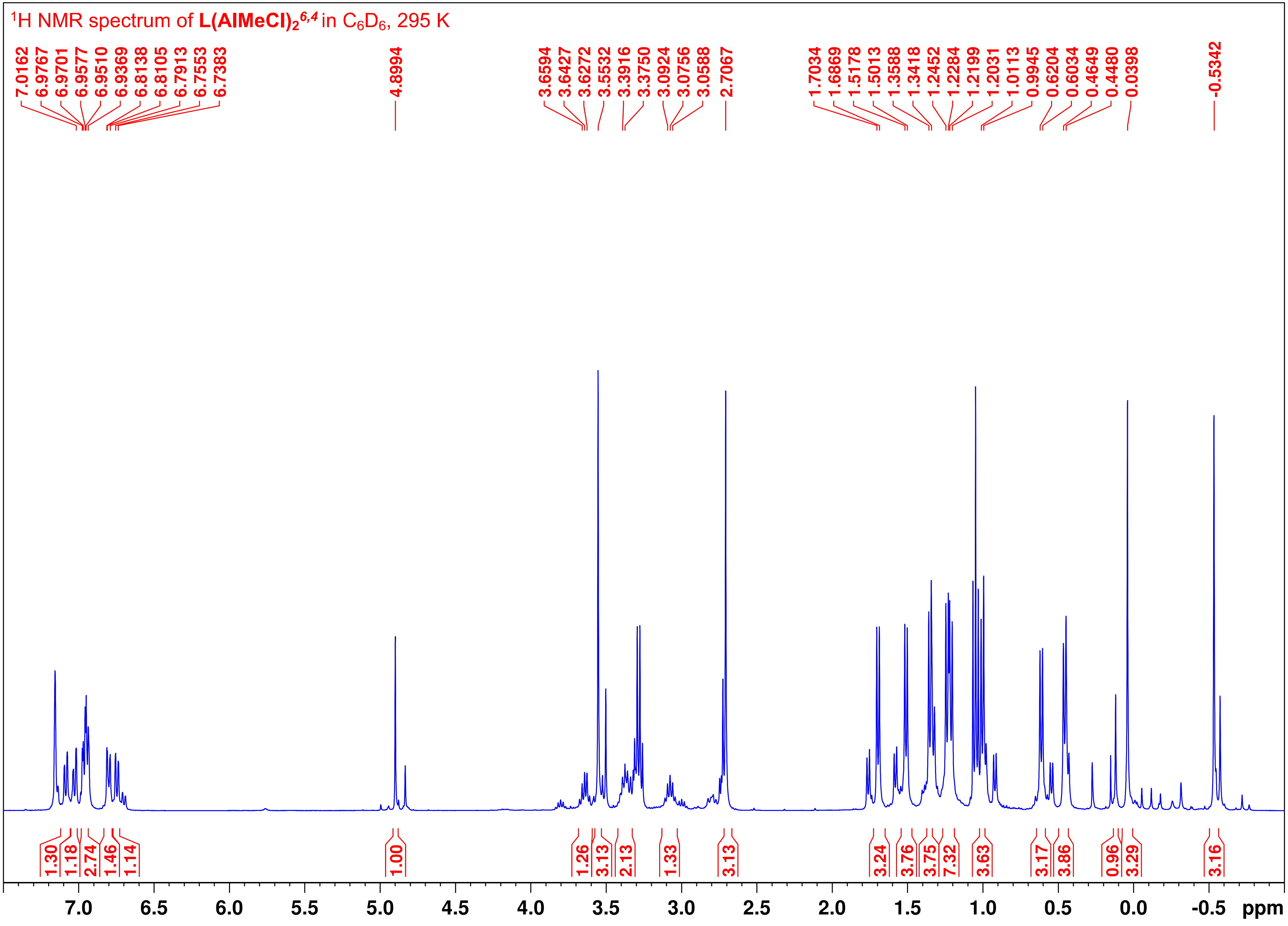

^{1}H NMR spectrum of L(AlMeCl)$_2$ ^{6,4} in C_6D_6, 295 K
7.0162
6.9767
6.9701
6.9577
6.9510
6.9369
6.8138
6.8105
6.7913
6.7553
6.7383
4.8994
3.6594
3.6427
3.6272
3.5532
3.3916
3.3750
3.0924
3.0756
3.0588
2.7067
1.7034
1.6869
1.5178
1.5013
1.3588
1.3418
1.2452
1.2284
1.2199
1.2031
1.0113
0.9945
0.6204
0.6034
0.4649
0.4480
0.0398
-0.5342
1.30
1.18
2.74
1.46
1.14
1.00
1.26
3.13
2.13
1.33
3.13
3.24
3.76
3.75
7.32
3.63
3.17
3.86
0.96
3.29
3.16
7.0
6.5
6.0
5.5
5.0
4.5
4.0
3.5
3.0
2.5
2.0
1.5
1.0
0.5
0.0
-0.5
ppm

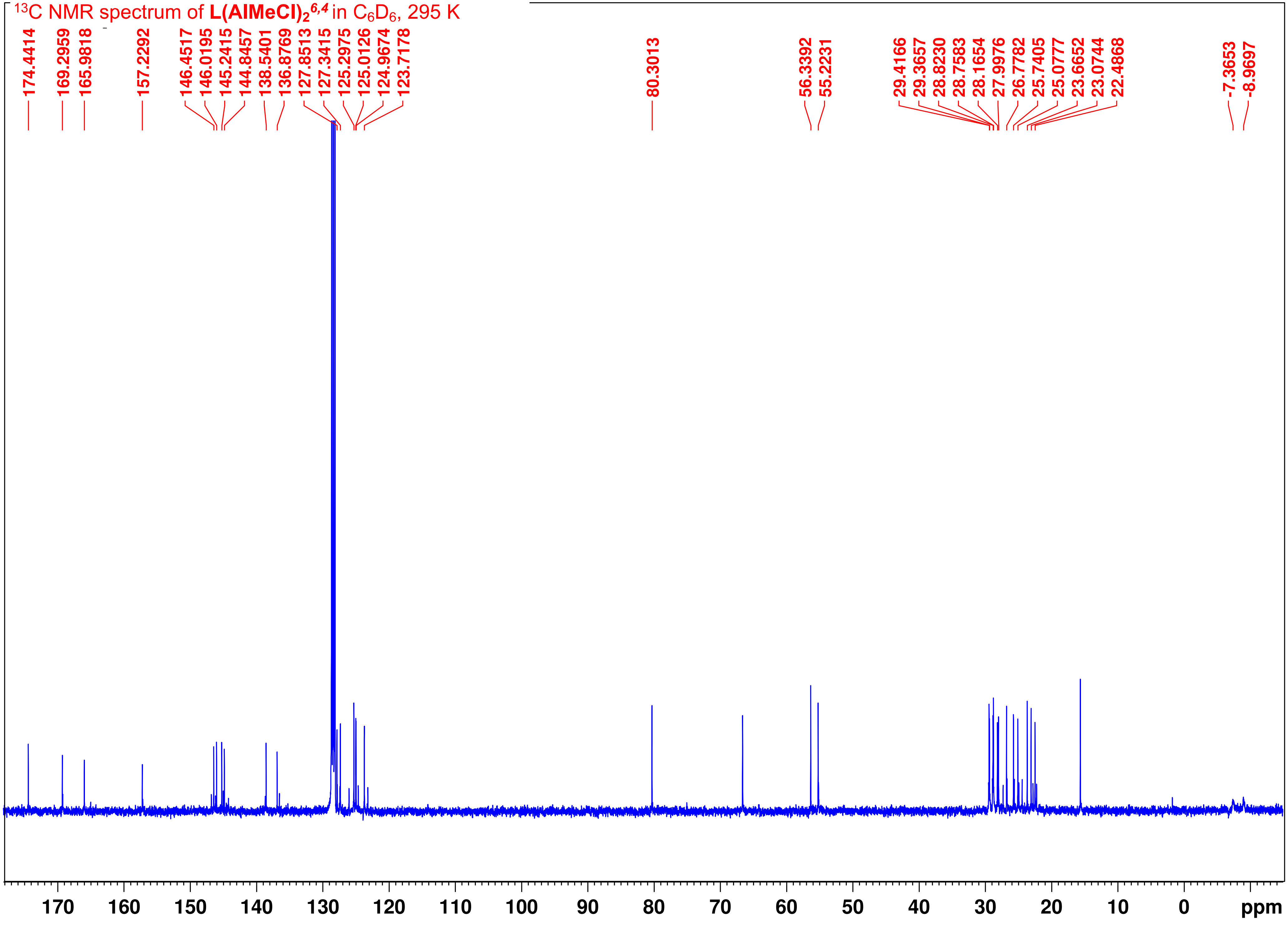
^{13}C NMR spectrum of **L(AlMeCl)$_2$^{6,4}** in C_6D_6, 295 K
174.4414
169.2959
165.9818
157.2292
146.4517
146.0195
145.2415
144.8457
138.5401
136.8769
127.8513
127.3415
125.2975
125.0126
124.9674
123.7178
80.3013
56.3392
55.2231
29.4166
29.3657
28.8230
28.7583
28.1654
27.9976
26.7782
25.7405
25.0777
23.6652
23.0744
22.4868
-7.3653
-8.9697
170
160
150
140
130
120
110
100
90
80
70
60
50
40
30
20
10
0
ppm

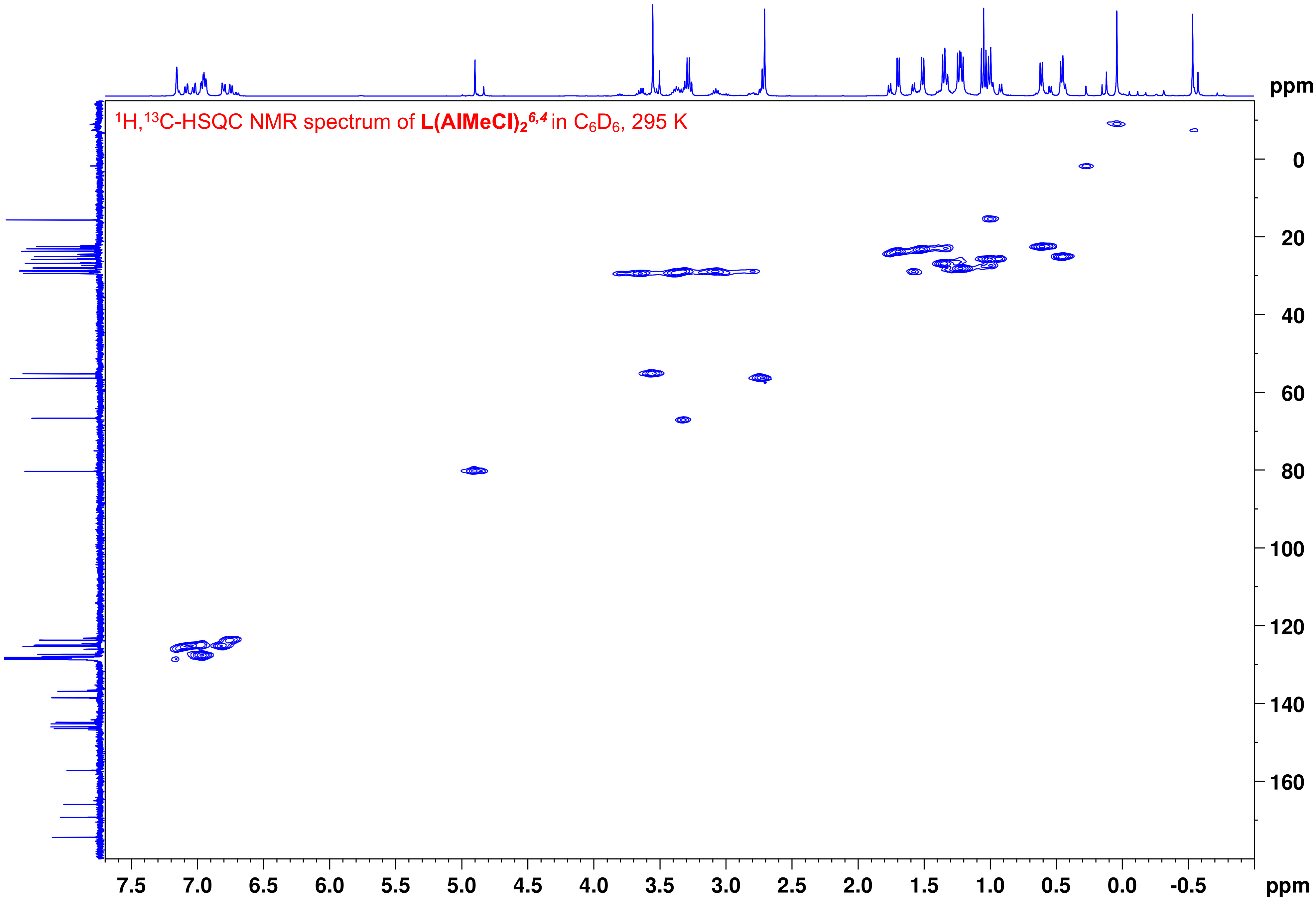
$^{1}H,^{13}C$-HSQC NMR spectrum of **$L(AlMeCl)_2$*^{6,4}*** in C_6D_6, 295 K
ppm
0
20
40
60
80
100
120
140
160
7.5
7.0
6.5
6.0
5.5
5.0
4.5
4.0
3.5
3.0
2.5
2.0
1.5
1.0
0.5
0.0
-0.5
ppm

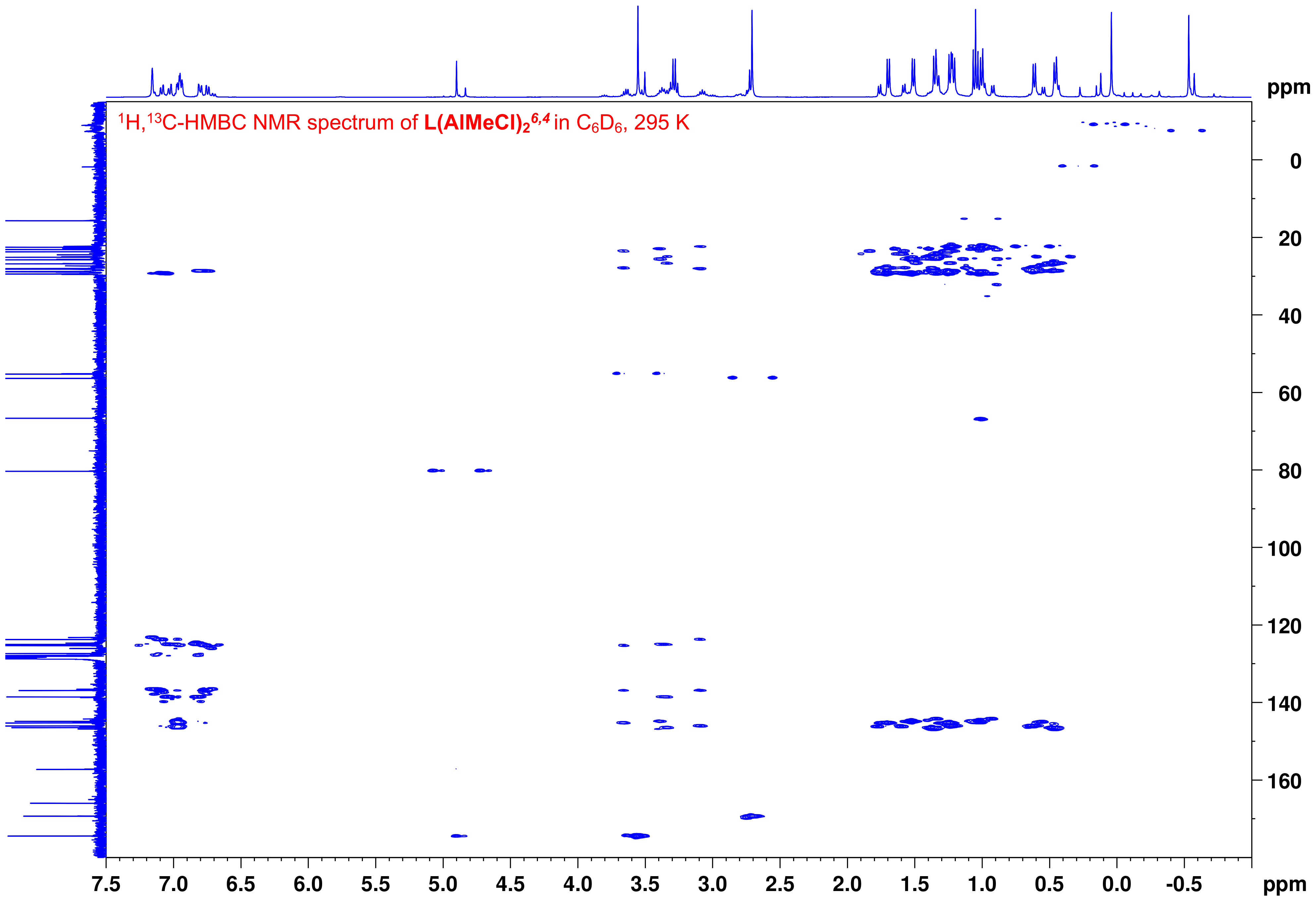
$^1H,^{13}C$-HMBC NMR spectrum of **$L(AlMeCl)_2$**^{6,4} in C_6D_6, 295 K
ppm
ppm

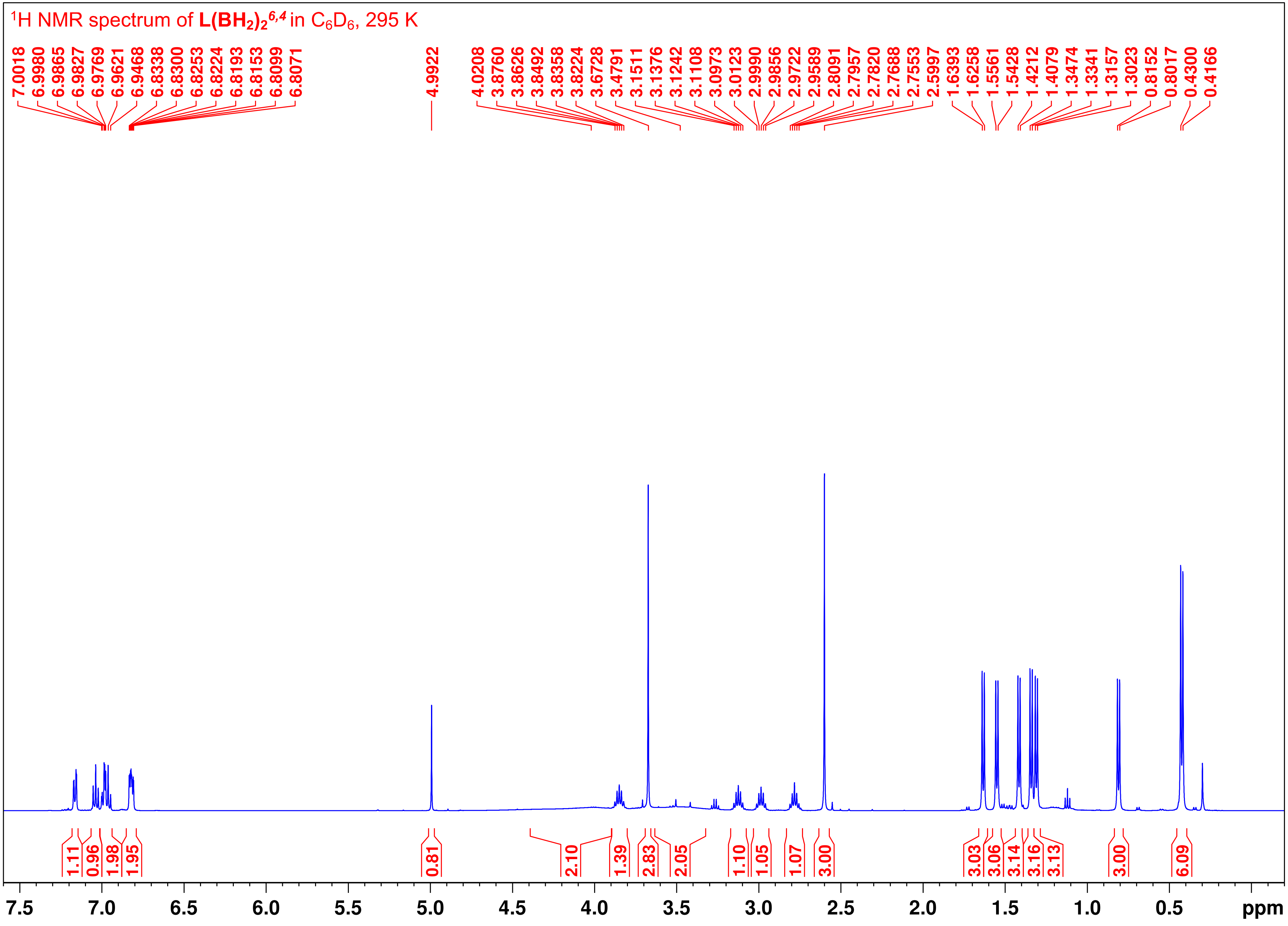
1H NMR spectrum of L(BH2)2 6,4 in C6D6, 295 K
7.0018
6.9980
6.9865
6.9827
6.9769
6.9621
6.9468
6.8338
6.8300
6.8253
6.8224
6.8193
6.8153
6.8099
6.8071
4.9922
4.0208
3.8760
3.8626
3.8492
3.8358
3.8224
3.6728
3.4791
3.1511
3.1376
3.1242
3.1108
3.0973
3.0123
2.9990
2.9856
2.9722
2.9589
2.8091
2.7957
2.7820
2.7688
2.7553
2.5997
1.6393
1.6258
1.5561
1.5428
1.4212
1.4079
1.3474
1.3341
1.3157
1.3023
0.8152
0.8017
0.4300
0.4166
1.11
0.96
1.98
1.95
0.81
2.10
1.39
2.83
2.05
1.10
1.05
1.07
3.00
3.03
3.06
3.14
3.16
3.13
3.00
6.09
7.5
7.0
6.5
6.0
5.5
5.0
4.5
4.0
3.5
3.0
2.5
2.0
1.5
1.0
0.5
ppm

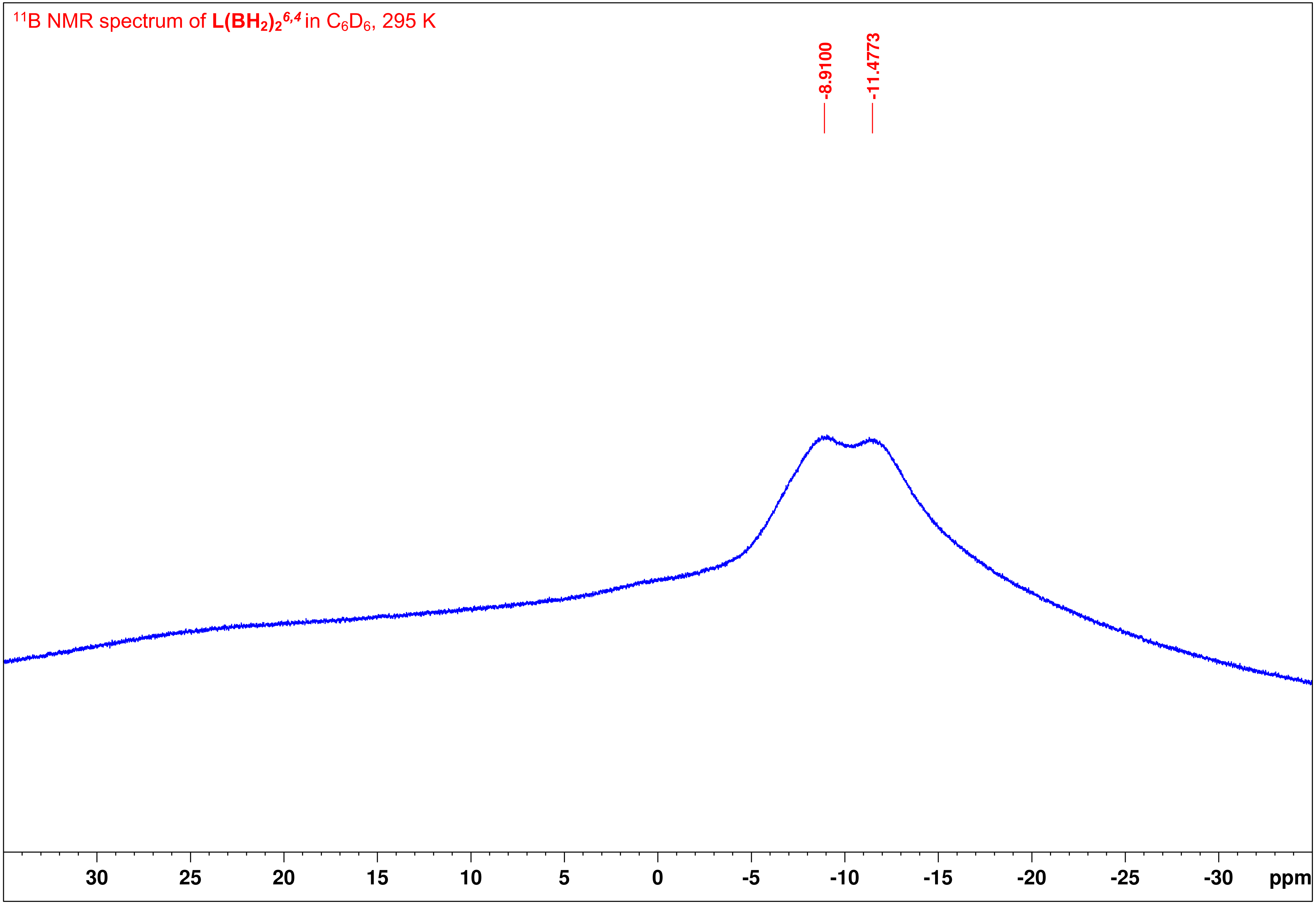
^{11}B NMR spectrum of **L(BH$_2$)$_2$**^{6,4} in C_6D_6, 295 K
-8.9100
-11.4773
30 25 20 15 10 5 0 -5 -10 -15 -20 -25 -30 ppm

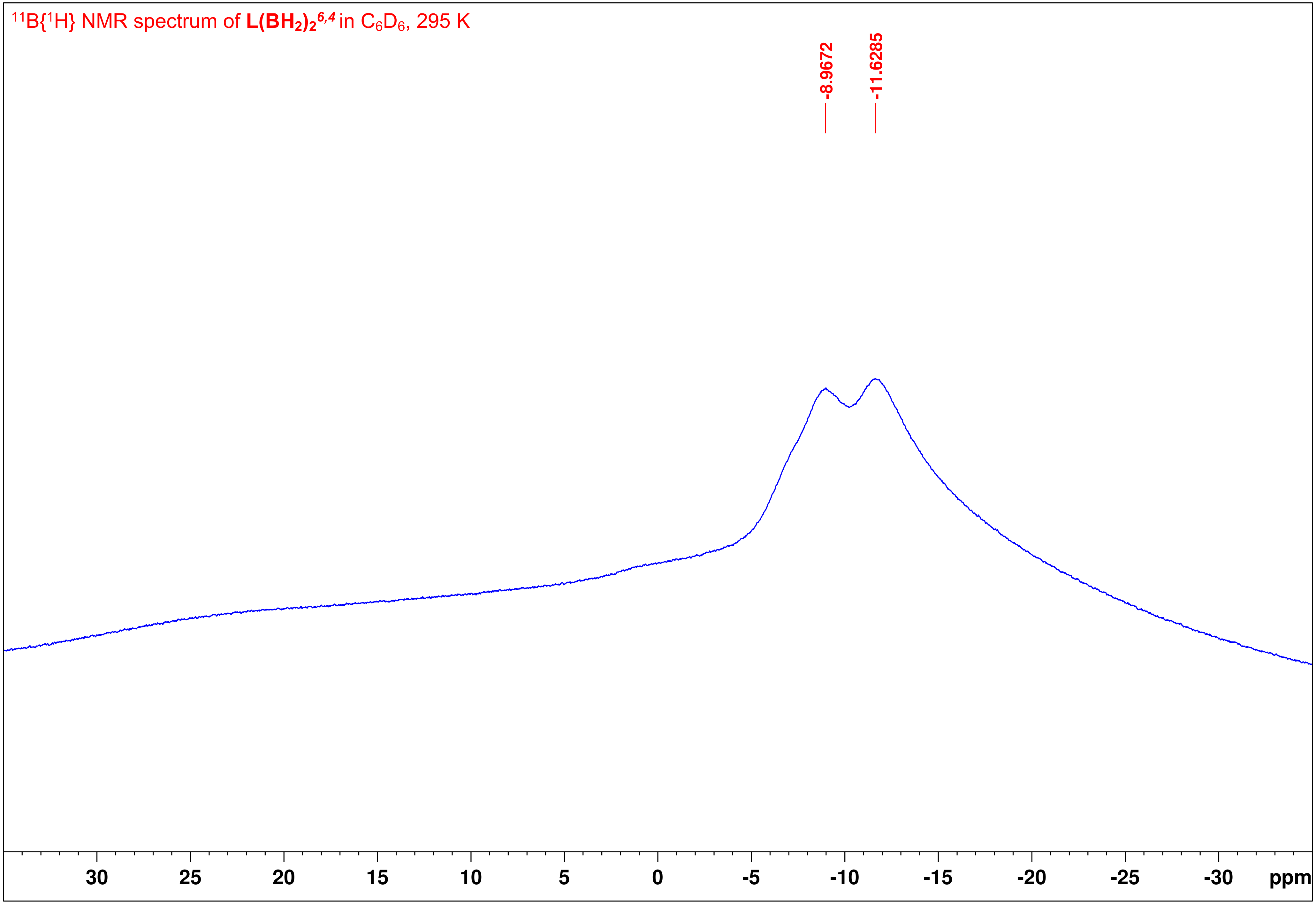

$^{11}B\{^{1}H\}$ NMR spectrum of $\mathbf{L(BH_2)_2}^{6,4}$ in C_6D_6, 295 K
-8.9672
-11.6285
30
25
20
15
10
5
0
-5
-10
-15
-20
-25
-30
ppm

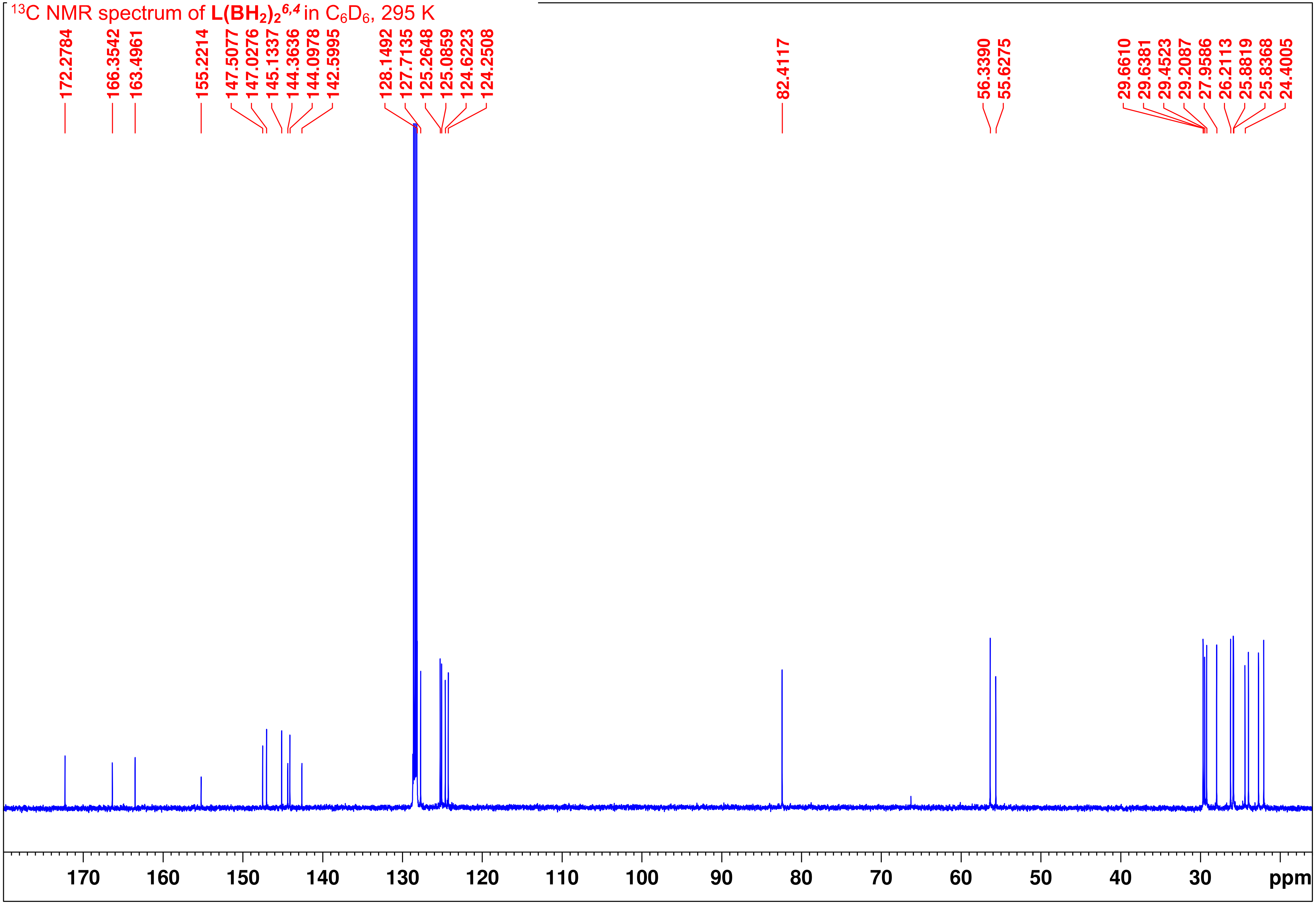

^{13}C NMR spectrum of $\mathbf{L(BH_2)_2}^{6,4}$ in C_6D_6, 295 K
172.2784
166.3542
163.4961
155.2214
147.5077
147.0276
145.1337
144.3636
144.0978
142.5995
128.1492
127.7135
125.2648
125.0859
124.6223
124.2508
82.4117
56.3390
55.6275
29.6610
29.6381
29.4523
29.2087
27.9586
26.2113
25.8819
25.8368
24.4005
170
160
150
140
130
120
110
100
90
80
70
60
50
40
30
ppm

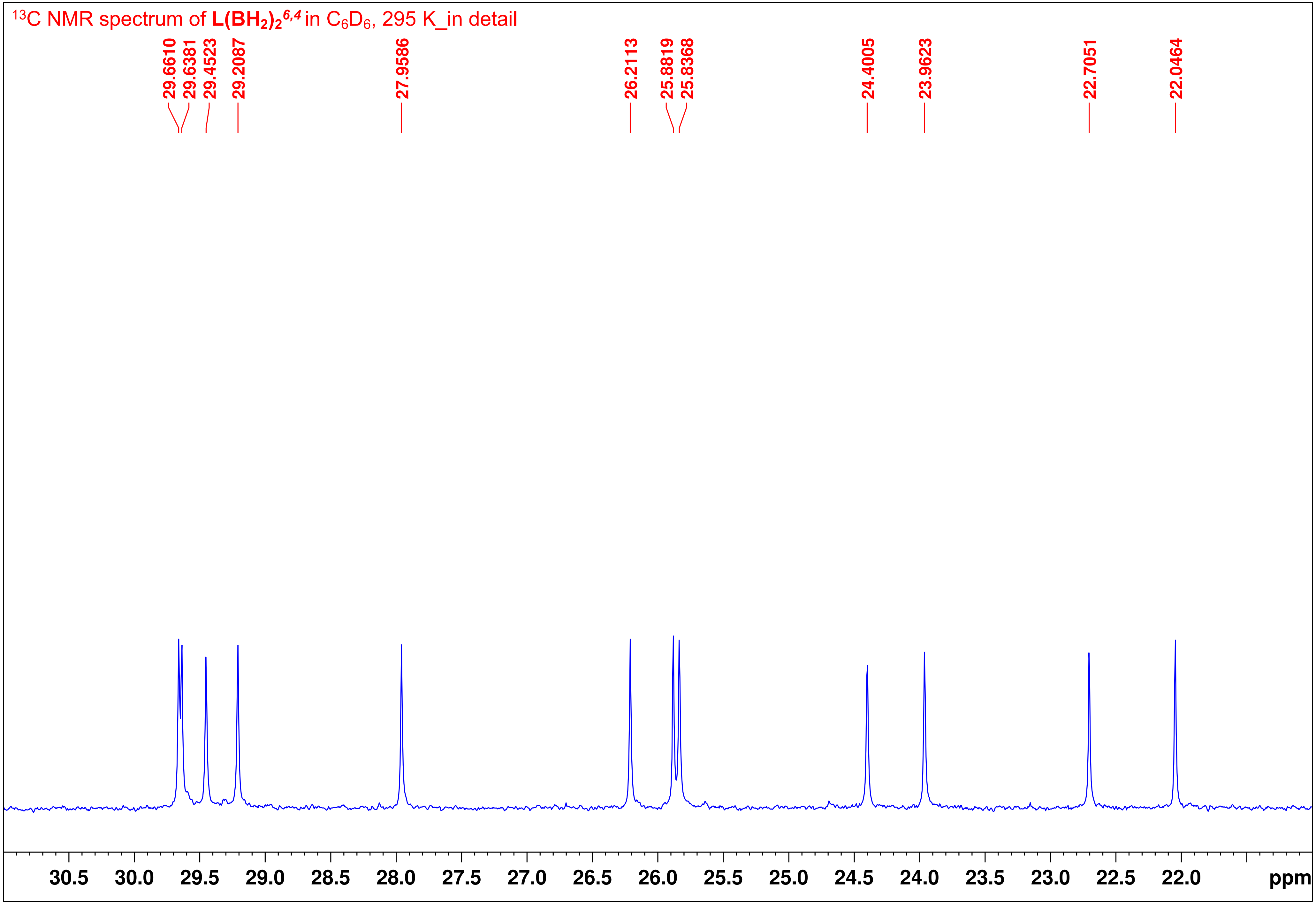

^{13}C NMR spectrum of **$L(BH_2)_2$**^{6,4} in C_6D_6, 295 K_in detail
29.6610
29.6381
29.4523
29.2087
27.9586
26.2113
25.8819
25.8368
24.4005
23.9623
22.7051
22.0464
30.5
30.0
29.5
29.0
28.5
28.0
27.5
27.0
26.5
26.0
25.5
25.0
24.5
24.0
23.5
23.0
22.5
22.0
ppm

^{13}C APT NMR spectrum of **$L(BH_2)_2$**^{6,4} in C_6D_6, 295 K

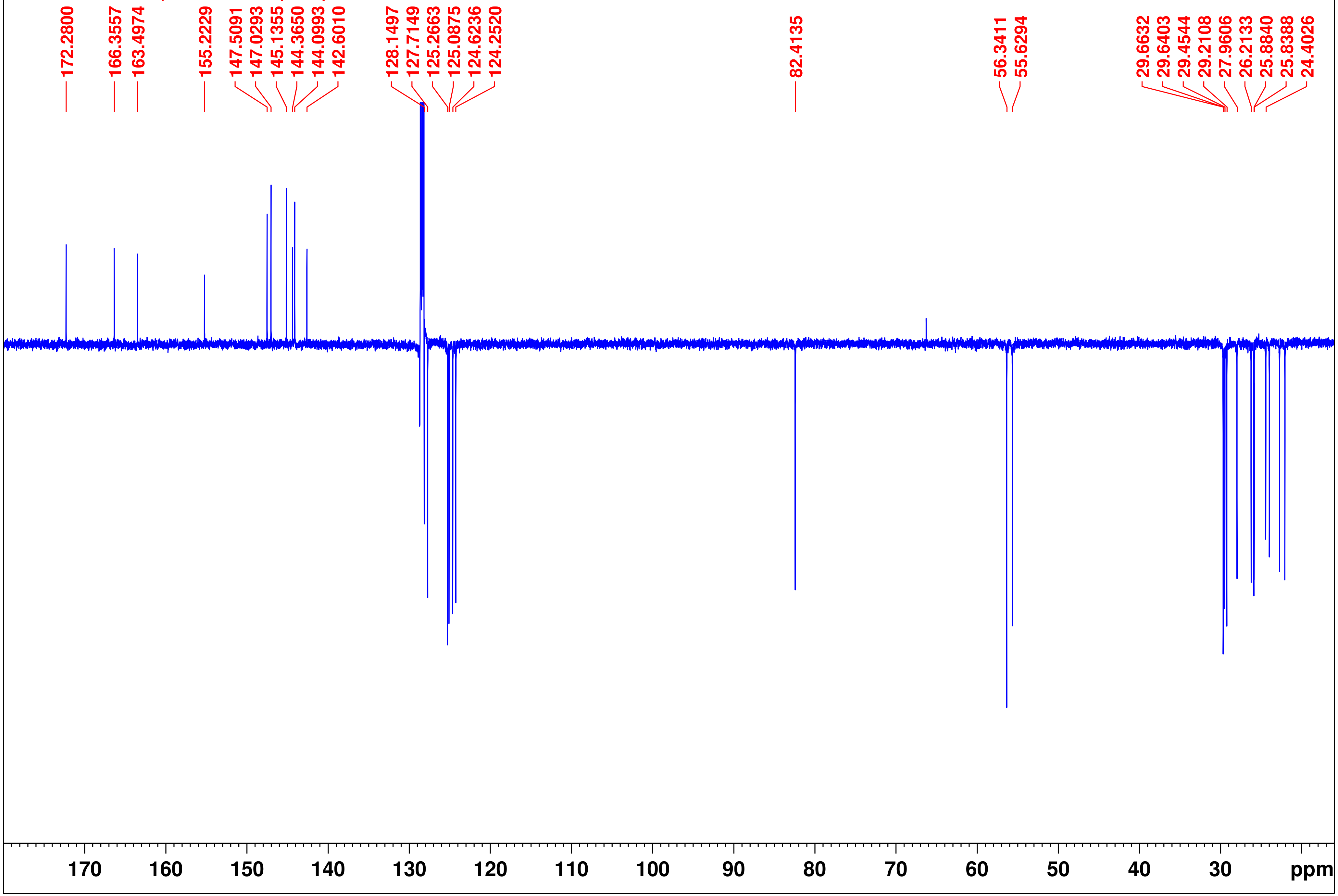

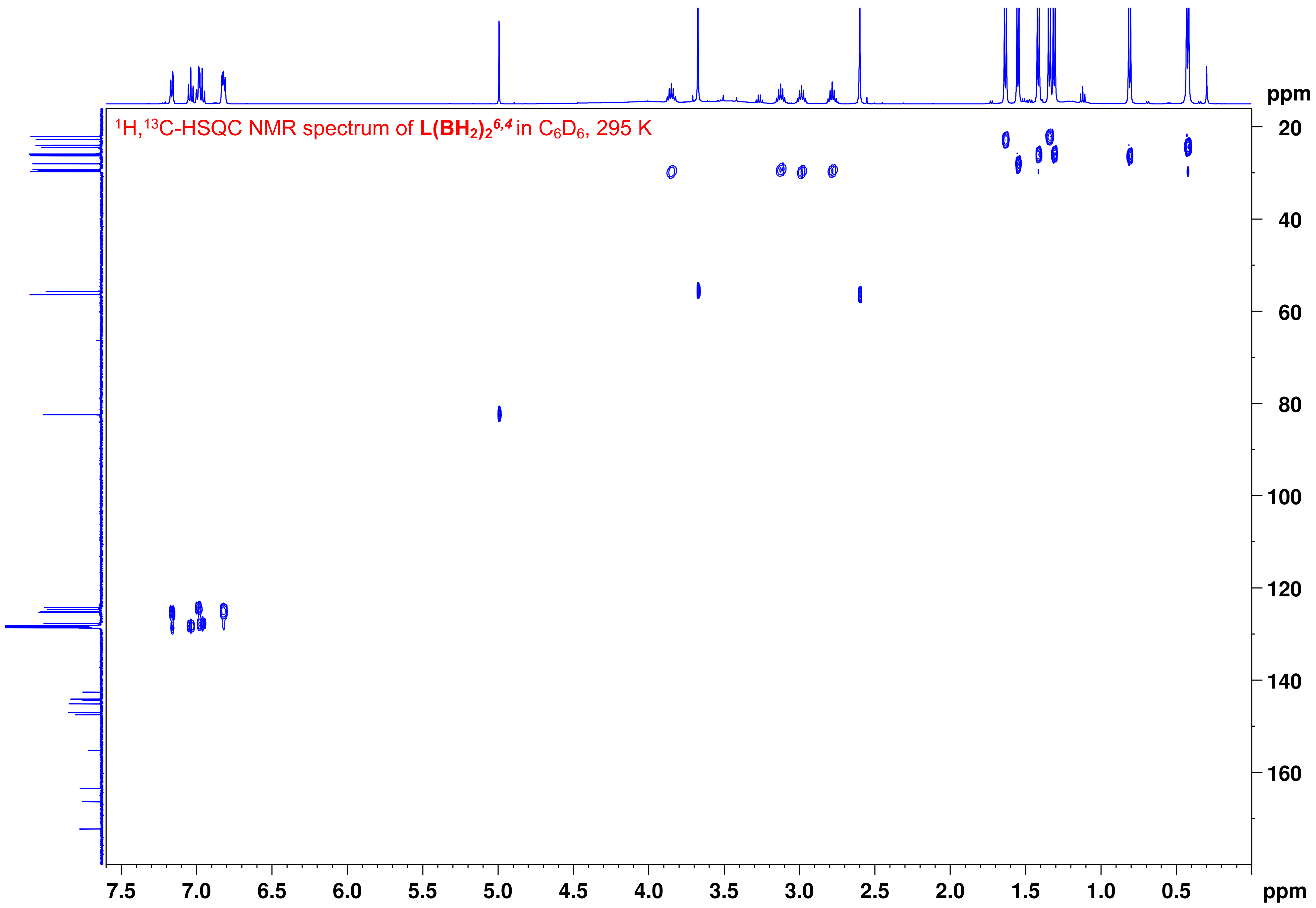

$^{1}H,^{13}C$-HSQC NMR spectrum of $\mathbf{L(BH_2)_2}^{6,4}$ in C_6D_6, 295 K
ppm
20
40
60
80
100
120
140
160
7.5
7.0
6.5
6.0
5.5
5.0
4.5
4.0
3.5
3.0
2.5
2.0
1.5
1.0
0.5
ppm

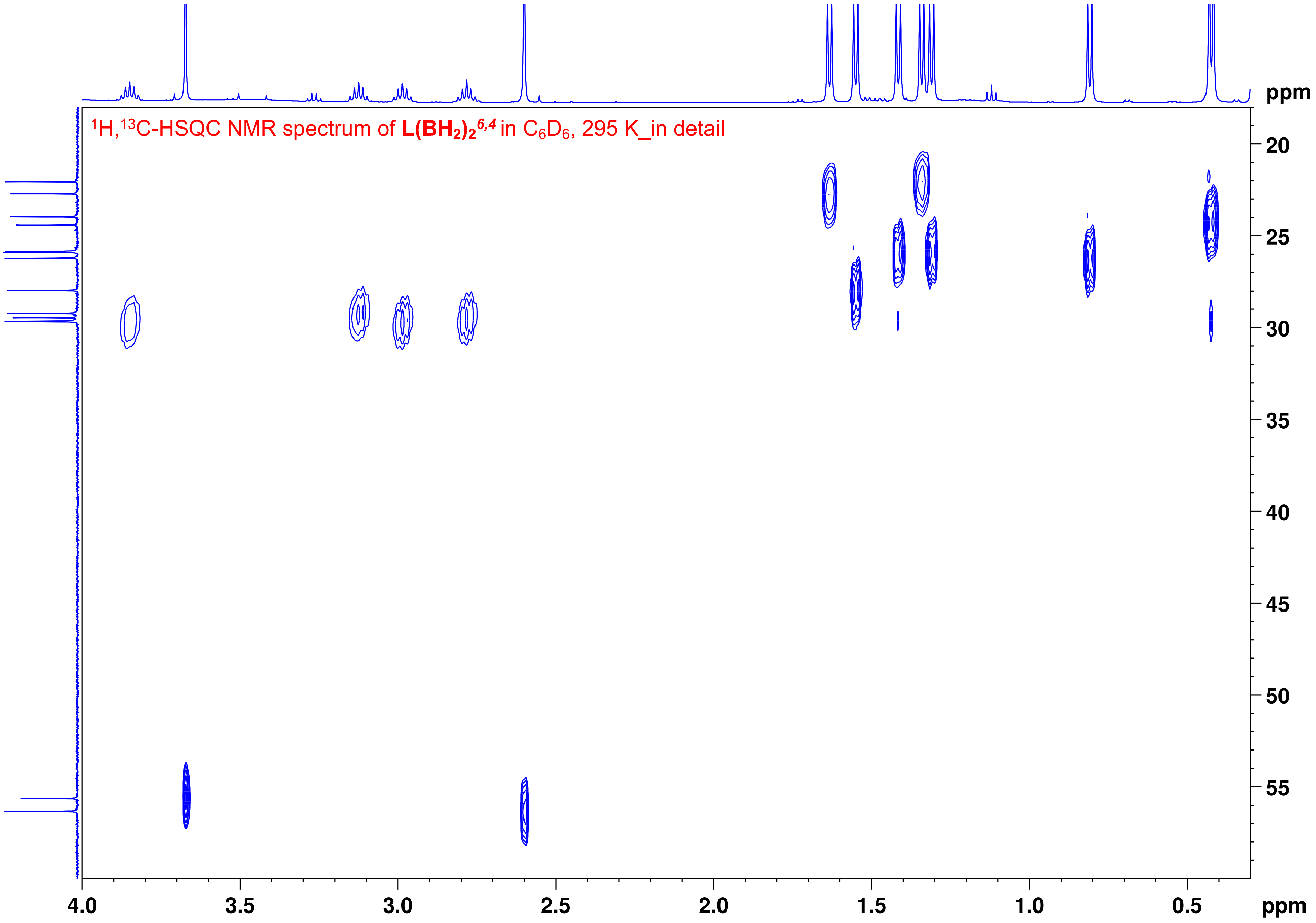

$^1H,^{13}C$-HSQC NMR spectrum of **L(BH$_2$)$_2$**^{6,4} in C_6D_6, 295 K_in detail
ppm
20
25
30
35
40
45
50
55
4.0
3.5
3.0
2.5
2.0
1.5
1.0
0.5
ppm

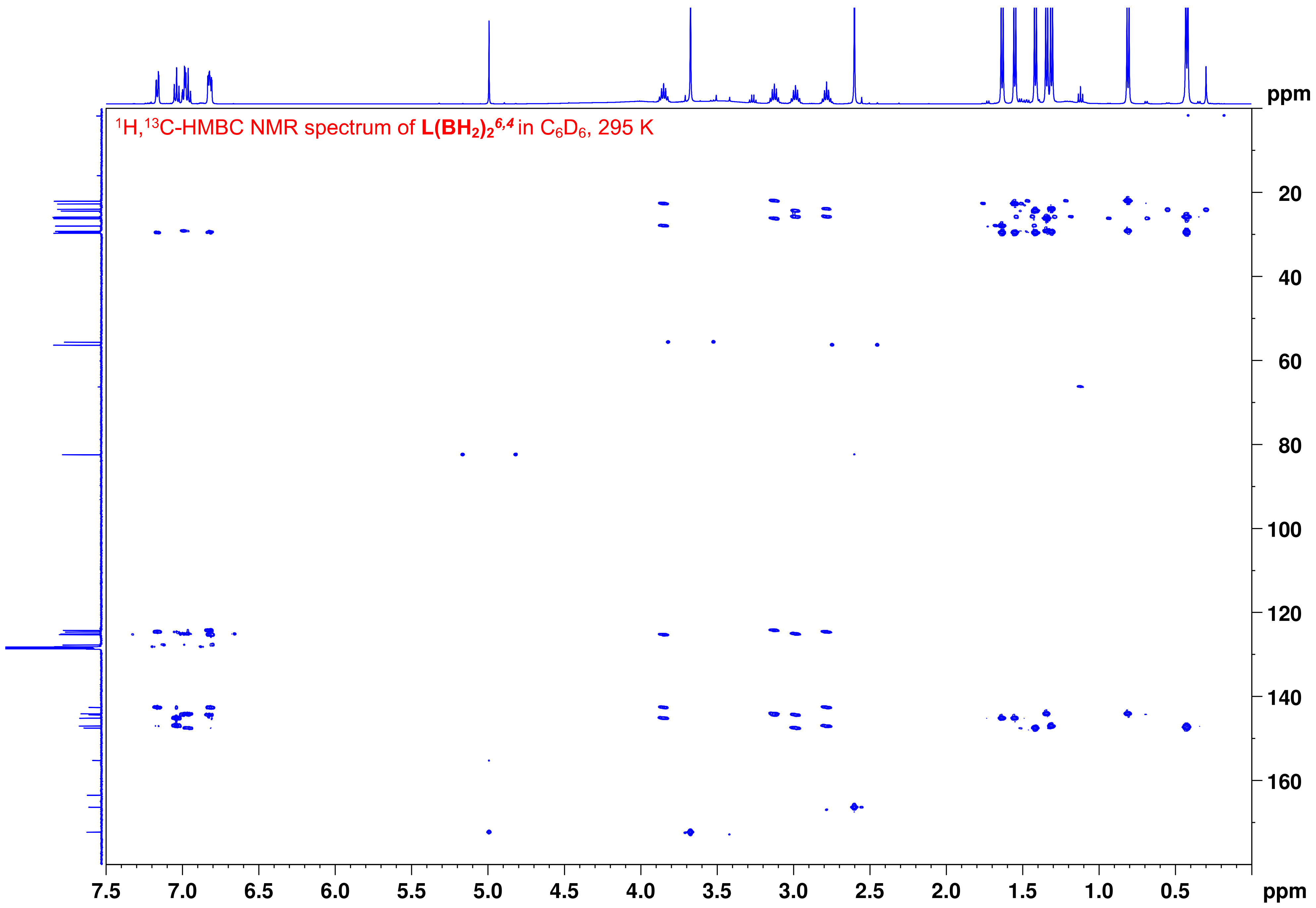

1H,13C-HMBC NMR spectrum of L(BH2)2 6,4 in C6D6, 295 K
ppm
20
40
60
80
100
120
140
160
7.5
7.0
6.5
6.0
5.5
5.0
4.5
4.0
3.5
3.0
2.5
2.0
1.5
1.0
0.5

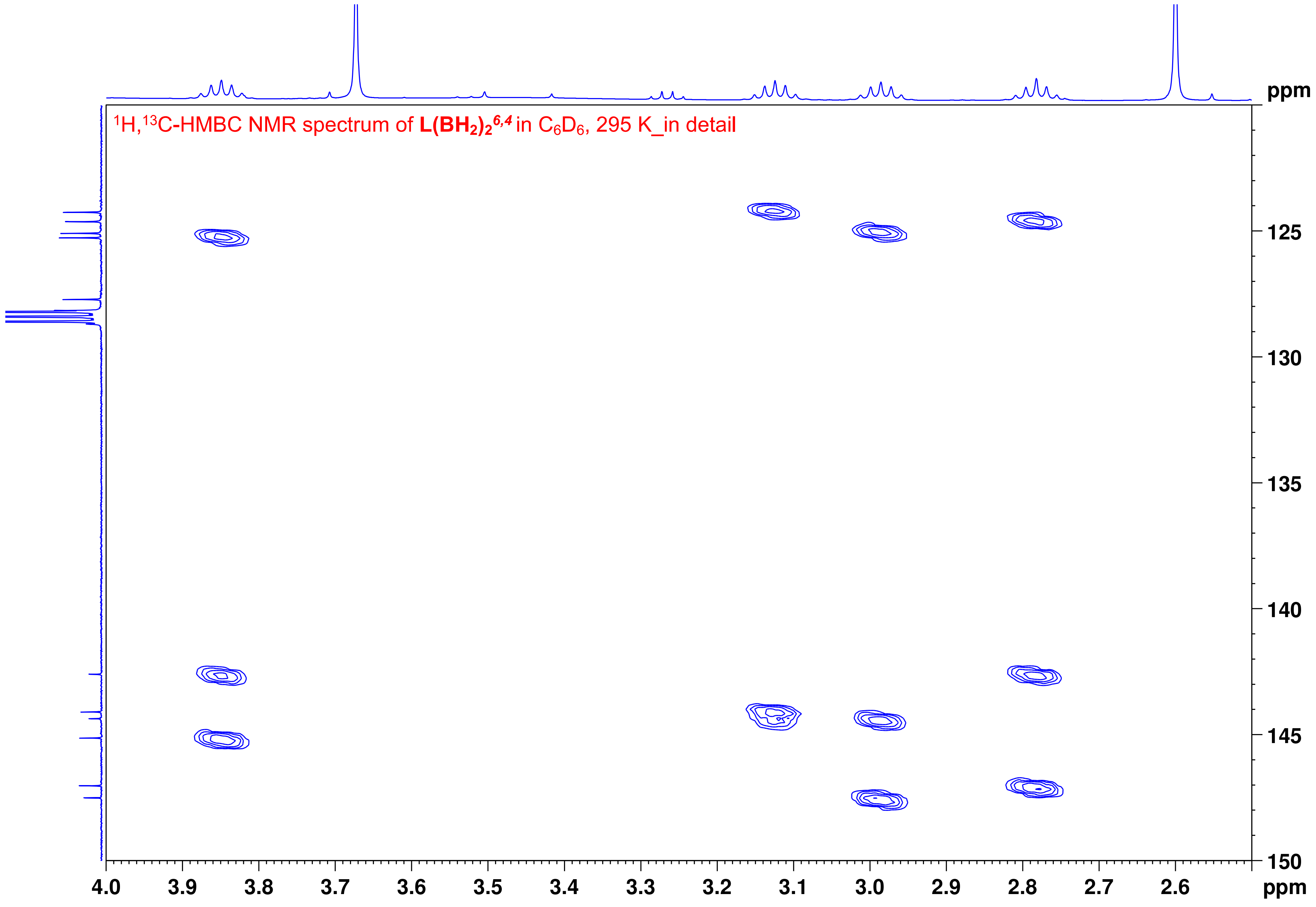
$^1H,^{13}C$-HMBC NMR spectrum of $\mathbf{L(BH_2)_2}^{\mathbf{6,4}}$ in C_6D_6, 295 K_in detail
ppm
125
130
135
140
145
150
4.0
3.9
3.8
3.7
3.6
3.5
3.4
3.3
3.2
3.1
3.0
2.9
2.8
2.7
2.6
ppm

^1H NMR spectrum of **$L(AlMe_2)^6(AlMeI)^4$** in C_6D_6, 295 K

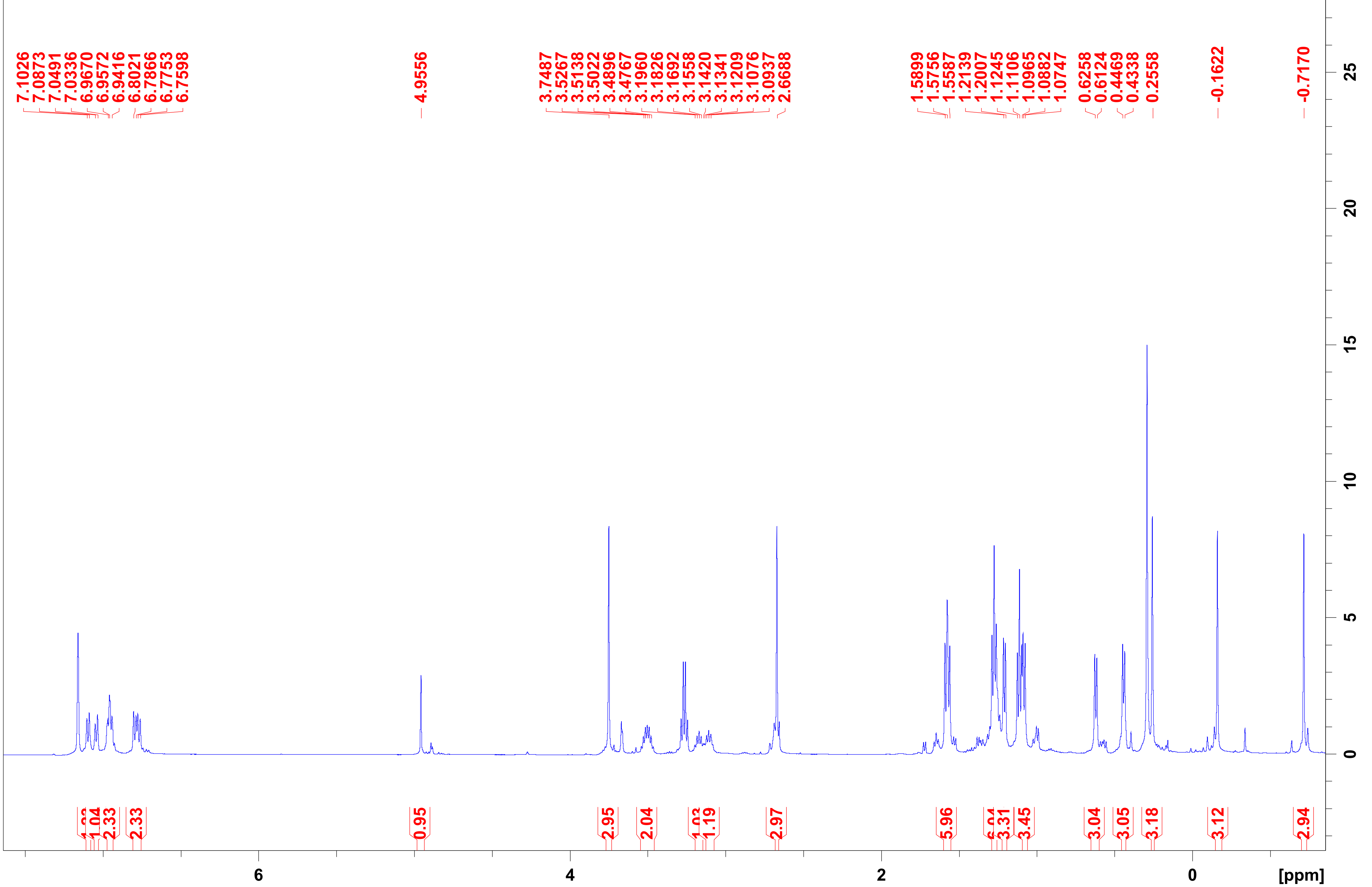

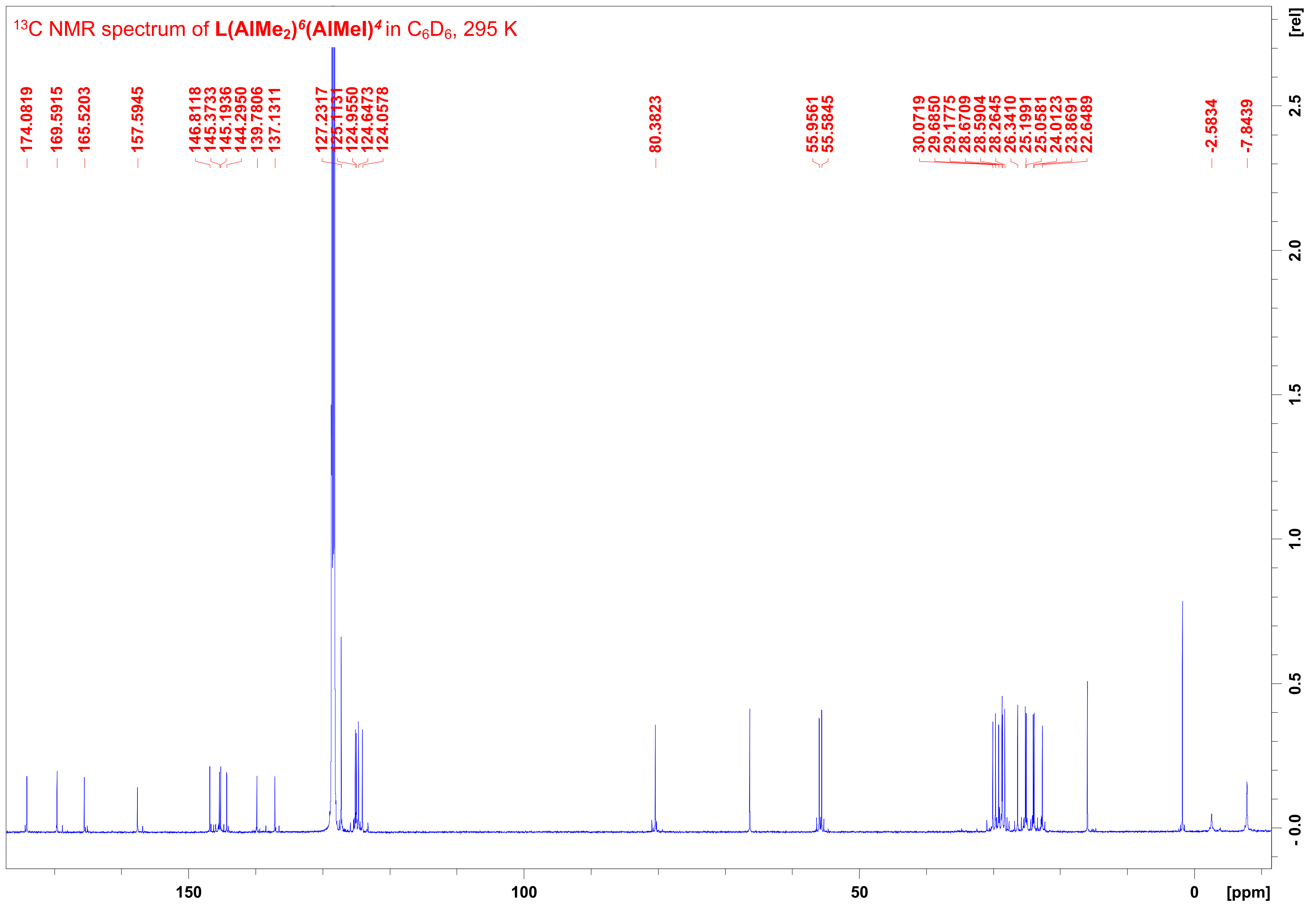

13C NMR spectrum of L(AlMe2)6(AlMeI)4 in C6D6, 295 K
174.0819
169.5915
165.5203
157.5945
146.8118
145.3733
145.1936
144.2950
139.7806
137.1311
127.2317
125.1131
124.9550
124.6473
124.0578
80.3823
55.9561
55.5845
30.0719
29.6850
29.1775
28.6709
28.5904
28.2645
26.3410
25.1991
25.0581
24.0123
23.8691
22.6489
-2.5834
-7.8439
[rel]
2.5
2.0
1.5
1.0
0.5
0.0
150
100
50
0
[ppm]

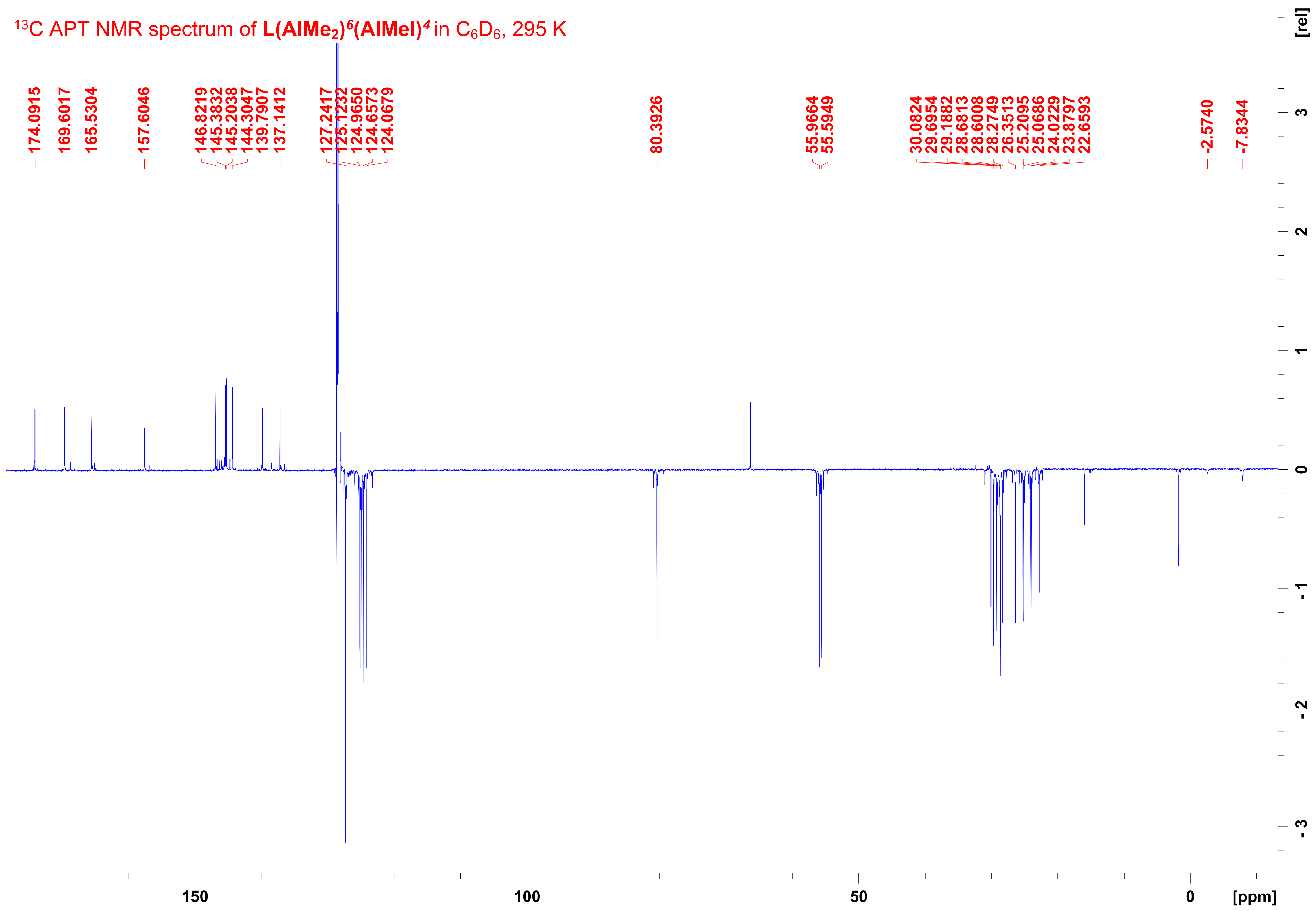

^{13}C APT NMR spectrum of **L(AlMe$_2$)^6(AlMeI)^4** in C_6D_6, 295 K
174.0915
169.6017
165.5304
157.6046
146.8219
145.3832
145.2038
144.3047
139.7907
137.1412
127.2417
125.1232
124.9650
124.6573
124.0679
80.3926
55.9664
55.5949
30.0824
29.6954
29.1882
28.6813
28.6008
28.2749
26.3513
25.2095
25.0686
24.0229
23.8797
22.6593
-2.5740
-7.8344
150
100
50
0
[ppm]
[rel]
3
2
1
0
-1
-2
-3

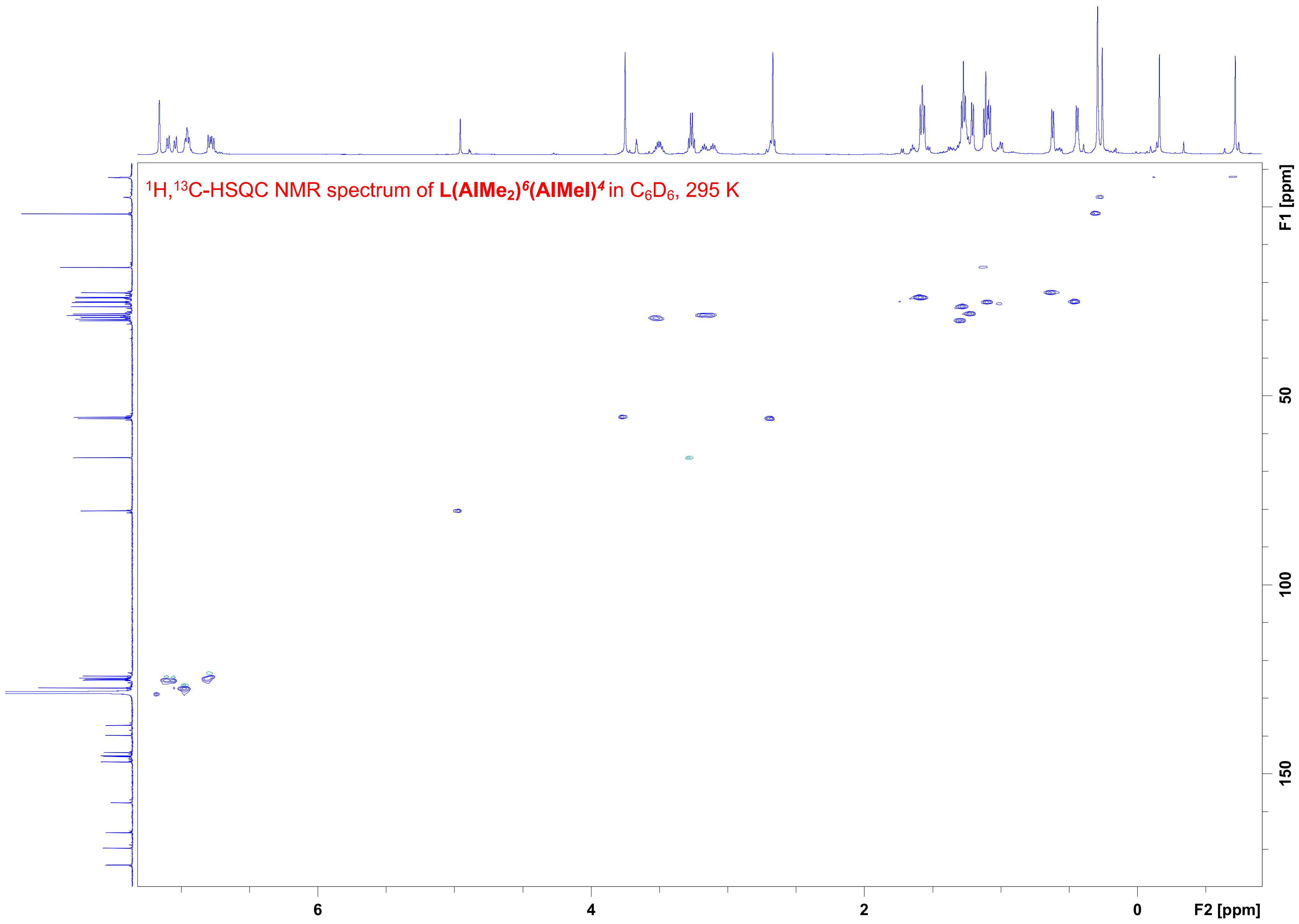
$^{1}H,^{13}C$-HSQC NMR spectrum of **L(AlMe$_2$)^6(AlMeI)^4** in C_6D_6, 295 K
F1 [ppm]
50
100
150
F2 [ppm]
6
4
2
0

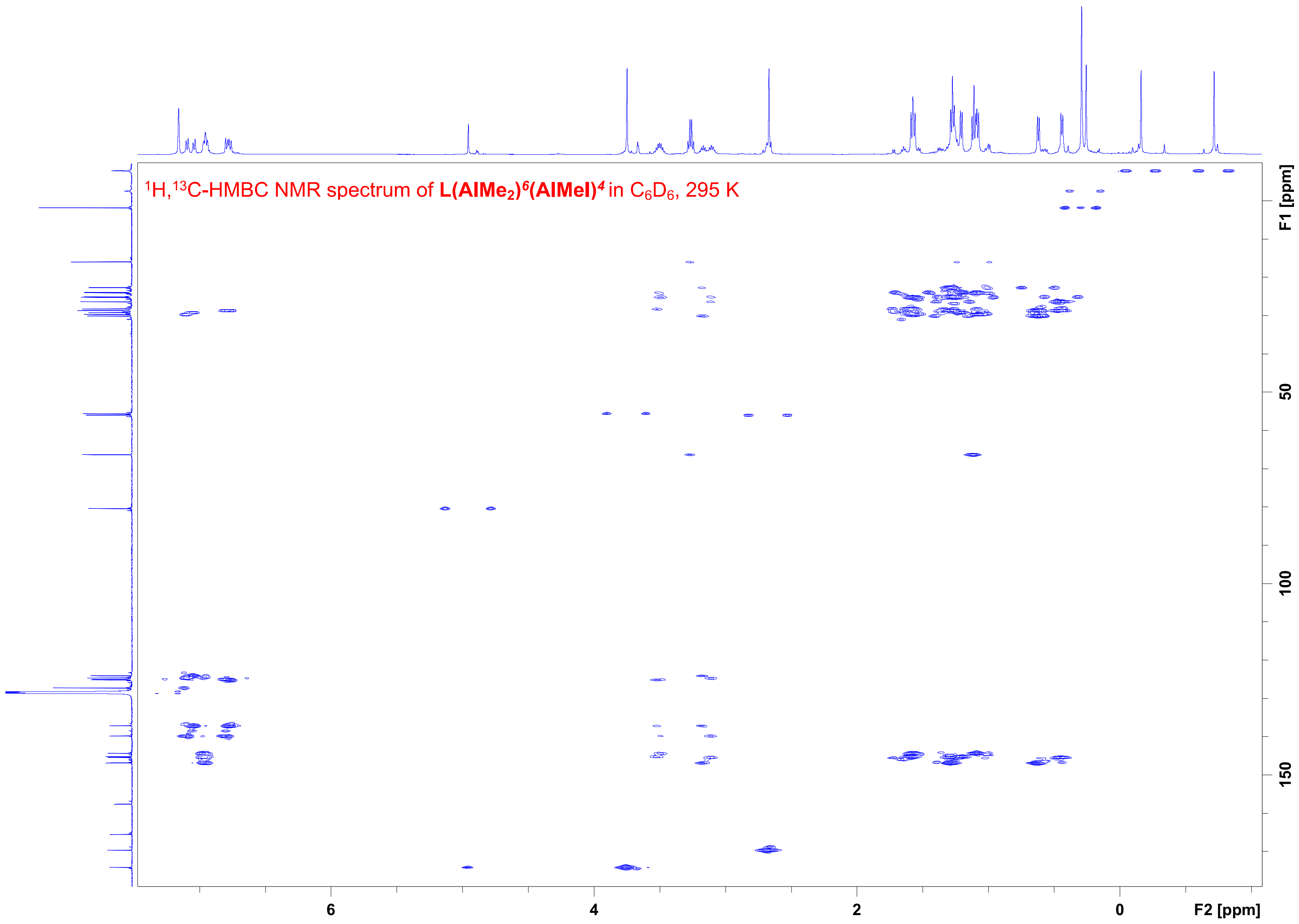
^1H,^{13}C-HMBC NMR spectrum of **L(AlMe$_2$)^6(AlMeI)^4** in C_6D_6, 295 K
F1 [ppm]
50
100
150
6
4
2
0
F2 [ppm]

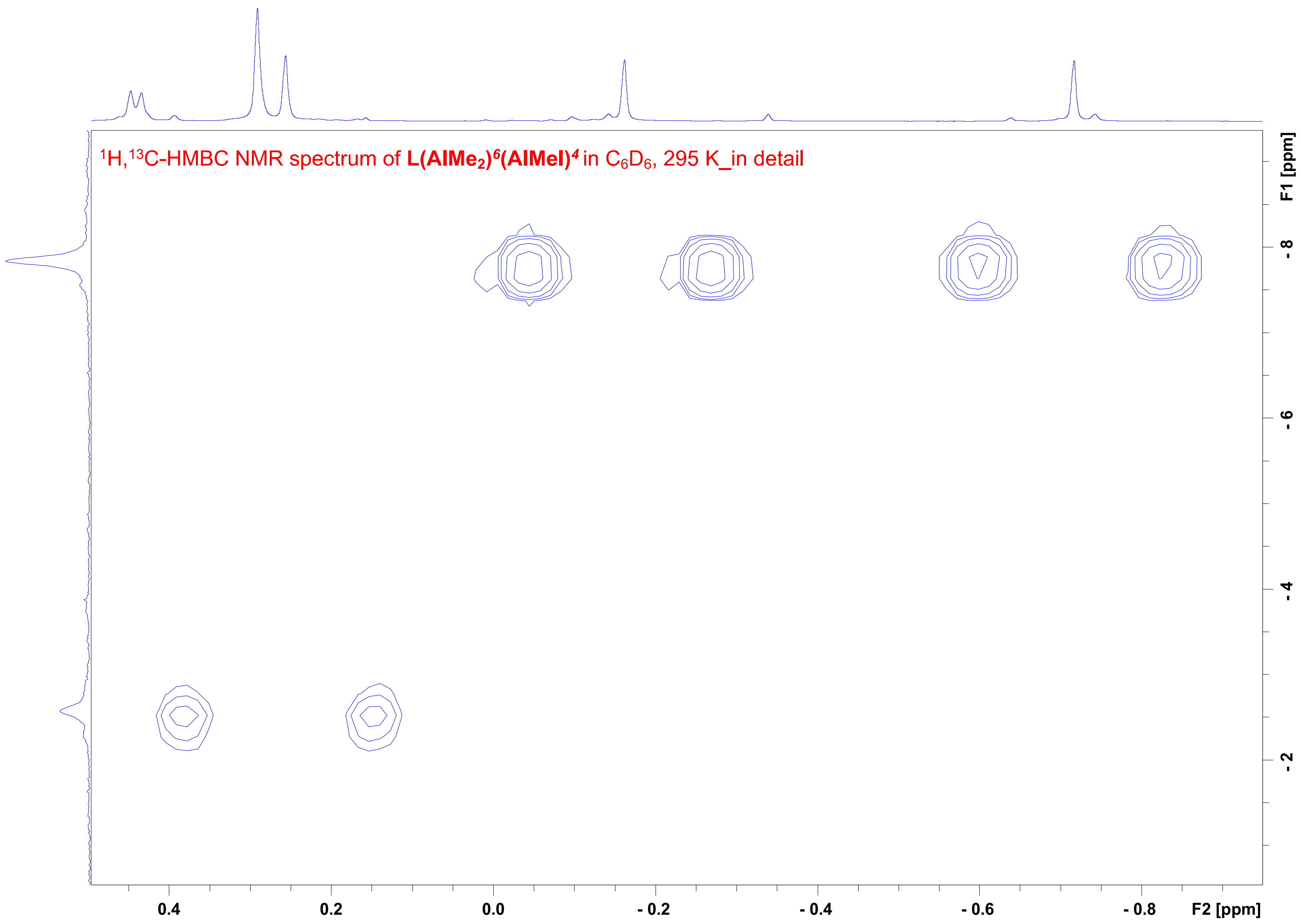

1H,13C-HMBC NMR spectrum of L(AlMe2)6(AlMeI)4 in C6D6, 295 K_in detail
F1 [ppm]
8
6
4
2
0.4
0.2
0.0
- 0.2
- 0.4
- 0.6
- 0.8
F2 [ppm]

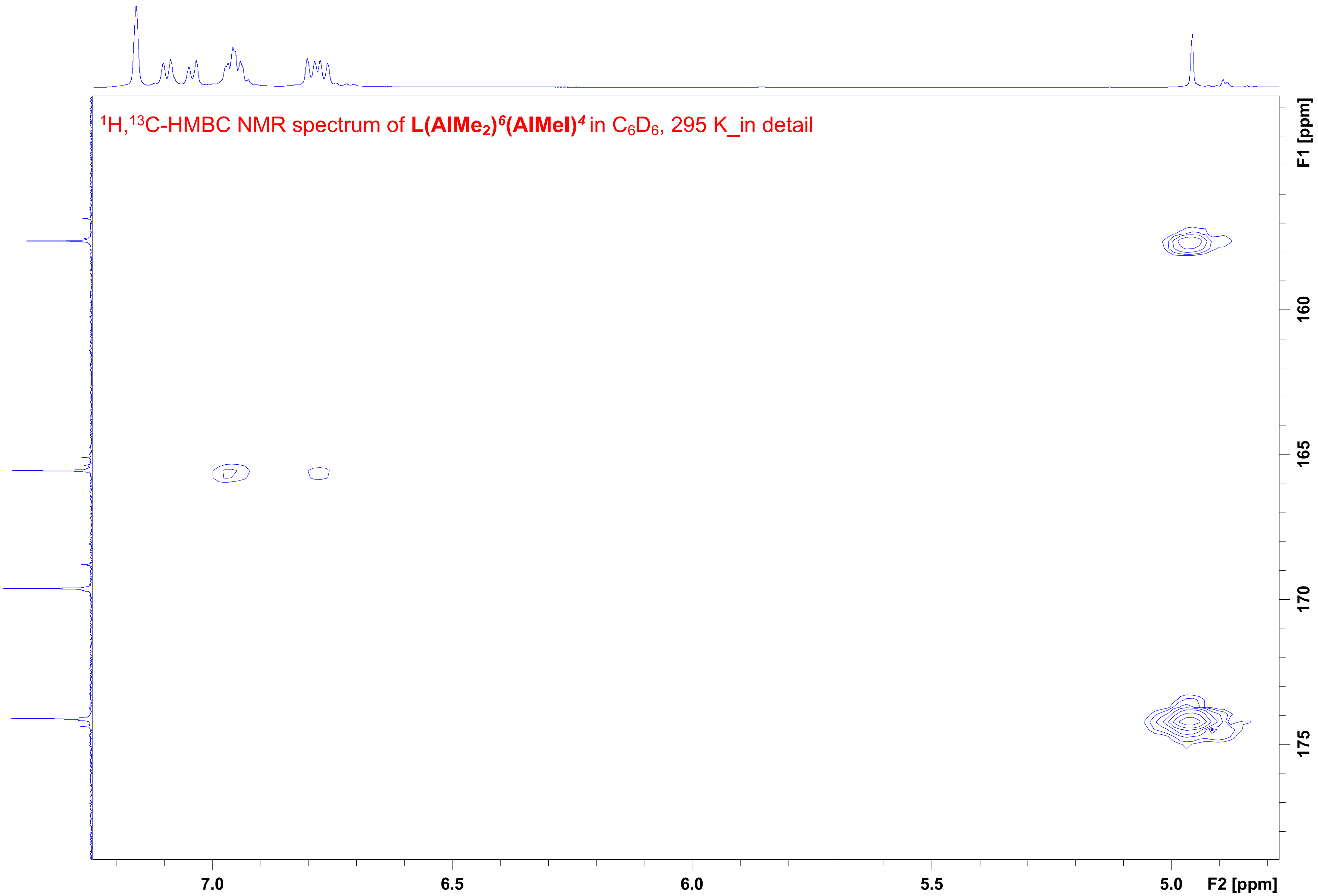

$^{1}H,^{13}C$-HMBC NMR spectrum of **L(AlMe$_2$)^6(AlMeI)^4** in C_6D_6, 295 K_in detail
F1 [ppm]
160
165
170
175
7.0
6.5
6.0
5.5
5.0
F2 [ppm]

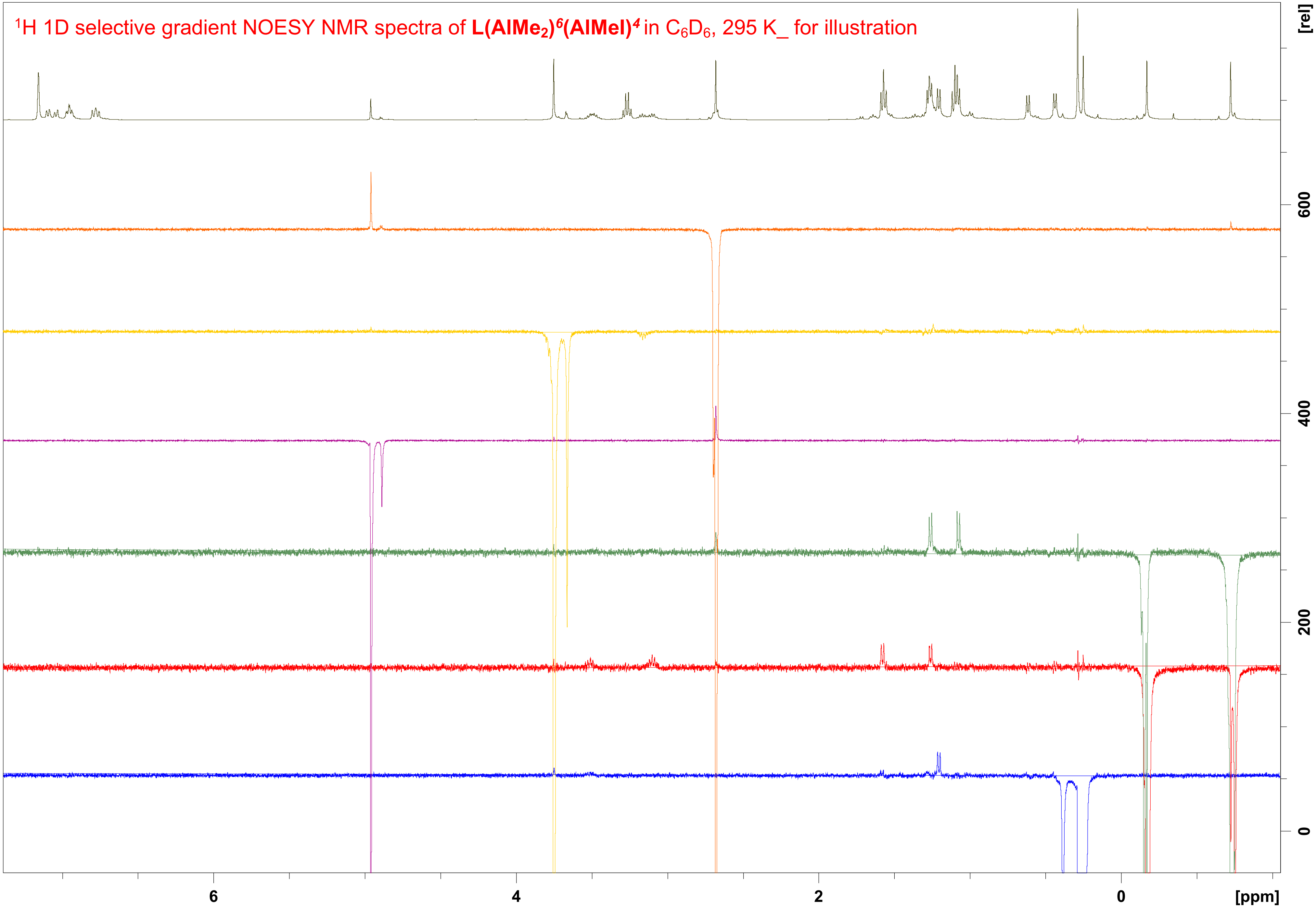

^{1}H 1D selective gradient NOESY NMR spectra of **$L(AlMe_2)^6(AlMeI)^4$** in C_6D_6, 295 K_ for illustration

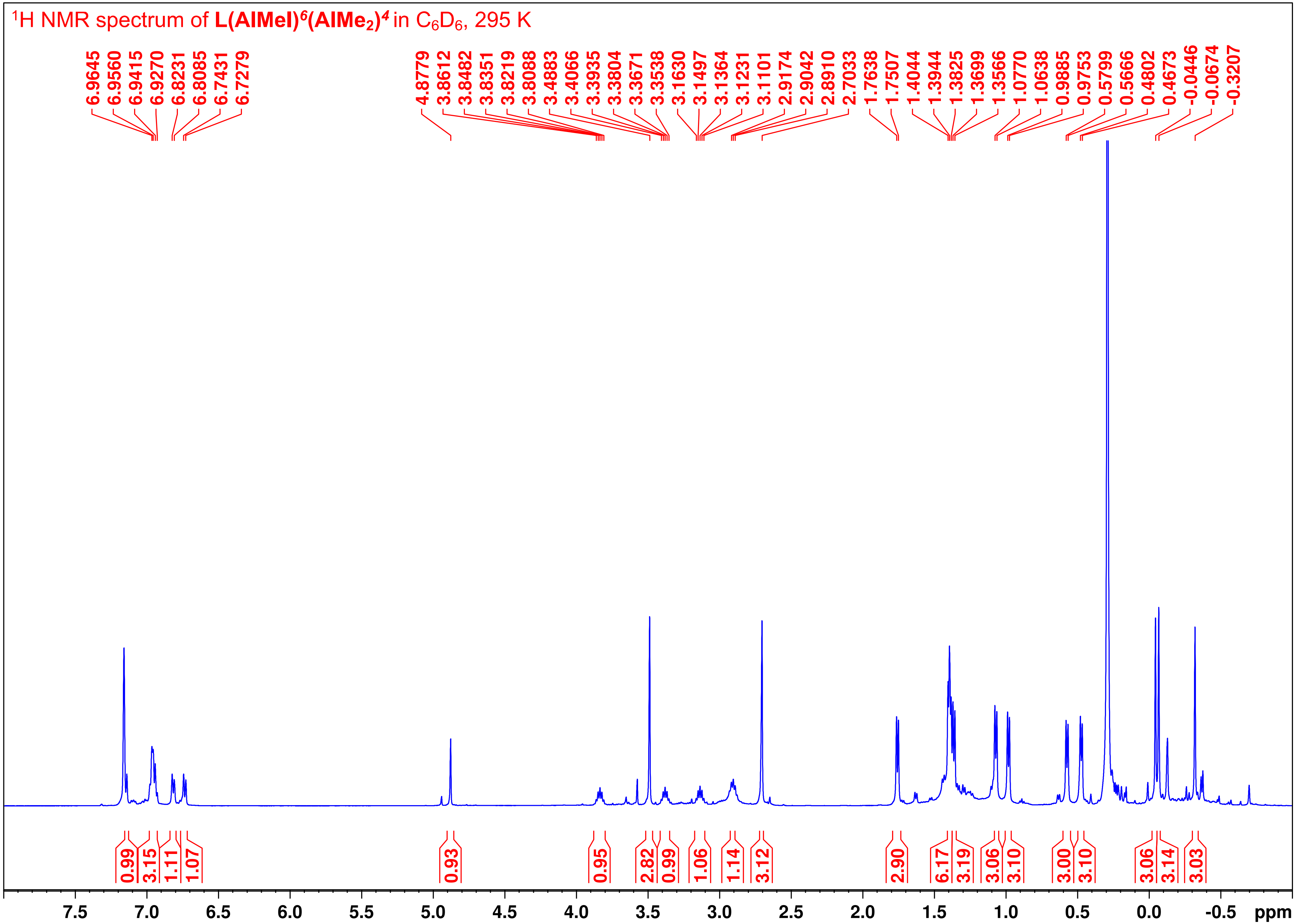

^1H NMR spectrum of $\mathbf{L(AlMeI)^6(AlMe_2)^4}$ in C_6D_6, 295 K
6.9645
6.9560
6.9415
6.9270
6.8231
6.8085
6.7431
6.7279
4.8779
3.8612
3.8482
3.8351
3.8219
3.8088
3.4883
3.4066
3.3935
3.3804
3.3671
3.3538
3.1630
3.1497
3.1364
3.1231
3.1101
2.9174
2.9042
2.8910
2.7033
1.7638
1.7507
1.4044
1.3944
1.3825
1.3699
1.3566
1.0770
1.0638
0.9885
0.9753
0.5799
0.5666
0.4802
0.4673
-0.0446
-0.0674
-0.3207
0.99
3.15
1.11
1.07
0.93
0.95
2.82
0.99
1.06
1.14
3.12
2.90
6.17
3.19
3.06
3.10
3.00
3.10
3.06
3.14
3.03
7.5
7.0
6.5
6.0
5.5
5.0
4.5
4.0
3.5
3.0
2.5
2.0
1.5
1.0
0.5
0.0
-0.5
ppm

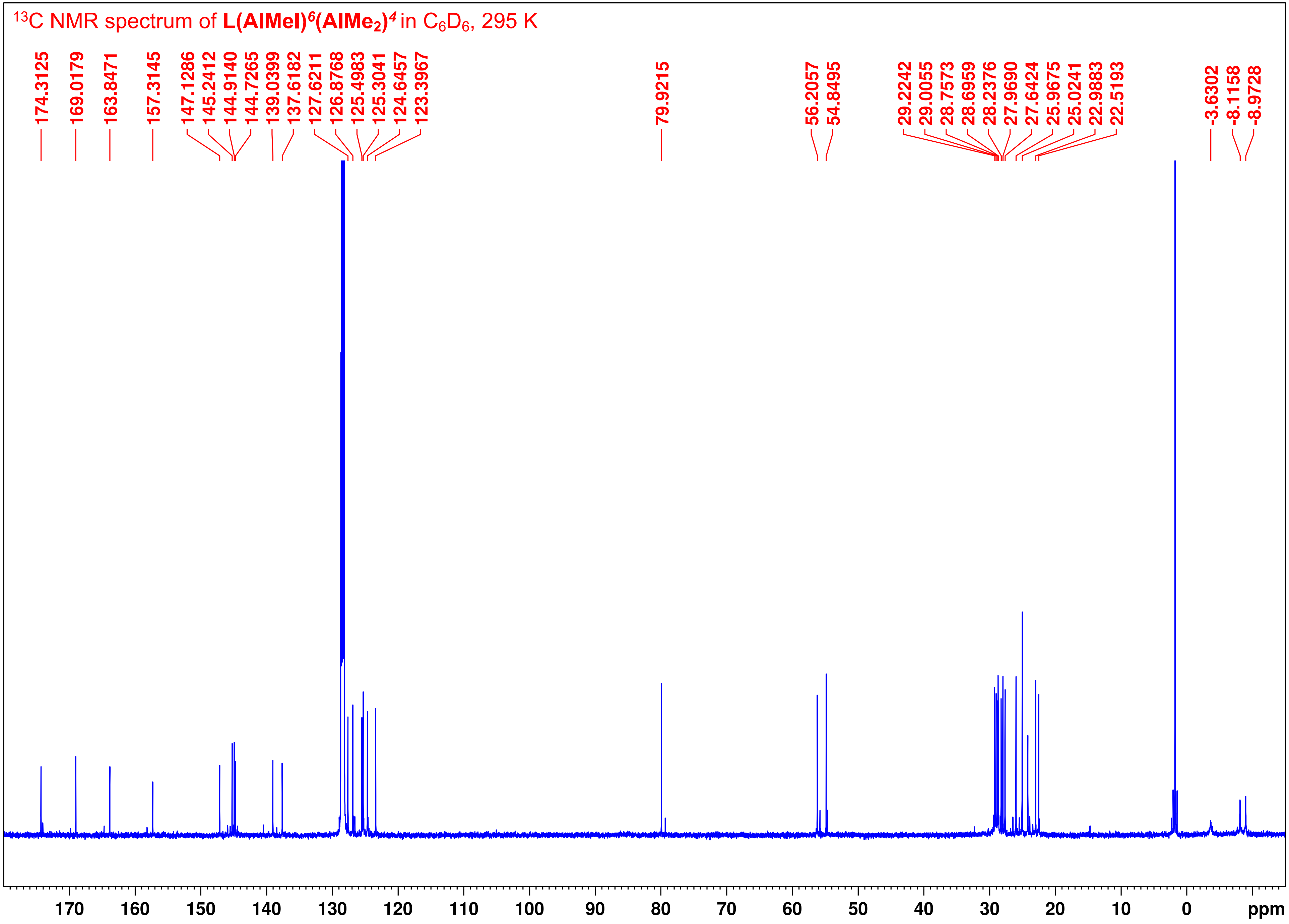

^{13}C NMR spectrum of **L(AlMeI)^{6}(AlMe$_2$)^{4}** in C_6D_6, 295 K
174.3125
169.0179
163.8471
157.3145
147.1286
145.2412
144.9140
144.7265
139.0399
137.6182
127.6211
126.8768
125.4983
125.3041
124.6457
123.3967
79.9215
56.2057
54.8495
29.2242
29.0055
28.7573
28.6959
28.2376
27.9690
27.6424
25.9675
25.0241
22.9883
22.5193
-3.6302
-8.1158
-8.9728
170 160 150 140 130 120 110 100 90 80 70 60 50 40 30 20 10 0 ppm

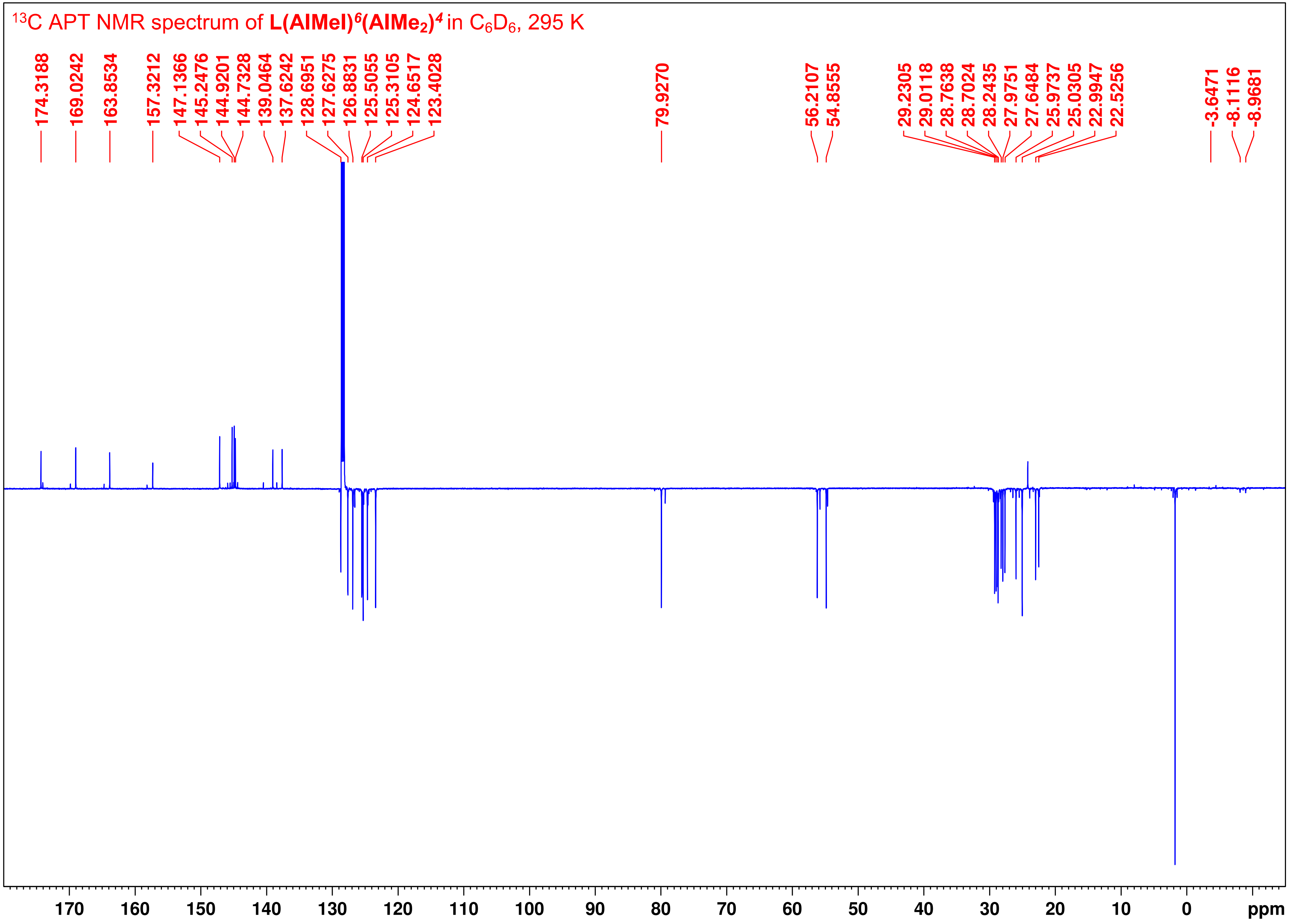

^{13}C APT NMR spectrum of **L(AlMeI)⁶(AlMe₂)⁴** in C_6D_6, 295 K
174.3188
169.0242
163.8534
157.3212
147.1366
145.2476
144.9201
144.7328
139.0464
137.6242
128.6951
127.6275
126.8831
125.5055
125.3105
124.6517
123.4028
79.9270
56.2107
54.8555
29.2305
29.0118
28.7638
28.7024
28.2435
27.9751
27.6484
25.9737
25.0305
22.9947
22.5256
-3.6471
-8.1116
-8.9681
170
160
150
140
130
120
110
100
90
80
70
60
50
40
30
20
10
0
ppm

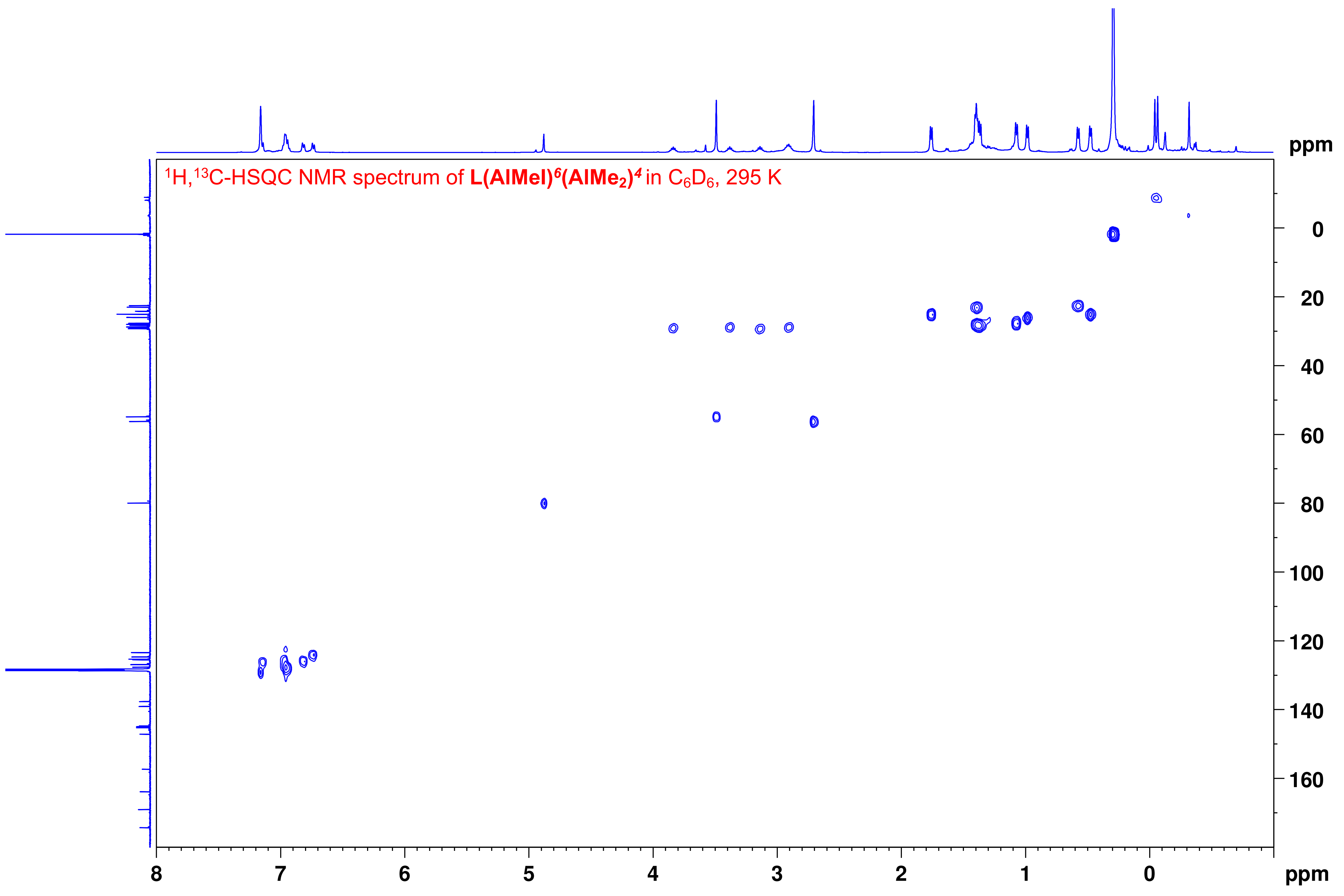

$^{1}H,^{13}C$-HSQC NMR spectrum of **L(AlMeI)^{6}(AlMe$_2$)^{4}** in C_6D_6, 295 K
ppm
0
20
40
60
80
100
120
140
160
8
7
6
5
4
3
2
1
0
ppm

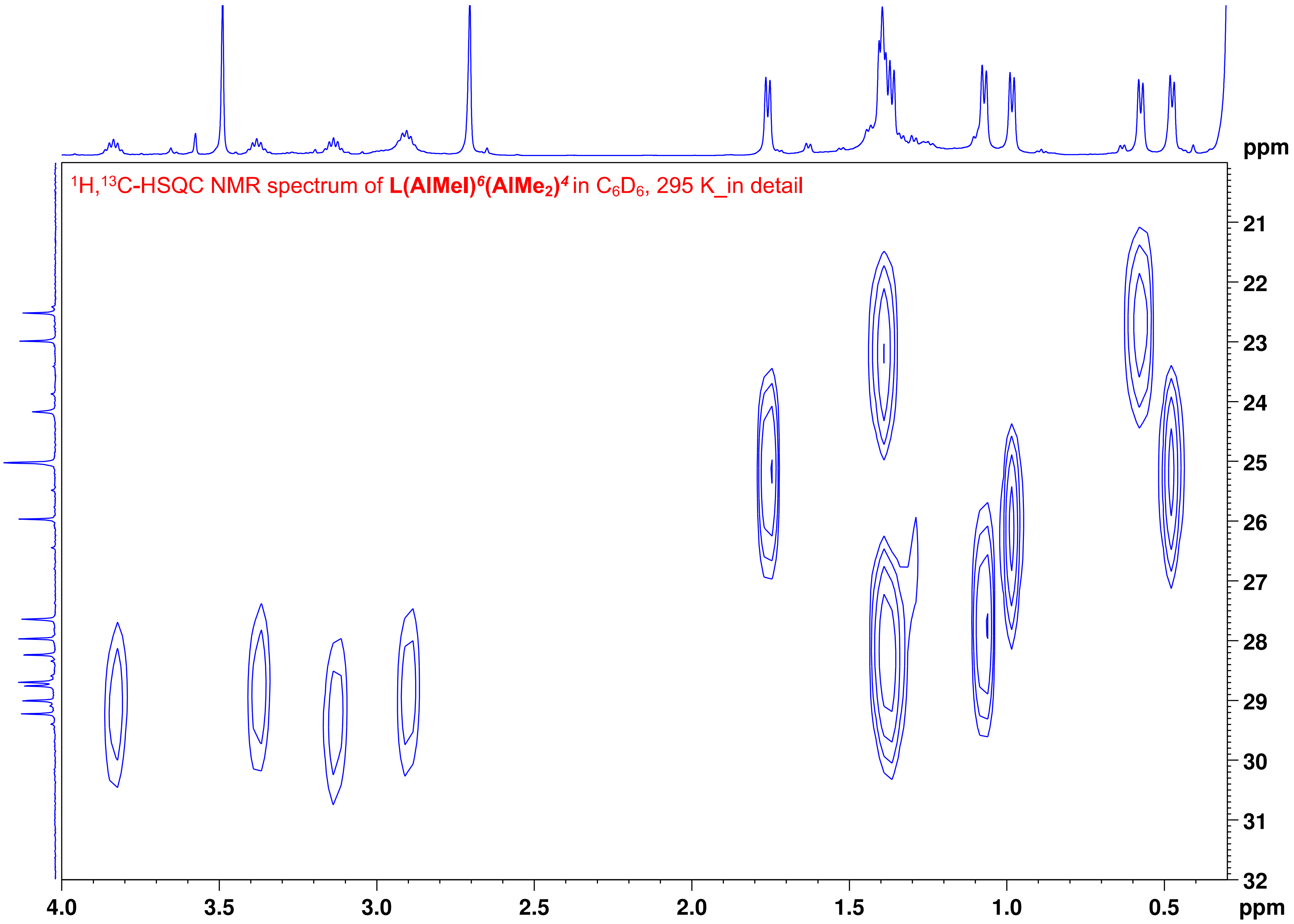
^{1}H,^{13}C-HSQC NMR spectrum of **L(AlMeI)[6](AlMe$_2$)[4]** in C_6D_6, 295 K_in detail
ppm
21
22
23
24
25
26
27
28
29
30
31
32
4.0
3.5
3.0
2.5
2.0
1.5
1.0
0.5
ppm

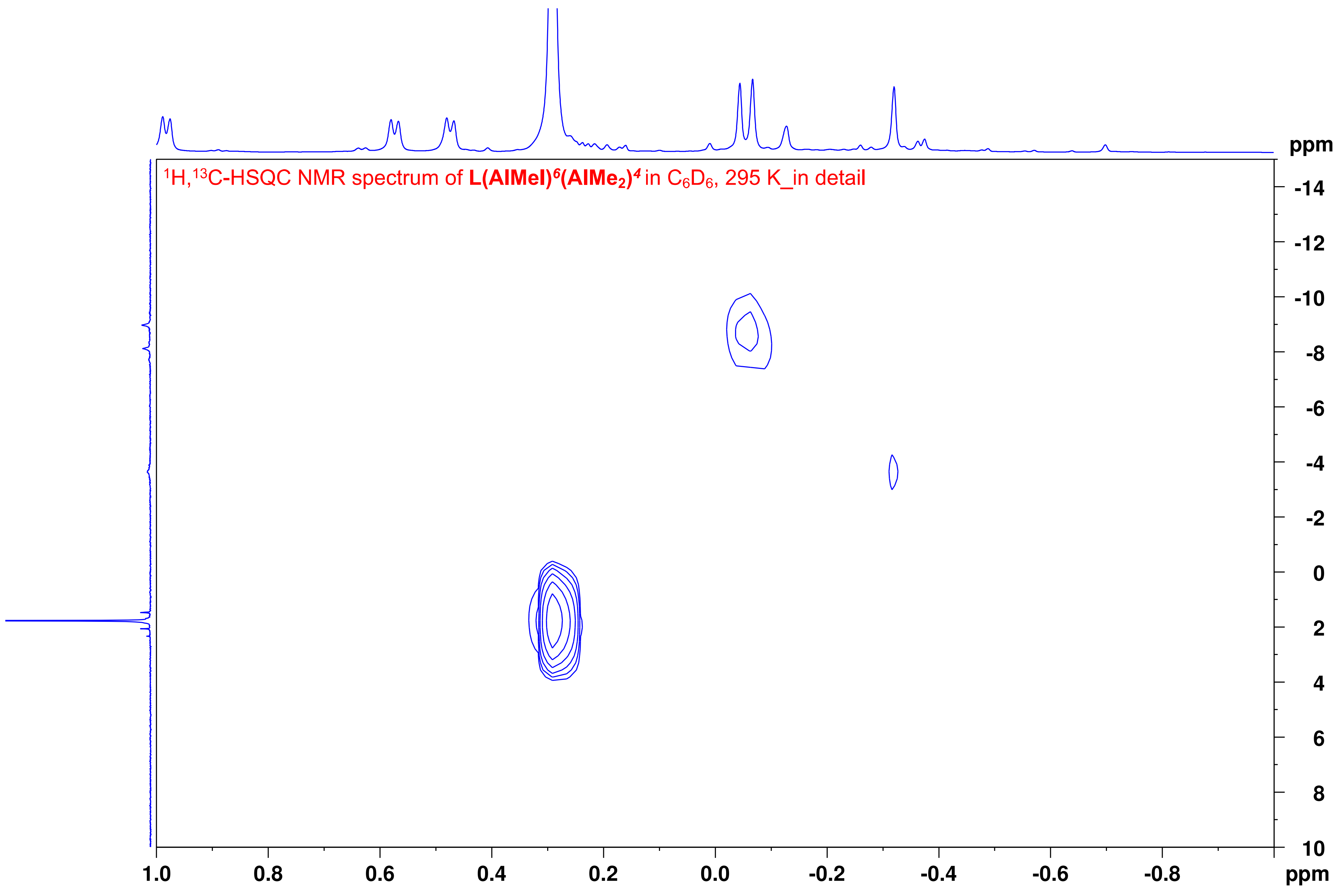

$^{1}H,^{13}C$-HSQC NMR spectrum of **L(AlMeI)^{6}(AlMe$_2$)^{4}** in C_6D_6, 295 K_in detail
ppm
-14
-12
-10
-8
-6
-4
-2
0
2
4
6
8
10
1.0
0.8
0.6
0.4
0.2
0.0
-0.2
-0.4
-0.6
-0.8
ppm

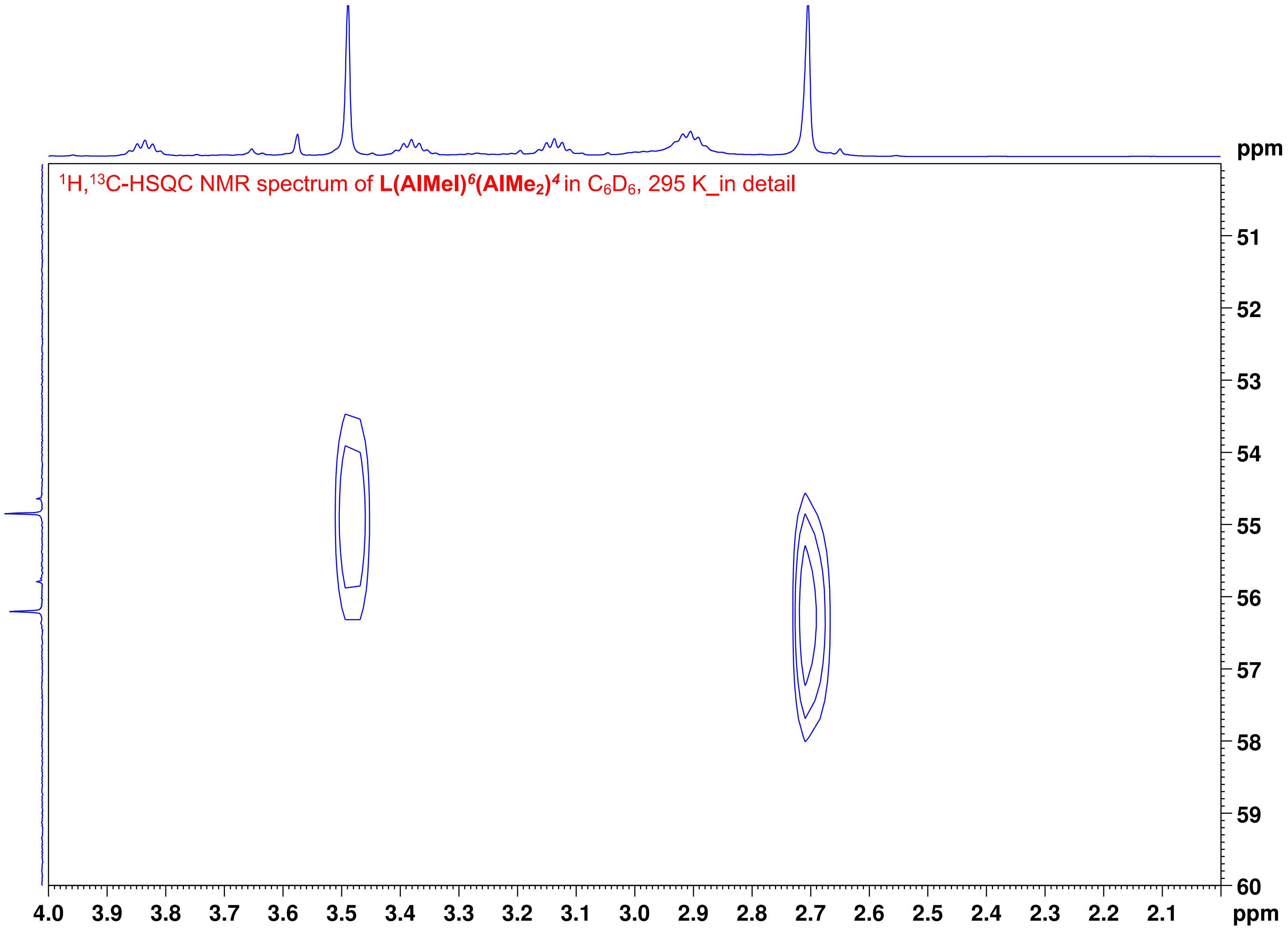

^{1}H,^{13}C-HSQC NMR spectrum of **L(AlMeI)^{6}(AlMe$_2$)^{4}** in C_6D_6, 295 K_in detail
ppm
51
52
53
54
55
56
57
58
59
60
4.0
3.9
3.8
3.7
3.6
3.5
3.4
3.3
3.2
3.1
3.0
2.9
2.8
2.7
2.6
2.5
2.4
2.3
2.2
2.1
ppm

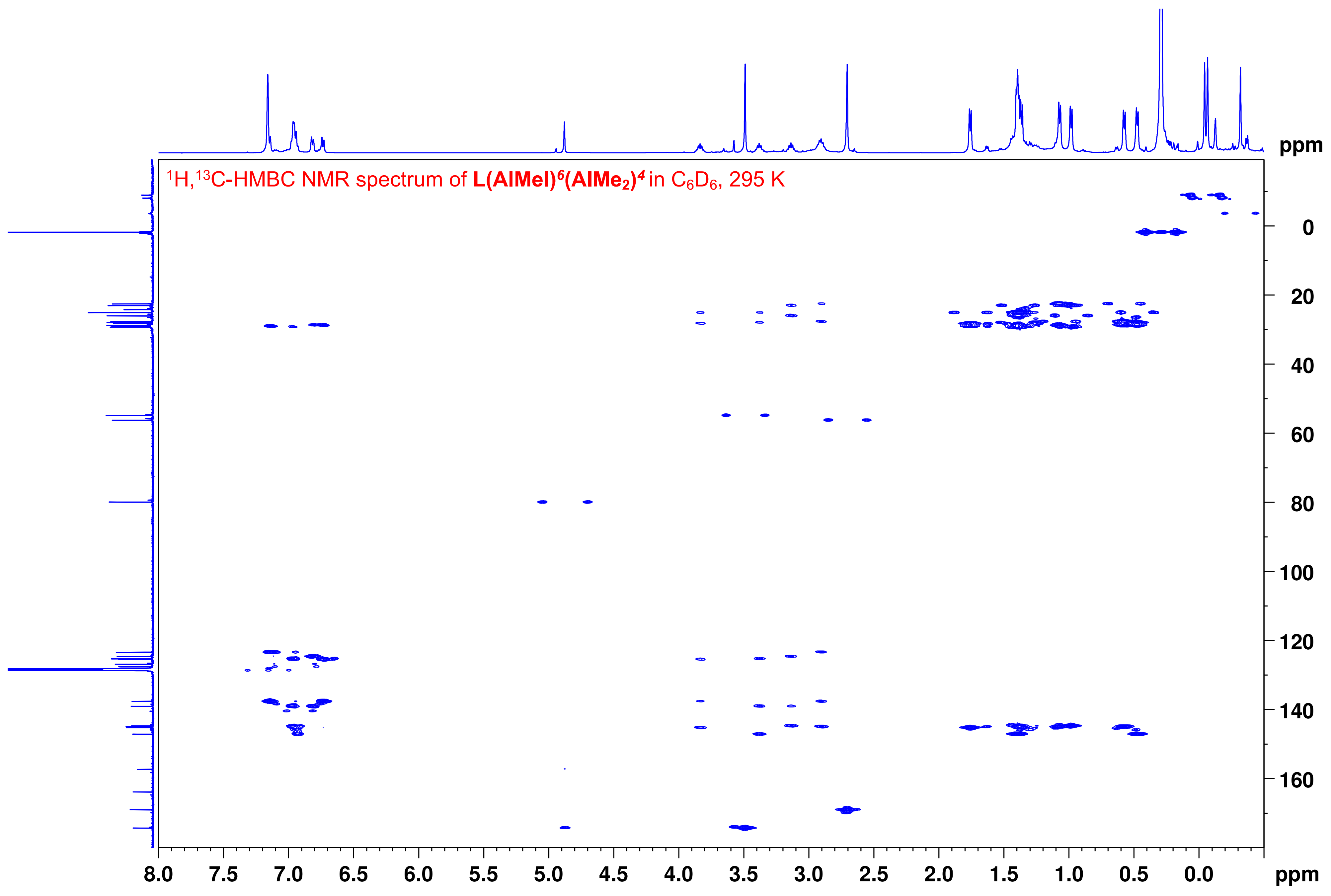

$^{1}H,^{13}C$-HMBC NMR spectrum of **L(AlMeI)^{6}(AlMe$_2$)^{4}** in C_6D_6, 295 K
ppm
0
20
40
60
80
100
120
140
160
8.0
7.5
7.0
6.5
6.0
5.5
5.0
4.5
4.0
3.5
3.0
2.5
2.0
1.5
1.0
0.5
0.0
ppm

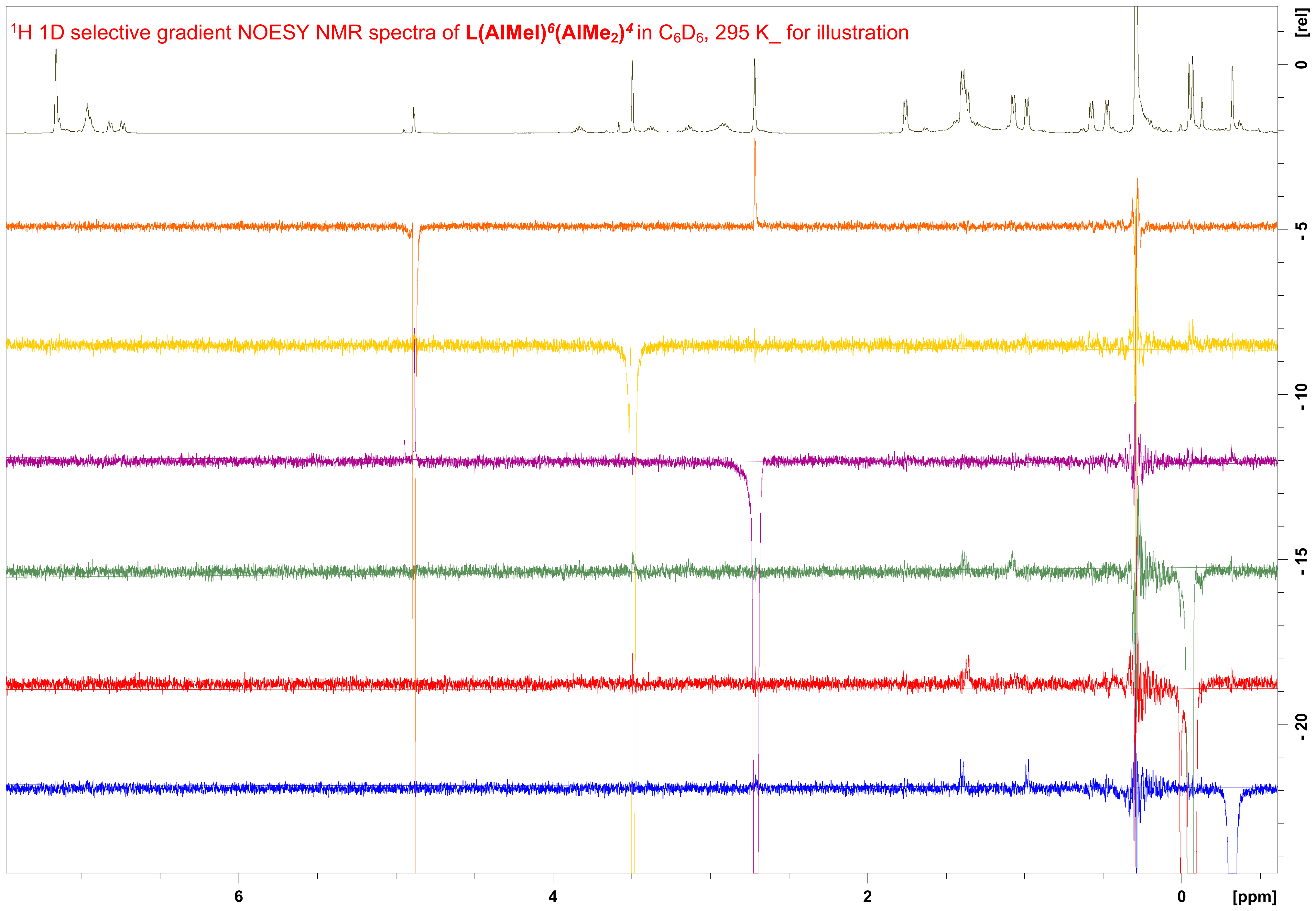

^{1}H 1D selective gradient NOESY NMR spectra of **$L(AlMeI)^{6}(AlMe_2)^{4}$** in C_6D_6, 295 K_ for illustration
[rel]
0
- 5
- 10
- 15
- 20
6
4
2
0
[ppm]
